# Discordance between HIV-1 Population in Plasma at Rebound after Structured Treatment Interruption and Archived Provirus Population in Peripheral Blood Mononuclear Cells

**DOI:** 10.1128/spectrum.01353-22

**Published:** 2022-06-14

**Authors:** Chynna M. Hendricks, Melanie N. Cash, Massimiliano S. Tagliamonte, Alberto Riva, Christian Brander, Anuska Llano, Marco Salemi, Mario Stevenson, Carla Mavian

**Affiliations:** a Emerging Pathogens Institute, University of Florida, Gainesville, Florida, USA; b Department of Pathology, Immunology and Laboratory Medicine, University of Florida, Gainesville, Florida, USA; c Interdisciplinary Center for Biotechnology Research, University of Florida, Gainesville, Florida, USA; d Department of Medicine, University of Miami, Miller School of Medicine, Miami, Florida, USA; e Division of Infectious Diseases, University of Miami, Miller School of Medicine, Miami, Florida, USA; f Department of Microbiology and Immunology, University of Miami, Miller School of Medicine, Miami, Florida, USA; g Hospital Universitari Germans Trias i Pujol, Badalona, Spain; Kumamoto University

**Keywords:** HIV-1, reservoir, therapy interruption, rebound, PBMCs, plasma, persistence, reservoirs

## Abstract

Antiretroviral therapy (ART) can sustain the suppression of plasma viremia to below detection levels. Infected individuals undergoing a treatment interruption exhibit rapid viral rebound in plasma viremia which is fueled by cellular reservoirs such as CD4^+^ T cells, myeloid cells, and potentially uncharacterized cellular sources. Interrogating the populations of viruses found during analytical treatment interruption (ATI) can give insights into the biologically competent reservoirs that persist under effective ART as well as the nature of the cellular reservoirs that enable viral persistence under ART. We interrogated plasma viremia from four rare cases of individuals undergoing sequential ATIs. We performed next-generation sequencing (NGS) on cell-associated viral DNA and cell-free virus to understand the interrelationship between sequential ATIs as well as the relationship between viral genomes in circulating peripheral blood mononuclear cells (PBMCs) and RNA from rebound plasma. We observed population differences between viral populations recrudescing at sequential ATIs as well as divergence between viral sequences in plasma and those in PBMCs. This indicated that viruses in PBMCs were not a major source of post-ATI viremia and highlights the role of anatomic reservoirs in post-ATI viremia and viral persistence.

**IMPORTANCE** Even with effective ART, HIV-1 persists at undetectable levels and rebounds in individuals who stop treatment. Cellular and anatomical reservoirs ignite viral rebound upon treatment interruption, remaining one of the key obstacles for HIV-1 cure. To further examine HIV-1 persistence, a better understanding of the distinct populations that fuel viral rebound is necessary to identify and target reservoirs and the eradication of HIV-1. This study investigates the populations of viruses found from proviral genomes from PBMCs and plasma at rebound from a unique cohort of individuals who underwent multiple rounds of treatment interruption. Using NGS, we characterized the subtypes of viral sequences and found divergence in viral populations between plasma and PBMCs at each rebound, suggesting that distinct viral populations appear at each treatment interruption.

## INTRODUCTION

HIV-1 persists in the face of effective combination antiretroviral therapy (cART), and those reservoirs that sustain HIV-1 persistence pose obstacles to the eradication of HIV-1. Following interruption of cART, these reservoirs fuel viral recrudescence (1–3). Most reservoir studies best characterize latently infected resting memory CD4^+^ T cells as HIV reservoirs capable of producing infectious virus following proviral reactivation (4–8). Several different cellular sources have been implicated in fueling rebounding viremia post-analytical treatment interruption (ATI), including integrated intact proviral genomes, transcriptionally active reservoirs, and expanded proviruses (9–13). Resting memory CD4^+^ T cell reservoirs have been found to contain up to 95% aberrant, non-replication-competent proviruses (14–17). A small fraction of peripheral blood mononuclear cells (PBMCs) contain transcriptionally active proviruses with few HIV-1 proviral-intact PBMCs able to reactivate expression of viral mRNA, suggesting that HIV-1-infected PBMCs contribute a small portion of viral rebound in plasma (18, 19). Several studies have shown that a fraction of viral variants, detected either in residual viremia during therapy (20–22) or at rebound after treatment interruption (23), are genetically distinct from virus present in T-cell subsets, supporting the role of additional cellular or anatomic reservoirs in viral persistence (24, 25). Other cell populations, such as myeloid cells, may also contribute to viral persistence and rebound in individuals on suppressive ART (24, 26, 27). Latently infected CD4^+^ T cells, circulating monocytes, and macrophages (27–32) have been shown to harbor both T-cell-tropic and macrophage-tropic viruses (26), leading to rebounding viremia after ATI (6, 33). Analysis of the composition of rebounding viruses following treatment interruption provides an unbiased way to learn more about the nature of the reservoirs that persist under ART. ATI provides an unbiased window into the dynamics of active reservoirs and what fuels recrudescing viremia.

Many studies examining the origin of recrudescing viremia after structured treatment interruption (STI) have focused on characterizing proviruses in PBMCs, predominantly in circulating CD4^+^ T cells. Populations of virus with identical sequences emerge in plasma after several years on suppressive cART (20, 34), originating from HIV-infected CD4^+^ T cells that cause persistent plasma viremia (35), as well as from blood CD14^+^ monocytes (28, 30–32). When comparing viral populations, discordance between ancestral proviral and viral plasma sequences has been reported when looking at *gag* (36), *pol* (37–39), *int* (38, 39), and *env* (35). This discordance found among viremia populations and proviruses found in cellular reservoirs suggests that perhaps there is an unknown source capable of fueling viral rebound in infected individuals off cART.

We exploited unique individuals who underwent sequential ATI to further investigate the potential reservoirs that drive rebounding viremia in plasma. We assessed whether HIV-1 viral rebound is fueled by a known existing population of proviral-intact PBMCs or from a new source at each ATI. We evaluated genetic concordance, or discordance, between the viral genomes that were found in plasma after ATI and the archival proviral genomes integrated in PBMCs, using deep sequencing techniques on longitudinal samples obtained from individuals undergoing sequential STI. Investigating the nature of these two viral populations is needed to further understand the origin and maintenance of HIV-1 reservoirs under effective ART.

## RESULTS

### Subtype discordance between HIV rebound viremia and proviral population.

This cohort (40) comprised 12 subjects that underwent guided treatment interruptions (GTI) between 1999 and 2001 in IrSiCaixa, Barcelona, Spain (Table 1). We analyzed 9 individuals that had two or more years of continuous ART, undetectable viral loads for at least 2 years, and a CD4/CD8 ratio of >1 for a minimum of 6 months before undergoing 3 or more interrupted highly active retroviral therapy treatments. These 9 individuals were enrolled in the arm of the study that had 3 or more subsequent GTI in a structured course. The subjects’ cART regimens were resumed when plasma viral loads reached >3,000 copies/mL at two consecutive time points or after 30 days, which was continued for 90 days before the next scheduled STI.

**TABLE 1:** Patient info^*a*^

Patient	NHC	Sex at birth	Birth region^*b*^	Race or ethnicity^*c*^	Transmission category	Yr of Dx	Subtype RNA	Subtype DNA
VBP2	264706	F	NA	Caucasian	IDU	1986	B	B, C, BC?
PCG3	245157	F	NA	National surname	HET	1992	B	B
SDS4	128466	F	NA	Caucasian	HET	1991	B	B
CGD5	295688	F	NA	Caucasian	IDU	1991		28_BF, 02_AG
EHD6	208223	F	NA	Caucasian	HET	1995	B	08_BC, 07_BC, C, BC?
SMR7	272070	F	NA	National surname		1996		08_BC, 07_BC, C, BC?
MMG8	292142	M	NA	Caucasian	HET	1996	28_BF	B
JOR10	148461	M	NA	Caucasian	MSM	1990	B	B, C, BC?
NTU12	250918	F	NA	Caucasian	IDU	1986	B, BF?	07_BC, C

a RNA type is the viral population at rebound in plasma; DNA type is the proviral population in PBMCs. For both the RNA and DNA types, question marks indicate a probable subtype. M/F, male/female; HET, heterosexual; Dx, diagnosis; probable subtype.

b Birth region information was not available (NA), but because all patients have national surnames, birth regions are most likely all national people, but this cannot be confirmed.

c Based on the national surnames, patient race and ethnicity is most likely Caucasian but cannot be confirmed.

For each patient, we amplified the V3 region of the *env* gene sequence (bp 811 to 1010 as of HBX2) from both cell-free virions from plasma and provirus from PBMCs from longitudinal time points during the rise, peak, or decay of viremia across sequential STI for each patient (see Table S1 in the supplemental material). We were able to extract and obtain RNA and DNA amplicons, from plasma and PBMC samples, respectively, from all the patients. During an initial genomic low-coverage screening, we detected HIV-1 sequences only from a subset of successfully amplified amplicons (see Table S1). Among the subjects and their time points that were successfully sequenced, we found that seven of the nine patients presented discordant populations of virus at rebound and in the reservoir (Table 1). For the two patients with concordant populations, patients PCG3 and SDS4, both viral and proviral populations were subtype B (Table 1).

Based on BLASTn analysis, we determined that the envelope region obtained from RNA from virions in plasma and DNA from provirus intact in PBMCs from the other seven patients were not the same subtype (Table 1; see also Table S1 in the supplemental material). In patient VBP2, the “best hit,” as defined below in Materials and Methods, for the sequences obtained from PBMCs was subtype C (similar to a sequence in the database isolated in India in 1993), while sequences retrieved from plasma samples from this patient all belonged to subtype B (Canada 1997). Similarly, for patient JOR10, proviral DNA sequences belonged to subtype C (India 1993), while the serotype found in the rebound viremic population was subtype B (Netherlands 1986 or France 1992) (Table 1; see also Table S2 in the supplemental material). Sequences from subtypes B and C were confirmed by the tool COMET (context-based modeling for expeditious typing); however, because we are only looking at a small region of *env*, we do not know whether the patients harbor subtype B, C, or a BC recombinant form.

For patient CGD5, the “best hit” for the DNA sequences obtained from PBMCs were heterogenous in subtype C (India 1993 or 1997), B (USA 1979, Germany 1986, or Canada 1997), and recombinant forms 28_BF (Brazil 2000) or 02_AG (Guinea-Bissau 2014), while sequences obtained from virions in plasma were not long enough for subtype identification (<150 bp). For patient EHD6, sequences obtained from PBMCs were like recombinant forms 08_BC (China 1996 or 1998) or 07_BC (China 2007) or like subtype C (India 1993), while plasma sequences belonged to subtype B (USA 2011). For patient SMR7, proviral sequences resembled recombinant forms 08_BC (China 1996, 1998, 2007, or 2009) or 07_BC (China 2007) or subtype C (India 1993, 1995, or 1997), with no sequence longer than 150 bp. In patient MMG8, proviral sequences were similar to subtype B (USA 1986), while plasma sequences were similar to 28_BF (Brazil 2000). Lastly, for patient NTU12, proviral sequences either belonged to recombinant form 07_BC (China 2007) or to subtype C (India 1993), with plasma RNA sequences resembling subtype B (USA 1986 or Germany 2011). For diverse reasons, these patients (CGD5, EHD9, SMR7, MMG8, and NTU12) were not used in further analyses because the sequences were either too divergent to be aligned, the lengths of the reads were not long enough to allow for phylogenetic analysis, or we were able to retrieve information from only one compartment (either plasma or PBMCs).

### Characteristics of plasma haplotypes and PBMC sequences in VBP2, PCG3, SDS4, and JOR10.

We performed a deep-coverage run to obtain a comprehensive view of the low-frequency variants found in reservoirs and rebound viremia populations for patients that had remaining PBMCs or from whom we had obtained long HIV-1 sequence reads. For these four patients, VBP2, JOR10, PCG3, and SDS4, we successfully sequenced matching HIV-1 subtypes (see Table S1 in the supplemental material), and we combined the results from the first low-coverage screening run (see Table S2) with the second deep-coverage sequencing run (see Table S3). Although we identified subtype discordance in patients VBP2 and JOR10 in the first run, these patients showed subtype B proviral sequences matching the subtype of the virions in plasma in the second run (Table 1). Therefore, we proceeded to investigate the characteristics of VBP2, PCG3, SDS4, and JOR10.

Because defective proviral genomes typically show major defects, such as an APOBEC-mediated hypermutation that results in premature stop codons or missense mutations, we checked for APOBEC-mediated hypermutation before analyzing the genetic sequences and found no such hypermutations among the sequences. We also eliminated sequences with stop codons, representing 1.8% of sequences in VBP2, 2.4% in PCG3, 2.6% in SDS4, and 0% JOR10, excluding the possibility that the mutations were due to sequencing errors (the error rate for Illumina platforms is less than 0.5%) (41).

In patient VBP2, the more abundant haplotypes were found in plasma at the time point 299 days post-first STI (one haplotype with 42,002 reads), time points showing clonal expansion (see Fig. S1 in the supplemental material), and at the time point 672 days (two haplotypes with 5,025 and 2,582 reads) (see Table S4); sequences in PBMCs had a minimum of 5 to a maximum of 12 reads (see Table S4).

We retrieved many sequences from patient PCG3, and several abundant haplotypes were found in plasma at time points that showed clonal expansion (see Fig. S2): at 19 days (nine haplotypes with over 1,000 reads) and at 294 days (one haplotype with 48,564 reads). Sequences from PBMCs had a minimum of 5 reads and a maximum of 28,513 reads (see Table S4).

Similarly, for patient SDS4, we also obtained an in-depth characterization of proviral and viral populations, finding clonal expansion at several time points (see Fig. S3 in the supplemental material). Over 200 haplotypes from plasma at all time points were composed of more than 1,000 reads each (34 of which had more than 10,000 reads, with a maximum of 78,449 reads), while the PBMC sequences had a minimum of 5 reads to a maximum of 1,785 reads (see Table S4).

Despite the deep-sequencing approach, a small number of HIV-1 reads was obtained from patient JOR10 (total between plasma and PBMCs of 9,448 reads). The most abundant plasma haplotype contained 8,393 reads, comprising 88% of the total reads, and was found at time point 622 days. Both plasma time points in this patient showed clonal expansion (see Fig. S4A and B). A total of 126 reads were recovered from PBMCs and assigned across nine clusters of sequences (minimum of 5 reads to a maximum of 27 reads) (see Table S4).

Intrahost genetic distances were around 5% for all patients when considering only subtype B sequences (see Table S5), which is in line with what has been previously reported for HIV-1 (42). However, for patient JOR10 the intrahost genetic distances were nearly doubled when we considered subtype C sequences in the proviral group (see Table S5). A genetic distance like an inter-subtype distance was also found when we compared the genetic distance between virion and proviral sequences within each patient, indicating that reservoir and viral sequences were genetically distant (see Table S6).

### Bayesian phyloanatomy reveals discordant proviral and rebound viremic populations within the same subtype.

**FIG 1:**
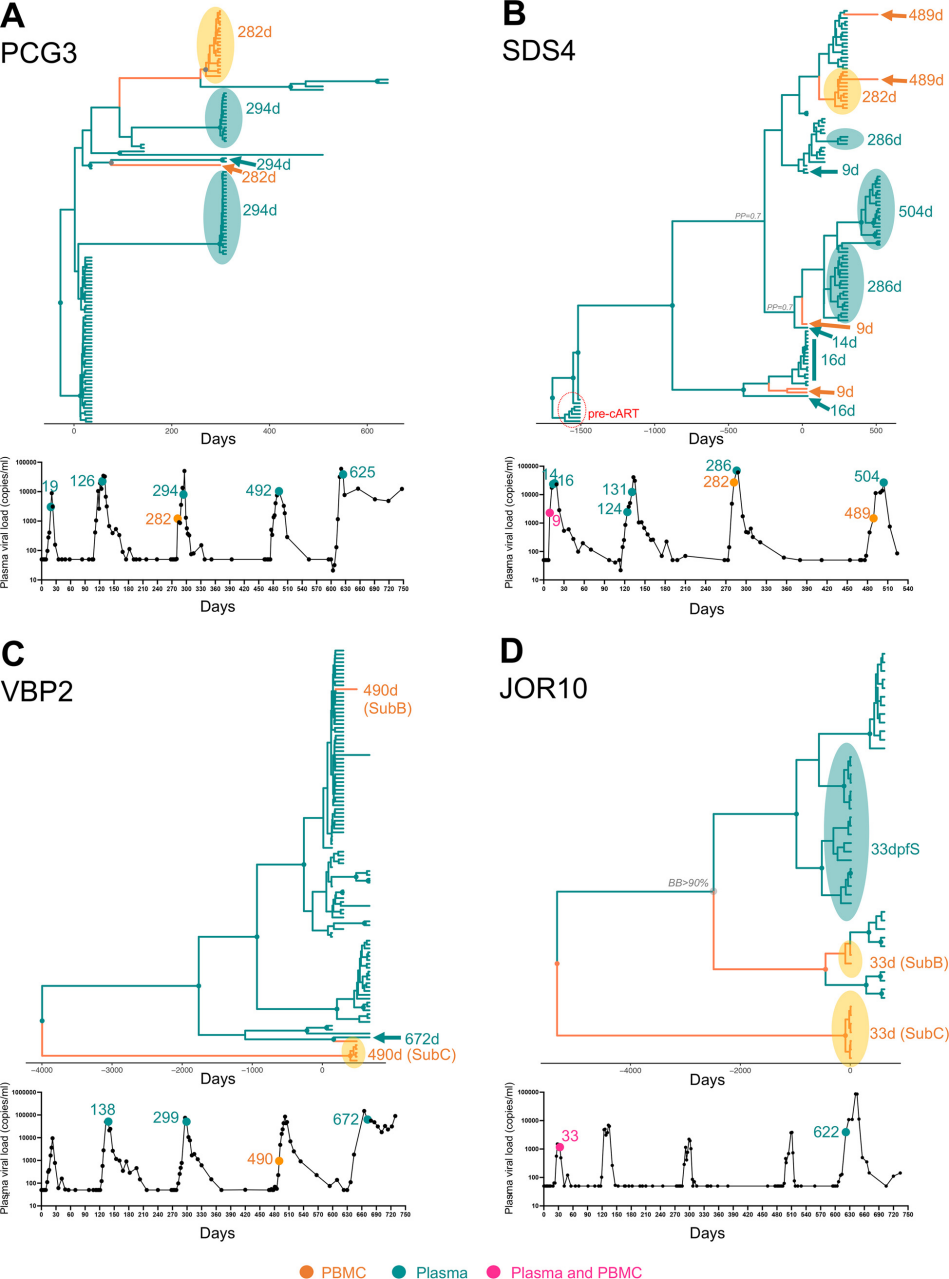
Bayesian phyloanatomy analysis of HIV-1 viral and proviral *env* sequences from four patients sampled during structured therapy interruption. Maximum clade credibility trees inferred from HIV-1 *env* sequences for each patient, PCG3 (A), SDS4 (B), VBP2 (C), and JOR10 (D), were scaled in time (days post-first STI) by enforcing an uncorrelated relaxed molecular clock calibrated for HIV-1 intrahost evolution (see Materials and Methods). Origin (plasma or PBMCs) of the ancestors at nodes was inferred using an asymmetric phylogeographic diffusion model, indicated by branches and internal nodes colored according to origin: green, viral sequences from plasma (RNA); orange, proviral sequences from PBMCs (DNA). Green and orange circles at nodes represent branches supported by PP > 0.9, with green or orange colors representing probability for the ancestor coming from either virion from plasma or provirus from PBMCs. Gray circles indicate PP > 0.8. Under each tree, the viral load curve indicates the viral load at the time point that is shown in the tree. Time points are highlighted as in the tree with green, orange, and purple, indicating a time point at which plasma, PBMCs, or both were sampled and successfully sequenced, respectively.

Next, we explored concordance or discordance of viral and proviral populations in both patients with concordant subtypes (PCG3 and SDS4), as well as in the two patients that showed both concordance and discordance in subtypes (VBP2 and JOR10). After subsampling patients VBP2, PCG3, and SDS4, we optimized viral genome genetic diversity and temporal distribution using TARDIS (43) and controlled for the presence of both phylogenetic signals (see Fig. S5). Therefore, we investigated the phylogenetic relationship and the time of emergence of viral and proviral populations using the Bayesian phyloanatomy framework (24), calibrating the rate for the HIV-1 intrahost evolutionary rate using the rate of 7.53 × 10^−3 ^nucleotide (nt) substitutions/site/year (26, 44) (Fig. 1). For the first patient with complementary subtypes, PCG3, we obtained sequences for the viral population in plasma from each of the five STIs at time points 19, 126, 294, 492, and 625 days, and all the samples were obtained at the rise of the viral load curve, during the expansion of the viral rebounding population, except for the plasma samples at 492 and 625 days, collected at the peak and fall of viremia, respectively (Fig. 1A). Proviral sequences were obtained at one time point during the third STI (282 days), halfway to the peak viral load, 14 days before peaking, and 12 days before the temporally close plasma time point collected at 294 days (Fig. 1). When we look at how proviral and viral populations at 282 and 294 days, respectively, are related, it is evident that they are not closely related (Fig. 1A). The posterior maximum clade credibility (MCC) tree shows a ladder-like shape, typical of intrahost evolution due to high genetic variability required to survive and infect new cells (Fig. 1A). Sequences are compartmentalized both temporally (with large monophyletic clades corresponding to a sampling point) and based on origin, with little or no intermixing of sequences representing the viral or proviral populations. The majority of the proviral sequences collected during the third STI at 282 days clustered in one major monophyletic clade (posterior probability [PP] > 0.8), except for one cluster of sequences (composed of 20 reads) that clustered among haplotypes derived from the plasma population. The large proviral clade is related to a group of sequences from later time points, 294 and 625 days, and related within; in contrasting, the unique proviral sequences (282 days) clustered with two plasma haplotypes obtained at time point 294 days and shared ancestry with the time to the most common ancestor (tMRCA) of 75 days and a 95% high posterior density interval of 41 to 131 days (PP = 0.8). Although these three sequences obtained from the same STI clustered together and showed apparent concordance, the long branch separating the plasma (294 days) and PBMCs (282 days) does not indicate an immediate phylogenetic relationship; rather, it suggests that unsampled intermediate sequences are missing, perhaps from another unsampled anatomical location (cell type or tissue), which may represent the real link between these two compartments. Interestingly, this heterogenous clade connected with one plasma haplotype collected during the first STI at 19 days with a high PP (PP > 0.9), again suggesting a much larger unsampled population connecting these haplotypes. Finally, patient PCG3 also displayed two major monophyletic clades of plasma populations at 294 days (PP = 1, PP = 0.99), thus close to the proviral time point from a temporal point of view; however, these monophyletic clades do not cluster with the proviral sequences that were sampled 12 days before, the time frame usually expected for HIV-1 to rebound with detectable viral load in blood (45–47). To understand whether similar reservoirs were seeding the rebound in different treatment interruptions, we performed compartmentalization analysis between DNA and RNA populations. The patterns observed in the MCC trees were supported by the compartmentalization analysis that detected the presence of moderate to strong population structure (Table 2).

**TABLE 2:** Intrahost compartmentalization analysis of virion (RNA) and provirus (DNA) sequences^*a*^

Patient	Hudson, Slatkin, and Maddison FST	Slatkin FST	Hudson, Boos, and Kaplan FST	Hudson (S_nn)
VBP2 (mixed)	***0.738***	***0.585***	**0.256**	***0.879***
VBP2 (mono)	NS	NS	NS	NS
PCG3	***0.468***	**0.306**	**0.293**	***0.994***
SDS4	**0.234**	0.132	***0.065***	***0.942***
JOR10 (mixed)	***0.696***	***0.533***	***0.411***	***0.938***
JOR10 (mono)	***0.452***	**0.292**	0.071	NS

a FST values in boldface and italics in shaded cells indicate a strong population structure; FST values in boldface but with no shading of cell indicate moderate population structure; FST values without special formatting or shading indicate absence of population structure. For patient VBP2, “mixed” indicates both subtype B and C sequences were present; “mono” indicates only subtype B sequences were present for the test. NS, nonsignificant, i.e., Prob(random FST > observed FST) > 0.05.

For the second patient with matching subtypes, SDS4, we obtained sequences from the viral population in plasma before initiation of cART and from each of the four STIs: three during the expansion of the viral rebounding population at 9, 131, and 286 days; four at the peak of STI viral rebound at 14 and 16 days from the first STI, at 124 days from the second STI; and 504 days from the fourth STI (Fig. 1B). We obtained sequences for the provirus population at three time points during the expansion of the viral rebounding population: one at 9 days from the first STI that matched the time point from plasma; and the other two at 282 and 489 days, corresponding to the third and fourth STI, respectively (Fig. 1B). The MCC tree shows intermixing of proviral and viral sequences, with sequences sampled before initiation of cART as the root of the tree (Fig. 1B). The sequences obtained from virus and provirus during longitudinal sampling of multiple STIs clustered in two large monophyletic clades, one containing a population of virus and proviruses sampled during the first STI and the second containing sequences from all STIs (Fig. 1B). Yet, sequences from virions and proviruses collected at the same time point (9 days) did not cluster together in the tree, with proviral sequences clustering in both major monophyletic clades and plasma haplotypes scattered across the phylogeny. Discordance at the same time point is an indicator that the identified reservoir has not been reactivated and thus is not contributing directly to the HIV-1 population circulating in plasma. The same trend was observed among PBMCs and plasma haplotypes sampled a few days apart within the same STI, such as PBMCs at 282 days and plasma at 286 days or PBMCs at 489 days and plasma at 504 days (Fig. 1B). Overall, this patient exhibited some degree of discordance; however, since the phylogenetic relationship across the clades was not well supported (even in the presence of phylogenetic signal), it is difficult to make a firm conclusion regarding concordance and discordance of this patient. Similarly, the compartmentalization analysis across different tests produced inconclusive results (Table 2).

In VBP2 and JOR10, discordance between populations in plasma and PBMCs was stark not only due to subtype heterogeneity (Fig. 1C). For VBP2, we obtained sequences from the viral population in plasma from three STIs, two at the peak of the STI at 138 and 299 days, corresponding to the second and third STI, respectively, as well as one while viral rebound was declining after the fifth STI at 672 days (Fig. 1C). A provirus population was only obtained from the fourth STI during the expansion of the viral rebounding population at 490 days (Fig. 1C). For patient VBP2, the compartmentalization analysis showed moderate to strong population structure, yet when comparing only subtype B sequences the analysis did not achieve statistical significance (Table 2). For JOR10, we obtained sequences from the viral population in plasma at the peak of viral rebound at 33 days during the first STI, as well as during the expansion of the viral rebounding population at 622 days from the fifth and last STI; provirus population was obtained from the matching plasma population at 33 days (Fig. 1D). Again, we found discordance at the same time point, evidence that the reservoir present in the PBMCs is not contributing to rebound. For patient JOR10, the compartmentalization analysis revealed strong population structure when we compared the data set with subtype B and C proviruses and weak population structure when we compared only subtype B reservoir to viral sequences (Table 2). In conclusion, proviral sequences among the four patients differed from the ones circulating in plasma, suggesting that these proviruses do not represent the immediate ancestor of the virus population found in plasma at rebound. Moreover, a trend observed among all patients was that viral clades from successive time points did not always branch out from the prior viral cluster (Fig. 1). This finding suggests that distinct populations were present at rebound and likely originated from distinct reactivated proviral reservoirs.

**FIG 2:**
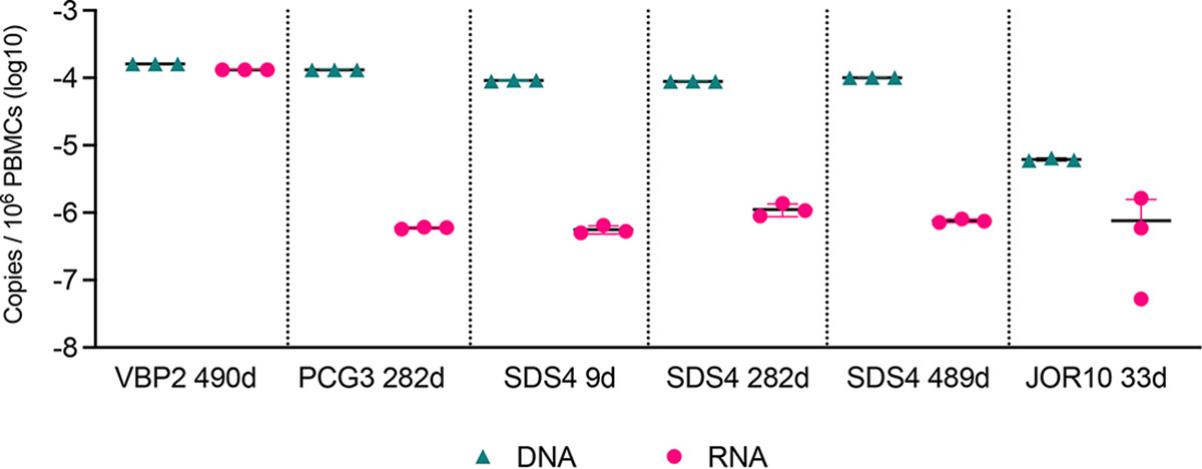
Levels of HIV-1 DNA and RNA in PBMCs. Comparison of cell-associated HIV-1 DNA (green triangles) and RNA (fuchsia circles) in PBMCs among subjects with detectable HIV-1 DNA. Estimates from ddPCR in triplicates are given individually, indicating the mean (black horizontal bar) and the standard deviation (double-lined and colored error bar; visible only for the RNA estimation of the time point 282 days in patient SDS4).

Finally, we determined the size and the transcription competence of the HIV-1 reservoir by estimating the total copies of integrated HIV-1 DNA and cell-associated HIV-1 RNA per cell, respectively, based on the quantification of total DNA and RNA by digital droplet PCR (ddPCR), a technique that offers greater precision, improved reproducibility, with similar sensitivity to quantitative PCR, for HIV-1 reservoir quantification (48). We performed ddPCR on total DNA and RNA extracted from PBMCs of each patient and time point for which we successfully retrieved HIV-1 proviral sequences. As expected, in each patient and time point that was tested, we found presence of a very small HIV-1 reservoir contributing to minimal expression of HIV-1 RNA, indicating that the size of the PBMC reservoir was approximately of 100 per million PBMCs, with approximately one transcriptionally active cell (Fig. 2). The expressed proviral RNA was three times lower than its corresponding pool of integrated DNA across samples and patients, suggesting that a considerable part of the integrated HIV-1 reservoir was either not activated or was defective (Fig. 2).

## DISCUSSION

Persistent HIV reservoirs are obstacles to the eradication of HIV infection (49). CD4^+^ T cells, particularly resting memory CD4^+^ T-cell reservoirs (4, 5), are one of the major barriers to HIV eradication (7, 8), fueling HIV rebound to pretherapy levels after therapy interruption (50). Conversely, some viral variants detected either in residual viremia during therapy (20–22) or at rebound after therapy interruption (23) are genetically distinct from virus present in T-cell subsets, suggesting the existence of additional reservoirs. Some studies have suggested limited associations between proviral DNA sequences obtained from peripheral CD4^+^ T cells (35, 36), or PBMCs (38), and viral RNA sequences obtained from plasma at the same time point. Given evidence of macrophages as an HIV reservoir during therapy (24, 26, 51, 52) and that a significant portion of SIV reservoirs investigated in the SIV/macaque model are located in different anatomical compartments, including myeloid compartments (24, 53, 54), it is not surprising that concordance of plasma and PBMC-based HIV populations has not been consistently reported (35–39).

To understand the contribution of peripheral blood cells to rebound, we applied deep-sequencing methods to obtain a high resolution of HIV-1 from plasma and PBMC populations based on the V3-loop region in nine patients before and after sequential STI. Compartmentalization and phylogenetic analyses of HIV-1 haplotypes derived from plasma and sequences from the PBMCs in the hypervariable V3 region clearly demonstrated compartmentalization between plasma and PBMC anatomical compartments, with evidence for the presence of diverse HIV-1 virions in plasma that do not have a recent ancestral origin in PBMCs. By looking at the evolution of viral sequences at many STI time points, we found that discordance occurs frequently and that the reservoir responsible for igniting viral rebound population is not always related to those found in PBMCs. Unexpectedly, we made the novel observation that there is subtype discordance between virions from plasma and the proviral reservoir. In our case, subtype C sequences were found as ancestral proviral reservoir in two patients, but not in plasma at rebound, suggesting that the subtype C reservoir was inactive or defective. It is known that upon HIV-1 infection, stringent genetic population bottleneck occurs, and that usually infection is driven by a single founder strain (55). A population bottleneck limits genetic diverse quasi-species when transmitting from a donor to the recipient host. However, the risk groups of several patients with subtype discordance were men who have sex with men (MSM) and injection drug users (IDU). Infections in MSM patients tend to be associated with increased multiplicity of founder viruses (55–59), while multiple subtypes and recombinant forms usually circulate among IDU patients (60–62). These results not only suggested discordance between reservoir and rebound populations, but also that the archival reservoir in these patients may have been the result of multiple infections with different subtypes, which is supported by the great diversity in subtypes and recombinant forms that have circulated in Spain from the 90s (63, 64). Our results perhaps even suggest possible intrahost recombination upon infection with different subtypes. Previous analysis of HIV-1 sequences using deep sequencing showed that intrahost recombination is common (65), especially in patients with AIDS-related malignancies (66). Discordance was also observed when subtypes were matching. The pool of viruses found in blood at rebound in the four subjects was not always intermixing with the provirus found at the same time point or at a time point temporally close to it. The degree of compartmentalization varied between patients as did the intergroup diversity. This could be due to factors such as how long the subject was infected or complexity of infection, as the subject might have been infected by multiple clones, possibly at different times, in comparison to patients which had only a recent clonal expansion.

We acknowledge the limitations of this study. By using next-generation sequencing (NGS) technology for the sequencing of both RNA and DNA, we could not account for clonal expansion. Nonetheless, we could determine more abundant haplotype sequences for the plasma population, based on the number of reads from templates obtained with the Primer ID method. Studies of full-length integrated HIV show that a high proportion of integrated HIV is defective due to internal deletions (15). The proportion of eliminated sequences here is comparatively low, indicating that many of the HIV reads in this study may be from defective genomes. This is another limitation of this study that reduces the power to detect matches between RNA and DNA seeding candidates. Because we tried to minimize PCR recombination based on bulk DNA PCR sequencing, we only studied one read of about 250 bp (lined to the primer). We recognize that the short portion of *env* and the presence of minimal PCR-mediated recombination in the proviral sequences are a limitation of this study. However, our analysis was based on the portion of envelope that contains the V3-loop region, which is critical for coreceptor binding (67). Viruses might share a subgenomic sequence but differ elsewhere in the genome (68, 69), and use of the V3 loop genomic region to associate rebound virus in plasma to provirus in PBMCs may not be sufficient. Although the V3 region is short, it is highly variable and a major determinant of coreceptor usage (70–73). Mutations, even single amino acid mutations in this region, have been correlated with tropism (70, 74). Therefore, it is possible that virus found in plasma at rebound would share the same, or highly similar, V3 region with its ancestral provirus population. Our results did not lead to this conclusion for the majority of the viruses and proviruses, either those sampled at a time point with matching plasma virus and PBMCs or at time points temporally close, suggesting that peak plasma viremia might not correspond to the peak of HIV-1 expression from the PBMC reservoir. Our study demonstrates that sources of post-ATI viremia are diverse and dynamic, even varying across sequential ATIs within individual subjects. Greater insight into the reservoirs that fuel viral rebound will help guide strategies that promote their elimination.

## MATERIALS AND METHODS

### Study subjects, human plasma, and PBMC samples.

Twenty-nine plasma samples and 17 PBMC samples representing multiple collection time points were obtained from nine individuals infected with HIV-1 subtype B. These nine individuals were gathered from studies previously carried out at IrSiCaixa Barcelona (40). During recruitment, subjects chosen had been on stable cART regimen for two or more years before undergoing GTI for up to 4 months before treatment was reinstated, either based on plasma viral load or CD4 nadir. Patient characteristics (sex, birthplace, ethnicity, transmission, etc.) are summarized in Table 1. Multiple plasma and PBMC samples were chosen from each of the nine patients from each of the subsequent STIs to allow for in-depth analysis of reservoir populations, as shown in Fig. 1.

### DNA and RNA extraction and cDNA synthesis.

Viral RNA was extracted from plasma samples using the QIAamp viral RNA minikit, and genomic DNA was extracted from PBMC samples using the ALL Prep DNA/RNA minikit (Qiagen, Germantown, MD) according to the manufacturer’s instructions. Viral RNA was extracted from 140 μL of plasma sample. The cDNA primer was comprised of a nonsense sequence at the 5′ end followed by a 9-nt randomized sequence, a 3-nt spacer sequence, and an HIV-1 gene-specific primer sequence at the 3′ end. The sequence of the Primer ID (75) cDNA primer (PID1_ V1V3HIV1_cDNA_R) was 5′-GTGTCACACGTCTATCGACTACGCCAGCTGTAGTCGATCTNNNNNNNNNCAGCCATTTTGCTCTACTAATGTTACAATGTGC-3′ (HBX2 numbering for the gene-specific region: 7238 to 7209; specific to the envelope polyprotein of HIV and the 3′ end of the V3 region). All primers were synthesized by Integrated DNA Technologies (Coralville, IA) as TruGrade DNA oligonucleotides with hand mixing of random nucleotides and purification by standard desalting. The SuperScript III first-strand synthesis system (Life Technologies, Carlsbad, CA) was used for cDNA synthesis of all plasma samples. Viral RNA was reverse transcribed using the Primer ID cDNA primer. A modified cDNA synthesis protocol was followed to maximize the length of the cDNA. Briefly, the RNA was incubated at 65°C for 5 min in the presence of the primer PID1_ V1V3HIV1_cDNA_R and then cooled quickly to 4°C. First-strand cDNA synthesis was performed in a 40-μL reaction volume containing reverse transcription buffer (10 mM Tris-HCl [pH 8.4], 25 mM KCl), 5 mM MgCl_2_, 10 mM dithiothreitol, 80 units of RNase-OUT (RNase inhibitor), and 40 units of SuperScript III reverse transcriptase (RT). The reaction mixture was heated to 50°C for 180 min, adding an additional 40 units of SuperScript III RT midway through the synthesis before polymerase inactivation at 85°C and the addition of 4 units of Escherichia coli RNase H for 20 min at 37°C. All cDNA was purified using Agencourt AMPure XP beads (Beckman Coulter, Brea, CA) to remove unused cDNA primer. The ratio of the volume of beads to cDNA reaction volume was 0.5. The beads were washed four times with 70% ethanol before the cDNA was eluted off the beads in nuclease-free water. RNA integrity numbers (RIN) were not quantifiable.

### ddPCR.

Total RNA and genomic DNA from PBMC samples were evaluated by digital droplet PCR in triplicate using a custom assay designed to the V3 region of HIV-1 (NC_001802) (Applied Biosystems, Waltham, MA). Briefly, first-strand cDNA synthesis was performed on the RNA samples as previously described. Following quantification, genomic DNA was diluted to a concentration of 3.45 ng/μL. This was not the case for the cDNA samples, because their concentration fell well below a final concentration of 3.45 ng/μL. For each ddPCR mixture, approximately 6 ng of genomic DNA was combined with QuantStudio 3D Master Mix and a custom primer/probe HIV-1 assay (final concentration 250 nM) designed to the V3 region of the envelope region of HIV-1 gp120. Primer and probe sequences located in the gp120 envelope region (NC_001802) were as follows: HIV-1 env forward primer, 5′-AATTGTACAAGACCCAACAACA-3′; HIV-1 env probe, 5′-(6-carboxyfluorescein)-GAGGACCAGGGAGAGCATT-(nonfluorescent quencher)-3′; HIV-1 env reverse primer, 5′-GTTACAATGTGCTTGTCTCATA-3′. Each sample was loaded onto the QuantStudio 3D digital PCR 20K Chip v2 and assembled. PCR amplification was performed using the ProFlex 2xFlat PCR system using the following cycling conditions: 1 cycle at 96°C for 10 min with 1.6°C/s ramping; 39 cycles of 60°C for 2 min and 98°C for 30 s with 1.6°C/s ramping; and 1 cycle at 60°C for 2 min with 1.6°C/s ramping, followed by a final hold at 10°C. Each chip was analyzed using the QuantStudio 3D Digital PCR instrument and then analyzed further using the AnalysisSuite software (Applied Biosystems, version 1.5.2), where replicate wells were merged prior to analysis. Three replicates were performed for each participant sample. The median of qualified (QT) cells was 17,557 (16,506 to 18,385), which comprised of 89 to 97% of the total cells assayed.

### PCR amplification for library preparation.

All cDNA was used for nested amplification after purification with Phusion DNA polymerase (New England BioLabs, Ipswich, MA) according to the manufacturer’s instructions. The first Illumina run was based on a region of approximately 800 bp of *env* containing the V3 region. The first-round PCR forward primer (PID3_1st_V1V3_F) was 5′-GCCTCCCTCGCGCCATCAGAGATGTGTATAAGAGACAGNNNNTTATGGGATCAAAGCCTAAAGCCATGTGTA-3′, in which the 5′ end of the primer included an Illumina transposon sequence, to incorporate the addition of adaptors for Illumina sequencing and a 4-nt-long index region, for multiplexing different samples in the same run, as well as sequence specific to the envelope polyprotein of HIV just upstream from the V1 region. The first-round PCR reverse primer (PID21_V1V3OR_cDNA_R) was 5′-GTGTCACACGTCTATCGACTACGCCAGCTG-3′. First-round PCR products were purified using Agencourt AMPure XP beads (Beckman Coulter, Indianapolis, IN). The ratio of volume of beads to PCR product volume was 0.5. The beads were washed three times with 70% ethanol before eluting the PCR mixtur off the beads in 50 μL of nuclease-free water. Two microliters of the first-strand reaction was used in a second-round PCR with the following primers: PID22_V1V3HIV1_IF, 5′-GCCTCCCTCGCGCCATCAGAGATGTGTATAAGAGACAGNNNNCCACTCTGTGTTAGTTTAAAGTGCA-3′; PID1_V1V3HIV1_cDNA_R, 5′-GTGTCACACGTCTATCGACTACGCCAGCTGTAGTCGATCTNNNNNNNNNCAGCCATTTTGCTCTACTAATGTTACAATGTGC-3′. The second Illumina run was based on an amplicon containing a smaller amplicon of the V3 region of *env* (344 bp). Second-strand synthesis was performed using a nested PCR approach that further targeted the V3 region of HIV-1 (NC_001802). Following bead purification of the first-strand synthesis product using Agencourt AMPure XP beads, second-strand synthesis was performed using Phusion DNA polymerase (New England BioLabs, Ipswich, MA) and the following primer set according to the manufacturer’s instructions: PID16_V3OF_6515F, 5′-TGTCAGCACAGTACAATGTACAC -3′; PID17_V3OR_6892R, 5′-GTGCGTTACAATTTCTGGGTCC-3′. The expected size of this region of *env* was 400 bp. Two microliters of the first-strand reaction mixture was used in a subsequent PCR that yielded a 34- bp product using the following primers: PID18_V3IF_6547F, 5′-GCCAGTAGTATCAACTCAACTGC -3′; PID19_V3IR_6869R, 5′-CTCCTGAGGATTGCTTAAAGAT-3′.

### Illumina library preparation and sequencing.

Library preparation was performed using a KAPA HyperPrep kit (Roche, Pleasanton, CA) with NEXTflex dual-indexed DNA barcodes (PerkinElmer, Waltham, MA) for multiplexing 46 samples in the same sequencing run. The size and purity of the library were determined using the 4150 TapeStation system (Agilent, Santa Clara, CA). The size of the library covering the entire V1-V3 region of *env* and primers, designed based on the HXB2 HIV-1 genome (6,594 to 7,238 bp) was determined to be around 800 bp. Constructed libraries were pooled and sequenced using MiSeq (Illumina, San Diego, CA) with 2 × 250-base paired-end sequencing. For the second Illumina run based on the amplification of the three distinct regions of HIV-1, size and purity of the pooled library were determined using a 4200 TapeStation system (Agilent) and the Qubit dsDNA HS assay kit (Life Technologies, Carlsbad, CA) according to the manufacturer’s instructions. Constructed libraries were pooled and sequenced using the NovaSeq 6000 sequencing system and the NovaSeq 6000 S1 reagent kit v1.5 200 cycle kit.

### Bioinformatic pipeline.

We employed the Primer ID approach, i.e., using unique molecular identifiers (UMIs), to eliminate reads representing PCR-mediated recombination, reduce the sequencing and PCR error to 0.01%, and reveal the true sampling depth (75–77). One limitation of the Primer ID is that this approach only works for RNA as a template. As for proviral DNA sequences, *in vitro* recombination is about 10% of reads generated from nested PCR (78). However, it also has been shown that at a site up to 200 bp from the primer there is little or no recombination (78). For this reason, to minimize PCR-mediated recombination, we analyzed only the read from the primer (about 250 bp).

To determine single template from RNA, we used UMIs (75). Haplotypes from plasma viruses were identified using a custom Python script (primerid.py) that examined each read pair, looking for UMIs in either the R1 or R2 read. Constraints included the following: reads shorter than 100 bp were discarded; if the UMI was found in one of the two mates, the other one should start with GCCTCCCTCGCGCCATCAGAGATGTG; the sequence after the UMI should be at least 150 bp. If the UMI was found, the associated sequence was stored. UMIs that differed only for one base were considered equivalent and their sequences were stored together. The result is a set of clusters, each one identified by a reference UMI (the one that occurs more often). The sequences contained in each cluster having more than five members were saved to a separate FASTA file. Each FASTA file was aligned with MAFFT (79) with the “reorder” option. A custom Python script (RNAconsensus.py) generated the consensus sequence for each alignment for the RNA samples, using a majority rule. Gaps were removed. The consensus sequences for all clusters were saved together in a single file.

We did not use the Primer ID method within the proviral DNA sequences to correct PCR errors, as correcting by number of sequencing reads with same Primer ID would be irrelevant, since they are generated from PCR siblings and only reflect the efficiency and bias of the PCR. For the analysis of the proviral DNA sequences, a CD-HIT pipeline based on a clustering approach was created to obtain sequences from proviral HIV-1 DNA integrated in PBMCs. We clustered reads that did not map to the human grch38 genome (NCBI BioProject PRJNA31257) using CD-HIT (80) by allowing generation of clusters of similar sequences at 99.8% and with at least five reads, regardless of UMIs. The sequences in each cluster were written to a separate FASTA file that was aligned with MAFFT (79) using the “adjustdirection” option. A custom Python script (DNAconsensus.py) generated the consensus sequence for each alignment, using a majority rule. Gaps were removed. The consensus sequences for all clusters were saved in a single file. Only R1 reads were utilized, as R2 reads have lower quality and a higher error rate (81). HIV sequences were identified using BLASTn (82) against the Los Alamo HIV database (www.hiv.lanl.gov), considering only sequences longer than 180 bp and that did not have stop codons. We defined as BLASTn “best hit” the hit with the smallest E value (with a range between 1E−40 and 1E−100), with over 90% of identical matches, and for which the start and end of the alignment were within 400 bp region of the HIV genome that includes the V3 loop.

The second Illumina run was performed on plasma and PBMC samples to obtain more in-depth coverage of the V3 region of patients VBP2, PGG3, SDS4, and JOR10 with NovaSeq. Data were analyzed as explained above using R1 and R2 reads that did not map to the human grch38 genome (NCBI BioProject PRJNA31257), and with R1 and R2 stitched reads (as paired end reads are overlapping). Read pairs were stitched by sliding them across each other from their 3′ ends and finding the relative position that provided the highest identity in the overlapping region, under the condition that the overlap should be at least 9 bases long and show at least 85% identity. Scripts are available upon request.

### Bioinformatic detection of recombination.

Identification of putative recombinant sequences and associated breakpoints were performed using the RDP, GENECONV, BootScan, MaxChi, CHIMAERA, SIScan, and 3Seq algorithms implemented in the RDP4 software using default settings with linear genome specification (83). Statistical evidence of recombination was indicated by *P* values of <0.05, and recombination events were considered as such if supported by at least six of the seven algorithms used (83, 84). No evidence of recombination was found.

### Phylogenetic and phylodynamic analyses.

COMET, a tool that excels in detecting and identifying new recombinant forms, was used to detect and confirm the subtypes (85). We explored hypermutation in the GG or GA context using the Hypermut algorithm (86). Intrapopulation for the in-between patients genetic p-distance was calculated using MegaX v.10.0.3 (87). Sequences from the two runs were aligned using MAFFT (79). Before performing the maximum likelihood (ML) phylogenetic and Bayesian phylodynamic analyses, we randomly subset the VBP2, PCG3, and SDS4 data sets targeting sequences by compartments (plasma and PBMC when needed) and by time point using TARDIS (43). These data sets contained a large number of sequences, many of which were nearly identical and therefore not contributing to the phylogenetic resolution. ML phylogenies based on *env* sequences were reconstructed using IQ-TREE (88), and the best-fitting evolutionary model was chosen according to Bayesian information criteria (89) and ultrafast bootstrap (BB) approximation (90). Presence of sufficient phylogenetic signal was evaluated by performing likelihood mapping analysis in IQ-TREE (88, 91, 92). Our Bayesian phyloanatomy approach (24) allowed us to trace movement and ancestry of the virus between the compartments. A maximum clade credibility (MCC) tree was obtained from a posterior distribution of phylogenies inferred with the Bayesian framework implemented in BEAST v1.10.4 (93), as previously done for HIV-1 (26). Briefly, an HKY nucleotide substitution model, uncorrelated molecular relaxed clock (94) assuming a mean HIV-1 intrahost evolutionary rate of 7.53 × 10^−3 ^nt substitutions/site/year (26, 44), consistent with those of intrahost virus estimations of HIV-1-infected individuals under therapy (95), and a Bayesian skyline plot demographic priors were enforced. The relaxed clock allows each branch of a phylogenetic tree to have its own evolutionary rate (94). This clock assumes that the evolutionary rate at one branch does not depend upon the rate at any of the neighboring branches. This means that the evolutionary rate across branches can change abruptly, i.e., going from fast to slow or slow to fast suddenly, rather than needing to steadily increase or decrease over multiple adjoining branches. For each data set, a Markov chain Monte Carlo analysis was run for 1 billion generations, sampling every 100,000 generations. Proper mixing of the Markov chain was assessed by calculating the effective sampling size of each parameter estimate, which resulted in >200 for all parameters at the end of the run, after 10% burn-in. The MCC trees were calculated from the posterior distribution with TreeAnnotator for each patient, and graphically edited in R using the ggtree package (96). The xml files are available upon request.

### Compartmentalization analysis.

We performed Wright’s measure of population subdivision (FST) test (97) to determine the degree of compartmentalization. FST is a distance-based method that compares the mean pairwise genetic distance between two sequences sampled at random from different compartments to the mean distance between sequences sampled at random from the same compartment. For the analysis, sequences were randomly subset to obtain balanced population sizes, as results would be biased by uneven sample sizes (98). Compartmentalization analyses were performed with Hyphy v.2.5.1 (99), and we calculated four distance-based indexes (97, 100–102) and two tree-based indexes (103, 104).

### Data availability.

Sequences used for phylogenetic analysis in this paper have been deposited in the GenBank database, under the following accession numbers: OM373655 to OM386647.
