# Supplementary material for: Discordance between HIV-1 Population in Plasma at Rebound after Structured Treatment Interruption and Archived Provirus Population in Peripheral Blood Mononuclear Cells
Source: Microbiol Spectr. 2022 Jun 14;10(4):e01353-22. doi: 10.1128/spectrum.01353-22 (PMC9431602; doi:10.1128/spectrum.01353-22)
Supplement: Supplemental file 1 — Supplemental material. Download spectrum.01353-22-s0001.pdf, PDF file, 6.5 MB [file spectrum.01353-22-s0001.pdf]

Supplementary Figures and Tables

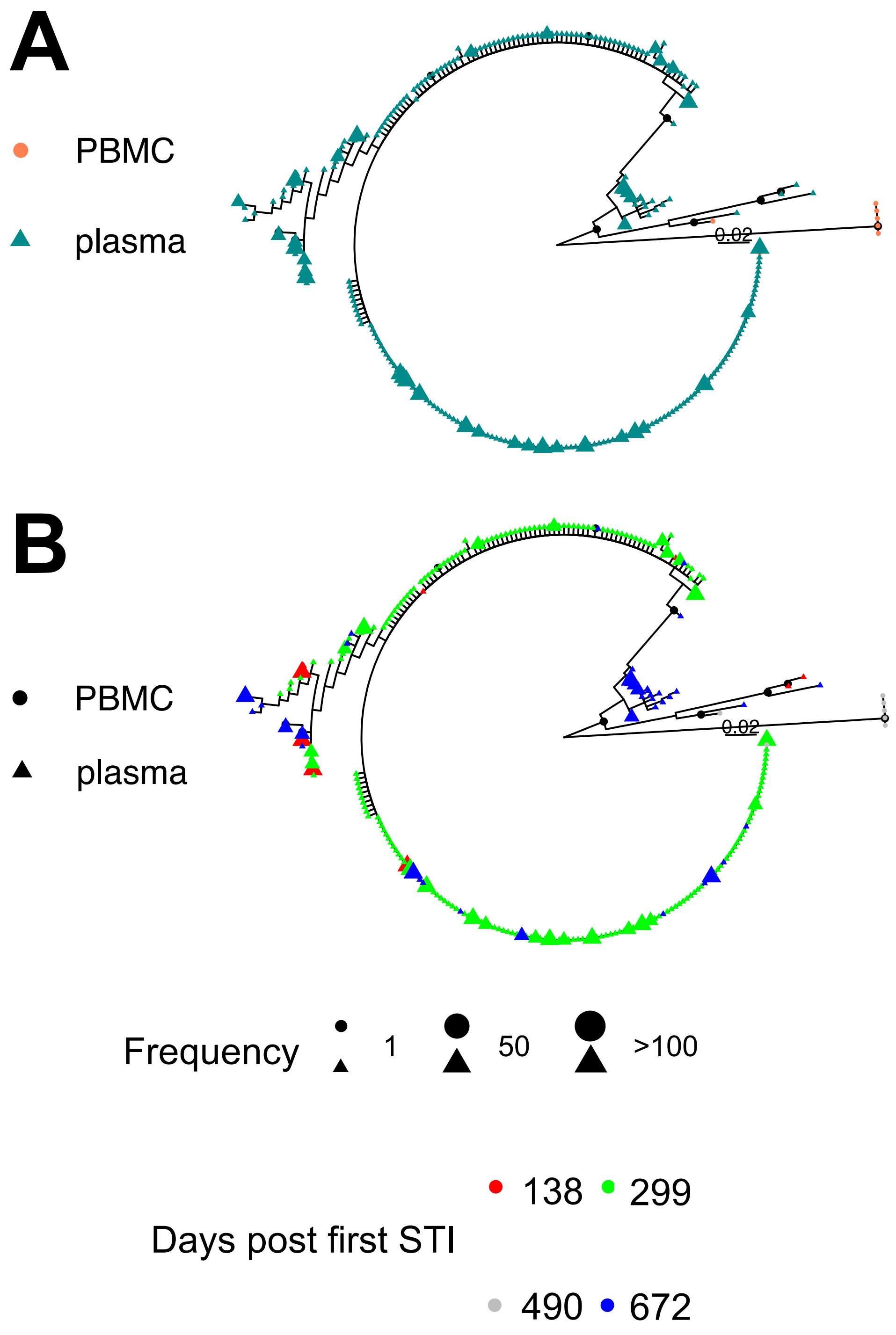

Figure S1. Maximum likelihood phylogeny of patient VBP2. Maximum likelihood tree with tips colored by (A) anatomical origin of the sequence, either PBMCs or plasma, or (B) by timepoint in days post first STI as shown in figure legend. Statistical support for clades is shown at nodes based on ultra-fast bootstrap values (> 90) by black circles.

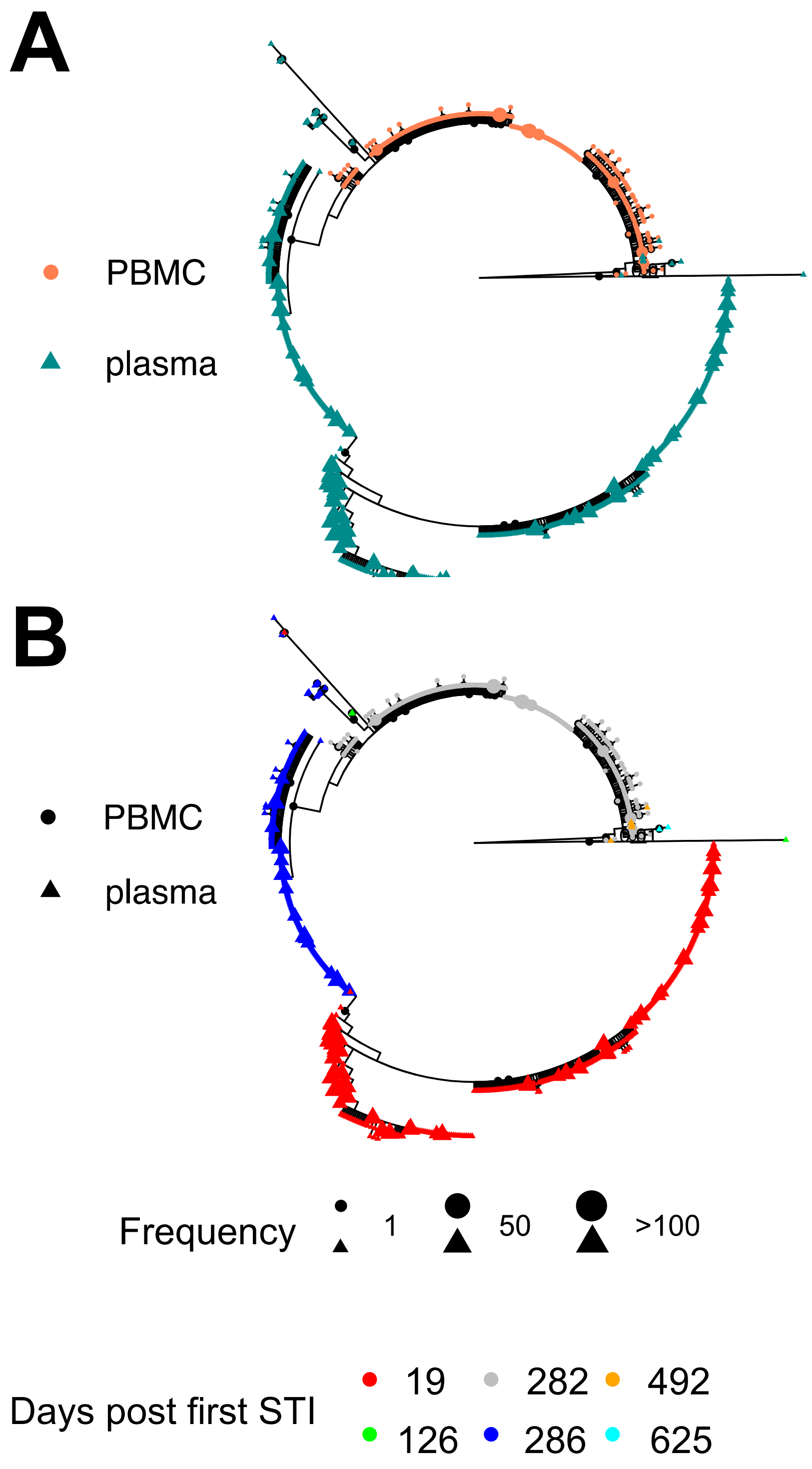

Figure S2. Maximum likelihood phylogeny of patient PCG3. Maximum likelihood tree with tips colored by (A) anatomical origin of the sequence, either PBMCs or plasma, or (B) by timepoint in days post first STI as shown in figure legend. Statistical support for clades is shown at nodes based on ultra-fast bootstrap values (> 90) by black circles.

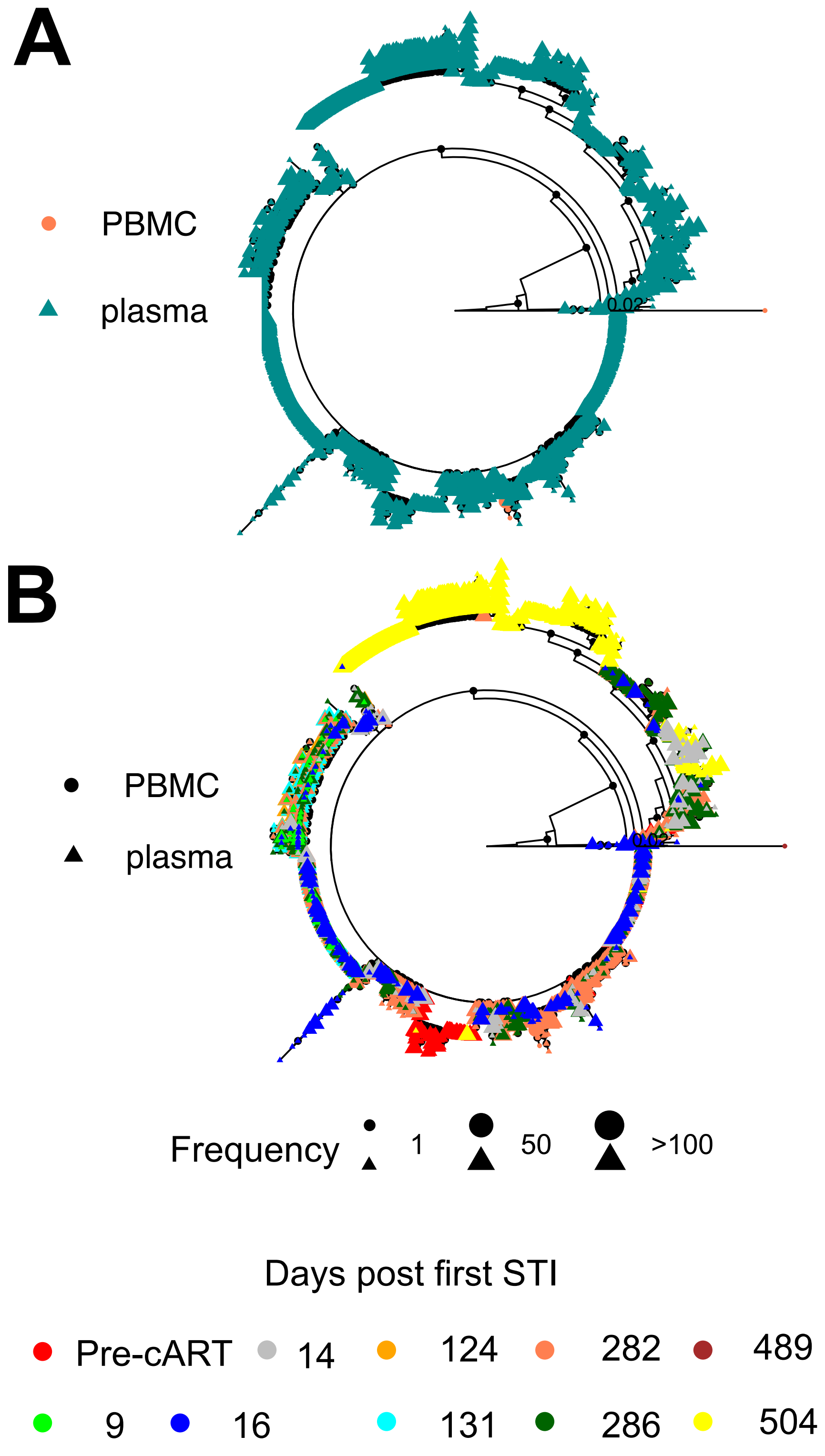

Figure S3. Maximum likelihood phylogeny of patient SDS4. Maximum likelihood tree with tips colored by (A) anatomical origin of the sequence, either PBMCs or plasma, or (B) by timepoint in

days post first STI as shown in figure legend. Statistical support for clades is shown at nodes based on ultra-fast bootstrap values (> 90) by black circles.

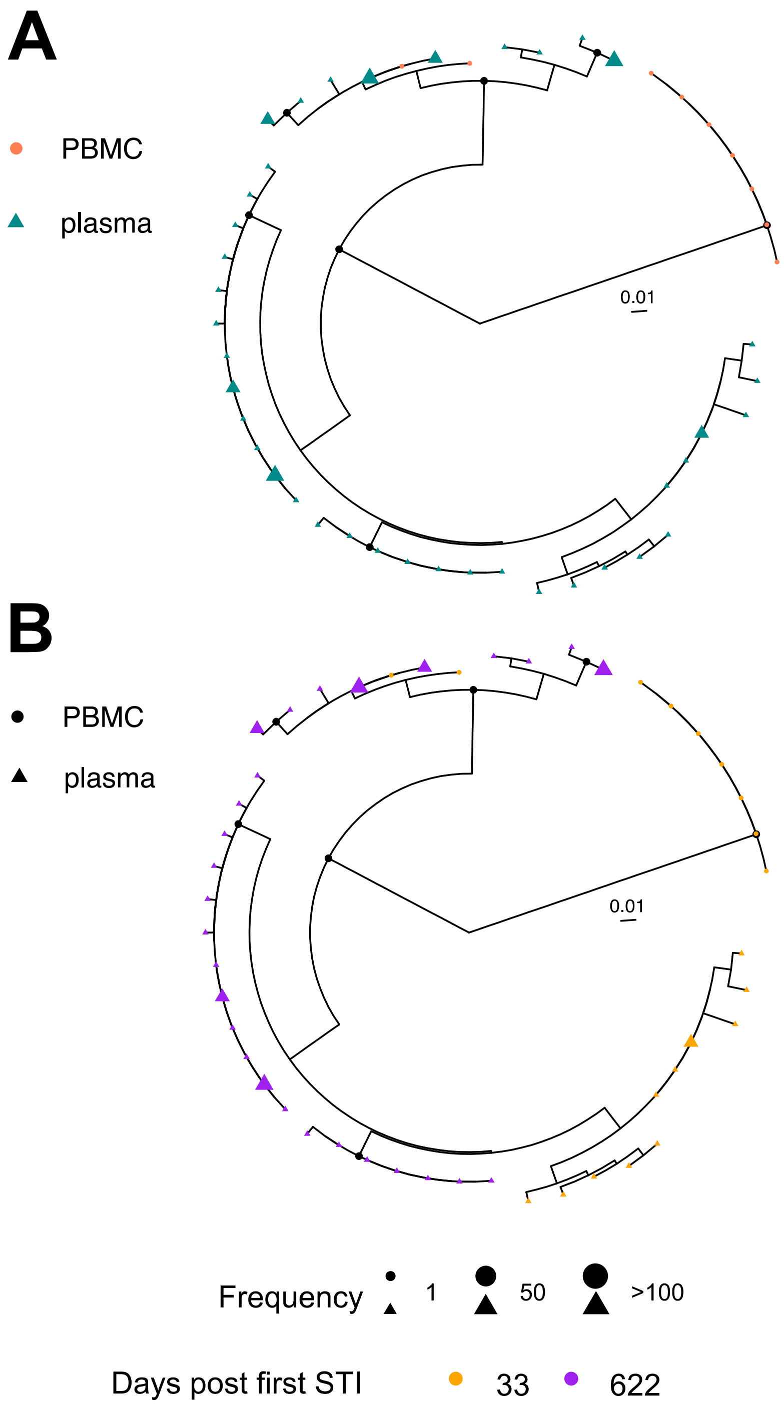

Figure S4. Maximum likelihood phylogeny of patient JOR10. Maximum likelihood tree with tips colored by (A) anatomical origin of the sequence, either PBMCs or plasma, or (B) by timepoint in days post first STI as shown in figure legend. Statistical support for clades is shown at nodes based on ultra-fast bootstrap values (> 90) by black circles.

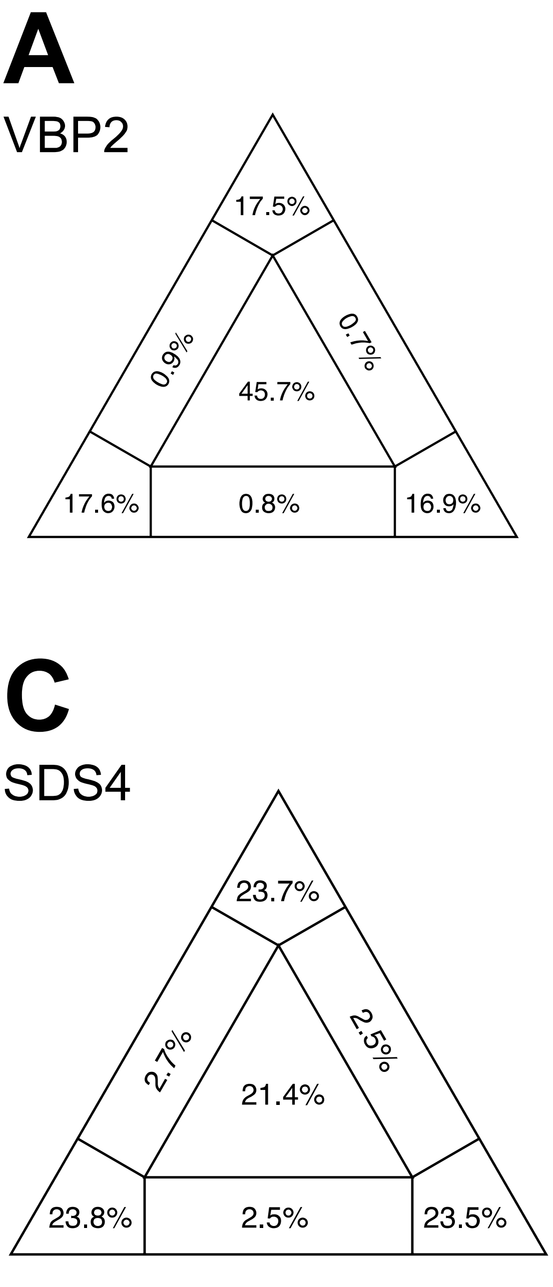

Figure S5. Phylogenetic signal in reduced datasets of VBP2, PCG3 and SDS4, and full dataset of JOR10. Likelihood mapping assesses the amount of phylogenetic noise in alignments, and if there is enough signal for phylogenetic inference. Values in the center of the triangle indicate the amount of completely unresolved quartets, while the sum of the values in the corners indicates the percentage of fully resolved quartets. Most alignments show strong signal, with very low percentage of unresolved quartets (percentage in the center of the triangle).

Patient	Timepoint	day from first STI	Sample	Viral Load	PCR 1st run
VBP2	11	23	Plasma	9507	-
VBP2	30	138	Plasma	51100	+
VBP2	53	299	Plasma	50600	+
VBP2	72	490	Plasma	952	-
VBP2	72	490	PBMC		+
VBP2	73	492	Plasma	4840	-
VBP2	91	672	Plasma	63000	+
PCG3	9	19	Plasma	3030	-
PCG3	27	126	Plasma	22500	+
PCG3	50	282	Plasma	1210	-
PCG3	50	282	PBMC		+
PCG3	55	294	Plasma	8000	+
PCG3	75	483	Plasma	1560	-
PCG3	75	483	PBMC		+
PCG3	79	492	Plasma	10300	+
PCG3	91	621	Plasma	59500	-
PCG3	92	625	PBMC		+
PCG3	92	625	Plasma	38500	+
SDS4	pre-CART	pre-CART	Plasma		+
SDS4	5	9	PBMC		+
SDS4	5	9	Plasma	2290	-
SDS4	7	14	Plasma	23441	+
SDS4	8	16	Plasma	25010	+
SDS4	24	124	Plasma	2440	+
SDS4	27	131	Plasma	12400	+
SDS4	44	279	Plasma	4780	-
SDS4	45	282	PBMC		+
SDS4	45	282	Plasma	26800	+
SDS4	46	286	Plasma	70000	+
SDS4	63	489	PBMC		+
SDS4	63	489	Plasma	1470	+
SDS4	67	504	Plasma	26700	+
CGD5	11	11	Plasma	3600	-
CGD5	25	25	Plasma	2450	-
CGD5	49	49	Plasma	1970	-
CGD5	70	70	Plasma	1060	-
CGD5	70	70	PBMC		+
CGD5	71	71	Plasma	2410	-

CGD5	**90**	**90**	Plasma	17600	+
CGD5	**91**	**91**	PBMC		+
CGD5	**91**	**91**	Plasma	13600	+
EHD6	**14**	**14**	Plasma	3840	-
EHD6	**29**	**29**	Plasma	1040	-
EHD6	**89**	**89**	Plasma	956	-
EHD6	**89**	**89**	PBMC		+
EHD6	**90**	**90**	Plasma	35500	+
EHD6	**pre-CART**	**pre-CART**	Plasma		+
SMR7	**15**	**15**	Plasma	2110	-
SMR7	**34**	**34**	Plasma	3950	-
SMR7	**57**	**57**	Plasma	1410	-
SMR7	**58**	**58**	Plasma	50	-
SMR7	**58**	**58**	PBMC		+
SMR7	**78**	**78**	Plasma	1210	-
SMR7	**94**	**94**	Plasma	974	-
SMR7	**94**	**94**	PBMC		+
SMR7	**99**	**99**	Plasma	710	+
MMG8	**8**	**8**	Plasma	11028	+
MMG8	**10**	**10**	PBMC		+
MMG8	**10**	**10**	Plasma	246000	+
MMG8	**23**	**23**	Plasma	59900	+
MMG8	**47**	**47**	Plasma	38300	-
MMG8	**71**	**71**	Plasma	15500	-
MMG8	**71**	**71**	PBMC		+
MMG8	**72**	**72**	Plasma	11500	-
JOR10	**14**	**33**	PBMC		+
JOR10	**14**	**33**	Plasma	1170	+
JOR10	**26**	**124**	Plasma	4780	-
JOR10	**54**	**299**	Plasma	2210	+
JOR10	**80**	**511**	Plasma	3840	-
JOR10	**94**	**622**	PBMC		-
JOR10	**94**	**622**	Plasma	3930	-
JOR10	**97**	**642**	Plasma	86500	+
NTU12	**31**	**31**	Plasma	7540	+
NTU12	**59**	**59**	Plasma	10600	-
NTU12	**77**	**77**	Plasma	6730	-
NTU12	**87**	**87**	PBMC		+
NTU12	**87**	**87**	Plasma	838	-
NTU12	**95**	**95**	Plasma	260000	+

NS= not sequenced

HIV match > 150 bp (1st run)	PCR 2nd run	HIV match > 150 bp (2nd run)
-	-	-
+	+	
+	+	
-	-	-
+	+	
-	-	-
+	+	
-	-	-
+	+	
-	-	-
+	+	
+	+	
-	-	-
-	+	
+	+	
-	-	-
-	+	
+	+	
+	+	
+	+	
-	+	
+	+	
-	+	
-	+	
-	+	
-	-	-
+	+	
+	+	
+	+	
+	+	
-	+	
+	+	
-	NS	NS
-	NS	NS
-	NS	NS
-	NS	NS
+	NS	NS
-	NS	NS

-	NS	NS
+	NS	NS
-	NS	NS
-	NS	NS
-	NS	NS
-	NS	NS
+	NS	NS
+	NS	NS
+	NS	NS
-	NS	NS
-	NS	NS
-	NS	NS
-	NS	NS
-	NS	NS
-	NS	NS
-	NS	NS
+	NS	NS
-	NS	NS
+	NS	NS
+	NS	NS
+	NS	NS
+	NS	NS
-	NS	NS
-	NS	NS
-	NS	NS
-	NS	NS
+	+	
+	+	
-	-	-
	+	
-	-	-
-	+	
-	-	-
+	+	
-	NS	NS
-	NS	NS
-	NS	NS
+	NS	NS
-	NS	NS
+	NS	NS

Table S1. Breakdown by patient of the PCR amplification success and viral load per timepoint.

Table S2. Raw reads, percent of stitched reads, percent of HIV reads, and number of HIV plasma haplotypes and PBMCs sequences for the first Illumina run**.**

Pt	Sample	Timepoint (d)	Reads	HIV reads	% HIV reads	HIV plasma haplotypes and PBMCs sequences
VBP2	PBMCs	490	225,001	46	0.02	6
VBP2	plasma	138	243,481	21	0.009	4
VBP2	plasma	299	176,224	22	0.01	4
VBP2	plasma	672	222,727	102	0.04	18
PCG3	PBMCs	282	127,318	33,279	26.13	370
PCG3	plasma	126	223,555	19	0.008	3
PCG3	plasma	294	196,390	88	0.04	16
PCG3	plasma	492	189,288	45	0.02	8
PCG3	plasma	652	243,206	16	0.007	3
SDS4	PBMCs	9	196,389	88	0.04	2
SDS4	PBMCs	282	282,806	1,921	0.67	11
SDS4	PBMCs	489	259,302	17	0.00	1
SDS4	plasma	pre-cART	215,800	32	0.01	6
SDS4	plasma	9	163,809	12	0.007	2
SDS4	plasma	124	329,539	5	0.002	1
SDS4	plasma	131	190,186	147	0.07	24
SDS4	plasma	504	276,643	10	0.004	3
JOR10	PBMCs	33	286,210	94	0.03	7
JOR10	plasma	33	259,216	67	0.02	11
JOR10	plasma	622	223,924	46	0.02	7

Pt: patient; d: days post first STI.

Table S3. Raw reads, percent of stitched reads, percent of HIV reads, and number of HIV plasma haplotypes and PBMCs sequences for the second Illumina run**.**

Pt	Sample	Timepoint (d)	Reads	Stitched reads	% Stitched reads	HIV reads	% HIV reads	HIV plasma haplotypes and PBMCs sequences
VBP2	plasma	138	24,826	1,457	5.90%	1010	69.3	11
VBP2	plasma	299	103,981	54,132	52.10%	48,172	88.98	210
VBP2	plasma	672	30,797	12,118	39.30%	9,155	75.54	47
PCG3	PBMCs	282	1,946,864	1,838	0.10%	20	1.08	1
PCG3	PBMCs	625	939,356	35	0.00%	35	100	1
PCG3	plasma	19	190,114	119,477	62.80%	104,813	87.72	338
PCG3	plasma	294	121,750	59,371	48.80%	53,859	90.71	238
SDS4	PBMCs	9	1,628,995	1,506	0.10%	35	2.32	1
SDS4	PBMCs	489	1,221,456	705	0.10%	31	4.39	1
SDS4	plasma	pre-cART	816,210	632,970	77.50%	183,496	28.98	1,440
SDS4	plasma	9	101,064	54,609	54.00%	49,617	90.85	191
SDS4	plasma	14	208,306	121,266	58.20%	102,338	84.39	422
SDS4	plasma	16	159,992	97,653	61.00%	77,253	79.10	467
SDS4	plasma	124	578,179	384,094	66.40%	43,350	11.28	723
SDS4	plasma	131	504,635	377,211	74.70%	40,993	10.86	730
SDS4	plasma	282	1,990,178	1,535,008	77.10%	650,537	42.38	3,811
SDS4	plasma	286	546,463	397,475	72.70%	250,048	62.90	1,337
SDS4	plasma	504	3,242,061	2,581,879	79.60%	597,777	23.15	2,838
JOR10	PBMCs	33	2,135,818	950	0.00%	32	3.36	2
JOR10	plasma	622	41,182	11,046	26.80%	9,114	82.50	21

Pt: patient; d: days post first STI.

Patient VBP2

haplotype	number of reads	timepoint
VBP-V3-plasma-53_Cluster_1_sequences=42002	42002	299
VBP-V3-plasma-91_Cluster_0_sequences=5025	5025	672
VBP-V3-plasma-91_Cluster_1287_sequences=2582	2582	672
VBP-V3-plasma-53_Cluster_87_sequences=515	515	299
VBP-V3-plasma-53_Cluster_42_sequences=463	463	299
VBP-V3-plasma-30_Cluster_3_sequences=290	290	138
VBP-V3-plasma-91_Cluster_5_sequences=256	256	672
VBP-V3-plasma-30_Cluster_5_sequences=225	225	138
VBP-V3-plasma-30_Cluster_2_sequences=187	187	138
VBP-V3-plasma-53_Cluster_39_sequences=174	174	299
VBP-V3-plasma-30_Cluster_8_sequences=165	165	138
VBP-V3-plasma-53_Cluster_36_sequences=157	157	299
VBP-V3-plasma-53_Cluster_115_sequences=127	127	299
VBP-V3-plasma-53_Cluster_96_sequences=115	115	299
VBP-V3-plasma-91_Cluster_56_sequences=111	111	672
VBP-V3-plasma-53_Cluster_27_sequences=106	106	299
VBP-V3-plasma-53_Cluster_89_sequences=101	101	299
VBP-V3-plasma-53_Cluster_143_sequences=99	99	299
VBP-V3-plasma-91_Cluster_1300_sequences=98	98	672
VBP-V3-plasma-53_Cluster_45_sequences=97	97	299
VBP-V3-plasma-53_Cluster_86_sequences=92	92	299
VBP-V3-plasma-53_Cluster_59_sequences=89	89	299
VBP-V3-plasma-53_Cluster_213_sequences=81	81	299
VBP-V3-plasma-53_Cluster_163_sequences=79	79	299
VBP-V3-plasma-53_Cluster_263_sequences=75	75	299
VBP-V3-plasma-53_Cluster_54_sequences=68	68	299
VBP-V3-plasma-91_Cluster_1296_sequences=60	60	672
VBP-V3-plasma-53_Cluster_76_sequences=60	60	299
VBP-V3-plasma-91_Cluster_72_sequences=59	59	672
VBP-V3-plasma-53_Cluster_14_sequences=59	59	299
VBP-V3-plasma-53_Cluster_33_sequences=59	59	299
VBP-V3-plasma-53_Cluster_90_sequences=58	58	299
VBP-V3-plasma-91_Cluster_2_sequences=57	57	672
VBP-V3-plasma-91_Cluster_41_sequences=55	55	672
VBP-V3-plasma-91_Cluster_92_sequences=55	55	672
VBP-V3-plasma-53_Cluster_50_sequences=54	54	299
VBP-V3-plasma-91_Cluster_1317_sequences=52	52	672
VBP-V3-plasma-53_Cluster_239_sequences=51	51	299
VBP-V3-plasma-53_Cluster_303_sequences=50	50	299
VBP-V3-plasma-30_Cluster_42_sequences=48	48	138
VBP-V3-plasma-91_Cluster_184_sequences=48	48	672
VBP-V3-plasma-91_Cluster_1295_sequences=48	48	672
VBP-V3-plasma-53_Cluster_740_sequences=48	48	299

VBP-V3-plasma-91_Cluster_1321_sequences=46	46	672
VBP-V3-plasma-53_Cluster_189_sequences=43	43	299
VBP-V3-plasma-53_Cluster_214_sequences=42	42	299
VBP-V3-plasma-53_Cluster_48_sequences=42	42	299
VBP-V3-plasma-53_Cluster_2648_sequences=40	40	299
VBP-V3-plasma-91_Cluster_13_sequences=39	39	672
VBP-V3-plasma-91_Cluster_1311_sequences=39	39	672
VBP-V3-plasma-53_Cluster_52_sequences=39	39	299
VBP-V3-plasma-53_Cluster_106_sequences=38	38	299
VBP-V3-plasma-53_Cluster_257_sequences=38	38	299
VBP-V3-plasma-53_Cluster_159_sequences=35	35	299
VBP-V3-plasma-53_Cluster_358_sequences=35	35	299
VBP-V3-plasma-53_Cluster_44_sequences=34	34	299
VBP-V3-plasma-53_Cluster_174_sequences=34	34	299
VBP-V3-plasma-91_Cluster_1_sequences=33	33	672
VBP-V3-plasma-53_Cluster_181_sequences=33	33	299
VBP-V3-plasma-53_Cluster_388_sequences=31	31	299
VBP-V3-plasma-53_Cluster_295_sequences=30	30	299
VBP-V3-plasma-53_Cluster_57_sequences=30	30	299
VBP-V3-plasma-53_Cluster_78_sequences=30	30	299
VBP-V3-plasma-30_Cluster_6_sequences=29	29	138
VBP-V3-plasma-91_Cluster_38_sequences=29	29	672
VBP-V3-plasma-91_Cluster_1291_sequences=29	29	672
VBP-V3-plasma-53_Cluster_408_sequences=29	29	299
VBP-V3-plasma-53_Cluster_1996_sequences=29	29	299
VBP-V3-plasma-53_Cluster_161_sequences=28	28	299
VBP-V3-plasma-53_Cluster_519_sequences=27	27	299
VBP-V3-plasma-53_Cluster_99_sequences=27	27	299
VBP-V3-plasma-53_Cluster_194_sequences=26	26	299
VBP-V3-plasma-53_Cluster_46_sequences=26	26	299
VBP-V3-plasma-91_Cluster_1447_sequences=25	25	672
VBP-V3-plasma-53_Cluster_148_sequences=25	25	299
VBP-V3-plasma-53_Cluster_270_sequences=25	25	299
VBP-V3-plasma-53_Cluster_317_sequences=25	25	299
VBP-V3-plasma-53_Cluster_494_sequences=25	25	299
VBP-V3-plasma-53_Cluster_721_sequences=25	25	299
VBP-V3-plasma-53_Cluster_13_sequences=25	25	299
VBP-V3-plasma-53_Cluster_18_sequences=25	25	299
VBP-V3-plasma-53_Cluster_196_sequences=25	25	299
VBP-V3-plasma-53_Cluster_72_sequences=25	25	299
VBP-V3-plasma-53_Cluster_357_sequences=24	24	299
VBP-V3-plasma-53_Cluster_938_sequences=24	24	299
VBP-V3-plasma-53_Cluster_658_sequences=24	24	299
VBP-V3-plasma-53_Cluster_65_sequences=24	24	299
VBP-V3-plasma-53_Cluster_229_sequences=24	24	299

VBP-V3-plasma-91_Cluster_145_sequences=22	22	672
VBP-V3-plasma-91_Cluster_122_sequences=22	22	672
VBP-V3-plasma-53_Cluster_149_sequences=22	22	299
VBP-V3-plasma-53_Cluster_95_sequences=22	22	299
VBP-V3-plasma-53_Cluster_421_sequences=22	22	299
VBP-V3-plasma-53_Cluster_232_sequences=22	22	299
VBP-V3-plasma-53_Cluster_6_sequences=22	22	299
VBP-V3-plasma-53_Cluster_964_sequences=22	22	299
VBP-V3-plasma-91_Cluster_329_sequences=21	21	672
VBP-V3-plasma-91_Cluster_1303_sequences=21	21	672
VBP-V3-plasma-53_Cluster_68_sequences=21	21	299
VBP-V3-plasma-53_Cluster_69_sequences=21	21	299
VBP-V3-plasma-53_Cluster_141_sequences=21	21	299
VBP-V3-plasma-53_Cluster_699_sequences=21	21	299
VBP-V3-plasma-53_Cluster_288_sequences=21	21	299
VBP-V3-plasma-53_Cluster_751_sequences=21	21	299
VBP-V3-plasma-91_Cluster_8_sequences=20	20	672
VBP-V3-plasma-91_Cluster_1424_sequences=20	20	672
VBP-V3-plasma-91_Cluster_1290_sequences=20	20	672
VBP-V3-plasma-53_Cluster_55_sequences=20	20	299
VBP-V3-plasma-53_Cluster_633_sequences=20	20	299
VBP-V3-plasma-53_Cluster_302_sequences=20	20	299
VBP-V3-plasma-53_Cluster_383_sequences=20	20	299
VBP-V3-plasma-53_Cluster_125_sequences=20	20	299
VBP-V3-plasma-53_Cluster_195_sequences=20	20	299
VBP-V3-plasma-53_Cluster_268_sequences=20	20	299
VBP-V3-plasma-53_Cluster_536_sequences=20	20	299
VBP-V3-plasma-53_Cluster_472_sequences=20	20	299
VBP-V3-plasma-30_Cluster_296_sequences=19	19	138
VBP-V3-plasma-91_Cluster_62_sequences=19	19	672
VBP-V3-plasma-53_Cluster_184_sequences=19	19	299
VBP-V3-plasma-53_Cluster_443_sequences=19	19	299
VBP-V3-plasma-53_Cluster_616_sequences=19	19	299
VBP-V3-plasma-53_Cluster_730_sequences=19	19	299
VBP-V3-plasma-53_Cluster_158_sequences=19	19	299
VBP-V3-plasma-53_Cluster_207_sequences=18	18	299
VBP-V3-plasma-53_Cluster_667_sequences=18	18	299
VBP-V3-plasma-53_Cluster_142_sequences=18	18	299
VBP-V3-plasma-53_Cluster_1047_sequences=18	18	299
VBP-V3-plasma-53_Cluster_53_sequences=18	18	299
VBP-V3-plasma-53_Cluster_564_sequences=18	18	299
VBP-V3-plasma-53_Cluster_754_sequences=18	18	299
VBP-V3-plasma-91_Cluster_20_sequences=17	17	672
VBP-V3-plasma-53_Cluster_126_sequences=17	17	299
VBP-V3-plasma-53_Cluster_398_sequences=17	17	299

VBP-V3-plasma-53_Cluster_511_sequences=17	17	299
VBP-V3-plasma-53_Cluster_56_sequences=17	17	299
VBP-V3-plasma-53_Cluster_130_sequences=17	17	299
VBP-V3-plasma-53_Cluster_344_sequences=17	17	299
VBP-V3-plasma-53_Cluster_243_sequences=17	17	299
VBP-V3-plasma-53_Cluster_457_sequences=17	17	299
VBP-V3-plasma-53_Cluster_103_sequences=17	17	299
VBP-V3-plasma-53_Cluster_1053_sequences=17	17	299
VBP-V3-plasma-53_Cluster_124_sequences=17	17	299
VBP-V3-plasma-53_Cluster_92_sequences=17	17	299
VBP-V3-plasma-91_Cluster_187_sequences=16	16	672
VBP-V3-plasma-91_Cluster_251_sequences=16	16	672
VBP-V3-plasma-53_Cluster_165_sequences=16	16	299
VBP-V3-plasma-53_Cluster_25_sequences=16	16	299
VBP-V3-plasma-53_Cluster_390_sequences=16	16	299
VBP-V3-plasma-53_Cluster_3_sequences=16	16	299
VBP-V3-plasma-53_Cluster_782_sequences=16	16	299
VBP-V3-plasma-53_Cluster_277_sequences=16	16	299
VBP-V3-plasma-53_Cluster_747_sequences=16	16	299
VBP-V3-plasma-53_Cluster_300_sequences=16	16	299
VBP-V3-plasma-53_Cluster_186_sequences=16	16	299
VBP-V3-plasma-53_Cluster_198_sequences=16	16	299
VBP-V3-plasma-53_Cluster_296_sequences=16	16	299
VBP-V3-plasma-53_Cluster_741_sequences=16	16	299
VBP-V3-plasma-91_Cluster_119_sequences=15	15	672
VBP-V3-plasma-91_Cluster_1325_sequences=15	15	672
VBP-V3-plasma-53_Cluster_1087_sequences=15	15	299
VBP-V3-plasma-53_Cluster_154_sequences=15	15	299
VBP-V3-plasma-53_Cluster_208_sequences=15	15	299
VBP-V3-plasma-53_Cluster_719_sequences=15	15	299
VBP-V3-plasma-53_Cluster_373_sequences=15	15	299
VBP-V3-plasma-53_Cluster_825_sequences=15	15	299
VBP-V3-plasma-53_Cluster_838_sequences=15	15	299
VBP-V3-plasma-53_Cluster_271_sequences=15	15	299
VBP-V3-plasma-53_Cluster_305_sequences=15	15	299
VBP-V3-plasma-53_Cluster_375_sequences=15	15	299
VBP-V3-plasma-91_Cluster_426_sequences=14	14	672
VBP-V3-plasma-91_Cluster_81_sequences=14	14	672
VBP-V3-plasma-91_Cluster_214_sequences=14	14	672
VBP-V3-plasma-91_Cluster_285_sequences=14	14	672
VBP-V3-plasma-53_Cluster_1396_sequences=14	14	299
VBP-V3-plasma-53_Cluster_273_sequences=14	14	299
VBP-V3-plasma-53_Cluster_30_sequences=14	14	299
VBP-V3-plasma-53_Cluster_545_sequences=14	14	299
VBP-V3-plasma-53_Cluster_745_sequences=14	14	299

VBP-V3-plasma-53_Cluster_40_sequences=14	14	299
VBP-V3-plasma-53_Cluster_173_sequences=14	14	299
VBP-V3-plasma-53_Cluster_1121_sequences=14	14	299
VBP-V3-plasma-53_Cluster_1420_sequences=14	14	299
VBP-V3-plasma-53_Cluster_2066_sequences=14	14	299
VBP-V3-plasma-53_Cluster_586_sequences=14	14	299
VBP-V3-plasma-30_Cluster_18_sequences=13	13	138
VBP-V3-plasma-91_Cluster_118_sequences=13	13	672
VBP-V3-plasma-91_Cluster_2025_sequences=13	13	672
VBP-V3-plasma-53_Cluster_275_sequences=13	13	299
VBP-V3-plasma-53_Cluster_304_sequences=13	13	299
VBP-V3-plasma-53_Cluster_307_sequences=13	13	299
VBP-V3-plasma-53_Cluster_468_sequences=13	13	299
VBP-V3-plasma-53_Cluster_961_sequences=13	13	299
VBP-V3-plasma-53_Cluster_469_sequences=13	13	299
VBP-V3-plasma-53_Cluster_423_sequences=13	13	299
VBP-V3-plasma-53_Cluster_621_sequences=13	13	299
VBP-V3-plasma-53_Cluster_597_sequences=13	13	299
VBP-V3-plasma-53_Cluster_41_sequences=13	13	299
VBP-V3-PBMC-72_Cluster_431_sequences=12	12	490
VBP-V3-plasma-30_Cluster_64_sequences=12	12	138
VBP-V3-plasma-91_Cluster_1304_sequences=12	12	672
VBP-V3-plasma-53_Cluster_1020_sequences=12	12	299
VBP-V3-plasma-53_Cluster_1039_sequences=12	12	299
VBP-V3-plasma-53_Cluster_122_sequences=12	12	299
VBP-V3-plasma-53_Cluster_253_sequences=12	12	299
VBP-V3-plasma-53_Cluster_320_sequences=12	12	299
VBP-V3-plasma-53_Cluster_70_sequences=12	12	299
VBP-V3-plasma-53_Cluster_987_sequences=12	12	299
VBP-V3-plasma-53_Cluster_526_sequences=12	12	299
VBP-V3-plasma-53_Cluster_29_sequences=12	12	299
VBP-V3-plasma-53_Cluster_332_sequences=12	12	299
VBP-V3-plasma-53_Cluster_2079_sequences=12	12	299
VBP-V3-plasma-53_Cluster_669_sequences=12	12	299
VBP-V3-plasma-53_Cluster_1022_sequences=12	12	299
VBP-V3-plasma-53_Cluster_1543_sequences=12	12	299
VBP-V3-plasma-53_Cluster_168_sequences=12	12	299
VBP-V3-plasma-53_Cluster_315_sequences=12	12	299
VBP-V3-plasma-53_Cluster_481_sequences=12	12	299
VBP-V3-plasma-53_Cluster_618_sequences=12	12	299
VBP-V3-plasma-53_Cluster_648_sequences=12	12	299
VBP-V3-plasma-53_Cluster_706_sequences=12	12	299
VBP-V3-PBMC-72_Cluster_13_sequences=11	11	490
VBP-V3-plasma-30_Cluster_299_sequences=11	11	138
VBP-V3-plasma-91_Cluster_1460_sequences=11	11	672

VBP-V3-plasma-53_Cluster_1037_sequences=11	11	299
VBP-V3-plasma-53_Cluster_1162_sequences=11	11	299
VBP-V3-plasma-53_Cluster_1216_sequences=11	11	299
VBP-V3-plasma-53_Cluster_1306_sequences=11	11	299
VBP-V3-plasma-53_Cluster_131_sequences=11	11	299
VBP-V3-plasma-53_Cluster_1323_sequences=11	11	299
VBP-V3-plasma-53_Cluster_20_sequences=11	11	299
VBP-V3-plasma-53_Cluster_2_sequences=11	11	299
VBP-V3-plasma-53_Cluster_321_sequences=11	11	299
VBP-V3-plasma-53_Cluster_473_sequences=11	11	299
VBP-V3-plasma-53_Cluster_491_sequences=11	11	299
VBP-V3-plasma-53_Cluster_252_sequences=11	11	299
VBP-V3-plasma-53_Cluster_1697_sequences=11	11	299
VBP-V3-plasma-53_Cluster_1111_sequences=11	11	299
VBP-V3-plasma-53_Cluster_134_sequences=11	11	299
VBP-V3-plasma-53_Cluster_217_sequences=11	11	299
VBP-V3-plasma-53_Cluster_579_sequences=11	11	299
VBP-V3-plasma-53_Cluster_123_sequences=11	11	299
VBP-V3-plasma-53_Cluster_574_sequences=11	11	299
VBP-V3-PBMC-72_Cluster_20392_sequences=10	10	490
VBP-V3-plasma-91_Cluster_173_sequences=10	10	672
VBP-V3-plasma-91_Cluster_60_sequences=10	10	672
VBP-V3-plasma-91_Cluster_78_sequences=10	10	672
VBP-V3-plasma-91_Cluster_142_sequences=10	10	672
VBP-V3-plasma-91_Cluster_1288_sequences=10	10	672
VBP-V3-plasma-91_Cluster_1524_sequences=10	10	672
VBP-V3-plasma-53_Cluster_1129_sequences=10	10	299
VBP-V3-plasma-53_Cluster_1314_sequences=10	10	299
VBP-V3-plasma-53_Cluster_337_sequences=10	10	299
VBP-V3-plasma-53_Cluster_521_sequences=10	10	299
VBP-V3-plasma-53_Cluster_776_sequences=10	10	299
VBP-V3-plasma-53_Cluster_791_sequences=10	10	299
VBP-V3-plasma-53_Cluster_966_sequences=10	10	299
VBP-V3-plasma-53_Cluster_530_sequences=10	10	299
VBP-V3-plasma-53_Cluster_870_sequences=10	10	299
VBP-V3-plasma-53_Cluster_1402_sequences=10	10	299
VBP-V3-plasma-53_Cluster_1624_sequences=10	10	299
VBP-V3-plasma-53_Cluster_283_sequences=10	10	299
VBP-V3-plasma-53_Cluster_325_sequences=10	10	299
VBP-V3-plasma-53_Cluster_559_sequences=10	10	299
VBP-V3-plasma-53_Cluster_757_sequences=10	10	299
VBP-V3-plasma-53_Cluster_2080_sequences=10	10	299
VBP-V3-PBMC-72_Cluster_19724_sequences=8	8	490
VBP-V3-PBMC-72_Cluster_2185_sequences=6	6	490
VBP-V3-PBMC-72_Cluster_552_sequences=5	5	490

VBP-V3-PBMC-72_Cluster_8822_sequences=5	5	490

Patient PCG3

haplotype	number of reads	timepoint
PCG-V3-plasma-9_Cluster_2_sequences=52292	52292	19
PCG-V3-plasma-55_Cluster_0_sequences=48564	48564	294
PCG-V3-PBMC-50_Cluster_2_sequences=28513	28513	282
PCG-V3-plasma-9_Cluster_7_sequences=19769	19769	19
PCG-V3-plasma-9_Cluster_48_sequences=4977	4977	19
PCG-V3-plasma-9_Cluster_11_sequences=4233	4233	19
PCG-V3-plasma-9_Cluster_24_sequences=2556	2556	19
PCG-V3-plasma-9_Cluster_1_sequences=2393	2393	19
PCG-V3-plasma-9_Cluster_37_sequences=2264	2264	19
PCG-V3-plasma-9_Cluster_34_sequences=1947	1947	19
PCG-V3-plasma-9_Cluster_30_sequences=1149	1149	19
PCG-V3-plasma-9_Cluster_38_sequences=934	934	19
PCG-V3-plasma-9_Cluster_96_sequences=738	738	19
PCG-V3-plasma-9_Cluster_49_sequences=539	539	19
PCG-V3-plasma-9_Cluster_52_sequences=529	529	19
PCG-V3-plasma-9_Cluster_62_sequences=519	519	19
PCG-V3-plasma-9_Cluster_69_sequences=377	377	19
PCG-V3-plasma-9_Cluster_13_sequences=310	310	19
PCG-V3-plasma-9_Cluster_146_sequences=271	271	19
PCG-V3-PBMC-50_Cluster_138_sequences=232	232	282
PCG-V3-plasma-55_Cluster_27_sequences=219	219	294
PCG-V3-plasma-55_Cluster_29_sequences=217	217	294
PCG-V3-plasma-9_Cluster_116_sequences=187	187	19
PCG-V3-plasma-9_Cluster_43_sequences=179	179	19
PCG-V3-plasma-9_Cluster_122_sequences=170	170	19
PCG-V3-plasma-9_Cluster_108_sequences=160	160	19
PCG-V3-plasma-9_Cluster_164_sequences=151	151	19
PCG-V3-plasma-9_Cluster_463_sequences=134	134	19
PCG-V3-plasma-9_Cluster_110_sequences=130	130	19
PCG-V3-plasma-55_Cluster_85_sequences=129	129	294
PCG-V3-plasma-9_Cluster_993_sequences=128	128	19
PCG-V3-plasma-55_Cluster_50_sequences=128	128	294
PCG-V3-plasma-9_Cluster_168_sequences=123	123	19
PCG-V3-plasma-9_Cluster_124_sequences=122	122	19
PCG-V3-plasma-9_Cluster_3_sequences=117	117	19
PCG-V3-plasma-9_Cluster_320_sequences=109	109	19
PCG-V3-plasma-9_Cluster_286_sequences=109	109	19
PCG-V3-plasma-9_Cluster_231_sequences=105	105	19
PCG-V3-plasma-9_Cluster_67_sequences=105	105	19
PCG-V3-plasma-9_Cluster_253_sequences=95	95	19
PCG-V3-PBMC-50_Cluster_287_sequences=93	93	282
PCG-V3-plasma-55_Cluster_15_sequences=88	88	294
PCG-V3-plasma-55_Cluster_97_sequences=87	87	294

PCG-V3-plasma-9_Cluster_1138_sequences=81	81	19
PCG-V3-PBMC-50_Cluster_199_sequences=80	80	282
PCG-V3-plasma-55_Cluster_1_sequences=79	79	294
PCG-V3-plasma-9_Cluster_181_sequences=78	78	19
PCG-V3-plasma-9_Cluster_450_sequences=78	78	19
PCG-V3-plasma-9_Cluster_388_sequences=78	78	19
PCG-V3-plasma-9_Cluster_279_sequences=78	78	19
PCG-V3-plasma-55_Cluster_151_sequences=75	75	294
PCG-V3-plasma-55_Cluster_53_sequences=71	71	294
PCG-V3-PBMC-50_Cluster_236_sequences=69	69	282
PCG-V3-plasma-9_Cluster_245_sequences=69	69	19
PCG-V3-plasma-9_Cluster_1093_sequences=66	66	19
PCG-V3-plasma-9_Cluster_143_sequences=65	65	19
PCG-V3-plasma-9_Cluster_662_sequences=65	65	19
PCG-V3-plasma-9_Cluster_601_sequences=65	65	19
PCG-V3-plasma-9_Cluster_9_sequences=64	64	19
PCG-V3-plasma-9_Cluster_169_sequences=63	63	19
PCG-V3-PBMC-50_Cluster_90_sequences=62	62	282
PCG-V3-plasma-9_Cluster_288_sequences=62	62	19
PCG-V3-plasma-9_Cluster_187_sequences=60	60	19
PCG-V3-plasma-55_Cluster_9_sequences=60	60	294
PCG-V3-plasma-9_Cluster_189_sequences=58	58	19
PCG-V3-plasma-55_Cluster_3_sequences=58	58	294
PCG-V3-plasma-55_Cluster_140_sequences=58	58	294
PCG-V3-plasma-9_Cluster_647_sequences=57	57	19
PCG-V3-plasma-55_Cluster_164_sequences=56	56	294
PCG-V3-PBMC-50_Cluster_670_sequences=55	55	282
PCG-V3-plasma-9_Cluster_813_sequences=52	52	19
PCG-V3-PBMC-50_Cluster_235_sequences=51	51	282
PCG-V3-plasma-9_Cluster_315_sequences=51	51	19
PCG-V3-plasma-55_Cluster_369_sequences=51	51	294
PCG-V3-plasma-9_Cluster_430_sequences=50	50	19
PCG-V3-plasma-9_Cluster_195_sequences=49	49	19
PCG-V3-plasma-9_Cluster_564_sequences=49	49	19
PCG-V3-plasma-9_Cluster_6329_sequences=49	49	19
PCG-V3-plasma-55_Cluster_231_sequences=49	49	294
PCG-V3-plasma-55_Cluster_61_sequences=49	49	294
PCG-V3-plasma-9_Cluster_10_sequences=48	48	19
PCG-V3-plasma-55_Cluster_183_sequences=48	48	294
PCG-V3-plasma-55_Cluster_91_sequences=48	48	294
PCG-V3-PBMC-50_Cluster_45_sequences=45	45	282
PCG-V3-plasma-9_Cluster_302_sequences=45	45	19
PCG-V3-plasma-9_Cluster_61_sequences=45	45	19
PCG-V3-plasma-9_Cluster_180_sequences=45	45	19
PCG-V3-PBMC-50_Cluster_5027_sequences=44	44	282

PCG-V3-PBMC-50_Cluster_1018_sequences=44	44	282
PCG-V3-PBMC-50_Cluster_828_sequences=44	44	282
PCG-V3-plasma-9_Cluster_209_sequences=43	43	19
PCG-V3-plasma-9_Cluster_361_sequences=42	42	19
PCG-V3-plasma-9_Cluster_433_sequences=42	42	19
PCG-V3-plasma-9_Cluster_369_sequences=42	42	19
PCG-V3-plasma-55_Cluster_67_sequences=42	42	294
PCG-V3-plasma-9_Cluster_120_sequences=41	41	19
PCG-V3-plasma-9_Cluster_363_sequences=41	41	19
PCG-V3-plasma-55_Cluster_435_sequences=41	41	294
PCG-V3-PBMC-50_Cluster_1961_sequences=40	40	282
PCG-V3-plasma-9_Cluster_660_sequences=40	40	19
PCG-V3-PBMC-50_Cluster_428_sequences=39	39	282
PCG-V3-plasma-9_Cluster_89_sequences=39	39	19
PCG-V3-plasma-9_Cluster_138_sequences=39	39	19
PCG-V3-plasma-9_Cluster_533_sequences=39	39	19
PCG-V3-plasma-9_Cluster_6954_sequences=39	39	19
PCG-V3-plasma-55_Cluster_10_sequences=39	39	294
PCG-V3-plasma-55_Cluster_299_sequences=39	39	294
PCG-V3-plasma-9_Cluster_523_sequences=38	38	19
PCG-V3-plasma-9_Cluster_60_sequences=38	38	19
PCG-V3-plasma-9_Cluster_536_sequences=38	38	19
PCG-V3-plasma-9_Cluster_188_sequences=38	38	19
PCG-V3-PBMC-50_Cluster_4372_sequences=37	37	282
PCG-V3-plasma-9_Cluster_243_sequences=37	37	19
PCG-V3-plasma-9_Cluster_752_sequences=37	37	19
PCG-V3-plasma-55_Cluster_152_sequences=37	37	294
PCG-V3-plasma-55_Cluster_185_sequences=37	37	294
PCG-V3-plasma-55_Cluster_2454_sequences=37	37	294
PCG-V3-plasma-55_Cluster_58_sequences=37	37	294
PCG-V3-plasma-9_Cluster_592_sequences=36	36	19
PCG-V3-plasma-55_Cluster_80_sequences=36	36	294
PCG-V3-plasma-55_Cluster_641_sequences=36	36	294
PCG-V3-PBMC-50_Cluster_2635_sequences=35	35	282
PCG-V3-PBMC-50_Cluster_676_sequences=35	35	282
PCG-V3-plasma-9_Cluster_12_sequences=35	35	19
PCG-V3-plasma-9_Cluster_1091_sequences=35	35	19
PCG-V3-plasma-9_Cluster_230_sequences=35	35	19
PCG-V3-plasma-9_Cluster_157_sequences=35	35	19
PCG-V3-plasma-55_Cluster_440_sequences=35	35	294
PCG-V3-plasma-55_Cluster_81_sequences=35	35	294
PCG-V3-PBMC-50_Cluster_115_sequences=34	34	282
PCG-V3-PBMC-50_Cluster_1451_sequences=32	32	282
PCG-V3-PBMC-50_Cluster_402_sequences=32	32	282
PCG-V3-plasma-9_Cluster_323_sequences=32	32	19

PCG-V3-plasma-55_Cluster_11_sequences=32	32	294
PCG-V3-plasma-55_Cluster_345_sequences=32	32	294
PCG-V3-plasma-55_Cluster_772_sequences=32	32	294
PCG-V3-plasma-55_Cluster_94_sequences=32	32	294
PCG-V3-plasma-55_Cluster_555_sequences=32	32	294
PCG-V3-plasma-55_Cluster_42_sequences=32	32	294
PCG-V3-plasma-9_Cluster_576_sequences=31	31	19
PCG-V3-plasma-9_Cluster_507_sequences=31	31	19
PCG-V3-plasma-55_Cluster_142_sequences=31	31	294
PCG-V3-PBMC-50_Cluster_120_sequences=30	30	282
PCG-V3-PBMC-50_Cluster_67_sequences=30	30	282
PCG-V3-plasma-9_Cluster_834_sequences=30	30	19
PCG-V3-plasma-9_Cluster_884_sequences=30	30	19
PCG-V3-plasma-9_Cluster_756_sequences=30	30	19
PCG-V3-PBMC-50_Cluster_1117_sequences=29	29	282
PCG-V3-PBMC-50_Cluster_2179_sequences=29	29	282
PCG-V3-PBMC-50_Cluster_773_sequences=29	29	282
PCG-V3-plasma-9_Cluster_375_sequences=29	29	19
PCG-V3-plasma-9_Cluster_1621_sequences=29	29	19
PCG-V3-plasma-55_Cluster_7_sequences=29	29	294
PCG-V3-plasma-55_Cluster_12_sequences=29	29	294
PCG-V3-PBMC-50_Cluster_2448_sequences=28	28	282
PCG-V3-plasma-9_Cluster_573_sequences=28	28	19
PCG-V3-plasma-9_Cluster_537_sequences=28	28	19
PCG-V3-plasma-9_Cluster_901_sequences=28	28	19
PCG-V3-plasma-55_Cluster_155_sequences=28	28	294
PCG-V3-plasma-55_Cluster_289_sequences=28	28	294
PCG-V3-plasma-55_Cluster_380_sequences=28	28	294
PCG-V3-PBMC-50_Cluster_1288_sequences=27	27	282
PCG-V3-PBMC-50_Cluster_185_sequences=27	27	282
PCG-V3-PBMC-50_Cluster_710_sequences=27	27	282
PCG-V3-plasma-9_Cluster_410_sequences=27	27	19
PCG-V3-plasma-55_Cluster_113_sequences=27	27	294
PCG-V3-plasma-55_Cluster_204_sequences=27	27	294
PCG-V3-PBMC-50_Cluster_169_sequences=26	26	282
PCG-V3-PBMC-50_Cluster_309_sequences=26	26	282
PCG-V3-plasma-9_Cluster_1119_sequences=26	26	19
PCG-V3-plasma-9_Cluster_337_sequences=26	26	19
PCG-V3-plasma-9_Cluster_806_sequences=26	26	19
PCG-V3-plasma-9_Cluster_212_sequences=26	26	19
PCG-V3-plasma-9_Cluster_3691_sequences=26	26	19
PCG-V3-plasma-55_Cluster_208_sequences=26	26	294
PCG-V3-plasma-55_Cluster_190_sequences=26	26	294
PCG-V3-plasma-55_Cluster_19_sequences=26	26	294
PCG-V3-PBMC-50_Cluster_1003_sequences=25	25	282

PCG-V3-PBMC-50_Cluster_1486_sequences=25	25	282
PCG-V3-PBMC-50_Cluster_1006_sequences=25	25	282
PCG-V3-plasma-9_Cluster_1154_sequences=25	25	19
PCG-V3-plasma-9_Cluster_2184_sequences=25	25	19
PCG-V3-plasma-9_Cluster_472_sequences=25	25	19
PCG-V3-plasma-9_Cluster_261_sequences=25	25	19
PCG-V3-plasma-9_Cluster_1131_sequences=25	25	19
PCG-V3-plasma-9_Cluster_798_sequences=25	25	19
PCG-V3-plasma-55_Cluster_451_sequences=25	25	294
PCG-V3-plasma-55_Cluster_1361_sequences=25	25	294
PCG-V3-plasma-55_Cluster_65_sequences=25	25	294
PCG-V3-PBMC-50_Cluster_275_sequences=24	24	282
PCG-V3-PBMC-50_Cluster_4237_sequences=24	24	282
PCG-V3-plasma-9_Cluster_174_sequences=24	24	19
PCG-V3-plasma-9_Cluster_219_sequences=24	24	19
PCG-V3-plasma-9_Cluster_3996_sequences=24	24	19
PCG-V3-plasma-9_Cluster_422_sequences=24	24	19
PCG-V3-plasma-9_Cluster_269_sequences=24	24	19
PCG-V3-plasma-9_Cluster_236_sequences=24	24	19
PCG-V3-plasma-55_Cluster_267_sequences=24	24	294
PCG-V3-plasma-55_Cluster_386_sequences=24	24	294
PCG-V3-plasma-55_Cluster_615_sequences=24	24	294
PCG-V3-plasma-55_Cluster_619_sequences=24	24	294
PCG-V3-plasma-55_Cluster_188_sequences=24	24	294
PCG-V3-PBMC-50_Cluster_274_sequences=23	23	282
PCG-V3-PBMC-50_Cluster_999_sequences=23	23	282
PCG-V3-PBMC-50_Cluster_1491_sequences=23	23	282
PCG-V3-PBMC-50_Cluster_1560_sequences=23	23	282
PCG-V3-PBMC-50_Cluster_1994_sequences=23	23	282
PCG-V3-plasma-9_Cluster_139_sequences=23	23	19
PCG-V3-plasma-9_Cluster_414_sequences=23	23	19
PCG-V3-plasma-9_Cluster_304_sequences=23	23	19
PCG-V3-plasma-9_Cluster_1218_sequences=23	23	19
PCG-V3-plasma-9_Cluster_284_sequences=23	23	19
PCG-V3-plasma-55_Cluster_49_sequences=23	23	294
PCG-V3-plasma-55_Cluster_479_sequences=23	23	294
PCG-V3-plasma-55_Cluster_82_sequences=23	23	294
PCG-V3-PBMC-50_Cluster_1649_sequences=22	22	282
PCG-V3-PBMC-50_Cluster_118_sequences=22	22	282
PCG-V3-plasma-9_Cluster_413_sequences=22	22	19
PCG-V3-plasma-9_Cluster_186_sequences=22	22	19
PCG-V3-plasma-9_Cluster_4461_sequences=22	22	19
PCG-V3-plasma-9_Cluster_1662_sequences=22	22	19
PCG-V3-plasma-9_Cluster_539_sequences=22	22	19
PCG-V3-plasma-9_Cluster_2401_sequences=22	22	19

PCG-V3-plasma-55_Cluster_59_sequences=22	22	294
PCG-V3-plasma-55_Cluster_400_sequences=22	22	294
PCG-V3-PBMC-50_Cluster_1874_sequences=21	21	282
PCG-V3-PBMC-50_Cluster_6010_sequences=21	21	282
PCG-V3-PBMC-50_Cluster_1024_sequences=21	21	282
PCG-V3-PBMC-50_Cluster_561_sequences=21	21	282
PCG-V3-PBMC-50_Cluster_647_sequences=21	21	282
PCG-V3-PBMC-50_Cluster_13_sequences=21	21	282
PCG-V3-plasma-9_Cluster_2483_sequences=21	21	19
PCG-V3-plasma-9_Cluster_492_sequences=21	21	19
PCG-V3-plasma-9_Cluster_538_sequences=21	21	19
PCG-V3-plasma-9_Cluster_94_sequences=21	21	19
PCG-V3-plasma-9_Cluster_531_sequences=21	21	19
PCG-V3-plasma-9_Cluster_501_sequences=21	21	19
PCG-V3-plasma-9_Cluster_1238_sequences=21	21	19
PCG-V3-plasma-9_Cluster_345_sequences=21	21	19
PCG-V3-plasma-9_Cluster_6328_sequences=21	21	19
PCG-V3-plasma-9_Cluster_374_sequences=21	21	19
PCG-V3-plasma-55_Cluster_1898_sequences=21	21	294
PCG-V3-plasma-55_Cluster_820_sequences=21	21	294
PCG-V3-plasma-55_Cluster_153_sequences=21	21	294
PCG-V3-plasma-55_Cluster_538_sequences=21	21	294
PCG-V3-plasma-55_Cluster_219_sequences=21	21	294
PCG-V3-PBMC-50_Cluster_5217_sequences=20	20	282
PCG-V3-PBMC-50_Cluster_26_sequences=20	20	282
PCG-V3-plasma-9_Cluster_1298_sequences=20	20	19
PCG-V3-plasma-9_Cluster_1516_sequences=20	20	19
PCG-V3-plasma-9_Cluster_171_sequences=20	20	19
PCG-V3-plasma-9_Cluster_486_sequences=20	20	19
PCG-V3-plasma-9_Cluster_925_sequences=20	20	19
PCG-V3-plasma-9_Cluster_329_sequences=20	20	19
PCG-V3-plasma-9_Cluster_1071_sequences=20	20	19
PCG-V3-plasma-9_Cluster_1973_sequences=20	20	19
PCG-V3-plasma-9_Cluster_274_sequences=20	20	19
PCG-V3-plasma-9_Cluster_50_sequences=20	20	19
PCG-V3-plasma-9_Cluster_689_sequences=20	20	19
PCG-V3-plasma-9_Cluster_947_sequences=20	20	19
PCG-V3-plasma-9_Cluster_23_sequences=20	20	19
PCG-V3-plasma-55_Cluster_1250_sequences=20	20	294
PCG-V3-plasma-55_Cluster_584_sequences=20	20	294
PCG-V3-plasma-55_Cluster_405_sequences=20	20	294
PCG-V3-plasma-55_Cluster_259_sequences=20	20	294
PCG-V3-plasma-55_Cluster_275_sequences=20	20	294
PCG-V3-PBMC-50_Cluster_5594_sequences=19	19	282
PCG-V3-PBMC-50_Cluster_1471_sequences=19	19	282

PCG-V3-PBMC-50_Cluster_793_sequences=19	19	282
PCG-V3-PBMC-50_Cluster_60_sequences=19	19	282
PCG-V3-plasma-9_Cluster_715_sequences=19	19	19
PCG-V3-plasma-9_Cluster_871_sequences=19	19	19
PCG-V3-plasma-9_Cluster_910_sequences=19	19	19
PCG-V3-plasma-9_Cluster_1176_sequences=19	19	19
PCG-V3-plasma-9_Cluster_334_sequences=19	19	19
PCG-V3-plasma-9_Cluster_499_sequences=19	19	19
PCG-V3-plasma-9_Cluster_1748_sequences=19	19	19
PCG-V3-plasma-55_Cluster_272_sequences=19	19	294
PCG-V3-plasma-55_Cluster_592_sequences=19	19	294
PCG-V3-plasma-55_Cluster_419_sequences=19	19	294
PCG-V3-plasma-55_Cluster_70_sequences=19	19	294
PCG-V3-PBMC-50_Cluster_4009_sequences=18	18	282
PCG-V3-PBMC-50_Cluster_1128_sequences=18	18	282
PCG-V3-PBMC-50_Cluster_4644_sequences=18	18	282
PCG-V3-PBMC-50_Cluster_5065_sequences=18	18	282
PCG-V3-PBMC-50_Cluster_1831_sequences=18	18	282
PCG-V3-PBMC-50_Cluster_2389_sequences=18	18	282
PCG-V3-PBMC-50_Cluster_2644_sequences=18	18	282
PCG-V3-PBMC-50_Cluster_303_sequences=18	18	282
PCG-V3-PBMC-50_Cluster_346_sequences=18	18	282
PCG-V3-PBMC-50_Cluster_3656_sequences=18	18	282
PCG-V3-PBMC-50_Cluster_3948_sequences=18	18	282
PCG-V3-plasma-9_Cluster_1338_sequences=18	18	19
PCG-V3-plasma-9_Cluster_330_sequences=18	18	19
PCG-V3-plasma-9_Cluster_3555_sequences=18	18	19
PCG-V3-plasma-9_Cluster_913_sequences=18	18	19
PCG-V3-plasma-9_Cluster_1099_sequences=18	18	19
PCG-V3-plasma-9_Cluster_1122_sequences=18	18	19
PCG-V3-plasma-9_Cluster_1172_sequences=18	18	19
PCG-V3-plasma-9_Cluster_135_sequences=18	18	19
PCG-V3-plasma-9_Cluster_2723_sequences=18	18	19
PCG-V3-plasma-9_Cluster_825_sequences=18	18	19
PCG-V3-plasma-9_Cluster_1796_sequences=18	18	19
PCG-V3-plasma-9_Cluster_156_sequences=18	18	19
PCG-V3-plasma-9_Cluster_794_sequences=18	18	19
PCG-V3-plasma-9_Cluster_965_sequences=18	18	19
PCG-V3-plasma-55_Cluster_149_sequences=18	18	294
PCG-V3-plasma-55_Cluster_552_sequences=18	18	294
PCG-V3-plasma-55_Cluster_947_sequences=18	18	294
PCG-V3-plasma-55_Cluster_21_sequences=18	18	294
PCG-V3-plasma-55_Cluster_100_sequences=18	18	294
PCG-V3-plasma-55_Cluster_34_sequences=18	18	294
PCG-V3-plasma-55_Cluster_357_sequences=18	18	294

PCG-V3-plasma-55_Cluster_499_sequences=18	18	294
PCG-V3-PBMC-50_Cluster_1424_sequences=17	17	282
PCG-V3-PBMC-50_Cluster_4804_sequences=17	17	282
PCG-V3-PBMC-50_Cluster_644_sequences=17	17	282
PCG-V3-PBMC-50_Cluster_6234_sequences=17	17	282
PCG-V3-PBMC-50_Cluster_3976_sequences=17	17	282
PCG-V3-PBMC-50_Cluster_3022_sequences=17	17	282
PCG-V3-PBMC-50_Cluster_5777_sequences=17	17	282
PCG-V3-PBMC-50_Cluster_1671_sequences=17	17	282
PCG-V3-PBMC-50_Cluster_370_sequences=17	17	282
PCG-V3-PBMC-50_Cluster_945_sequences=17	17	282
PCG-V3-PBMC-50_Cluster_5936_sequences=17	17	282
PCG-V3-PBMC-50_Cluster_4409_sequences=17	17	282
PCG-V3-plasma-9_Cluster_1163_sequences=17	17	19
PCG-V3-plasma-9_Cluster_3250_sequences=17	17	19
PCG-V3-plasma-9_Cluster_445_sequences=17	17	19
PCG-V3-plasma-9_Cluster_675_sequences=17	17	19
PCG-V3-plasma-9_Cluster_379_sequences=17	17	19
PCG-V3-plasma-9_Cluster_2313_sequences=17	17	19
PCG-V3-plasma-9_Cluster_1077_sequences=17	17	19
PCG-V3-plasma-9_Cluster_1240_sequences=17	17	19
PCG-V3-plasma-9_Cluster_131_sequences=17	17	19
PCG-V3-plasma-9_Cluster_256_sequences=17	17	19
PCG-V3-plasma-9_Cluster_561_sequences=17	17	19
PCG-V3-plasma-9_Cluster_751_sequences=17	17	19
PCG-V3-plasma-9_Cluster_1664_sequences=17	17	19
PCG-V3-plasma-9_Cluster_640_sequences=17	17	19
PCG-V3-plasma-9_Cluster_8276_sequences=17	17	19
PCG-V3-plasma-9_Cluster_1747_sequences=17	17	19
PCG-V3-plasma-9_Cluster_580_sequences=17	17	19
PCG-V3-plasma-9_Cluster_75_sequences=17	17	19
PCG-V3-plasma-9_Cluster_2384_sequences=17	17	19
PCG-V3-plasma-55_Cluster_247_sequences=17	17	294
PCG-V3-plasma-55_Cluster_32_sequences=17	17	294
PCG-V3-plasma-55_Cluster_709_sequences=17	17	294
PCG-V3-plasma-55_Cluster_161_sequences=17	17	294
PCG-V3-plasma-55_Cluster_406_sequences=17	17	294
PCG-V3-plasma-55_Cluster_296_sequences=17	17	294
PCG-V3-plasma-55_Cluster_2692_sequences=17	17	294
PCG-V3-plasma-55_Cluster_41_sequences=17	17	294
PCG-V3-plasma-55_Cluster_535_sequences=17	17	294
PCG-V3-plasma-55_Cluster_953_sequences=17	17	294
PCG-V3-PBMC-50_Cluster_1008_sequences=16	16	282
PCG-V3-PBMC-50_Cluster_4354_sequences=16	16	282
PCG-V3-PBMC-50_Cluster_1660_sequences=16	16	282

PCG-V3-PBMC-50_Cluster_1668_sequences=16	16	282
PCG-V3-PBMC-50_Cluster_253_sequences=16	16	282
PCG-V3-PBMC-50_Cluster_7878_sequences=16	16	282
PCG-V3-PBMC-50_Cluster_7910_sequences=16	16	282
PCG-V3-PBMC-50_Cluster_2391_sequences=16	16	282
PCG-V3-PBMC-50_Cluster_3922_sequences=16	16	282
PCG-V3-PBMC-50_Cluster_3725_sequences=16	16	282
PCG-V3-plasma-9_Cluster_1088_sequences=16	16	19
PCG-V3-plasma-9_Cluster_1367_sequences=16	16	19
PCG-V3-plasma-9_Cluster_144_sequences=16	16	19
PCG-V3-plasma-9_Cluster_424_sequences=16	16	19
PCG-V3-plasma-9_Cluster_443_sequences=16	16	19
PCG-V3-plasma-9_Cluster_681_sequences=16	16	19
PCG-V3-plasma-9_Cluster_1562_sequences=16	16	19
PCG-V3-plasma-9_Cluster_314_sequences=16	16	19
PCG-V3-plasma-9_Cluster_467_sequences=16	16	19
PCG-V3-plasma-9_Cluster_673_sequences=16	16	19
PCG-V3-plasma-9_Cluster_827_sequences=16	16	19
PCG-V3-plasma-9_Cluster_983_sequences=16	16	19
PCG-V3-plasma-9_Cluster_1010_sequences=16	16	19
PCG-V3-plasma-9_Cluster_656_sequences=16	16	19
PCG-V3-plasma-9_Cluster_134_sequences=16	16	19
PCG-V3-plasma-9_Cluster_431_sequences=16	16	19
PCG-V3-plasma-9_Cluster_2652_sequences=16	16	19
PCG-V3-plasma-9_Cluster_525_sequences=16	16	19
PCG-V3-plasma-55_Cluster_1137_sequences=16	16	294
PCG-V3-plasma-55_Cluster_1360_sequences=16	16	294
PCG-V3-plasma-55_Cluster_215_sequences=16	16	294
PCG-V3-plasma-55_Cluster_350_sequences=16	16	294
PCG-V3-plasma-55_Cluster_632_sequences=16	16	294
PCG-V3-plasma-55_Cluster_120_sequences=16	16	294
PCG-V3-plasma-55_Cluster_129_sequences=16	16	294
PCG-V3-plasma-55_Cluster_260_sequences=16	16	294
PCG-V3-plasma-55_Cluster_262_sequences=16	16	294
PCG-V3-plasma-55_Cluster_398_sequences=16	16	294
PCG-V3-plasma-55_Cluster_823_sequences=16	16	294
PCG-V3-plasma-55_Cluster_4039_sequences=16	16	294
PCG-V3-PBMC-50_Cluster_2590_sequences=15	15	282
PCG-V3-PBMC-50_Cluster_2610_sequences=15	15	282
PCG-V3-PBMC-50_Cluster_415_sequences=15	15	282
PCG-V3-PBMC-50_Cluster_3416_sequences=15	15	282
PCG-V3-PBMC-50_Cluster_5243_sequences=15	15	282
PCG-V3-plasma-9_Cluster_1018_sequences=15	15	19
PCG-V3-plasma-9_Cluster_1210_sequences=15	15	19
PCG-V3-plasma-9_Cluster_1592_sequences=15	15	19

PCG-V3-plasma-9_Cluster_2735_sequences=15	15	19
PCG-V3-plasma-9_Cluster_36_sequences=15	15	19
PCG-V3-plasma-9_Cluster_765_sequences=15	15	19
PCG-V3-plasma-9_Cluster_883_sequences=15	15	19
PCG-V3-plasma-9_Cluster_996_sequences=15	15	19
PCG-V3-plasma-9_Cluster_872_sequences=15	15	19
PCG-V3-plasma-9_Cluster_381_sequences=15	15	19
PCG-V3-plasma-9_Cluster_1315_sequences=15	15	19
PCG-V3-plasma-9_Cluster_2689_sequences=15	15	19
PCG-V3-plasma-9_Cluster_623_sequences=15	15	19
PCG-V3-plasma-9_Cluster_81_sequences=15	15	19
PCG-V3-plasma-9_Cluster_1067_sequences=15	15	19
PCG-V3-plasma-9_Cluster_1667_sequences=15	15	19
PCG-V3-plasma-9_Cluster_251_sequences=15	15	19
PCG-V3-plasma-9_Cluster_3649_sequences=15	15	19
PCG-V3-plasma-9_Cluster_20_sequences=15	15	19
PCG-V3-plasma-55_Cluster_1204_sequences=15	15	294
PCG-V3-plasma-55_Cluster_396_sequences=15	15	294
PCG-V3-plasma-55_Cluster_1062_sequences=15	15	294
PCG-V3-plasma-55_Cluster_224_sequences=15	15	294
PCG-V3-plasma-55_Cluster_62_sequences=15	15	294
PCG-V3-plasma-55_Cluster_196_sequences=15	15	294
PCG-V3-plasma-55_Cluster_639_sequences=15	15	294
PCG-V3-plasma-55_Cluster_464_sequences=15	15	294
PCG-V3-plasma-55_Cluster_530_sequences=15	15	294
PCG-V3-PBMC-50_Cluster_861_sequences=14	14	282
PCG-V3-PBMC-50_Cluster_4614_sequences=14	14	282
PCG-V3-PBMC-50_Cluster_7167_sequences=14	14	282
PCG-V3-PBMC-50_Cluster_749_sequences=14	14	282
PCG-V3-PBMC-50_Cluster_845_sequences=14	14	282
PCG-V3-PBMC-50_Cluster_164_sequences=14	14	282
PCG-V3-PBMC-50_Cluster_19375_sequences=14	14	282
PCG-V3-PBMC-50_Cluster_690_sequences=14	14	282
PCG-V3-plasma-9_Cluster_1045_sequences=14	14	19
PCG-V3-plasma-9_Cluster_1132_sequences=14	14	19
PCG-V3-plasma-9_Cluster_1231_sequences=14	14	19
PCG-V3-plasma-9_Cluster_1464_sequences=14	14	19
PCG-V3-plasma-9_Cluster_1608_sequences=14	14	19
PCG-V3-plasma-9_Cluster_1656_sequences=14	14	19
PCG-V3-plasma-9_Cluster_2392_sequences=14	14	19
PCG-V3-plasma-9_Cluster_1062_sequences=14	14	19
PCG-V3-plasma-9_Cluster_1359_sequences=14	14	19
PCG-V3-plasma-9_Cluster_366_sequences=14	14	19
PCG-V3-plasma-9_Cluster_1089_sequences=14	14	19
PCG-V3-plasma-9_Cluster_5044_sequences=14	14	19

PCG-V3-plasma-9_Cluster_2287_sequences=14	14	19
PCG-V3-plasma-9_Cluster_1393_sequences=14	14	19
PCG-V3-plasma-9_Cluster_133_sequences=14	14	19
PCG-V3-plasma-55_Cluster_1224_sequences=14	14	294
PCG-V3-plasma-55_Cluster_159_sequences=14	14	294
PCG-V3-plasma-55_Cluster_229_sequences=14	14	294
PCG-V3-plasma-55_Cluster_438_sequences=14	14	294
PCG-V3-plasma-55_Cluster_459_sequences=14	14	294
PCG-V3-plasma-55_Cluster_646_sequences=14	14	294
PCG-V3-plasma-55_Cluster_827_sequences=14	14	294
PCG-V3-plasma-55_Cluster_917_sequences=14	14	294
PCG-V3-plasma-55_Cluster_71_sequences=14	14	294
PCG-V3-plasma-55_Cluster_1345_sequences=14	14	294
PCG-V3-plasma-55_Cluster_150_sequences=14	14	294
PCG-V3-plasma-55_Cluster_377_sequences=14	14	294
PCG-V3-plasma-55_Cluster_2420_sequences=14	14	294
PCG-V3-plasma-55_Cluster_295_sequences=14	14	294
PCG-V3-plasma-55_Cluster_349_sequences=14	14	294
PCG-V3-plasma-55_Cluster_453_sequences=14	14	294
PCG-V3-plasma-55_Cluster_461_sequences=14	14	294
PCG-V3-plasma-55_Cluster_485_sequences=14	14	294
PCG-V3-plasma-55_Cluster_556_sequences=14	14	294
PCG-V3-plasma-55_Cluster_579_sequences=14	14	294
PCG-V3-PBMC-50_Cluster_1400_sequences=13	13	282
PCG-V3-PBMC-50_Cluster_1553_sequences=13	13	282
PCG-V3-PBMC-50_Cluster_2399_sequences=13	13	282
PCG-V3-PBMC-50_Cluster_5953_sequences=13	13	282
PCG-V3-PBMC-50_Cluster_638_sequences=13	13	282
PCG-V3-PBMC-50_Cluster_1383_sequences=13	13	282
PCG-V3-PBMC-50_Cluster_2646_sequences=13	13	282
PCG-V3-PBMC-50_Cluster_682_sequences=13	13	282
PCG-V3-PBMC-50_Cluster_822_sequences=13	13	282
PCG-V3-PBMC-50_Cluster_1892_sequences=13	13	282
PCG-V3-PBMC-50_Cluster_5138_sequences=13	13	282
PCG-V3-plasma-9_Cluster_2005_sequences=13	13	19
PCG-V3-plasma-9_Cluster_258_sequences=13	13	19
PCG-V3-plasma-9_Cluster_4094_sequences=13	13	19
PCG-V3-plasma-9_Cluster_535_sequences=13	13	19
PCG-V3-plasma-9_Cluster_964_sequences=13	13	19
PCG-V3-plasma-9_Cluster_998_sequences=13	13	19
PCG-V3-plasma-9_Cluster_677_sequences=13	13	19
PCG-V3-plasma-9_Cluster_1422_sequences=13	13	19
PCG-V3-plasma-9_Cluster_2081_sequences=13	13	19
PCG-V3-plasma-9_Cluster_2596_sequences=13	13	19
PCG-V3-plasma-9_Cluster_4008_sequences=13	13	19

PCG-V3-plasma-9_Cluster_905_sequences=13	13	19
PCG-V3-plasma-9_Cluster_421_sequences=13	13	19
PCG-V3-plasma-9_Cluster_1233_sequences=13	13	19
PCG-V3-plasma-55_Cluster_1131_sequences=13	13	294
PCG-V3-plasma-55_Cluster_298_sequences=13	13	294
PCG-V3-plasma-55_Cluster_750_sequences=13	13	294
PCG-V3-plasma-55_Cluster_862_sequences=13	13	294
PCG-V3-plasma-55_Cluster_891_sequences=13	13	294
PCG-V3-plasma-55_Cluster_93_sequences=13	13	294
PCG-V3-plasma-55_Cluster_96_sequences=13	13	294
PCG-V3-plasma-55_Cluster_221_sequences=13	13	294
PCG-V3-plasma-55_Cluster_571_sequences=13	13	294
PCG-V3-plasma-55_Cluster_1143_sequences=13	13	294
PCG-V3-plasma-55_Cluster_1720_sequences=13	13	294
PCG-V3-plasma-55_Cluster_889_sequences=13	13	294
PCG-V3-plasma-55_Cluster_110_sequences=13	13	294
PCG-V3-plasma-55_Cluster_197_sequences=13	13	294
PCG-V3-plasma-55_Cluster_408_sequences=13	13	294
PCG-V3-plasma-55_Cluster_620_sequences=13	13	294
PCG-V3-plasma-55_Cluster_645_sequences=13	13	294
PCG-V3-plasma-55_Cluster_732_sequences=13	13	294
PCG-V3-plasma-55_Cluster_735_sequences=13	13	294
PCG-V3-plasma-55_Cluster_4056_sequences=13	13	294
PCG-V3-PBMC-50_Cluster_5166_sequences=12	12	282
PCG-V3-PBMC-50_Cluster_5087_sequences=12	12	282
PCG-V3-PBMC-50_Cluster_1754_sequences=12	12	282
PCG-V3-PBMC-50_Cluster_2385_sequences=12	12	282
PCG-V3-PBMC-50_Cluster_4292_sequences=12	12	282
PCG-V3-PBMC-50_Cluster_12_sequences=12	12	282
PCG-V3-PBMC-50_Cluster_1301_sequences=12	12	282
PCG-V3-PBMC-50_Cluster_420_sequences=12	12	282
PCG-V3-PBMC-50_Cluster_1285_sequences=12	12	282
PCG-V3-PBMC-50_Cluster_4657_sequences=12	12	282
PCG-V3-plasma-9_Cluster_103_sequences=12	12	19
PCG-V3-plasma-9_Cluster_111_sequences=12	12	19
PCG-V3-plasma-9_Cluster_1443_sequences=12	12	19
PCG-V3-plasma-9_Cluster_1831_sequences=12	12	19
PCG-V3-plasma-9_Cluster_224_sequences=12	12	19
PCG-V3-plasma-9_Cluster_2282_sequences=12	12	19
PCG-V3-plasma-9_Cluster_299_sequences=12	12	19
PCG-V3-plasma-9_Cluster_382_sequences=12	12	19
PCG-V3-plasma-9_Cluster_42_sequences=12	12	19
PCG-V3-plasma-9_Cluster_631_sequences=12	12	19
PCG-V3-plasma-9_Cluster_668_sequences=12	12	19
PCG-V3-plasma-9_Cluster_767_sequences=12	12	19

PCG-V3-plasma-9_Cluster_1019_sequences=12	12	19
PCG-V3-plasma-9_Cluster_359_sequences=12	12	19
PCG-V3-plasma-9_Cluster_226_sequences=12	12	19
PCG-V3-plasma-9_Cluster_3532_sequences=12	12	19
PCG-V3-plasma-9_Cluster_419_sequences=12	12	19
PCG-V3-plasma-9_Cluster_435_sequences=12	12	19
PCG-V3-plasma-9_Cluster_581_sequences=12	12	19
PCG-V3-plasma-9_Cluster_2128_sequences=12	12	19
PCG-V3-plasma-9_Cluster_1236_sequences=12	12	19
PCG-V3-plasma-9_Cluster_2027_sequences=12	12	19
PCG-V3-plasma-9_Cluster_206_sequences=12	12	19
PCG-V3-plasma-9_Cluster_468_sequences=12	12	19
PCG-V3-plasma-9_Cluster_1086_sequences=12	12	19
PCG-V3-plasma-9_Cluster_518_sequences=12	12	19
PCG-V3-plasma-9_Cluster_1245_sequences=12	12	19
PCG-V3-plasma-9_Cluster_1335_sequences=12	12	19
PCG-V3-plasma-55_Cluster_1437_sequences=12	12	294
PCG-V3-plasma-55_Cluster_217_sequences=12	12	294
PCG-V3-plasma-55_Cluster_269_sequences=12	12	294
PCG-V3-plasma-55_Cluster_344_sequences=12	12	294
PCG-V3-plasma-55_Cluster_513_sequences=12	12	294
PCG-V3-plasma-55_Cluster_597_sequences=12	12	294
PCG-V3-plasma-55_Cluster_736_sequences=12	12	294
PCG-V3-plasma-55_Cluster_69_sequences=12	12	294
PCG-V3-plasma-55_Cluster_339_sequences=12	12	294
PCG-V3-plasma-55_Cluster_460_sequences=12	12	294
PCG-V3-plasma-55_Cluster_522_sequences=12	12	294
PCG-V3-plasma-55_Cluster_101_sequences=12	12	294
PCG-V3-plasma-55_Cluster_1047_sequences=12	12	294
PCG-V3-plasma-55_Cluster_1053_sequences=12	12	294
PCG-V3-plasma-55_Cluster_1136_sequences=12	12	294
PCG-V3-plasma-55_Cluster_549_sequences=12	12	294
PCG-V3-plasma-55_Cluster_603_sequences=12	12	294
PCG-V3-plasma-55_Cluster_683_sequences=12	12	294
PCG-V3-PBMC-50_Cluster_2962_sequences=11	11	282
PCG-V3-PBMC-50_Cluster_296_sequences=11	11	282
PCG-V3-PBMC-50_Cluster_1014_sequences=11	11	282
PCG-V3-PBMC-50_Cluster_6459_sequences=11	11	282
PCG-V3-PBMC-50_Cluster_4268_sequences=11	11	282
PCG-V3-PBMC-50_Cluster_716_sequences=11	11	282
PCG-V3-PBMC-50_Cluster_1029_sequences=11	11	282
PCG-V3-PBMC-50_Cluster_1072_sequences=11	11	282
PCG-V3-PBMC-50_Cluster_1238_sequences=11	11	282
PCG-V3-PBMC-50_Cluster_1436_sequences=11	11	282
PCG-V3-PBMC-50_Cluster_1819_sequences=11	11	282

PCG-V3-PBMC-50_Cluster_1915_sequences=11	11	282
PCG-V3-PBMC-50_Cluster_1945_sequences=11	11	282
PCG-V3-PBMC-50_Cluster_2022_sequences=11	11	282
PCG-V3-PBMC-50_Cluster_2036_sequences=11	11	282
PCG-V3-PBMC-50_Cluster_27_sequences=11	11	282
PCG-V3-PBMC-50_Cluster_3293_sequences=11	11	282
PCG-V3-PBMC-50_Cluster_3319_sequences=11	11	282
PCG-V3-PBMC-50_Cluster_5251_sequences=11	11	282
PCG-V3-PBMC-50_Cluster_913_sequences=11	11	282
PCG-V3-PBMC-50_Cluster_6225_sequences=11	11	282
PCG-V3-plasma-9_Cluster_1533_sequences=11	11	19
PCG-V3-plasma-9_Cluster_1649_sequences=11	11	19
PCG-V3-plasma-9_Cluster_371_sequences=11	11	19
PCG-V3-plasma-9_Cluster_4796_sequences=11	11	19
PCG-V3-plasma-9_Cluster_491_sequences=11	11	19
PCG-V3-plasma-9_Cluster_572_sequences=11	11	19
PCG-V3-plasma-9_Cluster_614_sequences=11	11	19
PCG-V3-plasma-9_Cluster_932_sequences=11	11	19
PCG-V3-plasma-9_Cluster_5451_sequences=11	11	19
PCG-V3-plasma-9_Cluster_2415_sequences=11	11	19
PCG-V3-plasma-9_Cluster_3684_sequences=11	11	19
PCG-V3-plasma-9_Cluster_4241_sequences=11	11	19
PCG-V3-plasma-9_Cluster_2186_sequences=11	11	19
PCG-V3-plasma-9_Cluster_3162_sequences=11	11	19
PCG-V3-plasma-9_Cluster_814_sequences=11	11	19
PCG-V3-plasma-9_Cluster_577_sequences=11	11	19
PCG-V3-plasma-9_Cluster_1277_sequences=11	11	19
PCG-V3-plasma-9_Cluster_1512_sequences=11	11	19
PCG-V3-plasma-9_Cluster_1629_sequences=11	11	19
PCG-V3-plasma-9_Cluster_621_sequences=11	11	19
PCG-V3-plasma-9_Cluster_386_sequences=11	11	19
PCG-V3-plasma-9_Cluster_874_sequences=11	11	19
PCG-V3-plasma-9_Cluster_2670_sequences=11	11	19
PCG-V3-plasma-9_Cluster_41_sequences=11	11	19
PCG-V3-plasma-9_Cluster_680_sequences=11	11	19
PCG-V3-plasma-9_Cluster_1387_sequences=11	11	19
PCG-V3-plasma-9_Cluster_2306_sequences=11	11	19
PCG-V3-plasma-9_Cluster_385_sequences=11	11	19
PCG-V3-plasma-9_Cluster_552_sequences=11	11	19
PCG-V3-plasma-9_Cluster_6952_sequences=11	11	19
PCG-V3-plasma-9_Cluster_991_sequences=11	11	19
PCG-V3-plasma-9_Cluster_10493_sequences=11	11	19
PCG-V3-plasma-55_Cluster_106_sequences=11	11	294
PCG-V3-plasma-55_Cluster_1220_sequences=11	11	294
PCG-V3-plasma-55_Cluster_130_sequences=11	11	294

PCG-V3-plasma-55_Cluster_1602_sequences=11	11	294
PCG-V3-plasma-55_Cluster_232_sequences=11	11	294
PCG-V3-plasma-55_Cluster_381_sequences=11	11	294
PCG-V3-plasma-55_Cluster_437_sequences=11	11	294
PCG-V3-plasma-55_Cluster_674_sequences=11	11	294
PCG-V3-plasma-55_Cluster_870_sequences=11	11	294
PCG-V3-plasma-55_Cluster_893_sequences=11	11	294
PCG-V3-plasma-55_Cluster_899_sequences=11	11	294
PCG-V3-plasma-55_Cluster_729_sequences=11	11	294
PCG-V3-plasma-55_Cluster_206_sequences=11	11	294
PCG-V3-plasma-55_Cluster_782_sequences=11	11	294
PCG-V3-plasma-55_Cluster_1083_sequences=11	11	294
PCG-V3-plasma-55_Cluster_1269_sequences=11	11	294
PCG-V3-plasma-55_Cluster_126_sequences=11	11	294
PCG-V3-plasma-55_Cluster_1412_sequences=11	11	294
PCG-V3-plasma-55_Cluster_2492_sequences=11	11	294
PCG-V3-plasma-55_Cluster_251_sequences=11	11	294
PCG-V3-plasma-55_Cluster_25_sequences=11	11	294
PCG-V3-plasma-55_Cluster_743_sequences=11	11	294
PCG-V3-plasma-55_Cluster_410_sequences=11	11	294
PCG-V3-plasma-55_Cluster_630_sequences=11	11	294
PCG-V3-plasma-55_Cluster_749_sequences=11	11	294
PCG-V3-plasma-55_Cluster_1006_sequences=11	11	294
PCG-V3-PBMC-50_Cluster_1950_sequences=10	10	282
PCG-V3-PBMC-50_Cluster_323_sequences=10	10	282
PCG-V3-PBMC-50_Cluster_4957_sequences=10	10	282
PCG-V3-PBMC-50_Cluster_266_sequences=10	10	282
PCG-V3-PBMC-50_Cluster_2566_sequences=10	10	282
PCG-V3-PBMC-50_Cluster_5014_sequences=10	10	282
PCG-V3-PBMC-50_Cluster_7444_sequences=10	10	282
PCG-V3-PBMC-50_Cluster_1367_sequences=10	10	282
PCG-V3-PBMC-50_Cluster_1519_sequences=10	10	282
PCG-V3-PBMC-50_Cluster_1604_sequences=10	10	282
PCG-V3-PBMC-50_Cluster_1824_sequences=10	10	282
PCG-V3-PBMC-50_Cluster_208_sequences=10	10	282
PCG-V3-PBMC-50_Cluster_2166_sequences=10	10	282
PCG-V3-PBMC-50_Cluster_2341_sequences=10	10	282
PCG-V3-PBMC-50_Cluster_2481_sequences=10	10	282
PCG-V3-PBMC-50_Cluster_2592_sequences=10	10	282
PCG-V3-PBMC-50_Cluster_283_sequences=10	10	282
PCG-V3-PBMC-50_Cluster_327_sequences=10	10	282
PCG-V3-PBMC-50_Cluster_386_sequences=10	10	282
PCG-V3-PBMC-50_Cluster_4108_sequences=10	10	282
PCG-V3-PBMC-50_Cluster_412_sequences=10	10	282
PCG-V3-PBMC-50_Cluster_564_sequences=10	10	282

PCG-V3-PBMC-50_Cluster_6770_sequences=10	10	282
PCG-V3-PBMC-50_Cluster_1720_sequences=10	10	282
PCG-V3-PBMC-50_Cluster_2463_sequences=10	10	282
PCG-V3-PBMC-50_Cluster_5236_sequences=10	10	282
PCG-V3-plasma-9_Cluster_1004_sequences=10	10	19
PCG-V3-plasma-9_Cluster_1223_sequences=10	10	19
PCG-V3-plasma-9_Cluster_1817_sequences=10	10	19
PCG-V3-plasma-9_Cluster_1870_sequences=10	10	19
PCG-V3-plasma-9_Cluster_2695_sequences=10	10	19
PCG-V3-plasma-9_Cluster_290_sequences=10	10	19
PCG-V3-plasma-9_Cluster_365_sequences=10	10	19
PCG-V3-plasma-9_Cluster_428_sequences=10	10	19
PCG-V3-plasma-9_Cluster_604_sequences=10	10	19
PCG-V3-plasma-9_Cluster_844_sequences=10	10	19
PCG-V3-plasma-9_Cluster_845_sequences=10	10	19
PCG-V3-plasma-9_Cluster_4088_sequences=10	10	19
PCG-V3-plasma-9_Cluster_3277_sequences=10	10	19
PCG-V3-plasma-9_Cluster_2393_sequences=10	10	19
PCG-V3-plasma-9_Cluster_56_sequences=10	10	19
PCG-V3-plasma-9_Cluster_582_sequences=10	10	19
PCG-V3-plasma-9_Cluster_792_sequences=10	10	19
PCG-V3-plasma-9_Cluster_229_sequences=10	10	19
PCG-V3-plasma-9_Cluster_438_sequences=10	10	19
PCG-V3-plasma-9_Cluster_1356_sequences=10	10	19
PCG-V3-plasma-9_Cluster_1563_sequences=10	10	19
PCG-V3-plasma-9_Cluster_2359_sequences=10	10	19
PCG-V3-plasma-9_Cluster_265_sequences=10	10	19
PCG-V3-plasma-9_Cluster_3824_sequences=10	10	19
PCG-V3-plasma-9_Cluster_482_sequences=10	10	19
PCG-V3-plasma-9_Cluster_636_sequences=10	10	19
PCG-V3-plasma-9_Cluster_865_sequences=10	10	19
PCG-V3-plasma-9_Cluster_950_sequences=10	10	19
PCG-V3-plasma-9_Cluster_202_sequences=10	10	19
PCG-V3-plasma-9_Cluster_797_sequences=10	10	19
PCG-V3-plasma-9_Cluster_2001_sequences=10	10	19
PCG-V3-plasma-9_Cluster_926_sequences=10	10	19
PCG-V3-plasma-9_Cluster_1364_sequences=10	10	19
PCG-V3-plasma-9_Cluster_930_sequences=10	10	19
PCG-V3-plasma-9_Cluster_1266_sequences=10	10	19
PCG-V3-plasma-9_Cluster_1547_sequences=10	10	19
PCG-V3-plasma-9_Cluster_1969_sequences=10	10	19
PCG-V3-plasma-9_Cluster_217_sequences=10	10	19
PCG-V3-plasma-9_Cluster_3713_sequences=10	10	19
PCG-V3-plasma-9_Cluster_802_sequences=10	10	19
PCG-V3-plasma-9_Cluster_1862_sequences=10	10	19

PCG-V3-plasma-9_Cluster_665_sequences=10	10	19
PCG-V3-plasma-9_Cluster_720_sequences=10	10	19
PCG-V3-plasma-55_Cluster_1415_sequences=10	10	294
PCG-V3-plasma-55_Cluster_157_sequences=10	10	294
PCG-V3-plasma-55_Cluster_182_sequences=10	10	294
PCG-V3-plasma-55_Cluster_236_sequences=10	10	294
PCG-V3-plasma-55_Cluster_2684_sequences=10	10	294
PCG-V3-plasma-55_Cluster_30_sequences=10	10	294
PCG-V3-plasma-55_Cluster_333_sequences=10	10	294
PCG-V3-plasma-55_Cluster_36_sequences=10	10	294
PCG-V3-plasma-55_Cluster_44_sequences=10	10	294
PCG-V3-plasma-55_Cluster_473_sequences=10	10	294
PCG-V3-plasma-55_Cluster_477_sequences=10	10	294
PCG-V3-plasma-55_Cluster_491_sequences=10	10	294
PCG-V3-plasma-55_Cluster_533_sequences=10	10	294
PCG-V3-plasma-55_Cluster_569_sequences=10	10	294
PCG-V3-plasma-55_Cluster_599_sequences=10	10	294
PCG-V3-plasma-55_Cluster_653_sequences=10	10	294
PCG-V3-plasma-55_Cluster_68_sequences=10	10	294
PCG-V3-plasma-55_Cluster_739_sequences=10	10	294
PCG-V3-plasma-55_Cluster_8_sequences=10	10	294
PCG-V3-plasma-55_Cluster_329_sequences=10	10	294
PCG-V3-plasma-55_Cluster_1688_sequences=10	10	294
PCG-V3-plasma-55_Cluster_490_sequences=10	10	294
PCG-V3-plasma-55_Cluster_640_sequences=10	10	294
PCG-V3-plasma-55_Cluster_617_sequences=10	10	294
PCG-V3-plasma-55_Cluster_52_sequences=10	10	294
PCG-V3-plasma-55_Cluster_218_sequences=10	10	294
PCG-V3-plasma-55_Cluster_156_sequences=10	10	294
PCG-V3-plasma-55_Cluster_364_sequences=10	10	294
PCG-V3-plasma-55_Cluster_353_sequences=10	10	294
PCG-V3-plasma-55_Cluster_379_sequences=10	10	294
PCG-V3-plasma-55_Cluster_701_sequences=10	10	294
PCG-V3-plasma-55_Cluster_837_sequences=10	10	294
PCG-V3-plasma-55_Cluster_GGGGTCAGC_sequence	9	294
PCG-V3-plasma-79_Cluster_CTGCGGGCG_sequence	9	492
PCG-V3-PBMC-50_Cluster_3521_sequences=9	9	282
PCG-V3-PBMC-50_Cluster_4149_sequences=9	9	282
PCG-V3-PBMC-50_Cluster_810_sequences=9	9	282
PCG-V3-PBMC-50_Cluster_6523_sequences=9	9	282
PCG-V3-PBMC-50_Cluster_1228_sequences=9	9	282
PCG-V3-PBMC-50_Cluster_7035_sequences=9	9	282
PCG-V3-PBMC-50_Cluster_505_sequences=9	9	282
PCG-V3-PBMC-50_Cluster_5103_sequences=9	9	282
PCG-V3-PBMC-50_Cluster_6221_sequences=9	9	282

PCG-V3-PBMC-50_Cluster_2746_sequences=9	9	282
PCG-V3-PBMC-50_Cluster_6027_sequences=9	9	282
PCG-V3-PBMC-50_Cluster_1254_sequences=9	9	282
PCG-V3-PBMC-50_Cluster_1591_sequences=9	9	282
PCG-V3-PBMC-50_Cluster_1787_sequences=9	9	282
PCG-V3-PBMC-50_Cluster_1899_sequences=9	9	282
PCG-V3-PBMC-50_Cluster_1919_sequences=9	9	282
PCG-V3-PBMC-50_Cluster_2773_sequences=9	9	282
PCG-V3-PBMC-50_Cluster_2946_sequences=9	9	282
PCG-V3-PBMC-50_Cluster_3863_sequences=9	9	282
PCG-V3-PBMC-50_Cluster_3911_sequences=9	9	282
PCG-V3-PBMC-50_Cluster_838_sequences=9	9	282
PCG-V3-PBMC-50_Cluster_921_sequences=9	9	282
PCG-V3-PBMC-50_Cluster_966_sequences=9	9	282
PCG-V3-PBMC-50_Cluster_397_sequences=9	9	282
PCG-V3-PBMC-50_Cluster_1792_sequences=9	9	282
PCG-V3-PBMC-50_Cluster_2189_sequences=9	9	282
PCG-V3-PBMC-50_Cluster_9674_sequences=9	9	282
PCG-V3-PBMC-50_Cluster_1336_sequences=9	9	282
PCG-V3-PBMC-50_Cluster_105_sequences=9	9	282
PCG-V3-plasma-27_Cluster_GATTCATAG_sequence	8	126
PCG-V3-plasma-55_Cluster_CAGTTTATC_sequences	8	294
PCG-V3-PBMC-50_Cluster_1714_sequences=8	8	282
PCG-V3-PBMC-50_Cluster_2075_sequences=8	8	282
PCG-V3-PBMC-50_Cluster_7747_sequences=8	8	282
PCG-V3-PBMC-50_Cluster_6310_sequences=8	8	282
PCG-V3-PBMC-50_Cluster_4468_sequences=8	8	282
PCG-V3-PBMC-50_Cluster_6316_sequences=8	8	282
PCG-V3-PBMC-50_Cluster_5648_sequences=8	8	282
PCG-V3-PBMC-50_Cluster_8203_sequences=8	8	282
PCG-V3-PBMC-50_Cluster_1004_sequences=8	8	282
PCG-V3-PBMC-50_Cluster_1110_sequences=8	8	282
PCG-V3-PBMC-50_Cluster_1194_sequences=8	8	282
PCG-V3-PBMC-50_Cluster_1435_sequences=8	8	282
PCG-V3-PBMC-50_Cluster_1605_sequences=8	8	282
PCG-V3-PBMC-50_Cluster_1744_sequences=8	8	282
PCG-V3-PBMC-50_Cluster_3025_sequences=8	8	282
PCG-V3-PBMC-50_Cluster_3241_sequences=8	8	282
PCG-V3-PBMC-50_Cluster_329_sequences=8	8	282
PCG-V3-PBMC-50_Cluster_337_sequences=8	8	282
PCG-V3-PBMC-50_Cluster_3547_sequences=8	8	282
PCG-V3-PBMC-50_Cluster_5049_sequences=8	8	282
PCG-V3-PBMC-50_Cluster_611_sequences=8	8	282
PCG-V3-PBMC-50_Cluster_650_sequences=8	8	282
PCG-V3-PBMC-50_Cluster_774_sequences=8	8	282

PCG-V3-PBMC-50_Cluster_834_sequences=8	8	282
PCG-V3-PBMC-50_Cluster_3169_sequences=8	8	282
PCG-V3-PBMC-50_Cluster_1265_sequences=8	8	282
PCG-V3-PBMC-50_Cluster_3392_sequences=8	8	282
PCG-V3-PBMC-50_Cluster_5361_sequences=8	8	282
PCG-V3-PBMC-50_Cluster_173_sequences=8	8	282
PCG-V3-PBMC-50_Cluster_3060_sequences=8	8	282
PCG-V3-PBMC-50_Cluster_1142_sequences=8	8	282
PCG-V3-PBMC-50_Cluster_10277_sequences=8	8	282
PCG-V3-PBMC-50_Cluster_1418_sequences=7	7	282
PCG-V3-PBMC-50_Cluster_3018_sequences=7	7	282
PCG-V3-PBMC-50_Cluster_3485_sequences=7	7	282
PCG-V3-PBMC-50_Cluster_3966_sequences=7	7	282
PCG-V3-PBMC-50_Cluster_6306_sequences=7	7	282
PCG-V3-PBMC-50_Cluster_7888_sequences=7	7	282
PCG-V3-PBMC-50_Cluster_6925_sequences=7	7	282
PCG-V3-PBMC-50_Cluster_431_sequences=7	7	282
PCG-V3-PBMC-50_Cluster_1296_sequences=7	7	282
PCG-V3-PBMC-50_Cluster_2685_sequences=7	7	282
PCG-V3-PBMC-50_Cluster_3382_sequences=7	7	282
PCG-V3-PBMC-50_Cluster_3867_sequences=7	7	282
PCG-V3-PBMC-50_Cluster_6972_sequences=7	7	282
PCG-V3-PBMC-50_Cluster_8752_sequences=7	7	282
PCG-V3-PBMC-50_Cluster_6235_sequences=7	7	282
PCG-V3-PBMC-50_Cluster_2144_sequences=7	7	282
PCG-V3-PBMC-50_Cluster_6299_sequences=7	7	282
PCG-V3-PBMC-50_Cluster_8505_sequences=7	7	282
PCG-V3-PBMC-50_Cluster_1300_sequences=7	7	282
PCG-V3-PBMC-50_Cluster_198_sequences=7	7	282
PCG-V3-PBMC-50_Cluster_3330_sequences=7	7	282
PCG-V3-PBMC-50_Cluster_349_sequences=7	7	282
PCG-V3-PBMC-50_Cluster_380_sequences=7	7	282
PCG-V3-PBMC-50_Cluster_4041_sequences=7	7	282
PCG-V3-PBMC-50_Cluster_4633_sequences=7	7	282
PCG-V3-PBMC-50_Cluster_522_sequences=7	7	282
PCG-V3-PBMC-50_Cluster_5309_sequences=7	7	282
PCG-V3-PBMC-50_Cluster_7074_sequences=7	7	282
PCG-V3-PBMC-50_Cluster_3627_sequences=7	7	282
PCG-V3-PBMC-50_Cluster_3091_sequences=7	7	282
PCG-V3-PBMC-50_Cluster_3118_sequences=7	7	282
PCG-V3-PBMC-50_Cluster_3979_sequences=7	7	282
PCG-V3-PBMC-50_Cluster_3196_sequences=7	7	282
PCG-V3-PBMC-50_Cluster_1203_sequences=7	7	282
PCG-V3-PBMC-50_Cluster_3039_sequences=7	7	282
PCG-V3-PBMC-50_Cluster_4158_sequences=7	7	282

PCG-V3-PBMC-50_Cluster_6757_sequences=7	7	282
PCG-V3-PBMC-50_Cluster_3834_sequences=7	7	282
PCG-V3-plasma-27_Cluster_CTGTTTAAA_sequences	6	126
PCG-V3-plasma-79_Cluster_CCGAGGCCG_sequence	6	492
PCG-V3-plasma-92_Cluster_GGGAGCGTT_sequenc	6	625
PCG-V3-PBMC-50_Cluster_1245_sequences=6	6	282
PCG-V3-PBMC-50_Cluster_1315_sequences=6	6	282
PCG-V3-PBMC-50_Cluster_1273_sequences=6	6	282
PCG-V3-PBMC-50_Cluster_2543_sequences=6	6	282
PCG-V3-PBMC-50_Cluster_6086_sequences=6	6	282
PCG-V3-PBMC-50_Cluster_6656_sequences=6	6	282
PCG-V3-PBMC-50_Cluster_6628_sequences=6	6	282
PCG-V3-PBMC-50_Cluster_7963_sequences=6	6	282
PCG-V3-PBMC-50_Cluster_6563_sequences=6	6	282
PCG-V3-PBMC-50_Cluster_2232_sequences=6	6	282
PCG-V3-PBMC-50_Cluster_7005_sequences=6	6	282
PCG-V3-PBMC-50_Cluster_108_sequences=6	6	282
PCG-V3-PBMC-50_Cluster_1195_sequences=6	6	282
PCG-V3-PBMC-50_Cluster_1347_sequences=6	6	282
PCG-V3-PBMC-50_Cluster_1897_sequences=6	6	282
PCG-V3-PBMC-50_Cluster_218_sequences=6	6	282
PCG-V3-PBMC-50_Cluster_2863_sequences=6	6	282
PCG-V3-PBMC-50_Cluster_3267_sequences=6	6	282
PCG-V3-PBMC-50_Cluster_3512_sequences=6	6	282
PCG-V3-PBMC-50_Cluster_3955_sequences=6	6	282
PCG-V3-PBMC-50_Cluster_4393_sequences=6	6	282
PCG-V3-PBMC-50_Cluster_4730_sequences=6	6	282
PCG-V3-PBMC-50_Cluster_575_sequences=6	6	282
PCG-V3-PBMC-50_Cluster_5772_sequences=6	6	282
PCG-V3-PBMC-50_Cluster_602_sequences=6	6	282
PCG-V3-PBMC-50_Cluster_858_sequences=6	6	282
PCG-V3-PBMC-50_Cluster_873_sequences=6	6	282
PCG-V3-PBMC-50_Cluster_5601_sequences=6	6	282
PCG-V3-PBMC-50_Cluster_2482_sequences=6	6	282
PCG-V3-PBMC-50_Cluster_547_sequences=6	6	282
PCG-V3-PBMC-50_Cluster_1645_sequences=6	6	282
PCG-V3-PBMC-50_Cluster_3980_sequences=6	6	282
PCG-V3-PBMC-50_Cluster_970_sequences=6	6	282
PCG-V3-PBMC-50_Cluster_2752_sequences=6	6	282
PCG-V3-PBMC-50_Cluster_2829_sequences=6	6	282
PCG-V3-PBMC-50_Cluster_1229_sequences=6	6	282
PCG-V3-PBMC-50_Cluster_1557_sequences=6	6	282
PCG-V3-PBMC-50_Cluster_1846_sequences=6	6	282
PCG-V3-PBMC-50_Cluster_3186_sequences=6	6	282
PCG-V3-PBMC-50_Cluster_4193_sequences=6	6	282

PCG-V3-PBMC-50_Cluster_817_sequences=6	6	282
PCG-V3-PBMC-50_Cluster_1771_sequences=6	6	282
PCG-V3-PBMC-50_Cluster_2731_sequences=6	6	282
PCG-V3-plasma-27_Cluster_GTCATTAAC_sequences	5	126
PCG-V3-plasma-55_Cluster_AATCGTTTC_sequences	5	294
PCG-V3-plasma-55_Cluster_AGAGCCCGT_sequence	5	294
PCG-V3-plasma-55_Cluster_ATGTTTCTC_sequences	5	294
PCG-V3-plasma-55_Cluster_CATTAGTAG_sequence	5	294
PCG-V3-plasma-55_Cluster_CGACTCACT_sequences	5	294
PCG-V3-plasma-55_Cluster_CTATCTGGC_sequences	5	294
PCG-V3-plasma-55_Cluster_GCAACGGGT_sequence	5	294
PCG-V3-plasma-55_Cluster_TAAGAGCTA_sequence	5	294
PCG-V3-plasma-55_Cluster_TGAAGGTGT_sequence	5	294
PCG-V3-plasma-55_Cluster_TGCTCTCGG_sequence	5	294
PCG-V3-plasma-55_Cluster_TTGTTAGTT_sequences	5	294
PCG-V3-plasma-79_Cluster_AGATAGAGG_sequenc	5	492
PCG-V3-plasma-79_Cluster_CTGAAGTTA_sequence	5	492
PCG-V3-plasma-79_Cluster_CTGTTGAAA_sequence	5	492
PCG-V3-plasma-79_Cluster_GATGTATAG_sequence	5	492
PCG-V3-plasma-79_Cluster_TAGGCACTC_sequence	5	492
PCG-V3-plasma-92_Cluster_GGCATGCCG_sequence	5	625
PCG-V3-PBMC-50_Cluster_3088_sequences=5	5	282
PCG-V3-PBMC-50_Cluster_3640_sequences=5	5	282
PCG-V3-PBMC-50_Cluster_7379_sequences=5	5	282
PCG-V3-PBMC-50_Cluster_7519_sequences=5	5	282
PCG-V3-PBMC-50_Cluster_8961_sequences=5	5	282
PCG-V3-PBMC-50_Cluster_9434_sequences=5	5	282
PCG-V3-PBMC-50_Cluster_1152_sequences=5	5	282
PCG-V3-PBMC-50_Cluster_4933_sequences=5	5	282
PCG-V3-PBMC-50_Cluster_2015_sequences=5	5	282
PCG-V3-PBMC-50_Cluster_5663_sequences=5	5	282
PCG-V3-PBMC-50_Cluster_6554_sequences=5	5	282
PCG-V3-PBMC-50_Cluster_5416_sequences=5	5	282
PCG-V3-PBMC-50_Cluster_1939_sequences=5	5	282
PCG-V3-PBMC-50_Cluster_2883_sequences=5	5	282
PCG-V3-PBMC-50_Cluster_3193_sequences=5	5	282
PCG-V3-PBMC-50_Cluster_5903_sequences=5	5	282
PCG-V3-PBMC-50_Cluster_1493_sequences=5	5	282
PCG-V3-PBMC-50_Cluster_1586_sequences=5	5	282
PCG-V3-PBMC-50_Cluster_1702_sequences=5	5	282
PCG-V3-PBMC-50_Cluster_1914_sequences=5	5	282
PCG-V3-PBMC-50_Cluster_1934_sequences=5	5	282
PCG-V3-PBMC-50_Cluster_1948_sequences=5	5	282
PCG-V3-PBMC-50_Cluster_2270_sequences=5	5	282
PCG-V3-PBMC-50_Cluster_2328_sequences=5	5	282

PCG-V3-PBMC-50_Cluster_2451_sequences=5	5	282
PCG-V3-PBMC-50_Cluster_24_sequences=5	5	282
PCG-V3-PBMC-50_Cluster_3007_sequences=5	5	282
PCG-V3-PBMC-50_Cluster_3142_sequences=5	5	282
PCG-V3-PBMC-50_Cluster_3176_sequences=5	5	282
PCG-V3-PBMC-50_Cluster_3219_sequences=5	5	282
PCG-V3-PBMC-50_Cluster_3299_sequences=5	5	282
PCG-V3-PBMC-50_Cluster_3841_sequences=5	5	282
PCG-V3-PBMC-50_Cluster_4004_sequences=5	5	282
PCG-V3-PBMC-50_Cluster_4233_sequences=5	5	282
PCG-V3-PBMC-50_Cluster_5047_sequences=5	5	282
PCG-V3-PBMC-50_Cluster_5338_sequences=5	5	282
PCG-V3-PBMC-50_Cluster_814_sequences=5	5	282
PCG-V3-PBMC-50_Cluster_8367_sequences=5	5	282
PCG-V3-PBMC-50_Cluster_884_sequences=5	5	282
PCG-V3-PBMC-50_Cluster_960_sequences=5	5	282
PCG-V3-PBMC-50_Cluster_3706_sequences=5	5	282
PCG-V3-PBMC-50_Cluster_2969_sequences=5	5	282
PCG-V3-PBMC-50_Cluster_404_sequences=5	5	282
PCG-V3-PBMC-50_Cluster_5927_sequences=5	5	282
PCG-V3-PBMC-50_Cluster_8962_sequences=5	5	282
PCG-V3-PBMC-50_Cluster_5691_sequences=5	5	282
PCG-V3-PBMC-50_Cluster_144_sequences=5	5	282
PCG-V3-PBMC-50_Cluster_154_sequences=5	5	282
PCG-V3-PBMC-50_Cluster_2371_sequences=5	5	282
PCG-V3-PBMC-50_Cluster_265_sequences=5	5	282
PCG-V3-PBMC-50_Cluster_3044_sequences=5	5	282
PCG-V3-PBMC-50_Cluster_3217_sequences=5	5	282
PCG-V3-PBMC-50_Cluster_6237_sequences=5	5	282
PCG-V3-PBMC-50_Cluster_5020_sequences=5	5	282
PCG-V3-PBMC-50_Cluster_1235_sequences=5	5	282
PCG-V3-PBMC-50_Cluster_715_sequences=5	5	282
PCG-V3-PBMC-50_Cluster_1710_sequences=5	5	282

Patient SDS4

haplotype	number of reads	timepoint
SDS-V3-plasma-45_Cluster_34_sequences=78449	282	78449
SDS-V3-plasma-5_Cluster_1_sequences=45662	9	45662
SDS-V3-plasma-67_Cluster_39_sequences=41660	504	41660
SDS-V3-plasma-46_Cluster_2_sequences=37316	286	37316
SDS-V3-plasma-45_Cluster_113_sequences=36763	282	36763
SDS-V3-plasma-67_Cluster_107_sequences=34801	504	34801
SDS-V3-plasma-45_Cluster_44_sequences=32025	282	32025
SDS-V3-plasma-46_Cluster_7_sequences=31887	286	31887
SDS-V3-plasma-67_Cluster_43_sequences=30418	504	30418
SDS-V3-plasma-45_Cluster_28_sequences=29782	282	29782
SDS-V3-plasma-67_Cluster_76_sequences=27896	504	27896
SDS-V3-plasma-67_Cluster_7_sequences=26488	504	26488
SDS-V3-plasma-7_Cluster_0_sequences=24721	14	24721
SDS-V3-plasma-45_Cluster_50_sequences=22019	282	22019
SDS-V3-plasma-67_Cluster_69_sequences=20366	504	20366
SDS-V3-plasma-67_Cluster_194_sequences=20111	504	20111
SDS-V3-plasma-45_Cluster_86_sequences=18666	282	18666
SDS-V3-plasma-7_Cluster_6_sequences=18184	14	18184
SDS-V3-plasma-67_Cluster_18_sequences=18154	504	18154
SDS-V3-plasma-8_Cluster_2264_sequences=18049	16	18049
SDS-V3-plasma-67_Cluster_146_sequences=17898	504	17898
SDS-V3-plasma-46_Cluster_10_sequences=17447	286	17447
SDS-V3-plasma-0_Cluster_44_sequences=15624	0	15624
SDS-V3-plasma-0_Cluster_54_sequences=15268	0	15268
SDS-V3-plasma-45_Cluster_5_sequences=14641	282	14641
SDS-V3-plasma-45_Cluster_48_sequences=14077	282	14077
SDS-V3-plasma-7_Cluster_2_sequences=14076	14	14076
SDS-V3-plasma-45_Cluster_88_sequences=12662	282	12662
SDS-V3-plasma-0_Cluster_25_sequences=11702	0	11702
SDS-V3-plasma-0_Cluster_10_sequences=11466	0	11466
SDS-V3-plasma-0_Cluster_137_sequences=11072	0	11072
SDS-V3-plasma-8_Cluster_7_sequences=10823	16	10823
SDS-V3-plasma-0_Cluster_81_sequences=10715	0	10715
SDS-V3-plasma-46_Cluster_74_sequences=10409	286	10409
SDS-V3-plasma-45_Cluster_277_sequences=9980	282	9980
SDS-V3-plasma-0_Cluster_105_sequences=9420	0	9420
SDS-V3-plasma-45_Cluster_53_sequences=9156	282	9156
SDS-V3-plasma-67_Cluster_419_sequences=9154	504	9154
SDS-V3-plasma-45_Cluster_40_sequences=9046	282	9046
SDS-V3-plasma-46_Cluster_55_sequences=8840	286	8840
SDS-V3-plasma-45_Cluster_84_sequences=8820	282	8820
SDS-V3-plasma-8_Cluster_2268_sequences=8731	16	8731
SDS-V3-plasma-0_Cluster_128_sequences=8673	0	8673

SDS-V3-plasma-45_Cluster_279_sequences=8642	282	8642
SDS-V3-plasma-45_Cluster_58_sequences=8167	282	8167
SDS-V3-plasma-46_Cluster_25_sequences=8097	286	8097
SDS-V3-plasma-67_Cluster_366_sequences=7992	504	7992
SDS-V3-plasma-45_Cluster_42_sequences=7256	282	7256
SDS-V3-plasma-46_Cluster_9_sequences=7162	286	7162
SDS-V3-plasma-8_Cluster_2259_sequences=6994	16	6994
SDS-V3-plasma-46_Cluster_48_sequences=6883	286	6883
SDS-V3-plasma-45_Cluster_56_sequences=6521	282	6521
SDS-V3-plasma-45_Cluster_259_sequences=6197	282	6197
SDS-V3-plasma-46_Cluster_16_sequences=6170	286	6170
SDS-V3-plasma-45_Cluster_282_sequences=5967	282	5967
SDS-V3-plasma-45_Cluster_57_sequences=5800	282	5800
SDS-V3-plasma-45_Cluster_102_sequences=5722	282	5722
SDS-V3-plasma-46_Cluster_24_sequences=5381	286	5381
SDS-V3-plasma-45_Cluster_139_sequences=5124	282	5124
SDS-V3-plasma-67_Cluster_143_sequences=5098	504	5098
SDS-V3-plasma-45_Cluster_85_sequences=4985	282	4985
SDS-V3-plasma-45_Cluster_225_sequences=4905	282	4905
SDS-V3-plasma-7_Cluster_7_sequences=4873	14	4873
SDS-V3-plasma-45_Cluster_87_sequences=4800	282	4800
SDS-V3-plasma-67_Cluster_131_sequences=4523	504	4523
SDS-V3-plasma-7_Cluster_39_sequences=4519	14	4519
SDS-V3-plasma-67_Cluster_384_sequences=4472	504	4472
SDS-V3-plasma-67_Cluster_66_sequences=4307	504	4307
SDS-V3-plasma-45_Cluster_513_sequences=4116	282	4116
SDS-V3-plasma-45_Cluster_247_sequences=4001	282	4001
SDS-V3-plasma-67_Cluster_77_sequences=3965	504	3965
SDS-V3-plasma-45_Cluster_188_sequences=3956	282	3956
SDS-V3-plasma-45_Cluster_105_sequences=3923	282	3923
SDS-V3-plasma-45_Cluster_31_sequences=3910	282	3910
SDS-V3-plasma-67_Cluster_88_sequences=3812	504	3812
SDS-V3-plasma-67_Cluster_124_sequences=3733	504	3733
SDS-V3-plasma-46_Cluster_23_sequences=3646	286	3646
SDS-V3-plasma-45_Cluster_240_sequences=3576	282	3576
SDS-V3-plasma-46_Cluster_5_sequences=3494	286	3494
SDS-V3-plasma-67_Cluster_102_sequences=3488	504	3488
SDS-V3-plasma-7_Cluster_52_sequences=3422	14	3422
SDS-V3-plasma-67_Cluster_564_sequences=3385	504	3385
SDS-V3-plasma-67_Cluster_70_sequences=3338	504	3338
SDS-V3-plasma-67_Cluster_166_sequences=3277	504	3277
SDS-V3-plasma-45_Cluster_260_sequences=3194	282	3194
SDS-V3-plasma-45_Cluster_153_sequences=3176	282	3176
SDS-V3-plasma-67_Cluster_569_sequences=3154	504	3154
SDS-V3-plasma-0_Cluster_118_sequences=3121	0	3121

SDS-V3-plasma-45_Cluster_315_sequences=3014	282	3014
SDS-V3-plasma-67_Cluster_136_sequences=2973	504	2973
SDS-V3-plasma-45_Cluster_404_sequences=2944	282	2944
SDS-V3-plasma-67_Cluster_175_sequences=2839	504	2839
SDS-V3-plasma-67_Cluster_518_sequences=2807	504	2807
SDS-V3-plasma-67_Cluster_262_sequences=2804	504	2804
SDS-V3-plasma-67_Cluster_463_sequences=2780	504	2780
SDS-V3-plasma-67_Cluster_296_sequences=2728	504	2728
SDS-V3-plasma-67_Cluster_170_sequences=2697	504	2697
SDS-V3-plasma-67_Cluster_378_sequences=2625	504	2625
SDS-V3-plasma-0_Cluster_172_sequences=2604	0	2604
SDS-V3-plasma-46_Cluster_65_sequences=2553	286	2553
SDS-V3-plasma-46_Cluster_135_sequences=2533	286	2533
SDS-V3-plasma-67_Cluster_319_sequences=2489	504	2489
SDS-V3-plasma-45_Cluster_429_sequences=2476	282	2476
SDS-V3-plasma-45_Cluster_162_sequences=2460	282	2460
SDS-V3-plasma-45_Cluster_388_sequences=2447	282	2447
SDS-V3-plasma-45_Cluster_209_sequences=2395	282	2395
SDS-V3-plasma-27_Cluster_37_sequences=2289	131	2289
SDS-V3-plasma-0_Cluster_116_sequences=2277	0	2277
SDS-V3-plasma-0_Cluster_72_sequences=2154	0	2154
SDS-V3-plasma-46_Cluster_230_sequences=2142	286	2142
SDS-V3-plasma-45_Cluster_460_sequences=2128	282	2128
SDS-V3-plasma-0_Cluster_84_sequences=2120	0	2120
SDS-V3-plasma-0_Cluster_58_sequences=2108	0	2108
SDS-V3-plasma-67_Cluster_329_sequences=2096	504	2096
SDS-V3-plasma-67_Cluster_276_sequences=2092	504	2092
SDS-V3-plasma-0_Cluster_38_sequences=2073	0	2073
SDS-V3-plasma-46_Cluster_104_sequences=2072	286	2072
SDS-V3-plasma-45_Cluster_258_sequences=2070	282	2070
SDS-V3-plasma-67_Cluster_548_sequences=2055	504	2055
SDS-V3-plasma-45_Cluster_163_sequences=2028	282	2028
SDS-V3-plasma-67_Cluster_800_sequences=1999	504	1999
SDS-V3-plasma-45_Cluster_167_sequences=1996	282	1996
SDS-V3-plasma-24_Cluster_137_sequences=1992	124	1992
SDS-V3-plasma-7_Cluster_41_sequences=1987	14	1987
SDS-V3-plasma-67_Cluster_192_sequences=1977	504	1977
SDS-V3-plasma-46_Cluster_200_sequences=1972	286	1972
SDS-V3-plasma-67_Cluster_432_sequences=1917	504	1917
SDS-V3-plasma-45_Cluster_366_sequences=1905	282	1905
SDS-V3-plasma-46_Cluster_197_sequences=1892	286	1892
SDS-V3-plasma-46_Cluster_233_sequences=1852	286	1852
SDS-V3-plasma-0_Cluster_190_sequences=1848	0	1848
SDS-V3-plasma-46_Cluster_269_sequences=1833	286	1833
SDS-V3-plasma-46_Cluster_115_sequences=1821	286	1821

SDS-V3-PBMC-45_Cluster_15036_sequences=1785	282	1785
SDS-V3-plasma-8_Cluster_2260_sequences=1782	16	1782
SDS-V3-plasma-45_Cluster_400_sequences=1772	282	1772
SDS-V3-plasma-45_Cluster_253_sequences=1748	282	1748
SDS-V3-plasma-45_Cluster_417_sequences=1722	282	1722
SDS-V3-plasma-67_Cluster_269_sequences=1702	504	1702
SDS-V3-plasma-46_Cluster_67_sequences=1701	286	1701
SDS-V3-plasma-46_Cluster_185_sequences=1688	286	1688
SDS-V3-plasma-45_Cluster_411_sequences=1678	282	1678
SDS-V3-plasma-45_Cluster_266_sequences=1661	282	1661
SDS-V3-plasma-45_Cluster_576_sequences=1622	282	1622
SDS-V3-plasma-46_Cluster_151_sequences=1615	286	1615
SDS-V3-plasma-45_Cluster_515_sequences=1608	282	1608
SDS-V3-plasma-67_Cluster_36_sequences=1602	504	1602
SDS-V3-plasma-67_Cluster_119_sequences=1579	504	1579
SDS-V3-plasma-7_Cluster_66_sequences=1578	14	1578
SDS-V3-plasma-46_Cluster_122_sequences=1569	286	1569
SDS-V3-plasma-45_Cluster_345_sequences=1558	282	1558
SDS-V3-plasma-8_Cluster_2296_sequences=1535	16	1535
SDS-V3-plasma-45_Cluster_195_sequences=1535	282	1535
SDS-V3-plasma-0_Cluster_130_sequences=1534	0	1534
SDS-V3-plasma-46_Cluster_132_sequences=1436	286	1436
SDS-V3-plasma-45_Cluster_512_sequences=1425	282	1425
SDS-V3-plasma-67_Cluster_189_sequences=1415	504	1415
SDS-V3-plasma-46_Cluster_42_sequences=1391	286	1391
SDS-V3-plasma-8_Cluster_2325_sequences=1368	16	1368
SDS-V3-plasma-67_Cluster_213_sequences=1368	504	1368
SDS-V3-plasma-7_Cluster_44_sequences=1347	14	1347
SDS-V3-plasma-67_Cluster_60_sequences=1344	504	1344
SDS-V3-plasma-45_Cluster_381_sequences=1335	282	1335
SDS-V3-plasma-45_Cluster_441_sequences=1327	282	1327
SDS-V3-plasma-0_Cluster_135_sequences=1312	0	1312
SDS-V3-plasma-67_Cluster_581_sequences=1279	504	1279
SDS-V3-plasma-0_Cluster_126_sequences=1268	0	1268
SDS-V3-plasma-45_Cluster_493_sequences=1261	282	1261
SDS-V3-plasma-0_Cluster_74_sequences=1246	0	1246
SDS-V3-plasma-45_Cluster_245_sequences=1239	282	1239
SDS-V3-plasma-46_Cluster_174_sequences=1239	286	1239
SDS-V3-plasma-45_Cluster_179_sequences=1204	282	1204
SDS-V3-plasma-24_Cluster_73_sequences=1200	124	1200
SDS-V3-plasma-0_Cluster_104_sequences=1193	0	1193
SDS-V3-plasma-45_Cluster_36_sequences=1172	282	1172
SDS-V3-plasma-45_Cluster_149_sequences=1170	282	1170
SDS-V3-plasma-7_Cluster_43_sequences=1169	14	1169
SDS-V3-plasma-67_Cluster_1475_sequences=1165	504	1165

SDS-V3-plasma-67_Cluster_313_sequences=1161	504	1161
SDS-V3-plasma-67_Cluster_48_sequences=1157	504	1157
SDS-V3-plasma-46_Cluster_109_sequences=1154	286	1154
SDS-V3-plasma-67_Cluster_451_sequences=1154	504	1154
SDS-V3-plasma-45_Cluster_979_sequences=1144	282	1144
SDS-V3-plasma-45_Cluster_481_sequences=1144	282	1144
SDS-V3-plasma-8_Cluster_2283_sequences=1132	16	1132
SDS-V3-plasma-67_Cluster_144_sequences=1112	504	1112
SDS-V3-plasma-67_Cluster_1015_sequences=1109	504	1109
SDS-V3-plasma-46_Cluster_207_sequences=1097	286	1097
SDS-V3-plasma-45_Cluster_98_sequences=1091	282	1091
SDS-V3-plasma-45_Cluster_613_sequences=1090	282	1090
SDS-V3-plasma-45_Cluster_181_sequences=1078	282	1078
SDS-V3-plasma-0_Cluster_573_sequences=1047	0	1047
SDS-V3-plasma-8_Cluster_2309_sequences=1040	16	1040
SDS-V3-plasma-67_Cluster_397_sequences=1038	504	1038
SDS-V3-plasma-45_Cluster_273_sequences=1036	282	1036
SDS-V3-plasma-7_Cluster_46_sequences=1034	14	1034
SDS-V3-plasma-45_Cluster_90_sequences=1034	282	1034
SDS-V3-plasma-67_Cluster_154_sequences=1033	504	1033
SDS-V3-plasma-0_Cluster_167_sequences=1025	0	1025
SDS-V3-plasma-67_Cluster_159_sequences=1018	504	1018
SDS-V3-plasma-45_Cluster_403_sequences=1000	282	1000
SDS-V3-plasma-45_Cluster_376_sequences=995	282	995
SDS-V3-plasma-45_Cluster_265_sequences=986	282	986
SDS-V3-plasma-45_Cluster_249_sequences=985	282	985
SDS-V3-plasma-67_Cluster_658_sequences=971	504	971
SDS-V3-plasma-67_Cluster_84_sequences=966	504	966
SDS-V3-plasma-67_Cluster_975_sequences=955	504	955
SDS-V3-plasma-67_Cluster_89_sequences=943	504	943
SDS-V3-plasma-67_Cluster_253_sequences=939	504	939
SDS-V3-plasma-67_Cluster_618_sequences=938	504	938
SDS-V3-plasma-67_Cluster_123_sequences=927	504	927
SDS-V3-plasma-45_Cluster_126_sequences=918	282	918
SDS-V3-plasma-67_Cluster_412_sequences=908	504	908
SDS-V3-plasma-45_Cluster_115_sequences=905	282	905
SDS-V3-plasma-67_Cluster_851_sequences=898	504	898
SDS-V3-plasma-67_Cluster_1196_sequences=898	504	898
SDS-V3-plasma-0_Cluster_168_sequences=895	0	895
SDS-V3-plasma-45_Cluster_199_sequences=893	282	893
SDS-V3-plasma-67_Cluster_308_sequences=873	504	873
SDS-V3-plasma-67_Cluster_578_sequences=871	504	871
SDS-V3-plasma-67_Cluster_155_sequences=871	504	871
SDS-V3-plasma-67_Cluster_21_sequences=859	504	859
SDS-V3-plasma-8_Cluster_2318_sequences=853	16	853

SDS-V3-plasma-8_Cluster_2314_sequences=853	16	853
SDS-V3-plasma-46_Cluster_78_sequences=840	286	840
SDS-V3-plasma-67_Cluster_98_sequences=838	504	838
SDS-V3-plasma-7_Cluster_16_sequences=835	14	835
SDS-V3-plasma-46_Cluster_36_sequences=835	286	835
SDS-V3-plasma-46_Cluster_358_sequences=835	286	835
SDS-V3-plasma-0_Cluster_537_sequences=826	0	826
SDS-V3-plasma-8_Cluster_2282_sequences=825	16	825
SDS-V3-plasma-67_Cluster_352_sequences=823	504	823
SDS-V3-plasma-46_Cluster_214_sequences=821	286	821
SDS-V3-plasma-67_Cluster_361_sequences=814	504	814
SDS-V3-plasma-67_Cluster_1065_sequences=810	504	810
SDS-V3-plasma-7_Cluster_58_sequences=809	14	809
SDS-V3-plasma-67_Cluster_2256_sequences=795	504	795
SDS-V3-plasma-7_Cluster_77_sequences=786	14	786
SDS-V3-plasma-45_Cluster_175_sequences=784	282	784
SDS-V3-plasma-46_Cluster_89_sequences=784	286	784
SDS-V3-plasma-67_Cluster_211_sequences=784	504	784
SDS-V3-plasma-46_Cluster_164_sequences=782	286	782
SDS-V3-plasma-67_Cluster_848_sequences=782	504	782
SDS-V3-plasma-67_Cluster_383_sequences=778	504	778
SDS-V3-plasma-67_Cluster_544_sequences=777	504	777
SDS-V3-plasma-67_Cluster_14018_sequences=776	504	776
SDS-V3-plasma-7_Cluster_37_sequences=772	14	772
SDS-V3-plasma-7_Cluster_59_sequences=771	14	771
SDS-V3-plasma-45_Cluster_663_sequences=769	282	769
SDS-V3-plasma-45_Cluster_1233_sequences=769	282	769
SDS-V3-plasma-45_Cluster_680_sequences=763	282	763
SDS-V3-plasma-46_Cluster_368_sequences=760	286	760
SDS-V3-plasma-67_Cluster_1678_sequences=759	504	759
SDS-V3-plasma-45_Cluster_1036_sequences=753	282	753
SDS-V3-plasma-46_Cluster_47_sequences=750	286	750
SDS-V3-plasma-46_Cluster_247_sequences=745	286	745
SDS-V3-plasma-67_Cluster_501_sequences=744	504	744
SDS-V3-plasma-45_Cluster_409_sequences=743	282	743
SDS-V3-plasma-45_Cluster_437_sequences=742	282	742
SDS-V3-plasma-67_Cluster_396_sequences=742	504	742
SDS-V3-plasma-67_Cluster_1790_sequences=742	504	742
SDS-V3-plasma-67_Cluster_1974_sequences=741	504	741
SDS-V3-plasma-8_Cluster_2273_sequences=740	16	740
SDS-V3-plasma-67_Cluster_42_sequences=740	504	740
SDS-V3-plasma-46_Cluster_136_sequences=733	286	733
SDS-V3-plasma-7_Cluster_22_sequences=730	14	730
SDS-V3-plasma-46_Cluster_284_sequences=729	286	729
SDS-V3-plasma-67_Cluster_385_sequences=729	504	729

SDS-V3-plasma-46_Cluster_116_sequences=727	286	727
SDS-V3-plasma-67_Cluster_1829_sequences=723	504	723
SDS-V3-plasma-8_Cluster_2390_sequences=719	16	719
SDS-V3-plasma-46_Cluster_147_sequences=719	286	719
SDS-V3-plasma-45_Cluster_879_sequences=717	282	717
SDS-V3-plasma-67_Cluster_242_sequences=714	504	714
SDS-V3-plasma-67_Cluster_164_sequences=710	504	710
SDS-V3-plasma-45_Cluster_736_sequences=708	282	708
SDS-V3-plasma-67_Cluster_816_sequences=708	504	708
SDS-V3-plasma-67_Cluster_87_sequences=707	504	707
SDS-V3-plasma-67_Cluster_420_sequences=705	504	705
SDS-V3-plasma-46_Cluster_191_sequences=704	286	704
SDS-V3-plasma-46_Cluster_448_sequences=701	286	701
SDS-V3-plasma-67_Cluster_418_sequences=701	504	701
SDS-V3-plasma-45_Cluster_585_sequences=699	282	699
SDS-V3-plasma-67_Cluster_1239_sequences=699	504	699
SDS-V3-plasma-67_Cluster_247_sequences=697	504	697
SDS-V3-plasma-67_Cluster_834_sequences=695	504	695
SDS-V3-plasma-67_Cluster_1268_sequences=694	504	694
SDS-V3-plasma-45_Cluster_354_sequences=692	282	692
SDS-V3-plasma-45_Cluster_397_sequences=691	282	691
SDS-V3-plasma-46_Cluster_108_sequences=690	286	690
SDS-V3-plasma-45_Cluster_780_sequences=688	282	688
SDS-V3-plasma-0_Cluster_750_sequences=687	0	687
SDS-V3-plasma-67_Cluster_971_sequences=674	504	674
SDS-V3-plasma-46_Cluster_414_sequences=672	286	672
SDS-V3-plasma-67_Cluster_46_sequences=665	504	665
SDS-V3-plasma-45_Cluster_454_sequences=658	282	658
SDS-V3-plasma-45_Cluster_257_sequences=654	282	654
SDS-V3-plasma-0_Cluster_176_sequences=647	0	647
SDS-V3-plasma-67_Cluster_1213_sequences=646	504	646
SDS-V3-plasma-45_Cluster_540_sequences=643	282	643
SDS-V3-plasma-45_Cluster_424_sequences=643	282	643
SDS-V3-plasma-67_Cluster_688_sequences=642	504	642
SDS-V3-plasma-46_Cluster_94_sequences=641	286	641
SDS-V3-plasma-46_Cluster_92_sequences=640	286	640
SDS-V3-plasma-67_Cluster_648_sequences=638	504	638
SDS-V3-plasma-67_Cluster_128_sequences=636	504	636
SDS-V3-plasma-67_Cluster_163_sequences=635	504	635
SDS-V3-plasma-7_Cluster_233_sequences=634	14	634
SDS-V3-plasma-67_Cluster_862_sequences=630	504	630
SDS-V3-plasma-67_Cluster_1260_sequences=624	504	624
SDS-V3-plasma-67_Cluster_47_sequences=623	504	623
SDS-V3-plasma-67_Cluster_1264_sequences=621	504	621
SDS-V3-plasma-67_Cluster_67_sequences=619	504	619

SDS-V3-plasma-67_Cluster_139_sequences=616	504	616
SDS-V3-plasma-67_Cluster_2059_sequences=615	504	615
SDS-V3-plasma-46_Cluster_146_sequences=614	286	614
SDS-V3-plasma-67_Cluster_620_sequences=613	504	613
SDS-V3-plasma-45_Cluster_627_sequences=610	282	610
SDS-V3-plasma-46_Cluster_184_sequences=610	286	610
SDS-V3-plasma-45_Cluster_211_sequences=608	282	608
SDS-V3-plasma-45_Cluster_802_sequences=603	282	603
SDS-V3-plasma-45_Cluster_959_sequences=603	282	603
SDS-V3-plasma-67_Cluster_955_sequences=600	504	600
SDS-V3-plasma-67_Cluster_275_sequences=596	504	596
SDS-V3-plasma-67_Cluster_723_sequences=593	504	593
SDS-V3-plasma-46_Cluster_114_sequences=588	286	588
SDS-V3-plasma-67_Cluster_407_sequences=588	504	588
SDS-V3-plasma-45_Cluster_221_sequences=586	282	586
SDS-V3-plasma-24_Cluster_148_sequences=583	124	583
SDS-V3-plasma-67_Cluster_632_sequences=583	504	583
SDS-V3-plasma-46_Cluster_95_sequences=582	286	582
SDS-V3-plasma-7_Cluster_110_sequences=581	14	581
SDS-V3-plasma-67_Cluster_270_sequences=581	504	581
SDS-V3-plasma-67_Cluster_685_sequences=571	504	571
SDS-V3-plasma-27_Cluster_74_sequences=569	131	569
SDS-V3-plasma-67_Cluster_104_sequences=568	504	568
SDS-V3-plasma-67_Cluster_108_sequences=567	504	567
SDS-V3-plasma-67_Cluster_1795_sequences=567	504	567
SDS-V3-plasma-67_Cluster_215_sequences=567	504	567
SDS-V3-plasma-45_Cluster_1802_sequences=566	282	566
SDS-V3-plasma-67_Cluster_656_sequences=565	504	565
SDS-V3-plasma-45_Cluster_541_sequences=564	282	564
SDS-V3-plasma-67_Cluster_1573_sequences=564	504	564
SDS-V3-plasma-67_Cluster_613_sequences=564	504	564
SDS-V3-plasma-67_Cluster_773_sequences=562	504	562
SDS-V3-plasma-67_Cluster_721_sequences=561	504	561
SDS-V3-plasma-67_Cluster_494_sequences=557	504	557
SDS-V3-plasma-46_Cluster_35_sequences=556	286	556
SDS-V3-plasma-67_Cluster_125_sequences=555	504	555
SDS-V3-plasma-67_Cluster_1562_sequences=553	504	553
SDS-V3-plasma-45_Cluster_52_sequences=549	282	549
SDS-V3-plasma-8_Cluster_2258_sequences=548	16	548
SDS-V3-plasma-45_Cluster_894_sequences=548	282	548
SDS-V3-plasma-67_Cluster_1889_sequences=545	504	545
SDS-V3-plasma-67_Cluster_1105_sequences=544	504	544
SDS-V3-plasma-0_Cluster_288_sequences=543	0	543
SDS-V3-plasma-46_Cluster_45_sequences=543	286	543
SDS-V3-plasma-67_Cluster_639_sequences=541	504	541

SDS-V3-plasma-67_Cluster_841_sequences=540	504	540
SDS-V3-plasma-67_Cluster_678_sequences=539	504	539
SDS-V3-plasma-67_Cluster_25_sequences=539	504	539
SDS-V3-plasma-45_Cluster_1189_sequences=538	282	538
SDS-V3-plasma-67_Cluster_1176_sequences=538	504	538
SDS-V3-plasma-67_Cluster_238_sequences=538	504	538
SDS-V3-plasma-67_Cluster_679_sequences=535	504	535
SDS-V3-plasma-8_Cluster_2416_sequences=534	16	534
SDS-V3-plasma-67_Cluster_5_sequences=533	504	533
SDS-V3-plasma-67_Cluster_1151_sequences=530	504	530
SDS-V3-plasma-67_Cluster_115_sequences=527	504	527
SDS-V3-plasma-67_Cluster_81_sequences=525	504	525
SDS-V3-plasma-67_Cluster_2513_sequences=524	504	524
SDS-V3-plasma-67_Cluster_517_sequences=520	504	520
SDS-V3-plasma-67_Cluster_1062_sequences=518	504	518
SDS-V3-plasma-8_Cluster_2350_sequences=516	16	516
SDS-V3-plasma-46_Cluster_105_sequences=515	286	515
SDS-V3-plasma-67_Cluster_422_sequences=512	504	512
SDS-V3-plasma-0_Cluster_514_sequences=511	0	511
SDS-V3-plasma-67_Cluster_482_sequences=510	504	510
SDS-V3-plasma-46_Cluster_724_sequences=509	286	509
SDS-V3-plasma-67_Cluster_4217_sequences=506	504	506
SDS-V3-plasma-0_Cluster_671_sequences=505	0	505
SDS-V3-plasma-45_Cluster_438_sequences=505	282	505
SDS-V3-plasma-67_Cluster_1031_sequences=502	504	502
SDS-V3-plasma-0_Cluster_240_sequences=500	0	500
SDS-V3-plasma-67_Cluster_633_sequences=498	504	498
SDS-V3-plasma-45_Cluster_1135_sequences=496	282	496
SDS-V3-plasma-45_Cluster_328_sequences=496	282	496
SDS-V3-plasma-67_Cluster_1622_sequences=495	504	495
SDS-V3-plasma-7_Cluster_32_sequences=494	14	494
SDS-V3-plasma-45_Cluster_2386_sequences=494	282	494
SDS-V3-plasma-67_Cluster_898_sequences=494	504	494
SDS-V3-plasma-67_Cluster_117_sequences=494	504	494
SDS-V3-plasma-67_Cluster_870_sequences=488	504	488
SDS-V3-plasma-24_Cluster_117_sequences=485	124	485
SDS-V3-plasma-46_Cluster_221_sequences=485	286	485
SDS-V3-plasma-24_Cluster_95_sequences=484	124	484
SDS-V3-plasma-45_Cluster_207_sequences=484	282	484
SDS-V3-plasma-46_Cluster_84_sequences=483	286	483
SDS-V3-plasma-7_Cluster_150_sequences=481	14	481
SDS-V3-plasma-67_Cluster_374_sequences=481	504	481
SDS-V3-plasma-7_Cluster_50_sequences=480	14	480
SDS-V3-plasma-45_Cluster_352_sequences=479	282	479
SDS-V3-plasma-45_Cluster_1061_sequences=478	282	478

SDS-V3-plasma-67_Cluster_1318_sequences=478	504	478
SDS-V3-plasma-67_Cluster_280_sequences=475	504	475
SDS-V3-plasma-45_Cluster_597_sequences=473	282	473
SDS-V3-plasma-67_Cluster_865_sequences=471	504	471
SDS-V3-plasma-45_Cluster_1928_sequences=470	282	470
SDS-V3-plasma-46_Cluster_652_sequences=470	286	470
SDS-V3-plasma-46_Cluster_619_sequences=470	286	470
SDS-V3-plasma-67_Cluster_1169_sequences=468	504	468
SDS-V3-plasma-67_Cluster_1345_sequences=463	504	463
SDS-V3-plasma-45_Cluster_763_sequences=462	282	462
SDS-V3-plasma-67_Cluster_1313_sequences=462	504	462
SDS-V3-plasma-7_Cluster_72_sequences=461	14	461
SDS-V3-plasma-45_Cluster_581_sequences=461	282	461
SDS-V3-plasma-67_Cluster_686_sequences=457	504	457
SDS-V3-plasma-67_Cluster_2300_sequences=457	504	457
SDS-V3-plasma-67_Cluster_589_sequences=456	504	456
SDS-V3-plasma-46_Cluster_518_sequences=455	286	455
SDS-V3-plasma-67_Cluster_896_sequences=455	504	455
SDS-V3-plasma-67_Cluster_455_sequences=454	504	454
SDS-V3-plasma-45_Cluster_503_sequences=453	282	453
SDS-V3-plasma-67_Cluster_1027_sequences=453	504	453
SDS-V3-plasma-67_Cluster_1049_sequences=452	504	452
SDS-V3-plasma-27_Cluster_342_sequences=451	131	451
SDS-V3-plasma-46_Cluster_710_sequences=451	286	451
SDS-V3-plasma-67_Cluster_382_sequences=451	504	451
SDS-V3-plasma-67_Cluster_1089_sequences=450	504	450
SDS-V3-plasma-46_Cluster_265_sequences=448	286	448
SDS-V3-plasma-7_Cluster_62_sequences=447	14	447
SDS-V3-plasma-67_Cluster_2118_sequences=446	504	446
SDS-V3-plasma-45_Cluster_590_sequences=445	282	445
SDS-V3-plasma-67_Cluster_344_sequences=445	504	445
SDS-V3-plasma-67_Cluster_1101_sequences=445	504	445
SDS-V3-plasma-67_Cluster_973_sequences=443	504	443
SDS-V3-plasma-45_Cluster_534_sequences=441	282	441
SDS-V3-plasma-67_Cluster_3074_sequences=440	504	440
SDS-V3-plasma-46_Cluster_281_sequences=439	286	439
SDS-V3-plasma-67_Cluster_100_sequences=437	504	437
SDS-V3-plasma-45_Cluster_238_sequences=436	282	436
SDS-V3-plasma-46_Cluster_405_sequences=436	286	436
SDS-V3-plasma-67_Cluster_963_sequences=435	504	435
SDS-V3-plasma-46_Cluster_160_sequences=434	286	434
SDS-V3-plasma-45_Cluster_377_sequences=433	282	433
SDS-V3-plasma-45_Cluster_812_sequences=433	282	433
SDS-V3-plasma-45_Cluster_94_sequences=432	282	432
SDS-V3-plasma-67_Cluster_1232_sequences=432	504	432

SDS-V3-plasma-67_Cluster_1481_sequences=432	504	432
SDS-V3-plasma-27_Cluster_193_sequences=431	131	431
SDS-V3-plasma-24_Cluster_74_sequences=430	124	430
SDS-V3-plasma-67_Cluster_271_sequences=430	504	430
SDS-V3-plasma-67_Cluster_607_sequences=430	504	430
SDS-V3-plasma-67_Cluster_878_sequences=430	504	430
SDS-V3-plasma-67_Cluster_1070_sequences=429	504	429
SDS-V3-plasma-0_Cluster_178_sequences=428	0	428
SDS-V3-plasma-24_Cluster_36_sequences=428	124	428
SDS-V3-plasma-67_Cluster_38_sequences=428	504	428
SDS-V3-plasma-67_Cluster_567_sequences=428	504	428
SDS-V3-plasma-7_Cluster_194_sequences=427	14	427
SDS-V3-plasma-45_Cluster_584_sequences=424	282	424
SDS-V3-plasma-45_Cluster_621_sequences=423	282	423
SDS-V3-plasma-67_Cluster_1757_sequences=419	504	419
SDS-V3-plasma-27_Cluster_352_sequences=418	131	418
SDS-V3-plasma-67_Cluster_112_sequences=417	504	417
SDS-V3-plasma-67_Cluster_6862_sequences=417	504	417
SDS-V3-plasma-7_Cluster_51_sequences=416	14	416
SDS-V3-plasma-45_Cluster_2208_sequences=415	282	415
SDS-V3-plasma-24_Cluster_258_sequences=414	124	414
SDS-V3-plasma-45_Cluster_677_sequences=412	282	412
SDS-V3-plasma-67_Cluster_1185_sequences=412	504	412
SDS-V3-plasma-45_Cluster_1492_sequences=411	282	411
SDS-V3-plasma-0_Cluster_33_sequences=410	0	410
SDS-V3-plasma-45_Cluster_582_sequences=410	282	410
SDS-V3-plasma-67_Cluster_1187_sequences=408	504	408
SDS-V3-plasma-27_Cluster_80_sequences=407	131	407
SDS-V3-plasma-45_Cluster_1027_sequences=407	282	407
SDS-V3-plasma-45_Cluster_110_sequences=405	282	405
SDS-V3-plasma-7_Cluster_94_sequences=404	14	404
SDS-V3-plasma-46_Cluster_20_sequences=404	286	404
SDS-V3-plasma-67_Cluster_1392_sequences=404	504	404
SDS-V3-plasma-24_Cluster_108_sequences=402	124	402
SDS-V3-plasma-7_Cluster_12_sequences=401	14	401
SDS-V3-plasma-67_Cluster_1051_sequences=401	504	401
SDS-V3-plasma-45_Cluster_1766_sequences=399	282	399
SDS-V3-plasma-67_Cluster_573_sequences=399	504	399
SDS-V3-plasma-0_Cluster_281_sequences=397	0	397
SDS-V3-plasma-0_Cluster_120_sequences=397	0	397
SDS-V3-plasma-45_Cluster_1577_sequences=397	282	397
SDS-V3-plasma-45_Cluster_339_sequences=397	282	397
SDS-V3-plasma-45_Cluster_419_sequences=395	282	395
SDS-V3-plasma-45_Cluster_383_sequences=394	282	394
SDS-V3-plasma-45_Cluster_573_sequences=394	282	394

SDS-V3-plasma-46_Cluster_112_sequences=394	286	394
SDS-V3-plasma-67_Cluster_2574_sequences=394	504	394
SDS-V3-plasma-45_Cluster_974_sequences=393	282	393
SDS-V3-plasma-67_Cluster_1941_sequences=393	504	393
SDS-V3-plasma-67_Cluster_762_sequences=393	504	393
SDS-V3-plasma-67_Cluster_425_sequences=391	504	391
SDS-V3-plasma-0_Cluster_211_sequences=390	0	390
SDS-V3-plasma-46_Cluster_1087_sequences=390	286	390
SDS-V3-plasma-67_Cluster_345_sequences=390	504	390
SDS-V3-plasma-67_Cluster_674_sequences=390	504	390
SDS-V3-plasma-67_Cluster_806_sequences=387	504	387
SDS-V3-plasma-67_Cluster_534_sequences=387	504	387
SDS-V3-plasma-8_Cluster_53_sequences=385	16	385
SDS-V3-plasma-45_Cluster_797_sequences=385	282	385
SDS-V3-plasma-45_Cluster_705_sequences=385	282	385
SDS-V3-plasma-0_Cluster_206_sequences=383	0	383
SDS-V3-plasma-45_Cluster_700_sequences=383	282	383
SDS-V3-plasma-67_Cluster_667_sequences=382	504	382
SDS-V3-plasma-45_Cluster_916_sequences=381	282	381
SDS-V3-plasma-67_Cluster_1633_sequences=381	504	381
SDS-V3-plasma-0_Cluster_699_sequences=379	0	379
SDS-V3-plasma-67_Cluster_111_sequences=379	504	379
SDS-V3-plasma-67_Cluster_1704_sequences=376	504	376
SDS-V3-plasma-45_Cluster_1314_sequences=374	282	374
SDS-V3-plasma-45_Cluster_136_sequences=374	282	374
SDS-V3-plasma-46_Cluster_217_sequences=374	286	374
SDS-V3-plasma-8_Cluster_2321_sequences=373	16	373
SDS-V3-plasma-27_Cluster_158_sequences=373	131	373
SDS-V3-plasma-67_Cluster_1009_sequences=372	504	372
SDS-V3-plasma-7_Cluster_230_sequences=371	14	371
SDS-V3-plasma-45_Cluster_1121_sequences=371	282	371
SDS-V3-plasma-24_Cluster_283_sequences=370	124	370
SDS-V3-plasma-45_Cluster_683_sequences=370	282	370
SDS-V3-plasma-46_Cluster_333_sequences=370	286	370
SDS-V3-plasma-67_Cluster_372_sequences=368	504	368
SDS-V3-plasma-67_Cluster_1242_sequences=366	504	366
SDS-V3-plasma-67_Cluster_113_sequences=366	504	366
SDS-V3-plasma-0_Cluster_49_sequences=365	0	365
SDS-V3-plasma-46_Cluster_623_sequences=365	286	365
SDS-V3-plasma-46_Cluster_638_sequences=365	286	365
SDS-V3-plasma-67_Cluster_332_sequences=365	504	365
SDS-V3-plasma-45_Cluster_40136_sequences=364	282	364
SDS-V3-plasma-67_Cluster_931_sequences=363	504	363
SDS-V3-plasma-67_Cluster_1324_sequences=363	504	363
SDS-V3-plasma-67_Cluster_1387_sequences=362	504	362

SDS-V3-plasma-67_Cluster_195_sequences=361	504	361
SDS-V3-plasma-67_Cluster_3428_sequences=359	504	359
SDS-V3-plasma-67_Cluster_2603_sequences=359	504	359
SDS-V3-plasma-67_Cluster_1074_sequences=359	504	359
SDS-V3-plasma-24_Cluster_18_sequences=358	124	358
SDS-V3-plasma-45_Cluster_1564_sequences=358	282	358
SDS-V3-plasma-45_Cluster_900_sequences=358	282	358
SDS-V3-plasma-7_Cluster_63_sequences=357	14	357
SDS-V3-plasma-45_Cluster_2274_sequences=357	282	357
SDS-V3-plasma-45_Cluster_426_sequences=357	282	357
SDS-V3-plasma-45_Cluster_1588_sequences=356	282	356
SDS-V3-plasma-45_Cluster_1094_sequences=356	282	356
SDS-V3-plasma-45_Cluster_1927_sequences=353	282	353
SDS-V3-plasma-0_Cluster_809_sequences=351	0	351
SDS-V3-plasma-8_Cluster_2288_sequences=351	16	351
SDS-V3-plasma-46_Cluster_686_sequences=350	286	350
SDS-V3-plasma-46_Cluster_789_sequences=350	286	350
SDS-V3-plasma-67_Cluster_563_sequences=350	504	350
SDS-V3-plasma-46_Cluster_386_sequences=349	286	349
SDS-V3-plasma-67_Cluster_751_sequences=347	504	347
SDS-V3-plasma-67_Cluster_1137_sequences=347	504	347
SDS-V3-plasma-0_Cluster_465_sequences=346	0	346
SDS-V3-plasma-45_Cluster_469_sequences=346	282	346
SDS-V3-plasma-67_Cluster_481_sequences=346	504	346
SDS-V3-plasma-45_Cluster_751_sequences=345	282	345
SDS-V3-plasma-67_Cluster_547_sequences=345	504	345
SDS-V3-plasma-45_Cluster_531_sequences=343	282	343
SDS-V3-plasma-45_Cluster_1936_sequences=341	282	341
SDS-V3-plasma-24_Cluster_43_sequences=339	124	339
SDS-V3-plasma-24_Cluster_191_sequences=337	124	337
SDS-V3-plasma-45_Cluster_337_sequences=337	282	337
SDS-V3-plasma-67_Cluster_1415_sequences=337	504	337
SDS-V3-plasma-45_Cluster_256_sequences=336	282	336
SDS-V3-plasma-67_Cluster_1138_sequences=336	504	336
SDS-V3-plasma-45_Cluster_536_sequences=334	282	334
SDS-V3-plasma-67_Cluster_330_sequences=334	504	334
SDS-V3-plasma-67_Cluster_428_sequences=334	504	334
SDS-V3-plasma-27_Cluster_5_sequences=333	131	333
SDS-V3-plasma-45_Cluster_63_sequences=332	282	332
SDS-V3-plasma-45_Cluster_218_sequences=332	282	332
SDS-V3-plasma-7_Cluster_101_sequences=331	14	331
SDS-V3-plasma-67_Cluster_565_sequences=331	504	331
SDS-V3-plasma-45_Cluster_1152_sequences=330	282	330
SDS-V3-plasma-45_Cluster_760_sequences=330	282	330
SDS-V3-plasma-67_Cluster_1912_sequences=330	504	330

SDS-V3-plasma-45_Cluster_2150_sequences=329	282	329
SDS-V3-plasma-46_Cluster_181_sequences=328	286	328
SDS-V3-plasma-67_Cluster_842_sequences=326	504	326
SDS-V3-plasma-8_Cluster_2323_sequences=325	16	325
SDS-V3-plasma-67_Cluster_958_sequences=322	504	322
SDS-V3-plasma-0_Cluster_630_sequences=321	0	321
SDS-V3-plasma-7_Cluster_443_sequences=321	14	321
SDS-V3-plasma-45_Cluster_489_sequences=321	282	321
SDS-V3-plasma-67_Cluster_406_sequences=321	504	321
SDS-V3-plasma-7_Cluster_86_sequences=320	14	320
SDS-V3-plasma-45_Cluster_666_sequences=320	282	320
SDS-V3-plasma-67_Cluster_467_sequences=319	504	319
SDS-V3-plasma-24_Cluster_2563_sequences=318	124	318
SDS-V3-plasma-46_Cluster_980_sequences=318	286	318
SDS-V3-plasma-67_Cluster_114_sequences=318	504	318
SDS-V3-plasma-67_Cluster_234_sequences=317	504	317
SDS-V3-plasma-67_Cluster_668_sequences=316	504	316
SDS-V3-plasma-46_Cluster_430_sequences=315	286	315
SDS-V3-plasma-24_Cluster_381_sequences=314	124	314
SDS-V3-plasma-67_Cluster_1574_sequences=314	504	314
SDS-V3-plasma-8_Cluster_2528_sequences=313	16	313
SDS-V3-plasma-45_Cluster_1099_sequences=313	282	313
SDS-V3-plasma-46_Cluster_791_sequences=313	286	313
SDS-V3-plasma-67_Cluster_636_sequences=313	504	313
SDS-V3-plasma-45_Cluster_2583_sequences=312	282	312
SDS-V3-plasma-67_Cluster_399_sequences=312	504	312
SDS-V3-plasma-0_Cluster_1034_sequences=311	0	311
SDS-V3-plasma-45_Cluster_2588_sequences=311	282	311
SDS-V3-plasma-45_Cluster_1387_sequences=311	282	311
SDS-V3-plasma-67_Cluster_791_sequences=311	504	311
SDS-V3-plasma-67_Cluster_853_sequences=311	504	311
SDS-V3-plasma-67_Cluster_233_sequences=311	504	311
SDS-V3-plasma-67_Cluster_250_sequences=309	504	309
SDS-V3-plasma-7_Cluster_201_sequences=307	14	307
SDS-V3-plasma-67_Cluster_584_sequences=307	504	307
SDS-V3-plasma-67_Cluster_610_sequences=307	504	307
SDS-V3-plasma-45_Cluster_919_sequences=306	282	306
SDS-V3-plasma-67_Cluster_1709_sequences=306	504	306
SDS-V3-plasma-0_Cluster_230_sequences=305	0	305
SDS-V3-plasma-67_Cluster_2255_sequences=305	504	305
SDS-V3-plasma-0_Cluster_89_sequences=304	0	304
SDS-V3-plasma-7_Cluster_93_sequences=304	14	304
SDS-V3-plasma-0_Cluster_267_sequences=303	0	303
SDS-V3-plasma-0_Cluster_162_sequences=303	0	303
SDS-V3-plasma-45_Cluster_1734_sequences=303	282	303

SDS-V3-plasma-67_Cluster_302_sequences=303	504	303
SDS-V3-plasma-67_Cluster_731_sequences=303	504	303
SDS-V3-plasma-67_Cluster_665_sequences=301	504	301
SDS-V3-plasma-45_Cluster_623_sequences=297	282	297
SDS-V3-plasma-46_Cluster_150_sequences=297	286	297
SDS-V3-plasma-67_Cluster_612_sequences=297	504	297
SDS-V3-plasma-67_Cluster_1471_sequences=297	504	297
SDS-V3-plasma-67_Cluster_884_sequences=297	504	297
SDS-V3-plasma-67_Cluster_337_sequences=296	504	296
SDS-V3-plasma-67_Cluster_807_sequences=296	504	296
SDS-V3-plasma-0_Cluster_517_sequences=294	0	294
SDS-V3-plasma-45_Cluster_1781_sequences=294	282	294
SDS-V3-plasma-46_Cluster_1176_sequences=294	286	294
SDS-V3-plasma-67_Cluster_744_sequences=294	504	294
SDS-V3-plasma-67_Cluster_2384_sequences=294	504	294
SDS-V3-plasma-24_Cluster_295_sequences=293	124	293
SDS-V3-plasma-0_Cluster_1226_sequences=292	0	292
SDS-V3-plasma-46_Cluster_272_sequences=292	286	292
SDS-V3-plasma-67_Cluster_798_sequences=291	504	291
SDS-V3-plasma-67_Cluster_373_sequences=291	504	291
SDS-V3-plasma-45_Cluster_840_sequences=290	282	290
SDS-V3-plasma-46_Cluster_389_sequences=290	286	290
SDS-V3-plasma-7_Cluster_87_sequences=288	14	288
SDS-V3-plasma-67_Cluster_219_sequences=288	504	288
SDS-V3-plasma-67_Cluster_61_sequences=288	504	288
SDS-V3-plasma-45_Cluster_1162_sequences=287	282	287
SDS-V3-plasma-67_Cluster_3480_sequences=287	504	287
SDS-V3-plasma-27_Cluster_106_sequences=286	131	286
SDS-V3-plasma-45_Cluster_1257_sequences=286	282	286
SDS-V3-plasma-45_Cluster_691_sequences=286	282	286
SDS-V3-plasma-67_Cluster_3926_sequences=286	504	286
SDS-V3-plasma-67_Cluster_803_sequences=286	504	286
SDS-V3-plasma-45_Cluster_617_sequences=285	282	285
SDS-V3-plasma-67_Cluster_2161_sequences=285	504	285
SDS-V3-plasma-67_Cluster_1722_sequences=285	504	285
SDS-V3-plasma-67_Cluster_363_sequences=284	504	284
SDS-V3-plasma-67_Cluster_93_sequences=284	504	284
SDS-V3-plasma-27_Cluster_66_sequences=283	131	283
SDS-V3-plasma-8_Cluster_2386_sequences=282	16	282
SDS-V3-plasma-27_Cluster_42_sequences=281	131	281
SDS-V3-plasma-27_Cluster_229_sequences=279	131	279
SDS-V3-plasma-27_Cluster_174_sequences=279	131	279
SDS-V3-plasma-67_Cluster_1546_sequences=279	504	279
SDS-V3-plasma-27_Cluster_146_sequences=278	131	278
SDS-V3-plasma-67_Cluster_1992_sequences=278	504	278

SDS-V3-plasma-45_Cluster_1873_sequences=277	282	277
SDS-V3-plasma-67_Cluster_405_sequences=277	504	277
SDS-V3-plasma-27_Cluster_684_sequences=276	131	276
SDS-V3-plasma-45_Cluster_1704_sequences=276	282	276
SDS-V3-plasma-67_Cluster_1742_sequences=276	504	276
SDS-V3-plasma-67_Cluster_82_sequences=276	504	276
SDS-V3-plasma-67_Cluster_559_sequences=275	504	275
SDS-V3-plasma-0_Cluster_1444_sequences=274	0	274
SDS-V3-plasma-46_Cluster_445_sequences=274	286	274
SDS-V3-plasma-67_Cluster_3164_sequences=274	504	274
SDS-V3-plasma-24_Cluster_270_sequences=273	124	273
SDS-V3-plasma-67_Cluster_1710_sequences=272	504	272
SDS-V3-plasma-67_Cluster_2899_sequences=272	504	272
SDS-V3-plasma-24_Cluster_35_sequences=271	124	271
SDS-V3-plasma-67_Cluster_596_sequences=271	504	271
SDS-V3-plasma-24_Cluster_567_sequences=270	124	270
SDS-V3-plasma-67_Cluster_2696_sequences=270	504	270
SDS-V3-plasma-67_Cluster_435_sequences=270	504	270
SDS-V3-plasma-0_Cluster_294_sequences=269	0	269
SDS-V3-plasma-45_Cluster_1017_sequences=269	282	269
SDS-V3-plasma-46_Cluster_479_sequences=269	286	269
SDS-V3-plasma-0_Cluster_439_sequences=268	0	268
SDS-V3-plasma-27_Cluster_122_sequences=268	131	268
SDS-V3-plasma-67_Cluster_1460_sequences=268	504	268
SDS-V3-plasma-67_Cluster_536_sequences=268	504	268
SDS-V3-plasma-67_Cluster_3194_sequences=267	504	267
SDS-V3-plasma-67_Cluster_160_sequences=267	504	267
SDS-V3-plasma-8_Cluster_2263_sequences=266	16	266
SDS-V3-plasma-67_Cluster_1491_sequences=266	504	266
SDS-V3-plasma-46_Cluster_13_sequences=265	286	265
SDS-V3-plasma-67_Cluster_2911_sequences=265	504	265
SDS-V3-plasma-0_Cluster_343_sequences=264	0	264
SDS-V3-plasma-67_Cluster_200_sequences=263	504	263
SDS-V3-plasma-67_Cluster_1632_sequences=263	504	263
SDS-V3-plasma-67_Cluster_515_sequences=263	504	263
SDS-V3-plasma-45_Cluster_5172_sequences=262	282	262
SDS-V3-plasma-67_Cluster_2089_sequences=262	504	262
SDS-V3-plasma-45_Cluster_1407_sequences=261	282	261
SDS-V3-plasma-67_Cluster_1355_sequences=261	504	261
SDS-V3-plasma-67_Cluster_522_sequences=261	504	261
SDS-V3-plasma-67_Cluster_970_sequences=261	504	261
SDS-V3-plasma-67_Cluster_713_sequences=261	504	261
SDS-V3-plasma-46_Cluster_367_sequences=260	286	260
SDS-V3-plasma-67_Cluster_2441_sequences=260	504	260
SDS-V3-plasma-0_Cluster_124_sequences=259	0	259

SDS-V3-plasma-67_Cluster_220_sequences=258	504	258
SDS-V3-plasma-46_Cluster_546_sequences=257	286	257
SDS-V3-plasma-67_Cluster_221_sequences=257	504	257
SDS-V3-plasma-27_Cluster_155_sequences=256	131	256
SDS-V3-plasma-46_Cluster_711_sequences=256	286	256
SDS-V3-plasma-67_Cluster_106_sequences=256	504	256
SDS-V3-plasma-45_Cluster_264_sequences=255	282	255
SDS-V3-plasma-45_Cluster_1646_sequences=255	282	255
SDS-V3-plasma-45_Cluster_436_sequences=255	282	255
SDS-V3-plasma-45_Cluster_661_sequences=255	282	255
SDS-V3-plasma-45_Cluster_168_sequences=255	282	255
SDS-V3-plasma-67_Cluster_657_sequences=255	504	255
SDS-V3-plasma-67_Cluster_838_sequences=255	504	255
SDS-V3-plasma-46_Cluster_81_sequences=254	286	254
SDS-V3-plasma-67_Cluster_209_sequences=254	504	254
SDS-V3-plasma-67_Cluster_1327_sequences=254	504	254
SDS-V3-plasma-67_Cluster_661_sequences=254	504	254
SDS-V3-plasma-67_Cluster_1126_sequences=253	504	253
SDS-V3-plasma-67_Cluster_1046_sequences=253	504	253
SDS-V3-plasma-0_Cluster_523_sequences=252	0	252
SDS-V3-plasma-45_Cluster_4835_sequences=252	282	252
SDS-V3-plasma-45_Cluster_334_sequences=252	282	252
SDS-V3-plasma-45_Cluster_124_sequences=251	282	251
SDS-V3-plasma-46_Cluster_484_sequences=250	286	250
SDS-V3-plasma-67_Cluster_49_sequences=250	504	250
SDS-V3-plasma-0_Cluster_8388_sequences=249	0	249
SDS-V3-plasma-45_Cluster_1807_sequences=249	282	249
SDS-V3-plasma-5_Cluster_94_sequences=248	9	248
SDS-V3-plasma-8_Cluster_2297_sequences=248	16	248
SDS-V3-plasma-67_Cluster_202_sequences=248	504	248
SDS-V3-plasma-24_Cluster_292_sequences=247	124	247
SDS-V3-plasma-67_Cluster_608_sequences=247	504	247
SDS-V3-plasma-67_Cluster_2159_sequences=246	504	246
SDS-V3-plasma-67_Cluster_1785_sequences=246	504	246
SDS-V3-plasma-27_Cluster_19_sequences=245	131	245
SDS-V3-plasma-67_Cluster_1751_sequences=245	504	245
SDS-V3-plasma-67_Cluster_1746_sequences=245	504	245
SDS-V3-plasma-67_Cluster_718_sequences=245	504	245
SDS-V3-plasma-8_Cluster_2465_sequences=244	16	244
SDS-V3-plasma-27_Cluster_117_sequences=244	131	244
SDS-V3-plasma-46_Cluster_111_sequences=244	286	244
SDS-V3-plasma-0_Cluster_266_sequences=243	0	243
SDS-V3-plasma-67_Cluster_222_sequences=243	504	243
SDS-V3-plasma-67_Cluster_94_sequences=242	504	242
SDS-V3-plasma-67_Cluster_1784_sequences=242	504	242

SDS-V3-plasma-67_Cluster_2310_sequences=241	504	241
SDS-V3-plasma-67_Cluster_474_sequences=241	504	241
SDS-V3-plasma-67_Cluster_549_sequences=241	504	241
SDS-V3-plasma-45_Cluster_950_sequences=240	282	240
SDS-V3-plasma-45_Cluster_405_sequences=240	282	240
SDS-V3-plasma-45_Cluster_2122_sequences=240	282	240
SDS-V3-plasma-67_Cluster_1590_sequences=240	504	240
SDS-V3-plasma-45_Cluster_399_sequences=239	282	239
SDS-V3-plasma-45_Cluster_699_sequences=239	282	239
SDS-V3-plasma-46_Cluster_444_sequences=239	286	239
SDS-V3-plasma-67_Cluster_2043_sequences=239	504	239
SDS-V3-plasma-0_Cluster_482_sequences=238	0	238
SDS-V3-plasma-67_Cluster_3614_sequences=238	504	238
SDS-V3-plasma-24_Cluster_132_sequences=237	124	237
SDS-V3-plasma-27_Cluster_6_sequences=237	131	237
SDS-V3-plasma-46_Cluster_513_sequences=237	286	237
SDS-V3-plasma-46_Cluster_446_sequences=237	286	237
SDS-V3-plasma-67_Cluster_2038_sequences=237	504	237
SDS-V3-plasma-67_Cluster_41_sequences=237	504	237
SDS-V3-plasma-67_Cluster_35_sequences=237	504	237
SDS-V3-plasma-8_Cluster_30_sequences=235	16	235
SDS-V3-plasma-45_Cluster_280_sequences=235	282	235
SDS-V3-plasma-46_Cluster_738_sequences=235	286	235
SDS-V3-plasma-67_Cluster_883_sequences=235	504	235
SDS-V3-plasma-67_Cluster_237_sequences=235	504	235
SDS-V3-plasma-45_Cluster_191_sequences=234	282	234
SDS-V3-plasma-27_Cluster_590_sequences=233	131	233
SDS-V3-plasma-67_Cluster_2778_sequences=233	504	233
SDS-V3-plasma-67_Cluster_137_sequences=233	504	233
SDS-V3-plasma-8_Cluster_2394_sequences=232	16	232
SDS-V3-plasma-24_Cluster_10_sequences=232	124	232
SDS-V3-plasma-27_Cluster_108_sequences=232	131	232
SDS-V3-plasma-67_Cluster_2566_sequences=232	504	232
SDS-V3-plasma-0_Cluster_65_sequences=231	0	231
SDS-V3-plasma-45_Cluster_254_sequences=231	282	231
SDS-V3-plasma-67_Cluster_2199_sequences=231	504	231
SDS-V3-plasma-67_Cluster_3013_sequences=231	504	231
SDS-V3-plasma-67_Cluster_56_sequences=231	504	231
SDS-V3-plasma-45_Cluster_101_sequences=230	282	230
SDS-V3-plasma-45_Cluster_4703_sequences=229	282	229
SDS-V3-plasma-0_Cluster_589_sequences=228	0	228
SDS-V3-plasma-24_Cluster_390_sequences=228	124	228
SDS-V3-plasma-45_Cluster_1410_sequences=228	282	228
SDS-V3-plasma-67_Cluster_2719_sequences=228	504	228
SDS-V3-plasma-0_Cluster_50_sequences=227	0	227

SDS-V3-plasma-27_Cluster_351_sequences=227	131	227
SDS-V3-plasma-45_Cluster_3505_sequences=227	282	227
SDS-V3-plasma-45_Cluster_1223_sequences=227	282	227
SDS-V3-plasma-45_Cluster_11870_sequences=227	282	227
SDS-V3-plasma-67_Cluster_863_sequences=227	504	227
SDS-V3-plasma-67_Cluster_2654_sequences=227	504	227
SDS-V3-plasma-45_Cluster_494_sequences=226	282	226
SDS-V3-plasma-67_Cluster_1729_sequences=226	504	226
SDS-V3-plasma-46_Cluster_492_sequences=225	286	225
SDS-V3-plasma-67_Cluster_1603_sequences=225	504	225
SDS-V3-plasma-67_Cluster_1270_sequences=225	504	225
SDS-V3-plasma-67_Cluster_833_sequences=225	504	225
SDS-V3-plasma-67_Cluster_1249_sequences=224	504	224
SDS-V3-plasma-7_Cluster_189_sequences=223	14	223
SDS-V3-plasma-45_Cluster_2724_sequences=223	282	223
SDS-V3-plasma-67_Cluster_1381_sequences=223	504	223
SDS-V3-plasma-0_Cluster_153_sequences=222	0	222
SDS-V3-plasma-0_Cluster_638_sequences=222	0	222
SDS-V3-plasma-7_Cluster_1_sequences=222	14	222
SDS-V3-plasma-67_Cluster_2555_sequences=222	504	222
SDS-V3-plasma-67_Cluster_3476_sequences=222	504	222
SDS-V3-plasma-67_Cluster_520_sequences=222	504	222
SDS-V3-plasma-67_Cluster_3119_sequences=222	504	222
SDS-V3-plasma-0_Cluster_159_sequences=221	0	221
SDS-V3-plasma-45_Cluster_2419_sequences=221	282	221
SDS-V3-plasma-46_Cluster_360_sequences=221	286	221
SDS-V3-plasma-67_Cluster_734_sequences=221	504	221
SDS-V3-plasma-67_Cluster_2259_sequences=221	504	221
SDS-V3-plasma-7_Cluster_283_sequences=220	14	220
SDS-V3-plasma-8_Cluster_2473_sequences=220	16	220
SDS-V3-plasma-45_Cluster_2908_sequences=220	282	220
SDS-V3-plasma-67_Cluster_2643_sequences=220	504	220
SDS-V3-plasma-67_Cluster_2629_sequences=220	504	220
SDS-V3-plasma-67_Cluster_752_sequences=220	504	220
SDS-V3-plasma-0_Cluster_56_sequences=219	0	219
SDS-V3-plasma-0_Cluster_90_sequences=219	0	219
SDS-V3-plasma-7_Cluster_265_sequences=219	14	219
SDS-V3-plasma-24_Cluster_179_sequences=219	124	219
SDS-V3-plasma-45_Cluster_77_sequences=219	282	219
SDS-V3-plasma-27_Cluster_252_sequences=218	131	218
SDS-V3-plasma-45_Cluster_38_sequences=218	282	218
SDS-V3-plasma-7_Cluster_323_sequences=217	14	217
SDS-V3-plasma-46_Cluster_409_sequences=217	286	217
SDS-V3-plasma-7_Cluster_659_sequences=216	14	216
SDS-V3-plasma-45_Cluster_302_sequences=216	282	216

SDS-V3-plasma-45_Cluster_448_sequences=216	282	216
SDS-V3-plasma-46_Cluster_640_sequences=216	286	216
SDS-V3-plasma-67_Cluster_408_sequences=216	504	216
SDS-V3-plasma-24_Cluster_130_sequences=215	124	215
SDS-V3-plasma-45_Cluster_2587_sequences=215	282	215
SDS-V3-plasma-46_Cluster_545_sequences=215	286	215
SDS-V3-plasma-67_Cluster_3806_sequences=215	504	215
SDS-V3-plasma-45_Cluster_1458_sequences=214	282	214
SDS-V3-plasma-67_Cluster_2104_sequences=214	504	214
SDS-V3-plasma-67_Cluster_398_sequences=214	504	214
SDS-V3-plasma-27_Cluster_133_sequences=213	131	213
SDS-V3-plasma-67_Cluster_83_sequences=213	504	213
SDS-V3-plasma-27_Cluster_284_sequences=212	131	212
SDS-V3-plasma-46_Cluster_868_sequences=212	286	212
SDS-V3-plasma-46_Cluster_859_sequences=212	286	212
SDS-V3-plasma-67_Cluster_438_sequences=212	504	212
SDS-V3-plasma-67_Cluster_130_sequences=212	504	212
SDS-V3-plasma-7_Cluster_720_sequences=211	14	211
SDS-V3-plasma-67_Cluster_1638_sequences=211	504	211
SDS-V3-plasma-0_Cluster_247_sequences=210	0	210
SDS-V3-plasma-45_Cluster_805_sequences=210	282	210
SDS-V3-plasma-45_Cluster_2996_sequences=210	282	210
SDS-V3-plasma-46_Cluster_365_sequences=210	286	210
SDS-V3-plasma-67_Cluster_2270_sequences=210	504	210
SDS-V3-plasma-67_Cluster_1139_sequences=209	504	209
SDS-V3-plasma-0_Cluster_2080_sequences=208	0	208
SDS-V3-plasma-0_Cluster_636_sequences=208	0	208
SDS-V3-plasma-24_Cluster_122_sequences=208	124	208
SDS-V3-plasma-67_Cluster_475_sequences=208	504	208
SDS-V3-plasma-67_Cluster_952_sequences=208	504	208
SDS-V3-plasma-45_Cluster_449_sequences=207	282	207
SDS-V3-plasma-27_Cluster_184_sequences=206	131	206
SDS-V3-plasma-45_Cluster_615_sequences=206	282	206
SDS-V3-plasma-0_Cluster_836_sequences=205	0	205
SDS-V3-plasma-45_Cluster_1248_sequences=205	282	205
SDS-V3-plasma-46_Cluster_502_sequences=205	286	205
SDS-V3-plasma-67_Cluster_333_sequences=205	504	205
SDS-V3-plasma-67_Cluster_322_sequences=205	504	205
SDS-V3-plasma-45_Cluster_442_sequences=204	282	204
SDS-V3-plasma-45_Cluster_808_sequences=204	282	204
SDS-V3-plasma-45_Cluster_362_sequences=204	282	204
SDS-V3-plasma-67_Cluster_1208_sequences=204	504	204
SDS-V3-plasma-27_Cluster_903_sequences=203	131	203
SDS-V3-plasma-45_Cluster_561_sequences=203	282	203
SDS-V3-plasma-46_Cluster_352_sequences=202	286	202

SDS-V3-plasma-46_Cluster_314_sequences=202	286	202
SDS-V3-plasma-46_Cluster_245_sequences=202	286	202
SDS-V3-plasma-67_Cluster_2931_sequences=202	504	202
SDS-V3-plasma-67_Cluster_2029_sequences=202	504	202
SDS-V3-plasma-67_Cluster_1459_sequences=202	504	202
SDS-V3-plasma-24_Cluster_11_sequences=201	124	201
SDS-V3-plasma-45_Cluster_1992_sequences=201	282	201
SDS-V3-plasma-46_Cluster_1435_sequences=201	286	201
SDS-V3-plasma-45_Cluster_3031_sequences=200	282	200
SDS-V3-plasma-45_Cluster_3160_sequences=200	282	200
SDS-V3-plasma-67_Cluster_540_sequences=200	504	200
SDS-V3-plasma-0_Cluster_189_sequences=199	0	199
SDS-V3-plasma-24_Cluster_221_sequences=199	124	199
SDS-V3-plasma-45_Cluster_311_sequences=199	282	199
SDS-V3-plasma-7_Cluster_31_sequences=198	14	198
SDS-V3-plasma-24_Cluster_38_sequences=198	124	198
SDS-V3-plasma-45_Cluster_1412_sequences=198	282	198
SDS-V3-plasma-67_Cluster_712_sequences=198	504	198
SDS-V3-plasma-67_Cluster_2511_sequences=198	504	198
SDS-V3-plasma-67_Cluster_75_sequences=198	504	198
SDS-V3-plasma-67_Cluster_410_sequences=198	504	198
SDS-V3-plasma-67_Cluster_1718_sequences=198	504	198
SDS-V3-plasma-67_Cluster_64_sequences=198	504	198
SDS-V3-plasma-45_Cluster_2898_sequences=197	282	197
SDS-V3-plasma-45_Cluster_2142_sequences=197	282	197
SDS-V3-plasma-45_Cluster_1272_sequences=197	282	197
SDS-V3-plasma-67_Cluster_1055_sequences=197	504	197
SDS-V3-plasma-67_Cluster_626_sequences=197	504	197
SDS-V3-plasma-0_Cluster_1002_sequences=196	0	196
SDS-V3-plasma-8_Cluster_2284_sequences=196	16	196
SDS-V3-plasma-45_Cluster_1097_sequences=196	282	196
SDS-V3-plasma-0_Cluster_394_sequences=195	0	195
SDS-V3-plasma-0_Cluster_106_sequences=195	0	195
SDS-V3-plasma-45_Cluster_2140_sequences=195	282	195
SDS-V3-plasma-45_Cluster_2178_sequences=195	282	195
SDS-V3-plasma-67_Cluster_3342_sequences=195	504	195
SDS-V3-plasma-67_Cluster_2237_sequences=194	504	194
SDS-V3-plasma-67_Cluster_1100_sequences=194	504	194
SDS-V3-plasma-67_Cluster_1597_sequences=194	504	194
SDS-V3-plasma-24_Cluster_127_sequences=193	124	193
SDS-V3-plasma-45_Cluster_1411_sequences=193	282	193
SDS-V3-plasma-67_Cluster_949_sequences=193	504	193
SDS-V3-plasma-67_Cluster_348_sequences=193	504	193
SDS-V3-plasma-67_Cluster_1036_sequences=193	504	193
SDS-V3-plasma-8_Cluster_2899_sequences=192	16	192

SDS-V3-plasma-24_Cluster_362_sequences=192	124	192
SDS-V3-plasma-27_Cluster_493_sequences=192	131	192
SDS-V3-plasma-46_Cluster_694_sequences=192	286	192
SDS-V3-plasma-67_Cluster_1970_sequences=192	504	192
SDS-V3-plasma-45_Cluster_471_sequences=191	282	191
SDS-V3-plasma-45_Cluster_902_sequences=191	282	191
SDS-V3-plasma-67_Cluster_2548_sequences=191	504	191
SDS-V3-plasma-7_Cluster_209_sequences=190	14	190
SDS-V3-plasma-45_Cluster_143_sequences=190	282	190
SDS-V3-plasma-67_Cluster_1478_sequences=190	504	190
SDS-V3-plasma-0_Cluster_1086_sequences=189	0	189
SDS-V3-plasma-45_Cluster_1922_sequences=189	282	189
SDS-V3-plasma-46_Cluster_534_sequences=189	286	189
SDS-V3-plasma-67_Cluster_810_sequences=189	504	189
SDS-V3-plasma-67_Cluster_300_sequences=188	504	188
SDS-V3-plasma-67_Cluster_228_sequences=187	504	187
SDS-V3-plasma-67_Cluster_3845_sequences=187	504	187
SDS-V3-plasma-8_Cluster_2429_sequences=186	16	186
SDS-V3-plasma-67_Cluster_1127_sequences=186	504	186
SDS-V3-plasma-67_Cluster_338_sequences=186	504	186
SDS-V3-plasma-0_Cluster_63_sequences=185	0	185
SDS-V3-plasma-45_Cluster_702_sequences=185	282	185
SDS-V3-plasma-46_Cluster_363_sequences=185	286	185
SDS-V3-plasma-46_Cluster_580_sequences=185	286	185
SDS-V3-plasma-27_Cluster_687_sequences=184	131	184
SDS-V3-plasma-45_Cluster_1598_sequences=184	282	184
SDS-V3-plasma-45_Cluster_517_sequences=184	282	184
SDS-V3-plasma-46_Cluster_165_sequences=184	286	184
SDS-V3-plasma-67_Cluster_919_sequences=184	504	184
SDS-V3-plasma-67_Cluster_176_sequences=184	504	184
SDS-V3-plasma-67_Cluster_2422_sequences=184	504	184
SDS-V3-plasma-7_Cluster_73_sequences=183	14	183
SDS-V3-plasma-8_Cluster_2508_sequences=183	16	183
SDS-V3-plasma-45_Cluster_2568_sequences=183	282	183
SDS-V3-plasma-67_Cluster_4206_sequences=183	504	183
SDS-V3-plasma-67_Cluster_423_sequences=183	504	183
SDS-V3-plasma-8_Cluster_2517_sequences=182	16	182
SDS-V3-plasma-27_Cluster_2957_sequences=182	131	182
SDS-V3-plasma-45_Cluster_609_sequences=182	282	182
SDS-V3-plasma-45_Cluster_173_sequences=182	282	182
SDS-V3-plasma-67_Cluster_90_sequences=182	504	182
SDS-V3-plasma-67_Cluster_2451_sequences=182	504	182
SDS-V3-plasma-67_Cluster_5386_sequences=181	504	181
SDS-V3-plasma-67_Cluster_44_sequences=181	504	181
SDS-V3-plasma-7_Cluster_164_sequences=180	14	180

SDS-V3-plasma-45_Cluster_1453_sequences=180	282	180
SDS-V3-plasma-45_Cluster_3133_sequences=180	282	180
SDS-V3-plasma-45_Cluster_76_sequences=180	282	180
SDS-V3-plasma-45_Cluster_1517_sequences=180	282	180
SDS-V3-plasma-67_Cluster_3502_sequences=180	504	180
SDS-V3-plasma-0_Cluster_1024_sequences=179	0	179
SDS-V3-plasma-24_Cluster_138_sequences=179	124	179
SDS-V3-plasma-24_Cluster_530_sequences=179	124	179
SDS-V3-plasma-45_Cluster_3211_sequences=179	282	179
SDS-V3-plasma-67_Cluster_2789_sequences=179	504	179
SDS-V3-plasma-67_Cluster_857_sequences=179	504	179
SDS-V3-plasma-67_Cluster_4544_sequences=179	504	179
SDS-V3-plasma-8_Cluster_2511_sequences=178	16	178
SDS-V3-plasma-45_Cluster_857_sequences=178	282	178
SDS-V3-plasma-67_Cluster_1005_sequences=178	504	178
SDS-V3-plasma-0_Cluster_631_sequences=177	0	177
SDS-V3-plasma-0_Cluster_185_sequences=177	0	177
SDS-V3-plasma-45_Cluster_2421_sequences=177	282	177
SDS-V3-plasma-45_Cluster_3079_sequences=177	282	177
SDS-V3-plasma-45_Cluster_1933_sequences=177	282	177
SDS-V3-plasma-67_Cluster_2533_sequences=177	504	177
SDS-V3-plasma-67_Cluster_2736_sequences=177	504	177
SDS-V3-plasma-67_Cluster_699_sequences=177	504	177
SDS-V3-plasma-45_Cluster_3563_sequences=176	282	176
SDS-V3-plasma-67_Cluster_1791_sequences=176	504	176
SDS-V3-plasma-67_Cluster_4415_sequences=176	504	176
SDS-V3-plasma-67_Cluster_1999_sequences=176	504	176
SDS-V3-plasma-7_Cluster_309_sequences=175	14	175
SDS-V3-plasma-67_Cluster_1771_sequences=175	504	175
SDS-V3-plasma-67_Cluster_2821_sequences=175	504	175
SDS-V3-plasma-67_Cluster_395_sequences=175	504	175
SDS-V3-plasma-27_Cluster_207_sequences=174	131	174
SDS-V3-plasma-27_Cluster_10_sequences=174	131	174
SDS-V3-plasma-45_Cluster_1155_sequences=174	282	174
SDS-V3-plasma-67_Cluster_355_sequences=174	504	174
SDS-V3-plasma-67_Cluster_4786_sequences=174	504	174
SDS-V3-plasma-67_Cluster_909_sequences=174	504	174
SDS-V3-plasma-67_Cluster_214_sequences=174	504	174
SDS-V3-plasma-67_Cluster_2076_sequences=174	504	174
SDS-V3-plasma-8_Cluster_2340_sequences=173	16	173
SDS-V3-plasma-45_Cluster_2748_sequences=173	282	173
SDS-V3-plasma-45_Cluster_3476_sequences=173	282	173
SDS-V3-plasma-67_Cluster_836_sequences=173	504	173
SDS-V3-plasma-67_Cluster_5266_sequences=173	504	173
SDS-V3-plasma-67_Cluster_966_sequences=173	504	173

SDS-V3-plasma-8_Cluster_2413_sequences=172	16	172
SDS-V3-plasma-45_Cluster_4473_sequences=172	282	172
SDS-V3-plasma-45_Cluster_2257_sequences=172	282	172
SDS-V3-plasma-0_Cluster_334_sequences=171	0	171
SDS-V3-plasma-7_Cluster_216_sequences=171	14	171
SDS-V3-plasma-27_Cluster_94_sequences=171	131	171
SDS-V3-plasma-45_Cluster_1425_sequences=171	282	171
SDS-V3-plasma-45_Cluster_1625_sequences=171	282	171
SDS-V3-plasma-45_Cluster_2854_sequences=171	282	171
SDS-V3-plasma-67_Cluster_3066_sequences=171	504	171
SDS-V3-plasma-0_Cluster_983_sequences=170	0	170
SDS-V3-plasma-8_Cluster_2277_sequences=170	16	170
SDS-V3-plasma-27_Cluster_125_sequences=170	131	170
SDS-V3-plasma-27_Cluster_793_sequences=170	131	170
SDS-V3-plasma-45_Cluster_416_sequences=170	282	170
SDS-V3-plasma-45_Cluster_484_sequences=170	282	170
SDS-V3-plasma-46_Cluster_551_sequences=170	286	170
SDS-V3-plasma-67_Cluster_824_sequences=170	504	170
SDS-V3-plasma-0_Cluster_244_sequences=169	0	169
SDS-V3-plasma-45_Cluster_75_sequences=169	282	169
SDS-V3-plasma-45_Cluster_1705_sequences=169	282	169
SDS-V3-plasma-67_Cluster_409_sequences=169	504	169
SDS-V3-plasma-67_Cluster_2773_sequences=169	504	169
SDS-V3-plasma-67_Cluster_54_sequences=169	504	169
SDS-V3-plasma-0_Cluster_378_sequences=168	0	168
SDS-V3-plasma-45_Cluster_998_sequences=168	282	168
SDS-V3-plasma-46_Cluster_709_sequences=168	286	168
SDS-V3-plasma-46_Cluster_77_sequences=168	286	168
SDS-V3-plasma-67_Cluster_1207_sequences=168	504	168
SDS-V3-plasma-67_Cluster_5570_sequences=168	504	168
SDS-V3-plasma-67_Cluster_2985_sequences=168	504	168
SDS-V3-plasma-67_Cluster_1477_sequences=168	504	168
SDS-V3-plasma-45_Cluster_2847_sequences=167	282	167
SDS-V3-plasma-45_Cluster_775_sequences=167	282	167
SDS-V3-plasma-0_Cluster_99_sequences=166	0	166
SDS-V3-plasma-8_Cluster_2466_sequences=166	16	166
SDS-V3-plasma-8_Cluster_2499_sequences=166	16	166
SDS-V3-plasma-24_Cluster_429_sequences=166	124	166
SDS-V3-plasma-45_Cluster_901_sequences=166	282	166
SDS-V3-plasma-45_Cluster_3621_sequences=166	282	166
SDS-V3-plasma-46_Cluster_234_sequences=166	286	166
SDS-V3-plasma-46_Cluster_26_sequences=166	286	166
SDS-V3-plasma-46_Cluster_260_sequences=166	286	166
SDS-V3-plasma-67_Cluster_2875_sequences=166	504	166
SDS-V3-plasma-67_Cluster_2607_sequences=166	504	166

SDS-V3-plasma-24_Cluster_372_sequences=165	124	165
SDS-V3-plasma-45_Cluster_2287_sequences=165	282	165
SDS-V3-plasma-46_Cluster_62_sequences=165	286	165
SDS-V3-plasma-67_Cluster_647_sequences=165	504	165
SDS-V3-plasma-67_Cluster_1582_sequences=165	504	165
SDS-V3-plasma-67_Cluster_1171_sequences=165	504	165
SDS-V3-plasma-8_Cluster_2739_sequences=164	16	164
SDS-V3-plasma-45_Cluster_658_sequences=164	282	164
SDS-V3-plasma-67_Cluster_2375_sequences=164	504	164
SDS-V3-plasma-67_Cluster_2037_sequences=164	504	164
SDS-V3-plasma-67_Cluster_471_sequences=164	504	164
SDS-V3-plasma-67_Cluster_933_sequences=164	504	164
SDS-V3-plasma-0_Cluster_635_sequences=163	0	163
SDS-V3-plasma-8_Cluster_38_sequences=163	16	163
SDS-V3-plasma-27_Cluster_200_sequences=163	131	163
SDS-V3-plasma-67_Cluster_133_sequences=163	504	163
SDS-V3-plasma-67_Cluster_3180_sequences=163	504	163
SDS-V3-plasma-24_Cluster_46_sequences=162	124	162
SDS-V3-plasma-45_Cluster_4159_sequences=162	282	162
SDS-V3-plasma-45_Cluster_487_sequences=162	282	162
SDS-V3-plasma-45_Cluster_4352_sequences=162	282	162
SDS-V3-plasma-45_Cluster_1852_sequences=162	282	162
SDS-V3-plasma-45_Cluster_3654_sequences=162	282	162
SDS-V3-plasma-45_Cluster_1685_sequences=162	282	162
SDS-V3-plasma-46_Cluster_677_sequences=162	286	162
SDS-V3-plasma-67_Cluster_478_sequences=162	504	162
SDS-V3-plasma-67_Cluster_1057_sequences=162	504	162
SDS-V3-plasma-67_Cluster_343_sequences=162	504	162
SDS-V3-plasma-27_Cluster_450_sequences=161	131	161
SDS-V3-plasma-45_Cluster_618_sequences=161	282	161
SDS-V3-plasma-45_Cluster_418_sequences=161	282	161
SDS-V3-plasma-45_Cluster_867_sequences=161	282	161
SDS-V3-plasma-45_Cluster_5079_sequences=161	282	161
SDS-V3-plasma-67_Cluster_2209_sequences=161	504	161
SDS-V3-plasma-67_Cluster_1362_sequences=161	504	161
SDS-V3-plasma-67_Cluster_2866_sequences=161	504	161
SDS-V3-plasma-8_Cluster_2276_sequences=160	16	160
SDS-V3-plasma-24_Cluster_2203_sequences=160	124	160
SDS-V3-plasma-24_Cluster_342_sequences=160	124	160
SDS-V3-plasma-45_Cluster_748_sequences=160	282	160
SDS-V3-plasma-45_Cluster_1291_sequences=160	282	160
SDS-V3-plasma-46_Cluster_866_sequences=160	286	160
SDS-V3-plasma-67_Cluster_840_sequences=160	504	160
SDS-V3-plasma-67_Cluster_593_sequences=160	504	160
SDS-V3-plasma-7_Cluster_300_sequences=159	14	159

SDS-V3-plasma-24_Cluster_465_sequences=159	124	159
SDS-V3-plasma-45_Cluster_3698_sequences=159	282	159
SDS-V3-plasma-45_Cluster_4718_sequences=159	282	159
SDS-V3-plasma-0_Cluster_529_sequences=158	0	158
SDS-V3-plasma-24_Cluster_290_sequences=158	124	158
SDS-V3-plasma-45_Cluster_1595_sequences=158	282	158
SDS-V3-plasma-45_Cluster_5254_sequences=158	282	158
SDS-V3-plasma-45_Cluster_3846_sequences=158	282	158
SDS-V3-plasma-67_Cluster_1715_sequences=158	504	158
SDS-V3-plasma-67_Cluster_1230_sequences=158	504	158
SDS-V3-plasma-27_Cluster_99_sequences=157	131	157
SDS-V3-plasma-45_Cluster_61_sequences=157	282	157
SDS-V3-plasma-45_Cluster_1164_sequences=157	282	157
SDS-V3-plasma-67_Cluster_2357_sequences=157	504	157
SDS-V3-plasma-67_Cluster_523_sequences=157	504	157
SDS-V3-plasma-45_Cluster_688_sequences=156	282	156
SDS-V3-plasma-45_Cluster_71_sequences=156	282	156
SDS-V3-plasma-67_Cluster_472_sequences=156	504	156
SDS-V3-plasma-67_Cluster_703_sequences=156	504	156
SDS-V3-plasma-67_Cluster_784_sequences=156	504	156
SDS-V3-plasma-67_Cluster_4373_sequences=156	504	156
SDS-V3-plasma-0_Cluster_733_sequences=155	0	155
SDS-V3-plasma-24_Cluster_229_sequences=155	124	155
SDS-V3-plasma-45_Cluster_1708_sequences=155	282	155
SDS-V3-plasma-45_Cluster_2857_sequences=155	282	155
SDS-V3-plasma-45_Cluster_955_sequences=155	282	155
SDS-V3-plasma-45_Cluster_1569_sequences=155	282	155
SDS-V3-plasma-67_Cluster_4219_sequences=155	504	155
SDS-V3-plasma-24_Cluster_541_sequences=154	124	154
SDS-V3-plasma-27_Cluster_424_sequences=154	131	154
SDS-V3-plasma-45_Cluster_1854_sequences=154	282	154
SDS-V3-plasma-67_Cluster_819_sequences=154	504	154
SDS-V3-plasma-67_Cluster_4036_sequences=154	504	154
SDS-V3-plasma-0_Cluster_155_sequences=153	0	153
SDS-V3-plasma-0_Cluster_95_sequences=153	0	153
SDS-V3-plasma-8_Cluster_2537_sequences=153	16	153
SDS-V3-plasma-8_Cluster_2295_sequences=153	16	153
SDS-V3-plasma-24_Cluster_815_sequences=153	124	153
SDS-V3-plasma-24_Cluster_50_sequences=153	124	153
SDS-V3-plasma-45_Cluster_1321_sequences=153	282	153
SDS-V3-plasma-67_Cluster_1708_sequences=153	504	153
SDS-V3-plasma-7_Cluster_53_sequences=152	14	152
SDS-V3-plasma-45_Cluster_1063_sequences=152	282	152
SDS-V3-plasma-46_Cluster_617_sequences=152	286	152
SDS-V3-plasma-46_Cluster_312_sequences=152	286	152

SDS-V3-plasma-67_Cluster_2474_sequences=152	504	152
SDS-V3-plasma-45_Cluster_1711_sequences=151	282	151
SDS-V3-plasma-46_Cluster_1936_sequences=151	286	151
SDS-V3-plasma-67_Cluster_4731_sequences=151	504	151
SDS-V3-plasma-0_Cluster_2071_sequences=150	0	150
SDS-V3-plasma-24_Cluster_788_sequences=150	124	150
SDS-V3-plasma-45_Cluster_204_sequences=150	282	150
SDS-V3-plasma-27_Cluster_4_sequences=149	131	149
SDS-V3-plasma-46_Cluster_193_sequences=149	286	149
SDS-V3-plasma-46_Cluster_503_sequences=149	286	149
SDS-V3-plasma-67_Cluster_4504_sequences=149	504	149
SDS-V3-plasma-67_Cluster_1711_sequences=149	504	149
SDS-V3-plasma-27_Cluster_18_sequences=148	131	148
SDS-V3-plasma-27_Cluster_23_sequences=148	131	148
SDS-V3-plasma-27_Cluster_116_sequences=148	131	148
SDS-V3-plasma-45_Cluster_3114_sequences=148	282	148
SDS-V3-plasma-45_Cluster_1475_sequences=148	282	148
SDS-V3-plasma-45_Cluster_106_sequences=148	282	148
SDS-V3-plasma-45_Cluster_5233_sequences=148	282	148
SDS-V3-plasma-45_Cluster_4046_sequences=148	282	148
SDS-V3-plasma-46_Cluster_869_sequences=148	286	148
SDS-V3-plasma-67_Cluster_627_sequences=148	504	148
SDS-V3-plasma-67_Cluster_1619_sequences=148	504	148
SDS-V3-plasma-0_Cluster_999_sequences=147	0	147
SDS-V3-plasma-24_Cluster_315_sequences=147	124	147
SDS-V3-plasma-45_Cluster_2544_sequences=147	282	147
SDS-V3-plasma-45_Cluster_669_sequences=147	282	147
SDS-V3-plasma-45_Cluster_2895_sequences=147	282	147
SDS-V3-plasma-46_Cluster_954_sequences=147	286	147
SDS-V3-plasma-46_Cluster_264_sequences=147	286	147
SDS-V3-plasma-0_Cluster_516_sequences=146	0	146
SDS-V3-plasma-0_Cluster_328_sequences=146	0	146
SDS-V3-plasma-24_Cluster_591_sequences=146	124	146
SDS-V3-plasma-45_Cluster_765_sequences=146	282	146
SDS-V3-plasma-67_Cluster_198_sequences=146	504	146
SDS-V3-plasma-67_Cluster_962_sequences=146	504	146
SDS-V3-plasma-67_Cluster_354_sequences=146	504	146
SDS-V3-plasma-45_Cluster_6462_sequences=145	282	145
SDS-V3-plasma-45_Cluster_1159_sequences=145	282	145
SDS-V3-plasma-45_Cluster_369_sequences=145	282	145
SDS-V3-plasma-46_Cluster_261_sequences=145	286	145
SDS-V3-plasma-67_Cluster_191_sequences=145	504	145
SDS-V3-plasma-67_Cluster_50_sequences=145	504	145
SDS-V3-plasma-67_Cluster_3956_sequences=145	504	145
SDS-V3-plasma-67_Cluster_190_sequences=145	504	145

SDS-V3-plasma-67_Cluster_71_sequences=145	504	145
SDS-V3-plasma-67_Cluster_4554_sequences=145	504	145
SDS-V3-plasma-0_Cluster_166_sequences=144	0	144
SDS-V3-plasma-0_Cluster_1020_sequences=144	0	144
SDS-V3-plasma-27_Cluster_27_sequences=144	131	144
SDS-V3-plasma-27_Cluster_150_sequences=144	131	144
SDS-V3-plasma-46_Cluster_213_sequences=144	286	144
SDS-V3-plasma-67_Cluster_3253_sequences=144	504	144
SDS-V3-plasma-67_Cluster_235_sequences=144	504	144
SDS-V3-plasma-67_Cluster_2854_sequences=144	504	144
SDS-V3-plasma-67_Cluster_1690_sequences=144	504	144
SDS-V3-plasma-67_Cluster_1728_sequences=144	504	144
SDS-V3-plasma-67_Cluster_40_sequences=144	504	144
SDS-V3-plasma-0_Cluster_2798_sequences=143	0	143
SDS-V3-plasma-24_Cluster_245_sequences=143	124	143
SDS-V3-plasma-27_Cluster_381_sequences=143	131	143
SDS-V3-plasma-45_Cluster_1543_sequences=143	282	143
SDS-V3-plasma-45_Cluster_893_sequences=143	282	143
SDS-V3-plasma-45_Cluster_3361_sequences=143	282	143
SDS-V3-plasma-45_Cluster_2197_sequences=143	282	143
SDS-V3-plasma-67_Cluster_1369_sequences=143	504	143
SDS-V3-plasma-67_Cluster_3732_sequences=143	504	143
SDS-V3-plasma-67_Cluster_7871_sequences=143	504	143
SDS-V3-plasma-67_Cluster_97_sequences=143	504	143
SDS-V3-plasma-24_Cluster_582_sequences=142	124	142
SDS-V3-plasma-24_Cluster_126_sequences=142	124	142
SDS-V3-plasma-24_Cluster_556_sequences=142	124	142
SDS-V3-plasma-45_Cluster_936_sequences=142	282	142
SDS-V3-plasma-45_Cluster_676_sequences=142	282	142
SDS-V3-plasma-67_Cluster_1385_sequences=142	504	142
SDS-V3-plasma-67_Cluster_2040_sequences=142	504	142
SDS-V3-plasma-67_Cluster_3173_sequences=142	504	142
SDS-V3-plasma-0_Cluster_129_sequences=141	0	141
SDS-V3-plasma-0_Cluster_436_sequences=141	0	141
SDS-V3-plasma-24_Cluster_44_sequences=141	124	141
SDS-V3-plasma-27_Cluster_237_sequences=141	131	141
SDS-V3-plasma-45_Cluster_2504_sequences=141	282	141
SDS-V3-plasma-45_Cluster_1562_sequences=141	282	141
SDS-V3-plasma-46_Cluster_2106_sequences=141	286	141
SDS-V3-plasma-67_Cluster_2299_sequences=141	504	141
SDS-V3-plasma-67_Cluster_2791_sequences=141	504	141
SDS-V3-plasma-67_Cluster_3144_sequences=141	504	141
SDS-V3-plasma-67_Cluster_2763_sequences=141	504	141
SDS-V3-plasma-67_Cluster_490_sequences=141	504	141
SDS-V3-plasma-67_Cluster_2075_sequences=141	504	141

SDS-V3-plasma-7_Cluster_134_sequences=140	14	140
SDS-V3-plasma-45_Cluster_3885_sequences=140	282	140
SDS-V3-plasma-45_Cluster_4914_sequences=140	282	140
SDS-V3-plasma-45_Cluster_1797_sequences=140	282	140
SDS-V3-plasma-45_Cluster_1870_sequences=140	282	140
SDS-V3-plasma-45_Cluster_3237_sequences=140	282	140
SDS-V3-plasma-45_Cluster_4127_sequences=140	282	140
SDS-V3-plasma-67_Cluster_1631_sequences=140	504	140
SDS-V3-plasma-67_Cluster_316_sequences=140	504	140
SDS-V3-plasma-67_Cluster_514_sequences=140	504	140
SDS-V3-plasma-67_Cluster_6377_sequences=140	504	140
SDS-V3-plasma-67_Cluster_4993_sequences=140	504	140
SDS-V3-plasma-67_Cluster_724_sequences=140	504	140
SDS-V3-plasma-67_Cluster_1701_sequences=140	504	140
SDS-V3-plasma-0_Cluster_241_sequences=139	0	139
SDS-V3-plasma-8_Cluster_92_sequences=139	16	139
SDS-V3-plasma-24_Cluster_184_sequences=139	124	139
SDS-V3-plasma-24_Cluster_459_sequences=139	124	139
SDS-V3-plasma-45_Cluster_532_sequences=139	282	139
SDS-V3-plasma-45_Cluster_4449_sequences=139	282	139
SDS-V3-plasma-45_Cluster_3501_sequences=139	282	139
SDS-V3-plasma-45_Cluster_3983_sequences=139	282	139
SDS-V3-plasma-67_Cluster_8892_sequences=139	504	139
SDS-V3-plasma-67_Cluster_572_sequences=139	504	139
SDS-V3-plasma-8_Cluster_2443_sequences=138	16	138
SDS-V3-plasma-27_Cluster_98_sequences=138	131	138
SDS-V3-plasma-45_Cluster_1440_sequences=138	282	138
SDS-V3-plasma-46_Cluster_1523_sequences=138	286	138
SDS-V3-plasma-8_Cluster_15_sequences=137	16	137
SDS-V3-plasma-46_Cluster_792_sequences=137	286	137
SDS-V3-plasma-46_Cluster_2674_sequences=137	286	137
SDS-V3-plasma-67_Cluster_6125_sequences=137	504	137
SDS-V3-plasma-67_Cluster_498_sequences=137	504	137
SDS-V3-plasma-27_Cluster_637_sequences=136	131	136
SDS-V3-plasma-27_Cluster_166_sequences=136	131	136
SDS-V3-plasma-45_Cluster_1363_sequences=136	282	136
SDS-V3-plasma-46_Cluster_128_sequences=136	286	136
SDS-V3-plasma-67_Cluster_856_sequences=136	504	136
SDS-V3-plasma-0_Cluster_1833_sequences=135	0	135
SDS-V3-plasma-24_Cluster_473_sequences=135	124	135
SDS-V3-plasma-24_Cluster_833_sequences=135	124	135
SDS-V3-plasma-24_Cluster_64_sequences=135	124	135
SDS-V3-plasma-45_Cluster_1486_sequences=135	282	135
SDS-V3-plasma-45_Cluster_1669_sequences=135	282	135
SDS-V3-plasma-45_Cluster_696_sequences=135	282	135

SDS-V3-plasma-67_Cluster_799_sequences=135	504	135
SDS-V3-plasma-67_Cluster_1224_sequences=135	504	135
SDS-V3-plasma-67_Cluster_196_sequences=135	504	135
SDS-V3-plasma-67_Cluster_503_sequences=135	504	135
SDS-V3-plasma-0_Cluster_1453_sequences=134	0	134
SDS-V3-plasma-7_Cluster_466_sequences=134	14	134
SDS-V3-plasma-8_Cluster_2582_sequences=134	16	134
SDS-V3-plasma-24_Cluster_192_sequences=134	124	134
SDS-V3-plasma-24_Cluster_70_sequences=134	124	134
SDS-V3-plasma-27_Cluster_182_sequences=134	131	134
SDS-V3-plasma-45_Cluster_952_sequences=134	282	134
SDS-V3-plasma-45_Cluster_1023_sequences=134	282	134
SDS-V3-plasma-45_Cluster_1660_sequences=134	282	134
SDS-V3-plasma-67_Cluster_3533_sequences=134	504	134
SDS-V3-plasma-67_Cluster_1727_sequences=134	504	134
SDS-V3-plasma-0_Cluster_289_sequences=133	0	133
SDS-V3-plasma-24_Cluster_291_sequences=133	124	133
SDS-V3-plasma-24_Cluster_37_sequences=133	124	133
SDS-V3-plasma-27_Cluster_8_sequences=133	131	133
SDS-V3-plasma-45_Cluster_2175_sequences=133	282	133
SDS-V3-plasma-45_Cluster_3338_sequences=133	282	133
SDS-V3-plasma-67_Cluster_101_sequences=133	504	133
SDS-V3-plasma-67_Cluster_1596_sequences=133	504	133
SDS-V3-plasma-67_Cluster_2326_sequences=133	504	133
SDS-V3-plasma-0_Cluster_383_sequences=132	0	132
SDS-V3-plasma-27_Cluster_317_sequences=132	131	132
SDS-V3-plasma-27_Cluster_738_sequences=132	131	132
SDS-V3-plasma-45_Cluster_825_sequences=132	282	132
SDS-V3-plasma-46_Cluster_632_sequences=132	286	132
SDS-V3-plasma-67_Cluster_2248_sequences=132	504	132
SDS-V3-plasma-67_Cluster_1402_sequences=132	504	132
SDS-V3-plasma-67_Cluster_2588_sequences=132	504	132
SDS-V3-plasma-67_Cluster_1_sequences=132	504	132
SDS-V3-plasma-0_Cluster_1827_sequences=131	0	131
SDS-V3-plasma-27_Cluster_594_sequences=131	131	131
SDS-V3-plasma-27_Cluster_82_sequences=131	131	131
SDS-V3-plasma-27_Cluster_153_sequences=131	131	131
SDS-V3-plasma-45_Cluster_312_sequences=131	282	131
SDS-V3-plasma-45_Cluster_501_sequences=131	282	131
SDS-V3-plasma-45_Cluster_1730_sequences=131	282	131
SDS-V3-plasma-45_Cluster_3618_sequences=131	282	131
SDS-V3-plasma-24_Cluster_116_sequences=130	124	130
SDS-V3-plasma-45_Cluster_478_sequences=130	282	130
SDS-V3-plasma-67_Cluster_1642_sequences=130	504	130
SDS-V3-plasma-67_Cluster_1712_sequences=130	504	130

SDS-V3-plasma-67_Cluster_464_sequences=130	504	130
SDS-V3-plasma-67_Cluster_3714_sequences=130	504	130
SDS-V3-plasma-67_Cluster_2781_sequences=130	504	130
SDS-V3-plasma-0_Cluster_674_sequences=129	0	129
SDS-V3-plasma-8_Cluster_2546_sequences=129	16	129
SDS-V3-plasma-24_Cluster_521_sequences=129	124	129
SDS-V3-plasma-45_Cluster_1043_sequences=129	282	129
SDS-V3-plasma-45_Cluster_972_sequences=129	282	129
SDS-V3-plasma-45_Cluster_1593_sequences=129	282	129
SDS-V3-plasma-46_Cluster_202_sequences=129	286	129
SDS-V3-plasma-67_Cluster_1430_sequences=129	504	129
SDS-V3-plasma-67_Cluster_3126_sequences=129	504	129
SDS-V3-plasma-67_Cluster_4212_sequences=129	504	129
SDS-V3-plasma-45_Cluster_4843_sequences=128	282	128
SDS-V3-plasma-45_Cluster_614_sequences=128	282	128
SDS-V3-plasma-45_Cluster_2418_sequences=128	282	128
SDS-V3-plasma-45_Cluster_3894_sequences=128	282	128
SDS-V3-plasma-45_Cluster_360_sequences=128	282	128
SDS-V3-plasma-45_Cluster_3153_sequences=128	282	128
SDS-V3-plasma-46_Cluster_98_sequences=128	286	128
SDS-V3-plasma-46_Cluster_579_sequences=128	286	128
SDS-V3-plasma-67_Cluster_444_sequences=128	504	128
SDS-V3-plasma-67_Cluster_1144_sequences=128	504	128
SDS-V3-plasma-67_Cluster_126_sequences=128	504	128
SDS-V3-plasma-67_Cluster_1549_sequences=128	504	128
SDS-V3-plasma-67_Cluster_486_sequences=128	504	128
SDS-V3-plasma-67_Cluster_53_sequences=128	504	128
SDS-V3-plasma-67_Cluster_691_sequences=128	504	128
SDS-V3-plasma-67_Cluster_1280_sequences=128	504	128
SDS-V3-plasma-0_Cluster_1296_sequences=127	0	127
SDS-V3-plasma-8_Cluster_3002_sequences=127	16	127
SDS-V3-plasma-24_Cluster_76_sequences=127	124	127
SDS-V3-plasma-24_Cluster_691_sequences=127	124	127
SDS-V3-plasma-24_Cluster_160_sequences=127	124	127
SDS-V3-plasma-45_Cluster_505_sequences=127	282	127
SDS-V3-plasma-45_Cluster_1132_sequences=127	282	127
SDS-V3-plasma-67_Cluster_687_sequences=127	504	127
SDS-V3-plasma-67_Cluster_804_sequences=127	504	127
SDS-V3-plasma-67_Cluster_837_sequences=127	504	127
SDS-V3-plasma-0_Cluster_650_sequences=126	0	126
SDS-V3-plasma-7_Cluster_100_sequences=126	14	126
SDS-V3-plasma-7_Cluster_79_sequences=126	14	126
SDS-V3-plasma-7_Cluster_422_sequences=126	14	126
SDS-V3-plasma-24_Cluster_559_sequences=126	124	126
SDS-V3-plasma-24_Cluster_486_sequences=126	124	126

SDS-V3-plasma-45_Cluster_898_sequences=126	282	126
SDS-V3-plasma-45_Cluster_1623_sequences=126	282	126
SDS-V3-plasma-46_Cluster_1016_sequences=126	286	126
SDS-V3-plasma-67_Cluster_1029_sequences=126	504	126
SDS-V3-plasma-67_Cluster_1416_sequences=126	504	126
SDS-V3-plasma-67_Cluster_303_sequences=126	504	126
SDS-V3-plasma-67_Cluster_3606_sequences=126	504	126
SDS-V3-plasma-67_Cluster_328_sequences=126	504	126
SDS-V3-plasma-7_Cluster_250_sequences=125	14	125
SDS-V3-plasma-24_Cluster_15_sequences=125	124	125
SDS-V3-plasma-27_Cluster_1470_sequences=125	131	125
SDS-V3-plasma-27_Cluster_345_sequences=125	131	125
SDS-V3-plasma-45_Cluster_1239_sequences=125	282	125
SDS-V3-plasma-45_Cluster_6422_sequences=125	282	125
SDS-V3-plasma-45_Cluster_1976_sequences=125	282	125
SDS-V3-plasma-45_Cluster_2795_sequences=125	282	125
SDS-V3-plasma-45_Cluster_1876_sequences=125	282	125
SDS-V3-plasma-45_Cluster_357_sequences=125	282	125
SDS-V3-plasma-45_Cluster_396_sequences=125	282	125
SDS-V3-plasma-67_Cluster_1575_sequences=125	504	125
SDS-V3-plasma-67_Cluster_3057_sequences=125	504	125
SDS-V3-plasma-67_Cluster_777_sequences=125	504	125
SDS-V3-plasma-0_Cluster_913_sequences=124	0	124
SDS-V3-plasma-0_Cluster_456_sequences=124	0	124
SDS-V3-plasma-8_Cluster_2630_sequences=124	16	124
SDS-V3-plasma-27_Cluster_28_sequences=124	131	124
SDS-V3-plasma-45_Cluster_1372_sequences=124	282	124
SDS-V3-plasma-45_Cluster_622_sequences=124	282	124
SDS-V3-plasma-45_Cluster_480_sequences=124	282	124
SDS-V3-plasma-45_Cluster_2542_sequences=124	282	124
SDS-V3-plasma-67_Cluster_1167_sequences=124	504	124
SDS-V3-plasma-67_Cluster_2048_sequences=124	504	124
SDS-V3-plasma-67_Cluster_2562_sequences=124	504	124
SDS-V3-plasma-0_Cluster_1587_sequences=123	0	123
SDS-V3-plasma-0_Cluster_619_sequences=123	0	123
SDS-V3-plasma-24_Cluster_516_sequences=123	124	123
SDS-V3-plasma-24_Cluster_174_sequences=123	124	123
SDS-V3-plasma-27_Cluster_435_sequences=123	131	123
SDS-V3-plasma-45_Cluster_310_sequences=123	282	123
SDS-V3-plasma-45_Cluster_412_sequences=123	282	123
SDS-V3-plasma-46_Cluster_3214_sequences=123	286	123
SDS-V3-plasma-46_Cluster_145_sequences=123	286	123
SDS-V3-plasma-67_Cluster_2553_sequences=123	504	123
SDS-V3-plasma-67_Cluster_6280_sequences=123	504	123
SDS-V3-plasma-67_Cluster_1035_sequences=123	504	123

SDS-V3-plasma-67_Cluster_1759_sequences=123	504	123
SDS-V3-plasma-67_Cluster_2453_sequences=123	504	123
SDS-V3-plasma-0_Cluster_1203_sequences=122	0	122
SDS-V3-plasma-7_Cluster_108_sequences=122	14	122
SDS-V3-plasma-24_Cluster_1094_sequences=122	124	122
SDS-V3-plasma-27_Cluster_136_sequences=122	131	122
SDS-V3-plasma-45_Cluster_2451_sequences=122	282	122
SDS-V3-plasma-45_Cluster_4539_sequences=122	282	122
SDS-V3-plasma-45_Cluster_997_sequences=122	282	122
SDS-V3-plasma-45_Cluster_1815_sequences=122	282	122
SDS-V3-plasma-45_Cluster_882_sequences=122	282	122
SDS-V3-plasma-67_Cluster_260_sequences=122	504	122
SDS-V3-plasma-67_Cluster_2954_sequences=122	504	122
SDS-V3-plasma-67_Cluster_4432_sequences=122	504	122
SDS-V3-plasma-67_Cluster_2703_sequences=122	504	122
SDS-V3-plasma-67_Cluster_1347_sequences=122	504	122
SDS-V3-plasma-67_Cluster_3212_sequences=122	504	122
SDS-V3-plasma-67_Cluster_1284_sequences=122	504	122
SDS-V3-plasma-0_Cluster_4386_sequences=121	0	121
SDS-V3-plasma-24_Cluster_370_sequences=121	124	121
SDS-V3-plasma-27_Cluster_147_sequences=121	131	121
SDS-V3-plasma-45_Cluster_1452_sequences=121	282	121
SDS-V3-plasma-45_Cluster_1055_sequences=121	282	121
SDS-V3-plasma-45_Cluster_848_sequences=121	282	121
SDS-V3-plasma-67_Cluster_1149_sequences=121	504	121
SDS-V3-plasma-67_Cluster_5433_sequences=121	504	121
SDS-V3-plasma-67_Cluster_1997_sequences=121	504	121
SDS-V3-plasma-27_Cluster_335_sequences=120	131	120
SDS-V3-plasma-45_Cluster_698_sequences=120	282	120
SDS-V3-plasma-45_Cluster_587_sequences=120	282	120
SDS-V3-plasma-45_Cluster_3198_sequences=120	282	120
SDS-V3-plasma-45_Cluster_1913_sequences=120	282	120
SDS-V3-plasma-45_Cluster_3820_sequences=120	282	120
SDS-V3-plasma-46_Cluster_427_sequences=120	286	120
SDS-V3-plasma-67_Cluster_2756_sequences=120	504	120
SDS-V3-plasma-67_Cluster_554_sequences=120	504	120
SDS-V3-plasma-67_Cluster_1655_sequences=120	504	120
SDS-V3-plasma-24_Cluster_546_sequences=119	124	119
SDS-V3-plasma-24_Cluster_63_sequences=119	124	119
SDS-V3-plasma-27_Cluster_143_sequences=119	131	119
SDS-V3-plasma-27_Cluster_149_sequences=119	131	119
SDS-V3-plasma-45_Cluster_695_sequences=119	282	119
SDS-V3-plasma-67_Cluster_4149_sequences=119	504	119
SDS-V3-plasma-0_Cluster_637_sequences=118	0	118
SDS-V3-plasma-0_Cluster_1030_sequences=118	0	118

SDS-V3-plasma-7_Cluster_55_sequences=118	14	118
SDS-V3-plasma-7_Cluster_314_sequences=118	14	118
SDS-V3-plasma-45_Cluster_1459_sequences=118	282	118
SDS-V3-plasma-45_Cluster_1002_sequences=118	282	118
SDS-V3-plasma-45_Cluster_1419_sequences=118	282	118
SDS-V3-plasma-67_Cluster_1218_sequences=118	504	118
SDS-V3-plasma-67_Cluster_96_sequences=118	504	118
SDS-V3-plasma-27_Cluster_478_sequences=117	131	117
SDS-V3-plasma-27_Cluster_360_sequences=117	131	117
SDS-V3-plasma-45_Cluster_356_sequences=117	282	117
SDS-V3-plasma-45_Cluster_569_sequences=117	282	117
SDS-V3-plasma-45_Cluster_2079_sequences=117	282	117
SDS-V3-plasma-45_Cluster_482_sequences=117	282	117
SDS-V3-plasma-46_Cluster_1111_sequences=117	286	117
SDS-V3-plasma-46_Cluster_377_sequences=117	286	117
SDS-V3-plasma-67_Cluster_831_sequences=117	504	117
SDS-V3-plasma-0_Cluster_880_sequences=116	0	116
SDS-V3-plasma-24_Cluster_444_sequences=116	124	116
SDS-V3-plasma-27_Cluster_163_sequences=116	131	116
SDS-V3-plasma-27_Cluster_63_sequences=116	131	116
SDS-V3-plasma-27_Cluster_884_sequences=116	131	116
SDS-V3-plasma-27_Cluster_222_sequences=116	131	116
SDS-V3-plasma-45_Cluster_5555_sequences=116	282	116
SDS-V3-plasma-45_Cluster_926_sequences=116	282	116
SDS-V3-plasma-45_Cluster_490_sequences=116	282	116
SDS-V3-plasma-67_Cluster_1827_sequences=116	504	116
SDS-V3-plasma-67_Cluster_1408_sequences=116	504	116
SDS-V3-plasma-67_Cluster_174_sequences=116	504	116
SDS-V3-plasma-67_Cluster_2056_sequences=116	504	116
SDS-V3-plasma-67_Cluster_617_sequences=116	504	116
SDS-V3-plasma-67_Cluster_2689_sequences=116	504	116
SDS-V3-plasma-67_Cluster_3751_sequences=116	504	116
SDS-V3-plasma-0_Cluster_2099_sequences=115	0	115
SDS-V3-plasma-27_Cluster_449_sequences=115	131	115
SDS-V3-plasma-27_Cluster_262_sequences=115	131	115
SDS-V3-plasma-45_Cluster_3397_sequences=115	282	115
SDS-V3-plasma-45_Cluster_735_sequences=115	282	115
SDS-V3-plasma-45_Cluster_293_sequences=115	282	115
SDS-V3-plasma-45_Cluster_1804_sequences=115	282	115
SDS-V3-plasma-45_Cluster_7549_sequences=115	282	115
SDS-V3-plasma-67_Cluster_5415_sequences=115	504	115
SDS-V3-plasma-67_Cluster_8859_sequences=115	504	115
SDS-V3-plasma-67_Cluster_3028_sequences=115	504	115
SDS-V3-plasma-67_Cluster_2783_sequences=115	504	115
SDS-V3-plasma-0_Cluster_2298_sequences=114	0	114

SDS-V3-plasma-0_Cluster_322_sequences=114	0	114
SDS-V3-plasma-45_Cluster_776_sequences=114	282	114
SDS-V3-plasma-45_Cluster_6470_sequences=114	282	114
SDS-V3-plasma-46_Cluster_51_sequences=114	286	114
SDS-V3-plasma-67_Cluster_1543_sequences=114	504	114
SDS-V3-plasma-67_Cluster_4088_sequences=114	504	114
SDS-V3-plasma-67_Cluster_1770_sequences=114	504	114
SDS-V3-plasma-67_Cluster_6752_sequences=114	504	114
SDS-V3-plasma-67_Cluster_92_sequences=114	504	114
SDS-V3-plasma-67_Cluster_2914_sequences=114	504	114
SDS-V3-plasma-67_Cluster_331_sequences=114	504	114
SDS-V3-plasma-67_Cluster_4712_sequences=114	504	114
SDS-V3-plasma-0_Cluster_705_sequences=113	0	113
SDS-V3-plasma-8_Cluster_2492_sequences=113	16	113
SDS-V3-plasma-24_Cluster_1388_sequences=113	124	113
SDS-V3-plasma-45_Cluster_2087_sequences=113	282	113
SDS-V3-plasma-45_Cluster_809_sequences=113	282	113
SDS-V3-plasma-45_Cluster_1578_sequences=113	282	113
SDS-V3-plasma-46_Cluster_243_sequences=113	286	113
SDS-V3-plasma-46_Cluster_2956_sequences=113	286	113
SDS-V3-plasma-0_Cluster_2330_sequences=112	0	112
SDS-V3-plasma-0_Cluster_569_sequences=112	0	112
SDS-V3-plasma-0_Cluster_1670_sequences=112	0	112
SDS-V3-plasma-27_Cluster_847_sequences=112	131	112
SDS-V3-plasma-45_Cluster_3381_sequences=112	282	112
SDS-V3-plasma-45_Cluster_79_sequences=112	282	112
SDS-V3-plasma-45_Cluster_5245_sequences=112	282	112
SDS-V3-plasma-45_Cluster_5613_sequences=112	282	112
SDS-V3-plasma-45_Cluster_3853_sequences=112	282	112
SDS-V3-plasma-67_Cluster_4701_sequences=112	504	112
SDS-V3-plasma-67_Cluster_3048_sequences=112	504	112
SDS-V3-plasma-67_Cluster_2543_sequences=112	504	112
SDS-V3-plasma-67_Cluster_3554_sequences=112	504	112
SDS-V3-plasma-67_Cluster_5670_sequences=112	504	112
SDS-V3-plasma-67_Cluster_697_sequences=112	504	112
SDS-V3-plasma-67_Cluster_1510_sequences=112	504	112
SDS-V3-plasma-67_Cluster_981_sequences=112	504	112
SDS-V3-plasma-67_Cluster_1898_sequences=112	504	112
SDS-V3-plasma-7_Cluster_432_sequences=111	14	111
SDS-V3-plasma-8_Cluster_2393_sequences=111	16	111
SDS-V3-plasma-24_Cluster_92_sequences=111	124	111
SDS-V3-plasma-24_Cluster_515_sequences=111	124	111
SDS-V3-plasma-24_Cluster_225_sequences=111	124	111
SDS-V3-plasma-27_Cluster_401_sequences=111	131	111
SDS-V3-plasma-45_Cluster_599_sequences=111	282	111

SDS-V3-plasma-45_Cluster_6114_sequences=111	282	111
SDS-V3-plasma-46_Cluster_123_sequences=111	286	111
SDS-V3-plasma-46_Cluster_412_sequences=111	286	111
SDS-V3-plasma-46_Cluster_663_sequences=111	286	111
SDS-V3-plasma-67_Cluster_1110_sequences=111	504	111
SDS-V3-plasma-67_Cluster_1624_sequences=111	504	111
SDS-V3-plasma-67_Cluster_2645_sequences=111	504	111
SDS-V3-plasma-67_Cluster_793_sequences=111	504	111
SDS-V3-plasma-67_Cluster_5728_sequences=111	504	111
SDS-V3-plasma-7_Cluster_78_sequences=110	14	110
SDS-V3-plasma-24_Cluster_1134_sequences=110	124	110
SDS-V3-plasma-24_Cluster_581_sequences=110	124	110
SDS-V3-plasma-45_Cluster_636_sequences=110	282	110
SDS-V3-plasma-45_Cluster_2896_sequences=110	282	110
SDS-V3-plasma-45_Cluster_4494_sequences=110	282	110
SDS-V3-plasma-45_Cluster_1497_sequences=110	282	110
SDS-V3-plasma-45_Cluster_2298_sequences=110	282	110
SDS-V3-plasma-45_Cluster_8739_sequences=110	282	110
SDS-V3-plasma-46_Cluster_1053_sequences=110	286	110
SDS-V3-plasma-46_Cluster_598_sequences=110	286	110
SDS-V3-plasma-67_Cluster_1955_sequences=110	504	110
SDS-V3-plasma-67_Cluster_2046_sequences=110	504	110
SDS-V3-plasma-67_Cluster_4615_sequences=110	504	110
SDS-V3-plasma-67_Cluster_4836_sequences=110	504	110
SDS-V3-plasma-67_Cluster_5193_sequences=110	504	110
SDS-V3-plasma-67_Cluster_5343_sequences=110	504	110
SDS-V3-plasma-67_Cluster_2828_sequences=110	504	110
SDS-V3-plasma-24_Cluster_506_sequences=109	124	109
SDS-V3-plasma-27_Cluster_801_sequences=109	131	109
SDS-V3-plasma-45_Cluster_7606_sequences=109	282	109
SDS-V3-plasma-45_Cluster_1786_sequences=109	282	109
SDS-V3-plasma-45_Cluster_564_sequences=109	282	109
SDS-V3-plasma-45_Cluster_3350_sequences=109	282	109
SDS-V3-plasma-67_Cluster_291_sequences=109	504	109
SDS-V3-plasma-67_Cluster_2318_sequences=109	504	109
SDS-V3-plasma-67_Cluster_872_sequences=109	504	109
SDS-V3-plasma-0_Cluster_1346_sequences=108	0	108
SDS-V3-plasma-7_Cluster_486_sequences=108	14	108
SDS-V3-plasma-24_Cluster_81_sequences=108	124	108
SDS-V3-plasma-45_Cluster_2778_sequences=108	282	108
SDS-V3-plasma-45_Cluster_2403_sequences=108	282	108
SDS-V3-plasma-45_Cluster_2044_sequences=108	282	108
SDS-V3-plasma-46_Cluster_825_sequences=108	286	108
SDS-V3-plasma-46_Cluster_321_sequences=108	286	108
SDS-V3-plasma-46_Cluster_22_sequences=108	286	108

SDS-V3-plasma-45_Cluster_10907_sequences=107	282	107
SDS-V3-plasma-45_Cluster_2015_sequences=107	282	107
SDS-V3-plasma-45_Cluster_81_sequences=107	282	107
SDS-V3-plasma-45_Cluster_789_sequences=107	282	107
SDS-V3-plasma-67_Cluster_598_sequences=107	504	107
SDS-V3-plasma-67_Cluster_3868_sequences=107	504	107
SDS-V3-plasma-67_Cluster_1053_sequences=107	504	107
SDS-V3-plasma-67_Cluster_2528_sequences=107	504	107
SDS-V3-plasma-67_Cluster_1570_sequences=107	504	107
SDS-V3-plasma-24_Cluster_207_sequences=106	124	106
SDS-V3-plasma-27_Cluster_455_sequences=106	131	106
SDS-V3-plasma-27_Cluster_912_sequences=106	131	106
SDS-V3-plasma-45_Cluster_877_sequences=106	282	106
SDS-V3-plasma-45_Cluster_1195_sequences=106	282	106
SDS-V3-plasma-45_Cluster_65686_sequences=106	282	106
SDS-V3-plasma-45_Cluster_3695_sequences=106	282	106
SDS-V3-plasma-45_Cluster_1199_sequences=106	282	106
SDS-V3-plasma-46_Cluster_2197_sequences=106	286	106
SDS-V3-plasma-46_Cluster_936_sequences=106	286	106
SDS-V3-plasma-67_Cluster_1689_sequences=106	504	106
SDS-V3-plasma-67_Cluster_2803_sequences=106	504	106
SDS-V3-plasma-67_Cluster_597_sequences=106	504	106
SDS-V3-plasma-0_Cluster_46_sequences=105	0	105
SDS-V3-plasma-7_Cluster_163_sequences=105	14	105
SDS-V3-plasma-27_Cluster_26_sequences=105	131	105
SDS-V3-plasma-45_Cluster_4674_sequences=105	282	105
SDS-V3-plasma-45_Cluster_864_sequences=105	282	105
SDS-V3-plasma-45_Cluster_2129_sequences=105	282	105
SDS-V3-plasma-45_Cluster_3523_sequences=105	282	105
SDS-V3-plasma-46_Cluster_837_sequences=105	286	105
SDS-V3-plasma-67_Cluster_2117_sequences=105	504	105
SDS-V3-plasma-67_Cluster_1816_sequences=105	504	105
SDS-V3-plasma-67_Cluster_998_sequences=105	504	105
SDS-V3-plasma-67_Cluster_1374_sequences=105	504	105
SDS-V3-plasma-8_Cluster_75_sequences=104	16	104
SDS-V3-plasma-8_Cluster_2409_sequences=104	16	104
SDS-V3-plasma-24_Cluster_24_sequences=104	124	104
SDS-V3-plasma-24_Cluster_934_sequences=104	124	104
SDS-V3-plasma-27_Cluster_474_sequences=104	131	104
SDS-V3-plasma-27_Cluster_283_sequences=104	131	104
SDS-V3-plasma-45_Cluster_1320_sequences=104	282	104
SDS-V3-plasma-46_Cluster_645_sequences=104	286	104
SDS-V3-plasma-67_Cluster_3226_sequences=104	504	104
SDS-V3-plasma-67_Cluster_869_sequences=104	504	104
SDS-V3-plasma-0_Cluster_96_sequences=103	0	103

SDS-V3-plasma-0_Cluster_421_sequences=103	0	103
SDS-V3-plasma-0_Cluster_27_sequences=103	0	103
SDS-V3-plasma-7_Cluster_413_sequences=103	14	103
SDS-V3-plasma-7_Cluster_381_sequences=103	14	103
SDS-V3-plasma-24_Cluster_970_sequences=103	124	103
SDS-V3-plasma-24_Cluster_118_sequences=103	124	103
SDS-V3-plasma-24_Cluster_368_sequences=103	124	103
SDS-V3-plasma-24_Cluster_156_sequences=103	124	103
SDS-V3-plasma-27_Cluster_64_sequences=103	131	103
SDS-V3-plasma-45_Cluster_6281_sequences=103	282	103
SDS-V3-plasma-45_Cluster_1181_sequences=103	282	103
SDS-V3-plasma-45_Cluster_4663_sequences=103	282	103
SDS-V3-plasma-67_Cluster_1693_sequences=103	504	103
SDS-V3-plasma-67_Cluster_1556_sequences=103	504	103
SDS-V3-plasma-67_Cluster_2295_sequences=103	504	103
SDS-V3-plasma-67_Cluster_255_sequences=103	504	103
SDS-V3-plasma-0_Cluster_496_sequences=102	0	102
SDS-V3-plasma-0_Cluster_700_sequences=102	0	102
SDS-V3-plasma-8_Cluster_2637_sequences=102	16	102
SDS-V3-plasma-8_Cluster_2333_sequences=102	16	102
SDS-V3-plasma-24_Cluster_69_sequences=102	124	102
SDS-V3-plasma-45_Cluster_1131_sequences=102	282	102
SDS-V3-plasma-45_Cluster_4066_sequences=102	282	102
SDS-V3-plasma-45_Cluster_1909_sequences=102	282	102
SDS-V3-plasma-46_Cluster_1590_sequences=102	286	102
SDS-V3-plasma-67_Cluster_2980_sequences=102	504	102
SDS-V3-plasma-67_Cluster_1463_sequences=102	504	102
SDS-V3-plasma-67_Cluster_3688_sequences=102	504	102
SDS-V3-plasma-67_Cluster_4536_sequences=102	504	102
SDS-V3-plasma-0_Cluster_94_sequences=101	0	101
SDS-V3-plasma-27_Cluster_575_sequences=101	131	101
SDS-V3-plasma-45_Cluster_4154_sequences=101	282	101
SDS-V3-plasma-45_Cluster_3614_sequences=101	282	101
SDS-V3-plasma-45_Cluster_1074_sequences=101	282	101
SDS-V3-plasma-45_Cluster_2328_sequences=101	282	101
SDS-V3-plasma-45_Cluster_4219_sequences=101	282	101
SDS-V3-plasma-45_Cluster_420_sequences=101	282	101
SDS-V3-plasma-45_Cluster_3224_sequences=101	282	101
SDS-V3-plasma-45_Cluster_711_sequences=101	282	101
SDS-V3-plasma-67_Cluster_2708_sequences=101	504	101
SDS-V3-plasma-67_Cluster_6929_sequences=101	504	101
SDS-V3-plasma-0_Cluster_1549_sequences=100	0	100
SDS-V3-plasma-8_Cluster_3287_sequences=100	16	100
SDS-V3-plasma-24_Cluster_158_sequences=100	124	100
SDS-V3-plasma-24_Cluster_387_sequences=100	124	100

SDS-V3-plasma-27_Cluster_436_sequences=100	131	100
SDS-V3-plasma-45_Cluster_837_sequences=100	282	100
SDS-V3-plasma-45_Cluster_856_sequences=100	282	100
SDS-V3-plasma-45_Cluster_2624_sequences=100	282	100
SDS-V3-plasma-45_Cluster_758_sequences=100	282	100
SDS-V3-plasma-46_Cluster_612_sequences=100	286	100
SDS-V3-plasma-67_Cluster_3815_sequences=100	504	100
SDS-V3-plasma-67_Cluster_5232_sequences=100	504	100
SDS-V3-plasma-67_Cluster_1680_sequences=100	504	100
SDS-V3-plasma-67_Cluster_2015_sequences=100	504	100
SDS-V3-plasma-67_Cluster_792_sequences=100	504	100
SDS-V3-plasma-67_Cluster_1228_sequences=100	504	100
SDS-V3-plasma-67_Cluster_1205_sequences=100	504	100
SDS-V3-plasma-67_Cluster_2780_sequences=100	504	100
SDS-V3-plasma-0_Cluster_2000_sequences=99	0	99
SDS-V3-plasma-0_Cluster_458_sequences=99	0	99
SDS-V3-plasma-7_Cluster_411_sequences=99	14	99
SDS-V3-plasma-8_Cluster_54_sequences=99	16	99
SDS-V3-plasma-8_Cluster_17_sequences=99	16	99
SDS-V3-plasma-8_Cluster_2815_sequences=99	16	99
SDS-V3-plasma-24_Cluster_407_sequences=99	124	99
SDS-V3-plasma-27_Cluster_533_sequences=99	131	99
SDS-V3-plasma-27_Cluster_344_sequences=99	131	99
SDS-V3-plasma-27_Cluster_402_sequences=99	131	99
SDS-V3-plasma-45_Cluster_7584_sequences=99	282	99
SDS-V3-plasma-45_Cluster_1821_sequences=99	282	99
SDS-V3-plasma-45_Cluster_6355_sequences=99	282	99
SDS-V3-plasma-46_Cluster_1684_sequences=99	286	99
SDS-V3-plasma-46_Cluster_2433_sequences=99	286	99
SDS-V3-plasma-67_Cluster_5533_sequences=99	504	99
SDS-V3-plasma-67_Cluster_1143_sequences=99	504	99
SDS-V3-plasma-67_Cluster_2000_sequences=99	504	99
SDS-V3-plasma-67_Cluster_1395_sequences=99	504	99
SDS-V3-plasma-0_Cluster_1341_sequences=98	0	98
SDS-V3-plasma-0_Cluster_1022_sequences=98	0	98
SDS-V3-plasma-8_Cluster_2358_sequences=98	16	98
SDS-V3-plasma-24_Cluster_840_sequences=98	124	98
SDS-V3-plasma-24_Cluster_414_sequences=98	124	98
SDS-V3-plasma-27_Cluster_496_sequences=98	131	98
SDS-V3-plasma-45_Cluster_2255_sequences=98	282	98
SDS-V3-plasma-45_Cluster_1157_sequences=98	282	98
SDS-V3-plasma-45_Cluster_4006_sequences=98	282	98
SDS-V3-plasma-67_Cluster_439_sequences=98	504	98
SDS-V3-plasma-67_Cluster_15798_sequences=98	504	98
SDS-V3-plasma-67_Cluster_1836_sequences=98	504	98

SDS-V3-plasma-67_Cluster_2546_sequences=98	504	98
SDS-V3-plasma-67_Cluster_2939_sequences=98	504	98
SDS-V3-plasma-67_Cluster_5987_sequences=98	504	98
SDS-V3-plasma-67_Cluster_4464_sequences=98	504	98
SDS-V3-plasma-67_Cluster_2794_sequences=98	504	98
SDS-V3-plasma-67_Cluster_2281_sequences=98	504	98
SDS-V3-plasma-0_Cluster_1666_sequences=97	0	97
SDS-V3-plasma-0_Cluster_295_sequences=97	0	97
SDS-V3-plasma-0_Cluster_796_sequences=97	0	97
SDS-V3-plasma-0_Cluster_2407_sequences=97	0	97
SDS-V3-plasma-0_Cluster_304_sequences=97	0	97
SDS-V3-plasma-24_Cluster_3736_sequences=97	124	97
SDS-V3-plasma-24_Cluster_224_sequences=97	124	97
SDS-V3-plasma-27_Cluster_704_sequences=97	131	97
SDS-V3-plasma-27_Cluster_145_sequences=97	131	97
SDS-V3-plasma-45_Cluster_7531_sequences=97	282	97
SDS-V3-plasma-45_Cluster_7870_sequences=97	282	97
SDS-V3-plasma-45_Cluster_2064_sequences=97	282	97
SDS-V3-plasma-45_Cluster_2956_sequences=97	282	97
SDS-V3-plasma-46_Cluster_1833_sequences=97	286	97
SDS-V3-plasma-46_Cluster_896_sequences=97	286	97
SDS-V3-plasma-67_Cluster_3209_sequences=97	504	97
SDS-V3-plasma-67_Cluster_210_sequences=97	504	97
SDS-V3-plasma-67_Cluster_199_sequences=97	504	97
SDS-V3-plasma-67_Cluster_926_sequences=97	504	97
SDS-V3-plasma-67_Cluster_1679_sequences=97	504	97
SDS-V3-plasma-67_Cluster_2883_sequences=97	504	97
SDS-V3-plasma-67_Cluster_2634_sequences=97	504	97
SDS-V3-plasma-24_Cluster_602_sequences=96	124	96
SDS-V3-plasma-27_Cluster_1654_sequences=96	131	96
SDS-V3-plasma-45_Cluster_492_sequences=96	282	96
SDS-V3-plasma-67_Cluster_646_sequences=96	504	96
SDS-V3-plasma-67_Cluster_4860_sequences=96	504	96
SDS-V3-plasma-67_Cluster_3430_sequences=96	504	96
SDS-V3-plasma-67_Cluster_304_sequences=96	504	96
SDS-V3-plasma-67_Cluster_6060_sequences=96	504	96
SDS-V3-plasma-67_Cluster_357_sequences=96	504	96
SDS-V3-plasma-67_Cluster_555_sequences=96	504	96
SDS-V3-plasma-0_Cluster_1150_sequences=95	0	95
SDS-V3-plasma-24_Cluster_441_sequences=95	124	95
SDS-V3-plasma-27_Cluster_75_sequences=95	131	95
SDS-V3-plasma-45_Cluster_3199_sequences=95	282	95
SDS-V3-plasma-45_Cluster_1443_sequences=95	282	95
SDS-V3-plasma-45_Cluster_3967_sequences=95	282	95
SDS-V3-plasma-45_Cluster_432_sequences=95	282	95

SDS-V3-plasma-45_Cluster_859_sequences=95	282	95
SDS-V3-plasma-45_Cluster_2276_sequences=95	282	95
SDS-V3-plasma-46_Cluster_178_sequences=95	286	95
SDS-V3-plasma-67_Cluster_1417_sequences=95	504	95
SDS-V3-plasma-67_Cluster_985_sequences=95	504	95
SDS-V3-plasma-67_Cluster_4158_sequences=95	504	95
SDS-V3-plasma-67_Cluster_3525_sequences=95	504	95
SDS-V3-plasma-8_Cluster_20_sequences=94	16	94
SDS-V3-plasma-27_Cluster_186_sequences=94	131	94
SDS-V3-plasma-27_Cluster_674_sequences=94	131	94
SDS-V3-plasma-45_Cluster_830_sequences=94	282	94
SDS-V3-plasma-45_Cluster_566_sequences=94	282	94
SDS-V3-plasma-45_Cluster_2867_sequences=94	282	94
SDS-V3-plasma-45_Cluster_1616_sequences=94	282	94
SDS-V3-plasma-45_Cluster_303_sequences=94	282	94
SDS-V3-plasma-45_Cluster_1194_sequences=94	282	94
SDS-V3-plasma-46_Cluster_1287_sequences=94	286	94
SDS-V3-plasma-67_Cluster_381_sequences=94	504	94
SDS-V3-plasma-67_Cluster_3431_sequences=94	504	94
SDS-V3-plasma-67_Cluster_5579_sequences=94	504	94
SDS-V3-plasma-67_Cluster_1234_sequences=94	504	94
SDS-V3-plasma-67_Cluster_9526_sequences=94	504	94
SDS-V3-plasma-67_Cluster_1495_sequences=94	504	94
SDS-V3-plasma-67_Cluster_4483_sequences=94	504	94
SDS-V3-plasma-67_Cluster_2745_sequences=94	504	94
SDS-V3-plasma-67_Cluster_441_sequences=94	504	94
SDS-V3-plasma-0_Cluster_1157_sequences=93	0	93
SDS-V3-plasma-0_Cluster_16910_sequences=93	0	93
SDS-V3-plasma-7_Cluster_182_sequences=93	14	93
SDS-V3-plasma-24_Cluster_776_sequences=93	124	93
SDS-V3-plasma-24_Cluster_12_sequences=93	124	93
SDS-V3-plasma-27_Cluster_331_sequences=93	131	93
SDS-V3-plasma-27_Cluster_414_sequences=93	131	93
SDS-V3-plasma-27_Cluster_950_sequences=93	131	93
SDS-V3-plasma-45_Cluster_2762_sequences=93	282	93
SDS-V3-plasma-45_Cluster_4738_sequences=93	282	93
SDS-V3-plasma-45_Cluster_1849_sequences=93	282	93
SDS-V3-plasma-45_Cluster_1003_sequences=93	282	93
SDS-V3-plasma-45_Cluster_6029_sequences=93	282	93
SDS-V3-plasma-45_Cluster_3917_sequences=93	282	93
SDS-V3-plasma-45_Cluster_2576_sequences=93	282	93
SDS-V3-plasma-45_Cluster_6031_sequences=93	282	93
SDS-V3-plasma-45_Cluster_1504_sequences=93	282	93
SDS-V3-plasma-45_Cluster_2906_sequences=93	282	93
SDS-V3-plasma-45_Cluster_451_sequences=93	282	93

SDS-V3-plasma-45_Cluster_3287_sequences=93	282	93
SDS-V3-plasma-46_Cluster_1451_sequences=93	286	93
SDS-V3-plasma-67_Cluster_2986_sequences=93	504	93
SDS-V3-plasma-67_Cluster_500_sequences=93	504	93
SDS-V3-plasma-67_Cluster_4792_sequences=93	504	93
SDS-V3-plasma-67_Cluster_5762_sequences=93	504	93
SDS-V3-plasma-67_Cluster_5577_sequences=93	504	93
SDS-V3-plasma-67_Cluster_4575_sequences=93	504	93
SDS-V3-plasma-67_Cluster_2874_sequences=93	504	93
SDS-V3-plasma-67_Cluster_1961_sequences=93	504	93
SDS-V3-plasma-0_Cluster_192_sequences=92	0	92
SDS-V3-plasma-0_Cluster_645_sequences=92	0	92
SDS-V3-plasma-0_Cluster_235_sequences=92	0	92
SDS-V3-plasma-24_Cluster_163_sequences=92	124	92
SDS-V3-plasma-27_Cluster_363_sequences=92	131	92
SDS-V3-plasma-45_Cluster_975_sequences=92	282	92
SDS-V3-plasma-45_Cluster_2117_sequences=92	282	92
SDS-V3-plasma-45_Cluster_1982_sequences=92	282	92
SDS-V3-plasma-45_Cluster_4767_sequences=92	282	92
SDS-V3-plasma-45_Cluster_5794_sequences=92	282	92
SDS-V3-plasma-45_Cluster_646_sequences=92	282	92
SDS-V3-plasma-45_Cluster_1612_sequences=92	282	92
SDS-V3-plasma-67_Cluster_4806_sequences=92	504	92
SDS-V3-plasma-67_Cluster_4800_sequences=92	504	92
SDS-V3-plasma-67_Cluster_230_sequences=92	504	92
SDS-V3-plasma-67_Cluster_677_sequences=92	504	92
SDS-V3-plasma-67_Cluster_1155_sequences=92	504	92
SDS-V3-plasma-67_Cluster_212_sequences=92	504	92
SDS-V3-plasma-67_Cluster_3368_sequences=92	504	92
SDS-V3-plasma-0_Cluster_1512_sequences=91	0	91
SDS-V3-plasma-0_Cluster_740_sequences=91	0	91
SDS-V3-plasma-5_Cluster_142_sequences=91	9	91
SDS-V3-plasma-8_Cluster_2592_sequences=91	16	91
SDS-V3-plasma-24_Cluster_34_sequences=91	124	91
SDS-V3-plasma-24_Cluster_357_sequences=91	124	91
SDS-V3-plasma-24_Cluster_51_sequences=91	124	91
SDS-V3-plasma-27_Cluster_204_sequences=91	131	91
SDS-V3-plasma-27_Cluster_556_sequences=91	131	91
SDS-V3-plasma-45_Cluster_5155_sequences=91	282	91
SDS-V3-plasma-45_Cluster_685_sequences=91	282	91
SDS-V3-plasma-45_Cluster_3480_sequences=91	282	91
SDS-V3-plasma-45_Cluster_951_sequences=91	282	91
SDS-V3-plasma-45_Cluster_80_sequences=91	282	91
SDS-V3-plasma-46_Cluster_88_sequences=91	286	91
SDS-V3-plasma-46_Cluster_608_sequences=91	286	91

SDS-V3-plasma-67_Cluster_3683_sequences=91	504	91
SDS-V3-plasma-67_Cluster_2733_sequences=91	504	91
SDS-V3-plasma-67_Cluster_827_sequences=91	504	91
SDS-V3-plasma-67_Cluster_3610_sequences=91	504	91
SDS-V3-plasma-67_Cluster_1469_sequences=91	504	91
SDS-V3-plasma-67_Cluster_7663_sequences=91	504	91
SDS-V3-plasma-67_Cluster_1376_sequences=91	504	91
SDS-V3-plasma-67_Cluster_2350_sequences=91	504	91
SDS-V3-plasma-0_Cluster_164_sequences=90	0	90
SDS-V3-plasma-0_Cluster_1235_sequences=90	0	90
SDS-V3-plasma-8_Cluster_2979_sequences=90	16	90
SDS-V3-plasma-24_Cluster_423_sequences=90	124	90
SDS-V3-plasma-24_Cluster_659_sequences=90	124	90
SDS-V3-plasma-24_Cluster_808_sequences=90	124	90
SDS-V3-plasma-27_Cluster_319_sequences=90	131	90
SDS-V3-plasma-45_Cluster_2842_sequences=90	282	90
SDS-V3-plasma-45_Cluster_981_sequences=90	282	90
SDS-V3-plasma-45_Cluster_1057_sequences=90	282	90
SDS-V3-plasma-45_Cluster_4500_sequences=90	282	90
SDS-V3-plasma-45_Cluster_4193_sequences=90	282	90
SDS-V3-plasma-45_Cluster_6711_sequences=90	282	90
SDS-V3-plasma-46_Cluster_1057_sequences=90	286	90
SDS-V3-plasma-67_Cluster_1641_sequences=90	504	90
SDS-V3-plasma-67_Cluster_3436_sequences=90	504	90
SDS-V3-plasma-67_Cluster_606_sequences=90	504	90
SDS-V3-plasma-0_Cluster_1070_sequences=89	0	89
SDS-V3-plasma-0_Cluster_255_sequences=89	0	89
SDS-V3-plasma-0_Cluster_272_sequences=89	0	89
SDS-V3-plasma-7_Cluster_307_sequences=89	14	89
SDS-V3-plasma-8_Cluster_2406_sequences=89	16	89
SDS-V3-plasma-24_Cluster_168_sequences=89	124	89
SDS-V3-plasma-24_Cluster_742_sequences=89	124	89
SDS-V3-plasma-45_Cluster_2577_sequences=89	282	89
SDS-V3-plasma-45_Cluster_7073_sequences=89	282	89
SDS-V3-plasma-45_Cluster_2860_sequences=89	282	89
SDS-V3-plasma-45_Cluster_3747_sequences=89	282	89
SDS-V3-plasma-45_Cluster_3054_sequences=89	282	89
SDS-V3-plasma-45_Cluster_4602_sequences=89	282	89
SDS-V3-plasma-67_Cluster_3928_sequences=89	504	89
SDS-V3-plasma-67_Cluster_2144_sequences=89	504	89
SDS-V3-plasma-67_Cluster_362_sequences=89	504	89
SDS-V3-plasma-67_Cluster_574_sequences=89	504	89
SDS-V3-plasma-67_Cluster_1653_sequences=89	504	89
SDS-V3-plasma-0_Cluster_208_sequences=88	0	88
SDS-V3-plasma-0_Cluster_725_sequences=88	0	88

SDS-V3-plasma-0_Cluster_497_sequences=88	0	88
SDS-V3-plasma-7_Cluster_661_sequences=88	14	88
SDS-V3-plasma-8_Cluster_2272_sequences=88	16	88
SDS-V3-plasma-24_Cluster_1636_sequences=88	124	88
SDS-V3-plasma-24_Cluster_825_sequences=88	124	88
SDS-V3-plasma-27_Cluster_1221_sequences=88	131	88
SDS-V3-plasma-27_Cluster_43_sequences=88	131	88
SDS-V3-plasma-45_Cluster_4683_sequences=88	282	88
SDS-V3-plasma-45_Cluster_6813_sequences=88	282	88
SDS-V3-plasma-45_Cluster_4351_sequences=88	282	88
SDS-V3-plasma-45_Cluster_1787_sequences=88	282	88
SDS-V3-plasma-46_Cluster_334_sequences=88	286	88
SDS-V3-plasma-67_Cluster_3587_sequences=88	504	88
SDS-V3-plasma-67_Cluster_91_sequences=88	504	88
SDS-V3-plasma-67_Cluster_4024_sequences=88	504	88
SDS-V3-plasma-67_Cluster_477_sequences=88	504	88
SDS-V3-plasma-67_Cluster_1775_sequences=88	504	88
SDS-V3-plasma-67_Cluster_1134_sequences=88	504	88
SDS-V3-plasma-67_Cluster_537_sequences=88	504	88
SDS-V3-plasma-7_Cluster_1306_sequences=87	14	87
SDS-V3-plasma-8_Cluster_2481_sequences=87	16	87
SDS-V3-plasma-24_Cluster_431_sequences=87	124	87
SDS-V3-plasma-27_Cluster_191_sequences=87	131	87
SDS-V3-plasma-27_Cluster_982_sequences=87	131	87
SDS-V3-plasma-27_Cluster_608_sequences=87	131	87
SDS-V3-plasma-45_Cluster_526_sequences=87	282	87
SDS-V3-plasma-45_Cluster_1945_sequences=87	282	87
SDS-V3-plasma-45_Cluster_2982_sequences=87	282	87
SDS-V3-plasma-45_Cluster_5171_sequences=87	282	87
SDS-V3-plasma-45_Cluster_1173_sequences=87	282	87
SDS-V3-plasma-45_Cluster_957_sequences=87	282	87
SDS-V3-plasma-45_Cluster_781_sequences=87	282	87
SDS-V3-plasma-45_Cluster_1539_sequences=87	282	87
SDS-V3-plasma-45_Cluster_1007_sequences=87	282	87
SDS-V3-plasma-45_Cluster_147_sequences=87	282	87
SDS-V3-plasma-67_Cluster_10921_sequences=87	504	87
SDS-V3-plasma-67_Cluster_4020_sequences=87	504	87
SDS-V3-plasma-67_Cluster_3399_sequences=87	504	87
SDS-V3-plasma-67_Cluster_2636_sequences=87	504	87
SDS-V3-plasma-67_Cluster_2671_sequences=87	504	87
SDS-V3-plasma-0_Cluster_2456_sequences=86	0	86
SDS-V3-plasma-0_Cluster_141_sequences=86	0	86
SDS-V3-plasma-8_Cluster_2587_sequences=86	16	86
SDS-V3-plasma-24_Cluster_143_sequences=86	124	86
SDS-V3-plasma-24_Cluster_332_sequences=86	124	86

SDS-V3-plasma-24_Cluster_186_sequences=86	124	86
SDS-V3-plasma-27_Cluster_467_sequences=86	131	86
SDS-V3-plasma-45_Cluster_2170_sequences=86	282	86
SDS-V3-plasma-45_Cluster_1144_sequences=86	282	86
SDS-V3-plasma-45_Cluster_14755_sequences=86	282	86
SDS-V3-plasma-45_Cluster_4654_sequences=86	282	86
SDS-V3-plasma-46_Cluster_493_sequences=86	286	86
SDS-V3-plasma-46_Cluster_451_sequences=86	286	86
SDS-V3-plasma-46_Cluster_531_sequences=86	286	86
SDS-V3-plasma-67_Cluster_2381_sequences=86	504	86
SDS-V3-plasma-67_Cluster_609_sequences=86	504	86
SDS-V3-plasma-67_Cluster_5053_sequences=86	504	86
SDS-V3-plasma-67_Cluster_1470_sequences=86	504	86
SDS-V3-plasma-67_Cluster_342_sequences=86	504	86
SDS-V3-plasma-0_Cluster_4573_sequences=85	0	85
SDS-V3-plasma-0_Cluster_406_sequences=85	0	85
SDS-V3-plasma-8_Cluster_2349_sequences=85	16	85
SDS-V3-plasma-24_Cluster_109_sequences=85	124	85
SDS-V3-plasma-24_Cluster_347_sequences=85	124	85
SDS-V3-plasma-27_Cluster_758_sequences=85	131	85
SDS-V3-plasma-27_Cluster_210_sequences=85	131	85
SDS-V3-plasma-45_Cluster_619_sequences=85	282	85
SDS-V3-plasma-45_Cluster_4720_sequences=85	282	85
SDS-V3-plasma-45_Cluster_1081_sequences=85	282	85
SDS-V3-plasma-45_Cluster_1255_sequences=85	282	85
SDS-V3-plasma-45_Cluster_5186_sequences=85	282	85
SDS-V3-plasma-45_Cluster_4200_sequences=85	282	85
SDS-V3-plasma-45_Cluster_548_sequences=85	282	85
SDS-V3-plasma-45_Cluster_298_sequences=85	282	85
SDS-V3-plasma-46_Cluster_3412_sequences=85	286	85
SDS-V3-plasma-67_Cluster_6547_sequences=85	504	85
SDS-V3-plasma-67_Cluster_6787_sequences=85	504	85
SDS-V3-plasma-67_Cluster_590_sequences=85	504	85
SDS-V3-plasma-67_Cluster_12027_sequences=85	504	85
SDS-V3-plasma-67_Cluster_10742_sequences=85	504	85
SDS-V3-plasma-67_Cluster_2695_sequences=85	504	85
SDS-V3-plasma-0_Cluster_150_sequences=84	0	84
SDS-V3-plasma-0_Cluster_202_sequences=84	0	84
SDS-V3-plasma-27_Cluster_57_sequences=84	131	84
SDS-V3-plasma-27_Cluster_929_sequences=84	131	84
SDS-V3-plasma-45_Cluster_336_sequences=84	282	84
SDS-V3-plasma-45_Cluster_373_sequences=84	282	84
SDS-V3-plasma-45_Cluster_7427_sequences=84	282	84
SDS-V3-plasma-45_Cluster_1695_sequences=84	282	84
SDS-V3-plasma-46_Cluster_1241_sequences=84	286	84

SDS-V3-plasma-67_Cluster_442_sequences=84	504	84
SDS-V3-plasma-67_Cluster_9533_sequences=84	504	84
SDS-V3-plasma-67_Cluster_5597_sequences=84	504	84
SDS-V3-plasma-67_Cluster_4884_sequences=84	504	84
SDS-V3-plasma-67_Cluster_3542_sequences=84	504	84
SDS-V3-plasma-0_Cluster_2713_sequences=83	0	83
SDS-V3-plasma-0_Cluster_1173_sequences=83	0	83
SDS-V3-plasma-0_Cluster_263_sequences=83	0	83
SDS-V3-plasma-0_Cluster_1040_sequences=83	0	83
SDS-V3-plasma-0_Cluster_681_sequences=83	0	83
SDS-V3-plasma-8_Cluster_2316_sequences=83	16	83
SDS-V3-plasma-24_Cluster_871_sequences=83	124	83
SDS-V3-plasma-24_Cluster_375_sequences=83	124	83
SDS-V3-plasma-24_Cluster_928_sequences=83	124	83
SDS-V3-plasma-24_Cluster_826_sequences=83	124	83
SDS-V3-plasma-24_Cluster_146_sequences=83	124	83
SDS-V3-plasma-27_Cluster_111_sequences=83	131	83
SDS-V3-plasma-27_Cluster_144_sequences=83	131	83
SDS-V3-plasma-27_Cluster_1365_sequences=83	131	83
SDS-V3-plasma-45_Cluster_4584_sequences=83	282	83
SDS-V3-plasma-45_Cluster_4757_sequences=83	282	83
SDS-V3-plasma-45_Cluster_2497_sequences=83	282	83
SDS-V3-plasma-45_Cluster_398_sequences=83	282	83
SDS-V3-plasma-45_Cluster_446_sequences=83	282	83
SDS-V3-plasma-45_Cluster_5593_sequences=83	282	83
SDS-V3-plasma-45_Cluster_73_sequences=83	282	83
SDS-V3-plasma-45_Cluster_4454_sequences=83	282	83
SDS-V3-plasma-46_Cluster_1708_sequences=83	286	83
SDS-V3-plasma-46_Cluster_1617_sequences=83	286	83
SDS-V3-plasma-67_Cluster_3053_sequences=83	504	83
SDS-V3-plasma-67_Cluster_2622_sequences=83	504	83
SDS-V3-plasma-67_Cluster_7910_sequences=83	504	83
SDS-V3-plasma-67_Cluster_1644_sequences=83	504	83
SDS-V3-plasma-0_Cluster_510_sequences=82	0	82
SDS-V3-plasma-0_Cluster_279_sequences=82	0	82
SDS-V3-plasma-0_Cluster_2341_sequences=82	0	82
SDS-V3-plasma-7_Cluster_170_sequences=82	14	82
SDS-V3-plasma-8_Cluster_2673_sequences=82	16	82
SDS-V3-plasma-27_Cluster_322_sequences=82	131	82
SDS-V3-plasma-27_Cluster_45_sequences=82	131	82
SDS-V3-plasma-27_Cluster_731_sequences=82	131	82
SDS-V3-plasma-45_Cluster_935_sequences=82	282	82
SDS-V3-plasma-45_Cluster_2621_sequences=82	282	82
SDS-V3-plasma-45_Cluster_768_sequences=82	282	82
SDS-V3-plasma-45_Cluster_755_sequences=82	282	82

SDS-V3-plasma-45_Cluster_1268_sequences=82	282	82
SDS-V3-plasma-45_Cluster_3659_sequences=82	282	82
SDS-V3-plasma-46_Cluster_154_sequences=82	286	82
SDS-V3-plasma-67_Cluster_3321_sequences=82	504	82
SDS-V3-plasma-67_Cluster_3413_sequences=82	504	82
SDS-V3-plasma-67_Cluster_427_sequences=82	504	82
SDS-V3-plasma-67_Cluster_2680_sequences=82	504	82
SDS-V3-plasma-67_Cluster_2321_sequences=82	504	82
SDS-V3-plasma-67_Cluster_1488_sequences=82	504	82
SDS-V3-plasma-0_Cluster_411_sequences=81	0	81
SDS-V3-plasma-0_Cluster_216_sequences=81	0	81
SDS-V3-plasma-7_Cluster_900_sequences=81	14	81
SDS-V3-plasma-24_Cluster_800_sequences=81	124	81
SDS-V3-plasma-24_Cluster_80_sequences=81	124	81
SDS-V3-plasma-27_Cluster_347_sequences=81	131	81
SDS-V3-plasma-45_Cluster_43_sequences=81	282	81
SDS-V3-plasma-45_Cluster_7597_sequences=81	282	81
SDS-V3-plasma-45_Cluster_3685_sequences=81	282	81
SDS-V3-plasma-45_Cluster_7670_sequences=81	282	81
SDS-V3-plasma-45_Cluster_2755_sequences=81	282	81
SDS-V3-plasma-45_Cluster_3926_sequences=81	282	81
SDS-V3-plasma-45_Cluster_2260_sequences=81	282	81
SDS-V3-plasma-45_Cluster_2726_sequences=81	282	81
SDS-V3-plasma-46_Cluster_723_sequences=81	286	81
SDS-V3-plasma-67_Cluster_306_sequences=81	504	81
SDS-V3-plasma-67_Cluster_2330_sequences=81	504	81
SDS-V3-plasma-67_Cluster_2143_sequences=81	504	81
SDS-V3-plasma-67_Cluster_1132_sequences=81	504	81
SDS-V3-plasma-0_Cluster_221_sequences=80	0	80
SDS-V3-plasma-0_Cluster_41_sequences=80	0	80
SDS-V3-plasma-0_Cluster_435_sequences=80	0	80
SDS-V3-plasma-0_Cluster_1962_sequences=80	0	80
SDS-V3-plasma-0_Cluster_1202_sequences=80	0	80
SDS-V3-plasma-0_Cluster_427_sequences=80	0	80
SDS-V3-plasma-24_Cluster_199_sequences=80	124	80
SDS-V3-plasma-24_Cluster_504_sequences=80	124	80
SDS-V3-plasma-24_Cluster_162_sequences=80	124	80
SDS-V3-plasma-27_Cluster_1943_sequences=80	131	80
SDS-V3-plasma-27_Cluster_183_sequences=80	131	80
SDS-V3-plasma-45_Cluster_5503_sequences=80	282	80
SDS-V3-plasma-45_Cluster_368_sequences=80	282	80
SDS-V3-plasma-45_Cluster_7258_sequences=80	282	80
SDS-V3-plasma-45_Cluster_2623_sequences=80	282	80
SDS-V3-plasma-46_Cluster_101_sequences=80	286	80
SDS-V3-plasma-46_Cluster_436_sequences=80	286	80

SDS-V3-plasma-46_Cluster_4223_sequences=80	286	80
SDS-V3-plasma-46_Cluster_879_sequences=80	286	80
SDS-V3-plasma-46_Cluster_1743_sequences=80	286	80
SDS-V3-plasma-67_Cluster_2590_sequences=80	504	80
SDS-V3-plasma-67_Cluster_7135_sequences=80	504	80
SDS-V3-plasma-67_Cluster_499_sequences=80	504	80
SDS-V3-plasma-67_Cluster_7536_sequences=80	504	80
SDS-V3-plasma-67_Cluster_5572_sequences=80	504	80
SDS-V3-plasma-67_Cluster_4233_sequences=80	504	80
SDS-V3-plasma-67_Cluster_1370_sequences=80	504	80
SDS-V3-plasma-67_Cluster_3904_sequences=80	504	80
SDS-V3-plasma-0_Cluster_1580_sequences=79	0	79
SDS-V3-plasma-0_Cluster_1813_sequences=79	0	79
SDS-V3-plasma-7_Cluster_1723_sequences=79	14	79
SDS-V3-plasma-8_Cluster_2502_sequences=79	16	79
SDS-V3-plasma-24_Cluster_1594_sequences=79	124	79
SDS-V3-plasma-27_Cluster_33_sequences=79	131	79
SDS-V3-plasma-27_Cluster_1834_sequences=79	131	79
SDS-V3-plasma-27_Cluster_1438_sequences=79	131	79
SDS-V3-plasma-27_Cluster_324_sequences=79	131	79
SDS-V3-plasma-27_Cluster_332_sequences=79	131	79
SDS-V3-plasma-27_Cluster_250_sequences=79	131	79
SDS-V3-plasma-45_Cluster_878_sequences=79	282	79
SDS-V3-plasma-45_Cluster_8045_sequences=79	282	79
SDS-V3-plasma-45_Cluster_2460_sequences=79	282	79
SDS-V3-plasma-45_Cluster_1855_sequences=79	282	79
SDS-V3-plasma-45_Cluster_401_sequences=79	282	79
SDS-V3-plasma-45_Cluster_570_sequences=79	282	79
SDS-V3-plasma-46_Cluster_1765_sequences=79	286	79
SDS-V3-plasma-46_Cluster_64_sequences=79	286	79
SDS-V3-plasma-46_Cluster_87_sequences=79	286	79
SDS-V3-plasma-46_Cluster_752_sequences=79	286	79
SDS-V3-plasma-67_Cluster_2068_sequences=79	504	79
SDS-V3-plasma-67_Cluster_162_sequences=79	504	79
SDS-V3-plasma-67_Cluster_3079_sequences=79	504	79
SDS-V3-plasma-67_Cluster_1973_sequences=79	504	79
SDS-V3-plasma-67_Cluster_15250_sequences=79	504	79
SDS-V3-plasma-0_Cluster_1000_sequences=78	0	78
SDS-V3-plasma-0_Cluster_624_sequences=78	0	78
SDS-V3-plasma-8_Cluster_2459_sequences=78	16	78
SDS-V3-plasma-8_Cluster_2405_sequences=78	16	78
SDS-V3-plasma-24_Cluster_300_sequences=78	124	78
SDS-V3-plasma-24_Cluster_670_sequences=78	124	78
SDS-V3-plasma-27_Cluster_230_sequences=78	131	78
SDS-V3-plasma-45_Cluster_1944_sequences=78	282	78

SDS-V3-plasma-45_Cluster_2426_sequences=78	282	78
SDS-V3-plasma-45_Cluster_1124_sequences=78	282	78
SDS-V3-plasma-45_Cluster_11033_sequences=78	282	78
SDS-V3-plasma-46_Cluster_227_sequences=78	286	78
SDS-V3-plasma-46_Cluster_2943_sequences=78	286	78
SDS-V3-plasma-67_Cluster_6703_sequences=78	504	78
SDS-V3-plasma-67_Cluster_510_sequences=78	504	78
SDS-V3-plasma-67_Cluster_591_sequences=78	504	78
SDS-V3-plasma-67_Cluster_1161_sequences=78	504	78
SDS-V3-plasma-67_Cluster_595_sequences=78	504	78
SDS-V3-plasma-67_Cluster_2757_sequences=78	504	78
SDS-V3-plasma-0_Cluster_765_sequences=77	0	77
SDS-V3-plasma-0_Cluster_1379_sequences=77	0	77
SDS-V3-plasma-7_Cluster_535_sequences=77	14	77
SDS-V3-plasma-7_Cluster_204_sequences=77	14	77
SDS-V3-plasma-24_Cluster_65_sequences=77	124	77
SDS-V3-plasma-24_Cluster_705_sequences=77	124	77
SDS-V3-plasma-24_Cluster_450_sequences=77	124	77
SDS-V3-plasma-24_Cluster_451_sequences=77	124	77
SDS-V3-plasma-24_Cluster_502_sequences=77	124	77
SDS-V3-plasma-24_Cluster_223_sequences=77	124	77
SDS-V3-plasma-27_Cluster_438_sequences=77	131	77
SDS-V3-plasma-27_Cluster_483_sequences=77	131	77
SDS-V3-plasma-27_Cluster_933_sequences=77	131	77
SDS-V3-plasma-27_Cluster_318_sequences=77	131	77
SDS-V3-plasma-27_Cluster_3750_sequences=77	131	77
SDS-V3-plasma-27_Cluster_803_sequences=77	131	77
SDS-V3-plasma-45_Cluster_835_sequences=77	282	77
SDS-V3-plasma-45_Cluster_1819_sequences=77	282	77
SDS-V3-plasma-45_Cluster_4290_sequences=77	282	77
SDS-V3-plasma-45_Cluster_37_sequences=77	282	77
SDS-V3-plasma-45_Cluster_1403_sequences=77	282	77
SDS-V3-plasma-45_Cluster_4636_sequences=77	282	77
SDS-V3-plasma-45_Cluster_5558_sequences=77	282	77
SDS-V3-plasma-46_Cluster_3514_sequences=77	286	77
SDS-V3-plasma-46_Cluster_590_sequences=77	286	77
SDS-V3-plasma-46_Cluster_373_sequences=77	286	77
SDS-V3-plasma-67_Cluster_7463_sequences=77	504	77
SDS-V3-plasma-67_Cluster_1423_sequences=77	504	77
SDS-V3-plasma-67_Cluster_1291_sequences=77	504	77
SDS-V3-plasma-67_Cluster_3859_sequences=77	504	77
SDS-V3-plasma-67_Cluster_5559_sequences=77	504	77
SDS-V3-plasma-67_Cluster_6474_sequences=77	504	77
SDS-V3-plasma-67_Cluster_2169_sequences=77	504	77
SDS-V3-plasma-0_Cluster_250_sequences=76	0	76

SDS-V3-plasma-0_Cluster_1261_sequences=76	0	76
SDS-V3-plasma-24_Cluster_324_sequences=76	124	76
SDS-V3-plasma-27_Cluster_1873_sequences=76	131	76
SDS-V3-plasma-27_Cluster_517_sequences=76	131	76
SDS-V3-plasma-27_Cluster_55_sequences=76	131	76
SDS-V3-plasma-27_Cluster_249_sequences=76	131	76
SDS-V3-plasma-45_Cluster_681_sequences=76	282	76
SDS-V3-plasma-45_Cluster_639_sequences=76	282	76
SDS-V3-plasma-45_Cluster_197_sequences=76	282	76
SDS-V3-plasma-45_Cluster_51_sequences=76	282	76
SDS-V3-plasma-45_Cluster_1431_sequences=76	282	76
SDS-V3-plasma-45_Cluster_6138_sequences=76	282	76
SDS-V3-plasma-45_Cluster_510_sequences=76	282	76
SDS-V3-plasma-45_Cluster_4647_sequences=76	282	76
SDS-V3-plasma-45_Cluster_1558_sequences=76	282	76
SDS-V3-plasma-46_Cluster_756_sequences=76	286	76
SDS-V3-plasma-67_Cluster_5376_sequences=76	504	76
SDS-V3-plasma-67_Cluster_8090_sequences=76	504	76
SDS-V3-plasma-67_Cluster_903_sequences=76	504	76
SDS-V3-plasma-67_Cluster_450_sequences=76	504	76
SDS-V3-plasma-67_Cluster_1674_sequences=76	504	76
SDS-V3-plasma-67_Cluster_2148_sequences=76	504	76
SDS-V3-plasma-67_Cluster_3473_sequences=76	504	76
SDS-V3-plasma-67_Cluster_3215_sequences=76	504	76
SDS-V3-plasma-67_Cluster_3796_sequences=76	504	76
SDS-V3-plasma-67_Cluster_1241_sequences=76	504	76
SDS-V3-plasma-67_Cluster_2844_sequences=76	504	76
SDS-V3-plasma-67_Cluster_8834_sequences=76	504	76
SDS-V3-plasma-0_Cluster_1594_sequences=75	0	75
SDS-V3-plasma-0_Cluster_1943_sequences=75	0	75
SDS-V3-plasma-0_Cluster_911_sequences=75	0	75
SDS-V3-plasma-0_Cluster_929_sequences=75	0	75
SDS-V3-plasma-5_Cluster_53_sequences=75	9	75
SDS-V3-plasma-7_Cluster_805_sequences=75	14	75
SDS-V3-plasma-24_Cluster_181_sequences=75	124	75
SDS-V3-plasma-24_Cluster_352_sequences=75	124	75
SDS-V3-plasma-24_Cluster_834_sequences=75	124	75
SDS-V3-plasma-24_Cluster_752_sequences=75	124	75
SDS-V3-plasma-27_Cluster_725_sequences=75	131	75
SDS-V3-plasma-27_Cluster_776_sequences=75	131	75
SDS-V3-plasma-27_Cluster_733_sequences=75	131	75
SDS-V3-plasma-45_Cluster_2733_sequences=75	282	75
SDS-V3-plasma-45_Cluster_6251_sequences=75	282	75
SDS-V3-plasma-45_Cluster_74_sequences=75	282	75
SDS-V3-plasma-45_Cluster_1349_sequences=75	282	75

SDS-V3-plasma-45_Cluster_12354_sequences=75	282	75
SDS-V3-plasma-45_Cluster_9261_sequences=75	282	75
SDS-V3-plasma-45_Cluster_717_sequences=75	282	75
SDS-V3-plasma-45_Cluster_1214_sequences=75	282	75
SDS-V3-plasma-46_Cluster_294_sequences=75	286	75
SDS-V3-plasma-46_Cluster_1044_sequences=75	286	75
SDS-V3-plasma-46_Cluster_1337_sequences=75	286	75
SDS-V3-plasma-46_Cluster_435_sequences=75	286	75
SDS-V3-plasma-67_Cluster_4348_sequences=75	504	75
SDS-V3-plasma-67_Cluster_204_sequences=75	504	75
SDS-V3-plasma-67_Cluster_14558_sequences=75	504	75
SDS-V3-plasma-67_Cluster_2208_sequences=75	504	75
SDS-V3-plasma-67_Cluster_2652_sequences=75	504	75
SDS-V3-plasma-67_Cluster_8121_sequences=75	504	75
SDS-V3-plasma-67_Cluster_4224_sequences=75	504	75
SDS-V3-plasma-67_Cluster_2408_sequences=75	504	75
SDS-V3-plasma-67_Cluster_529_sequences=75	504	75
SDS-V3-plasma-67_Cluster_6653_sequences=75	504	75
SDS-V3-plasma-67_Cluster_741_sequences=75	504	75
SDS-V3-plasma-67_Cluster_8941_sequences=75	504	75
SDS-V3-plasma-67_Cluster_13130_sequences=75	504	75
SDS-V3-plasma-0_Cluster_947_sequences=74	0	74
SDS-V3-plasma-0_Cluster_1501_sequences=74	0	74
SDS-V3-plasma-0_Cluster_3229_sequences=74	0	74
SDS-V3-plasma-0_Cluster_357_sequences=74	0	74
SDS-V3-plasma-8_Cluster_2500_sequences=74	16	74
SDS-V3-plasma-24_Cluster_1804_sequences=74	124	74
SDS-V3-plasma-24_Cluster_979_sequences=74	124	74
SDS-V3-plasma-24_Cluster_527_sequences=74	124	74
SDS-V3-plasma-24_Cluster_574_sequences=74	124	74
SDS-V3-plasma-27_Cluster_1066_sequences=74	131	74
SDS-V3-plasma-27_Cluster_214_sequences=74	131	74
SDS-V3-plasma-27_Cluster_572_sequences=74	131	74
SDS-V3-plasma-45_Cluster_1236_sequences=74	282	74
SDS-V3-plasma-45_Cluster_3850_sequences=74	282	74
SDS-V3-plasma-45_Cluster_3297_sequences=74	282	74
SDS-V3-plasma-45_Cluster_1032_sequences=74	282	74
SDS-V3-plasma-45_Cluster_5403_sequences=74	282	74
SDS-V3-plasma-45_Cluster_4951_sequences=74	282	74
SDS-V3-plasma-67_Cluster_507_sequences=74	504	74
SDS-V3-plasma-67_Cluster_1841_sequences=74	504	74
SDS-V3-plasma-67_Cluster_2568_sequences=74	504	74
SDS-V3-plasma-67_Cluster_4722_sequences=74	504	74
SDS-V3-plasma-0_Cluster_955_sequences=73	0	73
SDS-V3-plasma-0_Cluster_270_sequences=73	0	73

SDS-V3-plasma-0_Cluster_570_sequences=73	0	73
SDS-V3-plasma-24_Cluster_328_sequences=73	124	73
SDS-V3-plasma-24_Cluster_623_sequences=73	124	73
SDS-V3-plasma-27_Cluster_1165_sequences=73	131	73
SDS-V3-plasma-45_Cluster_7992_sequences=73	282	73
SDS-V3-plasma-45_Cluster_281_sequences=73	282	73
SDS-V3-plasma-45_Cluster_3081_sequences=73	282	73
SDS-V3-plasma-45_Cluster_47_sequences=73	282	73
SDS-V3-plasma-45_Cluster_3475_sequences=73	282	73
SDS-V3-plasma-45_Cluster_5156_sequences=73	282	73
SDS-V3-plasma-45_Cluster_3137_sequences=73	282	73
SDS-V3-plasma-45_Cluster_6704_sequences=73	282	73
SDS-V3-plasma-46_Cluster_205_sequences=73	286	73
SDS-V3-plasma-46_Cluster_1200_sequences=73	286	73
SDS-V3-plasma-67_Cluster_7717_sequences=73	504	73
SDS-V3-plasma-67_Cluster_1537_sequences=73	504	73
SDS-V3-plasma-67_Cluster_897_sequences=73	504	73
SDS-V3-plasma-67_Cluster_526_sequences=73	504	73
SDS-V3-plasma-67_Cluster_1145_sequences=73	504	73
SDS-V3-plasma-0_Cluster_925_sequences=72	0	72
SDS-V3-plasma-0_Cluster_2502_sequences=72	0	72
SDS-V3-plasma-0_Cluster_761_sequences=72	0	72
SDS-V3-plasma-7_Cluster_75_sequences=72	14	72
SDS-V3-plasma-8_Cluster_2606_sequences=72	16	72
SDS-V3-plasma-24_Cluster_758_sequences=72	124	72
SDS-V3-plasma-24_Cluster_1175_sequences=72	124	72
SDS-V3-plasma-27_Cluster_299_sequences=72	131	72
SDS-V3-plasma-27_Cluster_79_sequences=72	131	72
SDS-V3-plasma-45_Cluster_1197_sequences=72	282	72
SDS-V3-plasma-45_Cluster_370_sequences=72	282	72
SDS-V3-plasma-45_Cluster_3904_sequences=72	282	72
SDS-V3-plasma-45_Cluster_1813_sequences=72	282	72
SDS-V3-plasma-45_Cluster_1500_sequences=72	282	72
SDS-V3-plasma-45_Cluster_4101_sequences=72	282	72
SDS-V3-plasma-45_Cluster_142_sequences=72	282	72
SDS-V3-plasma-45_Cluster_2154_sequences=72	282	72
SDS-V3-plasma-45_Cluster_35_sequences=72	282	72
SDS-V3-plasma-45_Cluster_7703_sequences=72	282	72
SDS-V3-plasma-45_Cluster_4862_sequences=72	282	72
SDS-V3-plasma-45_Cluster_6727_sequences=72	282	72
SDS-V3-plasma-45_Cluster_103_sequences=72	282	72
SDS-V3-plasma-45_Cluster_9041_sequences=72	282	72
SDS-V3-plasma-45_Cluster_650_sequences=72	282	72
SDS-V3-plasma-45_Cluster_6708_sequences=72	282	72
SDS-V3-plasma-45_Cluster_1302_sequences=72	282	72

SDS-V3-plasma-45_Cluster_2373_sequences=72	282	72
SDS-V3-plasma-46_Cluster_1012_sequences=72	286	72
SDS-V3-plasma-46_Cluster_1871_sequences=72	286	72
SDS-V3-plasma-67_Cluster_2505_sequences=72	504	72
SDS-V3-plasma-67_Cluster_10631_sequences=72	504	72
SDS-V3-plasma-67_Cluster_891_sequences=72	504	72
SDS-V3-plasma-0_Cluster_1426_sequences=71	0	71
SDS-V3-plasma-0_Cluster_1635_sequences=71	0	71
SDS-V3-plasma-0_Cluster_2040_sequences=71	0	71
SDS-V3-plasma-0_Cluster_534_sequences=71	0	71
SDS-V3-plasma-0_Cluster_461_sequences=71	0	71
SDS-V3-plasma-0_Cluster_10823_sequences=71	0	71
SDS-V3-plasma-8_Cluster_2411_sequences=71	16	71
SDS-V3-plasma-8_Cluster_2809_sequences=71	16	71
SDS-V3-plasma-24_Cluster_1108_sequences=71	124	71
SDS-V3-plasma-24_Cluster_128_sequences=71	124	71
SDS-V3-plasma-24_Cluster_216_sequences=71	124	71
SDS-V3-plasma-24_Cluster_392_sequences=71	124	71
SDS-V3-plasma-24_Cluster_1004_sequences=71	124	71
SDS-V3-plasma-27_Cluster_244_sequences=71	131	71
SDS-V3-plasma-27_Cluster_649_sequences=71	131	71
SDS-V3-plasma-27_Cluster_96_sequences=71	131	71
SDS-V3-plasma-27_Cluster_32_sequences=71	131	71
SDS-V3-plasma-27_Cluster_54_sequences=71	131	71
SDS-V3-plasma-45_Cluster_7663_sequences=71	282	71
SDS-V3-plasma-45_Cluster_2155_sequences=71	282	71
SDS-V3-plasma-45_Cluster_4008_sequences=71	282	71
SDS-V3-plasma-45_Cluster_1267_sequences=71	282	71
SDS-V3-plasma-45_Cluster_5647_sequences=71	282	71
SDS-V3-plasma-45_Cluster_2125_sequences=71	282	71
SDS-V3-plasma-45_Cluster_1597_sequences=71	282	71
SDS-V3-plasma-45_Cluster_9331_sequences=71	282	71
SDS-V3-plasma-45_Cluster_684_sequences=71	282	71
SDS-V3-plasma-46_Cluster_1978_sequences=71	286	71
SDS-V3-plasma-46_Cluster_934_sequences=71	286	71
SDS-V3-plasma-46_Cluster_853_sequences=71	286	71
SDS-V3-plasma-46_Cluster_778_sequences=71	286	71
SDS-V3-plasma-46_Cluster_649_sequences=71	286	71
SDS-V3-plasma-46_Cluster_2012_sequences=71	286	71
SDS-V3-plasma-67_Cluster_858_sequences=71	504	71
SDS-V3-plasma-67_Cluster_1578_sequences=71	504	71
SDS-V3-plasma-67_Cluster_6108_sequences=71	504	71
SDS-V3-plasma-67_Cluster_3881_sequences=71	504	71
SDS-V3-plasma-67_Cluster_759_sequences=71	504	71
SDS-V3-plasma-0_Cluster_271_sequences=70	0	70

SDS-V3-plasma-0_Cluster_121_sequences=70	0	70
SDS-V3-plasma-0_Cluster_1153_sequences=70	0	70
SDS-V3-plasma-0_Cluster_217_sequences=70	0	70
SDS-V3-plasma-0_Cluster_502_sequences=70	0	70
SDS-V3-plasma-0_Cluster_2049_sequences=70	0	70
SDS-V3-plasma-8_Cluster_2369_sequences=70	16	70
SDS-V3-plasma-24_Cluster_208_sequences=70	124	70
SDS-V3-plasma-27_Cluster_165_sequences=70	131	70
SDS-V3-plasma-27_Cluster_46_sequences=70	131	70
SDS-V3-plasma-27_Cluster_796_sequences=70	131	70
SDS-V3-plasma-45_Cluster_8039_sequences=70	282	70
SDS-V3-plasma-45_Cluster_5756_sequences=70	282	70
SDS-V3-plasma-45_Cluster_5445_sequences=70	282	70
SDS-V3-plasma-45_Cluster_6388_sequences=70	282	70
SDS-V3-plasma-45_Cluster_6917_sequences=70	282	70
SDS-V3-plasma-45_Cluster_172_sequences=70	282	70
SDS-V3-plasma-45_Cluster_6619_sequences=70	282	70
SDS-V3-plasma-45_Cluster_2629_sequences=70	282	70
SDS-V3-plasma-45_Cluster_6321_sequences=70	282	70
SDS-V3-plasma-45_Cluster_1568_sequences=70	282	70
SDS-V3-plasma-45_Cluster_4569_sequences=70	282	70
SDS-V3-plasma-45_Cluster_1126_sequences=70	282	70
SDS-V3-plasma-45_Cluster_11247_sequences=70	282	70
SDS-V3-plasma-46_Cluster_2827_sequences=70	286	70
SDS-V3-plasma-46_Cluster_803_sequences=70	286	70
SDS-V3-plasma-46_Cluster_1831_sequences=70	286	70
SDS-V3-plasma-46_Cluster_743_sequences=70	286	70
SDS-V3-plasma-67_Cluster_2812_sequences=70	504	70
SDS-V3-plasma-67_Cluster_1040_sequences=70	504	70
SDS-V3-plasma-67_Cluster_1170_sequences=70	504	70
SDS-V3-plasma-67_Cluster_5742_sequences=70	504	70
SDS-V3-plasma-67_Cluster_1522_sequences=70	504	70
SDS-V3-plasma-67_Cluster_2776_sequences=70	504	70
SDS-V3-plasma-0_Cluster_466_sequences=69	0	69
SDS-V3-plasma-0_Cluster_485_sequences=69	0	69
SDS-V3-plasma-0_Cluster_1037_sequences=69	0	69
SDS-V3-plasma-0_Cluster_117_sequences=69	0	69
SDS-V3-plasma-0_Cluster_952_sequences=69	0	69
SDS-V3-plasma-24_Cluster_45_sequences=69	124	69
SDS-V3-plasma-24_Cluster_360_sequences=69	124	69
SDS-V3-plasma-24_Cluster_412_sequences=69	124	69
SDS-V3-plasma-45_Cluster_5296_sequences=69	282	69
SDS-V3-plasma-45_Cluster_11197_sequences=69	282	69
SDS-V3-plasma-45_Cluster_6622_sequences=69	282	69
SDS-V3-plasma-45_Cluster_4493_sequences=69	282	69

SDS-V3-plasma-45_Cluster_4252_sequences=69	282	69
SDS-V3-plasma-45_Cluster_7402_sequences=69	282	69
SDS-V3-plasma-45_Cluster_5997_sequences=69	282	69
SDS-V3-plasma-45_Cluster_387_sequences=69	282	69
SDS-V3-plasma-45_Cluster_1943_sequences=69	282	69
SDS-V3-plasma-45_Cluster_1620_sequences=69	282	69
SDS-V3-plasma-45_Cluster_606_sequences=69	282	69
SDS-V3-plasma-45_Cluster_3587_sequences=69	282	69
SDS-V3-plasma-45_Cluster_2894_sequences=69	282	69
SDS-V3-plasma-45_Cluster_945_sequences=69	282	69
SDS-V3-plasma-46_Cluster_5060_sequences=69	286	69
SDS-V3-plasma-46_Cluster_468_sequences=69	286	69
SDS-V3-plasma-46_Cluster_2790_sequences=69	286	69
SDS-V3-plasma-46_Cluster_703_sequences=69	286	69
SDS-V3-plasma-46_Cluster_337_sequences=69	286	69
SDS-V3-plasma-67_Cluster_907_sequences=69	504	69
SDS-V3-plasma-67_Cluster_4050_sequences=69	504	69
SDS-V3-plasma-67_Cluster_4905_sequences=69	504	69
SDS-V3-plasma-67_Cluster_3252_sequences=69	504	69
SDS-V3-plasma-67_Cluster_1412_sequences=69	504	69
SDS-V3-plasma-67_Cluster_1455_sequences=69	504	69
SDS-V3-plasma-67_Cluster_2697_sequences=69	504	69
SDS-V3-plasma-67_Cluster_6580_sequences=69	504	69
SDS-V3-plasma-67_Cluster_1870_sequences=69	504	69
SDS-V3-plasma-67_Cluster_4297_sequences=69	504	69
SDS-V3-plasma-67_Cluster_755_sequences=69	504	69
SDS-V3-plasma-67_Cluster_109_sequences=69	504	69
SDS-V3-plasma-0_Cluster_622_sequences=68	0	68
SDS-V3-plasma-0_Cluster_1504_sequences=68	0	68
SDS-V3-plasma-0_Cluster_985_sequences=68	0	68
SDS-V3-plasma-5_Cluster_19_sequences=68	9	68
SDS-V3-plasma-24_Cluster_91_sequences=68	124	68
SDS-V3-plasma-24_Cluster_136_sequences=68	124	68
SDS-V3-plasma-24_Cluster_1016_sequences=68	124	68
SDS-V3-plasma-27_Cluster_212_sequences=68	131	68
SDS-V3-plasma-27_Cluster_536_sequences=68	131	68
SDS-V3-plasma-27_Cluster_161_sequences=68	131	68
SDS-V3-plasma-45_Cluster_2498_sequences=68	282	68
SDS-V3-plasma-45_Cluster_1462_sequences=68	282	68
SDS-V3-plasma-45_Cluster_1343_sequences=68	282	68
SDS-V3-plasma-45_Cluster_4410_sequences=68	282	68
SDS-V3-plasma-45_Cluster_7888_sequences=68	282	68
SDS-V3-plasma-45_Cluster_6554_sequences=68	282	68
SDS-V3-plasma-45_Cluster_4826_sequences=68	282	68
SDS-V3-plasma-45_Cluster_1356_sequences=68	282	68

SDS-V3-plasma-45_Cluster_941_sequences=68	282	68
SDS-V3-plasma-45_Cluster_8500_sequences=68	282	68
SDS-V3-plasma-45_Cluster_3437_sequences=68	282	68
SDS-V3-plasma-45_Cluster_9670_sequences=68	282	68
SDS-V3-plasma-45_Cluster_9111_sequences=68	282	68
SDS-V3-plasma-45_Cluster_3250_sequences=68	282	68
SDS-V3-plasma-67_Cluster_1080_sequences=68	504	68
SDS-V3-plasma-67_Cluster_1067_sequences=68	504	68
SDS-V3-plasma-67_Cluster_575_sequences=68	504	68
SDS-V3-plasma-67_Cluster_165_sequences=68	504	68
SDS-V3-plasma-67_Cluster_4197_sequences=68	504	68
SDS-V3-plasma-67_Cluster_2604_sequences=68	504	68
SDS-V3-plasma-67_Cluster_1663_sequences=68	504	68
SDS-V3-plasma-67_Cluster_3182_sequences=68	504	68
SDS-V3-plasma-67_Cluster_17125_sequences=68	504	68
SDS-V3-plasma-0_Cluster_707_sequences=67	0	67
SDS-V3-plasma-0_Cluster_114_sequences=67	0	67
SDS-V3-plasma-0_Cluster_321_sequences=67	0	67
SDS-V3-plasma-0_Cluster_1383_sequences=67	0	67
SDS-V3-plasma-7_Cluster_918_sequences=67	14	67
SDS-V3-plasma-8_Cluster_2864_sequences=67	16	67
SDS-V3-plasma-24_Cluster_645_sequences=67	124	67
SDS-V3-plasma-24_Cluster_648_sequences=67	124	67
SDS-V3-plasma-24_Cluster_850_sequences=67	124	67
SDS-V3-plasma-27_Cluster_1942_sequences=67	131	67
SDS-V3-plasma-27_Cluster_50_sequences=67	131	67
SDS-V3-plasma-27_Cluster_853_sequences=67	131	67
SDS-V3-plasma-27_Cluster_95_sequences=67	131	67
SDS-V3-plasma-27_Cluster_487_sequences=67	131	67
SDS-V3-plasma-27_Cluster_984_sequences=67	131	67
SDS-V3-plasma-27_Cluster_730_sequences=67	131	67
SDS-V3-plasma-45_Cluster_2191_sequences=67	282	67
SDS-V3-plasma-45_Cluster_3362_sequences=67	282	67
SDS-V3-plasma-45_Cluster_5054_sequences=67	282	67
SDS-V3-plasma-45_Cluster_3729_sequences=67	282	67
SDS-V3-plasma-45_Cluster_5847_sequences=67	282	67
SDS-V3-plasma-45_Cluster_2809_sequences=67	282	67
SDS-V3-plasma-45_Cluster_707_sequences=67	282	67
SDS-V3-plasma-45_Cluster_4105_sequences=67	282	67
SDS-V3-plasma-45_Cluster_13013_sequences=67	282	67
SDS-V3-plasma-45_Cluster_516_sequences=67	282	67
SDS-V3-plasma-45_Cluster_3168_sequences=67	282	67
SDS-V3-plasma-46_Cluster_536_sequences=67	286	67
SDS-V3-plasma-67_Cluster_148_sequences=67	504	67
SDS-V3-plasma-67_Cluster_8034_sequences=67	504	67

SDS-V3-plasma-67_Cluster_4879_sequences=67	504	67
SDS-V3-plasma-67_Cluster_3395_sequences=67	504	67
SDS-V3-plasma-67_Cluster_414_sequences=67	504	67
SDS-V3-plasma-67_Cluster_1297_sequences=67	504	67
SDS-V3-plasma-0_Cluster_1211_sequences=66	0	66
SDS-V3-plasma-0_Cluster_729_sequences=66	0	66
SDS-V3-plasma-0_Cluster_1808_sequences=66	0	66
SDS-V3-plasma-5_Cluster_10_sequences=66	9	66
SDS-V3-plasma-24_Cluster_1347_sequences=66	124	66
SDS-V3-plasma-24_Cluster_140_sequences=66	124	66
SDS-V3-plasma-24_Cluster_235_sequences=66	124	66
SDS-V3-plasma-24_Cluster_82_sequences=66	124	66
SDS-V3-plasma-24_Cluster_1126_sequences=66	124	66
SDS-V3-plasma-24_Cluster_999_sequences=66	124	66
SDS-V3-plasma-27_Cluster_140_sequences=66	131	66
SDS-V3-plasma-27_Cluster_772_sequences=66	131	66
SDS-V3-plasma-27_Cluster_1351_sequences=66	131	66
SDS-V3-plasma-27_Cluster_175_sequences=66	131	66
SDS-V3-plasma-45_Cluster_535_sequences=66	282	66
SDS-V3-plasma-45_Cluster_8756_sequences=66	282	66
SDS-V3-plasma-45_Cluster_1523_sequences=66	282	66
SDS-V3-plasma-45_Cluster_2161_sequences=66	282	66
SDS-V3-plasma-45_Cluster_506_sequences=66	282	66
SDS-V3-plasma-45_Cluster_2102_sequences=66	282	66
SDS-V3-plasma-45_Cluster_1894_sequences=66	282	66
SDS-V3-plasma-45_Cluster_2797_sequences=66	282	66
SDS-V3-plasma-45_Cluster_5072_sequences=66	282	66
SDS-V3-plasma-45_Cluster_1847_sequences=66	282	66
SDS-V3-plasma-45_Cluster_3374_sequences=66	282	66
SDS-V3-plasma-45_Cluster_5120_sequences=66	282	66
SDS-V3-plasma-46_Cluster_434_sequences=66	286	66
SDS-V3-plasma-46_Cluster_2064_sequences=66	286	66
SDS-V3-plasma-67_Cluster_2872_sequences=66	504	66
SDS-V3-plasma-67_Cluster_946_sequences=66	504	66
SDS-V3-plasma-67_Cluster_3352_sequences=66	504	66
SDS-V3-plasma-67_Cluster_252_sequences=66	504	66
SDS-V3-plasma-67_Cluster_2361_sequences=66	504	66
SDS-V3-plasma-67_Cluster_3496_sequences=66	504	66
SDS-V3-plasma-67_Cluster_5907_sequences=66	504	66
SDS-V3-plasma-67_Cluster_815_sequences=66	504	66
SDS-V3-plasma-67_Cluster_3503_sequences=66	504	66
SDS-V3-plasma-67_Cluster_3358_sequences=66	504	66
SDS-V3-plasma-67_Cluster_350_sequences=66	504	66
SDS-V3-plasma-0_Cluster_210_sequences=65	0	65
SDS-V3-plasma-0_Cluster_1535_sequences=65	0	65

SDS-V3-plasma-0_Cluster_122_sequences=65	0	65
SDS-V3-plasma-0_Cluster_454_sequences=65	0	65
SDS-V3-plasma-0_Cluster_893_sequences=65	0	65
SDS-V3-plasma-0_Cluster_907_sequences=65	0	65
SDS-V3-plasma-5_Cluster_85_sequences=65	9	65
SDS-V3-plasma-24_Cluster_456_sequences=65	124	65
SDS-V3-plasma-24_Cluster_630_sequences=65	124	65
SDS-V3-plasma-24_Cluster_740_sequences=65	124	65
SDS-V3-plasma-24_Cluster_1056_sequences=65	124	65
SDS-V3-plasma-27_Cluster_886_sequences=65	131	65
SDS-V3-plasma-27_Cluster_254_sequences=65	131	65
SDS-V3-plasma-27_Cluster_618_sequences=65	131	65
SDS-V3-plasma-27_Cluster_337_sequences=65	131	65
SDS-V3-plasma-27_Cluster_289_sequences=65	131	65
SDS-V3-plasma-45_Cluster_6634_sequences=65	282	65
SDS-V3-plasma-45_Cluster_11032_sequences=65	282	65
SDS-V3-plasma-45_Cluster_5279_sequences=65	282	65
SDS-V3-plasma-45_Cluster_2404_sequences=65	282	65
SDS-V3-plasma-45_Cluster_4545_sequences=65	282	65
SDS-V3-plasma-45_Cluster_10851_sequences=65	282	65
SDS-V3-plasma-45_Cluster_14964_sequences=65	282	65
SDS-V3-plasma-45_Cluster_6769_sequences=65	282	65
SDS-V3-plasma-45_Cluster_721_sequences=65	282	65
SDS-V3-plasma-45_Cluster_1948_sequences=65	282	65
SDS-V3-plasma-45_Cluster_1733_sequences=65	282	65
SDS-V3-plasma-67_Cluster_3157_sequences=65	504	65
SDS-V3-plasma-67_Cluster_4971_sequences=65	504	65
SDS-V3-plasma-67_Cluster_2704_sequences=65	504	65
SDS-V3-plasma-67_Cluster_17010_sequences=65	504	65
SDS-V3-plasma-67_Cluster_4674_sequences=65	504	65
SDS-V3-plasma-67_Cluster_4881_sequences=65	504	65
SDS-V3-plasma-0_Cluster_4866_sequences=64	0	64
SDS-V3-plasma-0_Cluster_861_sequences=64	0	64
SDS-V3-plasma-0_Cluster_60_sequences=64	0	64
SDS-V3-plasma-0_Cluster_2562_sequences=64	0	64
SDS-V3-plasma-0_Cluster_1055_sequences=64	0	64
SDS-V3-plasma-0_Cluster_2847_sequences=64	0	64
SDS-V3-plasma-7_Cluster_694_sequences=64	14	64
SDS-V3-plasma-7_Cluster_430_sequences=64	14	64
SDS-V3-plasma-8_Cluster_2612_sequences=64	16	64
SDS-V3-plasma-24_Cluster_749_sequences=64	124	64
SDS-V3-plasma-24_Cluster_763_sequences=64	124	64
SDS-V3-plasma-24_Cluster_1124_sequences=64	124	64
SDS-V3-plasma-24_Cluster_231_sequences=64	124	64
SDS-V3-plasma-24_Cluster_540_sequences=64	124	64

SDS-V3-plasma-27_Cluster_995_sequences=64	131	64
SDS-V3-plasma-27_Cluster_1044_sequences=64	131	64
SDS-V3-plasma-45_Cluster_9791_sequences=64	282	64
SDS-V3-plasma-45_Cluster_1837_sequences=64	282	64
SDS-V3-plasma-45_Cluster_4623_sequences=64	282	64
SDS-V3-plasma-45_Cluster_725_sequences=64	282	64
SDS-V3-plasma-45_Cluster_3777_sequences=64	282	64
SDS-V3-plasma-45_Cluster_9949_sequences=64	282	64
SDS-V3-plasma-45_Cluster_909_sequences=64	282	64
SDS-V3-plasma-45_Cluster_7357_sequences=64	282	64
SDS-V3-plasma-45_Cluster_3047_sequences=64	282	64
SDS-V3-plasma-45_Cluster_2291_sequences=64	282	64
SDS-V3-plasma-45_Cluster_1498_sequences=64	282	64
SDS-V3-plasma-46_Cluster_2236_sequences=64	286	64
SDS-V3-plasma-46_Cluster_1993_sequences=64	286	64
SDS-V3-plasma-46_Cluster_771_sequences=64	286	64
SDS-V3-plasma-46_Cluster_380_sequences=64	286	64
SDS-V3-plasma-67_Cluster_1210_sequences=64	504	64
SDS-V3-plasma-67_Cluster_1191_sequences=64	504	64
SDS-V3-plasma-67_Cluster_714_sequences=64	504	64
SDS-V3-plasma-67_Cluster_7800_sequences=64	504	64
SDS-V3-plasma-67_Cluster_2107_sequences=64	504	64
SDS-V3-plasma-67_Cluster_4029_sequences=64	504	64
SDS-V3-plasma-67_Cluster_4290_sequences=64	504	64
SDS-V3-plasma-67_Cluster_13147_sequences=64	504	64
SDS-V3-plasma-0_Cluster_1375_sequences=63	0	63
SDS-V3-plasma-0_Cluster_1352_sequences=63	0	63
SDS-V3-plasma-7_Cluster_259_sequences=63	14	63
SDS-V3-plasma-7_Cluster_26_sequences=63	14	63
SDS-V3-plasma-7_Cluster_351_sequences=63	14	63
SDS-V3-plasma-8_Cluster_3543_sequences=63	16	63
SDS-V3-plasma-8_Cluster_3395_sequences=63	16	63
SDS-V3-plasma-24_Cluster_1075_sequences=63	124	63
SDS-V3-plasma-24_Cluster_687_sequences=63	124	63
SDS-V3-plasma-24_Cluster_718_sequences=63	124	63
SDS-V3-plasma-24_Cluster_762_sequences=63	124	63
SDS-V3-plasma-24_Cluster_893_sequences=63	124	63
SDS-V3-plasma-24_Cluster_961_sequences=63	124	63
SDS-V3-plasma-27_Cluster_874_sequences=63	131	63
SDS-V3-plasma-27_Cluster_138_sequences=63	131	63
SDS-V3-plasma-45_Cluster_4062_sequences=63	282	63
SDS-V3-plasma-45_Cluster_1018_sequences=63	282	63
SDS-V3-plasma-45_Cluster_1289_sequences=63	282	63
SDS-V3-plasma-45_Cluster_7740_sequences=63	282	63
SDS-V3-plasma-45_Cluster_185_sequences=63	282	63

SDS-V3-plasma-45_Cluster_2979_sequences=63	282	63
SDS-V3-plasma-45_Cluster_3639_sequences=63	282	63
SDS-V3-plasma-45_Cluster_1359_sequences=63	282	63
SDS-V3-plasma-45_Cluster_5527_sequences=63	282	63
SDS-V3-plasma-45_Cluster_1825_sequences=63	282	63
SDS-V3-plasma-45_Cluster_17248_sequences=63	282	63
SDS-V3-plasma-46_Cluster_1431_sequences=63	286	63
SDS-V3-plasma-46_Cluster_527_sequences=63	286	63
SDS-V3-plasma-67_Cluster_911_sequences=63	504	63
SDS-V3-plasma-67_Cluster_6626_sequences=63	504	63
SDS-V3-plasma-67_Cluster_5627_sequences=63	504	63
SDS-V3-plasma-67_Cluster_2873_sequences=63	504	63
SDS-V3-plasma-67_Cluster_4430_sequences=63	504	63
SDS-V3-plasma-67_Cluster_9120_sequences=63	504	63
SDS-V3-plasma-67_Cluster_400_sequences=63	504	63
SDS-V3-plasma-67_Cluster_8482_sequences=63	504	63
SDS-V3-plasma-67_Cluster_207_sequences=63	504	63
SDS-V3-plasma-67_Cluster_6373_sequences=63	504	63
SDS-V3-plasma-67_Cluster_1022_sequences=63	504	63
SDS-V3-plasma-67_Cluster_9672_sequences=63	504	63
SDS-V3-plasma-67_Cluster_2580_sequences=63	504	63
SDS-V3-plasma-0_Cluster_103_sequences=62	0	62
SDS-V3-plasma-0_Cluster_1285_sequences=62	0	62
SDS-V3-plasma-0_Cluster_1764_sequences=62	0	62
SDS-V3-plasma-7_Cluster_406_sequences=62	14	62
SDS-V3-plasma-8_Cluster_2351_sequences=62	16	62
SDS-V3-plasma-24_Cluster_1344_sequences=62	124	62
SDS-V3-plasma-24_Cluster_1200_sequences=62	124	62
SDS-V3-plasma-24_Cluster_243_sequences=62	124	62
SDS-V3-plasma-27_Cluster_274_sequences=62	131	62
SDS-V3-plasma-27_Cluster_610_sequences=62	131	62
SDS-V3-plasma-27_Cluster_680_sequences=62	131	62
SDS-V3-plasma-27_Cluster_1187_sequences=62	131	62
SDS-V3-plasma-45_Cluster_5483_sequences=62	282	62
SDS-V3-plasma-45_Cluster_4627_sequences=62	282	62
SDS-V3-plasma-45_Cluster_2211_sequences=62	282	62
SDS-V3-plasma-45_Cluster_7118_sequences=62	282	62
SDS-V3-plasma-45_Cluster_5591_sequences=62	282	62
SDS-V3-plasma-45_Cluster_3589_sequences=62	282	62
SDS-V3-plasma-45_Cluster_3927_sequences=62	282	62
SDS-V3-plasma-45_Cluster_4598_sequences=62	282	62
SDS-V3-plasma-45_Cluster_2431_sequences=62	282	62
SDS-V3-plasma-45_Cluster_1182_sequences=62	282	62
SDS-V3-plasma-45_Cluster_1896_sequences=62	282	62
SDS-V3-plasma-45_Cluster_8635_sequences=62	282	62

SDS-V3-plasma-45_Cluster_3166_sequences=62	282	62
SDS-V3-plasma-45_Cluster_2805_sequences=62	282	62
SDS-V3-plasma-45_Cluster_1823_sequences=62	282	62
SDS-V3-plasma-45_Cluster_9385_sequences=62	282	62
SDS-V3-plasma-45_Cluster_12100_sequences=62	282	62
SDS-V3-plasma-46_Cluster_1062_sequences=62	286	62
SDS-V3-plasma-67_Cluster_2479_sequences=62	504	62
SDS-V3-plasma-67_Cluster_377_sequences=62	504	62
SDS-V3-plasma-67_Cluster_5775_sequences=62	504	62
SDS-V3-plasma-67_Cluster_3652_sequences=62	504	62
SDS-V3-plasma-67_Cluster_4672_sequences=62	504	62
SDS-V3-plasma-67_Cluster_2904_sequences=62	504	62
SDS-V3-plasma-67_Cluster_4187_sequences=62	504	62
SDS-V3-plasma-0_Cluster_1238_sequences=61	0	61
SDS-V3-plasma-0_Cluster_2818_sequences=61	0	61
SDS-V3-plasma-0_Cluster_4387_sequences=61	0	61
SDS-V3-plasma-0_Cluster_3200_sequences=61	0	61
SDS-V3-plasma-0_Cluster_1363_sequences=61	0	61
SDS-V3-plasma-8_Cluster_2627_sequences=61	16	61
SDS-V3-plasma-8_Cluster_2634_sequences=61	16	61
SDS-V3-plasma-8_Cluster_2783_sequences=61	16	61
SDS-V3-plasma-24_Cluster_580_sequences=61	124	61
SDS-V3-plasma-24_Cluster_173_sequences=61	124	61
SDS-V3-plasma-27_Cluster_271_sequences=61	131	61
SDS-V3-plasma-27_Cluster_357_sequences=61	131	61
SDS-V3-plasma-45_Cluster_1004_sequences=61	282	61
SDS-V3-plasma-45_Cluster_1304_sequences=61	282	61
SDS-V3-plasma-45_Cluster_4057_sequences=61	282	61
SDS-V3-plasma-45_Cluster_1161_sequences=61	282	61
SDS-V3-plasma-45_Cluster_6376_sequences=61	282	61
SDS-V3-plasma-45_Cluster_6013_sequences=61	282	61
SDS-V3-plasma-45_Cluster_3929_sequences=61	282	61
SDS-V3-plasma-45_Cluster_3807_sequences=61	282	61
SDS-V3-plasma-45_Cluster_2952_sequences=61	282	61
SDS-V3-plasma-45_Cluster_2458_sequences=61	282	61
SDS-V3-plasma-45_Cluster_386_sequences=61	282	61
SDS-V3-plasma-45_Cluster_692_sequences=61	282	61
SDS-V3-plasma-45_Cluster_6911_sequences=61	282	61
SDS-V3-plasma-46_Cluster_2280_sequences=61	286	61
SDS-V3-plasma-46_Cluster_1333_sequences=61	286	61
SDS-V3-plasma-46_Cluster_4052_sequences=61	286	61
SDS-V3-plasma-46_Cluster_786_sequences=61	286	61
SDS-V3-plasma-67_Cluster_2290_sequences=61	504	61
SDS-V3-plasma-67_Cluster_950_sequences=61	504	61
SDS-V3-plasma-67_Cluster_3908_sequences=61	504	61

SDS-V3-plasma-67_Cluster_3577_sequences=61	504	61
SDS-V3-plasma-67_Cluster_4101_sequences=61	504	61
SDS-V3-plasma-67_Cluster_2662_sequences=61	504	61
SDS-V3-plasma-67_Cluster_4954_sequences=61	504	61
SDS-V3-plasma-67_Cluster_3802_sequences=61	504	61
SDS-V3-plasma-67_Cluster_4670_sequences=61	504	61
SDS-V3-plasma-67_Cluster_2085_sequences=61	504	61
SDS-V3-plasma-0_Cluster_2742_sequences=60	0	60
SDS-V3-plasma-0_Cluster_69_sequences=60	0	60
SDS-V3-plasma-0_Cluster_1616_sequences=60	0	60
SDS-V3-plasma-0_Cluster_42_sequences=60	0	60
SDS-V3-plasma-0_Cluster_5158_sequences=60	0	60
SDS-V3-plasma-0_Cluster_34_sequences=60	0	60
SDS-V3-plasma-24_Cluster_139_sequences=60	124	60
SDS-V3-plasma-24_Cluster_267_sequences=60	124	60
SDS-V3-plasma-24_Cluster_458_sequences=60	124	60
SDS-V3-plasma-24_Cluster_565_sequences=60	124	60
SDS-V3-plasma-24_Cluster_98_sequences=60	124	60
SDS-V3-plasma-27_Cluster_205_sequences=60	131	60
SDS-V3-plasma-27_Cluster_29_sequences=60	131	60
SDS-V3-plasma-27_Cluster_58_sequences=60	131	60
SDS-V3-plasma-45_Cluster_1946_sequences=60	282	60
SDS-V3-plasma-45_Cluster_6343_sequences=60	282	60
SDS-V3-plasma-45_Cluster_1115_sequences=60	282	60
SDS-V3-plasma-45_Cluster_3249_sequences=60	282	60
SDS-V3-plasma-45_Cluster_306_sequences=60	282	60
SDS-V3-plasma-45_Cluster_5582_sequences=60	282	60
SDS-V3-plasma-45_Cluster_4117_sequences=60	282	60
SDS-V3-plasma-45_Cluster_2250_sequences=60	282	60
SDS-V3-plasma-45_Cluster_6094_sequences=60	282	60
SDS-V3-plasma-45_Cluster_1350_sequences=60	282	60
SDS-V3-plasma-45_Cluster_7680_sequences=60	282	60
SDS-V3-plasma-45_Cluster_6006_sequences=60	282	60
SDS-V3-plasma-46_Cluster_982_sequences=60	286	60
SDS-V3-plasma-46_Cluster_779_sequences=60	286	60
SDS-V3-plasma-46_Cluster_549_sequences=60	286	60
SDS-V3-plasma-46_Cluster_2039_sequences=60	286	60
SDS-V3-plasma-46_Cluster_2653_sequences=60	286	60
SDS-V3-plasma-67_Cluster_2774_sequences=60	504	60
SDS-V3-plasma-67_Cluster_2842_sequences=60	504	60
SDS-V3-plasma-67_Cluster_4207_sequences=60	504	60
SDS-V3-plasma-67_Cluster_4256_sequences=60	504	60
SDS-V3-plasma-0_Cluster_173_sequences=59	0	59
SDS-V3-plasma-0_Cluster_557_sequences=59	0	59
SDS-V3-plasma-0_Cluster_9414_sequences=59	0	59

SDS-V3-plasma-0_Cluster_2453_sequences=59	0	59
SDS-V3-plasma-8_Cluster_2363_sequences=59	16	59
SDS-V3-plasma-8_Cluster_3103_sequences=59	16	59
SDS-V3-plasma-24_Cluster_386_sequences=59	124	59
SDS-V3-plasma-24_Cluster_482_sequences=59	124	59
SDS-V3-plasma-24_Cluster_77_sequences=59	124	59
SDS-V3-plasma-24_Cluster_7_sequences=59	124	59
SDS-V3-plasma-27_Cluster_36_sequences=59	131	59
SDS-V3-plasma-27_Cluster_782_sequences=59	131	59
SDS-V3-plasma-27_Cluster_101_sequences=59	131	59
SDS-V3-plasma-27_Cluster_123_sequences=59	131	59
SDS-V3-plasma-27_Cluster_130_sequences=59	131	59
SDS-V3-plasma-45_Cluster_7086_sequences=59	282	59
SDS-V3-plasma-45_Cluster_6702_sequences=59	282	59
SDS-V3-plasma-45_Cluster_3191_sequences=59	282	59
SDS-V3-plasma-45_Cluster_7856_sequences=59	282	59
SDS-V3-plasma-45_Cluster_5949_sequences=59	282	59
SDS-V3-plasma-45_Cluster_4074_sequences=59	282	59
SDS-V3-plasma-45_Cluster_10574_sequences=59	282	59
SDS-V3-plasma-45_Cluster_1145_sequences=59	282	59
SDS-V3-plasma-45_Cluster_4910_sequences=59	282	59
SDS-V3-plasma-45_Cluster_7812_sequences=59	282	59
SDS-V3-plasma-45_Cluster_1175_sequences=59	282	59
SDS-V3-plasma-45_Cluster_10466_sequences=59	282	59
SDS-V3-plasma-45_Cluster_1991_sequences=59	282	59
SDS-V3-plasma-45_Cluster_3428_sequences=59	282	59
SDS-V3-plasma-45_Cluster_2804_sequences=59	282	59
SDS-V3-plasma-45_Cluster_12861_sequences=59	282	59
SDS-V3-plasma-45_Cluster_4122_sequences=59	282	59
SDS-V3-plasma-46_Cluster_171_sequences=59	286	59
SDS-V3-plasma-46_Cluster_1166_sequences=59	286	59
SDS-V3-plasma-46_Cluster_922_sequences=59	286	59
SDS-V3-plasma-67_Cluster_3637_sequences=59	504	59
SDS-V3-plasma-67_Cluster_1862_sequences=59	504	59
SDS-V3-plasma-67_Cluster_2490_sequences=59	504	59
SDS-V3-plasma-67_Cluster_6187_sequences=59	504	59
SDS-V3-plasma-67_Cluster_4223_sequences=59	504	59
SDS-V3-plasma-67_Cluster_3447_sequences=59	504	59
SDS-V3-plasma-67_Cluster_1059_sequences=59	504	59
SDS-V3-plasma-0_Cluster_2278_sequences=58	0	58
SDS-V3-plasma-0_Cluster_639_sequences=58	0	58
SDS-V3-plasma-0_Cluster_1335_sequences=58	0	58
SDS-V3-plasma-0_Cluster_1671_sequences=58	0	58
SDS-V3-plasma-0_Cluster_4755_sequences=58	0	58
SDS-V3-plasma-0_Cluster_547_sequences=58	0	58

SDS-V3-plasma-0_Cluster_704_sequences=58	0	58
SDS-V3-plasma-7_Cluster_988_sequences=58	14	58
SDS-V3-plasma-7_Cluster_335_sequences=58	14	58
SDS-V3-plasma-8_Cluster_2495_sequences=58	16	58
SDS-V3-plasma-8_Cluster_2374_sequences=58	16	58
SDS-V3-plasma-24_Cluster_500_sequences=58	124	58
SDS-V3-plasma-24_Cluster_99_sequences=58	124	58
SDS-V3-plasma-24_Cluster_1854_sequences=58	124	58
SDS-V3-plasma-27_Cluster_53_sequences=58	131	58
SDS-V3-plasma-45_Cluster_1398_sequences=58	282	58
SDS-V3-plasma-45_Cluster_3851_sequences=58	282	58
SDS-V3-plasma-45_Cluster_1179_sequences=58	282	58
SDS-V3-plasma-45_Cluster_3722_sequences=58	282	58
SDS-V3-plasma-45_Cluster_7083_sequences=58	282	58
SDS-V3-plasma-45_Cluster_3367_sequences=58	282	58
SDS-V3-plasma-45_Cluster_4135_sequences=58	282	58
SDS-V3-plasma-45_Cluster_4803_sequences=58	282	58
SDS-V3-plasma-45_Cluster_4054_sequences=58	282	58
SDS-V3-plasma-45_Cluster_7042_sequences=58	282	58
SDS-V3-plasma-45_Cluster_8138_sequences=58	282	58
SDS-V3-plasma-45_Cluster_3241_sequences=58	282	58
SDS-V3-plasma-45_Cluster_3964_sequences=58	282	58
SDS-V3-plasma-45_Cluster_1077_sequences=58	282	58
SDS-V3-plasma-45_Cluster_2941_sequences=58	282	58
SDS-V3-plasma-46_Cluster_1555_sequences=58	286	58
SDS-V3-plasma-46_Cluster_1449_sequences=58	286	58
SDS-V3-plasma-46_Cluster_4150_sequences=58	286	58
SDS-V3-plasma-46_Cluster_1252_sequences=58	286	58
SDS-V3-plasma-67_Cluster_4472_sequences=58	504	58
SDS-V3-plasma-67_Cluster_7790_sequences=58	504	58
SDS-V3-plasma-67_Cluster_5295_sequences=58	504	58
SDS-V3-plasma-67_Cluster_239_sequences=58	504	58
SDS-V3-plasma-67_Cluster_3124_sequences=58	504	58
SDS-V3-plasma-0_Cluster_342_sequences=57	0	57
SDS-V3-plasma-0_Cluster_1015_sequences=57	0	57
SDS-V3-plasma-0_Cluster_2435_sequences=57	0	57
SDS-V3-plasma-0_Cluster_2494_sequences=57	0	57
SDS-V3-plasma-0_Cluster_3617_sequences=57	0	57
SDS-V3-plasma-0_Cluster_511_sequences=57	0	57
SDS-V3-plasma-5_Cluster_112_sequences=57	9	57
SDS-V3-plasma-7_Cluster_378_sequences=57	14	57
SDS-V3-plasma-8_Cluster_71_sequences=57	16	57
SDS-V3-plasma-8_Cluster_2748_sequences=57	16	57
SDS-V3-plasma-8_Cluster_3017_sequences=57	16	57
SDS-V3-plasma-8_Cluster_2683_sequences=57	16	57

SDS-V3-plasma-24_Cluster_90_sequences=57	124	57
SDS-V3-plasma-24_Cluster_389_sequences=57	124	57
SDS-V3-plasma-24_Cluster_784_sequences=57	124	57
SDS-V3-plasma-24_Cluster_715_sequences=57	124	57
SDS-V3-plasma-24_Cluster_579_sequences=57	124	57
SDS-V3-plasma-27_Cluster_1016_sequences=57	131	57
SDS-V3-plasma-27_Cluster_121_sequences=57	131	57
SDS-V3-plasma-27_Cluster_373_sequences=57	131	57
SDS-V3-plasma-27_Cluster_497_sequences=57	131	57
SDS-V3-plasma-27_Cluster_688_sequences=57	131	57
SDS-V3-plasma-45_Cluster_1614_sequences=57	282	57
SDS-V3-plasma-45_Cluster_11128_sequences=57	282	57
SDS-V3-plasma-45_Cluster_6290_sequences=57	282	57
SDS-V3-plasma-45_Cluster_3974_sequences=57	282	57
SDS-V3-plasma-45_Cluster_2099_sequences=57	282	57
SDS-V3-plasma-45_Cluster_1796_sequences=57	282	57
SDS-V3-plasma-45_Cluster_2638_sequences=57	282	57
SDS-V3-plasma-45_Cluster_1557_sequences=57	282	57
SDS-V3-plasma-45_Cluster_643_sequences=57	282	57
SDS-V3-plasma-45_Cluster_923_sequences=57	282	57
SDS-V3-plasma-45_Cluster_223_sequences=57	282	57
SDS-V3-plasma-45_Cluster_7099_sequences=57	282	57
SDS-V3-plasma-46_Cluster_577_sequences=57	286	57
SDS-V3-plasma-46_Cluster_511_sequences=57	286	57
SDS-V3-plasma-46_Cluster_1240_sequences=57	286	57
SDS-V3-plasma-46_Cluster_2681_sequences=57	286	57
SDS-V3-plasma-67_Cluster_5620_sequences=57	504	57
SDS-V3-plasma-67_Cluster_11050_sequences=57	504	57
SDS-V3-plasma-67_Cluster_1995_sequences=57	504	57
SDS-V3-plasma-67_Cluster_224_sequences=57	504	57
SDS-V3-plasma-67_Cluster_11224_sequences=57	504	57
SDS-V3-plasma-67_Cluster_5499_sequences=57	504	57
SDS-V3-plasma-67_Cluster_244_sequences=57	504	57
SDS-V3-plasma-67_Cluster_447_sequences=57	504	57
SDS-V3-plasma-0_Cluster_318_sequences=56	0	56
SDS-V3-plasma-0_Cluster_4372_sequences=56	0	56
SDS-V3-plasma-0_Cluster_1499_sequences=56	0	56
SDS-V3-plasma-0_Cluster_1500_sequences=56	0	56
SDS-V3-plasma-0_Cluster_1832_sequences=56	0	56
SDS-V3-plasma-0_Cluster_3230_sequences=56	0	56
SDS-V3-plasma-0_Cluster_792_sequences=56	0	56
SDS-V3-plasma-0_Cluster_353_sequences=56	0	56
SDS-V3-plasma-0_Cluster_1118_sequences=56	0	56
SDS-V3-plasma-7_Cluster_1208_sequences=56	14	56
SDS-V3-plasma-8_Cluster_44_sequences=56	16	56

SDS-V3-plasma-8_Cluster_2267_sequences=56	16	56
SDS-V3-plasma-24_Cluster_1257_sequences=56	124	56
SDS-V3-plasma-24_Cluster_1296_sequences=56	124	56
SDS-V3-plasma-24_Cluster_204_sequences=56	124	56
SDS-V3-plasma-24_Cluster_544_sequences=56	124	56
SDS-V3-plasma-24_Cluster_14_sequences=56	124	56
SDS-V3-plasma-24_Cluster_1189_sequences=56	124	56
SDS-V3-plasma-24_Cluster_209_sequences=56	124	56
SDS-V3-plasma-27_Cluster_1332_sequences=56	131	56
SDS-V3-plasma-27_Cluster_251_sequences=56	131	56
SDS-V3-plasma-45_Cluster_4165_sequences=56	282	56
SDS-V3-plasma-45_Cluster_2714_sequences=56	282	56
SDS-V3-plasma-45_Cluster_6346_sequences=56	282	56
SDS-V3-plasma-45_Cluster_1445_sequences=56	282	56
SDS-V3-plasma-45_Cluster_8965_sequences=56	282	56
SDS-V3-plasma-45_Cluster_7178_sequences=56	282	56
SDS-V3-plasma-45_Cluster_3445_sequences=56	282	56
SDS-V3-plasma-45_Cluster_4292_sequences=56	282	56
SDS-V3-plasma-45_Cluster_7696_sequences=56	282	56
SDS-V3-plasma-45_Cluster_5938_sequences=56	282	56
SDS-V3-plasma-45_Cluster_18733_sequences=56	282	56
SDS-V3-plasma-45_Cluster_3127_sequences=56	282	56
SDS-V3-plasma-45_Cluster_9172_sequences=56	282	56
SDS-V3-plasma-45_Cluster_799_sequences=56	282	56
SDS-V3-plasma-45_Cluster_4887_sequences=56	282	56
SDS-V3-plasma-45_Cluster_4615_sequences=56	282	56
SDS-V3-plasma-45_Cluster_2876_sequences=56	282	56
SDS-V3-plasma-45_Cluster_2452_sequences=56	282	56
SDS-V3-plasma-45_Cluster_3792_sequences=56	282	56
SDS-V3-plasma-45_Cluster_7149_sequences=56	282	56
SDS-V3-plasma-45_Cluster_8808_sequences=56	282	56
SDS-V3-plasma-46_Cluster_2925_sequences=56	286	56
SDS-V3-plasma-46_Cluster_1437_sequences=56	286	56
SDS-V3-plasma-46_Cluster_4614_sequences=56	286	56
SDS-V3-plasma-46_Cluster_1147_sequences=56	286	56
SDS-V3-plasma-46_Cluster_728_sequences=56	286	56
SDS-V3-plasma-46_Cluster_1156_sequences=56	286	56
SDS-V3-plasma-67_Cluster_1177_sequences=56	504	56
SDS-V3-plasma-67_Cluster_3520_sequences=56	504	56
SDS-V3-plasma-67_Cluster_5161_sequences=56	504	56
SDS-V3-plasma-67_Cluster_10489_sequences=56	504	56
SDS-V3-plasma-67_Cluster_10733_sequences=56	504	56
SDS-V3-plasma-67_Cluster_2294_sequences=56	504	56
SDS-V3-plasma-67_Cluster_9886_sequences=56	504	56
SDS-V3-plasma-67_Cluster_287_sequences=56	504	56

SDS-V3-plasma-67_Cluster_6133_sequences=56	504	56
SDS-V3-PBMC-5_Cluster_35_sequences=55	9	55
SDS-V3-plasma-0_Cluster_392_sequences=55	0	55
SDS-V3-plasma-0_Cluster_5466_sequences=55	0	55
SDS-V3-plasma-0_Cluster_853_sequences=55	0	55
SDS-V3-plasma-0_Cluster_1246_sequences=55	0	55
SDS-V3-plasma-0_Cluster_227_sequences=55	0	55
SDS-V3-plasma-24_Cluster_1406_sequences=55	124	55
SDS-V3-plasma-24_Cluster_1639_sequences=55	124	55
SDS-V3-plasma-24_Cluster_310_sequences=55	124	55
SDS-V3-plasma-24_Cluster_723_sequences=55	124	55
SDS-V3-plasma-24_Cluster_1242_sequences=55	124	55
SDS-V3-plasma-27_Cluster_537_sequences=55	131	55
SDS-V3-plasma-27_Cluster_932_sequences=55	131	55
SDS-V3-plasma-45_Cluster_2917_sequences=55	282	55
SDS-V3-plasma-45_Cluster_6863_sequences=55	282	55
SDS-V3-plasma-45_Cluster_1858_sequences=55	282	55
SDS-V3-plasma-45_Cluster_1323_sequences=55	282	55
SDS-V3-plasma-45_Cluster_4596_sequences=55	282	55
SDS-V3-plasma-45_Cluster_5854_sequences=55	282	55
SDS-V3-plasma-45_Cluster_3610_sequences=55	282	55
SDS-V3-plasma-45_Cluster_4432_sequences=55	282	55
SDS-V3-plasma-45_Cluster_954_sequences=55	282	55
SDS-V3-plasma-45_Cluster_1237_sequences=55	282	55
SDS-V3-plasma-45_Cluster_6479_sequences=55	282	55
SDS-V3-plasma-45_Cluster_899_sequences=55	282	55
SDS-V3-plasma-45_Cluster_655_sequences=55	282	55
SDS-V3-plasma-45_Cluster_5453_sequences=55	282	55
SDS-V3-plasma-45_Cluster_278_sequences=55	282	55
SDS-V3-plasma-45_Cluster_7046_sequences=55	282	55
SDS-V3-plasma-45_Cluster_5638_sequences=55	282	55
SDS-V3-plasma-46_Cluster_283_sequences=55	286	55
SDS-V3-plasma-46_Cluster_589_sequences=55	286	55
SDS-V3-plasma-46_Cluster_4202_sequences=55	286	55
SDS-V3-plasma-46_Cluster_761_sequences=55	286	55
SDS-V3-plasma-67_Cluster_4658_sequences=55	504	55
SDS-V3-plasma-67_Cluster_2095_sequences=55	504	55
SDS-V3-plasma-67_Cluster_2642_sequences=55	504	55
SDS-V3-plasma-67_Cluster_5836_sequences=55	504	55
SDS-V3-plasma-67_Cluster_6567_sequences=55	504	55
SDS-V3-plasma-67_Cluster_1166_sequences=55	504	55
SDS-V3-plasma-67_Cluster_8930_sequences=55	504	55
SDS-V3-plasma-0_Cluster_199_sequences=54	0	54
SDS-V3-plasma-0_Cluster_3175_sequences=54	0	54
SDS-V3-plasma-0_Cluster_2137_sequences=54	0	54

SDS-V3-plasma-0_Cluster_1723_sequences=54	0	54
SDS-V3-plasma-0_Cluster_397_sequences=54	0	54
SDS-V3-plasma-0_Cluster_777_sequences=54	0	54
SDS-V3-plasma-0_Cluster_1062_sequences=54	0	54
SDS-V3-plasma-0_Cluster_1255_sequences=54	0	54
SDS-V3-plasma-5_Cluster_144_sequences=54	9	54
SDS-V3-plasma-5_Cluster_6_sequences=54	9	54
SDS-V3-plasma-8_Cluster_3007_sequences=54	16	54
SDS-V3-plasma-24_Cluster_61_sequences=54	124	54
SDS-V3-plasma-27_Cluster_41_sequences=54	131	54
SDS-V3-plasma-27_Cluster_612_sequences=54	131	54
SDS-V3-plasma-27_Cluster_700_sequences=54	131	54
SDS-V3-plasma-27_Cluster_13_sequences=54	131	54
SDS-V3-plasma-45_Cluster_1656_sequences=54	282	54
SDS-V3-plasma-45_Cluster_4859_sequences=54	282	54
SDS-V3-plasma-45_Cluster_4188_sequences=54	282	54
SDS-V3-plasma-45_Cluster_8453_sequences=54	282	54
SDS-V3-plasma-45_Cluster_3845_sequences=54	282	54
SDS-V3-plasma-45_Cluster_6685_sequences=54	282	54
SDS-V3-plasma-45_Cluster_3498_sequences=54	282	54
SDS-V3-plasma-45_Cluster_3093_sequences=54	282	54
SDS-V3-plasma-45_Cluster_9159_sequences=54	282	54
SDS-V3-plasma-45_Cluster_1575_sequences=54	282	54
SDS-V3-plasma-45_Cluster_4485_sequences=54	282	54
SDS-V3-plasma-45_Cluster_12223_sequences=54	282	54
SDS-V3-plasma-45_Cluster_2310_sequences=54	282	54
SDS-V3-plasma-45_Cluster_8948_sequences=54	282	54
SDS-V3-plasma-45_Cluster_1590_sequences=54	282	54
SDS-V3-plasma-45_Cluster_5187_sequences=54	282	54
SDS-V3-plasma-45_Cluster_4080_sequences=54	282	54
SDS-V3-plasma-45_Cluster_2359_sequences=54	282	54
SDS-V3-plasma-45_Cluster_4273_sequences=54	282	54
SDS-V3-plasma-46_Cluster_1355_sequences=54	286	54
SDS-V3-plasma-46_Cluster_797_sequences=54	286	54
SDS-V3-plasma-67_Cluster_14321_sequences=54	504	54
SDS-V3-plasma-67_Cluster_3569_sequences=54	504	54
SDS-V3-plasma-67_Cluster_570_sequences=54	504	54
SDS-V3-plasma-67_Cluster_2779_sequences=54	504	54
SDS-V3-plasma-67_Cluster_550_sequences=54	504	54
SDS-V3-plasma-67_Cluster_9318_sequences=54	504	54
SDS-V3-plasma-67_Cluster_4102_sequences=54	504	54
SDS-V3-plasma-0_Cluster_5744_sequences=53	0	53
SDS-V3-plasma-0_Cluster_591_sequences=53	0	53
SDS-V3-plasma-0_Cluster_4027_sequences=53	0	53
SDS-V3-plasma-0_Cluster_587_sequences=53	0	53

SDS-V3-plasma-0_Cluster_2983_sequences=53	0	53
SDS-V3-plasma-0_Cluster_1254_sequences=53	0	53
SDS-V3-plasma-0_Cluster_2208_sequences=53	0	53
SDS-V3-plasma-7_Cluster_400_sequences=53	14	53
SDS-V3-plasma-7_Cluster_461_sequences=53	14	53
SDS-V3-plasma-8_Cluster_2478_sequences=53	16	53
SDS-V3-plasma-8_Cluster_2408_sequences=53	16	53
SDS-V3-plasma-24_Cluster_1203_sequences=53	124	53
SDS-V3-plasma-24_Cluster_1405_sequences=53	124	53
SDS-V3-plasma-24_Cluster_1763_sequences=53	124	53
SDS-V3-plasma-24_Cluster_198_sequences=53	124	53
SDS-V3-plasma-27_Cluster_640_sequences=53	131	53
SDS-V3-plasma-27_Cluster_714_sequences=53	131	53
SDS-V3-plasma-27_Cluster_604_sequences=53	131	53
SDS-V3-plasma-27_Cluster_353_sequences=53	131	53
SDS-V3-plasma-27_Cluster_124_sequences=53	131	53
SDS-V3-plasma-27_Cluster_992_sequences=53	131	53
SDS-V3-plasma-27_Cluster_339_sequences=53	131	53
SDS-V3-plasma-27_Cluster_12_sequences=53	131	53
SDS-V3-plasma-45_Cluster_8191_sequences=53	282	53
SDS-V3-plasma-45_Cluster_8076_sequences=53	282	53
SDS-V3-plasma-45_Cluster_6842_sequences=53	282	53
SDS-V3-plasma-45_Cluster_1968_sequences=53	282	53
SDS-V3-plasma-45_Cluster_8537_sequences=53	282	53
SDS-V3-plasma-45_Cluster_7910_sequences=53	282	53
SDS-V3-plasma-45_Cluster_10820_sequences=53	282	53
SDS-V3-plasma-45_Cluster_9929_sequences=53	282	53
SDS-V3-plasma-45_Cluster_4714_sequences=53	282	53
SDS-V3-plasma-45_Cluster_2951_sequences=53	282	53
SDS-V3-plasma-45_Cluster_1639_sequences=53	282	53
SDS-V3-plasma-45_Cluster_824_sequences=53	282	53
SDS-V3-plasma-45_Cluster_3662_sequences=53	282	53
SDS-V3-plasma-45_Cluster_939_sequences=53	282	53
SDS-V3-plasma-45_Cluster_5564_sequences=53	282	53
SDS-V3-plasma-45_Cluster_65683_sequences=53	282	53
SDS-V3-plasma-45_Cluster_4470_sequences=53	282	53
SDS-V3-plasma-45_Cluster_5599_sequences=53	282	53
SDS-V3-plasma-45_Cluster_3033_sequences=53	282	53
SDS-V3-plasma-45_Cluster_17094_sequences=53	282	53
SDS-V3-plasma-45_Cluster_3526_sequences=53	282	53
SDS-V3-plasma-45_Cluster_11749_sequences=53	282	53
SDS-V3-plasma-45_Cluster_5974_sequences=53	282	53
SDS-V3-plasma-45_Cluster_793_sequences=53	282	53
SDS-V3-plasma-46_Cluster_2002_sequences=53	286	53
SDS-V3-plasma-46_Cluster_3966_sequences=53	286	53

SDS-V3-plasma-46_Cluster_196_sequences=53	286	53
SDS-V3-plasma-67_Cluster_5440_sequences=53	504	53
SDS-V3-plasma-67_Cluster_6024_sequences=53	504	53
SDS-V3-plasma-67_Cluster_6521_sequences=53	504	53
SDS-V3-plasma-67_Cluster_387_sequences=53	504	53
SDS-V3-plasma-67_Cluster_7185_sequences=53	504	53
SDS-V3-plasma-67_Cluster_2494_sequences=53	504	53
SDS-V3-plasma-67_Cluster_3474_sequences=53	504	53
SDS-V3-plasma-67_Cluster_45_sequences=53	504	53
SDS-V3-plasma-67_Cluster_4431_sequences=53	504	53
SDS-V3-plasma-67_Cluster_9476_sequences=53	504	53
SDS-V3-plasma-67_Cluster_2771_sequences=53	504	53
SDS-V3-plasma-67_Cluster_10438_sequences=53	504	53
SDS-V3-plasma-0_Cluster_475_sequences=52	0	52
SDS-V3-plasma-0_Cluster_131_sequences=52	0	52
SDS-V3-plasma-0_Cluster_1117_sequences=52	0	52
SDS-V3-plasma-0_Cluster_1151_sequences=52	0	52
SDS-V3-plasma-0_Cluster_5216_sequences=52	0	52
SDS-V3-plasma-0_Cluster_564_sequences=52	0	52
SDS-V3-plasma-0_Cluster_2912_sequences=52	0	52
SDS-V3-plasma-0_Cluster_2633_sequences=52	0	52
SDS-V3-plasma-8_Cluster_2279_sequences=52	16	52
SDS-V3-plasma-8_Cluster_3070_sequences=52	16	52
SDS-V3-plasma-24_Cluster_284_sequences=52	124	52
SDS-V3-plasma-24_Cluster_286_sequences=52	124	52
SDS-V3-plasma-24_Cluster_974_sequences=52	124	52
SDS-V3-plasma-24_Cluster_2081_sequences=52	124	52
SDS-V3-plasma-24_Cluster_448_sequences=52	124	52
SDS-V3-plasma-27_Cluster_1168_sequences=52	131	52
SDS-V3-plasma-27_Cluster_169_sequences=52	131	52
SDS-V3-plasma-27_Cluster_1883_sequences=52	131	52
SDS-V3-plasma-27_Cluster_194_sequences=52	131	52
SDS-V3-plasma-27_Cluster_2741_sequences=52	131	52
SDS-V3-plasma-27_Cluster_367_sequences=52	131	52
SDS-V3-plasma-27_Cluster_421_sequences=52	131	52
SDS-V3-plasma-27_Cluster_777_sequences=52	131	52
SDS-V3-plasma-27_Cluster_1122_sequences=52	131	52
SDS-V3-plasma-45_Cluster_730_sequences=52	282	52
SDS-V3-plasma-45_Cluster_7599_sequences=52	282	52
SDS-V3-plasma-45_Cluster_380_sequences=52	282	52
SDS-V3-plasma-45_Cluster_1091_sequences=52	282	52
SDS-V3-plasma-45_Cluster_1615_sequences=52	282	52
SDS-V3-plasma-45_Cluster_4293_sequences=52	282	52
SDS-V3-plasma-45_Cluster_4879_sequences=52	282	52
SDS-V3-plasma-45_Cluster_8886_sequences=52	282	52

SDS-V3-plasma-45_Cluster_7306_sequences=52	282	52
SDS-V3-plasma-45_Cluster_5826_sequences=52	282	52
SDS-V3-plasma-45_Cluster_1971_sequences=52	282	52
SDS-V3-plasma-45_Cluster_3116_sequences=52	282	52
SDS-V3-plasma-45_Cluster_2563_sequences=52	282	52
SDS-V3-plasma-45_Cluster_2083_sequences=52	282	52
SDS-V3-plasma-45_Cluster_119_sequences=52	282	52
SDS-V3-plasma-45_Cluster_5505_sequences=52	282	52
SDS-V3-plasma-45_Cluster_2206_sequences=52	282	52
SDS-V3-plasma-45_Cluster_2202_sequences=52	282	52
SDS-V3-plasma-45_Cluster_2826_sequences=52	282	52
SDS-V3-plasma-46_Cluster_2496_sequences=52	286	52
SDS-V3-plasma-46_Cluster_842_sequences=52	286	52
SDS-V3-plasma-46_Cluster_2065_sequences=52	286	52
SDS-V3-plasma-67_Cluster_7489_sequences=52	504	52
SDS-V3-plasma-67_Cluster_6111_sequences=52	504	52
SDS-V3-plasma-67_Cluster_1058_sequences=52	504	52
SDS-V3-plasma-67_Cluster_9874_sequences=52	504	52
SDS-V3-plasma-67_Cluster_5999_sequences=52	504	52
SDS-V3-plasma-67_Cluster_203_sequences=52	504	52
SDS-V3-plasma-67_Cluster_2903_sequences=52	504	52
SDS-V3-plasma-0_Cluster_1404_sequences=51	0	51
SDS-V3-plasma-0_Cluster_721_sequences=51	0	51
SDS-V3-plasma-0_Cluster_2583_sequences=51	0	51
SDS-V3-plasma-0_Cluster_603_sequences=51	0	51
SDS-V3-plasma-0_Cluster_1979_sequences=51	0	51
SDS-V3-plasma-0_Cluster_1043_sequences=51	0	51
SDS-V3-plasma-0_Cluster_2888_sequences=51	0	51
SDS-V3-plasma-0_Cluster_419_sequences=51	0	51
SDS-V3-plasma-0_Cluster_2965_sequences=51	0	51
SDS-V3-plasma-0_Cluster_4730_sequences=51	0	51
SDS-V3-plasma-0_Cluster_1936_sequences=51	0	51
SDS-V3-plasma-0_Cluster_1727_sequences=51	0	51
SDS-V3-plasma-8_Cluster_2798_sequences=51	16	51
SDS-V3-plasma-8_Cluster_2381_sequences=51	16	51
SDS-V3-plasma-24_Cluster_120_sequences=51	124	51
SDS-V3-plasma-24_Cluster_1247_sequences=51	124	51
SDS-V3-plasma-24_Cluster_54_sequences=51	124	51
SDS-V3-plasma-24_Cluster_89_sequences=51	124	51
SDS-V3-plasma-24_Cluster_523_sequences=51	124	51
SDS-V3-plasma-24_Cluster_30_sequences=51	124	51
SDS-V3-plasma-24_Cluster_925_sequences=51	124	51
SDS-V3-plasma-27_Cluster_1133_sequences=51	131	51
SDS-V3-plasma-27_Cluster_583_sequences=51	131	51
SDS-V3-plasma-27_Cluster_24_sequences=51	131	51

SDS-V3-plasma-27_Cluster_49_sequences=51	131	51
SDS-V3-plasma-45_Cluster_9796_sequences=51	282	51
SDS-V3-plasma-45_Cluster_5474_sequences=51	282	51
SDS-V3-plasma-45_Cluster_3144_sequences=51	282	51
SDS-V3-plasma-45_Cluster_1554_sequences=51	282	51
SDS-V3-plasma-45_Cluster_1706_sequences=51	282	51
SDS-V3-plasma-45_Cluster_2413_sequences=51	282	51
SDS-V3-plasma-45_Cluster_10580_sequences=51	282	51
SDS-V3-plasma-45_Cluster_1170_sequences=51	282	51
SDS-V3-plasma-45_Cluster_6814_sequences=51	282	51
SDS-V3-plasma-45_Cluster_6289_sequences=51	282	51
SDS-V3-plasma-45_Cluster_393_sequences=51	282	51
SDS-V3-plasma-45_Cluster_620_sequences=51	282	51
SDS-V3-plasma-45_Cluster_1202_sequences=51	282	51
SDS-V3-plasma-45_Cluster_2237_sequences=51	282	51
SDS-V3-plasma-45_Cluster_1580_sequences=51	282	51
SDS-V3-plasma-45_Cluster_59_sequences=51	282	51
SDS-V3-plasma-45_Cluster_547_sequences=51	282	51
SDS-V3-plasma-45_Cluster_2608_sequences=51	282	51
SDS-V3-plasma-45_Cluster_5732_sequences=51	282	51
SDS-V3-plasma-46_Cluster_946_sequences=51	286	51
SDS-V3-plasma-67_Cluster_9991_sequences=51	504	51
SDS-V3-plasma-67_Cluster_2878_sequences=51	504	51
SDS-V3-plasma-67_Cluster_4623_sequences=51	504	51
SDS-V3-plasma-67_Cluster_8480_sequences=51	504	51
SDS-V3-plasma-67_Cluster_11784_sequences=51	504	51
SDS-V3-plasma-67_Cluster_5051_sequences=51	504	51
SDS-V3-plasma-67_Cluster_12243_sequences=51	504	51
SDS-V3-plasma-67_Cluster_5214_sequences=51	504	51
SDS-V3-plasma-0_Cluster_726_sequences=50	0	50
SDS-V3-plasma-0_Cluster_249_sequences=50	0	50
SDS-V3-plasma-0_Cluster_2394_sequences=50	0	50
SDS-V3-plasma-0_Cluster_1080_sequences=50	0	50
SDS-V3-plasma-0_Cluster_1823_sequences=50	0	50
SDS-V3-plasma-7_Cluster_769_sequences=50	14	50
SDS-V3-plasma-7_Cluster_185_sequences=50	14	50
SDS-V3-plasma-7_Cluster_136_sequences=50	14	50
SDS-V3-plasma-8_Cluster_2782_sequences=50	16	50
SDS-V3-plasma-24_Cluster_549_sequences=50	124	50
SDS-V3-plasma-24_Cluster_566_sequences=50	124	50
SDS-V3-plasma-24_Cluster_6_sequences=50	124	50
SDS-V3-plasma-24_Cluster_86_sequences=50	124	50
SDS-V3-plasma-24_Cluster_96_sequences=50	124	50
SDS-V3-plasma-24_Cluster_936_sequences=50	124	50
SDS-V3-plasma-24_Cluster_1762_sequences=50	124	50

SDS-V3-plasma-27_Cluster_355_sequences=50	131	50
SDS-V3-plasma-27_Cluster_1056_sequences=50	131	50
SDS-V3-plasma-27_Cluster_1345_sequences=50	131	50
SDS-V3-plasma-45_Cluster_69_sequences=50	282	50
SDS-V3-plasma-45_Cluster_770_sequences=50	282	50
SDS-V3-plasma-45_Cluster_1118_sequences=50	282	50
SDS-V3-plasma-45_Cluster_4084_sequences=50	282	50
SDS-V3-plasma-45_Cluster_4913_sequences=50	282	50
SDS-V3-plasma-45_Cluster_2572_sequences=50	282	50
SDS-V3-plasma-45_Cluster_1282_sequences=50	282	50
SDS-V3-plasma-45_Cluster_14441_sequences=50	282	50
SDS-V3-plasma-45_Cluster_1721_sequences=50	282	50
SDS-V3-plasma-45_Cluster_3163_sequences=50	282	50
SDS-V3-plasma-45_Cluster_10297_sequences=50	282	50
SDS-V3-plasma-45_Cluster_11628_sequences=50	282	50
SDS-V3-plasma-45_Cluster_2152_sequences=50	282	50
SDS-V3-plasma-45_Cluster_1358_sequences=50	282	50
SDS-V3-plasma-45_Cluster_866_sequences=50	282	50
SDS-V3-plasma-45_Cluster_816_sequences=50	282	50
SDS-V3-plasma-46_Cluster_1705_sequences=50	286	50
SDS-V3-plasma-46_Cluster_2569_sequences=50	286	50
SDS-V3-plasma-46_Cluster_540_sequences=50	286	50
SDS-V3-plasma-46_Cluster_1582_sequences=50	286	50
SDS-V3-plasma-46_Cluster_2438_sequences=50	286	50
SDS-V3-plasma-46_Cluster_2904_sequences=50	286	50
SDS-V3-plasma-46_Cluster_1381_sequences=50	286	50
SDS-V3-plasma-46_Cluster_1954_sequences=50	286	50
SDS-V3-plasma-46_Cluster_2333_sequences=50	286	50
SDS-V3-plasma-46_Cluster_1640_sequences=50	286	50
SDS-V3-plasma-46_Cluster_21_sequences=50	286	50
SDS-V3-plasma-67_Cluster_11436_sequences=50	504	50
SDS-V3-plasma-67_Cluster_1342_sequences=50	504	50
SDS-V3-plasma-67_Cluster_18357_sequences=50	504	50
SDS-V3-plasma-67_Cluster_706_sequences=50	504	50
SDS-V3-plasma-67_Cluster_12183_sequences=50	504	50
SDS-V3-plasma-67_Cluster_2130_sequences=50	504	50
SDS-V3-plasma-67_Cluster_1050_sequences=50	504	50
SDS-V3-plasma-67_Cluster_4477_sequences=50	504	50
SDS-V3-plasma-67_Cluster_576_sequences=50	504	50
SDS-V3-plasma-67_Cluster_2022_sequences=50	504	50
SDS-V3-plasma-0_Cluster_5183_sequences=49	0	49
SDS-V3-plasma-0_Cluster_1204_sequences=49	0	49
SDS-V3-plasma-0_Cluster_1345_sequences=49	0	49
SDS-V3-plasma-0_Cluster_1698_sequences=49	0	49
SDS-V3-plasma-0_Cluster_961_sequences=49	0	49

SDS-V3-plasma-0_Cluster_1494_sequences=49	0	49
SDS-V3-plasma-7_Cluster_279_sequences=49	14	49
SDS-V3-plasma-8_Cluster_2494_sequences=49	16	49
SDS-V3-plasma-24_Cluster_1546_sequences=49	124	49
SDS-V3-plasma-24_Cluster_463_sequences=49	124	49
SDS-V3-plasma-24_Cluster_306_sequences=49	124	49
SDS-V3-plasma-27_Cluster_1079_sequences=49	131	49
SDS-V3-plasma-27_Cluster_1272_sequences=49	131	49
SDS-V3-plasma-27_Cluster_1420_sequences=49	131	49
SDS-V3-plasma-27_Cluster_624_sequences=49	131	49
SDS-V3-plasma-27_Cluster_77_sequences=49	131	49
SDS-V3-plasma-27_Cluster_179_sequences=49	131	49
SDS-V3-plasma-45_Cluster_1810_sequences=49	282	49
SDS-V3-plasma-45_Cluster_6177_sequences=49	282	49
SDS-V3-plasma-45_Cluster_3597_sequences=49	282	49
SDS-V3-plasma-45_Cluster_1960_sequences=49	282	49
SDS-V3-plasma-45_Cluster_687_sequences=49	282	49
SDS-V3-plasma-45_Cluster_1809_sequences=49	282	49
SDS-V3-plasma-45_Cluster_131_sequences=49	282	49
SDS-V3-plasma-45_Cluster_7238_sequences=49	282	49
SDS-V3-plasma-45_Cluster_4726_sequences=49	282	49
SDS-V3-plasma-45_Cluster_4256_sequences=49	282	49
SDS-V3-plasma-45_Cluster_4563_sequences=49	282	49
SDS-V3-plasma-45_Cluster_1645_sequences=49	282	49
SDS-V3-plasma-45_Cluster_60_sequences=49	282	49
SDS-V3-plasma-45_Cluster_5414_sequences=49	282	49
SDS-V3-plasma-45_Cluster_10348_sequences=49	282	49
SDS-V3-plasma-46_Cluster_142_sequences=49	286	49
SDS-V3-plasma-46_Cluster_1565_sequences=49	286	49
SDS-V3-plasma-46_Cluster_1060_sequences=49	286	49
SDS-V3-plasma-46_Cluster_1206_sequences=49	286	49
SDS-V3-plasma-46_Cluster_601_sequences=49	286	49
SDS-V3-plasma-46_Cluster_3451_sequences=49	286	49
SDS-V3-plasma-67_Cluster_2885_sequences=49	504	49
SDS-V3-plasma-67_Cluster_1451_sequences=49	504	49
SDS-V3-plasma-67_Cluster_2895_sequences=49	504	49
SDS-V3-plasma-67_Cluster_1833_sequences=49	504	49
SDS-V3-plasma-67_Cluster_1263_sequences=49	504	49
SDS-V3-plasma-67_Cluster_3615_sequences=49	504	49
SDS-V3-plasma-67_Cluster_63_sequences=49	504	49
SDS-V3-plasma-67_Cluster_3687_sequences=49	504	49
SDS-V3-plasma-67_Cluster_2047_sequences=49	504	49
SDS-V3-plasma-67_Cluster_2359_sequences=49	504	49
SDS-V3-plasma-0_Cluster_715_sequences=48	0	48
SDS-V3-plasma-0_Cluster_2345_sequences=48	0	48

SDS-V3-plasma-0_Cluster_2413_sequences=48	0	48
SDS-V3-plasma-0_Cluster_2096_sequences=48	0	48
SDS-V3-plasma-0_Cluster_285_sequences=48	0	48
SDS-V3-plasma-0_Cluster_7115_sequences=48	0	48
SDS-V3-plasma-7_Cluster_1207_sequences=48	14	48
SDS-V3-plasma-7_Cluster_177_sequences=48	14	48
SDS-V3-plasma-7_Cluster_518_sequences=48	14	48
SDS-V3-plasma-7_Cluster_1014_sequences=48	14	48
SDS-V3-plasma-7_Cluster_99_sequences=48	14	48
SDS-V3-plasma-8_Cluster_2651_sequences=48	16	48
SDS-V3-plasma-24_Cluster_1269_sequences=48	124	48
SDS-V3-plasma-24_Cluster_2037_sequences=48	124	48
SDS-V3-plasma-24_Cluster_385_sequences=48	124	48
SDS-V3-plasma-24_Cluster_483_sequences=48	124	48
SDS-V3-plasma-24_Cluster_755_sequences=48	124	48
SDS-V3-plasma-27_Cluster_129_sequences=48	131	48
SDS-V3-plasma-27_Cluster_2749_sequences=48	131	48
SDS-V3-plasma-27_Cluster_224_sequences=48	131	48
SDS-V3-plasma-45_Cluster_11667_sequences=48	282	48
SDS-V3-plasma-45_Cluster_10506_sequences=48	282	48
SDS-V3-plasma-45_Cluster_8394_sequences=48	282	48
SDS-V3-plasma-45_Cluster_7694_sequences=48	282	48
SDS-V3-plasma-45_Cluster_125_sequences=48	282	48
SDS-V3-plasma-45_Cluster_1303_sequences=48	282	48
SDS-V3-plasma-45_Cluster_3294_sequences=48	282	48
SDS-V3-plasma-45_Cluster_8449_sequences=48	282	48
SDS-V3-plasma-45_Cluster_2620_sequences=48	282	48
SDS-V3-plasma-45_Cluster_8238_sequences=48	282	48
SDS-V3-plasma-45_Cluster_291_sequences=48	282	48
SDS-V3-plasma-45_Cluster_1256_sequences=48	282	48
SDS-V3-plasma-45_Cluster_6302_sequences=48	282	48
SDS-V3-plasma-45_Cluster_10483_sequences=48	282	48
SDS-V3-plasma-45_Cluster_7898_sequences=48	282	48
SDS-V3-plasma-45_Cluster_1451_sequences=48	282	48
SDS-V3-plasma-46_Cluster_433_sequences=48	286	48
SDS-V3-plasma-46_Cluster_2593_sequences=48	286	48
SDS-V3-plasma-46_Cluster_4048_sequences=48	286	48
SDS-V3-plasma-46_Cluster_1469_sequences=48	286	48
SDS-V3-plasma-46_Cluster_629_sequences=48	286	48
SDS-V3-plasma-46_Cluster_514_sequences=48	286	48
SDS-V3-plasma-46_Cluster_2073_sequences=48	286	48
SDS-V3-plasma-46_Cluster_734_sequences=48	286	48
SDS-V3-plasma-67_Cluster_12576_sequences=48	504	48
SDS-V3-plasma-67_Cluster_2266_sequences=48	504	48
SDS-V3-plasma-67_Cluster_4737_sequences=48	504	48

SDS-V3-plasma-0_Cluster_1493_sequences=47	0	47
SDS-V3-plasma-0_Cluster_4482_sequences=47	0	47
SDS-V3-plasma-0_Cluster_753_sequences=47	0	47
SDS-V3-plasma-0_Cluster_2174_sequences=47	0	47
SDS-V3-plasma-0_Cluster_32_sequences=47	0	47
SDS-V3-plasma-0_Cluster_29_sequences=47	0	47
SDS-V3-plasma-0_Cluster_2852_sequences=47	0	47
SDS-V3-plasma-5_Cluster_392_sequences=47	9	47
SDS-V3-plasma-8_Cluster_2882_sequences=47	16	47
SDS-V3-plasma-8_Cluster_2795_sequences=47	16	47
SDS-V3-plasma-24_Cluster_717_sequences=47	124	47
SDS-V3-plasma-24_Cluster_842_sequences=47	124	47
SDS-V3-plasma-24_Cluster_1196_sequences=47	124	47
SDS-V3-plasma-24_Cluster_144_sequences=47	124	47
SDS-V3-plasma-27_Cluster_11_sequences=47	131	47
SDS-V3-plasma-27_Cluster_1568_sequences=47	131	47
SDS-V3-plasma-27_Cluster_156_sequences=47	131	47
SDS-V3-plasma-27_Cluster_1584_sequences=47	131	47
SDS-V3-plasma-27_Cluster_558_sequences=47	131	47
SDS-V3-plasma-27_Cluster_592_sequences=47	131	47
SDS-V3-plasma-27_Cluster_652_sequences=47	131	47
SDS-V3-plasma-27_Cluster_703_sequences=47	131	47
SDS-V3-plasma-27_Cluster_71_sequences=47	131	47
SDS-V3-plasma-27_Cluster_233_sequences=47	131	47
SDS-V3-plasma-27_Cluster_530_sequences=47	131	47
SDS-V3-plasma-27_Cluster_366_sequences=47	131	47
SDS-V3-plasma-27_Cluster_651_sequences=47	131	47
SDS-V3-plasma-45_Cluster_8954_sequences=47	282	47
SDS-V3-plasma-45_Cluster_12451_sequences=47	282	47
SDS-V3-plasma-45_Cluster_331_sequences=47	282	47
SDS-V3-plasma-45_Cluster_13698_sequences=47	282	47
SDS-V3-plasma-45_Cluster_3392_sequences=47	282	47
SDS-V3-plasma-45_Cluster_6123_sequences=47	282	47
SDS-V3-plasma-45_Cluster_6440_sequences=47	282	47
SDS-V3-plasma-45_Cluster_347_sequences=47	282	47
SDS-V3-plasma-45_Cluster_5180_sequences=47	282	47
SDS-V3-plasma-45_Cluster_764_sequences=47	282	47
SDS-V3-plasma-45_Cluster_11217_sequences=47	282	47
SDS-V3-plasma-45_Cluster_2469_sequences=47	282	47
SDS-V3-plasma-45_Cluster_4508_sequences=47	282	47
SDS-V3-plasma-45_Cluster_640_sequences=47	282	47
SDS-V3-plasma-45_Cluster_8846_sequences=47	282	47
SDS-V3-plasma-45_Cluster_787_sequences=47	282	47
SDS-V3-plasma-46_Cluster_798_sequences=47	286	47
SDS-V3-plasma-46_Cluster_2377_sequences=47	286	47

SDS-V3-plasma-46_Cluster_2766_sequences=47	286	47
SDS-V3-plasma-46_Cluster_1974_sequences=47	286	47
SDS-V3-plasma-46_Cluster_819_sequences=47	286	47
SDS-V3-plasma-46_Cluster_1172_sequences=47	286	47
SDS-V3-plasma-67_Cluster_2647_sequences=47	504	47
SDS-V3-plasma-67_Cluster_6094_sequences=47	504	47
SDS-V3-plasma-67_Cluster_6360_sequences=47	504	47
SDS-V3-plasma-67_Cluster_4445_sequences=47	504	47
SDS-V3-plasma-67_Cluster_340_sequences=47	504	47
SDS-V3-plasma-67_Cluster_3695_sequences=47	504	47
SDS-V3-plasma-67_Cluster_4758_sequences=47	504	47
SDS-V3-plasma-67_Cluster_1344_sequences=47	504	47
SDS-V3-plasma-67_Cluster_14044_sequences=47	504	47
SDS-V3-plasma-67_Cluster_2817_sequences=47	504	47
SDS-V3-plasma-0_Cluster_1169_sequences=46	0	46
SDS-V3-plasma-0_Cluster_581_sequences=46	0	46
SDS-V3-plasma-0_Cluster_4728_sequences=46	0	46
SDS-V3-plasma-0_Cluster_982_sequences=46	0	46
SDS-V3-plasma-5_Cluster_1808_sequences=46	9	46
SDS-V3-plasma-8_Cluster_2347_sequences=46	16	46
SDS-V3-plasma-24_Cluster_153_sequences=46	124	46
SDS-V3-plasma-24_Cluster_1071_sequences=46	124	46
SDS-V3-plasma-24_Cluster_1682_sequences=46	124	46
SDS-V3-plasma-24_Cluster_633_sequences=46	124	46
SDS-V3-plasma-24_Cluster_827_sequences=46	124	46
SDS-V3-plasma-24_Cluster_615_sequences=46	124	46
SDS-V3-plasma-24_Cluster_614_sequences=46	124	46
SDS-V3-plasma-24_Cluster_601_sequences=46	124	46
SDS-V3-plasma-27_Cluster_1325_sequences=46	131	46
SDS-V3-plasma-27_Cluster_515_sequences=46	131	46
SDS-V3-plasma-27_Cluster_216_sequences=46	131	46
SDS-V3-plasma-27_Cluster_1346_sequences=46	131	46
SDS-V3-plasma-45_Cluster_10795_sequences=46	282	46
SDS-V3-plasma-45_Cluster_176_sequences=46	282	46
SDS-V3-plasma-45_Cluster_13096_sequences=46	282	46
SDS-V3-plasma-45_Cluster_1030_sequences=46	282	46
SDS-V3-plasma-45_Cluster_5426_sequences=46	282	46
SDS-V3-plasma-45_Cluster_10517_sequences=46	282	46
SDS-V3-plasma-45_Cluster_4237_sequences=46	282	46
SDS-V3-plasma-45_Cluster_871_sequences=46	282	46
SDS-V3-plasma-45_Cluster_3171_sequences=46	282	46
SDS-V3-plasma-45_Cluster_785_sequences=46	282	46
SDS-V3-plasma-45_Cluster_5318_sequences=46	282	46
SDS-V3-plasma-45_Cluster_1547_sequences=46	282	46
SDS-V3-plasma-45_Cluster_1891_sequences=46	282	46

SDS-V3-plasma-45_Cluster_4088_sequences=46	282	46
SDS-V3-plasma-45_Cluster_16064_sequences=46	282	46
SDS-V3-plasma-46_Cluster_201_sequences=46	286	46
SDS-V3-plasma-46_Cluster_2284_sequences=46	286	46
SDS-V3-plasma-46_Cluster_1834_sequences=46	286	46
SDS-V3-plasma-46_Cluster_4394_sequences=46	286	46
SDS-V3-plasma-46_Cluster_774_sequences=46	286	46
SDS-V3-plasma-67_Cluster_5291_sequences=46	504	46
SDS-V3-plasma-67_Cluster_473_sequences=46	504	46
SDS-V3-plasma-67_Cluster_2520_sequences=46	504	46
SDS-V3-plasma-67_Cluster_4518_sequences=46	504	46
SDS-V3-plasma-67_Cluster_6557_sequences=46	504	46
SDS-V3-plasma-67_Cluster_2728_sequences=46	504	46
SDS-V3-plasma-67_Cluster_4169_sequences=46	504	46
SDS-V3-plasma-67_Cluster_8826_sequences=46	504	46
SDS-V3-plasma-67_Cluster_248_sequences=46	504	46
SDS-V3-plasma-67_Cluster_17_sequences=46	504	46
SDS-V3-plasma-0_Cluster_2802_sequences=45	0	45
SDS-V3-plasma-0_Cluster_2166_sequences=45	0	45
SDS-V3-plasma-0_Cluster_1218_sequences=45	0	45
SDS-V3-plasma-0_Cluster_615_sequences=45	0	45
SDS-V3-plasma-7_Cluster_435_sequences=45	14	45
SDS-V3-plasma-8_Cluster_4148_sequences=45	16	45
SDS-V3-plasma-8_Cluster_2836_sequences=45	16	45
SDS-V3-plasma-8_Cluster_3604_sequences=45	16	45
SDS-V3-plasma-8_Cluster_3504_sequences=45	16	45
SDS-V3-plasma-24_Cluster_1437_sequences=45	124	45
SDS-V3-plasma-24_Cluster_391_sequences=45	124	45
SDS-V3-plasma-24_Cluster_822_sequences=45	124	45
SDS-V3-plasma-24_Cluster_170_sequences=45	124	45
SDS-V3-plasma-24_Cluster_68_sequences=45	124	45
SDS-V3-plasma-24_Cluster_816_sequences=45	124	45
SDS-V3-plasma-27_Cluster_1222_sequences=45	131	45
SDS-V3-plasma-27_Cluster_134_sequences=45	131	45
SDS-V3-plasma-27_Cluster_695_sequences=45	131	45
SDS-V3-plasma-27_Cluster_819_sequences=45	131	45
SDS-V3-plasma-27_Cluster_895_sequences=45	131	45
SDS-V3-plasma-27_Cluster_622_sequences=45	131	45
SDS-V3-plasma-27_Cluster_91_sequences=45	131	45
SDS-V3-plasma-27_Cluster_275_sequences=45	131	45
SDS-V3-plasma-27_Cluster_2730_sequences=45	131	45
SDS-V3-plasma-45_Cluster_7097_sequences=45	282	45
SDS-V3-plasma-45_Cluster_4469_sequences=45	282	45
SDS-V3-plasma-45_Cluster_4974_sequences=45	282	45
SDS-V3-plasma-45_Cluster_5183_sequences=45	282	45

SDS-V3-plasma-45_Cluster_14591_sequences=45	282	45
SDS-V3-plasma-45_Cluster_2243_sequences=45	282	45
SDS-V3-plasma-45_Cluster_958_sequences=45	282	45
SDS-V3-plasma-45_Cluster_7523_sequences=45	282	45
SDS-V3-plasma-45_Cluster_2537_sequences=45	282	45
SDS-V3-plasma-45_Cluster_5686_sequences=45	282	45
SDS-V3-plasma-45_Cluster_2109_sequences=45	282	45
SDS-V3-plasma-45_Cluster_5269_sequences=45	282	45
SDS-V3-plasma-45_Cluster_667_sequences=45	282	45
SDS-V3-plasma-45_Cluster_8361_sequences=45	282	45
SDS-V3-plasma-45_Cluster_70118_sequences=45	282	45
SDS-V3-plasma-45_Cluster_1788_sequences=45	282	45
SDS-V3-plasma-45_Cluster_7619_sequences=45	282	45
SDS-V3-plasma-45_Cluster_8283_sequences=45	282	45
SDS-V3-plasma-45_Cluster_1965_sequences=45	282	45
SDS-V3-plasma-46_Cluster_244_sequences=45	286	45
SDS-V3-plasma-46_Cluster_1223_sequences=45	286	45
SDS-V3-plasma-46_Cluster_673_sequences=45	286	45
SDS-V3-plasma-46_Cluster_1442_sequences=45	286	45
SDS-V3-plasma-46_Cluster_3693_sequences=45	286	45
SDS-V3-plasma-46_Cluster_2110_sequences=45	286	45
SDS-V3-plasma-46_Cluster_353_sequences=45	286	45
SDS-V3-plasma-67_Cluster_5202_sequences=45	504	45
SDS-V3-plasma-67_Cluster_13918_sequences=45	504	45
SDS-V3-plasma-67_Cluster_4773_sequences=45	504	45
SDS-V3-plasma-67_Cluster_2929_sequences=45	504	45
SDS-V3-plasma-67_Cluster_3567_sequences=45	504	45
SDS-V3-plasma-67_Cluster_4661_sequences=45	504	45
SDS-V3-plasma-67_Cluster_9184_sequences=45	504	45
SDS-V3-plasma-67_Cluster_2132_sequences=45	504	45
SDS-V3-plasma-67_Cluster_15854_sequences=45	504	45
SDS-V3-plasma-67_Cluster_1675_sequences=45	504	45
SDS-V3-plasma-67_Cluster_1465_sequences=45	504	45
SDS-V3-plasma-67_Cluster_987_sequences=45	504	45
SDS-V3-plasma-0_Cluster_1265_sequences=44	0	44
SDS-V3-plasma-0_Cluster_1315_sequences=44	0	44
SDS-V3-plasma-0_Cluster_1644_sequences=44	0	44
SDS-V3-plasma-0_Cluster_3061_sequences=44	0	44
SDS-V3-plasma-0_Cluster_2379_sequences=44	0	44
SDS-V3-plasma-0_Cluster_816_sequences=44	0	44
SDS-V3-plasma-0_Cluster_4434_sequences=44	0	44
SDS-V3-plasma-0_Cluster_690_sequences=44	0	44
SDS-V3-plasma-0_Cluster_2014_sequences=44	0	44
SDS-V3-plasma-0_Cluster_2753_sequences=44	0	44
SDS-V3-plasma-0_Cluster_405_sequences=44	0	44

SDS-V3-plasma-0_Cluster_520_sequences=44	0	44
SDS-V3-plasma-0_Cluster_5268_sequences=44	0	44
SDS-V3-plasma-0_Cluster_604_sequences=44	0	44
SDS-V3-plasma-0_Cluster_1703_sequences=44	0	44
SDS-V3-plasma-0_Cluster_1266_sequences=44	0	44
SDS-V3-plasma-0_Cluster_1648_sequences=44	0	44
SDS-V3-plasma-5_Cluster_23_sequences=44	9	44
SDS-V3-plasma-7_Cluster_226_sequences=44	14	44
SDS-V3-plasma-7_Cluster_433_sequences=44	14	44
SDS-V3-plasma-7_Cluster_732_sequences=44	14	44
SDS-V3-plasma-8_Cluster_3271_sequences=44	16	44
SDS-V3-plasma-8_Cluster_2621_sequences=44	16	44
SDS-V3-plasma-24_Cluster_1237_sequences=44	124	44
SDS-V3-plasma-24_Cluster_1633_sequences=44	124	44
SDS-V3-plasma-24_Cluster_321_sequences=44	124	44
SDS-V3-plasma-24_Cluster_351_sequences=44	124	44
SDS-V3-plasma-24_Cluster_193_sequences=44	124	44
SDS-V3-plasma-24_Cluster_2552_sequences=44	124	44
SDS-V3-plasma-24_Cluster_378_sequences=44	124	44
SDS-V3-plasma-24_Cluster_419_sequences=44	124	44
SDS-V3-plasma-24_Cluster_608_sequences=44	124	44
SDS-V3-plasma-27_Cluster_1298_sequences=44	131	44
SDS-V3-plasma-27_Cluster_1526_sequences=44	131	44
SDS-V3-plasma-27_Cluster_1898_sequences=44	131	44
SDS-V3-plasma-45_Cluster_11027_sequences=44	282	44
SDS-V3-plasma-45_Cluster_7768_sequences=44	282	44
SDS-V3-plasma-45_Cluster_148_sequences=44	282	44
SDS-V3-plasma-45_Cluster_11971_sequences=44	282	44
SDS-V3-plasma-45_Cluster_1609_sequences=44	282	44
SDS-V3-plasma-45_Cluster_672_sequences=44	282	44
SDS-V3-plasma-45_Cluster_759_sequences=44	282	44
SDS-V3-plasma-45_Cluster_2003_sequences=44	282	44
SDS-V3-plasma-45_Cluster_2353_sequences=44	282	44
SDS-V3-plasma-45_Cluster_10853_sequences=44	282	44
SDS-V3-plasma-45_Cluster_11456_sequences=44	282	44
SDS-V3-plasma-45_Cluster_11760_sequences=44	282	44
SDS-V3-plasma-45_Cluster_12491_sequences=44	282	44
SDS-V3-plasma-45_Cluster_129_sequences=44	282	44
SDS-V3-plasma-45_Cluster_3595_sequences=44	282	44
SDS-V3-plasma-45_Cluster_3874_sequences=44	282	44
SDS-V3-plasma-45_Cluster_9354_sequences=44	282	44
SDS-V3-plasma-45_Cluster_8280_sequences=44	282	44
SDS-V3-plasma-45_Cluster_1978_sequences=44	282	44
SDS-V3-plasma-45_Cluster_2925_sequences=44	282	44
SDS-V3-plasma-45_Cluster_262_sequences=44	282	44

SDS-V3-plasma-45_Cluster_1903_sequences=44	282	44
SDS-V3-plasma-45_Cluster_4669_sequences=44	282	44
SDS-V3-plasma-45_Cluster_8390_sequences=44	282	44
SDS-V3-plasma-45_Cluster_2409_sequences=44	282	44
SDS-V3-plasma-45_Cluster_1570_sequences=44	282	44
SDS-V3-plasma-45_Cluster_323_sequences=44	282	44
SDS-V3-plasma-45_Cluster_8768_sequences=44	282	44
SDS-V3-plasma-45_Cluster_4694_sequences=44	282	44
SDS-V3-plasma-46_Cluster_2838_sequences=44	286	44
SDS-V3-plasma-46_Cluster_2017_sequences=44	286	44
SDS-V3-plasma-46_Cluster_8_sequences=44	286	44
SDS-V3-plasma-67_Cluster_935_sequences=44	504	44
SDS-V3-plasma-67_Cluster_9884_sequences=44	504	44
SDS-V3-plasma-67_Cluster_11259_sequences=44	504	44
SDS-V3-plasma-67_Cluster_6091_sequences=44	504	44
SDS-V3-plasma-67_Cluster_2305_sequences=44	504	44
SDS-V3-plasma-67_Cluster_8745_sequences=44	504	44
SDS-V3-plasma-67_Cluster_9273_sequences=44	504	44
SDS-V3-plasma-67_Cluster_7490_sequences=44	504	44
SDS-V3-plasma-0_Cluster_887_sequences=43	0	43
SDS-V3-plasma-0_Cluster_2224_sequences=43	0	43
SDS-V3-plasma-0_Cluster_2260_sequences=43	0	43
SDS-V3-plasma-0_Cluster_1771_sequences=43	0	43
SDS-V3-plasma-0_Cluster_2346_sequences=43	0	43
SDS-V3-plasma-0_Cluster_542_sequences=43	0	43
SDS-V3-plasma-0_Cluster_1110_sequences=43	0	43
SDS-V3-plasma-0_Cluster_1351_sequences=43	0	43
SDS-V3-plasma-5_Cluster_565_sequences=43	9	43
SDS-V3-plasma-8_Cluster_2290_sequences=43	16	43
SDS-V3-plasma-8_Cluster_3474_sequences=43	16	43
SDS-V3-plasma-8_Cluster_2681_sequences=43	16	43
SDS-V3-plasma-24_Cluster_94_sequences=43	124	43
SDS-V3-plasma-24_Cluster_935_sequences=43	124	43
SDS-V3-plasma-27_Cluster_1312_sequences=43	131	43
SDS-V3-plasma-27_Cluster_296_sequences=43	131	43
SDS-V3-plasma-27_Cluster_44_sequences=43	131	43
SDS-V3-plasma-27_Cluster_573_sequences=43	131	43
SDS-V3-plasma-27_Cluster_88_sequences=43	131	43
SDS-V3-plasma-27_Cluster_246_sequences=43	131	43
SDS-V3-plasma-27_Cluster_151_sequences=43	131	43
SDS-V3-plasma-45_Cluster_7445_sequences=43	282	43
SDS-V3-plasma-45_Cluster_2589_sequences=43	282	43
SDS-V3-plasma-45_Cluster_3058_sequences=43	282	43
SDS-V3-plasma-45_Cluster_1610_sequences=43	282	43
SDS-V3-plasma-45_Cluster_3310_sequences=43	282	43

SDS-V3-plasma-45_Cluster_7171_sequences=43	282	43
SDS-V3-plasma-45_Cluster_543_sequences=43	282	43
SDS-V3-plasma-45_Cluster_7297_sequences=43	282	43
SDS-V3-plasma-45_Cluster_7441_sequences=43	282	43
SDS-V3-plasma-45_Cluster_348_sequences=43	282	43
SDS-V3-plasma-45_Cluster_13469_sequences=43	282	43
SDS-V3-plasma-45_Cluster_8326_sequences=43	282	43
SDS-V3-plasma-45_Cluster_2401_sequences=43	282	43
SDS-V3-plasma-45_Cluster_1893_sequences=43	282	43
SDS-V3-plasma-45_Cluster_4667_sequences=43	282	43
SDS-V3-plasma-45_Cluster_10050_sequences=43	282	43
SDS-V3-plasma-46_Cluster_2228_sequences=43	286	43
SDS-V3-plasma-46_Cluster_1039_sequences=43	286	43
SDS-V3-plasma-46_Cluster_1489_sequences=43	286	43
SDS-V3-plasma-46_Cluster_58_sequences=43	286	43
SDS-V3-plasma-46_Cluster_843_sequences=43	286	43
SDS-V3-plasma-46_Cluster_1912_sequences=43	286	43
SDS-V3-plasma-67_Cluster_5626_sequences=43	504	43
SDS-V3-plasma-67_Cluster_161_sequences=43	504	43
SDS-V3-plasma-67_Cluster_6113_sequences=43	504	43
SDS-V3-plasma-67_Cluster_11125_sequences=43	504	43
SDS-V3-plasma-67_Cluster_530_sequences=43	504	43
SDS-V3-plasma-67_Cluster_5318_sequences=43	504	43
SDS-V3-plasma-67_Cluster_3694_sequences=43	504	43
SDS-V3-plasma-0_Cluster_1274_sequences=42	0	42
SDS-V3-plasma-0_Cluster_2119_sequences=42	0	42
SDS-V3-plasma-0_Cluster_2214_sequences=42	0	42
SDS-V3-plasma-0_Cluster_1407_sequences=42	0	42
SDS-V3-plasma-0_Cluster_1887_sequences=42	0	42
SDS-V3-plasma-0_Cluster_1890_sequences=42	0	42
SDS-V3-plasma-0_Cluster_40_sequences=42	0	42
SDS-V3-plasma-0_Cluster_2779_sequences=42	0	42
SDS-V3-plasma-0_Cluster_1865_sequences=42	0	42
SDS-V3-plasma-7_Cluster_345_sequences=42	14	42
SDS-V3-plasma-7_Cluster_421_sequences=42	14	42
SDS-V3-plasma-8_Cluster_2308_sequences=42	16	42
SDS-V3-plasma-8_Cluster_3680_sequences=42	16	42
SDS-V3-plasma-24_Cluster_1298_sequences=42	124	42
SDS-V3-plasma-24_Cluster_1600_sequences=42	124	42
SDS-V3-plasma-24_Cluster_26_sequences=42	124	42
SDS-V3-plasma-24_Cluster_371_sequences=42	124	42
SDS-V3-plasma-24_Cluster_877_sequences=42	124	42
SDS-V3-plasma-24_Cluster_409_sequences=42	124	42
SDS-V3-plasma-24_Cluster_377_sequences=42	124	42
SDS-V3-plasma-24_Cluster_428_sequences=42	124	42

SDS-V3-plasma-24_Cluster_415_sequences=42	124	42
SDS-V3-plasma-24_Cluster_212_sequences=42	124	42
SDS-V3-plasma-27_Cluster_1439_sequences=42	131	42
SDS-V3-plasma-27_Cluster_1190_sequences=42	131	42
SDS-V3-plasma-27_Cluster_2226_sequences=42	131	42
SDS-V3-plasma-27_Cluster_104_sequences=42	131	42
SDS-V3-plasma-27_Cluster_390_sequences=42	131	42
SDS-V3-plasma-27_Cluster_735_sequences=42	131	42
SDS-V3-plasma-45_Cluster_1409_sequences=42	282	42
SDS-V3-plasma-45_Cluster_1857_sequences=42	282	42
SDS-V3-plasma-45_Cluster_4965_sequences=42	282	42
SDS-V3-plasma-45_Cluster_6047_sequences=42	282	42
SDS-V3-plasma-45_Cluster_12103_sequences=42	282	42
SDS-V3-plasma-45_Cluster_2553_sequences=42	282	42
SDS-V3-plasma-45_Cluster_11878_sequences=42	282	42
SDS-V3-plasma-45_Cluster_8682_sequences=42	282	42
SDS-V3-plasma-45_Cluster_1139_sequences=42	282	42
SDS-V3-plasma-45_Cluster_1653_sequences=42	282	42
SDS-V3-plasma-45_Cluster_13408_sequences=42	282	42
SDS-V3-plasma-45_Cluster_6712_sequences=42	282	42
SDS-V3-plasma-45_Cluster_7661_sequences=42	282	42
SDS-V3-plasma-45_Cluster_3351_sequences=42	282	42
SDS-V3-plasma-45_Cluster_4501_sequences=42	282	42
SDS-V3-plasma-45_Cluster_3554_sequences=42	282	42
SDS-V3-plasma-45_Cluster_24118_sequences=42	282	42
SDS-V3-plasma-45_Cluster_1925_sequences=42	282	42
SDS-V3-plasma-45_Cluster_993_sequences=42	282	42
SDS-V3-plasma-45_Cluster_8210_sequences=42	282	42
SDS-V3-plasma-45_Cluster_12390_sequences=42	282	42
SDS-V3-plasma-45_Cluster_608_sequences=42	282	42
SDS-V3-plasma-45_Cluster_16979_sequences=42	282	42
SDS-V3-plasma-45_Cluster_5703_sequences=42	282	42
SDS-V3-plasma-45_Cluster_4304_sequences=42	282	42
SDS-V3-plasma-45_Cluster_8471_sequences=42	282	42
SDS-V3-plasma-46_Cluster_807_sequences=42	286	42
SDS-V3-plasma-46_Cluster_1264_sequences=42	286	42
SDS-V3-plasma-46_Cluster_408_sequences=42	286	42
SDS-V3-plasma-46_Cluster_950_sequences=42	286	42
SDS-V3-plasma-46_Cluster_131_sequences=42	286	42
SDS-V3-plasma-46_Cluster_964_sequences=42	286	42
SDS-V3-plasma-46_Cluster_1395_sequences=42	286	42
SDS-V3-plasma-46_Cluster_1733_sequences=42	286	42
SDS-V3-plasma-67_Cluster_5360_sequences=42	504	42
SDS-V3-plasma-67_Cluster_3803_sequences=42	504	42
SDS-V3-plasma-67_Cluster_13586_sequences=42	504	42

SDS-V3-plasma-67_Cluster_511_sequences=42	504	42
SDS-V3-plasma-67_Cluster_10241_sequences=42	504	42
SDS-V3-plasma-67_Cluster_16919_sequences=42	504	42
SDS-V3-plasma-67_Cluster_1713_sequences=42	504	42
SDS-V3-plasma-67_Cluster_9091_sequences=42	504	42
SDS-V3-plasma-67_Cluster_1346_sequences=42	504	42
SDS-V3-plasma-67_Cluster_4180_sequences=42	504	42
SDS-V3-plasma-67_Cluster_132_sequences=42	504	42
SDS-V3-plasma-67_Cluster_2934_sequences=42	504	42
SDS-V3-plasma-67_Cluster_7367_sequences=42	504	42
SDS-V3-plasma-67_Cluster_8472_sequences=42	504	42
SDS-V3-plasma-67_Cluster_150_sequences=42	504	42
SDS-V3-plasma-67_Cluster_6863_sequences=42	504	42
SDS-V3-plasma-67_Cluster_4259_sequences=42	504	42
SDS-V3-plasma-67_Cluster_6260_sequences=42	504	42
SDS-V3-plasma-67_Cluster_5203_sequences=42	504	42
SDS-V3-plasma-67_Cluster_1335_sequences=42	504	42
SDS-V3-plasma-67_Cluster_1380_sequences=42	504	42
SDS-V3-plasma-0_Cluster_1162_sequences=41	0	41
SDS-V3-plasma-0_Cluster_165_sequences=41	0	41
SDS-V3-plasma-0_Cluster_1805_sequences=41	0	41
SDS-V3-plasma-0_Cluster_182_sequences=41	0	41
SDS-V3-plasma-0_Cluster_2349_sequences=41	0	41
SDS-V3-plasma-0_Cluster_2947_sequences=41	0	41
SDS-V3-plasma-0_Cluster_877_sequences=41	0	41
SDS-V3-plasma-0_Cluster_926_sequences=41	0	41
SDS-V3-plasma-0_Cluster_2169_sequences=41	0	41
SDS-V3-plasma-0_Cluster_2048_sequences=41	0	41
SDS-V3-plasma-5_Cluster_15_sequences=41	9	41
SDS-V3-plasma-5_Cluster_28_sequences=41	9	41
SDS-V3-plasma-7_Cluster_427_sequences=41	14	41
SDS-V3-plasma-7_Cluster_123_sequences=41	14	41
SDS-V3-plasma-7_Cluster_705_sequences=41	14	41
SDS-V3-plasma-8_Cluster_2324_sequences=41	16	41
SDS-V3-plasma-8_Cluster_2906_sequences=41	16	41
SDS-V3-plasma-8_Cluster_2440_sequences=41	16	41
SDS-V3-plasma-8_Cluster_2457_sequences=41	16	41
SDS-V3-plasma-24_Cluster_1086_sequences=41	124	41
SDS-V3-plasma-24_Cluster_1317_sequences=41	124	41
SDS-V3-plasma-24_Cluster_1508_sequences=41	124	41
SDS-V3-plasma-24_Cluster_206_sequences=41	124	41
SDS-V3-plasma-24_Cluster_230_sequences=41	124	41
SDS-V3-plasma-24_Cluster_232_sequences=41	124	41
SDS-V3-plasma-24_Cluster_962_sequences=41	124	41
SDS-V3-plasma-24_Cluster_787_sequences=41	124	41

SDS-V3-plasma-24_Cluster_525_sequences=41	124	41
SDS-V3-plasma-24_Cluster_889_sequences=41	124	41
SDS-V3-plasma-27_Cluster_535_sequences=41	131	41
SDS-V3-plasma-27_Cluster_1020_sequences=41	131	41
SDS-V3-plasma-27_Cluster_1791_sequences=41	131	41
SDS-V3-plasma-27_Cluster_187_sequences=41	131	41
SDS-V3-plasma-27_Cluster_1341_sequences=41	131	41
SDS-V3-plasma-27_Cluster_1025_sequences=41	131	41
SDS-V3-plasma-27_Cluster_295_sequences=41	131	41
SDS-V3-plasma-45_Cluster_15000_sequences=41	282	41
SDS-V3-plasma-45_Cluster_7457_sequences=41	282	41
SDS-V3-plasma-45_Cluster_10412_sequences=41	282	41
SDS-V3-plasma-45_Cluster_1400_sequences=41	282	41
SDS-V3-plasma-45_Cluster_9409_sequences=41	282	41
SDS-V3-plasma-45_Cluster_5007_sequences=41	282	41
SDS-V3-plasma-45_Cluster_4949_sequences=41	282	41
SDS-V3-plasma-45_Cluster_710_sequences=41	282	41
SDS-V3-plasma-45_Cluster_1402_sequences=41	282	41
SDS-V3-plasma-45_Cluster_5182_sequences=41	282	41
SDS-V3-plasma-45_Cluster_2078_sequences=41	282	41
SDS-V3-plasma-45_Cluster_9774_sequences=41	282	41
SDS-V3-plasma-45_Cluster_4137_sequences=41	282	41
SDS-V3-plasma-45_Cluster_5449_sequences=41	282	41
SDS-V3-plasma-45_Cluster_2829_sequences=41	282	41
SDS-V3-plasma-45_Cluster_15803_sequences=41	282	41
SDS-V3-plasma-45_Cluster_7477_sequences=41	282	41
SDS-V3-plasma-45_Cluster_6970_sequences=41	282	41
SDS-V3-plasma-45_Cluster_15918_sequences=41	282	41
SDS-V3-plasma-45_Cluster_2389_sequences=41	282	41
SDS-V3-plasma-45_Cluster_2376_sequences=41	282	41
SDS-V3-plasma-45_Cluster_3934_sequences=41	282	41
SDS-V3-plasma-45_Cluster_10059_sequences=41	282	41
SDS-V3-plasma-45_Cluster_8875_sequences=41	282	41
SDS-V3-plasma-46_Cluster_1031_sequences=41	286	41
SDS-V3-plasma-46_Cluster_102_sequences=41	286	41
SDS-V3-plasma-46_Cluster_740_sequences=41	286	41
SDS-V3-plasma-46_Cluster_192_sequences=41	286	41
SDS-V3-plasma-46_Cluster_401_sequences=41	286	41
SDS-V3-plasma-46_Cluster_643_sequences=41	286	41
SDS-V3-plasma-67_Cluster_3364_sequences=41	504	41
SDS-V3-plasma-67_Cluster_12801_sequences=41	504	41
SDS-V3-plasma-67_Cluster_16007_sequences=41	504	41
SDS-V3-plasma-67_Cluster_2709_sequences=41	504	41
SDS-V3-plasma-67_Cluster_3696_sequences=41	504	41
SDS-V3-plasma-67_Cluster_8874_sequences=41	504	41

SDS-V3-plasma-67_Cluster_7842_sequences=41	504	41
SDS-V3-plasma-67_Cluster_2395_sequences=41	504	41
SDS-V3-plasma-67_Cluster_5142_sequences=41	504	41
SDS-V3-plasma-67_Cluster_4614_sequences=41	504	41
SDS-V3-plasma-67_Cluster_12344_sequences=41	504	41
SDS-V3-plasma-0_Cluster_1099_sequences=40	0	40
SDS-V3-plasma-0_Cluster_1350_sequences=40	0	40
SDS-V3-plasma-0_Cluster_490_sequences=40	0	40
SDS-V3-plasma-0_Cluster_6275_sequences=40	0	40
SDS-V3-plasma-0_Cluster_4106_sequences=40	0	40
SDS-V3-plasma-0_Cluster_52_sequences=40	0	40
SDS-V3-plasma-0_Cluster_1985_sequences=40	0	40
SDS-V3-plasma-0_Cluster_2619_sequences=40	0	40
SDS-V3-plasma-0_Cluster_4100_sequences=40	0	40
SDS-V3-plasma-0_Cluster_2258_sequences=40	0	40
SDS-V3-plasma-7_Cluster_130_sequences=40	14	40
SDS-V3-plasma-7_Cluster_355_sequences=40	14	40
SDS-V3-plasma-7_Cluster_1050_sequences=40	14	40
SDS-V3-plasma-7_Cluster_270_sequences=40	14	40
SDS-V3-plasma-7_Cluster_137_sequences=40	14	40
SDS-V3-plasma-24_Cluster_1158_sequences=40	124	40
SDS-V3-plasma-24_Cluster_1313_sequences=40	124	40
SDS-V3-plasma-24_Cluster_1489_sequences=40	124	40
SDS-V3-plasma-24_Cluster_247_sequences=40	124	40
SDS-V3-plasma-24_Cluster_635_sequences=40	124	40
SDS-V3-plasma-24_Cluster_708_sequences=40	124	40
SDS-V3-plasma-24_Cluster_40_sequences=40	124	40
SDS-V3-plasma-24_Cluster_520_sequences=40	124	40
SDS-V3-plasma-24_Cluster_60_sequences=40	124	40
SDS-V3-plasma-24_Cluster_470_sequences=40	124	40
SDS-V3-plasma-24_Cluster_613_sequences=40	124	40
SDS-V3-plasma-24_Cluster_859_sequences=40	124	40
SDS-V3-plasma-27_Cluster_1037_sequences=40	131	40
SDS-V3-plasma-27_Cluster_148_sequences=40	131	40
SDS-V3-plasma-27_Cluster_235_sequences=40	131	40
SDS-V3-plasma-27_Cluster_398_sequences=40	131	40
SDS-V3-plasma-45_Cluster_3373_sequences=40	282	40
SDS-V3-plasma-45_Cluster_3774_sequences=40	282	40
SDS-V3-plasma-45_Cluster_822_sequences=40	282	40
SDS-V3-plasma-45_Cluster_1684_sequences=40	282	40
SDS-V3-plasma-45_Cluster_284_sequences=40	282	40
SDS-V3-plasma-45_Cluster_7558_sequences=40	282	40
SDS-V3-plasma-45_Cluster_289_sequences=40	282	40
SDS-V3-plasma-45_Cluster_8661_sequences=40	282	40
SDS-V3-plasma-45_Cluster_3570_sequences=40	282	40

SDS-V3-plasma-45_Cluster_7039_sequences=40	282	40
SDS-V3-plasma-45_Cluster_3625_sequences=40	282	40
SDS-V3-plasma-45_Cluster_17964_sequences=40	282	40
SDS-V3-plasma-45_Cluster_5202_sequences=40	282	40
SDS-V3-plasma-45_Cluster_16575_sequences=40	282	40
SDS-V3-plasma-45_Cluster_2937_sequences=40	282	40
SDS-V3-plasma-45_Cluster_6687_sequences=40	282	40
SDS-V3-plasma-45_Cluster_11356_sequences=40	282	40
SDS-V3-plasma-45_Cluster_17370_sequences=40	282	40
SDS-V3-plasma-45_Cluster_5223_sequences=40	282	40
SDS-V3-plasma-45_Cluster_9624_sequences=40	282	40
SDS-V3-plasma-45_Cluster_304_sequences=40	282	40
SDS-V3-plasma-46_Cluster_3043_sequences=40	286	40
SDS-V3-plasma-46_Cluster_1015_sequences=40	286	40
SDS-V3-plasma-46_Cluster_882_sequences=40	286	40
SDS-V3-plasma-46_Cluster_4247_sequences=40	286	40
SDS-V3-plasma-67_Cluster_1317_sequences=40	504	40
SDS-V3-plasma-67_Cluster_662_sequences=40	504	40
SDS-V3-plasma-67_Cluster_2145_sequences=40	504	40
SDS-V3-plasma-67_Cluster_11943_sequences=40	504	40
SDS-V3-plasma-67_Cluster_2659_sequences=40	504	40
SDS-V3-plasma-67_Cluster_571_sequences=40	504	40
SDS-V3-plasma-67_Cluster_5889_sequences=40	504	40
SDS-V3-plasma-67_Cluster_9105_sequences=40	504	40
SDS-V3-plasma-67_Cluster_1869_sequences=40	504	40
SDS-V3-plasma-67_Cluster_14054_sequences=40	504	40
SDS-V3-plasma-67_Cluster_9109_sequences=40	504	40
SDS-V3-plasma-67_Cluster_8163_sequences=40	504	40
SDS-V3-plasma-67_Cluster_4961_sequences=40	504	40
SDS-V3-plasma-67_Cluster_145_sequences=40	504	40
SDS-V3-plasma-67_Cluster_121_sequences=40	504	40
SDS-V3-plasma-67_Cluster_15023_sequences=40	504	40
SDS-V3-plasma-67_Cluster_2069_sequences=40	504	40
SDS-V3-plasma-0_Cluster_5005_sequences=39	0	39
SDS-V3-plasma-0_Cluster_812_sequences=39	0	39
SDS-V3-plasma-0_Cluster_85_sequences=39	0	39
SDS-V3-plasma-0_Cluster_553_sequences=39	0	39
SDS-V3-plasma-0_Cluster_1140_sequences=39	0	39
SDS-V3-plasma-0_Cluster_1882_sequences=39	0	39
SDS-V3-plasma-0_Cluster_35_sequences=39	0	39
SDS-V3-plasma-0_Cluster_441_sequences=39	0	39
SDS-V3-plasma-0_Cluster_351_sequences=39	0	39
SDS-V3-plasma-0_Cluster_2436_sequences=39	0	39
SDS-V3-plasma-0_Cluster_5223_sequences=39	0	39
SDS-V3-plasma-0_Cluster_5838_sequences=39	0	39

SDS-V3-plasma-0_Cluster_865_sequences=39	0	39
SDS-V3-plasma-0_Cluster_951_sequences=39	0	39
SDS-V3-plasma-5_Cluster_21_sequences=39	9	39
SDS-V3-plasma-5_Cluster_167_sequences=39	9	39
SDS-V3-plasma-7_Cluster_907_sequences=39	14	39
SDS-V3-plasma-7_Cluster_551_sequences=39	14	39
SDS-V3-plasma-7_Cluster_1131_sequences=39	14	39
SDS-V3-plasma-8_Cluster_39_sequences=39	16	39
SDS-V3-plasma-8_Cluster_2489_sequences=39	16	39
SDS-V3-plasma-8_Cluster_3208_sequences=39	16	39
SDS-V3-plasma-8_Cluster_3174_sequences=39	16	39
SDS-V3-plasma-24_Cluster_1524_sequences=39	124	39
SDS-V3-plasma-24_Cluster_220_sequences=39	124	39
SDS-V3-plasma-24_Cluster_618_sequences=39	124	39
SDS-V3-plasma-24_Cluster_1266_sequences=39	124	39
SDS-V3-plasma-24_Cluster_697_sequences=39	124	39
SDS-V3-plasma-27_Cluster_1060_sequences=39	131	39
SDS-V3-plasma-27_Cluster_1752_sequences=39	131	39
SDS-V3-plasma-27_Cluster_3437_sequences=39	131	39
SDS-V3-plasma-27_Cluster_620_sequences=39	131	39
SDS-V3-plasma-27_Cluster_718_sequences=39	131	39
SDS-V3-plasma-27_Cluster_2090_sequences=39	131	39
SDS-V3-plasma-27_Cluster_141_sequences=39	131	39
SDS-V3-plasma-27_Cluster_685_sequences=39	131	39
SDS-V3-plasma-27_Cluster_2086_sequences=39	131	39
SDS-V3-plasma-27_Cluster_2073_sequences=39	131	39
SDS-V3-plasma-27_Cluster_113_sequences=39	131	39
SDS-V3-plasma-45_Cluster_2936_sequences=39	282	39
SDS-V3-plasma-45_Cluster_1086_sequences=39	282	39
SDS-V3-plasma-45_Cluster_3213_sequences=39	282	39
SDS-V3-plasma-45_Cluster_351_sequences=39	282	39
SDS-V3-plasma-45_Cluster_1423_sequences=39	282	39
SDS-V3-plasma-45_Cluster_5885_sequences=39	282	39
SDS-V3-plasma-45_Cluster_4681_sequences=39	282	39
SDS-V3-plasma-45_Cluster_4606_sequences=39	282	39
SDS-V3-plasma-45_Cluster_5881_sequences=39	282	39
SDS-V3-plasma-45_Cluster_1069_sequences=39	282	39
SDS-V3-plasma-45_Cluster_1138_sequences=39	282	39
SDS-V3-plasma-45_Cluster_7063_sequences=39	282	39
SDS-V3-plasma-45_Cluster_2475_sequences=39	282	39
SDS-V3-plasma-45_Cluster_4424_sequences=39	282	39
SDS-V3-plasma-45_Cluster_5110_sequences=39	282	39
SDS-V3-plasma-45_Cluster_887_sequences=39	282	39
SDS-V3-plasma-45_Cluster_3576_sequences=39	282	39
SDS-V3-plasma-45_Cluster_5404_sequences=39	282	39

SDS-V3-plasma-45_Cluster_4559_sequences=39	282	39
SDS-V3-plasma-45_Cluster_2597_sequences=39	282	39
SDS-V3-plasma-45_Cluster_6976_sequences=39	282	39
SDS-V3-plasma-45_Cluster_6735_sequences=39	282	39
SDS-V3-plasma-45_Cluster_2470_sequences=39	282	39
SDS-V3-plasma-45_Cluster_8810_sequences=39	282	39
SDS-V3-plasma-45_Cluster_4059_sequences=39	282	39
SDS-V3-plasma-45_Cluster_5011_sequences=39	282	39
SDS-V3-plasma-45_Cluster_6649_sequences=39	282	39
SDS-V3-plasma-45_Cluster_9625_sequences=39	282	39
SDS-V3-plasma-46_Cluster_962_sequences=39	286	39
SDS-V3-plasma-46_Cluster_376_sequences=39	286	39
SDS-V3-plasma-46_Cluster_2547_sequences=39	286	39
SDS-V3-plasma-46_Cluster_1313_sequences=39	286	39
SDS-V3-plasma-46_Cluster_338_sequences=39	286	39
SDS-V3-plasma-46_Cluster_2720_sequences=39	286	39
SDS-V3-plasma-46_Cluster_1118_sequences=39	286	39
SDS-V3-plasma-46_Cluster_61_sequences=39	286	39
SDS-V3-plasma-67_Cluster_365_sequences=39	504	39
SDS-V3-plasma-67_Cluster_3876_sequences=39	504	39
SDS-V3-plasma-67_Cluster_3316_sequences=39	504	39
SDS-V3-plasma-67_Cluster_5792_sequences=39	504	39
SDS-V3-plasma-67_Cluster_7261_sequences=39	504	39
SDS-V3-plasma-67_Cluster_1256_sequences=39	504	39
SDS-V3-plasma-67_Cluster_539_sequences=39	504	39
SDS-V3-plasma-67_Cluster_937_sequences=39	504	39
SDS-V3-plasma-67_Cluster_6924_sequences=39	504	39
SDS-V3-plasma-67_Cluster_4450_sequences=39	504	39
SDS-V3-plasma-67_Cluster_5019_sequences=39	504	39
SDS-V3-plasma-67_Cluster_4764_sequences=39	504	39
SDS-V3-plasma-0_Cluster_1376_sequences=38	0	38
SDS-V3-plasma-0_Cluster_1249_sequences=38	0	38
SDS-V3-plasma-0_Cluster_5071_sequences=38	0	38
SDS-V3-plasma-0_Cluster_960_sequences=38	0	38
SDS-V3-plasma-0_Cluster_2455_sequences=38	0	38
SDS-V3-plasma-0_Cluster_3380_sequences=38	0	38
SDS-V3-plasma-0_Cluster_1470_sequences=38	0	38
SDS-V3-plasma-0_Cluster_1716_sequences=38	0	38
SDS-V3-plasma-0_Cluster_200_sequences=38	0	38
SDS-V3-plasma-0_Cluster_371_sequences=38	0	38
SDS-V3-plasma-0_Cluster_1365_sequences=38	0	38
SDS-V3-plasma-0_Cluster_261_sequences=38	0	38
SDS-V3-plasma-5_Cluster_141_sequences=38	9	38
SDS-V3-plasma-7_Cluster_515_sequences=38	14	38
SDS-V3-plasma-7_Cluster_1967_sequences=38	14	38

SDS-V3-plasma-8_Cluster_2844_sequences=38	16	38
SDS-V3-plasma-24_Cluster_1206_sequences=38	124	38
SDS-V3-plasma-24_Cluster_13_sequences=38	124	38
SDS-V3-plasma-24_Cluster_1690_sequences=38	124	38
SDS-V3-plasma-24_Cluster_1751_sequences=38	124	38
SDS-V3-plasma-24_Cluster_2023_sequences=38	124	38
SDS-V3-plasma-24_Cluster_422_sequences=38	124	38
SDS-V3-plasma-24_Cluster_716_sequences=38	124	38
SDS-V3-plasma-24_Cluster_350_sequences=38	124	38
SDS-V3-plasma-27_Cluster_1546_sequences=38	131	38
SDS-V3-plasma-27_Cluster_508_sequences=38	131	38
SDS-V3-plasma-27_Cluster_566_sequences=38	131	38
SDS-V3-plasma-27_Cluster_642_sequences=38	131	38
SDS-V3-plasma-27_Cluster_7_sequences=38	131	38
SDS-V3-plasma-27_Cluster_809_sequences=38	131	38
SDS-V3-plasma-27_Cluster_388_sequences=38	131	38
SDS-V3-plasma-27_Cluster_1041_sequences=38	131	38
SDS-V3-plasma-27_Cluster_180_sequences=38	131	38
SDS-V3-plasma-45_Cluster_151_sequences=38	282	38
SDS-V3-plasma-45_Cluster_11548_sequences=38	282	38
SDS-V3-plasma-45_Cluster_8645_sequences=38	282	38
SDS-V3-plasma-45_Cluster_10426_sequences=38	282	38
SDS-V3-plasma-45_Cluster_2076_sequences=38	282	38
SDS-V3-plasma-45_Cluster_5373_sequences=38	282	38
SDS-V3-plasma-45_Cluster_5646_sequences=38	282	38
SDS-V3-plasma-45_Cluster_68_sequences=38	282	38
SDS-V3-plasma-45_Cluster_7741_sequences=38	282	38
SDS-V3-plasma-45_Cluster_3753_sequences=38	282	38
SDS-V3-plasma-45_Cluster_7320_sequences=38	282	38
SDS-V3-plasma-45_Cluster_9856_sequences=38	282	38
SDS-V3-plasma-45_Cluster_9120_sequences=38	282	38
SDS-V3-plasma-45_Cluster_11889_sequences=38	282	38
SDS-V3-plasma-45_Cluster_8127_sequences=38	282	38
SDS-V3-plasma-45_Cluster_6667_sequences=38	282	38
SDS-V3-plasma-45_Cluster_5791_sequences=38	282	38
SDS-V3-plasma-45_Cluster_3903_sequences=38	282	38
SDS-V3-plasma-45_Cluster_9197_sequences=38	282	38
SDS-V3-plasma-45_Cluster_108_sequences=38	282	38
SDS-V3-plasma-45_Cluster_2803_sequences=38	282	38
SDS-V3-plasma-45_Cluster_6635_sequences=38	282	38
SDS-V3-plasma-45_Cluster_8819_sequences=38	282	38
SDS-V3-plasma-45_Cluster_3399_sequences=38	282	38
SDS-V3-plasma-45_Cluster_1845_sequences=38	282	38
SDS-V3-plasma-45_Cluster_1510_sequences=38	282	38
SDS-V3-plasma-45_Cluster_1051_sequences=38	282	38

SDS-V3-plasma-45_Cluster_3343_sequences=38	282	38
SDS-V3-plasma-45_Cluster_16501_sequences=38	282	38
SDS-V3-plasma-45_Cluster_8943_sequences=38	282	38
SDS-V3-plasma-45_Cluster_3247_sequences=38	282	38
SDS-V3-plasma-45_Cluster_4462_sequences=38	282	38
SDS-V3-plasma-45_Cluster_6149_sequences=38	282	38
SDS-V3-plasma-45_Cluster_2393_sequences=38	282	38
SDS-V3-plasma-45_Cluster_1611_sequences=38	282	38
SDS-V3-plasma-45_Cluster_13808_sequences=38	282	38
SDS-V3-plasma-45_Cluster_444_sequences=38	282	38
SDS-V3-plasma-45_Cluster_6276_sequences=38	282	38
SDS-V3-plasma-45_Cluster_2756_sequences=38	282	38
SDS-V3-plasma-45_Cluster_8633_sequences=38	282	38
SDS-V3-plasma-46_Cluster_2193_sequences=38	286	38
SDS-V3-plasma-46_Cluster_2107_sequences=38	286	38
SDS-V3-plasma-46_Cluster_361_sequences=38	286	38
SDS-V3-plasma-46_Cluster_21591_sequences=38	286	38
SDS-V3-plasma-46_Cluster_521_sequences=38	286	38
SDS-V3-plasma-46_Cluster_4824_sequences=38	286	38
SDS-V3-plasma-46_Cluster_7234_sequences=38	286	38
SDS-V3-plasma-46_Cluster_809_sequences=38	286	38
SDS-V3-plasma-46_Cluster_1615_sequences=38	286	38
SDS-V3-plasma-67_Cluster_7361_sequences=38	504	38
SDS-V3-plasma-67_Cluster_6223_sequences=38	504	38
SDS-V3-plasma-67_Cluster_6034_sequences=38	504	38
SDS-V3-plasma-67_Cluster_4344_sequences=38	504	38
SDS-V3-plasma-67_Cluster_4829_sequences=38	504	38
SDS-V3-plasma-67_Cluster_3422_sequences=38	504	38
SDS-V3-plasma-67_Cluster_1860_sequences=38	504	38
SDS-V3-plasma-67_Cluster_3132_sequences=38	504	38
SDS-V3-plasma-67_Cluster_17564_sequences=38	504	38
SDS-V3-plasma-67_Cluster_2050_sequences=38	504	38
SDS-V3-plasma-67_Cluster_3115_sequences=38	504	38
SDS-V3-plasma-67_Cluster_2746_sequences=38	504	38
SDS-V3-plasma-67_Cluster_3549_sequences=38	504	38
SDS-V3-plasma-67_Cluster_623_sequences=38	504	38
SDS-V3-plasma-67_Cluster_6817_sequences=38	504	38
SDS-V3-plasma-67_Cluster_5434_sequences=38	504	38
SDS-V3-plasma-67_Cluster_12711_sequences=38	504	38
SDS-V3-plasma-0_Cluster_1554_sequences=37	0	37
SDS-V3-plasma-0_Cluster_1318_sequences=37	0	37
SDS-V3-plasma-0_Cluster_134_sequences=37	0	37
SDS-V3-plasma-0_Cluster_2180_sequences=37	0	37
SDS-V3-plasma-0_Cluster_504_sequences=37	0	37
SDS-V3-plasma-0_Cluster_5412_sequences=37	0	37

SDS-V3-plasma-0_Cluster_797_sequences=37	0	37
SDS-V3-plasma-0_Cluster_1262_sequences=37	0	37
SDS-V3-plasma-0_Cluster_831_sequences=37	0	37
SDS-V3-plasma-0_Cluster_4908_sequences=37	0	37
SDS-V3-plasma-7_Cluster_474_sequences=37	14	37
SDS-V3-plasma-7_Cluster_291_sequences=37	14	37
SDS-V3-plasma-7_Cluster_113_sequences=37	14	37
SDS-V3-plasma-24_Cluster_135_sequences=37	124	37
SDS-V3-plasma-24_Cluster_1951_sequences=37	124	37
SDS-V3-plasma-24_Cluster_288_sequences=37	124	37
SDS-V3-plasma-24_Cluster_862_sequences=37	124	37
SDS-V3-plasma-24_Cluster_857_sequences=37	124	37
SDS-V3-plasma-24_Cluster_560_sequences=37	124	37
SDS-V3-plasma-24_Cluster_554_sequences=37	124	37
SDS-V3-plasma-24_Cluster_713_sequences=37	124	37
SDS-V3-plasma-24_Cluster_860_sequences=37	124	37
SDS-V3-plasma-27_Cluster_109_sequences=37	131	37
SDS-V3-plasma-27_Cluster_132_sequences=37	131	37
SDS-V3-plasma-27_Cluster_1706_sequences=37	131	37
SDS-V3-plasma-27_Cluster_328_sequences=37	131	37
SDS-V3-plasma-27_Cluster_2933_sequences=37	131	37
SDS-V3-plasma-27_Cluster_231_sequences=37	131	37
SDS-V3-plasma-27_Cluster_519_sequences=37	131	37
SDS-V3-plasma-45_Cluster_164_sequences=37	282	37
SDS-V3-plasma-45_Cluster_8320_sequences=37	282	37
SDS-V3-plasma-45_Cluster_1985_sequences=37	282	37
SDS-V3-plasma-45_Cluster_5543_sequences=37	282	37
SDS-V3-plasma-45_Cluster_1101_sequences=37	282	37
SDS-V3-plasma-45_Cluster_12098_sequences=37	282	37
SDS-V3-plasma-45_Cluster_14832_sequences=37	282	37
SDS-V3-plasma-45_Cluster_9476_sequences=37	282	37
SDS-V3-plasma-45_Cluster_4590_sequences=37	282	37
SDS-V3-plasma-45_Cluster_4877_sequences=37	282	37
SDS-V3-plasma-45_Cluster_5031_sequences=37	282	37
SDS-V3-plasma-45_Cluster_222_sequences=37	282	37
SDS-V3-plasma-45_Cluster_3758_sequences=37	282	37
SDS-V3-plasma-45_Cluster_11291_sequences=37	282	37
SDS-V3-plasma-45_Cluster_2235_sequences=37	282	37
SDS-V3-plasma-45_Cluster_2579_sequences=37	282	37
SDS-V3-plasma-45_Cluster_9845_sequences=37	282	37
SDS-V3-plasma-45_Cluster_22257_sequences=37	282	37
SDS-V3-plasma-45_Cluster_14839_sequences=37	282	37
SDS-V3-plasma-45_Cluster_390_sequences=37	282	37
SDS-V3-plasma-45_Cluster_6853_sequences=37	282	37
SDS-V3-plasma-45_Cluster_325_sequences=37	282	37

SDS-V3-plasma-45_Cluster_4817_sequences=37	282	37
SDS-V3-plasma-45_Cluster_6640_sequences=37	282	37
SDS-V3-plasma-45_Cluster_66645_sequences=37	282	37
SDS-V3-plasma-46_Cluster_1709_sequences=37	286	37
SDS-V3-plasma-46_Cluster_571_sequences=37	286	37
SDS-V3-plasma-46_Cluster_1612_sequences=37	286	37
SDS-V3-plasma-46_Cluster_1792_sequences=37	286	37
SDS-V3-plasma-46_Cluster_836_sequences=37	286	37
SDS-V3-plasma-46_Cluster_1752_sequences=37	286	37
SDS-V3-plasma-46_Cluster_2352_sequences=37	286	37
SDS-V3-plasma-46_Cluster_2665_sequences=37	286	37
SDS-V3-plasma-46_Cluster_4566_sequences=37	286	37
SDS-V3-plasma-46_Cluster_732_sequences=37	286	37
SDS-V3-plasma-46_Cluster_388_sequences=37	286	37
SDS-V3-plasma-46_Cluster_2811_sequences=37	286	37
SDS-V3-plasma-46_Cluster_1801_sequences=37	286	37
SDS-V3-plasma-46_Cluster_2707_sequences=37	286	37
SDS-V3-plasma-46_Cluster_3044_sequences=37	286	37
SDS-V3-plasma-46_Cluster_584_sequences=37	286	37
SDS-V3-plasma-46_Cluster_2159_sequences=37	286	37
SDS-V3-plasma-67_Cluster_7428_sequences=37	504	37
SDS-V3-plasma-67_Cluster_3191_sequences=37	504	37
SDS-V3-plasma-67_Cluster_7608_sequences=37	504	37
SDS-V3-plasma-67_Cluster_20068_sequences=37	504	37
SDS-V3-plasma-67_Cluster_7778_sequences=37	504	37
SDS-V3-plasma-67_Cluster_14433_sequences=37	504	37
SDS-V3-plasma-67_Cluster_3771_sequences=37	504	37
SDS-V3-plasma-67_Cluster_8376_sequences=37	504	37
SDS-V3-plasma-67_Cluster_12031_sequences=37	504	37
SDS-V3-plasma-67_Cluster_1300_sequences=37	504	37
SDS-V3-plasma-67_Cluster_37_sequences=37	504	37
SDS-V3-plasma-67_Cluster_1060_sequences=37	504	37
SDS-V3-plasma-67_Cluster_5756_sequences=37	504	37
SDS-V3-plasma-67_Cluster_2131_sequences=37	504	37
SDS-V3-plasma-67_Cluster_1128_sequences=37	504	37
SDS-V3-plasma-67_Cluster_6311_sequences=37	504	37
SDS-V3-plasma-0_Cluster_4884_sequences=36	0	36
SDS-V3-plasma-0_Cluster_1222_sequences=36	0	36
SDS-V3-plasma-0_Cluster_2611_sequences=36	0	36
SDS-V3-plasma-0_Cluster_382_sequences=36	0	36
SDS-V3-plasma-0_Cluster_1571_sequences=36	0	36
SDS-V3-plasma-0_Cluster_679_sequences=36	0	36
SDS-V3-plasma-0_Cluster_3004_sequences=36	0	36
SDS-V3-plasma-0_Cluster_688_sequences=36	0	36
SDS-V3-plasma-0_Cluster_1010_sequences=36	0	36

SDS-V3-plasma-0_Cluster_1486_sequences=36	0	36
SDS-V3-plasma-0_Cluster_1744_sequences=36	0	36
SDS-V3-plasma-0_Cluster_2864_sequences=36	0	36
SDS-V3-plasma-0_Cluster_464_sequences=36	0	36
SDS-V3-plasma-0_Cluster_2835_sequences=36	0	36
SDS-V3-plasma-0_Cluster_1802_sequences=36	0	36
SDS-V3-plasma-5_Cluster_92_sequences=36	9	36
SDS-V3-plasma-7_Cluster_910_sequences=36	14	36
SDS-V3-plasma-7_Cluster_1405_sequences=36	14	36
SDS-V3-plasma-24_Cluster_23_sequences=36	124	36
SDS-V3-plasma-24_Cluster_1310_sequences=36	124	36
SDS-V3-plasma-24_Cluster_589_sequences=36	124	36
SDS-V3-plasma-24_Cluster_823_sequences=36	124	36
SDS-V3-plasma-24_Cluster_107_sequences=36	124	36
SDS-V3-plasma-27_Cluster_1606_sequences=36	131	36
SDS-V3-plasma-27_Cluster_2019_sequences=36	131	36
SDS-V3-plasma-27_Cluster_376_sequences=36	131	36
SDS-V3-plasma-27_Cluster_568_sequences=36	131	36
SDS-V3-plasma-27_Cluster_76_sequences=36	131	36
SDS-V3-plasma-27_Cluster_1269_sequences=36	131	36
SDS-V3-plasma-27_Cluster_2006_sequences=36	131	36
SDS-V3-plasma-45_Cluster_6438_sequences=36	282	36
SDS-V3-plasma-45_Cluster_2121_sequences=36	282	36
SDS-V3-plasma-45_Cluster_5160_sequences=36	282	36
SDS-V3-plasma-45_Cluster_10739_sequences=36	282	36
SDS-V3-plasma-45_Cluster_1902_sequences=36	282	36
SDS-V3-plasma-45_Cluster_3069_sequences=36	282	36
SDS-V3-plasma-45_Cluster_4255_sequences=36	282	36
SDS-V3-plasma-45_Cluster_1496_sequences=36	282	36
SDS-V3-plasma-45_Cluster_8947_sequences=36	282	36
SDS-V3-plasma-45_Cluster_2636_sequences=36	282	36
SDS-V3-plasma-45_Cluster_1707_sequences=36	282	36
SDS-V3-plasma-45_Cluster_2486_sequences=36	282	36
SDS-V3-plasma-45_Cluster_236_sequences=36	282	36
SDS-V3-plasma-45_Cluster_2512_sequences=36	282	36
SDS-V3-plasma-45_Cluster_737_sequences=36	282	36
SDS-V3-plasma-45_Cluster_6571_sequences=36	282	36
SDS-V3-plasma-45_Cluster_11737_sequences=36	282	36
SDS-V3-plasma-45_Cluster_10616_sequences=36	282	36
SDS-V3-plasma-45_Cluster_2534_sequences=36	282	36
SDS-V3-plasma-45_Cluster_8122_sequences=36	282	36
SDS-V3-plasma-45_Cluster_2169_sequences=36	282	36
SDS-V3-plasma-45_Cluster_27080_sequences=36	282	36
SDS-V3-plasma-45_Cluster_7168_sequences=36	282	36
SDS-V3-plasma-45_Cluster_6899_sequences=36	282	36

SDS-V3-plasma-45_Cluster_7798_sequences=36	282	36
SDS-V3-plasma-45_Cluster_4374_sequences=36	282	36
SDS-V3-plasma-45_Cluster_13129_sequences=36	282	36
SDS-V3-plasma-45_Cluster_6527_sequences=36	282	36
SDS-V3-plasma-45_Cluster_4820_sequences=36	282	36
SDS-V3-plasma-45_Cluster_8160_sequences=36	282	36
SDS-V3-plasma-45_Cluster_11174_sequences=36	282	36
SDS-V3-plasma-45_Cluster_3151_sequences=36	282	36
SDS-V3-plasma-46_Cluster_583_sequences=36	286	36
SDS-V3-plasma-46_Cluster_1736_sequences=36	286	36
SDS-V3-plasma-46_Cluster_2468_sequences=36	286	36
SDS-V3-plasma-46_Cluster_2628_sequences=36	286	36
SDS-V3-plasma-46_Cluster_938_sequences=36	286	36
SDS-V3-plasma-46_Cluster_627_sequences=36	286	36
SDS-V3-plasma-46_Cluster_787_sequences=36	286	36
SDS-V3-plasma-46_Cluster_1574_sequences=36	286	36
SDS-V3-plasma-67_Cluster_795_sequences=36	504	36
SDS-V3-plasma-67_Cluster_2577_sequences=36	504	36
SDS-V3-plasma-67_Cluster_22479_sequences=36	504	36
SDS-V3-plasma-67_Cluster_4767_sequences=36	504	36
SDS-V3-plasma-67_Cluster_5781_sequences=36	504	36
SDS-V3-plasma-67_Cluster_6768_sequences=36	504	36
SDS-V3-plasma-67_Cluster_9431_sequences=36	504	36
SDS-V3-plasma-67_Cluster_2147_sequences=36	504	36
SDS-V3-plasma-67_Cluster_6901_sequences=36	504	36
SDS-V3-plasma-67_Cluster_1033_sequences=36	504	36
SDS-V3-plasma-67_Cluster_4310_sequences=36	504	36
SDS-V3-plasma-67_Cluster_20620_sequences=36	504	36
SDS-V3-plasma-67_Cluster_6141_sequences=36	504	36
SDS-V3-plasma-67_Cluster_11829_sequences=36	504	36
SDS-V3-PBMC-5_Cluster_31_sequences=35	9	35
SDS-V3-plasma-0_Cluster_1529_sequences=35	0	35
SDS-V3-plasma-0_Cluster_2055_sequences=35	0	35
SDS-V3-plasma-0_Cluster_2806_sequences=35	0	35
SDS-V3-plasma-0_Cluster_6994_sequences=35	0	35
SDS-V3-plasma-0_Cluster_1489_sequences=35	0	35
SDS-V3-plasma-0_Cluster_1263_sequences=35	0	35
SDS-V3-plasma-0_Cluster_1319_sequences=35	0	35
SDS-V3-plasma-0_Cluster_3660_sequences=35	0	35
SDS-V3-plasma-0_Cluster_492_sequences=35	0	35
SDS-V3-plasma-0_Cluster_770_sequences=35	0	35
SDS-V3-plasma-0_Cluster_409_sequences=35	0	35
SDS-V3-plasma-5_Cluster_598_sequences=35	9	35
SDS-V3-plasma-5_Cluster_163_sequences=35	9	35
SDS-V3-plasma-7_Cluster_511_sequences=35	14	35

SDS-V3-plasma-7_Cluster_358_sequences=35	14	35
SDS-V3-plasma-7_Cluster_69_sequences=35	14	35
SDS-V3-plasma-7_Cluster_1835_sequences=35	14	35
SDS-V3-plasma-8_Cluster_202_sequences=35	16	35
SDS-V3-plasma-8_Cluster_2714_sequences=35	16	35
SDS-V3-plasma-8_Cluster_2564_sequences=35	16	35
SDS-V3-plasma-8_Cluster_2655_sequences=35	16	35
SDS-V3-plasma-8_Cluster_3220_sequences=35	16	35
SDS-V3-plasma-8_Cluster_2896_sequences=35	16	35
SDS-V3-plasma-24_Cluster_228_sequences=35	124	35
SDS-V3-plasma-24_Cluster_25_sequences=35	124	35
SDS-V3-plasma-24_Cluster_426_sequences=35	124	35
SDS-V3-plasma-24_Cluster_443_sequences=35	124	35
SDS-V3-plasma-24_Cluster_497_sequences=35	124	35
SDS-V3-plasma-24_Cluster_239_sequences=35	124	35
SDS-V3-plasma-24_Cluster_449_sequences=35	124	35
SDS-V3-plasma-24_Cluster_121_sequences=35	124	35
SDS-V3-plasma-27_Cluster_1598_sequences=35	131	35
SDS-V3-plasma-27_Cluster_189_sequences=35	131	35
SDS-V3-plasma-27_Cluster_2219_sequences=35	131	35
SDS-V3-plasma-27_Cluster_407_sequences=35	131	35
SDS-V3-plasma-27_Cluster_948_sequences=35	131	35
SDS-V3-plasma-45_Cluster_523_sequences=35	282	35
SDS-V3-plasma-45_Cluster_7451_sequences=35	282	35
SDS-V3-plasma-45_Cluster_1373_sequences=35	282	35
SDS-V3-plasma-45_Cluster_3580_sequences=35	282	35
SDS-V3-plasma-45_Cluster_3923_sequences=35	282	35
SDS-V3-plasma-45_Cluster_1186_sequences=35	282	35
SDS-V3-plasma-45_Cluster_3276_sequences=35	282	35
SDS-V3-plasma-45_Cluster_228_sequences=35	282	35
SDS-V3-plasma-45_Cluster_12387_sequences=35	282	35
SDS-V3-plasma-45_Cluster_9207_sequences=35	282	35
SDS-V3-plasma-45_Cluster_3889_sequences=35	282	35
SDS-V3-plasma-45_Cluster_8237_sequences=35	282	35
SDS-V3-plasma-45_Cluster_49_sequences=35	282	35
SDS-V3-plasma-45_Cluster_3759_sequences=35	282	35
SDS-V3-plasma-45_Cluster_3121_sequences=35	282	35
SDS-V3-plasma-45_Cluster_3628_sequences=35	282	35
SDS-V3-plasma-45_Cluster_5409_sequences=35	282	35
SDS-V3-plasma-45_Cluster_2973_sequences=35	282	35
SDS-V3-plasma-45_Cluster_4760_sequences=35	282	35
SDS-V3-plasma-45_Cluster_1384_sequences=35	282	35
SDS-V3-plasma-45_Cluster_7403_sequences=35	282	35
SDS-V3-plasma-45_Cluster_4236_sequences=35	282	35
SDS-V3-plasma-45_Cluster_9479_sequences=35	282	35

SDS-V3-plasma-45_Cluster_3449_sequences=35	282	35
SDS-V3-plasma-45_Cluster_6299_sequences=35	282	35
SDS-V3-plasma-45_Cluster_1489_sequences=35	282	35
SDS-V3-plasma-45_Cluster_3867_sequences=35	282	35
SDS-V3-plasma-45_Cluster_6747_sequences=35	282	35
SDS-V3-plasma-45_Cluster_12872_sequences=35	282	35
SDS-V3-plasma-45_Cluster_1566_sequences=35	282	35
SDS-V3-plasma-45_Cluster_1592_sequences=35	282	35
SDS-V3-plasma-45_Cluster_31352_sequences=35	282	35
SDS-V3-plasma-45_Cluster_66624_sequences=35	282	35
SDS-V3-plasma-46_Cluster_2859_sequences=35	286	35
SDS-V3-plasma-46_Cluster_5668_sequences=35	286	35
SDS-V3-plasma-46_Cluster_287_sequences=35	286	35
SDS-V3-plasma-46_Cluster_2769_sequences=35	286	35
SDS-V3-plasma-46_Cluster_383_sequences=35	286	35
SDS-V3-plasma-46_Cluster_576_sequences=35	286	35
SDS-V3-plasma-46_Cluster_3868_sequences=35	286	35
SDS-V3-plasma-46_Cluster_2176_sequences=35	286	35
SDS-V3-plasma-46_Cluster_3615_sequences=35	286	35
SDS-V3-plasma-67_Cluster_351_sequences=35	504	35
SDS-V3-plasma-67_Cluster_13623_sequences=35	504	35
SDS-V3-plasma-67_Cluster_9053_sequences=35	504	35
SDS-V3-plasma-67_Cluster_7298_sequences=35	504	35
SDS-V3-plasma-67_Cluster_6854_sequences=35	504	35
SDS-V3-plasma-67_Cluster_7412_sequences=35	504	35
SDS-V3-plasma-67_Cluster_1773_sequences=35	504	35
SDS-V3-plasma-67_Cluster_4091_sequences=35	504	35
SDS-V3-plasma-67_Cluster_4492_sequences=35	504	35
SDS-V3-plasma-67_Cluster_431_sequences=35	504	35
SDS-V3-plasma-67_Cluster_1505_sequences=35	504	35
SDS-V3-plasma-67_Cluster_2767_sequences=35	504	35
SDS-V3-plasma-67_Cluster_4869_sequences=35	504	35
SDS-V3-plasma-0_Cluster_1953_sequences=34	0	34
SDS-V3-plasma-0_Cluster_1576_sequences=34	0	34
SDS-V3-plasma-0_Cluster_2332_sequences=34	0	34
SDS-V3-plasma-0_Cluster_4768_sequences=34	0	34
SDS-V3-plasma-0_Cluster_5179_sequences=34	0	34
SDS-V3-plasma-0_Cluster_977_sequences=34	0	34
SDS-V3-plasma-0_Cluster_576_sequences=34	0	34
SDS-V3-plasma-0_Cluster_1514_sequences=34	0	34
SDS-V3-plasma-0_Cluster_2092_sequences=34	0	34
SDS-V3-plasma-0_Cluster_59_sequences=34	0	34
SDS-V3-plasma-0_Cluster_7414_sequences=34	0	34
SDS-V3-plasma-0_Cluster_12901_sequences=34	0	34
SDS-V3-plasma-0_Cluster_3927_sequences=34	0	34

SDS-V3-plasma-0_Cluster_1850_sequences=34	0	34
SDS-V3-plasma-0_Cluster_1896_sequences=34	0	34
SDS-V3-plasma-0_Cluster_248_sequences=34	0	34
SDS-V3-plasma-0_Cluster_3139_sequences=34	0	34
SDS-V3-plasma-0_Cluster_32672_sequences=34	0	34
SDS-V3-plasma-7_Cluster_135_sequences=34	14	34
SDS-V3-plasma-7_Cluster_138_sequences=34	14	34
SDS-V3-plasma-7_Cluster_359_sequences=34	14	34
SDS-V3-plasma-8_Cluster_288_sequences=34	16	34
SDS-V3-plasma-8_Cluster_2410_sequences=34	16	34
SDS-V3-plasma-8_Cluster_2904_sequences=34	16	34
SDS-V3-plasma-8_Cluster_2474_sequences=34	16	34
SDS-V3-plasma-8_Cluster_225_sequences=34	16	34
SDS-V3-plasma-8_Cluster_2749_sequences=34	16	34
SDS-V3-plasma-24_Cluster_1085_sequences=34	124	34
SDS-V3-plasma-24_Cluster_238_sequences=34	124	34
SDS-V3-plasma-24_Cluster_951_sequences=34	124	34
SDS-V3-plasma-24_Cluster_2773_sequences=34	124	34
SDS-V3-plasma-24_Cluster_573_sequences=34	124	34
SDS-V3-plasma-24_Cluster_1514_sequences=34	124	34
SDS-V3-plasma-24_Cluster_963_sequences=34	124	34
SDS-V3-plasma-27_Cluster_118_sequences=34	131	34
SDS-V3-plasma-27_Cluster_269_sequences=34	131	34
SDS-V3-plasma-27_Cluster_276_sequences=34	131	34
SDS-V3-plasma-27_Cluster_393_sequences=34	131	34
SDS-V3-plasma-27_Cluster_555_sequences=34	131	34
SDS-V3-plasma-27_Cluster_2447_sequences=34	131	34
SDS-V3-plasma-27_Cluster_209_sequences=34	131	34
SDS-V3-plasma-27_Cluster_1075_sequences=34	131	34
SDS-V3-plasma-27_Cluster_771_sequences=34	131	34
SDS-V3-plasma-45_Cluster_308_sequences=34	282	34
SDS-V3-plasma-45_Cluster_3398_sequences=34	282	34
SDS-V3-plasma-45_Cluster_2347_sequences=34	282	34
SDS-V3-plasma-45_Cluster_712_sequences=34	282	34
SDS-V3-plasma-45_Cluster_19441_sequences=34	282	34
SDS-V3-plasma-45_Cluster_4849_sequences=34	282	34
SDS-V3-plasma-45_Cluster_8428_sequences=34	282	34
SDS-V3-plasma-45_Cluster_3814_sequences=34	282	34
SDS-V3-plasma-45_Cluster_8929_sequences=34	282	34
SDS-V3-plasma-45_Cluster_12815_sequences=34	282	34
SDS-V3-plasma-45_Cluster_3577_sequences=34	282	34
SDS-V3-plasma-45_Cluster_890_sequences=34	282	34
SDS-V3-plasma-45_Cluster_6022_sequences=34	282	34
SDS-V3-plasma-45_Cluster_918_sequences=34	282	34
SDS-V3-plasma-45_Cluster_3315_sequences=34	282	34

SDS-V3-plasma-45_Cluster_14064_sequences=34	282	34
SDS-V3-plasma-45_Cluster_14067_sequences=34	282	34
SDS-V3-plasma-45_Cluster_3335_sequences=34	282	34
SDS-V3-plasma-45_Cluster_943_sequences=34	282	34
SDS-V3-plasma-45_Cluster_9674_sequences=34	282	34
SDS-V3-plasma-45_Cluster_2683_sequences=34	282	34
SDS-V3-plasma-45_Cluster_17268_sequences=34	282	34
SDS-V3-plasma-45_Cluster_11801_sequences=34	282	34
SDS-V3-plasma-45_Cluster_10041_sequences=34	282	34
SDS-V3-plasma-45_Cluster_4026_sequences=34	282	34
SDS-V3-plasma-45_Cluster_5541_sequences=34	282	34
SDS-V3-plasma-45_Cluster_5606_sequences=34	282	34
SDS-V3-plasma-45_Cluster_7739_sequences=34	282	34
SDS-V3-plasma-45_Cluster_12062_sequences=34	282	34
SDS-V3-plasma-45_Cluster_10757_sequences=34	282	34
SDS-V3-plasma-45_Cluster_4475_sequences=34	282	34
SDS-V3-plasma-45_Cluster_694_sequences=34	282	34
SDS-V3-plasma-45_Cluster_2667_sequences=34	282	34
SDS-V3-plasma-46_Cluster_4235_sequences=34	286	34
SDS-V3-plasma-46_Cluster_6185_sequences=34	286	34
SDS-V3-plasma-46_Cluster_3135_sequences=34	286	34
SDS-V3-plasma-46_Cluster_3785_sequences=34	286	34
SDS-V3-plasma-46_Cluster_3120_sequences=34	286	34
SDS-V3-plasma-46_Cluster_767_sequences=34	286	34
SDS-V3-plasma-46_Cluster_731_sequences=34	286	34
SDS-V3-plasma-46_Cluster_1061_sequences=34	286	34
SDS-V3-plasma-46_Cluster_2294_sequences=34	286	34
SDS-V3-plasma-67_Cluster_1018_sequences=34	504	34
SDS-V3-plasma-67_Cluster_8113_sequences=34	504	34
SDS-V3-plasma-67_Cluster_10583_sequences=34	504	34
SDS-V3-plasma-67_Cluster_1949_sequences=34	504	34
SDS-V3-plasma-67_Cluster_4318_sequences=34	504	34
SDS-V3-plasma-67_Cluster_739_sequences=34	504	34
SDS-V3-plasma-67_Cluster_978_sequences=34	504	34
SDS-V3-plasma-67_Cluster_20839_sequences=34	504	34
SDS-V3-plasma-67_Cluster_24975_sequences=34	504	34
SDS-V3-plasma-67_Cluster_334_sequences=34	504	34
SDS-V3-plasma-67_Cluster_4272_sequences=34	504	34
SDS-V3-plasma-67_Cluster_6527_sequences=34	504	34
SDS-V3-plasma-67_Cluster_6697_sequences=34	504	34
SDS-V3-plasma-67_Cluster_6738_sequences=34	504	34
SDS-V3-plasma-67_Cluster_1804_sequences=34	504	34
SDS-V3-plasma-67_Cluster_5531_sequences=34	504	34
SDS-V3-plasma-67_Cluster_11456_sequences=34	504	34
SDS-V3-plasma-67_Cluster_310_sequences=34	504	34

SDS-V3-plasma-67_Cluster_10860_sequences=34	504	34
SDS-V3-plasma-67_Cluster_86_sequences=34	504	34
SDS-V3-plasma-67_Cluster_10302_sequences=34	504	34
SDS-V3-plasma-67_Cluster_2077_sequences=34	504	34
SDS-V3-PBMC-5_Cluster_526_sequences=33	9	33
SDS-V3-plasma-0_Cluster_407_sequences=33	0	33
SDS-V3-plasma-0_Cluster_605_sequences=33	0	33
SDS-V3-plasma-0_Cluster_1581_sequences=33	0	33
SDS-V3-plasma-0_Cluster_1418_sequences=33	0	33
SDS-V3-plasma-0_Cluster_380_sequences=33	0	33
SDS-V3-plasma-0_Cluster_1142_sequences=33	0	33
SDS-V3-plasma-0_Cluster_252_sequences=33	0	33
SDS-V3-plasma-0_Cluster_2972_sequences=33	0	33
SDS-V3-plasma-0_Cluster_627_sequences=33	0	33
SDS-V3-plasma-0_Cluster_555_sequences=33	0	33
SDS-V3-plasma-0_Cluster_408_sequences=33	0	33
SDS-V3-plasma-0_Cluster_356_sequences=33	0	33
SDS-V3-plasma-0_Cluster_1241_sequences=33	0	33
SDS-V3-plasma-0_Cluster_936_sequences=33	0	33
SDS-V3-plasma-0_Cluster_3746_sequences=33	0	33
SDS-V3-plasma-0_Cluster_2758_sequences=33	0	33
SDS-V3-plasma-0_Cluster_1154_sequences=33	0	33
SDS-V3-plasma-0_Cluster_1714_sequences=33	0	33
SDS-V3-plasma-5_Cluster_8_sequences=33	9	33
SDS-V3-plasma-7_Cluster_646_sequences=33	14	33
SDS-V3-plasma-7_Cluster_799_sequences=33	14	33
SDS-V3-plasma-8_Cluster_2424_sequences=33	16	33
SDS-V3-plasma-8_Cluster_3484_sequences=33	16	33
SDS-V3-plasma-24_Cluster_111_sequences=33	124	33
SDS-V3-plasma-24_Cluster_436_sequences=33	124	33
SDS-V3-plasma-24_Cluster_513_sequences=33	124	33
SDS-V3-plasma-24_Cluster_875_sequences=33	124	33
SDS-V3-plasma-24_Cluster_2052_sequences=33	124	33
SDS-V3-plasma-24_Cluster_1488_sequences=33	124	33
SDS-V3-plasma-24_Cluster_1363_sequences=33	124	33
SDS-V3-plasma-24_Cluster_355_sequences=33	124	33
SDS-V3-plasma-24_Cluster_149_sequences=33	124	33
SDS-V3-plasma-27_Cluster_1112_sequences=33	131	33
SDS-V3-plasma-27_Cluster_1225_sequences=33	131	33
SDS-V3-plasma-27_Cluster_15_sequences=33	131	33
SDS-V3-plasma-27_Cluster_495_sequences=33	131	33
SDS-V3-plasma-27_Cluster_1682_sequences=33	131	33
SDS-V3-plasma-27_Cluster_255_sequences=33	131	33
SDS-V3-plasma-27_Cluster_975_sequences=33	131	33
SDS-V3-plasma-27_Cluster_1233_sequences=33	131	33

SDS-V3-plasma-27_Cluster_2112_sequences=33	131	33
SDS-V3-plasma-27_Cluster_580_sequences=33	131	33
SDS-V3-plasma-27_Cluster_87_sequences=33	131	33
SDS-V3-plasma-45_Cluster_11866_sequences=33	282	33
SDS-V3-plasma-45_Cluster_13204_sequences=33	282	33
SDS-V3-plasma-45_Cluster_13022_sequences=33	282	33
SDS-V3-plasma-45_Cluster_1879_sequences=33	282	33
SDS-V3-plasma-45_Cluster_3681_sequences=33	282	33
SDS-V3-plasma-45_Cluster_485_sequences=33	282	33
SDS-V3-plasma-45_Cluster_647_sequences=33	282	33
SDS-V3-plasma-45_Cluster_3125_sequences=33	282	33
SDS-V3-plasma-45_Cluster_14923_sequences=33	282	33
SDS-V3-plasma-45_Cluster_2686_sequences=33	282	33
SDS-V3-plasma-45_Cluster_5161_sequences=33	282	33
SDS-V3-plasma-45_Cluster_1470_sequences=33	282	33
SDS-V3-plasma-45_Cluster_15223_sequences=33	282	33
SDS-V3-plasma-45_Cluster_2763_sequences=33	282	33
SDS-V3-plasma-45_Cluster_551_sequences=33	282	33
SDS-V3-plasma-45_Cluster_9732_sequences=33	282	33
SDS-V3-plasma-45_Cluster_5597_sequences=33	282	33
SDS-V3-plasma-45_Cluster_10481_sequences=33	282	33
SDS-V3-plasma-45_Cluster_17865_sequences=33	282	33
SDS-V3-plasma-45_Cluster_4355_sequences=33	282	33
SDS-V3-plasma-45_Cluster_55973_sequences=33	282	33
SDS-V3-plasma-45_Cluster_286_sequences=33	282	33
SDS-V3-plasma-45_Cluster_3470_sequences=33	282	33
SDS-V3-plasma-45_Cluster_2983_sequences=33	282	33
SDS-V3-plasma-45_Cluster_66647_sequences=33	282	33
SDS-V3-plasma-45_Cluster_7004_sequences=33	282	33
SDS-V3-plasma-45_Cluster_6_sequences=33	282	33
SDS-V3-plasma-45_Cluster_2632_sequences=33	282	33
SDS-V3-plasma-45_Cluster_2463_sequences=33	282	33
SDS-V3-plasma-45_Cluster_17953_sequences=33	282	33
SDS-V3-plasma-45_Cluster_217_sequences=33	282	33
SDS-V3-plasma-45_Cluster_10145_sequences=33	282	33
SDS-V3-plasma-45_Cluster_18516_sequences=33	282	33
SDS-V3-plasma-45_Cluster_3531_sequences=33	282	33
SDS-V3-plasma-46_Cluster_2340_sequences=33	286	33
SDS-V3-plasma-46_Cluster_1492_sequences=33	286	33
SDS-V3-plasma-46_Cluster_148_sequences=33	286	33
SDS-V3-plasma-46_Cluster_1579_sequences=33	286	33
SDS-V3-plasma-46_Cluster_1499_sequences=33	286	33
SDS-V3-plasma-46_Cluster_1780_sequences=33	286	33
SDS-V3-plasma-46_Cluster_68_sequences=33	286	33
SDS-V3-plasma-46_Cluster_4574_sequences=33	286	33

SDS-V3-plasma-67_Cluster_9213_sequences=33	504	33
SDS-V3-plasma-67_Cluster_2735_sequences=33	504	33
SDS-V3-plasma-67_Cluster_7906_sequences=33	504	33
SDS-V3-plasma-67_Cluster_1339_sequences=33	504	33
SDS-V3-plasma-67_Cluster_445_sequences=33	504	33
SDS-V3-plasma-67_Cluster_9375_sequences=33	504	33
SDS-V3-plasma-67_Cluster_10374_sequences=33	504	33
SDS-V3-plasma-67_Cluster_193_sequences=33	504	33
SDS-V3-plasma-67_Cluster_217_sequences=33	504	33
SDS-V3-plasma-67_Cluster_4533_sequences=33	504	33
SDS-V3-plasma-67_Cluster_4891_sequences=33	504	33
SDS-V3-plasma-67_Cluster_5147_sequences=33	504	33
SDS-V3-plasma-67_Cluster_5372_sequences=33	504	33
SDS-V3-plasma-67_Cluster_5494_sequences=33	504	33
SDS-V3-plasma-67_Cluster_2641_sequences=33	504	33
SDS-V3-plasma-67_Cluster_1442_sequences=33	504	33
SDS-V3-plasma-67_Cluster_7082_sequences=33	504	33
SDS-V3-plasma-67_Cluster_6211_sequences=33	504	33
SDS-V3-plasma-67_Cluster_1928_sequences=33	504	33
SDS-V3-plasma-0_Cluster_623_sequences=32	0	32
SDS-V3-plasma-0_Cluster_4020_sequences=32	0	32
SDS-V3-plasma-0_Cluster_1728_sequences=32	0	32
SDS-V3-plasma-0_Cluster_55_sequences=32	0	32
SDS-V3-plasma-0_Cluster_132_sequences=32	0	32
SDS-V3-plasma-0_Cluster_549_sequences=32	0	32
SDS-V3-plasma-0_Cluster_1158_sequences=32	0	32
SDS-V3-plasma-0_Cluster_1903_sequences=32	0	32
SDS-V3-plasma-0_Cluster_5175_sequences=32	0	32
SDS-V3-plasma-0_Cluster_6358_sequences=32	0	32
SDS-V3-plasma-0_Cluster_643_sequences=32	0	32
SDS-V3-plasma-0_Cluster_1393_sequences=32	0	32
SDS-V3-plasma-0_Cluster_2993_sequences=32	0	32
SDS-V3-plasma-0_Cluster_9779_sequences=32	0	32
SDS-V3-plasma-0_Cluster_3858_sequences=32	0	32
SDS-V3-plasma-0_Cluster_568_sequences=32	0	32
SDS-V3-plasma-0_Cluster_471_sequences=32	0	32
SDS-V3-plasma-5_Cluster_30_sequences=32	9	32
SDS-V3-plasma-7_Cluster_395_sequences=32	14	32
SDS-V3-plasma-7_Cluster_773_sequences=32	14	32
SDS-V3-plasma-7_Cluster_928_sequences=32	14	32
SDS-V3-plasma-7_Cluster_379_sequences=32	14	32
SDS-V3-plasma-7_Cluster_1594_sequences=32	14	32
SDS-V3-plasma-8_Cluster_3469_sequences=32	16	32
SDS-V3-plasma-8_Cluster_2853_sequences=32	16	32
SDS-V3-plasma-24_Cluster_1813_sequences=32	124	32

SDS-V3-plasma-24_Cluster_197_sequences=32	124	32
SDS-V3-plasma-24_Cluster_2274_sequences=32	124	32
SDS-V3-plasma-24_Cluster_58_sequences=32	124	32
SDS-V3-plasma-24_Cluster_856_sequences=32	124	32
SDS-V3-plasma-24_Cluster_678_sequences=32	124	32
SDS-V3-plasma-24_Cluster_133_sequences=32	124	32
SDS-V3-plasma-24_Cluster_2353_sequences=32	124	32
SDS-V3-plasma-24_Cluster_679_sequences=32	124	32
SDS-V3-plasma-24_Cluster_358_sequences=32	124	32
SDS-V3-plasma-27_Cluster_1460_sequences=32	131	32
SDS-V3-plasma-27_Cluster_1573_sequences=32	131	32
SDS-V3-plasma-27_Cluster_668_sequences=32	131	32
SDS-V3-plasma-27_Cluster_315_sequences=32	131	32
SDS-V3-plasma-27_Cluster_170_sequences=32	131	32
SDS-V3-plasma-27_Cluster_632_sequences=32	131	32
SDS-V3-plasma-27_Cluster_538_sequences=32	131	32
SDS-V3-plasma-27_Cluster_1031_sequences=32	131	32
SDS-V3-plasma-27_Cluster_579_sequences=32	131	32
SDS-V3-plasma-27_Cluster_586_sequences=32	131	32
SDS-V3-plasma-27_Cluster_833_sequences=32	131	32
SDS-V3-plasma-45_Cluster_9211_sequences=32	282	32
SDS-V3-plasma-45_Cluster_20832_sequences=32	282	32
SDS-V3-plasma-45_Cluster_1434_sequences=32	282	32
SDS-V3-plasma-45_Cluster_550_sequences=32	282	32
SDS-V3-plasma-45_Cluster_12893_sequences=32	282	32
SDS-V3-plasma-45_Cluster_10257_sequences=32	282	32
SDS-V3-plasma-45_Cluster_5990_sequences=32	282	32
SDS-V3-plasma-45_Cluster_9961_sequences=32	282	32
SDS-V3-plasma-45_Cluster_1604_sequences=32	282	32
SDS-V3-plasma-45_Cluster_5181_sequences=32	282	32
SDS-V3-plasma-45_Cluster_2171_sequences=32	282	32
SDS-V3-plasma-45_Cluster_3312_sequences=32	282	32
SDS-V3-plasma-45_Cluster_10847_sequences=32	282	32
SDS-V3-plasma-45_Cluster_15903_sequences=32	282	32
SDS-V3-plasma-45_Cluster_1981_sequences=32	282	32
SDS-V3-plasma-45_Cluster_2387_sequences=32	282	32
SDS-V3-plasma-45_Cluster_885_sequences=32	282	32
SDS-V3-plasma-45_Cluster_3699_sequences=32	282	32
SDS-V3-plasma-45_Cluster_612_sequences=32	282	32
SDS-V3-plasma-45_Cluster_3220_sequences=32	282	32
SDS-V3-plasma-45_Cluster_1692_sequences=32	282	32
SDS-V3-plasma-45_Cluster_3522_sequences=32	282	32
SDS-V3-plasma-45_Cluster_3683_sequences=32	282	32
SDS-V3-plasma-45_Cluster_4044_sequences=32	282	32
SDS-V3-plasma-45_Cluster_9899_sequences=32	282	32

SDS-V3-plasma-45_Cluster_978_sequences=32	282	32
SDS-V3-plasma-45_Cluster_7192_sequences=32	282	32
SDS-V3-plasma-45_Cluster_1416_sequences=32	282	32
SDS-V3-plasma-45_Cluster_2256_sequences=32	282	32
SDS-V3-plasma-45_Cluster_111_sequences=32	282	32
SDS-V3-plasma-45_Cluster_6621_sequences=32	282	32
SDS-V3-plasma-45_Cluster_5649_sequences=32	282	32
SDS-V3-plasma-45_Cluster_7819_sequences=32	282	32
SDS-V3-plasma-45_Cluster_15503_sequences=32	282	32
SDS-V3-plasma-45_Cluster_8340_sequences=32	282	32
SDS-V3-plasma-45_Cluster_2018_sequences=32	282	32
SDS-V3-plasma-45_Cluster_689_sequences=32	282	32
SDS-V3-plasma-45_Cluster_344_sequences=32	282	32
SDS-V3-plasma-45_Cluster_858_sequences=32	282	32
SDS-V3-plasma-46_Cluster_2413_sequences=32	286	32
SDS-V3-plasma-46_Cluster_1812_sequences=32	286	32
SDS-V3-plasma-46_Cluster_2696_sequences=32	286	32
SDS-V3-plasma-46_Cluster_6195_sequences=32	286	32
SDS-V3-plasma-46_Cluster_2642_sequences=32	286	32
SDS-V3-plasma-46_Cluster_3014_sequences=32	286	32
SDS-V3-plasma-46_Cluster_2303_sequences=32	286	32
SDS-V3-plasma-46_Cluster_2247_sequences=32	286	32
SDS-V3-plasma-46_Cluster_3001_sequences=32	286	32
SDS-V3-plasma-46_Cluster_5375_sequences=32	286	32
SDS-V3-plasma-67_Cluster_1494_sequences=32	504	32
SDS-V3-plasma-67_Cluster_5569_sequences=32	504	32
SDS-V3-plasma-67_Cluster_11153_sequences=32	504	32
SDS-V3-plasma-67_Cluster_5182_sequences=32	504	32
SDS-V3-plasma-67_Cluster_1307_sequences=32	504	32
SDS-V3-plasma-67_Cluster_5394_sequences=32	504	32
SDS-V3-plasma-67_Cluster_134_sequences=32	504	32
SDS-V3-plasma-67_Cluster_15075_sequences=32	504	32
SDS-V3-plasma-67_Cluster_7083_sequences=32	504	32
SDS-V3-plasma-67_Cluster_32133_sequences=32	504	32
SDS-V3-plasma-67_Cluster_4438_sequences=32	504	32
SDS-V3-plasma-67_Cluster_11483_sequences=32	504	32
SDS-V3-plasma-67_Cluster_3939_sequences=32	504	32
SDS-V3-plasma-67_Cluster_130621_sequences=32	504	32
SDS-V3-PBMC-45_Cluster_23659_sequences=31	282	31
SDS-V3-PBMC-63_Cluster_21_sequences=31	489	31
SDS-V3-plasma-0_Cluster_1873_sequences=31	0	31
SDS-V3-plasma-0_Cluster_1508_sequences=31	0	31
SDS-V3-plasma-0_Cluster_1537_sequences=31	0	31
SDS-V3-plasma-0_Cluster_1286_sequences=31	0	31
SDS-V3-plasma-0_Cluster_2164_sequences=31	0	31

SDS-V3-plasma-0_Cluster_863_sequences=31	0	31
SDS-V3-plasma-0_Cluster_1215_sequences=31	0	31
SDS-V3-plasma-0_Cluster_1912_sequences=31	0	31
SDS-V3-plasma-0_Cluster_2152_sequences=31	0	31
SDS-V3-plasma-0_Cluster_297_sequences=31	0	31
SDS-V3-plasma-0_Cluster_3744_sequences=31	0	31
SDS-V3-plasma-0_Cluster_2046_sequences=31	0	31
SDS-V3-plasma-0_Cluster_6612_sequences=31	0	31
SDS-V3-plasma-0_Cluster_414_sequences=31	0	31
SDS-V3-plasma-0_Cluster_2433_sequences=31	0	31
SDS-V3-plasma-0_Cluster_1923_sequences=31	0	31
SDS-V3-plasma-0_Cluster_590_sequences=31	0	31
SDS-V3-plasma-5_Cluster_319_sequences=31	9	31
SDS-V3-plasma-5_Cluster_577_sequences=31	9	31
SDS-V3-plasma-5_Cluster_672_sequences=31	9	31
SDS-V3-plasma-7_Cluster_775_sequences=31	14	31
SDS-V3-plasma-7_Cluster_290_sequences=31	14	31
SDS-V3-plasma-7_Cluster_674_sequences=31	14	31
SDS-V3-plasma-8_Cluster_2285_sequences=31	16	31
SDS-V3-plasma-8_Cluster_2389_sequences=31	16	31
SDS-V3-plasma-8_Cluster_5763_sequences=31	16	31
SDS-V3-plasma-24_Cluster_1883_sequences=31	124	31
SDS-V3-plasma-24_Cluster_2061_sequences=31	124	31
SDS-V3-plasma-24_Cluster_277_sequences=31	124	31
SDS-V3-plasma-24_Cluster_869_sequences=31	124	31
SDS-V3-plasma-24_Cluster_28_sequences=31	124	31
SDS-V3-plasma-24_Cluster_1807_sequences=31	124	31
SDS-V3-plasma-24_Cluster_314_sequences=31	124	31
SDS-V3-plasma-24_Cluster_2573_sequences=31	124	31
SDS-V3-plasma-24_Cluster_1725_sequences=31	124	31
SDS-V3-plasma-27_Cluster_1032_sequences=31	131	31
SDS-V3-plasma-27_Cluster_1199_sequences=31	131	31
SDS-V3-plasma-27_Cluster_2293_sequences=31	131	31
SDS-V3-plasma-27_Cluster_3423_sequences=31	131	31
SDS-V3-plasma-27_Cluster_445_sequences=31	131	31
SDS-V3-plasma-27_Cluster_606_sequences=31	131	31
SDS-V3-plasma-27_Cluster_898_sequences=31	131	31
SDS-V3-plasma-27_Cluster_1806_sequences=31	131	31
SDS-V3-plasma-27_Cluster_3918_sequences=31	131	31
SDS-V3-plasma-27_Cluster_524_sequences=31	131	31
SDS-V3-plasma-27_Cluster_2210_sequences=31	131	31
SDS-V3-plasma-45_Cluster_3139_sequences=31	282	31
SDS-V3-plasma-45_Cluster_2246_sequences=31	282	31
SDS-V3-plasma-45_Cluster_4362_sequences=31	282	31
SDS-V3-plasma-45_Cluster_14744_sequences=31	282	31

SDS-V3-plasma-45_Cluster_4215_sequences=31	282	31
SDS-V3-plasma-45_Cluster_2334_sequences=31	282	31
SDS-V3-plasma-45_Cluster_11543_sequences=31	282	31
SDS-V3-plasma-45_Cluster_12567_sequences=31	282	31
SDS-V3-plasma-45_Cluster_13748_sequences=31	282	31
SDS-V3-plasma-45_Cluster_17388_sequences=31	282	31
SDS-V3-plasma-45_Cluster_10989_sequences=31	282	31
SDS-V3-plasma-45_Cluster_11958_sequences=31	282	31
SDS-V3-plasma-45_Cluster_2258_sequences=31	282	31
SDS-V3-plasma-45_Cluster_3225_sequences=31	282	31
SDS-V3-plasma-45_Cluster_3646_sequences=31	282	31
SDS-V3-plasma-45_Cluster_946_sequences=31	282	31
SDS-V3-plasma-45_Cluster_9662_sequences=31	282	31
SDS-V3-plasma-45_Cluster_100_sequences=31	282	31
SDS-V3-plasma-45_Cluster_10770_sequences=31	282	31
SDS-V3-plasma-45_Cluster_2529_sequences=31	282	31
SDS-V3-plasma-45_Cluster_521_sequences=31	282	31
SDS-V3-plasma-45_Cluster_5465_sequences=31	282	31
SDS-V3-plasma-45_Cluster_1846_sequences=31	282	31
SDS-V3-plasma-45_Cluster_3881_sequences=31	282	31
SDS-V3-plasma-45_Cluster_8487_sequences=31	282	31
SDS-V3-plasma-45_Cluster_6877_sequences=31	282	31
SDS-V3-plasma-45_Cluster_4254_sequences=31	282	31
SDS-V3-plasma-45_Cluster_12712_sequences=31	282	31
SDS-V3-plasma-45_Cluster_12611_sequences=31	282	31
SDS-V3-plasma-45_Cluster_12235_sequences=31	282	31
SDS-V3-plasma-45_Cluster_1538_sequences=31	282	31
SDS-V3-plasma-45_Cluster_3954_sequences=31	282	31
SDS-V3-plasma-45_Cluster_6565_sequences=31	282	31
SDS-V3-plasma-45_Cluster_68979_sequences=31	282	31
SDS-V3-plasma-45_Cluster_12180_sequences=31	282	31
SDS-V3-plasma-45_Cluster_1072_sequences=31	282	31
SDS-V3-plasma-45_Cluster_5355_sequences=31	282	31
SDS-V3-plasma-45_Cluster_1601_sequences=31	282	31
SDS-V3-plasma-45_Cluster_6126_sequences=31	282	31
SDS-V3-plasma-45_Cluster_9233_sequences=31	282	31
SDS-V3-plasma-45_Cluster_624_sequences=31	282	31
SDS-V3-plasma-45_Cluster_15113_sequences=31	282	31
SDS-V3-plasma-45_Cluster_14399_sequences=31	282	31
SDS-V3-plasma-45_Cluster_193_sequences=31	282	31
SDS-V3-plasma-45_Cluster_4511_sequences=31	282	31
SDS-V3-plasma-45_Cluster_5229_sequences=31	282	31
SDS-V3-plasma-45_Cluster_8680_sequences=31	282	31
SDS-V3-plasma-46_Cluster_1344_sequences=31	286	31
SDS-V3-plasma-46_Cluster_371_sequences=31	286	31

SDS-V3-plasma-46_Cluster_1919_sequences=31	286	31
SDS-V3-plasma-46_Cluster_1557_sequences=31	286	31
SDS-V3-plasma-46_Cluster_2355_sequences=31	286	31
SDS-V3-plasma-46_Cluster_782_sequences=31	286	31
SDS-V3-plasma-46_Cluster_2206_sequences=31	286	31
SDS-V3-plasma-46_Cluster_4990_sequences=31	286	31
SDS-V3-plasma-46_Cluster_5252_sequences=31	286	31
SDS-V3-plasma-46_Cluster_300_sequences=31	286	31
SDS-V3-plasma-46_Cluster_2390_sequences=31	286	31
SDS-V3-plasma-46_Cluster_2727_sequences=31	286	31
SDS-V3-plasma-46_Cluster_1970_sequences=31	286	31
SDS-V3-plasma-67_Cluster_10940_sequences=31	504	31
SDS-V3-plasma-67_Cluster_2595_sequences=31	504	31
SDS-V3-plasma-67_Cluster_6002_sequences=31	504	31
SDS-V3-plasma-67_Cluster_9472_sequences=31	504	31
SDS-V3-plasma-67_Cluster_3511_sequences=31	504	31
SDS-V3-plasma-67_Cluster_13222_sequences=31	504	31
SDS-V3-plasma-67_Cluster_502_sequences=31	504	31
SDS-V3-plasma-67_Cluster_14610_sequences=31	504	31
SDS-V3-plasma-67_Cluster_1404_sequences=31	504	31
SDS-V3-plasma-67_Cluster_14581_sequences=31	504	31
SDS-V3-plasma-67_Cluster_3470_sequences=31	504	31
SDS-V3-plasma-67_Cluster_460_sequences=31	504	31
SDS-V3-plasma-67_Cluster_5163_sequences=31	504	31
SDS-V3-plasma-67_Cluster_3187_sequences=31	504	31
SDS-V3-plasma-67_Cluster_3596_sequences=31	504	31
SDS-V3-plasma-67_Cluster_3918_sequences=31	504	31
SDS-V3-plasma-67_Cluster_2320_sequences=31	504	31
SDS-V3-plasma-67_Cluster_9685_sequences=31	504	31
SDS-V3-plasma-67_Cluster_1618_sequences=31	504	31
SDS-V3-plasma-67_Cluster_6310_sequences=31	504	31
SDS-V3-plasma-67_Cluster_8123_sequences=31	504	31
SDS-V3-plasma-67_Cluster_7418_sequences=31	504	31
SDS-V3-plasma-67_Cluster_1627_sequences=31	504	31
SDS-V3-plasma-67_Cluster_5034_sequences=31	504	31
SDS-V3-plasma-67_Cluster_2806_sequences=31	504	31
SDS-V3-plasma-67_Cluster_5771_sequences=31	504	31
SDS-V3-plasma-67_Cluster_5104_sequences=31	504	31
SDS-V3-plasma-67_Cluster_3505_sequences=31	504	31
SDS-V3-plasma-0_Cluster_2250_sequences=30	0	30
SDS-V3-plasma-0_Cluster_147_sequences=30	0	30
SDS-V3-plasma-0_Cluster_212_sequences=30	0	30
SDS-V3-plasma-0_Cluster_2326_sequences=30	0	30
SDS-V3-plasma-0_Cluster_331_sequences=30	0	30
SDS-V3-plasma-0_Cluster_4097_sequences=30	0	30

SDS-V3-plasma-0_Cluster_4839_sequences=30	0	30
SDS-V3-plasma-0_Cluster_1937_sequences=30	0	30
SDS-V3-plasma-0_Cluster_2294_sequences=30	0	30
SDS-V3-plasma-0_Cluster_1017_sequences=30	0	30
SDS-V3-plasma-0_Cluster_1859_sequences=30	0	30
SDS-V3-plasma-0_Cluster_2878_sequences=30	0	30
SDS-V3-plasma-0_Cluster_3189_sequences=30	0	30
SDS-V3-plasma-0_Cluster_3590_sequences=30	0	30
SDS-V3-plasma-0_Cluster_360_sequences=30	0	30
SDS-V3-plasma-0_Cluster_6491_sequences=30	0	30
SDS-V3-plasma-0_Cluster_9773_sequences=30	0	30
SDS-V3-plasma-0_Cluster_3180_sequences=30	0	30
SDS-V3-plasma-0_Cluster_2144_sequences=30	0	30
SDS-V3-plasma-0_Cluster_2154_sequences=30	0	30
SDS-V3-plasma-0_Cluster_6426_sequences=30	0	30
SDS-V3-plasma-5_Cluster_368_sequences=30	9	30
SDS-V3-plasma-5_Cluster_37_sequences=30	9	30
SDS-V3-plasma-5_Cluster_48_sequences=30	9	30
SDS-V3-plasma-5_Cluster_542_sequences=30	9	30
SDS-V3-plasma-7_Cluster_719_sequences=30	14	30
SDS-V3-plasma-7_Cluster_457_sequences=30	14	30
SDS-V3-plasma-8_Cluster_186_sequences=30	16	30
SDS-V3-plasma-8_Cluster_2674_sequences=30	16	30
SDS-V3-plasma-8_Cluster_2274_sequences=30	16	30
SDS-V3-plasma-8_Cluster_2993_sequences=30	16	30
SDS-V3-plasma-24_Cluster_1894_sequences=30	124	30
SDS-V3-plasma-24_Cluster_329_sequences=30	124	30
SDS-V3-plasma-24_Cluster_1677_sequences=30	124	30
SDS-V3-plasma-24_Cluster_244_sequences=30	124	30
SDS-V3-plasma-24_Cluster_2187_sequences=30	124	30
SDS-V3-plasma-24_Cluster_1289_sequences=30	124	30
SDS-V3-plasma-27_Cluster_1383_sequences=30	131	30
SDS-V3-plasma-27_Cluster_1472_sequences=30	131	30
SDS-V3-plasma-27_Cluster_2290_sequences=30	131	30
SDS-V3-plasma-27_Cluster_988_sequences=30	131	30
SDS-V3-plasma-27_Cluster_629_sequences=30	131	30
SDS-V3-plasma-27_Cluster_227_sequences=30	131	30
SDS-V3-plasma-45_Cluster_1755_sequences=30	282	30
SDS-V3-plasma-45_Cluster_4010_sequences=30	282	30
SDS-V3-plasma-45_Cluster_20038_sequences=30	282	30
SDS-V3-plasma-45_Cluster_1326_sequences=30	282	30
SDS-V3-plasma-45_Cluster_9982_sequences=30	282	30
SDS-V3-plasma-45_Cluster_4214_sequences=30	282	30
SDS-V3-plasma-45_Cluster_11594_sequences=30	282	30
SDS-V3-plasma-45_Cluster_235_sequences=30	282	30

SDS-V3-plasma-45_Cluster_7277_sequences=30	282	30
SDS-V3-plasma-45_Cluster_8866_sequences=30	282	30
SDS-V3-plasma-45_Cluster_13208_sequences=30	282	30
SDS-V3-plasma-45_Cluster_496_sequences=30	282	30
SDS-V3-plasma-45_Cluster_11218_sequences=30	282	30
SDS-V3-plasma-45_Cluster_511_sequences=30	282	30
SDS-V3-plasma-45_Cluster_9810_sequences=30	282	30
SDS-V3-plasma-45_Cluster_8409_sequences=30	282	30
SDS-V3-plasma-45_Cluster_13351_sequences=30	282	30
SDS-V3-plasma-45_Cluster_7460_sequences=30	282	30
SDS-V3-plasma-45_Cluster_6487_sequences=30	282	30
SDS-V3-plasma-45_Cluster_15742_sequences=30	282	30
SDS-V3-plasma-45_Cluster_3633_sequences=30	282	30
SDS-V3-plasma-45_Cluster_7392_sequences=30	282	30
SDS-V3-plasma-45_Cluster_7958_sequences=30	282	30
SDS-V3-plasma-45_Cluster_4586_sequences=30	282	30
SDS-V3-plasma-45_Cluster_4040_sequences=30	282	30
SDS-V3-plasma-45_Cluster_7989_sequences=30	282	30
SDS-V3-plasma-45_Cluster_20170_sequences=30	282	30
SDS-V3-plasma-45_Cluster_8764_sequences=30	282	30
SDS-V3-plasma-45_Cluster_3254_sequences=30	282	30
SDS-V3-plasma-45_Cluster_5380_sequences=30	282	30
SDS-V3-plasma-45_Cluster_2370_sequences=30	282	30
SDS-V3-plasma-45_Cluster_3412_sequences=30	282	30
SDS-V3-plasma-45_Cluster_1961_sequences=30	282	30
SDS-V3-plasma-45_Cluster_6763_sequences=30	282	30
SDS-V3-plasma-45_Cluster_13151_sequences=30	282	30
SDS-V3-plasma-45_Cluster_4268_sequences=30	282	30
SDS-V3-plasma-45_Cluster_11110_sequences=30	282	30
SDS-V3-plasma-45_Cluster_16695_sequences=30	282	30
SDS-V3-plasma-46_Cluster_1089_sequences=30	286	30
SDS-V3-plasma-46_Cluster_3611_sequences=30	286	30
SDS-V3-plasma-46_Cluster_1023_sequences=30	286	30
SDS-V3-plasma-46_Cluster_1898_sequences=30	286	30
SDS-V3-plasma-46_Cluster_2837_sequences=30	286	30
SDS-V3-plasma-46_Cluster_208_sequences=30	286	30
SDS-V3-plasma-46_Cluster_4401_sequences=30	286	30
SDS-V3-plasma-46_Cluster_1960_sequences=30	286	30
SDS-V3-plasma-46_Cluster_2465_sequences=30	286	30
SDS-V3-plasma-46_Cluster_37_sequences=30	286	30
SDS-V3-plasma-46_Cluster_4011_sequences=30	286	30
SDS-V3-plasma-46_Cluster_500_sequences=30	286	30
SDS-V3-plasma-46_Cluster_2591_sequences=30	286	30
SDS-V3-plasma-46_Cluster_350_sequences=30	286	30
SDS-V3-plasma-46_Cluster_404_sequences=30	286	30

SDS-V3-plasma-67_Cluster_3451_sequences=30	504	30
SDS-V3-plasma-67_Cluster_850_sequences=30	504	30
SDS-V3-plasma-67_Cluster_1097_sequences=30	504	30
SDS-V3-plasma-67_Cluster_8252_sequences=30	504	30
SDS-V3-plasma-67_Cluster_393_sequences=30	504	30
SDS-V3-plasma-67_Cluster_4927_sequences=30	504	30
SDS-V3-plasma-67_Cluster_1183_sequences=30	504	30
SDS-V3-plasma-67_Cluster_1386_sequences=30	504	30
SDS-V3-plasma-67_Cluster_1741_sequences=30	504	30
SDS-V3-plasma-67_Cluster_180_sequences=30	504	30
SDS-V3-plasma-67_Cluster_2601_sequences=30	504	30
SDS-V3-plasma-67_Cluster_5551_sequences=30	504	30
SDS-V3-plasma-67_Cluster_2653_sequences=30	504	30
SDS-V3-plasma-67_Cluster_12063_sequences=30	504	30
SDS-V3-plasma-67_Cluster_9416_sequences=30	504	30
SDS-V3-plasma-67_Cluster_2223_sequences=30	504	30
SDS-V3-plasma-67_Cluster_14702_sequences=30	504	30
SDS-V3-plasma-0_Cluster_161_sequences=29	0	29
SDS-V3-plasma-0_Cluster_720_sequences=29	0	29
SDS-V3-plasma-0_Cluster_158_sequences=29	0	29
SDS-V3-plasma-0_Cluster_2333_sequences=29	0	29
SDS-V3-plasma-0_Cluster_2613_sequences=29	0	29
SDS-V3-plasma-0_Cluster_4049_sequences=29	0	29
SDS-V3-plasma-0_Cluster_7995_sequences=29	0	29
SDS-V3-plasma-0_Cluster_2050_sequences=29	0	29
SDS-V3-plasma-0_Cluster_2245_sequences=29	0	29
SDS-V3-plasma-0_Cluster_3485_sequences=29	0	29
SDS-V3-plasma-0_Cluster_1320_sequences=29	0	29
SDS-V3-plasma-0_Cluster_2213_sequences=29	0	29
SDS-V3-plasma-0_Cluster_1307_sequences=29	0	29
SDS-V3-plasma-0_Cluster_1061_sequences=29	0	29
SDS-V3-plasma-0_Cluster_1826_sequences=29	0	29
SDS-V3-plasma-0_Cluster_376_sequences=29	0	29
SDS-V3-plasma-0_Cluster_4860_sequences=29	0	29
SDS-V3-plasma-0_Cluster_1119_sequences=29	0	29
SDS-V3-plasma-0_Cluster_2692_sequences=29	0	29
SDS-V3-plasma-0_Cluster_345_sequences=29	0	29
SDS-V3-plasma-0_Cluster_2842_sequences=29	0	29
SDS-V3-plasma-0_Cluster_1064_sequences=29	0	29
SDS-V3-plasma-0_Cluster_909_sequences=29	0	29
SDS-V3-plasma-5_Cluster_132_sequences=29	9	29
SDS-V3-plasma-5_Cluster_98_sequences=29	9	29
SDS-V3-plasma-7_Cluster_315_sequences=29	14	29
SDS-V3-plasma-7_Cluster_1705_sequences=29	14	29
SDS-V3-plasma-7_Cluster_586_sequences=29	14	29

SDS-V3-plasma-7_Cluster_405_sequences=29	14	29
SDS-V3-plasma-7_Cluster_114_sequences=29	14	29
SDS-V3-plasma-7_Cluster_2054_sequences=29	14	29
SDS-V3-plasma-8_Cluster_133_sequences=29	16	29
SDS-V3-plasma-8_Cluster_3032_sequences=29	16	29
SDS-V3-plasma-8_Cluster_3712_sequences=29	16	29
SDS-V3-plasma-8_Cluster_3053_sequences=29	16	29
SDS-V3-plasma-8_Cluster_2653_sequences=29	16	29
SDS-V3-plasma-8_Cluster_3648_sequences=29	16	29
SDS-V3-plasma-8_Cluster_3000_sequences=29	16	29
SDS-V3-plasma-8_Cluster_2291_sequences=29	16	29
SDS-V3-plasma-24_Cluster_1449_sequences=29	124	29
SDS-V3-plasma-24_Cluster_261_sequences=29	124	29
SDS-V3-plasma-24_Cluster_398_sequences=29	124	29
SDS-V3-plasma-24_Cluster_403_sequences=29	124	29
SDS-V3-plasma-24_Cluster_577_sequences=29	124	29
SDS-V3-plasma-24_Cluster_609_sequences=29	124	29
SDS-V3-plasma-24_Cluster_652_sequences=29	124	29
SDS-V3-plasma-24_Cluster_1227_sequences=29	124	29
SDS-V3-plasma-24_Cluster_719_sequences=29	124	29
SDS-V3-plasma-24_Cluster_1006_sequences=29	124	29
SDS-V3-plasma-24_Cluster_253_sequences=29	124	29
SDS-V3-plasma-27_Cluster_110_sequences=29	131	29
SDS-V3-plasma-27_Cluster_429_sequences=29	131	29
SDS-V3-plasma-27_Cluster_1796_sequences=29	131	29
SDS-V3-plasma-27_Cluster_1401_sequences=29	131	29
SDS-V3-plasma-27_Cluster_794_sequences=29	131	29
SDS-V3-plasma-27_Cluster_1381_sequences=29	131	29
SDS-V3-plasma-27_Cluster_1004_sequences=29	131	29
SDS-V3-plasma-27_Cluster_30_sequences=29	131	29
SDS-V3-plasma-27_Cluster_2376_sequences=29	131	29
SDS-V3-plasma-27_Cluster_1291_sequences=29	131	29
SDS-V3-plasma-45_Cluster_23083_sequences=29	282	29
SDS-V3-plasma-45_Cluster_11789_sequences=29	282	29
SDS-V3-plasma-45_Cluster_2552_sequences=29	282	29
SDS-V3-plasma-45_Cluster_6278_sequences=29	282	29
SDS-V3-plasma-45_Cluster_13909_sequences=29	282	29
SDS-V3-plasma-45_Cluster_10669_sequences=29	282	29
SDS-V3-plasma-45_Cluster_13407_sequences=29	282	29
SDS-V3-plasma-45_Cluster_16938_sequences=29	282	29
SDS-V3-plasma-45_Cluster_18410_sequences=29	282	29
SDS-V3-plasma-45_Cluster_554_sequences=29	282	29
SDS-V3-plasma-45_Cluster_82_sequences=29	282	29
SDS-V3-plasma-45_Cluster_6021_sequences=29	282	29
SDS-V3-plasma-45_Cluster_3658_sequences=29	282	29

SDS-V3-plasma-45_Cluster_13025_sequences=29	282	29
SDS-V3-plasma-45_Cluster_7243_sequences=29	282	29
SDS-V3-plasma-45_Cluster_2223_sequences=29	282	29
SDS-V3-plasma-45_Cluster_2445_sequences=29	282	29
SDS-V3-plasma-45_Cluster_2528_sequences=29	282	29
SDS-V3-plasma-45_Cluster_5762_sequences=29	282	29
SDS-V3-plasma-45_Cluster_9169_sequences=29	282	29
SDS-V3-plasma-45_Cluster_4528_sequences=29	282	29
SDS-V3-plasma-45_Cluster_9136_sequences=29	282	29
SDS-V3-plasma-45_Cluster_13479_sequences=29	282	29
SDS-V3-plasma-45_Cluster_2613_sequences=29	282	29
SDS-V3-plasma-45_Cluster_11686_sequences=29	282	29
SDS-V3-plasma-45_Cluster_6481_sequences=29	282	29
SDS-V3-plasma-45_Cluster_9279_sequences=29	282	29
SDS-V3-plasma-45_Cluster_4226_sequences=29	282	29
SDS-V3-plasma-45_Cluster_4854_sequences=29	282	29
SDS-V3-plasma-45_Cluster_12747_sequences=29	282	29
SDS-V3-plasma-45_Cluster_18297_sequences=29	282	29
SDS-V3-plasma-45_Cluster_242_sequences=29	282	29
SDS-V3-plasma-45_Cluster_4024_sequences=29	282	29
SDS-V3-plasma-45_Cluster_11285_sequences=29	282	29
SDS-V3-plasma-45_Cluster_2428_sequences=29	282	29
SDS-V3-plasma-45_Cluster_3775_sequences=29	282	29
SDS-V3-plasma-45_Cluster_1640_sequences=29	282	29
SDS-V3-plasma-45_Cluster_2995_sequences=29	282	29
SDS-V3-plasma-45_Cluster_9862_sequences=29	282	29
SDS-V3-plasma-45_Cluster_189_sequences=29	282	29
SDS-V3-plasma-46_Cluster_1155_sequences=29	286	29
SDS-V3-plasma-46_Cluster_2626_sequences=29	286	29
SDS-V3-plasma-46_Cluster_4325_sequences=29	286	29
SDS-V3-plasma-46_Cluster_1532_sequences=29	286	29
SDS-V3-plasma-46_Cluster_458_sequences=29	286	29
SDS-V3-plasma-46_Cluster_1404_sequences=29	286	29
SDS-V3-plasma-46_Cluster_1086_sequences=29	286	29
SDS-V3-plasma-46_Cluster_3813_sequences=29	286	29
SDS-V3-plasma-46_Cluster_1171_sequences=29	286	29
SDS-V3-plasma-46_Cluster_3852_sequences=29	286	29
SDS-V3-plasma-46_Cluster_733_sequences=29	286	29
SDS-V3-plasma-46_Cluster_1818_sequences=29	286	29
SDS-V3-plasma-46_Cluster_532_sequences=29	286	29
SDS-V3-plasma-46_Cluster_1085_sequences=29	286	29
SDS-V3-plasma-46_Cluster_316_sequences=29	286	29
SDS-V3-plasma-46_Cluster_806_sequences=29	286	29
SDS-V3-plasma-46_Cluster_3927_sequences=29	286	29
SDS-V3-plasma-46_Cluster_8201_sequences=29	286	29

SDS-V3-plasma-67_Cluster_3035_sequences=29	504	29
SDS-V3-plasma-67_Cluster_5276_sequences=29	504	29
SDS-V3-plasma-67_Cluster_2211_sequences=29	504	29
SDS-V3-plasma-67_Cluster_2644_sequences=29	504	29
SDS-V3-plasma-67_Cluster_6292_sequences=29	504	29
SDS-V3-plasma-67_Cluster_6857_sequences=29	504	29
SDS-V3-plasma-67_Cluster_294_sequences=29	504	29
SDS-V3-plasma-67_Cluster_10643_sequences=29	504	29
SDS-V3-plasma-67_Cluster_1766_sequences=29	504	29
SDS-V3-plasma-67_Cluster_18111_sequences=29	504	29
SDS-V3-plasma-67_Cluster_4008_sequences=29	504	29
SDS-V3-plasma-67_Cluster_2514_sequences=29	504	29
SDS-V3-plasma-67_Cluster_5407_sequences=29	504	29
SDS-V3-plasma-67_Cluster_4244_sequences=29	504	29
SDS-V3-plasma-67_Cluster_2042_sequences=29	504	29
SDS-V3-plasma-67_Cluster_158_sequences=29	504	29
SDS-V3-plasma-67_Cluster_10422_sequences=29	504	29
SDS-V3-plasma-67_Cluster_10064_sequences=29	504	29
SDS-V3-plasma-67_Cluster_6415_sequences=29	504	29
SDS-V3-plasma-67_Cluster_266_sequences=29	504	29
SDS-V3-plasma-0_Cluster_1152_sequences=28	0	28
SDS-V3-plasma-0_Cluster_822_sequences=28	0	28
SDS-V3-plasma-0_Cluster_838_sequences=28	0	28
SDS-V3-plasma-0_Cluster_928_sequences=28	0	28
SDS-V3-plasma-0_Cluster_6003_sequences=28	0	28
SDS-V3-plasma-0_Cluster_2792_sequences=28	0	28
SDS-V3-plasma-0_Cluster_989_sequences=28	0	28
SDS-V3-plasma-0_Cluster_2458_sequences=28	0	28
SDS-V3-plasma-0_Cluster_5335_sequences=28	0	28
SDS-V3-plasma-0_Cluster_1104_sequences=28	0	28
SDS-V3-plasma-0_Cluster_2076_sequences=28	0	28
SDS-V3-plasma-0_Cluster_26_sequences=28	0	28
SDS-V3-plasma-0_Cluster_4458_sequences=28	0	28
SDS-V3-plasma-0_Cluster_4892_sequences=28	0	28
SDS-V3-plasma-0_Cluster_5471_sequences=28	0	28
SDS-V3-plasma-0_Cluster_586_sequences=28	0	28
SDS-V3-plasma-0_Cluster_602_sequences=28	0	28
SDS-V3-plasma-0_Cluster_1100_sequences=28	0	28
SDS-V3-plasma-0_Cluster_273_sequences=28	0	28
SDS-V3-plasma-0_Cluster_4905_sequences=28	0	28
SDS-V3-plasma-0_Cluster_7554_sequences=28	0	28
SDS-V3-plasma-7_Cluster_1677_sequences=28	14	28
SDS-V3-plasma-7_Cluster_383_sequences=28	14	28
SDS-V3-plasma-7_Cluster_933_sequences=28	14	28
SDS-V3-plasma-7_Cluster_863_sequences=28	14	28

SDS-V3-plasma-7_Cluster_1042_sequences=28	14	28
SDS-V3-plasma-7_Cluster_360_sequences=28	14	28
SDS-V3-plasma-8_Cluster_2703_sequences=28	16	28
SDS-V3-plasma-8_Cluster_167_sequences=28	16	28
SDS-V3-plasma-8_Cluster_2826_sequences=28	16	28
SDS-V3-plasma-8_Cluster_4153_sequences=28	16	28
SDS-V3-plasma-8_Cluster_2728_sequences=28	16	28
SDS-V3-plasma-8_Cluster_3228_sequences=28	16	28
SDS-V3-plasma-8_Cluster_2315_sequences=28	16	28
SDS-V3-plasma-8_Cluster_2456_sequences=28	16	28
SDS-V3-plasma-24_Cluster_2151_sequences=28	124	28
SDS-V3-plasma-24_Cluster_326_sequences=28	124	28
SDS-V3-plasma-24_Cluster_399_sequences=28	124	28
SDS-V3-plasma-24_Cluster_4049_sequences=28	124	28
SDS-V3-plasma-24_Cluster_21_sequences=28	124	28
SDS-V3-plasma-24_Cluster_325_sequences=28	124	28
SDS-V3-plasma-24_Cluster_97_sequences=28	124	28
SDS-V3-plasma-24_Cluster_547_sequences=28	124	28
SDS-V3-plasma-24_Cluster_798_sequences=28	124	28
SDS-V3-plasma-24_Cluster_508_sequences=28	124	28
SDS-V3-plasma-27_Cluster_221_sequences=28	131	28
SDS-V3-plasma-27_Cluster_285_sequences=28	131	28
SDS-V3-plasma-27_Cluster_3455_sequences=28	131	28
SDS-V3-plasma-27_Cluster_834_sequences=28	131	28
SDS-V3-plasma-27_Cluster_2366_sequences=28	131	28
SDS-V3-plasma-27_Cluster_2057_sequences=28	131	28
SDS-V3-plasma-27_Cluster_157_sequences=28	131	28
SDS-V3-plasma-27_Cluster_286_sequences=28	131	28
SDS-V3-plasma-27_Cluster_1900_sequences=28	131	28
SDS-V3-plasma-27_Cluster_452_sequences=28	131	28
SDS-V3-plasma-27_Cluster_217_sequences=28	131	28
SDS-V3-plasma-27_Cluster_1050_sequences=28	131	28
SDS-V3-plasma-27_Cluster_1370_sequences=28	131	28
SDS-V3-plasma-27_Cluster_2336_sequences=28	131	28
SDS-V3-plasma-27_Cluster_1642_sequences=28	131	28
SDS-V3-plasma-27_Cluster_755_sequences=28	131	28
SDS-V3-plasma-27_Cluster_2556_sequences=28	131	28
SDS-V3-plasma-27_Cluster_2942_sequences=28	131	28
SDS-V3-plasma-45_Cluster_5643_sequences=28	282	28
SDS-V3-plasma-45_Cluster_2920_sequences=28	282	28
SDS-V3-plasma-45_Cluster_7818_sequences=28	282	28
SDS-V3-plasma-45_Cluster_7188_sequences=28	282	28
SDS-V3-plasma-45_Cluster_5529_sequences=28	282	28
SDS-V3-plasma-45_Cluster_5642_sequences=28	282	28
SDS-V3-plasma-45_Cluster_4585_sequences=28	282	28

SDS-V3-plasma-45_Cluster_6950_sequences=28	282	28
SDS-V3-plasma-45_Cluster_8498_sequences=28	282	28
SDS-V3-plasma-45_Cluster_9972_sequences=28	282	28
SDS-V3-plasma-45_Cluster_1537_sequences=28	282	28
SDS-V3-plasma-45_Cluster_7890_sequences=28	282	28
SDS-V3-plasma-45_Cluster_8817_sequences=28	282	28
SDS-V3-plasma-45_Cluster_10238_sequences=28	282	28
SDS-V3-plasma-45_Cluster_11162_sequences=28	282	28
SDS-V3-plasma-45_Cluster_593_sequences=28	282	28
SDS-V3-plasma-45_Cluster_1251_sequences=28	282	28
SDS-V3-plasma-45_Cluster_137_sequences=28	282	28
SDS-V3-plasma-45_Cluster_8672_sequences=28	282	28
SDS-V3-plasma-45_Cluster_1587_sequences=28	282	28
SDS-V3-plasma-45_Cluster_6650_sequences=28	282	28
SDS-V3-plasma-45_Cluster_5859_sequences=28	282	28
SDS-V3-plasma-45_Cluster_2224_sequences=28	282	28
SDS-V3-plasma-45_Cluster_4599_sequences=28	282	28
SDS-V3-plasma-45_Cluster_8922_sequences=28	282	28
SDS-V3-plasma-45_Cluster_5911_sequences=28	282	28
SDS-V3-plasma-45_Cluster_19887_sequences=28	282	28
SDS-V3-plasma-45_Cluster_18476_sequences=28	282	28
SDS-V3-plasma-45_Cluster_9022_sequences=28	282	28
SDS-V3-plasma-45_Cluster_10486_sequences=28	282	28
SDS-V3-plasma-45_Cluster_2868_sequences=28	282	28
SDS-V3-plasma-45_Cluster_7464_sequences=28	282	28
SDS-V3-plasma-45_Cluster_1390_sequences=28	282	28
SDS-V3-plasma-45_Cluster_1993_sequences=28	282	28
SDS-V3-plasma-45_Cluster_1516_sequences=28	282	28
SDS-V3-plasma-45_Cluster_467_sequences=28	282	28
SDS-V3-plasma-45_Cluster_2603_sequences=28	282	28
SDS-V3-plasma-45_Cluster_1022_sequences=28	282	28
SDS-V3-plasma-45_Cluster_2429_sequences=28	282	28
SDS-V3-plasma-45_Cluster_5512_sequences=28	282	28
SDS-V3-plasma-45_Cluster_2295_sequences=28	282	28
SDS-V3-plasma-45_Cluster_3170_sequences=28	282	28
SDS-V3-plasma-45_Cluster_537_sequences=28	282	28
SDS-V3-plasma-45_Cluster_5863_sequences=28	282	28
SDS-V3-plasma-45_Cluster_12830_sequences=28	282	28
SDS-V3-plasma-45_Cluster_1123_sequences=28	282	28
SDS-V3-plasma-45_Cluster_2791_sequences=28	282	28
SDS-V3-plasma-45_Cluster_5653_sequences=28	282	28
SDS-V3-plasma-45_Cluster_10281_sequences=28	282	28
SDS-V3-plasma-45_Cluster_16555_sequences=28	282	28
SDS-V3-plasma-45_Cluster_2565_sequences=28	282	28
SDS-V3-plasma-45_Cluster_4679_sequences=28	282	28

SDS-V3-plasma-45_Cluster_9441_sequences=28	282	28
SDS-V3-plasma-45_Cluster_6268_sequences=28	282	28
SDS-V3-plasma-45_Cluster_7880_sequences=28	282	28
SDS-V3-plasma-45_Cluster_10362_sequences=28	282	28
SDS-V3-plasma-45_Cluster_5298_sequences=28	282	28
SDS-V3-plasma-45_Cluster_8736_sequences=28	282	28
SDS-V3-plasma-46_Cluster_1255_sequences=28	286	28
SDS-V3-plasma-46_Cluster_3568_sequences=28	286	28
SDS-V3-plasma-46_Cluster_661_sequences=28	286	28
SDS-V3-plasma-46_Cluster_3228_sequences=28	286	28
SDS-V3-plasma-46_Cluster_969_sequences=28	286	28
SDS-V3-plasma-46_Cluster_2796_sequences=28	286	28
SDS-V3-plasma-46_Cluster_1224_sequences=28	286	28
SDS-V3-plasma-46_Cluster_3865_sequences=28	286	28
SDS-V3-plasma-46_Cluster_403_sequences=28	286	28
SDS-V3-plasma-46_Cluster_929_sequences=28	286	28
SDS-V3-plasma-46_Cluster_2875_sequences=28	286	28
SDS-V3-plasma-67_Cluster_13186_sequences=28	504	28
SDS-V3-plasma-67_Cluster_8556_sequences=28	504	28
SDS-V3-plasma-67_Cluster_2631_sequences=28	504	28
SDS-V3-plasma-67_Cluster_14267_sequences=28	504	28
SDS-V3-plasma-67_Cluster_3624_sequences=28	504	28
SDS-V3-plasma-67_Cluster_2380_sequences=28	504	28
SDS-V3-plasma-67_Cluster_6607_sequences=28	504	28
SDS-V3-plasma-67_Cluster_1617_sequences=28	504	28
SDS-V3-plasma-67_Cluster_20040_sequences=28	504	28
SDS-V3-plasma-67_Cluster_27107_sequences=28	504	28
SDS-V3-plasma-67_Cluster_4288_sequences=28	504	28
SDS-V3-plasma-67_Cluster_4752_sequences=28	504	28
SDS-V3-plasma-67_Cluster_9164_sequences=28	504	28
SDS-V3-plasma-67_Cluster_13296_sequences=28	504	28
SDS-V3-plasma-67_Cluster_2790_sequences=28	504	28
SDS-V3-plasma-67_Cluster_1406_sequences=28	504	28
SDS-V3-plasma-67_Cluster_7655_sequences=28	504	28
SDS-V3-plasma-67_Cluster_65_sequences=28	504	28
SDS-V3-plasma-67_Cluster_1194_sequences=28	504	28
SDS-V3-plasma-67_Cluster_8742_sequences=28	504	28
SDS-V3-plasma-67_Cluster_5013_sequences=28	504	28
SDS-V3-plasma-67_Cluster_1253_sequences=28	504	28
SDS-V3-plasma-67_Cluster_35746_sequences=28	504	28
SDS-V3-plasma-0_Cluster_5257_sequences=27	0	27
SDS-V3-plasma-0_Cluster_3202_sequences=27	0	27
SDS-V3-plasma-0_Cluster_1638_sequences=27	0	27
SDS-V3-plasma-0_Cluster_1243_sequences=27	0	27
SDS-V3-plasma-0_Cluster_2919_sequences=27	0	27

SDS-V3-plasma-0_Cluster_3220_sequences=27	0	27
SDS-V3-plasma-0_Cluster_3496_sequences=27	0	27
SDS-V3-plasma-0_Cluster_37_sequences=27	0	27
SDS-V3-plasma-0_Cluster_4378_sequences=27	0	27
SDS-V3-plasma-0_Cluster_4801_sequences=27	0	27
SDS-V3-plasma-0_Cluster_3602_sequences=27	0	27
SDS-V3-plasma-0_Cluster_1044_sequences=27	0	27
SDS-V3-plasma-0_Cluster_3760_sequences=27	0	27
SDS-V3-plasma-0_Cluster_1121_sequences=27	0	27
SDS-V3-plasma-0_Cluster_251_sequences=27	0	27
SDS-V3-plasma-0_Cluster_5743_sequences=27	0	27
SDS-V3-plasma-0_Cluster_1724_sequences=27	0	27
SDS-V3-plasma-0_Cluster_3371_sequences=27	0	27
SDS-V3-plasma-0_Cluster_4166_sequences=27	0	27
SDS-V3-plasma-0_Cluster_4190_sequences=27	0	27
SDS-V3-plasma-0_Cluster_5424_sequences=27	0	27
SDS-V3-plasma-0_Cluster_6368_sequences=27	0	27
SDS-V3-plasma-0_Cluster_2875_sequences=27	0	27
SDS-V3-plasma-0_Cluster_1767_sequences=27	0	27
SDS-V3-plasma-0_Cluster_5504_sequences=27	0	27
SDS-V3-plasma-0_Cluster_491_sequences=27	0	27
SDS-V3-plasma-0_Cluster_10364_sequences=27	0	27
SDS-V3-plasma-0_Cluster_136_sequences=27	0	27
SDS-V3-plasma-0_Cluster_262_sequences=27	0	27
SDS-V3-plasma-0_Cluster_1622_sequences=27	0	27
SDS-V3-plasma-5_Cluster_983_sequences=27	9	27
SDS-V3-plasma-5_Cluster_64_sequences=27	9	27
SDS-V3-plasma-5_Cluster_170_sequences=27	9	27
SDS-V3-plasma-7_Cluster_602_sequences=27	14	27
SDS-V3-plasma-7_Cluster_450_sequences=27	14	27
SDS-V3-plasma-7_Cluster_2013_sequences=27	14	27
SDS-V3-plasma-7_Cluster_407_sequences=27	14	27
SDS-V3-plasma-7_Cluster_387_sequences=27	14	27
SDS-V3-plasma-8_Cluster_4327_sequences=27	16	27
SDS-V3-plasma-8_Cluster_2799_sequences=27	16	27
SDS-V3-plasma-8_Cluster_3234_sequences=27	16	27
SDS-V3-plasma-8_Cluster_3826_sequences=27	16	27
SDS-V3-plasma-8_Cluster_2327_sequences=27	16	27
SDS-V3-plasma-8_Cluster_4308_sequences=27	16	27
SDS-V3-plasma-8_Cluster_2812_sequences=27	16	27
SDS-V3-plasma-8_Cluster_2415_sequences=27	16	27
SDS-V3-plasma-24_Cluster_1003_sequences=27	124	27
SDS-V3-plasma-24_Cluster_112_sequences=27	124	27
SDS-V3-plasma-24_Cluster_1295_sequences=27	124	27
SDS-V3-plasma-24_Cluster_1589_sequences=27	124	27

SDS-V3-plasma-24_Cluster_1675_sequences=27	124	27
SDS-V3-plasma-24_Cluster_203_sequences=27	124	27
SDS-V3-plasma-24_Cluster_2418_sequences=27	124	27
SDS-V3-plasma-24_Cluster_2634_sequences=27	124	27
SDS-V3-plasma-24_Cluster_2939_sequences=27	124	27
SDS-V3-plasma-24_Cluster_404_sequences=27	124	27
SDS-V3-plasma-24_Cluster_453_sequences=27	124	27
SDS-V3-plasma-24_Cluster_620_sequences=27	124	27
SDS-V3-plasma-24_Cluster_9_sequences=27	124	27
SDS-V3-plasma-24_Cluster_1184_sequences=27	124	27
SDS-V3-plasma-24_Cluster_273_sequences=27	124	27
SDS-V3-plasma-24_Cluster_706_sequences=27	124	27
SDS-V3-plasma-24_Cluster_1491_sequences=27	124	27
SDS-V3-plasma-24_Cluster_2534_sequences=27	124	27
SDS-V3-plasma-24_Cluster_2216_sequences=27	124	27
SDS-V3-plasma-27_Cluster_1722_sequences=27	131	27
SDS-V3-plasma-27_Cluster_2271_sequences=27	131	27
SDS-V3-plasma-27_Cluster_2849_sequences=27	131	27
SDS-V3-plasma-27_Cluster_638_sequences=27	131	27
SDS-V3-plasma-27_Cluster_702_sequences=27	131	27
SDS-V3-plasma-27_Cluster_829_sequences=27	131	27
SDS-V3-plasma-27_Cluster_896_sequences=27	131	27
SDS-V3-plasma-27_Cluster_89_sequences=27	131	27
SDS-V3-plasma-27_Cluster_2147_sequences=27	131	27
SDS-V3-plasma-27_Cluster_1721_sequences=27	131	27
SDS-V3-plasma-45_Cluster_7788_sequences=27	282	27
SDS-V3-plasma-45_Cluster_20180_sequences=27	282	27
SDS-V3-plasma-45_Cluster_3913_sequences=27	282	27
SDS-V3-plasma-45_Cluster_13991_sequences=27	282	27
SDS-V3-plasma-45_Cluster_22018_sequences=27	282	27
SDS-V3-plasma-45_Cluster_2217_sequences=27	282	27
SDS-V3-plasma-45_Cluster_2435_sequences=27	282	27
SDS-V3-plasma-45_Cluster_4971_sequences=27	282	27
SDS-V3-plasma-45_Cluster_6772_sequences=27	282	27
SDS-V3-plasma-45_Cluster_8753_sequences=27	282	27
SDS-V3-plasma-45_Cluster_6562_sequences=27	282	27
SDS-V3-plasma-45_Cluster_23706_sequences=27	282	27
SDS-V3-plasma-45_Cluster_116_sequences=27	282	27
SDS-V3-plasma-45_Cluster_2201_sequences=27	282	27
SDS-V3-plasma-45_Cluster_6217_sequences=27	282	27
SDS-V3-plasma-45_Cluster_986_sequences=27	282	27
SDS-V3-plasma-45_Cluster_2427_sequences=27	282	27
SDS-V3-plasma-45_Cluster_2737_sequences=27	282	27
SDS-V3-plasma-45_Cluster_888_sequences=27	282	27
SDS-V3-plasma-45_Cluster_4544_sequences=27	282	27

SDS-V3-plasma-45_Cluster_4491_sequences=27	282	27
SDS-V3-plasma-45_Cluster_4582_sequences=27	282	27
SDS-V3-plasma-45_Cluster_753_sequences=27	282	27
SDS-V3-plasma-45_Cluster_4892_sequences=27	282	27
SDS-V3-plasma-45_Cluster_19525_sequences=27	282	27
SDS-V3-plasma-45_Cluster_5781_sequences=27	282	27
SDS-V3-plasma-45_Cluster_3098_sequences=27	282	27
SDS-V3-plasma-45_Cluster_4052_sequences=27	282	27
SDS-V3-plasma-45_Cluster_5307_sequences=27	282	27
SDS-V3-plasma-45_Cluster_12985_sequences=27	282	27
SDS-V3-plasma-45_Cluster_4576_sequences=27	282	27
SDS-V3-plasma-45_Cluster_3765_sequences=27	282	27
SDS-V3-plasma-45_Cluster_3790_sequences=27	282	27
SDS-V3-plasma-45_Cluster_12126_sequences=27	282	27
SDS-V3-plasma-45_Cluster_11163_sequences=27	282	27
SDS-V3-plasma-45_Cluster_722_sequences=27	282	27
SDS-V3-plasma-45_Cluster_528_sequences=27	282	27
SDS-V3-plasma-45_Cluster_1892_sequences=27	282	27
SDS-V3-plasma-45_Cluster_1885_sequences=27	282	27
SDS-V3-plasma-45_Cluster_14374_sequences=27	282	27
SDS-V3-plasma-45_Cluster_7720_sequences=27	282	27
SDS-V3-plasma-45_Cluster_9149_sequences=27	282	27
SDS-V3-plasma-45_Cluster_2186_sequences=27	282	27
SDS-V3-plasma-45_Cluster_3233_sequences=27	282	27
SDS-V3-plasma-45_Cluster_2283_sequences=27	282	27
SDS-V3-plasma-46_Cluster_765_sequences=27	286	27
SDS-V3-plasma-46_Cluster_759_sequences=27	286	27
SDS-V3-plasma-46_Cluster_2013_sequences=27	286	27
SDS-V3-plasma-46_Cluster_11776_sequences=27	286	27
SDS-V3-plasma-46_Cluster_3495_sequences=27	286	27
SDS-V3-plasma-46_Cluster_29_sequences=27	286	27
SDS-V3-plasma-46_Cluster_4153_sequences=27	286	27
SDS-V3-plasma-46_Cluster_621_sequences=27	286	27
SDS-V3-plasma-46_Cluster_878_sequences=27	286	27
SDS-V3-plasma-46_Cluster_2603_sequences=27	286	27
SDS-V3-plasma-46_Cluster_2470_sequences=27	286	27
SDS-V3-plasma-46_Cluster_3470_sequences=27	286	27
SDS-V3-plasma-46_Cluster_1036_sequences=27	286	27
SDS-V3-plasma-46_Cluster_3059_sequences=27	286	27
SDS-V3-plasma-46_Cluster_3307_sequences=27	286	27
SDS-V3-plasma-67_Cluster_6428_sequences=27	504	27
SDS-V3-plasma-67_Cluster_782_sequences=27	504	27
SDS-V3-plasma-67_Cluster_8166_sequences=27	504	27
SDS-V3-plasma-67_Cluster_13589_sequences=27	504	27
SDS-V3-plasma-67_Cluster_424_sequences=27	504	27

SDS-V3-plasma-67_Cluster_6731_sequences=27	504	27
SDS-V3-plasma-67_Cluster_9232_sequences=27	504	27
SDS-V3-plasma-67_Cluster_2079_sequences=27	504	27
SDS-V3-plasma-67_Cluster_6214_sequences=27	504	27
SDS-V3-plasma-67_Cluster_4779_sequences=27	504	27
SDS-V3-plasma-67_Cluster_6540_sequences=27	504	27
SDS-V3-plasma-67_Cluster_5213_sequences=27	504	27
SDS-V3-plasma-67_Cluster_2067_sequences=27	504	27
SDS-V3-plasma-67_Cluster_18551_sequences=27	504	27
SDS-V3-plasma-67_Cluster_2412_sequences=27	504	27
SDS-V3-plasma-67_Cluster_22912_sequences=27	504	27
SDS-V3-plasma-67_Cluster_5280_sequences=27	504	27
SDS-V3-plasma-67_Cluster_6242_sequences=27	504	27
SDS-V3-plasma-67_Cluster_2242_sequences=27	504	27
SDS-V3-plasma-67_Cluster_3988_sequences=27	504	27
SDS-V3-plasma-67_Cluster_1512_sequences=27	504	27
SDS-V3-plasma-67_Cluster_980_sequences=27	504	27
SDS-V3-plasma-67_Cluster_2193_sequences=27	504	27
SDS-V3-plasma-67_Cluster_8380_sequences=27	504	27
SDS-V3-plasma-0_Cluster_7098_sequences=26	0	26
SDS-V3-plasma-0_Cluster_1038_sequences=26	0	26
SDS-V3-plasma-0_Cluster_2691_sequences=26	0	26
SDS-V3-plasma-0_Cluster_2817_sequences=26	0	26
SDS-V3-plasma-0_Cluster_2940_sequences=26	0	26
SDS-V3-plasma-0_Cluster_3274_sequences=26	0	26
SDS-V3-plasma-0_Cluster_83_sequences=26	0	26
SDS-V3-plasma-0_Cluster_9471_sequences=26	0	26
SDS-V3-plasma-0_Cluster_981_sequences=26	0	26
SDS-V3-plasma-0_Cluster_988_sequences=26	0	26
SDS-V3-plasma-0_Cluster_1325_sequences=26	0	26
SDS-V3-plasma-0_Cluster_220_sequences=26	0	26
SDS-V3-plasma-0_Cluster_3067_sequences=26	0	26
SDS-V3-plasma-0_Cluster_3091_sequences=26	0	26
SDS-V3-plasma-0_Cluster_4271_sequences=26	0	26
SDS-V3-plasma-0_Cluster_6148_sequences=26	0	26
SDS-V3-plasma-0_Cluster_6678_sequences=26	0	26
SDS-V3-plasma-0_Cluster_6545_sequences=26	0	26
SDS-V3-plasma-0_Cluster_3889_sequences=26	0	26
SDS-V3-plasma-0_Cluster_500_sequences=26	0	26
SDS-V3-plasma-0_Cluster_1783_sequences=26	0	26
SDS-V3-plasma-5_Cluster_128_sequences=26	9	26
SDS-V3-plasma-5_Cluster_74_sequences=26	9	26
SDS-V3-plasma-7_Cluster_1240_sequences=26	14	26
SDS-V3-plasma-7_Cluster_684_sequences=26	14	26
SDS-V3-plasma-7_Cluster_1716_sequences=26	14	26

SDS-V3-plasma-8_Cluster_125_sequences=26	16	26
SDS-V3-plasma-8_Cluster_3979_sequences=26	16	26
SDS-V3-plasma-8_Cluster_3011_sequences=26	16	26
SDS-V3-plasma-8_Cluster_3185_sequences=26	16	26
SDS-V3-plasma-8_Cluster_3698_sequences=26	16	26
SDS-V3-plasma-24_Cluster_1009_sequences=26	124	26
SDS-V3-plasma-24_Cluster_1191_sequences=26	124	26
SDS-V3-plasma-24_Cluster_1193_sequences=26	124	26
SDS-V3-plasma-24_Cluster_1721_sequences=26	124	26
SDS-V3-plasma-24_Cluster_2597_sequences=26	124	26
SDS-V3-plasma-24_Cluster_416_sequences=26	124	26
SDS-V3-plasma-24_Cluster_726_sequences=26	124	26
SDS-V3-plasma-24_Cluster_729_sequences=26	124	26
SDS-V3-plasma-24_Cluster_496_sequences=26	124	26
SDS-V3-plasma-24_Cluster_2472_sequences=26	124	26
SDS-V3-plasma-24_Cluster_123_sequences=26	124	26
SDS-V3-plasma-27_Cluster_1532_sequences=26	131	26
SDS-V3-plasma-27_Cluster_1677_sequences=26	131	26
SDS-V3-plasma-27_Cluster_1720_sequences=26	131	26
SDS-V3-plasma-27_Cluster_73_sequences=26	131	26
SDS-V3-plasma-27_Cluster_1692_sequences=26	131	26
SDS-V3-plasma-45_Cluster_2180_sequences=26	282	26
SDS-V3-plasma-45_Cluster_18573_sequences=26	282	26
SDS-V3-plasma-45_Cluster_4340_sequences=26	282	26
SDS-V3-plasma-45_Cluster_3989_sequences=26	282	26
SDS-V3-plasma-45_Cluster_11658_sequences=26	282	26
SDS-V3-plasma-45_Cluster_5588_sequences=26	282	26
SDS-V3-plasma-45_Cluster_644_sequences=26	282	26
SDS-V3-plasma-45_Cluster_3348_sequences=26	282	26
SDS-V3-plasma-45_Cluster_8634_sequences=26	282	26
SDS-V3-plasma-45_Cluster_10889_sequences=26	282	26
SDS-V3-plasma-45_Cluster_6768_sequences=26	282	26
SDS-V3-plasma-45_Cluster_1253_sequences=26	282	26
SDS-V3-plasma-45_Cluster_6073_sequences=26	282	26
SDS-V3-plasma-45_Cluster_6999_sequences=26	282	26
SDS-V3-plasma-45_Cluster_7350_sequences=26	282	26
SDS-V3-plasma-45_Cluster_6509_sequences=26	282	26
SDS-V3-plasma-45_Cluster_13526_sequences=26	282	26
SDS-V3-plasma-45_Cluster_3607_sequences=26	282	26
SDS-V3-plasma-45_Cluster_14956_sequences=26	282	26
SDS-V3-plasma-45_Cluster_9072_sequences=26	282	26
SDS-V3-plasma-45_Cluster_4788_sequences=26	282	26
SDS-V3-plasma-45_Cluster_2832_sequences=26	282	26
SDS-V3-plasma-45_Cluster_3218_sequences=26	282	26
SDS-V3-plasma-45_Cluster_4668_sequences=26	282	26

SDS-V3-plasma-45_Cluster_4823_sequences=26	282	26
SDS-V3-plasma-45_Cluster_6252_sequences=26	282	26
SDS-V3-plasma-45_Cluster_6105_sequences=26	282	26
SDS-V3-plasma-45_Cluster_2229_sequences=26	282	26
SDS-V3-plasma-45_Cluster_4490_sequences=26	282	26
SDS-V3-plasma-45_Cluster_118_sequences=26	282	26
SDS-V3-plasma-45_Cluster_1484_sequences=26	282	26
SDS-V3-plasma-45_Cluster_24442_sequences=26	282	26
SDS-V3-plasma-45_Cluster_15637_sequences=26	282	26
SDS-V3-plasma-45_Cluster_6663_sequences=26	282	26
SDS-V3-plasma-45_Cluster_2242_sequences=26	282	26
SDS-V3-plasma-45_Cluster_6946_sequences=26	282	26
SDS-V3-plasma-45_Cluster_6353_sequences=26	282	26
SDS-V3-plasma-45_Cluster_2134_sequences=26	282	26
SDS-V3-plasma-45_Cluster_477_sequences=26	282	26
SDS-V3-plasma-45_Cluster_38259_sequences=26	282	26
SDS-V3-plasma-45_Cluster_4206_sequences=26	282	26
SDS-V3-plasma-45_Cluster_24858_sequences=26	282	26
SDS-V3-plasma-45_Cluster_6518_sequences=26	282	26
SDS-V3-plasma-45_Cluster_2021_sequences=26	282	26
SDS-V3-plasma-45_Cluster_472_sequences=26	282	26
SDS-V3-plasma-45_Cluster_3717_sequences=26	282	26
SDS-V3-plasma-45_Cluster_744_sequences=26	282	26
SDS-V3-plasma-45_Cluster_2118_sequences=26	282	26
SDS-V3-plasma-45_Cluster_3273_sequences=26	282	26
SDS-V3-plasma-45_Cluster_13346_sequences=26	282	26
SDS-V3-plasma-46_Cluster_1153_sequences=26	286	26
SDS-V3-plasma-46_Cluster_3737_sequences=26	286	26
SDS-V3-plasma-46_Cluster_5094_sequences=26	286	26
SDS-V3-plasma-46_Cluster_1445_sequences=26	286	26
SDS-V3-plasma-46_Cluster_21503_sequences=26	286	26
SDS-V3-plasma-46_Cluster_49_sequences=26	286	26
SDS-V3-plasma-46_Cluster_2921_sequences=26	286	26
SDS-V3-plasma-46_Cluster_6610_sequences=26	286	26
SDS-V3-plasma-46_Cluster_107_sequences=26	286	26
SDS-V3-plasma-46_Cluster_2032_sequences=26	286	26
SDS-V3-plasma-46_Cluster_3288_sequences=26	286	26
SDS-V3-plasma-46_Cluster_741_sequences=26	286	26
SDS-V3-plasma-46_Cluster_9903_sequences=26	286	26
SDS-V3-plasma-46_Cluster_4281_sequences=26	286	26
SDS-V3-plasma-46_Cluster_1700_sequences=26	286	26
SDS-V3-plasma-46_Cluster_5428_sequences=26	286	26
SDS-V3-plasma-46_Cluster_812_sequences=26	286	26
SDS-V3-plasma-46_Cluster_121_sequences=26	286	26
SDS-V3-plasma-46_Cluster_3800_sequences=26	286	26

SDS-V3-plasma-46_Cluster_6820_sequences=26	286	26
SDS-V3-plasma-46_Cluster_5489_sequences=26	286	26
SDS-V3-plasma-46_Cluster_494_sequences=26	286	26
SDS-V3-plasma-46_Cluster_472_sequences=26	286	26
SDS-V3-plasma-46_Cluster_3177_sequences=26	286	26
SDS-V3-plasma-67_Cluster_4610_sequences=26	504	26
SDS-V3-plasma-67_Cluster_13721_sequences=26	504	26
SDS-V3-plasma-67_Cluster_6409_sequences=26	504	26
SDS-V3-plasma-67_Cluster_10882_sequences=26	504	26
SDS-V3-plasma-67_Cluster_560_sequences=26	504	26
SDS-V3-plasma-67_Cluster_3295_sequences=26	504	26
SDS-V3-plasma-67_Cluster_957_sequences=26	504	26
SDS-V3-plasma-67_Cluster_5844_sequences=26	504	26
SDS-V3-plasma-67_Cluster_3895_sequences=26	504	26
SDS-V3-plasma-67_Cluster_2971_sequences=26	504	26
SDS-V3-plasma-67_Cluster_5006_sequences=26	504	26
SDS-V3-plasma-67_Cluster_2579_sequences=26	504	26
SDS-V3-plasma-67_Cluster_10961_sequences=26	504	26
SDS-V3-plasma-67_Cluster_15232_sequences=26	504	26
SDS-V3-plasma-67_Cluster_1761_sequences=26	504	26
SDS-V3-plasma-67_Cluster_22297_sequences=26	504	26
SDS-V3-plasma-67_Cluster_2940_sequences=26	504	26
SDS-V3-plasma-67_Cluster_299_sequences=26	504	26
SDS-V3-plasma-67_Cluster_3954_sequences=26	504	26
SDS-V3-plasma-67_Cluster_5413_sequences=26	504	26
SDS-V3-plasma-67_Cluster_641_sequences=26	504	26
SDS-V3-plasma-67_Cluster_7779_sequences=26	504	26
SDS-V3-plasma-67_Cluster_3944_sequences=26	504	26
SDS-V3-plasma-67_Cluster_4312_sequences=26	504	26
SDS-V3-plasma-67_Cluster_12726_sequences=26	504	26
SDS-V3-plasma-67_Cluster_1914_sequences=26	504	26
SDS-V3-plasma-67_Cluster_9094_sequences=26	504	26
SDS-V3-plasma-67_Cluster_22668_sequences=26	504	26
SDS-V3-plasma-67_Cluster_5323_sequences=26	504	26
SDS-V3-plasma-67_Cluster_9968_sequences=26	504	26
SDS-V3-plasma-67_Cluster_15147_sequences=26	504	26
SDS-V3-plasma-67_Cluster_7289_sequences=26	504	26
SDS-V3-plasma-0_Cluster_363_sequences=25	0	25
SDS-V3-plasma-0_Cluster_3451_sequences=25	0	25
SDS-V3-plasma-0_Cluster_1738_sequences=25	0	25
SDS-V3-plasma-0_Cluster_214_sequences=25	0	25
SDS-V3-plasma-0_Cluster_654_sequences=25	0	25
SDS-V3-plasma-0_Cluster_6625_sequences=25	0	25
SDS-V3-plasma-0_Cluster_560_sequences=25	0	25
SDS-V3-plasma-0_Cluster_404_sequences=25	0	25

SDS-V3-plasma-0_Cluster_2482_sequences=25	0	25
SDS-V3-plasma-0_Cluster_2287_sequences=25	0	25
SDS-V3-plasma-0_Cluster_2337_sequences=25	0	25
SDS-V3-plasma-0_Cluster_1063_sequences=25	0	25
SDS-V3-plasma-0_Cluster_188_sequences=25	0	25
SDS-V3-plasma-0_Cluster_113_sequences=25	0	25
SDS-V3-plasma-0_Cluster_2057_sequences=25	0	25
SDS-V3-plasma-0_Cluster_2270_sequences=25	0	25
SDS-V3-plasma-0_Cluster_233_sequences=25	0	25
SDS-V3-plasma-0_Cluster_3305_sequences=25	0	25
SDS-V3-plasma-0_Cluster_4163_sequences=25	0	25
SDS-V3-plasma-0_Cluster_5609_sequences=25	0	25
SDS-V3-plasma-0_Cluster_1815_sequences=25	0	25
SDS-V3-plasma-0_Cluster_9586_sequences=25	0	25
SDS-V3-plasma-0_Cluster_2136_sequences=25	0	25
SDS-V3-plasma-0_Cluster_3306_sequences=25	0	25
SDS-V3-plasma-5_Cluster_160_sequences=25	9	25
SDS-V3-plasma-5_Cluster_219_sequences=25	9	25
SDS-V3-plasma-5_Cluster_24_sequences=25	9	25
SDS-V3-plasma-7_Cluster_121_sequences=25	14	25
SDS-V3-plasma-7_Cluster_184_sequences=25	14	25
SDS-V3-plasma-7_Cluster_984_sequences=25	14	25
SDS-V3-plasma-7_Cluster_8023_sequences=25	14	25
SDS-V3-plasma-7_Cluster_221_sequences=25	14	25
SDS-V3-plasma-7_Cluster_819_sequences=25	14	25
SDS-V3-plasma-8_Cluster_8_sequences=25	16	25
SDS-V3-plasma-8_Cluster_2679_sequences=25	16	25
SDS-V3-plasma-8_Cluster_2599_sequences=25	16	25
SDS-V3-plasma-8_Cluster_2832_sequences=25	16	25
SDS-V3-plasma-8_Cluster_2884_sequences=25	16	25
SDS-V3-plasma-8_Cluster_2928_sequences=25	16	25
SDS-V3-plasma-8_Cluster_2575_sequences=25	16	25
SDS-V3-plasma-8_Cluster_3024_sequences=25	16	25
SDS-V3-plasma-8_Cluster_3911_sequences=25	16	25
SDS-V3-plasma-24_Cluster_1469_sequences=25	124	25
SDS-V3-plasma-24_Cluster_1492_sequences=25	124	25
SDS-V3-plasma-24_Cluster_1647_sequences=25	124	25
SDS-V3-plasma-24_Cluster_1887_sequences=25	124	25
SDS-V3-plasma-24_Cluster_53_sequences=25	124	25
SDS-V3-plasma-24_Cluster_1412_sequences=25	124	25
SDS-V3-plasma-24_Cluster_484_sequences=25	124	25
SDS-V3-plasma-24_Cluster_991_sequences=25	124	25
SDS-V3-plasma-24_Cluster_41_sequences=25	124	25
SDS-V3-plasma-27_Cluster_115_sequences=25	131	25
SDS-V3-plasma-27_Cluster_159_sequences=25	131	25

SDS-V3-plasma-27_Cluster_1655_sequences=25	131	25
SDS-V3-plasma-27_Cluster_201_sequences=25	131	25
SDS-V3-plasma-27_Cluster_3659_sequences=25	131	25
SDS-V3-plasma-27_Cluster_5349_sequences=25	131	25
SDS-V3-plasma-27_Cluster_821_sequences=25	131	25
SDS-V3-plasma-27_Cluster_983_sequences=25	131	25
SDS-V3-plasma-27_Cluster_3411_sequences=25	131	25
SDS-V3-plasma-27_Cluster_1599_sequences=25	131	25
SDS-V3-plasma-27_Cluster_309_sequences=25	131	25
SDS-V3-plasma-27_Cluster_1435_sequences=25	131	25
SDS-V3-plasma-27_Cluster_689_sequences=25	131	25
SDS-V3-plasma-27_Cluster_523_sequences=25	131	25
SDS-V3-plasma-27_Cluster_3982_sequences=25	131	25
SDS-V3-plasma-27_Cluster_2448_sequences=25	131	25
SDS-V3-plasma-45_Cluster_9428_sequences=25	282	25
SDS-V3-plasma-45_Cluster_15531_sequences=25	282	25
SDS-V3-plasma-45_Cluster_625_sequences=25	282	25
SDS-V3-plasma-45_Cluster_3244_sequences=25	282	25
SDS-V3-plasma-45_Cluster_4285_sequences=25	282	25
SDS-V3-plasma-45_Cluster_8232_sequences=25	282	25
SDS-V3-plasma-45_Cluster_8818_sequences=25	282	25
SDS-V3-plasma-45_Cluster_10097_sequences=25	282	25
SDS-V3-plasma-45_Cluster_1515_sequences=25	282	25
SDS-V3-plasma-45_Cluster_8706_sequences=25	282	25
SDS-V3-plasma-45_Cluster_12524_sequences=25	282	25
SDS-V3-plasma-45_Cluster_14935_sequences=25	282	25
SDS-V3-plasma-45_Cluster_10099_sequences=25	282	25
SDS-V3-plasma-45_Cluster_1231_sequences=25	282	25
SDS-V3-plasma-45_Cluster_12933_sequences=25	282	25
SDS-V3-plasma-45_Cluster_13001_sequences=25	282	25
SDS-V3-plasma-45_Cluster_1941_sequences=25	282	25
SDS-V3-plasma-45_Cluster_3108_sequences=25	282	25
SDS-V3-plasma-45_Cluster_3301_sequences=25	282	25
SDS-V3-plasma-45_Cluster_4136_sequences=25	282	25
SDS-V3-plasma-45_Cluster_4170_sequences=25	282	25
SDS-V3-plasma-45_Cluster_2226_sequences=25	282	25
SDS-V3-plasma-45_Cluster_10978_sequences=25	282	25
SDS-V3-plasma-45_Cluster_3549_sequences=25	282	25
SDS-V3-plasma-45_Cluster_12563_sequences=25	282	25
SDS-V3-plasma-45_Cluster_13640_sequences=25	282	25
SDS-V3-plasma-45_Cluster_6263_sequences=25	282	25
SDS-V3-plasma-45_Cluster_26517_sequences=25	282	25
SDS-V3-plasma-45_Cluster_3403_sequences=25	282	25
SDS-V3-plasma-45_Cluster_10949_sequences=25	282	25
SDS-V3-plasma-45_Cluster_2961_sequences=25	282	25

SDS-V3-plasma-45_Cluster_1831_sequences=25	282	25
SDS-V3-plasma-45_Cluster_2394_sequences=25	282	25
SDS-V3-plasma-45_Cluster_19978_sequences=25	282	25
SDS-V3-plasma-45_Cluster_17343_sequences=25	282	25
SDS-V3-plasma-45_Cluster_2954_sequences=25	282	25
SDS-V3-plasma-45_Cluster_3520_sequences=25	282	25
SDS-V3-plasma-45_Cluster_20012_sequences=25	282	25
SDS-V3-plasma-45_Cluster_645_sequences=25	282	25
SDS-V3-plasma-45_Cluster_3490_sequences=25	282	25
SDS-V3-plasma-45_Cluster_17617_sequences=25	282	25
SDS-V3-plasma-45_Cluster_5463_sequences=25	282	25
SDS-V3-plasma-45_Cluster_5311_sequences=25	282	25
SDS-V3-plasma-45_Cluster_2675_sequences=25	282	25
SDS-V3-plasma-45_Cluster_5916_sequences=25	282	25
SDS-V3-plasma-45_Cluster_2789_sequences=25	282	25
SDS-V3-plasma-45_Cluster_13403_sequences=25	282	25
SDS-V3-plasma-45_Cluster_3832_sequences=25	282	25
SDS-V3-plasma-45_Cluster_3448_sequences=25	282	25
SDS-V3-plasma-45_Cluster_1117_sequences=25	282	25
SDS-V3-plasma-45_Cluster_10960_sequences=25	282	25
SDS-V3-plasma-45_Cluster_1243_sequences=25	282	25
SDS-V3-plasma-45_Cluster_1977_sequences=25	282	25
SDS-V3-plasma-45_Cluster_10545_sequences=25	282	25
SDS-V3-plasma-45_Cluster_25868_sequences=25	282	25
SDS-V3-plasma-45_Cluster_709_sequences=25	282	25
SDS-V3-plasma-45_Cluster_16632_sequences=25	282	25
SDS-V3-plasma-45_Cluster_4753_sequences=25	282	25
SDS-V3-plasma-45_Cluster_4979_sequences=25	282	25
SDS-V3-plasma-45_Cluster_7546_sequences=25	282	25
SDS-V3-plasma-45_Cluster_7651_sequences=25	282	25
SDS-V3-plasma-45_Cluster_12946_sequences=25	282	25
SDS-V3-plasma-45_Cluster_4994_sequences=25	282	25
SDS-V3-plasma-46_Cluster_2645_sequences=25	286	25
SDS-V3-plasma-46_Cluster_3398_sequences=25	286	25
SDS-V3-plasma-46_Cluster_12582_sequences=25	286	25
SDS-V3-plasma-46_Cluster_4781_sequences=25	286	25
SDS-V3-plasma-46_Cluster_1105_sequences=25	286	25
SDS-V3-plasma-46_Cluster_2708_sequences=25	286	25
SDS-V3-plasma-46_Cluster_175_sequences=25	286	25
SDS-V3-plasma-46_Cluster_858_sequences=25	286	25
SDS-V3-plasma-46_Cluster_1711_sequences=25	286	25
SDS-V3-plasma-46_Cluster_2697_sequences=25	286	25
SDS-V3-plasma-46_Cluster_3083_sequences=25	286	25
SDS-V3-plasma-46_Cluster_3520_sequences=25	286	25
SDS-V3-plasma-46_Cluster_4162_sequences=25	286	25

SDS-V3-plasma-46_Cluster_1607_sequences=25	286	25
SDS-V3-plasma-46_Cluster_515_sequences=25	286	25
SDS-V3-plasma-46_Cluster_4245_sequences=25	286	25
SDS-V3-plasma-46_Cluster_1123_sequences=25	286	25
SDS-V3-plasma-46_Cluster_7000_sequences=25	286	25
SDS-V3-plasma-46_Cluster_497_sequences=25	286	25
SDS-V3-plasma-46_Cluster_4560_sequences=25	286	25
SDS-V3-plasma-46_Cluster_14401_sequences=25	286	25
SDS-V3-plasma-46_Cluster_1179_sequences=25	286	25
SDS-V3-plasma-67_Cluster_3140_sequences=25	504	25
SDS-V3-plasma-67_Cluster_1129_sequences=25	504	25
SDS-V3-plasma-67_Cluster_12269_sequences=25	504	25
SDS-V3-plasma-67_Cluster_6895_sequences=25	504	25
SDS-V3-plasma-67_Cluster_2293_sequences=25	504	25
SDS-V3-plasma-67_Cluster_13659_sequences=25	504	25
SDS-V3-plasma-67_Cluster_5340_sequences=25	504	25
SDS-V3-plasma-67_Cluster_3344_sequences=25	504	25
SDS-V3-plasma-67_Cluster_2527_sequences=25	504	25
SDS-V3-plasma-67_Cluster_25711_sequences=25	504	25
SDS-V3-plasma-67_Cluster_8858_sequences=25	504	25
SDS-V3-plasma-67_Cluster_9211_sequences=25	504	25
SDS-V3-plasma-67_Cluster_4319_sequences=25	504	25
SDS-V3-plasma-67_Cluster_9946_sequences=25	504	25
SDS-V3-plasma-67_Cluster_4944_sequences=25	504	25
SDS-V3-plasma-67_Cluster_809_sequences=25	504	25
SDS-V3-plasma-67_Cluster_17206_sequences=25	504	25
SDS-V3-plasma-67_Cluster_1815_sequences=25	504	25
SDS-V3-plasma-67_Cluster_5243_sequences=25	504	25
SDS-V3-plasma-67_Cluster_5815_sequences=25	504	25
SDS-V3-plasma-67_Cluster_58_sequences=25	504	25
SDS-V3-plasma-67_Cluster_6042_sequences=25	504	25
SDS-V3-plasma-67_Cluster_6922_sequences=25	504	25
SDS-V3-plasma-67_Cluster_8801_sequences=25	504	25
SDS-V3-plasma-67_Cluster_9542_sequences=25	504	25
SDS-V3-plasma-67_Cluster_9621_sequences=25	504	25
SDS-V3-plasma-67_Cluster_15776_sequences=25	504	25
SDS-V3-plasma-67_Cluster_3938_sequences=25	504	25
SDS-V3-plasma-67_Cluster_1338_sequences=25	504	25
SDS-V3-plasma-67_Cluster_7488_sequences=25	504	25
SDS-V3-plasma-67_Cluster_6679_sequences=25	504	25
SDS-V3-plasma-67_Cluster_10664_sequences=25	504	25
SDS-V3-plasma-67_Cluster_15987_sequences=25	504	25
SDS-V3-plasma-67_Cluster_5653_sequences=25	504	25
SDS-V3-plasma-67_Cluster_4748_sequences=25	504	25
SDS-V3-plasma-67_Cluster_11321_sequences=25	504	25

SDS-V3-plasma-67_Cluster_12809_sequences=25	504	25
SDS-V3-plasma-67_Cluster_14716_sequences=25	504	25
SDS-V3-plasma-67_Cluster_3671_sequences=25	504	25
SDS-V3-plasma-67_Cluster_3255_sequences=25	504	25
SDS-V3-plasma-0_Cluster_3437_sequences=24	0	24
SDS-V3-plasma-0_Cluster_3462_sequences=24	0	24
SDS-V3-plasma-0_Cluster_1423_sequences=24	0	24
SDS-V3-plasma-0_Cluster_1134_sequences=24	0	24
SDS-V3-plasma-0_Cluster_1436_sequences=24	0	24
SDS-V3-plasma-0_Cluster_1467_sequences=24	0	24
SDS-V3-plasma-0_Cluster_4303_sequences=24	0	24
SDS-V3-plasma-0_Cluster_1611_sequences=24	0	24
SDS-V3-plasma-0_Cluster_10112_sequences=24	0	24
SDS-V3-plasma-0_Cluster_1327_sequences=24	0	24
SDS-V3-plasma-0_Cluster_1482_sequences=24	0	24
SDS-V3-plasma-0_Cluster_169_sequences=24	0	24
SDS-V3-plasma-0_Cluster_872_sequences=24	0	24
SDS-V3-plasma-0_Cluster_969_sequences=24	0	24
SDS-V3-plasma-0_Cluster_6983_sequences=24	0	24
SDS-V3-plasma-0_Cluster_6231_sequences=24	0	24
SDS-V3-plasma-0_Cluster_151_sequences=24	0	24
SDS-V3-plasma-0_Cluster_5214_sequences=24	0	24
SDS-V3-plasma-0_Cluster_3088_sequences=24	0	24
SDS-V3-plasma-0_Cluster_2011_sequences=24	0	24
SDS-V3-plasma-0_Cluster_5232_sequences=24	0	24
SDS-V3-plasma-0_Cluster_5514_sequences=24	0	24
SDS-V3-plasma-0_Cluster_2264_sequences=24	0	24
SDS-V3-plasma-0_Cluster_808_sequences=24	0	24
SDS-V3-plasma-5_Cluster_467_sequences=24	9	24
SDS-V3-plasma-5_Cluster_14_sequences=24	9	24
SDS-V3-plasma-5_Cluster_233_sequences=24	9	24
SDS-V3-plasma-5_Cluster_83_sequences=24	9	24
SDS-V3-plasma-7_Cluster_495_sequences=24	14	24
SDS-V3-plasma-7_Cluster_713_sequences=24	14	24
SDS-V3-plasma-8_Cluster_138_sequences=24	16	24
SDS-V3-plasma-8_Cluster_68_sequences=24	16	24
SDS-V3-plasma-8_Cluster_3133_sequences=24	16	24
SDS-V3-plasma-8_Cluster_3105_sequences=24	16	24
SDS-V3-plasma-8_Cluster_3990_sequences=24	16	24
SDS-V3-plasma-8_Cluster_4075_sequences=24	16	24
SDS-V3-plasma-8_Cluster_3563_sequences=24	16	24
SDS-V3-plasma-8_Cluster_2305_sequences=24	16	24
SDS-V3-plasma-24_Cluster_1634_sequences=24	124	24
SDS-V3-plasma-24_Cluster_1872_sequences=24	124	24
SDS-V3-plasma-24_Cluster_218_sequences=24	124	24

SDS-V3-plasma-24_Cluster_2263_sequences=24	124	24
SDS-V3-plasma-24_Cluster_274_sequences=24	124	24
SDS-V3-plasma-24_Cluster_3595_sequences=24	124	24
SDS-V3-plasma-24_Cluster_367_sequences=24	124	24
SDS-V3-plasma-24_Cluster_1800_sequences=24	124	24
SDS-V3-plasma-24_Cluster_981_sequences=24	124	24
SDS-V3-plasma-24_Cluster_395_sequences=24	124	24
SDS-V3-plasma-24_Cluster_202_sequences=24	124	24
SDS-V3-plasma-24_Cluster_803_sequences=24	124	24
SDS-V3-plasma-24_Cluster_939_sequences=24	124	24
SDS-V3-plasma-27_Cluster_1082_sequences=24	131	24
SDS-V3-plasma-27_Cluster_1115_sequences=24	131	24
SDS-V3-plasma-27_Cluster_16_sequences=24	131	24
SDS-V3-plasma-27_Cluster_2279_sequences=24	131	24
SDS-V3-plasma-27_Cluster_2716_sequences=24	131	24
SDS-V3-plasma-27_Cluster_38_sequences=24	131	24
SDS-V3-plasma-27_Cluster_441_sequences=24	131	24
SDS-V3-plasma-27_Cluster_518_sequences=24	131	24
SDS-V3-plasma-27_Cluster_721_sequences=24	131	24
SDS-V3-plasma-27_Cluster_1820_sequences=24	131	24
SDS-V3-plasma-27_Cluster_3747_sequences=24	131	24
SDS-V3-plasma-27_Cluster_1297_sequences=24	131	24
SDS-V3-plasma-27_Cluster_1849_sequences=24	131	24
SDS-V3-plasma-27_Cluster_2209_sequences=24	131	24
SDS-V3-plasma-27_Cluster_1734_sequences=24	131	24
SDS-V3-plasma-27_Cluster_1443_sequences=24	131	24
SDS-V3-plasma-45_Cluster_6839_sequences=24	282	24
SDS-V3-plasma-45_Cluster_5359_sequences=24	282	24
SDS-V3-plasma-45_Cluster_5786_sequences=24	282	24
SDS-V3-plasma-45_Cluster_10772_sequences=24	282	24
SDS-V3-plasma-45_Cluster_14642_sequences=24	282	24
SDS-V3-plasma-45_Cluster_15884_sequences=24	282	24
SDS-V3-plasma-45_Cluster_3578_sequences=24	282	24
SDS-V3-plasma-45_Cluster_10278_sequences=24	282	24
SDS-V3-plasma-45_Cluster_12230_sequences=24	282	24
SDS-V3-plasma-45_Cluster_145_sequences=24	282	24
SDS-V3-plasma-45_Cluster_7592_sequences=24	282	24
SDS-V3-plasma-45_Cluster_8435_sequences=24	282	24
SDS-V3-plasma-45_Cluster_1102_sequences=24	282	24
SDS-V3-plasma-45_Cluster_1953_sequences=24	282	24
SDS-V3-plasma-45_Cluster_605_sequences=24	282	24
SDS-V3-plasma-45_Cluster_14788_sequences=24	282	24
SDS-V3-plasma-45_Cluster_3100_sequences=24	282	24
SDS-V3-plasma-45_Cluster_6674_sequences=24	282	24
SDS-V3-plasma-45_Cluster_8919_sequences=24	282	24

SDS-V3-plasma-45_Cluster_11123_sequences=24	282	24
SDS-V3-plasma-45_Cluster_12662_sequences=24	282	24
SDS-V3-plasma-45_Cluster_5570_sequences=24	282	24
SDS-V3-plasma-45_Cluster_7746_sequences=24	282	24
SDS-V3-plasma-45_Cluster_27804_sequences=24	282	24
SDS-V3-plasma-45_Cluster_10275_sequences=24	282	24
SDS-V3-plasma-45_Cluster_7642_sequences=24	282	24
SDS-V3-plasma-45_Cluster_3479_sequences=24	282	24
SDS-V3-plasma-45_Cluster_14043_sequences=24	282	24
SDS-V3-plasma-45_Cluster_4881_sequences=24	282	24
SDS-V3-plasma-45_Cluster_1963_sequences=24	282	24
SDS-V3-plasma-45_Cluster_4968_sequences=24	282	24
SDS-V3-plasma-45_Cluster_9423_sequences=24	282	24
SDS-V3-plasma-45_Cluster_12835_sequences=24	282	24
SDS-V3-plasma-45_Cluster_15873_sequences=24	282	24
SDS-V3-plasma-45_Cluster_3752_sequences=24	282	24
SDS-V3-plasma-45_Cluster_13883_sequences=24	282	24
SDS-V3-plasma-45_Cluster_1509_sequences=24	282	24
SDS-V3-plasma-45_Cluster_6092_sequences=24	282	24
SDS-V3-plasma-45_Cluster_22939_sequences=24	282	24
SDS-V3-plasma-45_Cluster_5680_sequences=24	282	24
SDS-V3-plasma-45_Cluster_10180_sequences=24	282	24
SDS-V3-plasma-45_Cluster_31958_sequences=24	282	24
SDS-V3-plasma-45_Cluster_22857_sequences=24	282	24
SDS-V3-plasma-45_Cluster_16399_sequences=24	282	24
SDS-V3-plasma-45_Cluster_16652_sequences=24	282	24
SDS-V3-plasma-45_Cluster_1937_sequences=24	282	24
SDS-V3-plasma-45_Cluster_11296_sequences=24	282	24
SDS-V3-plasma-45_Cluster_1088_sequences=24	282	24
SDS-V3-plasma-45_Cluster_12461_sequences=24	282	24
SDS-V3-plasma-45_Cluster_7408_sequences=24	282	24
SDS-V3-plasma-45_Cluster_7532_sequences=24	282	24
SDS-V3-plasma-45_Cluster_5609_sequences=24	282	24
SDS-V3-plasma-45_Cluster_19030_sequences=24	282	24
SDS-V3-plasma-45_Cluster_8608_sequences=24	282	24
SDS-V3-plasma-45_Cluster_5535_sequences=24	282	24
SDS-V3-plasma-45_Cluster_6436_sequences=24	282	24
SDS-V3-plasma-45_Cluster_4457_sequences=24	282	24
SDS-V3-plasma-45_Cluster_3260_sequences=24	282	24
SDS-V3-plasma-45_Cluster_8795_sequences=24	282	24
SDS-V3-plasma-45_Cluster_33_sequences=24	282	24
SDS-V3-plasma-45_Cluster_9310_sequences=24	282	24
SDS-V3-plasma-45_Cluster_11784_sequences=24	282	24
SDS-V3-plasma-45_Cluster_8688_sequences=24	282	24
SDS-V3-plasma-45_Cluster_62_sequences=24	282	24

SDS-V3-plasma-45_Cluster_16098_sequences=24	282	24
SDS-V3-plasma-45_Cluster_9109_sequences=24	282	24
SDS-V3-plasma-45_Cluster_6778_sequences=24	282	24
SDS-V3-plasma-45_Cluster_14621_sequences=24	282	24
SDS-V3-plasma-45_Cluster_18021_sequences=24	282	24
SDS-V3-plasma-45_Cluster_2156_sequences=24	282	24
SDS-V3-plasma-45_Cluster_7227_sequences=24	282	24
SDS-V3-plasma-45_Cluster_5271_sequences=24	282	24
SDS-V3-plasma-45_Cluster_4945_sequences=24	282	24
SDS-V3-plasma-45_Cluster_11121_sequences=24	282	24
SDS-V3-plasma-45_Cluster_5471_sequences=24	282	24
SDS-V3-plasma-45_Cluster_9347_sequences=24	282	24
SDS-V3-plasma-45_Cluster_18107_sequences=24	282	24
SDS-V3-plasma-45_Cluster_3764_sequences=24	282	24
SDS-V3-plasma-46_Cluster_3009_sequences=24	286	24
SDS-V3-plasma-46_Cluster_8150_sequences=24	286	24
SDS-V3-plasma-46_Cluster_379_sequences=24	286	24
SDS-V3-plasma-46_Cluster_1270_sequences=24	286	24
SDS-V3-plasma-46_Cluster_303_sequences=24	286	24
SDS-V3-plasma-46_Cluster_2464_sequences=24	286	24
SDS-V3-plasma-46_Cluster_1112_sequences=24	286	24
SDS-V3-plasma-46_Cluster_2957_sequences=24	286	24
SDS-V3-plasma-46_Cluster_1494_sequences=24	286	24
SDS-V3-plasma-46_Cluster_8782_sequences=24	286	24
SDS-V3-plasma-46_Cluster_814_sequences=24	286	24
SDS-V3-plasma-46_Cluster_1551_sequences=24	286	24
SDS-V3-plasma-46_Cluster_1730_sequences=24	286	24
SDS-V3-plasma-46_Cluster_7136_sequences=24	286	24
SDS-V3-plasma-46_Cluster_9122_sequences=24	286	24
SDS-V3-plasma-46_Cluster_4730_sequences=24	286	24
SDS-V3-plasma-46_Cluster_5377_sequences=24	286	24
SDS-V3-plasma-46_Cluster_3000_sequences=24	286	24
SDS-V3-plasma-46_Cluster_1649_sequences=24	286	24
SDS-V3-plasma-46_Cluster_1610_sequences=24	286	24
SDS-V3-plasma-46_Cluster_12986_sequences=24	286	24
SDS-V3-plasma-46_Cluster_1402_sequences=24	286	24
SDS-V3-plasma-67_Cluster_24132_sequences=24	504	24
SDS-V3-plasma-67_Cluster_100402_sequences=24	504	24
SDS-V3-plasma-67_Cluster_25479_sequences=24	504	24
SDS-V3-plasma-67_Cluster_3991_sequences=24	504	24
SDS-V3-plasma-67_Cluster_10669_sequences=24	504	24
SDS-V3-plasma-67_Cluster_8537_sequences=24	504	24
SDS-V3-plasma-67_Cluster_20968_sequences=24	504	24
SDS-V3-plasma-67_Cluster_8052_sequences=24	504	24
SDS-V3-plasma-67_Cluster_2916_sequences=24	504	24

SDS-V3-plasma-67_Cluster_1190_sequences=24	504	24
SDS-V3-plasma-67_Cluster_5744_sequences=24	504	24
SDS-V3-plasma-67_Cluster_4446_sequences=24	504	24
SDS-V3-plasma-67_Cluster_1437_sequences=24	504	24
SDS-V3-plasma-67_Cluster_15853_sequences=24	504	24
SDS-V3-plasma-67_Cluster_17478_sequences=24	504	24
SDS-V3-plasma-67_Cluster_2932_sequences=24	504	24
SDS-V3-plasma-67_Cluster_37762_sequences=24	504	24
SDS-V3-plasma-67_Cluster_4360_sequences=24	504	24
SDS-V3-plasma-67_Cluster_8489_sequences=24	504	24
SDS-V3-plasma-67_Cluster_14824_sequences=24	504	24
SDS-V3-plasma-67_Cluster_24848_sequences=24	504	24
SDS-V3-plasma-67_Cluster_20678_sequences=24	504	24
SDS-V3-plasma-67_Cluster_932_sequences=24	504	24
SDS-V3-plasma-67_Cluster_2439_sequences=24	504	24
SDS-V3-plasma-67_Cluster_1739_sequences=24	504	24
SDS-V3-plasma-67_Cluster_14711_sequences=24	504	24
SDS-V3-plasma-67_Cluster_6838_sequences=24	504	24
SDS-V3-plasma-67_Cluster_2369_sequences=24	504	24
SDS-V3-plasma-67_Cluster_701_sequences=24	504	24
SDS-V3-plasma-67_Cluster_6259_sequences=24	504	24
SDS-V3-plasma-0_Cluster_191_sequences=23	0	23
SDS-V3-plasma-0_Cluster_1399_sequences=23	0	23
SDS-V3-plasma-0_Cluster_1770_sequences=23	0	23
SDS-V3-plasma-0_Cluster_186_sequences=23	0	23
SDS-V3-plasma-0_Cluster_242_sequences=23	0	23
SDS-V3-plasma-0_Cluster_2478_sequences=23	0	23
SDS-V3-plasma-0_Cluster_2865_sequences=23	0	23
SDS-V3-plasma-0_Cluster_3632_sequences=23	0	23
SDS-V3-plasma-0_Cluster_39_sequences=23	0	23
SDS-V3-plasma-0_Cluster_4987_sequences=23	0	23
SDS-V3-plasma-0_Cluster_820_sequences=23	0	23
SDS-V3-plasma-0_Cluster_6442_sequences=23	0	23
SDS-V3-plasma-0_Cluster_3240_sequences=23	0	23
SDS-V3-plasma-0_Cluster_3048_sequences=23	0	23
SDS-V3-plasma-0_Cluster_2591_sequences=23	0	23
SDS-V3-plasma-0_Cluster_1186_sequences=23	0	23
SDS-V3-plasma-0_Cluster_26457_sequences=23	0	23
SDS-V3-plasma-0_Cluster_1366_sequences=23	0	23
SDS-V3-plasma-0_Cluster_14833_sequences=23	0	23
SDS-V3-plasma-0_Cluster_2489_sequences=23	0	23
SDS-V3-plasma-0_Cluster_4833_sequences=23	0	23
SDS-V3-plasma-0_Cluster_1359_sequences=23	0	23
SDS-V3-plasma-0_Cluster_1852_sequences=23	0	23
SDS-V3-plasma-0_Cluster_2035_sequences=23	0	23

SDS-V3-plasma-0_Cluster_362_sequences=23	0	23
SDS-V3-plasma-0_Cluster_3690_sequences=23	0	23
SDS-V3-plasma-0_Cluster_480_sequences=23	0	23
SDS-V3-plasma-0_Cluster_751_sequences=23	0	23
SDS-V3-plasma-0_Cluster_139_sequences=23	0	23
SDS-V3-plasma-0_Cluster_367_sequences=23	0	23
SDS-V3-plasma-0_Cluster_786_sequences=23	0	23
SDS-V3-plasma-0_Cluster_1920_sequences=23	0	23
SDS-V3-plasma-0_Cluster_6975_sequences=23	0	23
SDS-V3-plasma-0_Cluster_8386_sequences=23	0	23
SDS-V3-plasma-0_Cluster_1960_sequences=23	0	23
SDS-V3-plasma-0_Cluster_910_sequences=23	0	23
SDS-V3-plasma-0_Cluster_7111_sequences=23	0	23
SDS-V3-plasma-0_Cluster_764_sequences=23	0	23
SDS-V3-plasma-5_Cluster_272_sequences=23	9	23
SDS-V3-plasma-5_Cluster_375_sequences=23	9	23
SDS-V3-plasma-5_Cluster_47_sequences=23	9	23
SDS-V3-plasma-5_Cluster_55_sequences=23	9	23
SDS-V3-plasma-5_Cluster_80_sequences=23	9	23
SDS-V3-plasma-7_Cluster_784_sequences=23	14	23
SDS-V3-plasma-7_Cluster_945_sequences=23	14	23
SDS-V3-plasma-7_Cluster_718_sequences=23	14	23
SDS-V3-plasma-7_Cluster_2644_sequences=23	14	23
SDS-V3-plasma-7_Cluster_320_sequences=23	14	23
SDS-V3-plasma-7_Cluster_1328_sequences=23	14	23
SDS-V3-plasma-7_Cluster_2615_sequences=23	14	23
SDS-V3-plasma-7_Cluster_916_sequences=23	14	23
SDS-V3-plasma-7_Cluster_832_sequences=23	14	23
SDS-V3-plasma-8_Cluster_4007_sequences=23	16	23
SDS-V3-plasma-8_Cluster_2598_sequences=23	16	23
SDS-V3-plasma-8_Cluster_3211_sequences=23	16	23
SDS-V3-plasma-8_Cluster_4538_sequences=23	16	23
SDS-V3-plasma-8_Cluster_2975_sequences=23	16	23
SDS-V3-plasma-8_Cluster_3430_sequences=23	16	23
SDS-V3-plasma-8_Cluster_8921_sequences=23	16	23
SDS-V3-plasma-8_Cluster_2862_sequences=23	16	23
SDS-V3-plasma-8_Cluster_2666_sequences=23	16	23
SDS-V3-plasma-24_Cluster_346_sequences=23	124	23
SDS-V3-plasma-24_Cluster_488_sequences=23	124	23
SDS-V3-plasma-24_Cluster_612_sequences=23	124	23
SDS-V3-plasma-24_Cluster_748_sequences=23	124	23
SDS-V3-plasma-24_Cluster_818_sequences=23	124	23
SDS-V3-plasma-24_Cluster_1319_sequences=23	124	23
SDS-V3-plasma-24_Cluster_226_sequences=23	124	23
SDS-V3-plasma-24_Cluster_3568_sequences=23	124	23

SDS-V3-plasma-24_Cluster_1395_sequences=23	124	23
SDS-V3-plasma-24_Cluster_865_sequences=23	124	23
SDS-V3-plasma-24_Cluster_943_sequences=23	124	23
SDS-V3-plasma-24_Cluster_100_sequences=23	124	23
SDS-V3-plasma-24_Cluster_1413_sequences=23	124	23
SDS-V3-plasma-24_Cluster_734_sequences=23	124	23
SDS-V3-plasma-27_Cluster_1467_sequences=23	131	23
SDS-V3-plasma-27_Cluster_1548_sequences=23	131	23
SDS-V3-plasma-27_Cluster_1807_sequences=23	131	23
SDS-V3-plasma-27_Cluster_3101_sequences=23	131	23
SDS-V3-plasma-27_Cluster_3_sequences=23	131	23
SDS-V3-plasma-27_Cluster_412_sequences=23	131	23
SDS-V3-plasma-27_Cluster_626_sequences=23	131	23
SDS-V3-plasma-27_Cluster_742_sequences=23	131	23
SDS-V3-plasma-27_Cluster_768_sequences=23	131	23
SDS-V3-plasma-27_Cluster_958_sequences=23	131	23
SDS-V3-plasma-27_Cluster_676_sequences=23	131	23
SDS-V3-plasma-27_Cluster_773_sequences=23	131	23
SDS-V3-plasma-27_Cluster_1501_sequences=23	131	23
SDS-V3-plasma-27_Cluster_85_sequences=23	131	23
SDS-V3-plasma-27_Cluster_1983_sequences=23	131	23
SDS-V3-plasma-45_Cluster_10411_sequences=23	282	23
SDS-V3-plasma-45_Cluster_13409_sequences=23	282	23
SDS-V3-plasma-45_Cluster_3962_sequences=23	282	23
SDS-V3-plasma-45_Cluster_97_sequences=23	282	23
SDS-V3-plasma-45_Cluster_1956_sequences=23	282	23
SDS-V3-plasma-45_Cluster_3061_sequences=23	282	23
SDS-V3-plasma-45_Cluster_3732_sequences=23	282	23
SDS-V3-plasma-45_Cluster_4763_sequences=23	282	23
SDS-V3-plasma-45_Cluster_7082_sequences=23	282	23
SDS-V3-plasma-45_Cluster_13737_sequences=23	282	23
SDS-V3-plasma-45_Cluster_1585_sequences=23	282	23
SDS-V3-plasma-45_Cluster_3346_sequences=23	282	23
SDS-V3-plasma-45_Cluster_6351_sequences=23	282	23
SDS-V3-plasma-45_Cluster_6512_sequences=23	282	23
SDS-V3-plasma-45_Cluster_18324_sequences=23	282	23
SDS-V3-plasma-45_Cluster_12548_sequences=23	282	23
SDS-V3-plasma-45_Cluster_9549_sequences=23	282	23
SDS-V3-plasma-45_Cluster_1844_sequences=23	282	23
SDS-V3-plasma-45_Cluster_3415_sequences=23	282	23
SDS-V3-plasma-45_Cluster_3601_sequences=23	282	23
SDS-V3-plasma-45_Cluster_9238_sequences=23	282	23
SDS-V3-plasma-45_Cluster_11061_sequences=23	282	23
SDS-V3-plasma-45_Cluster_2080_sequences=23	282	23
SDS-V3-plasma-45_Cluster_4037_sequences=23	282	23

SDS-V3-plasma-45_Cluster_5276_sequences=23	282	23
SDS-V3-plasma-45_Cluster_5290_sequences=23	282	23
SDS-V3-plasma-45_Cluster_8524_sequences=23	282	23
SDS-V3-plasma-45_Cluster_3984_sequences=23	282	23
SDS-V3-plasma-45_Cluster_5637_sequences=23	282	23
SDS-V3-plasma-45_Cluster_21669_sequences=23	282	23
SDS-V3-plasma-45_Cluster_13828_sequences=23	282	23
SDS-V3-plasma-45_Cluster_2935_sequences=23	282	23
SDS-V3-plasma-45_Cluster_4875_sequences=23	282	23
SDS-V3-plasma-45_Cluster_16903_sequences=23	282	23
SDS-V3-plasma-45_Cluster_9519_sequences=23	282	23
SDS-V3-plasma-45_Cluster_8101_sequences=23	282	23
SDS-V3-plasma-45_Cluster_14288_sequences=23	282	23
SDS-V3-plasma-45_Cluster_8047_sequences=23	282	23
SDS-V3-plasma-45_Cluster_6157_sequences=23	282	23
SDS-V3-plasma-45_Cluster_750_sequences=23	282	23
SDS-V3-plasma-45_Cluster_8981_sequences=23	282	23
SDS-V3-plasma-45_Cluster_538_sequences=23	282	23
SDS-V3-plasma-45_Cluster_1025_sequences=23	282	23
SDS-V3-plasma-45_Cluster_16090_sequences=23	282	23
SDS-V3-plasma-45_Cluster_961_sequences=23	282	23
SDS-V3-plasma-45_Cluster_2628_sequences=23	282	23
SDS-V3-plasma-45_Cluster_13798_sequences=23	282	23
SDS-V3-plasma-45_Cluster_10126_sequences=23	282	23
SDS-V3-plasma-45_Cluster_5384_sequences=23	282	23
SDS-V3-plasma-45_Cluster_568_sequences=23	282	23
SDS-V3-plasma-45_Cluster_321_sequences=23	282	23
SDS-V3-plasma-45_Cluster_4693_sequences=23	282	23
SDS-V3-plasma-45_Cluster_9879_sequences=23	282	23
SDS-V3-plasma-45_Cluster_20477_sequences=23	282	23
SDS-V3-plasma-45_Cluster_12667_sequences=23	282	23
SDS-V3-plasma-45_Cluster_14757_sequences=23	282	23
SDS-V3-plasma-45_Cluster_9015_sequences=23	282	23
SDS-V3-plasma-45_Cluster_3488_sequences=23	282	23
SDS-V3-plasma-45_Cluster_1926_sequences=23	282	23
SDS-V3-plasma-46_Cluster_1307_sequences=23	286	23
SDS-V3-plasma-46_Cluster_1850_sequences=23	286	23
SDS-V3-plasma-46_Cluster_195_sequences=23	286	23
SDS-V3-plasma-46_Cluster_1486_sequences=23	286	23
SDS-V3-plasma-46_Cluster_2514_sequences=23	286	23
SDS-V3-plasma-46_Cluster_3247_sequences=23	286	23
SDS-V3-plasma-46_Cluster_622_sequences=23	286	23
SDS-V3-plasma-46_Cluster_8339_sequences=23	286	23
SDS-V3-plasma-46_Cluster_3359_sequences=23	286	23
SDS-V3-plasma-46_Cluster_1077_sequences=23	286	23

SDS-V3-plasma-46_Cluster_206_sequences=23	286	23
SDS-V3-plasma-46_Cluster_4775_sequences=23	286	23
SDS-V3-plasma-46_Cluster_31_sequences=23	286	23
SDS-V3-plasma-46_Cluster_3950_sequences=23	286	23
SDS-V3-plasma-46_Cluster_1720_sequences=23	286	23
SDS-V3-plasma-46_Cluster_1739_sequences=23	286	23
SDS-V3-plasma-46_Cluster_374_sequences=23	286	23
SDS-V3-plasma-46_Cluster_5689_sequences=23	286	23
SDS-V3-plasma-46_Cluster_137_sequences=23	286	23
SDS-V3-plasma-46_Cluster_1301_sequences=23	286	23
SDS-V3-plasma-46_Cluster_23067_sequences=23	286	23
SDS-V3-plasma-46_Cluster_3256_sequences=23	286	23
SDS-V3-plasma-46_Cluster_4160_sequences=23	286	23
SDS-V3-plasma-46_Cluster_3607_sequences=23	286	23
SDS-V3-plasma-46_Cluster_2566_sequences=23	286	23
SDS-V3-plasma-46_Cluster_21947_sequences=23	286	23
SDS-V3-plasma-67_Cluster_3047_sequences=23	504	23
SDS-V3-plasma-67_Cluster_14792_sequences=23	504	23
SDS-V3-plasma-67_Cluster_3923_sequences=23	504	23
SDS-V3-plasma-67_Cluster_10658_sequences=23	504	23
SDS-V3-plasma-67_Cluster_12524_sequences=23	504	23
SDS-V3-plasma-67_Cluster_17063_sequences=23	504	23
SDS-V3-plasma-67_Cluster_7729_sequences=23	504	23
SDS-V3-plasma-67_Cluster_17326_sequences=23	504	23
SDS-V3-plasma-67_Cluster_20787_sequences=23	504	23
SDS-V3-plasma-67_Cluster_3605_sequences=23	504	23
SDS-V3-plasma-67_Cluster_20752_sequences=23	504	23
SDS-V3-plasma-67_Cluster_13118_sequences=23	504	23
SDS-V3-plasma-67_Cluster_1044_sequences=23	504	23
SDS-V3-plasma-67_Cluster_13090_sequences=23	504	23
SDS-V3-plasma-67_Cluster_1579_sequences=23	504	23
SDS-V3-plasma-67_Cluster_1697_sequences=23	504	23
SDS-V3-plasma-67_Cluster_4010_sequences=23	504	23
SDS-V3-plasma-67_Cluster_5114_sequences=23	504	23
SDS-V3-plasma-67_Cluster_7605_sequences=23	504	23
SDS-V3-plasma-67_Cluster_9127_sequences=23	504	23
SDS-V3-plasma-67_Cluster_1606_sequences=23	504	23
SDS-V3-plasma-67_Cluster_1116_sequences=23	504	23
SDS-V3-plasma-67_Cluster_4707_sequences=23	504	23
SDS-V3-plasma-67_Cluster_10243_sequences=23	504	23
SDS-V3-plasma-67_Cluster_18688_sequences=23	504	23
SDS-V3-plasma-67_Cluster_3341_sequences=23	504	23
SDS-V3-plasma-67_Cluster_1891_sequences=23	504	23
SDS-V3-plasma-67_Cluster_8711_sequences=23	504	23
SDS-V3-plasma-0_Cluster_478_sequences=22	0	22

SDS-V3-plasma-0_Cluster_13027_sequences=22	0	22
SDS-V3-plasma-0_Cluster_395_sequences=22	0	22
SDS-V3-plasma-0_Cluster_7857_sequences=22	0	22
SDS-V3-plasma-0_Cluster_3801_sequences=22	0	22
SDS-V3-plasma-0_Cluster_2239_sequences=22	0	22
SDS-V3-plasma-0_Cluster_201_sequences=22	0	22
SDS-V3-plasma-0_Cluster_2202_sequences=22	0	22
SDS-V3-plasma-0_Cluster_3339_sequences=22	0	22
SDS-V3-plasma-0_Cluster_2615_sequences=22	0	22
SDS-V3-plasma-0_Cluster_457_sequences=22	0	22
SDS-V3-plasma-0_Cluster_583_sequences=22	0	22
SDS-V3-plasma-0_Cluster_1005_sequences=22	0	22
SDS-V3-plasma-0_Cluster_1214_sequences=22	0	22
SDS-V3-plasma-0_Cluster_2211_sequences=22	0	22
SDS-V3-plasma-0_Cluster_2820_sequences=22	0	22
SDS-V3-plasma-0_Cluster_4714_sequences=22	0	22
SDS-V3-plasma-0_Cluster_5561_sequences=22	0	22
SDS-V3-plasma-0_Cluster_766_sequences=22	0	22
SDS-V3-plasma-0_Cluster_246_sequences=22	0	22
SDS-V3-plasma-0_Cluster_5300_sequences=22	0	22
SDS-V3-plasma-0_Cluster_1809_sequences=22	0	22
SDS-V3-plasma-0_Cluster_3699_sequences=22	0	22
SDS-V3-plasma-0_Cluster_1778_sequences=22	0	22
SDS-V3-plasma-0_Cluster_840_sequences=22	0	22
SDS-V3-plasma-0_Cluster_10160_sequences=22	0	22
SDS-V3-plasma-0_Cluster_3218_sequences=22	0	22
SDS-V3-plasma-0_Cluster_693_sequences=22	0	22
SDS-V3-plasma-0_Cluster_1178_sequences=22	0	22
SDS-V3-plasma-0_Cluster_2414_sequences=22	0	22
SDS-V3-plasma-0_Cluster_3100_sequences=22	0	22
SDS-V3-plasma-5_Cluster_287_sequences=22	9	22
SDS-V3-plasma-7_Cluster_1000_sequences=22	14	22
SDS-V3-plasma-7_Cluster_2301_sequences=22	14	22
SDS-V3-plasma-7_Cluster_491_sequences=22	14	22
SDS-V3-plasma-7_Cluster_2766_sequences=22	14	22
SDS-V3-plasma-7_Cluster_197_sequences=22	14	22
SDS-V3-plasma-7_Cluster_731_sequences=22	14	22
SDS-V3-plasma-7_Cluster_673_sequences=22	14	22
SDS-V3-plasma-7_Cluster_3767_sequences=22	14	22
SDS-V3-plasma-7_Cluster_483_sequences=22	14	22
SDS-V3-plasma-7_Cluster_3000_sequences=22	14	22
SDS-V3-plasma-7_Cluster_670_sequences=22	14	22
SDS-V3-plasma-8_Cluster_265_sequences=22	16	22
SDS-V3-plasma-8_Cluster_4889_sequences=22	16	22
SDS-V3-plasma-8_Cluster_3003_sequences=22	16	22

SDS-V3-plasma-8_Cluster_6159_sequences=22	16	22
SDS-V3-plasma-8_Cluster_2767_sequences=22	16	22
SDS-V3-plasma-8_Cluster_2605_sequences=22	16	22
SDS-V3-plasma-24_Cluster_1119_sequences=22	124	22
SDS-V3-plasma-24_Cluster_2094_sequences=22	124	22
SDS-V3-plasma-24_Cluster_2409_sequences=22	124	22
SDS-V3-plasma-24_Cluster_906_sequences=22	124	22
SDS-V3-plasma-24_Cluster_987_sequences=22	124	22
SDS-V3-plasma-24_Cluster_1093_sequences=22	124	22
SDS-V3-plasma-24_Cluster_1374_sequences=22	124	22
SDS-V3-plasma-24_Cluster_1212_sequences=22	124	22
SDS-V3-plasma-24_Cluster_1027_sequences=22	124	22
SDS-V3-plasma-24_Cluster_2254_sequences=22	124	22
SDS-V3-plasma-24_Cluster_1889_sequences=22	124	22
SDS-V3-plasma-24_Cluster_8814_sequences=22	124	22
SDS-V3-plasma-27_Cluster_1106_sequences=22	131	22
SDS-V3-plasma-27_Cluster_1111_sequences=22	131	22
SDS-V3-plasma-27_Cluster_446_sequences=22	131	22
SDS-V3-plasma-27_Cluster_448_sequences=22	131	22
SDS-V3-plasma-27_Cluster_503_sequences=22	131	22
SDS-V3-plasma-27_Cluster_723_sequences=22	131	22
SDS-V3-plasma-27_Cluster_854_sequences=22	131	22
SDS-V3-plasma-27_Cluster_945_sequences=22	131	22
SDS-V3-plasma-27_Cluster_3219_sequences=22	131	22
SDS-V3-plasma-27_Cluster_447_sequences=22	131	22
SDS-V3-plasma-27_Cluster_247_sequences=22	131	22
SDS-V3-plasma-27_Cluster_203_sequences=22	131	22
SDS-V3-plasma-27_Cluster_1335_sequences=22	131	22
SDS-V3-plasma-27_Cluster_3181_sequences=22	131	22
SDS-V3-plasma-27_Cluster_711_sequences=22	131	22
SDS-V3-plasma-27_Cluster_2296_sequences=22	131	22
SDS-V3-plasma-27_Cluster_1052_sequences=22	131	22
SDS-V3-plasma-45_Cluster_7610_sequences=22	282	22
SDS-V3-plasma-45_Cluster_3539_sequences=22	282	22
SDS-V3-plasma-45_Cluster_4713_sequences=22	282	22
SDS-V3-plasma-45_Cluster_16502_sequences=22	282	22
SDS-V3-plasma-45_Cluster_4836_sequences=22	282	22
SDS-V3-plasma-45_Cluster_2014_sequences=22	282	22
SDS-V3-plasma-45_Cluster_2517_sequences=22	282	22
SDS-V3-plasma-45_Cluster_3413_sequences=22	282	22
SDS-V3-plasma-45_Cluster_4227_sequences=22	282	22
SDS-V3-plasma-45_Cluster_5963_sequences=22	282	22
SDS-V3-plasma-45_Cluster_7970_sequences=22	282	22
SDS-V3-plasma-45_Cluster_8444_sequences=22	282	22
SDS-V3-plasma-45_Cluster_6297_sequences=22	282	22

SDS-V3-plasma-45_Cluster_7185_sequences=22	282	22
SDS-V3-plasma-45_Cluster_3138_sequences=22	282	22
SDS-V3-plasma-45_Cluster_4201_sequences=22	282	22
SDS-V3-plasma-45_Cluster_15682_sequences=22	282	22
SDS-V3-plasma-45_Cluster_7160_sequences=22	282	22
SDS-V3-plasma-45_Cluster_7624_sequences=22	282	22
SDS-V3-plasma-45_Cluster_16022_sequences=22	282	22
SDS-V3-plasma-45_Cluster_17186_sequences=22	282	22
SDS-V3-plasma-45_Cluster_112_sequences=22	282	22
SDS-V3-plasma-45_Cluster_15541_sequences=22	282	22
SDS-V3-plasma-45_Cluster_14758_sequences=22	282	22
SDS-V3-plasma-45_Cluster_14860_sequences=22	282	22
SDS-V3-plasma-45_Cluster_5325_sequences=22	282	22
SDS-V3-plasma-45_Cluster_5634_sequences=22	282	22
SDS-V3-plasma-45_Cluster_10152_sequences=22	282	22
SDS-V3-plasma-45_Cluster_13379_sequences=22	282	22
SDS-V3-plasma-45_Cluster_9600_sequences=22	282	22
SDS-V3-plasma-45_Cluster_14950_sequences=22	282	22
SDS-V3-plasma-45_Cluster_3366_sequences=22	282	22
SDS-V3-plasma-45_Cluster_1805_sequences=22	282	22
SDS-V3-plasma-45_Cluster_703_sequences=22	282	22
SDS-V3-plasma-45_Cluster_23876_sequences=22	282	22
SDS-V3-plasma-45_Cluster_2448_sequences=22	282	22
SDS-V3-plasma-45_Cluster_14908_sequences=22	282	22
SDS-V3-plasma-45_Cluster_6322_sequences=22	282	22
SDS-V3-plasma-45_Cluster_4670_sequences=22	282	22
SDS-V3-plasma-45_Cluster_4324_sequences=22	282	22
SDS-V3-plasma-45_Cluster_9267_sequences=22	282	22
SDS-V3-plasma-45_Cluster_8393_sequences=22	282	22
SDS-V3-plasma-45_Cluster_11584_sequences=22	282	22
SDS-V3-plasma-45_Cluster_8529_sequences=22	282	22
SDS-V3-plasma-45_Cluster_6887_sequences=22	282	22
SDS-V3-plasma-45_Cluster_299_sequences=22	282	22
SDS-V3-plasma-45_Cluster_13350_sequences=22	282	22
SDS-V3-plasma-45_Cluster_5138_sequences=22	282	22
SDS-V3-plasma-45_Cluster_13555_sequences=22	282	22
SDS-V3-plasma-45_Cluster_727_sequences=22	282	22
SDS-V3-plasma-45_Cluster_7162_sequences=22	282	22
SDS-V3-plasma-45_Cluster_3866_sequences=22	282	22
SDS-V3-plasma-45_Cluster_791_sequences=22	282	22
SDS-V3-plasma-45_Cluster_2929_sequences=22	282	22
SDS-V3-plasma-45_Cluster_2926_sequences=22	282	22
SDS-V3-plasma-45_Cluster_15026_sequences=22	282	22
SDS-V3-plasma-45_Cluster_246_sequences=22	282	22
SDS-V3-plasma-45_Cluster_10993_sequences=22	282	22

SDS-V3-plasma-45_Cluster_3190_sequences=22	282	22
SDS-V3-plasma-45_Cluster_12661_sequences=22	282	22
SDS-V3-plasma-45_Cluster_14619_sequences=22	282	22
SDS-V3-plasma-45_Cluster_9538_sequences=22	282	22
SDS-V3-plasma-45_Cluster_32415_sequences=22	282	22
SDS-V3-plasma-45_Cluster_6859_sequences=22	282	22
SDS-V3-plasma-45_Cluster_4682_sequences=22	282	22
SDS-V3-plasma-45_Cluster_13750_sequences=22	282	22
SDS-V3-plasma-45_Cluster_19731_sequences=22	282	22
SDS-V3-plasma-45_Cluster_4156_sequences=22	282	22
SDS-V3-plasma-45_Cluster_5672_sequences=22	282	22
SDS-V3-plasma-46_Cluster_2051_sequences=22	286	22
SDS-V3-plasma-46_Cluster_8474_sequences=22	286	22
SDS-V3-plasma-46_Cluster_4198_sequences=22	286	22
SDS-V3-plasma-46_Cluster_2495_sequences=22	286	22
SDS-V3-plasma-46_Cluster_8542_sequences=22	286	22
SDS-V3-plasma-46_Cluster_7574_sequences=22	286	22
SDS-V3-plasma-46_Cluster_895_sequences=22	286	22
SDS-V3-plasma-46_Cluster_2590_sequences=22	286	22
SDS-V3-plasma-46_Cluster_3426_sequences=22	286	22
SDS-V3-plasma-46_Cluster_2442_sequences=22	286	22
SDS-V3-plasma-46_Cluster_7569_sequences=22	286	22
SDS-V3-plasma-46_Cluster_4863_sequences=22	286	22
SDS-V3-plasma-46_Cluster_2090_sequences=22	286	22
SDS-V3-plasma-46_Cluster_6039_sequences=22	286	22
SDS-V3-plasma-46_Cluster_6123_sequences=22	286	22
SDS-V3-plasma-46_Cluster_210_sequences=22	286	22
SDS-V3-plasma-46_Cluster_5681_sequences=22	286	22
SDS-V3-plasma-46_Cluster_6991_sequences=22	286	22
SDS-V3-plasma-46_Cluster_993_sequences=22	286	22
SDS-V3-plasma-46_Cluster_6221_sequences=22	286	22
SDS-V3-plasma-46_Cluster_4102_sequences=22	286	22
SDS-V3-plasma-46_Cluster_1618_sequences=22	286	22
SDS-V3-plasma-46_Cluster_211_sequences=22	286	22
SDS-V3-plasma-46_Cluster_6120_sequences=22	286	22
SDS-V3-plasma-46_Cluster_110_sequences=22	286	22
SDS-V3-plasma-46_Cluster_392_sequences=22	286	22
SDS-V3-plasma-46_Cluster_4903_sequences=22	286	22
SDS-V3-plasma-46_Cluster_231_sequences=22	286	22
SDS-V3-plasma-46_Cluster_4002_sequences=22	286	22
SDS-V3-plasma-67_Cluster_993_sequences=22	504	22
SDS-V3-plasma-67_Cluster_7830_sequences=22	504	22
SDS-V3-plasma-67_Cluster_3198_sequences=22	504	22
SDS-V3-plasma-67_Cluster_5245_sequences=22	504	22
SDS-V3-plasma-67_Cluster_27228_sequences=22	504	22

SDS-V3-plasma-67_Cluster_14378_sequences=22	504	22
SDS-V3-plasma-67_Cluster_5176_sequences=22	504	22
SDS-V3-plasma-67_Cluster_15090_sequences=22	504	22
SDS-V3-plasma-67_Cluster_1113_sequences=22	504	22
SDS-V3-plasma-67_Cluster_7092_sequences=22	504	22
SDS-V3-plasma-67_Cluster_906_sequences=22	504	22
SDS-V3-plasma-67_Cluster_5683_sequences=22	504	22
SDS-V3-plasma-67_Cluster_6393_sequences=22	504	22
SDS-V3-plasma-67_Cluster_1551_sequences=22	504	22
SDS-V3-plasma-67_Cluster_1567_sequences=22	504	22
SDS-V3-plasma-67_Cluster_19179_sequences=22	504	22
SDS-V3-plasma-67_Cluster_2639_sequences=22	504	22
SDS-V3-plasma-67_Cluster_3646_sequences=22	504	22
SDS-V3-plasma-67_Cluster_4093_sequences=22	504	22
SDS-V3-plasma-67_Cluster_4725_sequences=22	504	22
SDS-V3-plasma-67_Cluster_5319_sequences=22	504	22
SDS-V3-plasma-67_Cluster_6293_sequences=22	504	22
SDS-V3-plasma-67_Cluster_5313_sequences=22	504	22
SDS-V3-plasma-67_Cluster_5665_sequences=22	504	22
SDS-V3-plasma-67_Cluster_12146_sequences=22	504	22
SDS-V3-plasma-67_Cluster_6647_sequences=22	504	22
SDS-V3-plasma-67_Cluster_4874_sequences=22	504	22
SDS-V3-plasma-67_Cluster_14643_sequences=22	504	22
SDS-V3-plasma-67_Cluster_6220_sequences=22	504	22
SDS-V3-plasma-67_Cluster_1843_sequences=22	504	22
SDS-V3-plasma-67_Cluster_1849_sequences=22	504	22
SDS-V3-plasma-67_Cluster_10596_sequences=22	504	22
SDS-V3-plasma-67_Cluster_51147_sequences=22	504	22
SDS-V3-PBMC-45_Cluster_26030_sequences=21	282	21
SDS-V3-plasma-0_Cluster_1846_sequences=21	0	21
SDS-V3-plasma-0_Cluster_1732_sequences=21	0	21
SDS-V3-plasma-0_Cluster_1814_sequences=21	0	21
SDS-V3-plasma-0_Cluster_2719_sequences=21	0	21
SDS-V3-plasma-0_Cluster_3285_sequences=21	0	21
SDS-V3-plasma-0_Cluster_4644_sequences=21	0	21
SDS-V3-plasma-0_Cluster_4646_sequences=21	0	21
SDS-V3-plasma-0_Cluster_6254_sequences=21	0	21
SDS-V3-plasma-0_Cluster_4680_sequences=21	0	21
SDS-V3-plasma-0_Cluster_10953_sequences=21	0	21
SDS-V3-plasma-0_Cluster_462_sequences=21	0	21
SDS-V3-plasma-0_Cluster_2327_sequences=21	0	21
SDS-V3-plasma-0_Cluster_3278_sequences=21	0	21
SDS-V3-plasma-0_Cluster_5897_sequences=21	0	21
SDS-V3-plasma-0_Cluster_2760_sequences=21	0	21
SDS-V3-plasma-0_Cluster_127_sequences=21	0	21

SDS-V3-plasma-0_Cluster_2074_sequences=21	0	21
SDS-V3-plasma-0_Cluster_2196_sequences=21	0	21
SDS-V3-plasma-0_Cluster_3406_sequences=21	0	21
SDS-V3-plasma-0_Cluster_4868_sequences=21	0	21
SDS-V3-plasma-0_Cluster_768_sequences=21	0	21
SDS-V3-plasma-0_Cluster_498_sequences=21	0	21
SDS-V3-plasma-0_Cluster_75_sequences=21	0	21
SDS-V3-plasma-0_Cluster_727_sequences=21	0	21
SDS-V3-plasma-0_Cluster_7037_sequences=21	0	21
SDS-V3-plasma-0_Cluster_53_sequences=21	0	21
SDS-V3-plasma-0_Cluster_1653_sequences=21	0	21
SDS-V3-plasma-0_Cluster_1445_sequences=21	0	21
SDS-V3-plasma-0_Cluster_1567_sequences=21	0	21
SDS-V3-plasma-5_Cluster_819_sequences=21	9	21
SDS-V3-plasma-7_Cluster_549_sequences=21	14	21
SDS-V3-plasma-7_Cluster_366_sequences=21	14	21
SDS-V3-plasma-7_Cluster_459_sequences=21	14	21
SDS-V3-plasma-7_Cluster_498_sequences=21	14	21
SDS-V3-plasma-7_Cluster_218_sequences=21	14	21
SDS-V3-plasma-7_Cluster_2687_sequences=21	14	21
SDS-V3-plasma-7_Cluster_3903_sequences=21	14	21
SDS-V3-plasma-8_Cluster_273_sequences=21	16	21
SDS-V3-plasma-8_Cluster_2452_sequences=21	16	21
SDS-V3-plasma-8_Cluster_2506_sequences=21	16	21
SDS-V3-plasma-8_Cluster_3536_sequences=21	16	21
SDS-V3-plasma-8_Cluster_3163_sequences=21	16	21
SDS-V3-plasma-8_Cluster_2986_sequences=21	16	21
SDS-V3-plasma-8_Cluster_2665_sequences=21	16	21
SDS-V3-plasma-24_Cluster_1120_sequences=21	124	21
SDS-V3-plasma-24_Cluster_1856_sequences=21	124	21
SDS-V3-plasma-24_Cluster_201_sequences=21	124	21
SDS-V3-plasma-24_Cluster_2119_sequences=21	124	21
SDS-V3-plasma-24_Cluster_2870_sequences=21	124	21
SDS-V3-plasma-24_Cluster_650_sequences=21	124	21
SDS-V3-plasma-24_Cluster_764_sequences=21	124	21
SDS-V3-plasma-24_Cluster_976_sequences=21	124	21
SDS-V3-plasma-24_Cluster_982_sequences=21	124	21
SDS-V3-plasma-24_Cluster_781_sequences=21	124	21
SDS-V3-plasma-24_Cluster_1578_sequences=21	124	21
SDS-V3-plasma-24_Cluster_2331_sequences=21	124	21
SDS-V3-plasma-24_Cluster_3122_sequences=21	124	21
SDS-V3-plasma-24_Cluster_1335_sequences=21	124	21
SDS-V3-plasma-24_Cluster_2436_sequences=21	124	21
SDS-V3-plasma-24_Cluster_1087_sequences=21	124	21
SDS-V3-plasma-27_Cluster_1563_sequences=21	131	21

SDS-V3-plasma-27_Cluster_17_sequences=21	131	21
SDS-V3-plasma-27_Cluster_2305_sequences=21	131	21
SDS-V3-plasma-27_Cluster_293_sequences=21	131	21
SDS-V3-plasma-27_Cluster_3896_sequences=21	131	21
SDS-V3-plasma-27_Cluster_403_sequences=21	131	21
SDS-V3-plasma-27_Cluster_410_sequences=21	131	21
SDS-V3-plasma-27_Cluster_660_sequences=21	131	21
SDS-V3-plasma-27_Cluster_779_sequences=21	131	21
SDS-V3-plasma-27_Cluster_811_sequences=21	131	21
SDS-V3-plasma-27_Cluster_1499_sequences=21	131	21
SDS-V3-plasma-27_Cluster_918_sequences=21	131	21
SDS-V3-plasma-27_Cluster_481_sequences=21	131	21
SDS-V3-plasma-27_Cluster_188_sequences=21	131	21
SDS-V3-plasma-27_Cluster_3231_sequences=21	131	21
SDS-V3-plasma-27_Cluster_2007_sequences=21	131	21
SDS-V3-plasma-27_Cluster_40_sequences=21	131	21
SDS-V3-plasma-27_Cluster_1379_sequences=21	131	21
SDS-V3-plasma-27_Cluster_1311_sequences=21	131	21
SDS-V3-plasma-27_Cluster_491_sequences=21	131	21
SDS-V3-plasma-27_Cluster_313_sequences=21	131	21
SDS-V3-plasma-27_Cluster_1175_sequences=21	131	21
SDS-V3-plasma-45_Cluster_11143_sequences=21	282	21
SDS-V3-plasma-45_Cluster_27265_sequences=21	282	21
SDS-V3-plasma-45_Cluster_4935_sequences=21	282	21
SDS-V3-plasma-45_Cluster_18526_sequences=21	282	21
SDS-V3-plasma-45_Cluster_7962_sequences=21	282	21
SDS-V3-plasma-45_Cluster_5204_sequences=21	282	21
SDS-V3-plasma-45_Cluster_5354_sequences=21	282	21
SDS-V3-plasma-45_Cluster_5114_sequences=21	282	21
SDS-V3-plasma-45_Cluster_12346_sequences=21	282	21
SDS-V3-plasma-45_Cluster_13102_sequences=21	282	21
SDS-V3-plasma-45_Cluster_19437_sequences=21	282	21
SDS-V3-plasma-45_Cluster_12032_sequences=21	282	21
SDS-V3-plasma-45_Cluster_1388_sequences=21	282	21
SDS-V3-plasma-45_Cluster_5362_sequences=21	282	21
SDS-V3-plasma-45_Cluster_4173_sequences=21	282	21
SDS-V3-plasma-45_Cluster_14831_sequences=21	282	21
SDS-V3-plasma-45_Cluster_15352_sequences=21	282	21
SDS-V3-plasma-45_Cluster_12284_sequences=21	282	21
SDS-V3-plasma-45_Cluster_14261_sequences=21	282	21
SDS-V3-plasma-45_Cluster_2390_sequences=21	282	21
SDS-V3-plasma-45_Cluster_10503_sequences=21	282	21
SDS-V3-plasma-45_Cluster_13662_sequences=21	282	21
SDS-V3-plasma-45_Cluster_24666_sequences=21	282	21
SDS-V3-plasma-45_Cluster_130_sequences=21	282	21

SDS-V3-plasma-45_Cluster_7851_sequences=21	282	21
SDS-V3-plasma-45_Cluster_24097_sequences=21	282	21
SDS-V3-plasma-45_Cluster_8408_sequences=21	282	21
SDS-V3-plasma-45_Cluster_5376_sequences=21	282	21
SDS-V3-plasma-45_Cluster_4780_sequences=21	282	21
SDS-V3-plasma-45_Cluster_2263_sequences=21	282	21
SDS-V3-plasma-45_Cluster_3602_sequences=21	282	21
SDS-V3-plasma-45_Cluster_43957_sequences=21	282	21
SDS-V3-plasma-45_Cluster_6519_sequences=21	282	21
SDS-V3-plasma-45_Cluster_3339_sequences=21	282	21
SDS-V3-plasma-45_Cluster_8256_sequences=21	282	21
SDS-V3-plasma-45_Cluster_4560_sequences=21	282	21
SDS-V3-plasma-45_Cluster_7809_sequences=21	282	21
SDS-V3-plasma-45_Cluster_969_sequences=21	282	21
SDS-V3-plasma-45_Cluster_3022_sequences=21	282	21
SDS-V3-plasma-45_Cluster_146_sequences=21	282	21
SDS-V3-plasma-45_Cluster_12718_sequences=21	282	21
SDS-V3-plasma-45_Cluster_10774_sequences=21	282	21
SDS-V3-plasma-45_Cluster_12501_sequences=21	282	21
SDS-V3-plasma-45_Cluster_23844_sequences=21	282	21
SDS-V3-plasma-45_Cluster_18900_sequences=21	282	21
SDS-V3-plasma-45_Cluster_23716_sequences=21	282	21
SDS-V3-plasma-45_Cluster_6528_sequences=21	282	21
SDS-V3-plasma-45_Cluster_4450_sequences=21	282	21
SDS-V3-plasma-45_Cluster_20042_sequences=21	282	21
SDS-V3-plasma-45_Cluster_6102_sequences=21	282	21
SDS-V3-plasma-45_Cluster_11276_sequences=21	282	21
SDS-V3-plasma-45_Cluster_7868_sequences=21	282	21
SDS-V3-plasma-45_Cluster_12107_sequences=21	282	21
SDS-V3-plasma-45_Cluster_22073_sequences=21	282	21
SDS-V3-plasma-45_Cluster_7850_sequences=21	282	21
SDS-V3-plasma-45_Cluster_4675_sequences=21	282	21
SDS-V3-plasma-45_Cluster_4562_sequences=21	282	21
SDS-V3-plasma-45_Cluster_15626_sequences=21	282	21
SDS-V3-plasma-45_Cluster_1275_sequences=21	282	21
SDS-V3-plasma-45_Cluster_132_sequences=21	282	21
SDS-V3-plasma-45_Cluster_1984_sequences=21	282	21
SDS-V3-plasma-45_Cluster_19519_sequences=21	282	21
SDS-V3-plasma-45_Cluster_3768_sequences=21	282	21
SDS-V3-plasma-45_Cluster_20415_sequences=21	282	21
SDS-V3-plasma-45_Cluster_9277_sequences=21	282	21
SDS-V3-plasma-45_Cluster_9073_sequences=21	282	21
SDS-V3-plasma-45_Cluster_15319_sequences=21	282	21
SDS-V3-plasma-45_Cluster_92_sequences=21	282	21
SDS-V3-plasma-45_Cluster_1647_sequences=21	282	21

SDS-V3-plasma-45_Cluster_11129_sequences=21	282	21
SDS-V3-plasma-45_Cluster_170_sequences=21	282	21
SDS-V3-plasma-45_Cluster_2591_sequences=21	282	21
SDS-V3-plasma-45_Cluster_11611_sequences=21	282	21
SDS-V3-plasma-45_Cluster_2194_sequences=21	282	21
SDS-V3-plasma-45_Cluster_7463_sequences=21	282	21
SDS-V3-plasma-45_Cluster_20709_sequences=21	282	21
SDS-V3-plasma-45_Cluster_8649_sequences=21	282	21
SDS-V3-plasma-45_Cluster_19059_sequences=21	282	21
SDS-V3-plasma-45_Cluster_4332_sequences=21	282	21
SDS-V3-plasma-45_Cluster_66656_sequences=21	282	21
SDS-V3-plasma-46_Cluster_15189_sequences=21	286	21
SDS-V3-plasma-46_Cluster_6526_sequences=21	286	21
SDS-V3-plasma-46_Cluster_3080_sequences=21	286	21
SDS-V3-plasma-46_Cluster_6434_sequences=21	286	21
SDS-V3-plasma-46_Cluster_1476_sequences=21	286	21
SDS-V3-plasma-46_Cluster_251_sequences=21	286	21
SDS-V3-plasma-46_Cluster_5612_sequences=21	286	21
SDS-V3-plasma-46_Cluster_3137_sequences=21	286	21
SDS-V3-plasma-46_Cluster_4887_sequences=21	286	21
SDS-V3-plasma-46_Cluster_4374_sequences=21	286	21
SDS-V3-plasma-46_Cluster_983_sequences=21	286	21
SDS-V3-plasma-46_Cluster_2084_sequences=21	286	21
SDS-V3-plasma-46_Cluster_2195_sequences=21	286	21
SDS-V3-plasma-46_Cluster_15231_sequences=21	286	21
SDS-V3-plasma-46_Cluster_1187_sequences=21	286	21
SDS-V3-plasma-46_Cluster_1207_sequences=21	286	21
SDS-V3-plasma-46_Cluster_1458_sequences=21	286	21
SDS-V3-plasma-46_Cluster_3274_sequences=21	286	21
SDS-V3-plasma-46_Cluster_610_sequences=21	286	21
SDS-V3-plasma-46_Cluster_1459_sequences=21	286	21
SDS-V3-plasma-46_Cluster_1130_sequences=21	286	21
SDS-V3-plasma-46_Cluster_2876_sequences=21	286	21
SDS-V3-plasma-46_Cluster_3999_sequences=21	286	21
SDS-V3-plasma-46_Cluster_1929_sequences=21	286	21
SDS-V3-plasma-46_Cluster_2156_sequences=21	286	21
SDS-V3-plasma-46_Cluster_3455_sequences=21	286	21
SDS-V3-plasma-46_Cluster_21913_sequences=21	286	21
SDS-V3-plasma-46_Cluster_5348_sequences=21	286	21
SDS-V3-plasma-46_Cluster_4708_sequences=21	286	21
SDS-V3-plasma-46_Cluster_1562_sequences=21	286	21
SDS-V3-plasma-67_Cluster_9607_sequences=21	504	21
SDS-V3-plasma-67_Cluster_18514_sequences=21	504	21
SDS-V3-plasma-67_Cluster_13852_sequences=21	504	21
SDS-V3-plasma-67_Cluster_666_sequences=21	504	21

SDS-V3-plasma-67_Cluster_1895_sequences=21	504	21
SDS-V3-plasma-67_Cluster_22614_sequences=21	504	21
SDS-V3-plasma-67_Cluster_6753_sequences=21	504	21
SDS-V3-plasma-67_Cluster_15287_sequences=21	504	21
SDS-V3-plasma-67_Cluster_7481_sequences=21	504	21
SDS-V3-plasma-67_Cluster_6656_sequences=21	504	21
SDS-V3-plasma-67_Cluster_7055_sequences=21	504	21
SDS-V3-plasma-67_Cluster_1432_sequences=21	504	21
SDS-V3-plasma-67_Cluster_12510_sequences=21	504	21
SDS-V3-plasma-67_Cluster_14574_sequences=21	504	21
SDS-V3-plasma-67_Cluster_14025_sequences=21	504	21
SDS-V3-plasma-67_Cluster_14037_sequences=21	504	21
SDS-V3-plasma-67_Cluster_14842_sequences=21	504	21
SDS-V3-plasma-67_Cluster_1636_sequences=21	504	21
SDS-V3-plasma-67_Cluster_24861_sequences=21	504	21
SDS-V3-plasma-67_Cluster_2826_sequences=21	504	21
SDS-V3-plasma-67_Cluster_3465_sequences=21	504	21
SDS-V3-plasma-67_Cluster_4665_sequences=21	504	21
SDS-V3-plasma-67_Cluster_2630_sequences=21	504	21
SDS-V3-plasma-67_Cluster_7115_sequences=21	504	21
SDS-V3-plasma-67_Cluster_10224_sequences=21	504	21
SDS-V3-plasma-67_Cluster_11544_sequences=21	504	21
SDS-V3-plasma-67_Cluster_867_sequences=21	504	21
SDS-V3-plasma-67_Cluster_26039_sequences=21	504	21
SDS-V3-plasma-67_Cluster_10582_sequences=21	504	21
SDS-V3-plasma-67_Cluster_23110_sequences=21	504	21
SDS-V3-plasma-67_Cluster_11145_sequences=21	504	21
SDS-V3-plasma-67_Cluster_21243_sequences=21	504	21
SDS-V3-plasma-67_Cluster_2366_sequences=21	504	21
SDS-V3-plasma-67_Cluster_9330_sequences=21	504	21
SDS-V3-plasma-67_Cluster_9839_sequences=21	504	21
SDS-V3-plasma-67_Cluster_6052_sequences=21	504	21
SDS-V3-plasma-67_Cluster_3314_sequences=21	504	21
SDS-V3-plasma-67_Cluster_5524_sequences=21	504	21
SDS-V3-plasma-67_Cluster_7958_sequences=21	504	21
SDS-V3-plasma-67_Cluster_76354_sequences=21	504	21
SDS-V3-plasma-67_Cluster_76635_sequences=21	504	21
SDS-V3-plasma-67_Cluster_8326_sequences=21	504	21
SDS-V3-plasma-67_Cluster_17720_sequences=21	504	21
SDS-V3-plasma-67_Cluster_2101_sequences=21	504	21
SDS-V3-plasma-67_Cluster_47445_sequences=21	504	21
SDS-V3-plasma-67_Cluster_5478_sequences=21	504	21
SDS-V3-plasma-67_Cluster_1023_sequences=21	504	21
SDS-V3-plasma-67_Cluster_7045_sequences=21	504	21
SDS-V3-plasma-0_Cluster_3852_sequences=20	0	20

SDS-V3-plasma-0_Cluster_3103_sequences=20	0	20
SDS-V3-plasma-0_Cluster_8166_sequences=20	0	20
SDS-V3-plasma-0_Cluster_1252_sequences=20	0	20
SDS-V3-plasma-0_Cluster_1825_sequences=20	0	20
SDS-V3-plasma-0_Cluster_2443_sequences=20	0	20
SDS-V3-plasma-0_Cluster_567_sequences=20	0	20
SDS-V3-plasma-0_Cluster_826_sequences=20	0	20
SDS-V3-plasma-0_Cluster_1739_sequences=20	0	20
SDS-V3-plasma-0_Cluster_3487_sequences=20	0	20
SDS-V3-plasma-0_Cluster_484_sequences=20	0	20
SDS-V3-plasma-0_Cluster_849_sequences=20	0	20
SDS-V3-plasma-0_Cluster_45_sequences=20	0	20
SDS-V3-plasma-0_Cluster_744_sequences=20	0	20
SDS-V3-plasma-0_Cluster_5518_sequences=20	0	20
SDS-V3-plasma-0_Cluster_3717_sequences=20	0	20
SDS-V3-plasma-0_Cluster_3825_sequences=20	0	20
SDS-V3-plasma-0_Cluster_1075_sequences=20	0	20
SDS-V3-plasma-0_Cluster_2543_sequences=20	0	20
SDS-V3-plasma-0_Cluster_2598_sequences=20	0	20
SDS-V3-plasma-0_Cluster_451_sequences=20	0	20
SDS-V3-plasma-0_Cluster_4694_sequences=20	0	20
SDS-V3-plasma-0_Cluster_971_sequences=20	0	20
SDS-V3-plasma-0_Cluster_2472_sequences=20	0	20
SDS-V3-plasma-0_Cluster_5726_sequences=20	0	20
SDS-V3-plasma-0_Cluster_8958_sequences=20	0	20
SDS-V3-plasma-0_Cluster_904_sequences=20	0	20
SDS-V3-plasma-0_Cluster_3765_sequences=20	0	20
SDS-V3-plasma-0_Cluster_1589_sequences=20	0	20
SDS-V3-plasma-0_Cluster_3539_sequences=20	0	20
SDS-V3-plasma-0_Cluster_3499_sequences=20	0	20
SDS-V3-plasma-0_Cluster_8974_sequences=20	0	20
SDS-V3-plasma-0_Cluster_300_sequences=20	0	20
SDS-V3-plasma-5_Cluster_102_sequences=20	9	20
SDS-V3-plasma-5_Cluster_335_sequences=20	9	20
SDS-V3-plasma-5_Cluster_240_sequences=20	9	20
SDS-V3-plasma-5_Cluster_79_sequences=20	9	20
SDS-V3-plasma-5_Cluster_194_sequences=20	9	20
SDS-V3-plasma-5_Cluster_310_sequences=20	9	20
SDS-V3-plasma-5_Cluster_403_sequences=20	9	20
SDS-V3-plasma-7_Cluster_157_sequences=20	14	20
SDS-V3-plasma-7_Cluster_756_sequences=20	14	20
SDS-V3-plasma-7_Cluster_1557_sequences=20	14	20
SDS-V3-plasma-7_Cluster_1063_sequences=20	14	20
SDS-V3-plasma-7_Cluster_2770_sequences=20	14	20
SDS-V3-plasma-7_Cluster_253_sequences=20	14	20

SDS-V3-plasma-7_Cluster_1820_sequences=20	14	20
SDS-V3-plasma-8_Cluster_228_sequences=20	16	20
SDS-V3-plasma-8_Cluster_45_sequences=20	16	20
SDS-V3-plasma-8_Cluster_3455_sequences=20	16	20
SDS-V3-plasma-8_Cluster_149_sequences=20	16	20
SDS-V3-plasma-8_Cluster_2791_sequences=20	16	20
SDS-V3-plasma-8_Cluster_2353_sequences=20	16	20
SDS-V3-plasma-8_Cluster_2423_sequences=20	16	20
SDS-V3-plasma-8_Cluster_2755_sequences=20	16	20
SDS-V3-plasma-8_Cluster_2584_sequences=20	16	20
SDS-V3-plasma-24_Cluster_1494_sequences=20	124	20
SDS-V3-plasma-24_Cluster_1983_sequences=20	124	20
SDS-V3-plasma-24_Cluster_440_sequences=20	124	20
SDS-V3-plasma-24_Cluster_48_sequences=20	124	20
SDS-V3-plasma-24_Cluster_55_sequences=20	124	20
SDS-V3-plasma-24_Cluster_902_sequences=20	124	20
SDS-V3-plasma-24_Cluster_946_sequences=20	124	20
SDS-V3-plasma-24_Cluster_242_sequences=20	124	20
SDS-V3-plasma-24_Cluster_2444_sequences=20	124	20
SDS-V3-plasma-24_Cluster_1011_sequences=20	124	20
SDS-V3-plasma-24_Cluster_1531_sequences=20	124	20
SDS-V3-plasma-24_Cluster_1736_sequences=20	124	20
SDS-V3-plasma-24_Cluster_1209_sequences=20	124	20
SDS-V3-plasma-24_Cluster_1587_sequences=20	124	20
SDS-V3-plasma-27_Cluster_2056_sequences=20	131	20
SDS-V3-plasma-27_Cluster_2072_sequences=20	131	20
SDS-V3-plasma-27_Cluster_2350_sequences=20	131	20
SDS-V3-plasma-27_Cluster_3325_sequences=20	131	20
SDS-V3-plasma-27_Cluster_3327_sequences=20	131	20
SDS-V3-plasma-27_Cluster_34_sequences=20	131	20
SDS-V3-plasma-27_Cluster_35_sequences=20	131	20
SDS-V3-plasma-27_Cluster_520_sequences=20	131	20
SDS-V3-plasma-27_Cluster_813_sequences=20	131	20
SDS-V3-plasma-27_Cluster_949_sequences=20	131	20
SDS-V3-plasma-27_Cluster_952_sequences=20	131	20
SDS-V3-plasma-27_Cluster_411_sequences=20	131	20
SDS-V3-plasma-27_Cluster_1121_sequences=20	131	20
SDS-V3-plasma-27_Cluster_644_sequences=20	131	20
SDS-V3-plasma-27_Cluster_1955_sequences=20	131	20
SDS-V3-plasma-27_Cluster_1833_sequences=20	131	20
SDS-V3-plasma-45_Cluster_13455_sequences=20	282	20
SDS-V3-plasma-45_Cluster_8268_sequences=20	282	20
SDS-V3-plasma-45_Cluster_10789_sequences=20	282	20
SDS-V3-plasma-45_Cluster_13930_sequences=20	282	20
SDS-V3-plasma-45_Cluster_11390_sequences=20	282	20

SDS-V3-plasma-45_Cluster_7295_sequences=20	282	20
SDS-V3-plasma-45_Cluster_8796_sequences=20	282	20
SDS-V3-plasma-45_Cluster_8146_sequences=20	282	20
SDS-V3-plasma-45_Cluster_5879_sequences=20	282	20
SDS-V3-plasma-45_Cluster_7687_sequences=20	282	20
SDS-V3-plasma-45_Cluster_3018_sequences=20	282	20
SDS-V3-plasma-45_Cluster_10564_sequences=20	282	20
SDS-V3-plasma-45_Cluster_11190_sequences=20	282	20
SDS-V3-plasma-45_Cluster_13113_sequences=20	282	20
SDS-V3-plasma-45_Cluster_16154_sequences=20	282	20
SDS-V3-plasma-45_Cluster_16244_sequences=20	282	20
SDS-V3-plasma-45_Cluster_1898_sequences=20	282	20
SDS-V3-plasma-45_Cluster_5258_sequences=20	282	20
SDS-V3-plasma-45_Cluster_6042_sequences=20	282	20
SDS-V3-plasma-45_Cluster_10755_sequences=20	282	20
SDS-V3-plasma-45_Cluster_2949_sequences=20	282	20
SDS-V3-plasma-45_Cluster_3106_sequences=20	282	20
SDS-V3-plasma-45_Cluster_3755_sequences=20	282	20
SDS-V3-plasma-45_Cluster_196_sequences=20	282	20
SDS-V3-plasma-45_Cluster_9066_sequences=20	282	20
SDS-V3-plasma-45_Cluster_1169_sequences=20	282	20
SDS-V3-plasma-45_Cluster_5689_sequences=20	282	20
SDS-V3-plasma-45_Cluster_1184_sequences=20	282	20
SDS-V3-plasma-45_Cluster_12733_sequences=20	282	20
SDS-V3-plasma-45_Cluster_1851_sequences=20	282	20
SDS-V3-plasma-45_Cluster_14579_sequences=20	282	20
SDS-V3-plasma-45_Cluster_22521_sequences=20	282	20
SDS-V3-plasma-45_Cluster_3270_sequences=20	282	20
SDS-V3-plasma-45_Cluster_839_sequences=20	282	20
SDS-V3-plasma-45_Cluster_7907_sequences=20	282	20
SDS-V3-plasma-45_Cluster_13852_sequences=20	282	20
SDS-V3-plasma-45_Cluster_11017_sequences=20	282	20
SDS-V3-plasma-45_Cluster_17510_sequences=20	282	20
SDS-V3-plasma-45_Cluster_555_sequences=20	282	20
SDS-V3-plasma-45_Cluster_22352_sequences=20	282	20
SDS-V3-plasma-45_Cluster_3078_sequences=20	282	20
SDS-V3-plasma-45_Cluster_16593_sequences=20	282	20
SDS-V3-plasma-45_Cluster_2746_sequences=20	282	20
SDS-V3-plasma-45_Cluster_13421_sequences=20	282	20
SDS-V3-plasma-45_Cluster_24781_sequences=20	282	20
SDS-V3-plasma-45_Cluster_6717_sequences=20	282	20
SDS-V3-plasma-45_Cluster_2780_sequences=20	282	20
SDS-V3-plasma-45_Cluster_6213_sequences=20	282	20
SDS-V3-plasma-45_Cluster_14032_sequences=20	282	20
SDS-V3-plasma-45_Cluster_5469_sequences=20	282	20

SDS-V3-plasma-45_Cluster_4235_sequences=20	282	20
SDS-V3-plasma-45_Cluster_2303_sequences=20	282	20
SDS-V3-plasma-45_Cluster_12987_sequences=20	282	20
SDS-V3-plasma-45_Cluster_3721_sequences=20	282	20
SDS-V3-plasma-45_Cluster_18825_sequences=20	282	20
SDS-V3-plasma-45_Cluster_10918_sequences=20	282	20
SDS-V3-plasma-45_Cluster_63996_sequences=20	282	20
SDS-V3-plasma-45_Cluster_6608_sequences=20	282	20
SDS-V3-plasma-45_Cluster_2059_sequences=20	282	20
SDS-V3-plasma-45_Cluster_996_sequences=20	282	20
SDS-V3-plasma-45_Cluster_2676_sequences=20	282	20
SDS-V3-plasma-45_Cluster_7517_sequences=20	282	20
SDS-V3-plasma-45_Cluster_2555_sequences=20	282	20
SDS-V3-plasma-45_Cluster_5554_sequences=20	282	20
SDS-V3-plasma-45_Cluster_22383_sequences=20	282	20
SDS-V3-plasma-45_Cluster_18383_sequences=20	282	20
SDS-V3-plasma-45_Cluster_10056_sequences=20	282	20
SDS-V3-plasma-45_Cluster_12542_sequences=20	282	20
SDS-V3-plasma-45_Cluster_6045_sequences=20	282	20
SDS-V3-plasma-45_Cluster_33368_sequences=20	282	20
SDS-V3-plasma-45_Cluster_11522_sequences=20	282	20
SDS-V3-plasma-45_Cluster_3435_sequences=20	282	20
SDS-V3-plasma-45_Cluster_5624_sequences=20	282	20
SDS-V3-plasma-45_Cluster_3760_sequences=20	282	20
SDS-V3-plasma-45_Cluster_6741_sequences=20	282	20
SDS-V3-plasma-45_Cluster_17642_sequences=20	282	20
SDS-V3-plasma-45_Cluster_10702_sequences=20	282	20
SDS-V3-plasma-45_Cluster_2743_sequences=20	282	20
SDS-V3-plasma-45_Cluster_985_sequences=20	282	20
SDS-V3-plasma-46_Cluster_1124_sequences=20	286	20
SDS-V3-plasma-46_Cluster_4142_sequences=20	286	20
SDS-V3-plasma-46_Cluster_4871_sequences=20	286	20
SDS-V3-plasma-46_Cluster_1481_sequences=20	286	20
SDS-V3-plasma-46_Cluster_3148_sequences=20	286	20
SDS-V3-plasma-46_Cluster_6367_sequences=20	286	20
SDS-V3-plasma-46_Cluster_8090_sequences=20	286	20
SDS-V3-plasma-46_Cluster_13473_sequences=20	286	20
SDS-V3-plasma-46_Cluster_1556_sequences=20	286	20
SDS-V3-plasma-46_Cluster_2123_sequences=20	286	20
SDS-V3-plasma-46_Cluster_309_sequences=20	286	20
SDS-V3-plasma-46_Cluster_4061_sequences=20	286	20
SDS-V3-plasma-46_Cluster_4313_sequences=20	286	20
SDS-V3-plasma-46_Cluster_5765_sequences=20	286	20
SDS-V3-plasma-46_Cluster_6658_sequences=20	286	20
SDS-V3-plasma-46_Cluster_1622_sequences=20	286	20

SDS-V3-plasma-46_Cluster_2185_sequences=20	286	20
SDS-V3-plasma-46_Cluster_1134_sequences=20	286	20
SDS-V3-plasma-46_Cluster_1272_sequences=20	286	20
SDS-V3-plasma-46_Cluster_2030_sequences=20	286	20
SDS-V3-plasma-46_Cluster_263_sequences=20	286	20
SDS-V3-plasma-46_Cluster_4836_sequences=20	286	20
SDS-V3-plasma-46_Cluster_6294_sequences=20	286	20
SDS-V3-plasma-46_Cluster_3649_sequences=20	286	20
SDS-V3-plasma-46_Cluster_4109_sequences=20	286	20
SDS-V3-plasma-46_Cluster_1227_sequences=20	286	20
SDS-V3-plasma-46_Cluster_3094_sequences=20	286	20
SDS-V3-plasma-46_Cluster_4179_sequences=20	286	20
SDS-V3-plasma-46_Cluster_1957_sequences=20	286	20
SDS-V3-plasma-67_Cluster_15846_sequences=20	504	20
SDS-V3-plasma-67_Cluster_11557_sequences=20	504	20
SDS-V3-plasma-67_Cluster_23692_sequences=20	504	20
SDS-V3-plasma-67_Cluster_10230_sequences=20	504	20
SDS-V3-plasma-67_Cluster_1825_sequences=20	504	20
SDS-V3-plasma-67_Cluster_57_sequences=20	504	20
SDS-V3-plasma-67_Cluster_21940_sequences=20	504	20
SDS-V3-plasma-67_Cluster_10113_sequences=20	504	20
SDS-V3-plasma-67_Cluster_13715_sequences=20	504	20
SDS-V3-plasma-67_Cluster_3720_sequences=20	504	20
SDS-V3-plasma-67_Cluster_35068_sequences=20	504	20
SDS-V3-plasma-67_Cluster_22527_sequences=20	504	20
SDS-V3-plasma-67_Cluster_13358_sequences=20	504	20
SDS-V3-plasma-67_Cluster_1913_sequences=20	504	20
SDS-V3-plasma-67_Cluster_2982_sequences=20	504	20
SDS-V3-plasma-67_Cluster_3651_sequences=20	504	20
SDS-V3-plasma-67_Cluster_3903_sequences=20	504	20
SDS-V3-plasma-67_Cluster_4447_sequences=20	504	20
SDS-V3-plasma-67_Cluster_6701_sequences=20	504	20
SDS-V3-plasma-67_Cluster_9022_sequences=20	504	20
SDS-V3-plasma-67_Cluster_9212_sequences=20	504	20
SDS-V3-plasma-67_Cluster_18579_sequences=20	504	20
SDS-V3-plasma-67_Cluster_7170_sequences=20	504	20
SDS-V3-plasma-67_Cluster_1700_sequences=20	504	20
SDS-V3-plasma-67_Cluster_14877_sequences=20	504	20
SDS-V3-plasma-67_Cluster_4966_sequences=20	504	20
SDS-V3-plasma-67_Cluster_11482_sequences=20	504	20
SDS-V3-plasma-67_Cluster_19138_sequences=20	504	20
SDS-V3-plasma-67_Cluster_17662_sequences=20	504	20
SDS-V3-plasma-67_Cluster_15342_sequences=20	504	20
SDS-V3-plasma-67_Cluster_1781_sequences=20	504	20
SDS-V3-plasma-67_Cluster_20107_sequences=20	504	20

SDS-V3-plasma-67_Cluster_17783_sequences=20	504	20
SDS-V3-plasma-67_Cluster_28475_sequences=20	504	20
SDS-V3-plasma-67_Cluster_27763_sequences=20	504	20
SDS-V3-plasma-67_Cluster_20177_sequences=20	504	20
SDS-V3-plasma-67_Cluster_27308_sequences=20	504	20
SDS-V3-plasma-67_Cluster_29919_sequences=20	504	20
SDS-V3-plasma-67_Cluster_5605_sequences=20	504	20
SDS-V3-plasma-67_Cluster_13504_sequences=20	504	20
SDS-V3-plasma-67_Cluster_1880_sequences=20	504	20
SDS-V3-plasma-67_Cluster_7372_sequences=20	504	20
SDS-V3-plasma-0_Cluster_1637_sequences=19	0	19
SDS-V3-plasma-0_Cluster_2371_sequences=19	0	19
SDS-V3-plasma-0_Cluster_1300_sequences=19	0	19
SDS-V3-plasma-0_Cluster_1860_sequences=19	0	19
SDS-V3-plasma-0_Cluster_3492_sequences=19	0	19
SDS-V3-plasma-0_Cluster_4592_sequences=19	0	19
SDS-V3-plasma-0_Cluster_585_sequences=19	0	19
SDS-V3-plasma-0_Cluster_7190_sequences=19	0	19
SDS-V3-plasma-0_Cluster_8287_sequences=19	0	19
SDS-V3-plasma-0_Cluster_3196_sequences=19	0	19
SDS-V3-plasma-0_Cluster_6956_sequences=19	0	19
SDS-V3-plasma-0_Cluster_373_sequences=19	0	19
SDS-V3-plasma-0_Cluster_2110_sequences=19	0	19
SDS-V3-plasma-0_Cluster_6993_sequences=19	0	19
SDS-V3-plasma-0_Cluster_2985_sequences=19	0	19
SDS-V3-plasma-0_Cluster_1915_sequences=19	0	19
SDS-V3-plasma-0_Cluster_143_sequences=19	0	19
SDS-V3-plasma-0_Cluster_618_sequences=19	0	19
SDS-V3-plasma-0_Cluster_3583_sequences=19	0	19
SDS-V3-plasma-0_Cluster_265_sequences=19	0	19
SDS-V3-plasma-0_Cluster_5329_sequences=19	0	19
SDS-V3-plasma-0_Cluster_1565_sequences=19	0	19
SDS-V3-plasma-0_Cluster_25170_sequences=19	0	19
SDS-V3-plasma-0_Cluster_4399_sequences=19	0	19
SDS-V3-plasma-0_Cluster_834_sequences=19	0	19
SDS-V3-plasma-0_Cluster_1299_sequences=19	0	19
SDS-V3-plasma-0_Cluster_476_sequences=19	0	19
SDS-V3-plasma-0_Cluster_377_sequences=19	0	19
SDS-V3-plasma-0_Cluster_2712_sequences=19	0	19
SDS-V3-plasma-5_Cluster_514_sequences=19	9	19
SDS-V3-plasma-5_Cluster_723_sequences=19	9	19
SDS-V3-plasma-5_Cluster_665_sequences=19	9	19
SDS-V3-plasma-7_Cluster_553_sequences=19	14	19
SDS-V3-plasma-7_Cluster_1170_sequences=19	14	19
SDS-V3-plasma-7_Cluster_934_sequences=19	14	19

SDS-V3-plasma-7_Cluster_2598_sequences=19	14	19
SDS-V3-plasma-7_Cluster_475_sequences=19	14	19
SDS-V3-plasma-7_Cluster_1837_sequences=19	14	19
SDS-V3-plasma-7_Cluster_4389_sequences=19	14	19
SDS-V3-plasma-7_Cluster_2693_sequences=19	14	19
SDS-V3-plasma-7_Cluster_539_sequences=19	14	19
SDS-V3-plasma-7_Cluster_205_sequences=19	14	19
SDS-V3-plasma-7_Cluster_2203_sequences=19	14	19
SDS-V3-plasma-7_Cluster_3240_sequences=19	14	19
SDS-V3-plasma-7_Cluster_1804_sequences=19	14	19
SDS-V3-plasma-7_Cluster_855_sequences=19	14	19
SDS-V3-plasma-7_Cluster_60_sequences=19	14	19
SDS-V3-plasma-8_Cluster_150_sequences=19	16	19
SDS-V3-plasma-8_Cluster_5192_sequences=19	16	19
SDS-V3-plasma-8_Cluster_3284_sequences=19	16	19
SDS-V3-plasma-8_Cluster_3533_sequences=19	16	19
SDS-V3-plasma-8_Cluster_2740_sequences=19	16	19
SDS-V3-plasma-8_Cluster_3138_sequences=19	16	19
SDS-V3-plasma-8_Cluster_2675_sequences=19	16	19
SDS-V3-plasma-8_Cluster_4187_sequences=19	16	19
SDS-V3-plasma-8_Cluster_2503_sequences=19	16	19
SDS-V3-plasma-8_Cluster_4166_sequences=19	16	19
SDS-V3-plasma-8_Cluster_2843_sequences=19	16	19
SDS-V3-plasma-8_Cluster_3846_sequences=19	16	19
SDS-V3-plasma-8_Cluster_2631_sequences=19	16	19
SDS-V3-plasma-24_Cluster_1063_sequences=19	124	19
SDS-V3-plasma-24_Cluster_1260_sequences=19	124	19
SDS-V3-plasma-24_Cluster_1766_sequences=19	124	19
SDS-V3-plasma-24_Cluster_3892_sequences=19	124	19
SDS-V3-plasma-24_Cluster_647_sequences=19	124	19
SDS-V3-plasma-24_Cluster_2646_sequences=19	124	19
SDS-V3-plasma-24_Cluster_3919_sequences=19	124	19
SDS-V3-plasma-24_Cluster_379_sequences=19	124	19
SDS-V3-plasma-24_Cluster_2655_sequences=19	124	19
SDS-V3-plasma-24_Cluster_947_sequences=19	124	19
SDS-V3-plasma-24_Cluster_152_sequences=19	124	19
SDS-V3-plasma-24_Cluster_469_sequences=19	124	19
SDS-V3-plasma-24_Cluster_1969_sequences=19	124	19
SDS-V3-plasma-24_Cluster_2087_sequences=19	124	19
SDS-V3-plasma-24_Cluster_3382_sequences=19	124	19
SDS-V3-plasma-27_Cluster_1117_sequences=19	131	19
SDS-V3-plasma-27_Cluster_1969_sequences=19	131	19
SDS-V3-plasma-27_Cluster_2405_sequences=19	131	19
SDS-V3-plasma-27_Cluster_2420_sequences=19	131	19
SDS-V3-plasma-27_Cluster_3303_sequences=19	131	19

SDS-V3-plasma-27_Cluster_542_sequences=19	131	19
SDS-V3-plasma-27_Cluster_576_sequences=19	131	19
SDS-V3-plasma-27_Cluster_2615_sequences=19	131	19
SDS-V3-plasma-27_Cluster_990_sequences=19	131	19
SDS-V3-plasma-27_Cluster_469_sequences=19	131	19
SDS-V3-plasma-27_Cluster_587_sequences=19	131	19
SDS-V3-plasma-27_Cluster_938_sequences=19	131	19
SDS-V3-plasma-27_Cluster_389_sequences=19	131	19
SDS-V3-plasma-27_Cluster_1178_sequences=19	131	19
SDS-V3-plasma-45_Cluster_1531_sequences=19	282	19
SDS-V3-plasma-45_Cluster_33237_sequences=19	282	19
SDS-V3-plasma-45_Cluster_13382_sequences=19	282	19
SDS-V3-plasma-45_Cluster_2793_sequences=19	282	19
SDS-V3-plasma-45_Cluster_4739_sequences=19	282	19
SDS-V3-plasma-45_Cluster_15868_sequences=19	282	19
SDS-V3-plasma-45_Cluster_4133_sequences=19	282	19
SDS-V3-plasma-45_Cluster_10595_sequences=19	282	19
SDS-V3-plasma-45_Cluster_13267_sequences=19	282	19
SDS-V3-plasma-45_Cluster_3048_sequences=19	282	19
SDS-V3-plasma-45_Cluster_3783_sequences=19	282	19
SDS-V3-plasma-45_Cluster_65_sequences=19	282	19
SDS-V3-plasma-45_Cluster_7023_sequences=19	282	19
SDS-V3-plasma-45_Cluster_8831_sequences=19	282	19
SDS-V3-plasma-45_Cluster_9152_sequences=19	282	19
SDS-V3-plasma-45_Cluster_11250_sequences=19	282	19
SDS-V3-plasma-45_Cluster_16615_sequences=19	282	19
SDS-V3-plasma-45_Cluster_6643_sequences=19	282	19
SDS-V3-plasma-45_Cluster_13402_sequences=19	282	19
SDS-V3-plasma-45_Cluster_7927_sequences=19	282	19
SDS-V3-plasma-45_Cluster_9640_sequences=19	282	19
SDS-V3-plasma-45_Cluster_7324_sequences=19	282	19
SDS-V3-plasma-45_Cluster_10884_sequences=19	282	19
SDS-V3-plasma-45_Cluster_12604_sequences=19	282	19
SDS-V3-plasma-45_Cluster_12879_sequences=19	282	19
SDS-V3-plasma-45_Cluster_21121_sequences=19	282	19
SDS-V3-plasma-45_Cluster_13945_sequences=19	282	19
SDS-V3-plasma-45_Cluster_36145_sequences=19	282	19
SDS-V3-plasma-45_Cluster_17939_sequences=19	282	19
SDS-V3-plasma-45_Cluster_27747_sequences=19	282	19
SDS-V3-plasma-45_Cluster_2447_sequences=19	282	19
SDS-V3-plasma-45_Cluster_908_sequences=19	282	19
SDS-V3-plasma-45_Cluster_4885_sequences=19	282	19
SDS-V3-plasma-45_Cluster_9887_sequences=19	282	19
SDS-V3-plasma-45_Cluster_5705_sequences=19	282	19
SDS-V3-plasma-45_Cluster_5808_sequences=19	282	19

SDS-V3-plasma-45_Cluster_1385_sequences=19	282	19
SDS-V3-plasma-45_Cluster_2239_sequences=19	282	19
SDS-V3-plasma-45_Cluster_25352_sequences=19	282	19
SDS-V3-plasma-45_Cluster_3547_sequences=19	282	19
SDS-V3-plasma-45_Cluster_1603_sequences=19	282	19
SDS-V3-plasma-45_Cluster_13171_sequences=19	282	19
SDS-V3-plasma-45_Cluster_21484_sequences=19	282	19
SDS-V3-plasma-45_Cluster_11035_sequences=19	282	19
SDS-V3-plasma-45_Cluster_6100_sequences=19	282	19
SDS-V3-plasma-45_Cluster_12606_sequences=19	282	19
SDS-V3-plasma-45_Cluster_24062_sequences=19	282	19
SDS-V3-plasma-45_Cluster_15685_sequences=19	282	19
SDS-V3-plasma-45_Cluster_7307_sequences=19	282	19
SDS-V3-plasma-45_Cluster_14316_sequences=19	282	19
SDS-V3-plasma-45_Cluster_6530_sequences=19	282	19
SDS-V3-plasma-45_Cluster_7648_sequences=19	282	19
SDS-V3-plasma-45_Cluster_14612_sequences=19	282	19
SDS-V3-plasma-45_Cluster_15518_sequences=19	282	19
SDS-V3-plasma-45_Cluster_1650_sequences=19	282	19
SDS-V3-plasma-45_Cluster_14000_sequences=19	282	19
SDS-V3-plasma-45_Cluster_66671_sequences=19	282	19
SDS-V3-plasma-45_Cluster_728_sequences=19	282	19
SDS-V3-plasma-45_Cluster_6505_sequences=19	282	19
SDS-V3-plasma-45_Cluster_9915_sequences=19	282	19
SDS-V3-plasma-45_Cluster_10576_sequences=19	282	19
SDS-V3-plasma-45_Cluster_20856_sequences=19	282	19
SDS-V3-plasma-45_Cluster_11982_sequences=19	282	19
SDS-V3-plasma-45_Cluster_13620_sequences=19	282	19
SDS-V3-plasma-45_Cluster_13641_sequences=19	282	19
SDS-V3-plasma-45_Cluster_579_sequences=19	282	19
SDS-V3-plasma-45_Cluster_7319_sequences=19	282	19
SDS-V3-plasma-45_Cluster_2266_sequences=19	282	19
SDS-V3-plasma-45_Cluster_24281_sequences=19	282	19
SDS-V3-plasma-45_Cluster_21171_sequences=19	282	19
SDS-V3-plasma-45_Cluster_6134_sequences=19	282	19
SDS-V3-plasma-45_Cluster_16873_sequences=19	282	19
SDS-V3-plasma-45_Cluster_12803_sequences=19	282	19
SDS-V3-plasma-45_Cluster_10151_sequences=19	282	19
SDS-V3-plasma-45_Cluster_9405_sequences=19	282	19
SDS-V3-plasma-45_Cluster_1345_sequences=19	282	19
SDS-V3-plasma-45_Cluster_10011_sequences=19	282	19
SDS-V3-plasma-45_Cluster_6044_sequences=19	282	19
SDS-V3-plasma-45_Cluster_4589_sequences=19	282	19
SDS-V3-plasma-45_Cluster_17216_sequences=19	282	19
SDS-V3-plasma-45_Cluster_2453_sequences=19	282	19

SDS-V3-plasma-45_Cluster_13057_sequences=19	282	19
SDS-V3-plasma-45_Cluster_3863_sequences=19	282	19
SDS-V3-plasma-45_Cluster_12890_sequences=19	282	19
SDS-V3-plasma-45_Cluster_12612_sequences=19	282	19
SDS-V3-plasma-45_Cluster_11762_sequences=19	282	19
SDS-V3-plasma-45_Cluster_3039_sequences=19	282	19
SDS-V3-plasma-45_Cluster_4655_sequences=19	282	19
SDS-V3-plasma-46_Cluster_2843_sequences=19	286	19
SDS-V3-plasma-46_Cluster_857_sequences=19	286	19
SDS-V3-plasma-46_Cluster_1677_sequences=19	286	19
SDS-V3-plasma-46_Cluster_6347_sequences=19	286	19
SDS-V3-plasma-46_Cluster_2140_sequences=19	286	19
SDS-V3-plasma-46_Cluster_3002_sequences=19	286	19
SDS-V3-plasma-46_Cluster_692_sequences=19	286	19
SDS-V3-plasma-46_Cluster_5692_sequences=19	286	19
SDS-V3-plasma-46_Cluster_5982_sequences=19	286	19
SDS-V3-plasma-46_Cluster_7014_sequences=19	286	19
SDS-V3-plasma-46_Cluster_1397_sequences=19	286	19
SDS-V3-plasma-46_Cluster_1713_sequences=19	286	19
SDS-V3-plasma-46_Cluster_1920_sequences=19	286	19
SDS-V3-plasma-46_Cluster_5955_sequences=19	286	19
SDS-V3-plasma-46_Cluster_1189_sequences=19	286	19
SDS-V3-plasma-46_Cluster_3504_sequences=19	286	19
SDS-V3-plasma-46_Cluster_596_sequences=19	286	19
SDS-V3-plasma-46_Cluster_7908_sequences=19	286	19
SDS-V3-plasma-46_Cluster_995_sequences=19	286	19
SDS-V3-plasma-46_Cluster_7741_sequences=19	286	19
SDS-V3-plasma-46_Cluster_2694_sequences=19	286	19
SDS-V3-plasma-46_Cluster_1281_sequences=19	286	19
SDS-V3-plasma-46_Cluster_3917_sequences=19	286	19
SDS-V3-plasma-46_Cluster_3333_sequences=19	286	19
SDS-V3-plasma-46_Cluster_1886_sequences=19	286	19
SDS-V3-plasma-46_Cluster_424_sequences=19	286	19
SDS-V3-plasma-46_Cluster_8032_sequences=19	286	19
SDS-V3-plasma-46_Cluster_4770_sequences=19	286	19
SDS-V3-plasma-46_Cluster_8099_sequences=19	286	19
SDS-V3-plasma-46_Cluster_11676_sequences=19	286	19
SDS-V3-plasma-46_Cluster_6074_sequences=19	286	19
SDS-V3-plasma-46_Cluster_2722_sequences=19	286	19
SDS-V3-plasma-46_Cluster_6825_sequences=19	286	19
SDS-V3-plasma-67_Cluster_21022_sequences=19	504	19
SDS-V3-plasma-67_Cluster_5898_sequences=19	504	19
SDS-V3-plasma-67_Cluster_10351_sequences=19	504	19
SDS-V3-plasma-67_Cluster_149_sequences=19	504	19
SDS-V3-plasma-67_Cluster_9813_sequences=19	504	19

SDS-V3-plasma-67_Cluster_17149_sequences=19	504	19
SDS-V3-plasma-67_Cluster_16768_sequences=19	504	19
SDS-V3-plasma-67_Cluster_5356_sequences=19	504	19
SDS-V3-plasma-67_Cluster_6312_sequences=19	504	19
SDS-V3-plasma-67_Cluster_31584_sequences=19	504	19
SDS-V3-plasma-67_Cluster_8527_sequences=19	504	19
SDS-V3-plasma-67_Cluster_15143_sequences=19	504	19
SDS-V3-plasma-67_Cluster_3889_sequences=19	504	19
SDS-V3-plasma-67_Cluster_5823_sequences=19	504	19
SDS-V3-plasma-67_Cluster_9634_sequences=19	504	19
SDS-V3-plasma-67_Cluster_7879_sequences=19	504	19
SDS-V3-plasma-67_Cluster_4649_sequences=19	504	19
SDS-V3-plasma-67_Cluster_16780_sequences=19	504	19
SDS-V3-plasma-67_Cluster_6512_sequences=19	504	19
SDS-V3-plasma-67_Cluster_5713_sequences=19	504	19
SDS-V3-plasma-67_Cluster_8394_sequences=19	504	19
SDS-V3-plasma-67_Cluster_15437_sequences=19	504	19
SDS-V3-plasma-67_Cluster_1363_sequences=19	504	19
SDS-V3-plasma-67_Cluster_6576_sequences=19	504	19
SDS-V3-plasma-67_Cluster_3531_sequences=19	504	19
SDS-V3-plasma-67_Cluster_5892_sequences=19	504	19
SDS-V3-plasma-67_Cluster_10106_sequences=19	504	19
SDS-V3-plasma-67_Cluster_10657_sequences=19	504	19
SDS-V3-plasma-67_Cluster_13675_sequences=19	504	19
SDS-V3-plasma-67_Cluster_13813_sequences=19	504	19
SDS-V3-plasma-67_Cluster_15212_sequences=19	504	19
SDS-V3-plasma-67_Cluster_15410_sequences=19	504	19
SDS-V3-plasma-67_Cluster_15628_sequences=19	504	19
SDS-V3-plasma-67_Cluster_1988_sequences=19	504	19
SDS-V3-plasma-67_Cluster_20633_sequences=19	504	19
SDS-V3-plasma-67_Cluster_2665_sequences=19	504	19
SDS-V3-plasma-67_Cluster_284_sequences=19	504	19
SDS-V3-plasma-67_Cluster_30128_sequences=19	504	19
SDS-V3-plasma-67_Cluster_3054_sequences=19	504	19
SDS-V3-plasma-67_Cluster_4242_sequences=19	504	19
SDS-V3-plasma-67_Cluster_4651_sequences=19	504	19
SDS-V3-plasma-67_Cluster_9117_sequences=19	504	19
SDS-V3-plasma-67_Cluster_1476_sequences=19	504	19
SDS-V3-plasma-67_Cluster_5507_sequences=19	504	19
SDS-V3-plasma-67_Cluster_19449_sequences=19	504	19
SDS-V3-plasma-67_Cluster_10254_sequences=19	504	19
SDS-V3-plasma-67_Cluster_1692_sequences=19	504	19
SDS-V3-plasma-67_Cluster_3819_sequences=19	504	19
SDS-V3-plasma-67_Cluster_16299_sequences=19	504	19
SDS-V3-plasma-67_Cluster_11754_sequences=19	504	19

SDS-V3-plasma-67_Cluster_7988_sequences=19	504	19
SDS-V3-plasma-67_Cluster_7431_sequences=19	504	19
SDS-V3-plasma-67_Cluster_8182_sequences=19	504	19
SDS-V3-plasma-67_Cluster_17267_sequences=19	504	19
SDS-V3-plasma-67_Cluster_10125_sequences=19	504	19
SDS-V3-plasma-67_Cluster_3025_sequences=19	504	19
SDS-V3-plasma-67_Cluster_16853_sequences=19	504	19
SDS-V3-plasma-67_Cluster_11268_sequences=19	504	19
SDS-V3-plasma-67_Cluster_19639_sequences=19	504	19
SDS-V3-plasma-67_Cluster_21232_sequences=19	504	19
SDS-V3-plasma-67_Cluster_12867_sequences=19	504	19
SDS-V3-plasma-67_Cluster_17691_sequences=19	504	19
SDS-V3-plasma-67_Cluster_21982_sequences=19	504	19
SDS-V3-plasma-67_Cluster_14012_sequences=19	504	19
SDS-V3-plasma-67_Cluster_11208_sequences=19	504	19
SDS-V3-plasma-67_Cluster_2627_sequences=19	504	19
SDS-V3-plasma-67_Cluster_15262_sequences=19	504	19
SDS-V3-plasma-67_Cluster_2990_sequences=19	504	19
SDS-V3-plasma-67_Cluster_750_sequences=19	504	19
SDS-V3-plasma-67_Cluster_5821_sequences=19	504	19
SDS-V3-plasma-67_Cluster_7591_sequences=19	504	19
SDS-V3-plasma-67_Cluster_7144_sequences=19	504	19
SDS-V3-plasma-67_Cluster_2455_sequences=19	504	19
SDS-V3-plasma-0_Cluster_1166_sequences=18	0	18
SDS-V3-plasma-0_Cluster_2828_sequences=18	0	18
SDS-V3-plasma-0_Cluster_991_sequences=18	0	18
SDS-V3-plasma-0_Cluster_1558_sequences=18	0	18
SDS-V3-plasma-0_Cluster_2853_sequences=18	0	18
SDS-V3-plasma-0_Cluster_4131_sequences=18	0	18
SDS-V3-plasma-0_Cluster_4678_sequences=18	0	18
SDS-V3-plasma-0_Cluster_9132_sequences=18	0	18
SDS-V3-plasma-0_Cluster_8165_sequences=18	0	18
SDS-V3-plasma-0_Cluster_1415_sequences=18	0	18
SDS-V3-plasma-0_Cluster_5614_sequences=18	0	18
SDS-V3-plasma-0_Cluster_4781_sequences=18	0	18
SDS-V3-plasma-0_Cluster_2363_sequences=18	0	18
SDS-V3-plasma-0_Cluster_7364_sequences=18	0	18
SDS-V3-plasma-0_Cluster_8205_sequences=18	0	18
SDS-V3-plasma-0_Cluster_644_sequences=18	0	18
SDS-V3-plasma-0_Cluster_2069_sequences=18	0	18
SDS-V3-plasma-0_Cluster_2189_sequences=18	0	18
SDS-V3-plasma-0_Cluster_2235_sequences=18	0	18
SDS-V3-plasma-0_Cluster_2838_sequences=18	0	18
SDS-V3-plasma-0_Cluster_3269_sequences=18	0	18
SDS-V3-plasma-0_Cluster_4077_sequences=18	0	18

SDS-V3-plasma-0_Cluster_4735_sequences=18	0	18
SDS-V3-plasma-0_Cluster_5512_sequences=18	0	18
SDS-V3-plasma-0_Cluster_565_sequences=18	0	18
SDS-V3-plasma-0_Cluster_1198_sequences=18	0	18
SDS-V3-plasma-0_Cluster_1792_sequences=18	0	18
SDS-V3-plasma-0_Cluster_1450_sequences=18	0	18
SDS-V3-plasma-0_Cluster_4340_sequences=18	0	18
SDS-V3-plasma-0_Cluster_1074_sequences=18	0	18
SDS-V3-plasma-0_Cluster_2051_sequences=18	0	18
SDS-V3-plasma-0_Cluster_2018_sequences=18	0	18
SDS-V3-plasma-0_Cluster_455_sequences=18	0	18
SDS-V3-plasma-0_Cluster_2406_sequences=18	0	18
SDS-V3-plasma-0_Cluster_3560_sequences=18	0	18
SDS-V3-plasma-5_Cluster_58_sequences=18	9	18
SDS-V3-plasma-5_Cluster_738_sequences=18	9	18
SDS-V3-plasma-5_Cluster_322_sequences=18	9	18
SDS-V3-plasma-5_Cluster_104_sequences=18	9	18
SDS-V3-plasma-5_Cluster_45_sequences=18	9	18
SDS-V3-plasma-7_Cluster_234_sequences=18	14	18
SDS-V3-plasma-7_Cluster_482_sequences=18	14	18
SDS-V3-plasma-7_Cluster_1061_sequences=18	14	18
SDS-V3-plasma-7_Cluster_2003_sequences=18	14	18
SDS-V3-plasma-7_Cluster_2648_sequences=18	14	18
SDS-V3-plasma-7_Cluster_2582_sequences=18	14	18
SDS-V3-plasma-8_Cluster_303_sequences=18	16	18
SDS-V3-plasma-8_Cluster_3365_sequences=18	16	18
SDS-V3-plasma-8_Cluster_2449_sequences=18	16	18
SDS-V3-plasma-8_Cluster_3460_sequences=18	16	18
SDS-V3-plasma-8_Cluster_2360_sequences=18	16	18
SDS-V3-plasma-8_Cluster_4024_sequences=18	16	18
SDS-V3-plasma-8_Cluster_2610_sequences=18	16	18
SDS-V3-plasma-8_Cluster_2684_sequences=18	16	18
SDS-V3-plasma-8_Cluster_2971_sequences=18	16	18
SDS-V3-plasma-8_Cluster_3115_sequences=18	16	18
SDS-V3-plasma-8_Cluster_4585_sequences=18	16	18
SDS-V3-plasma-8_Cluster_2766_sequences=18	16	18
SDS-V3-plasma-8_Cluster_3196_sequences=18	16	18
SDS-V3-plasma-8_Cluster_3257_sequences=18	16	18
SDS-V3-plasma-8_Cluster_3907_sequences=18	16	18
SDS-V3-plasma-8_Cluster_3267_sequences=18	16	18
SDS-V3-plasma-8_Cluster_5519_sequences=18	16	18
SDS-V3-plasma-24_Cluster_1131_sequences=18	124	18
SDS-V3-plasma-24_Cluster_1877_sequences=18	124	18
SDS-V3-plasma-24_Cluster_2012_sequences=18	124	18
SDS-V3-plasma-24_Cluster_2289_sequences=18	124	18

SDS-V3-plasma-24_Cluster_33_sequences=18	124	18
SDS-V3-plasma-24_Cluster_493_sequences=18	124	18
SDS-V3-plasma-24_Cluster_669_sequences=18	124	18
SDS-V3-plasma-24_Cluster_919_sequences=18	124	18
SDS-V3-plasma-24_Cluster_929_sequences=18	124	18
SDS-V3-plasma-24_Cluster_1839_sequences=18	124	18
SDS-V3-plasma-24_Cluster_603_sequences=18	124	18
SDS-V3-plasma-24_Cluster_293_sequences=18	124	18
SDS-V3-plasma-24_Cluster_1106_sequences=18	124	18
SDS-V3-plasma-24_Cluster_2191_sequences=18	124	18
SDS-V3-plasma-24_Cluster_2328_sequences=18	124	18
SDS-V3-plasma-24_Cluster_854_sequences=18	124	18
SDS-V3-plasma-27_Cluster_1123_sequences=18	131	18
SDS-V3-plasma-27_Cluster_206_sequences=18	131	18
SDS-V3-plasma-27_Cluster_287_sequences=18	131	18
SDS-V3-plasma-27_Cluster_391_sequences=18	131	18
SDS-V3-plasma-27_Cluster_4579_sequences=18	131	18
SDS-V3-plasma-27_Cluster_826_sequences=18	131	18
SDS-V3-plasma-27_Cluster_1354_sequences=18	131	18
SDS-V3-plasma-27_Cluster_994_sequences=18	131	18
SDS-V3-plasma-27_Cluster_1459_sequences=18	131	18
SDS-V3-plasma-27_Cluster_951_sequences=18	131	18
SDS-V3-plasma-27_Cluster_1785_sequences=18	131	18
SDS-V3-plasma-27_Cluster_258_sequences=18	131	18
SDS-V3-plasma-27_Cluster_571_sequences=18	131	18
SDS-V3-plasma-27_Cluster_1166_sequences=18	131	18
SDS-V3-plasma-27_Cluster_226_sequences=18	131	18
SDS-V3-plasma-27_Cluster_3629_sequences=18	131	18
SDS-V3-plasma-27_Cluster_1253_sequences=18	131	18
SDS-V3-plasma-27_Cluster_1960_sequences=18	131	18
SDS-V3-plasma-27_Cluster_956_sequences=18	131	18
SDS-V3-plasma-45_Cluster_6001_sequences=18	282	18
SDS-V3-plasma-45_Cluster_8057_sequences=18	282	18
SDS-V3-plasma-45_Cluster_5429_sequences=18	282	18
SDS-V3-plasma-45_Cluster_38290_sequences=18	282	18
SDS-V3-plasma-45_Cluster_16896_sequences=18	282	18
SDS-V3-plasma-45_Cluster_5619_sequences=18	282	18
SDS-V3-plasma-45_Cluster_11373_sequences=18	282	18
SDS-V3-plasma-45_Cluster_10608_sequences=18	282	18
SDS-V3-plasma-45_Cluster_12075_sequences=18	282	18
SDS-V3-plasma-45_Cluster_8912_sequences=18	282	18
SDS-V3-plasma-45_Cluster_4176_sequences=18	282	18
SDS-V3-plasma-45_Cluster_16190_sequences=18	282	18
SDS-V3-plasma-45_Cluster_6592_sequences=18	282	18
SDS-V3-plasma-45_Cluster_5807_sequences=18	282	18

SDS-V3-plasma-45_Cluster_12173_sequences=18	282	18
SDS-V3-plasma-45_Cluster_13324_sequences=18	282	18
SDS-V3-plasma-45_Cluster_13857_sequences=18	282	18
SDS-V3-plasma-45_Cluster_24705_sequences=18	282	18
SDS-V3-plasma-45_Cluster_3517_sequences=18	282	18
SDS-V3-plasma-45_Cluster_4211_sequences=18	282	18
SDS-V3-plasma-45_Cluster_5739_sequences=18	282	18
SDS-V3-plasma-45_Cluster_6208_sequences=18	282	18
SDS-V3-plasma-45_Cluster_4712_sequences=18	282	18
SDS-V3-plasma-45_Cluster_15762_sequences=18	282	18
SDS-V3-plasma-45_Cluster_18894_sequences=18	282	18
SDS-V3-plasma-45_Cluster_19417_sequences=18	282	18
SDS-V3-plasma-45_Cluster_14243_sequences=18	282	18
SDS-V3-plasma-45_Cluster_8891_sequences=18	282	18
SDS-V3-plasma-45_Cluster_7758_sequences=18	282	18
SDS-V3-plasma-45_Cluster_10393_sequences=18	282	18
SDS-V3-plasma-45_Cluster_10561_sequences=18	282	18
SDS-V3-plasma-45_Cluster_11126_sequences=18	282	18
SDS-V3-plasma-45_Cluster_13319_sequences=18	282	18
SDS-V3-plasma-45_Cluster_18323_sequences=18	282	18
SDS-V3-plasma-45_Cluster_5670_sequences=18	282	18
SDS-V3-plasma-45_Cluster_2293_sequences=18	282	18
SDS-V3-plasma-45_Cluster_2437_sequences=18	282	18
SDS-V3-plasma-45_Cluster_4698_sequences=18	282	18
SDS-V3-plasma-45_Cluster_7292_sequences=18	282	18
SDS-V3-plasma-45_Cluster_13687_sequences=18	282	18
SDS-V3-plasma-45_Cluster_14417_sequences=18	282	18
SDS-V3-plasma-45_Cluster_7541_sequences=18	282	18
SDS-V3-plasma-45_Cluster_4540_sequences=18	282	18
SDS-V3-plasma-45_Cluster_25231_sequences=18	282	18
SDS-V3-plasma-45_Cluster_9820_sequences=18	282	18
SDS-V3-plasma-45_Cluster_3653_sequences=18	282	18
SDS-V3-plasma-45_Cluster_16929_sequences=18	282	18
SDS-V3-plasma-45_Cluster_3123_sequences=18	282	18
SDS-V3-plasma-45_Cluster_21914_sequences=18	282	18
SDS-V3-plasma-45_Cluster_14395_sequences=18	282	18
SDS-V3-plasma-45_Cluster_8765_sequences=18	282	18
SDS-V3-plasma-45_Cluster_7309_sequences=18	282	18
SDS-V3-plasma-45_Cluster_4116_sequences=18	282	18
SDS-V3-plasma-45_Cluster_17275_sequences=18	282	18
SDS-V3-plasma-45_Cluster_14799_sequences=18	282	18
SDS-V3-plasma-45_Cluster_14103_sequences=18	282	18
SDS-V3-plasma-45_Cluster_22758_sequences=18	282	18
SDS-V3-plasma-45_Cluster_5302_sequences=18	282	18
SDS-V3-plasma-45_Cluster_1540_sequences=18	282	18

SDS-V3-plasma-45_Cluster_14206_sequences=18	282	18
SDS-V3-plasma-45_Cluster_1146_sequences=18	282	18
SDS-V3-plasma-45_Cluster_8616_sequences=18	282	18
SDS-V3-plasma-45_Cluster_13587_sequences=18	282	18
SDS-V3-plasma-45_Cluster_5327_sequences=18	282	18
SDS-V3-plasma-45_Cluster_2041_sequences=18	282	18
SDS-V3-plasma-45_Cluster_8050_sequences=18	282	18
SDS-V3-plasma-45_Cluster_29206_sequences=18	282	18
SDS-V3-plasma-45_Cluster_19787_sequences=18	282	18
SDS-V3-plasma-45_Cluster_2702_sequences=18	282	18
SDS-V3-plasma-45_Cluster_5368_sequences=18	282	18
SDS-V3-plasma-45_Cluster_6383_sequences=18	282	18
SDS-V3-plasma-45_Cluster_2464_sequences=18	282	18
SDS-V3-plasma-45_Cluster_19077_sequences=18	282	18
SDS-V3-plasma-45_Cluster_12800_sequences=18	282	18
SDS-V3-plasma-45_Cluster_17193_sequences=18	282	18
SDS-V3-plasma-45_Cluster_8260_sequences=18	282	18
SDS-V3-plasma-45_Cluster_107_sequences=18	282	18
SDS-V3-plasma-45_Cluster_1778_sequences=18	282	18
SDS-V3-plasma-45_Cluster_16969_sequences=18	282	18
SDS-V3-plasma-45_Cluster_12961_sequences=18	282	18
SDS-V3-plasma-45_Cluster_11000_sequences=18	282	18
SDS-V3-plasma-45_Cluster_8369_sequences=18	282	18
SDS-V3-plasma-45_Cluster_9044_sequences=18	282	18
SDS-V3-plasma-45_Cluster_3785_sequences=18	282	18
SDS-V3-plasma-45_Cluster_509_sequences=18	282	18
SDS-V3-plasma-45_Cluster_4224_sequences=18	282	18
SDS-V3-plasma-45_Cluster_6713_sequences=18	282	18
SDS-V3-plasma-45_Cluster_976_sequences=18	282	18
SDS-V3-plasma-45_Cluster_155_sequences=18	282	18
SDS-V3-plasma-45_Cluster_8669_sequences=18	282	18
SDS-V3-plasma-45_Cluster_5492_sequences=18	282	18
SDS-V3-plasma-45_Cluster_44918_sequences=18	282	18
SDS-V3-plasma-45_Cluster_5461_sequences=18	282	18
SDS-V3-plasma-45_Cluster_12325_sequences=18	282	18
SDS-V3-plasma-45_Cluster_23230_sequences=18	282	18
SDS-V3-plasma-45_Cluster_12175_sequences=18	282	18
SDS-V3-plasma-45_Cluster_7294_sequences=18	282	18
SDS-V3-plasma-45_Cluster_798_sequences=18	282	18
SDS-V3-plasma-45_Cluster_9176_sequences=18	282	18
SDS-V3-plasma-45_Cluster_5635_sequences=18	282	18
SDS-V3-plasma-45_Cluster_3015_sequences=18	282	18
SDS-V3-plasma-45_Cluster_66690_sequences=18	282	18
SDS-V3-plasma-46_Cluster_2033_sequences=18	286	18
SDS-V3-plasma-46_Cluster_7404_sequences=18	286	18

SDS-V3-plasma-46_Cluster_7531_sequences=18	286	18
SDS-V3-plasma-46_Cluster_3535_sequences=18	286	18
SDS-V3-plasma-46_Cluster_2361_sequences=18	286	18
SDS-V3-plasma-46_Cluster_491_sequences=18	286	18
SDS-V3-plasma-46_Cluster_1083_sequences=18	286	18
SDS-V3-plasma-46_Cluster_8465_sequences=18	286	18
SDS-V3-plasma-46_Cluster_7822_sequences=18	286	18
SDS-V3-plasma-46_Cluster_2225_sequences=18	286	18
SDS-V3-plasma-46_Cluster_3892_sequences=18	286	18
SDS-V3-plasma-46_Cluster_2404_sequences=18	286	18
SDS-V3-plasma-46_Cluster_1148_sequences=18	286	18
SDS-V3-plasma-46_Cluster_1632_sequences=18	286	18
SDS-V3-plasma-46_Cluster_2147_sequences=18	286	18
SDS-V3-plasma-46_Cluster_2334_sequences=18	286	18
SDS-V3-plasma-46_Cluster_3050_sequences=18	286	18
SDS-V3-plasma-46_Cluster_4505_sequences=18	286	18
SDS-V3-plasma-46_Cluster_4603_sequences=18	286	18
SDS-V3-plasma-46_Cluster_4680_sequences=18	286	18
SDS-V3-plasma-46_Cluster_706_sequences=18	286	18
SDS-V3-plasma-46_Cluster_8223_sequences=18	286	18
SDS-V3-plasma-46_Cluster_9516_sequences=18	286	18
SDS-V3-plasma-46_Cluster_1210_sequences=18	286	18
SDS-V3-plasma-46_Cluster_1761_sequences=18	286	18
SDS-V3-plasma-46_Cluster_4445_sequences=18	286	18
SDS-V3-plasma-46_Cluster_2221_sequences=18	286	18
SDS-V3-plasma-46_Cluster_679_sequences=18	286	18
SDS-V3-plasma-46_Cluster_5337_sequences=18	286	18
SDS-V3-plasma-46_Cluster_2585_sequences=18	286	18
SDS-V3-plasma-46_Cluster_4879_sequences=18	286	18
SDS-V3-plasma-46_Cluster_4236_sequences=18	286	18
SDS-V3-plasma-46_Cluster_1058_sequences=18	286	18
SDS-V3-plasma-46_Cluster_4820_sequences=18	286	18
SDS-V3-plasma-46_Cluster_2183_sequences=18	286	18
SDS-V3-plasma-46_Cluster_2198_sequences=18	286	18
SDS-V3-plasma-67_Cluster_5784_sequences=18	504	18
SDS-V3-plasma-67_Cluster_11022_sequences=18	504	18
SDS-V3-plasma-67_Cluster_16004_sequences=18	504	18
SDS-V3-plasma-67_Cluster_6338_sequences=18	504	18
SDS-V3-plasma-67_Cluster_9419_sequences=18	504	18
SDS-V3-plasma-67_Cluster_8231_sequences=18	504	18
SDS-V3-plasma-67_Cluster_43335_sequences=18	504	18
SDS-V3-plasma-67_Cluster_15719_sequences=18	504	18
SDS-V3-plasma-67_Cluster_14847_sequences=18	504	18
SDS-V3-plasma-67_Cluster_7478_sequences=18	504	18
SDS-V3-plasma-67_Cluster_4972_sequences=18	504	18

SDS-V3-plasma-67_Cluster_6232_sequences=18	504	18
SDS-V3-plasma-67_Cluster_360_sequences=18	504	18
SDS-V3-plasma-67_Cluster_9295_sequences=18	504	18
SDS-V3-plasma-67_Cluster_8328_sequences=18	504	18
SDS-V3-plasma-67_Cluster_4698_sequences=18	504	18
SDS-V3-plasma-67_Cluster_21779_sequences=18	504	18
SDS-V3-plasma-67_Cluster_12349_sequences=18	504	18
SDS-V3-plasma-67_Cluster_4145_sequences=18	504	18
SDS-V3-plasma-67_Cluster_7106_sequences=18	504	18
SDS-V3-plasma-67_Cluster_12303_sequences=18	504	18
SDS-V3-plasma-67_Cluster_16840_sequences=18	504	18
SDS-V3-plasma-67_Cluster_17376_sequences=18	504	18
SDS-V3-plasma-67_Cluster_23463_sequences=18	504	18
SDS-V3-plasma-67_Cluster_3251_sequences=18	504	18
SDS-V3-plasma-67_Cluster_3537_sequences=18	504	18
SDS-V3-plasma-67_Cluster_3885_sequences=18	504	18
SDS-V3-plasma-67_Cluster_3973_sequences=18	504	18
SDS-V3-plasma-67_Cluster_51184_sequences=18	504	18
SDS-V3-plasma-67_Cluster_6563_sequences=18	504	18
SDS-V3-plasma-67_Cluster_7575_sequences=18	504	18
SDS-V3-plasma-67_Cluster_7828_sequences=18	504	18
SDS-V3-plasma-67_Cluster_78_sequences=18	504	18
SDS-V3-plasma-67_Cluster_7414_sequences=18	504	18
SDS-V3-plasma-67_Cluster_12275_sequences=18	504	18
SDS-V3-plasma-67_Cluster_13442_sequences=18	504	18
SDS-V3-plasma-67_Cluster_5649_sequences=18	504	18
SDS-V3-plasma-67_Cluster_13578_sequences=18	504	18
SDS-V3-plasma-67_Cluster_8395_sequences=18	504	18
SDS-V3-plasma-67_Cluster_30416_sequences=18	504	18
SDS-V3-plasma-67_Cluster_2612_sequences=18	504	18
SDS-V3-plasma-67_Cluster_9570_sequences=18	504	18
SDS-V3-plasma-67_Cluster_7059_sequences=18	504	18
SDS-V3-plasma-67_Cluster_10836_sequences=18	504	18
SDS-V3-plasma-67_Cluster_8931_sequences=18	504	18
SDS-V3-plasma-67_Cluster_23901_sequences=18	504	18
SDS-V3-plasma-67_Cluster_15448_sequences=18	504	18
SDS-V3-plasma-67_Cluster_15384_sequences=18	504	18
SDS-V3-plasma-67_Cluster_12904_sequences=18	504	18
SDS-V3-plasma-67_Cluster_3183_sequences=18	504	18
SDS-V3-plasma-67_Cluster_11623_sequences=18	504	18
SDS-V3-plasma-67_Cluster_76631_sequences=18	504	18
SDS-V3-plasma-67_Cluster_9209_sequences=18	504	18
SDS-V3-plasma-67_Cluster_9459_sequences=18	504	18
SDS-V3-plasma-67_Cluster_11378_sequences=18	504	18
SDS-V3-plasma-67_Cluster_24700_sequences=18	504	18

SDS-V3-PBMC-63_Cluster_1209_sequences=17	489	17
SDS-V3-plasma-0_Cluster_2133_sequences=17	0	17
SDS-V3-plasma-0_Cluster_1662_sequences=17	0	17
SDS-V3-plasma-0_Cluster_1582_sequences=17	0	17
SDS-V3-plasma-0_Cluster_1889_sequences=17	0	17
SDS-V3-plasma-0_Cluster_11652_sequences=17	0	17
SDS-V3-plasma-0_Cluster_1237_sequences=17	0	17
SDS-V3-plasma-0_Cluster_2063_sequences=17	0	17
SDS-V3-plasma-0_Cluster_2215_sequences=17	0	17
SDS-V3-plasma-0_Cluster_2271_sequences=17	0	17
SDS-V3-plasma-0_Cluster_258_sequences=17	0	17
SDS-V3-plasma-0_Cluster_3633_sequences=17	0	17
SDS-V3-plasma-0_Cluster_3751_sequences=17	0	17
SDS-V3-plasma-0_Cluster_390_sequences=17	0	17
SDS-V3-plasma-0_Cluster_4962_sequences=17	0	17
SDS-V3-plasma-0_Cluster_5206_sequences=17	0	17
SDS-V3-plasma-0_Cluster_6031_sequences=17	0	17
SDS-V3-plasma-0_Cluster_6120_sequences=17	0	17
SDS-V3-plasma-0_Cluster_827_sequences=17	0	17
SDS-V3-plasma-0_Cluster_841_sequences=17	0	17
SDS-V3-plasma-0_Cluster_1021_sequences=17	0	17
SDS-V3-plasma-0_Cluster_2034_sequences=17	0	17
SDS-V3-plasma-0_Cluster_25180_sequences=17	0	17
SDS-V3-plasma-0_Cluster_13342_sequences=17	0	17
SDS-V3-plasma-0_Cluster_3385_sequences=17	0	17
SDS-V3-plasma-0_Cluster_154_sequences=17	0	17
SDS-V3-plasma-0_Cluster_4323_sequences=17	0	17
SDS-V3-plasma-0_Cluster_1475_sequences=17	0	17
SDS-V3-plasma-0_Cluster_10183_sequences=17	0	17
SDS-V3-plasma-0_Cluster_1510_sequences=17	0	17
SDS-V3-plasma-0_Cluster_2987_sequences=17	0	17
SDS-V3-plasma-0_Cluster_8621_sequences=17	0	17
SDS-V3-plasma-0_Cluster_1454_sequences=17	0	17
SDS-V3-plasma-0_Cluster_915_sequences=17	0	17
SDS-V3-plasma-0_Cluster_738_sequences=17	0	17
SDS-V3-plasma-0_Cluster_695_sequences=17	0	17
SDS-V3-plasma-0_Cluster_7082_sequences=17	0	17
SDS-V3-plasma-0_Cluster_780_sequences=17	0	17
SDS-V3-plasma-0_Cluster_4692_sequences=17	0	17
SDS-V3-plasma-0_Cluster_1048_sequences=17	0	17
SDS-V3-plasma-0_Cluster_2547_sequences=17	0	17
SDS-V3-plasma-0_Cluster_2841_sequences=17	0	17
SDS-V3-plasma-0_Cluster_4477_sequences=17	0	17
SDS-V3-plasma-0_Cluster_5399_sequences=17	0	17
SDS-V3-plasma-0_Cluster_5989_sequences=17	0	17

SDS-V3-plasma-0_Cluster_12221_sequences=17	0	17
SDS-V3-plasma-0_Cluster_3786_sequences=17	0	17
SDS-V3-plasma-0_Cluster_399_sequences=17	0	17
SDS-V3-plasma-0_Cluster_4442_sequences=17	0	17
SDS-V3-plasma-0_Cluster_3044_sequences=17	0	17
SDS-V3-plasma-0_Cluster_1364_sequences=17	0	17
SDS-V3-plasma-0_Cluster_1388_sequences=17	0	17
SDS-V3-plasma-0_Cluster_4918_sequences=17	0	17
SDS-V3-plasma-0_Cluster_687_sequences=17	0	17
SDS-V3-plasma-5_Cluster_150_sequences=17	9	17
SDS-V3-plasma-5_Cluster_378_sequences=17	9	17
SDS-V3-plasma-5_Cluster_507_sequences=17	9	17
SDS-V3-plasma-5_Cluster_615_sequences=17	9	17
SDS-V3-plasma-5_Cluster_173_sequences=17	9	17
SDS-V3-plasma-5_Cluster_208_sequences=17	9	17
SDS-V3-plasma-5_Cluster_329_sequences=17	9	17
SDS-V3-plasma-5_Cluster_552_sequences=17	9	17
SDS-V3-plasma-5_Cluster_56_sequences=17	9	17
SDS-V3-plasma-5_Cluster_701_sequences=17	9	17
SDS-V3-plasma-5_Cluster_779_sequences=17	9	17
SDS-V3-plasma-7_Cluster_1457_sequences=17	14	17
SDS-V3-plasma-7_Cluster_691_sequences=17	14	17
SDS-V3-plasma-7_Cluster_124_sequences=17	14	17
SDS-V3-plasma-7_Cluster_1790_sequences=17	14	17
SDS-V3-plasma-7_Cluster_285_sequences=17	14	17
SDS-V3-plasma-7_Cluster_3185_sequences=17	14	17
SDS-V3-plasma-7_Cluster_4087_sequences=17	14	17
SDS-V3-plasma-7_Cluster_1864_sequences=17	14	17
SDS-V3-plasma-7_Cluster_2533_sequences=17	14	17
SDS-V3-plasma-7_Cluster_1054_sequences=17	14	17
SDS-V3-plasma-7_Cluster_8021_sequences=17	14	17
SDS-V3-plasma-7_Cluster_980_sequences=17	14	17
SDS-V3-plasma-7_Cluster_7899_sequences=17	14	17
SDS-V3-plasma-7_Cluster_1393_sequences=17	14	17
SDS-V3-plasma-7_Cluster_1722_sequences=17	14	17
SDS-V3-plasma-8_Cluster_158_sequences=17	16	17
SDS-V3-plasma-8_Cluster_292_sequences=17	16	17
SDS-V3-plasma-8_Cluster_4205_sequences=17	16	17
SDS-V3-plasma-8_Cluster_2946_sequences=17	16	17
SDS-V3-plasma-8_Cluster_2531_sequences=17	16	17
SDS-V3-plasma-8_Cluster_4746_sequences=17	16	17
SDS-V3-plasma-8_Cluster_2504_sequences=17	16	17
SDS-V3-plasma-8_Cluster_6143_sequences=17	16	17
SDS-V3-plasma-8_Cluster_4645_sequences=17	16	17
SDS-V3-plasma-24_Cluster_1028_sequences=17	124	17

SDS-V3-plasma-24_Cluster_1267_sequences=17	124	17
SDS-V3-plasma-24_Cluster_1792_sequences=17	124	17
SDS-V3-plasma-24_Cluster_27_sequences=17	124	17
SDS-V3-plasma-24_Cluster_3189_sequences=17	124	17
SDS-V3-plasma-24_Cluster_3897_sequences=17	124	17
SDS-V3-plasma-24_Cluster_593_sequences=17	124	17
SDS-V3-plasma-24_Cluster_599_sequences=17	124	17
SDS-V3-plasma-24_Cluster_59_sequences=17	124	17
SDS-V3-plasma-24_Cluster_768_sequences=17	124	17
SDS-V3-plasma-24_Cluster_771_sequences=17	124	17
SDS-V3-plasma-24_Cluster_528_sequences=17	124	17
SDS-V3-plasma-24_Cluster_2380_sequences=17	124	17
SDS-V3-plasma-24_Cluster_1835_sequences=17	124	17
SDS-V3-plasma-24_Cluster_3706_sequences=17	124	17
SDS-V3-plasma-24_Cluster_464_sequences=17	124	17
SDS-V3-plasma-24_Cluster_3238_sequences=17	124	17
SDS-V3-plasma-24_Cluster_101_sequences=17	124	17
SDS-V3-plasma-24_Cluster_985_sequences=17	124	17
SDS-V3-plasma-24_Cluster_2465_sequences=17	124	17
SDS-V3-plasma-24_Cluster_311_sequences=17	124	17
SDS-V3-plasma-24_Cluster_533_sequences=17	124	17
SDS-V3-plasma-27_Cluster_2109_sequences=17	131	17
SDS-V3-plasma-27_Cluster_2365_sequences=17	131	17
SDS-V3-plasma-27_Cluster_2424_sequences=17	131	17
SDS-V3-plasma-27_Cluster_734_sequences=17	131	17
SDS-V3-plasma-27_Cluster_1181_sequences=17	131	17
SDS-V3-plasma-27_Cluster_2361_sequences=17	131	17
SDS-V3-plasma-27_Cluster_1092_sequences=17	131	17
SDS-V3-plasma-27_Cluster_3438_sequences=17	131	17
SDS-V3-plasma-27_Cluster_465_sequences=17	131	17
SDS-V3-plasma-27_Cluster_1507_sequences=17	131	17
SDS-V3-plasma-27_Cluster_1575_sequences=17	131	17
SDS-V3-plasma-27_Cluster_1072_sequences=17	131	17
SDS-V3-plasma-27_Cluster_2829_sequences=17	131	17
SDS-V3-plasma-27_Cluster_2208_sequences=17	131	17
SDS-V3-plasma-27_Cluster_1238_sequences=17	131	17
SDS-V3-plasma-27_Cluster_559_sequences=17	131	17
SDS-V3-plasma-27_Cluster_1310_sequences=17	131	17
SDS-V3-plasma-27_Cluster_2474_sequences=17	131	17
SDS-V3-plasma-27_Cluster_1566_sequences=17	131	17
SDS-V3-plasma-27_Cluster_1764_sequences=17	131	17
SDS-V3-plasma-45_Cluster_11775_sequences=17	282	17
SDS-V3-plasma-45_Cluster_17517_sequences=17	282	17
SDS-V3-plasma-45_Cluster_1776_sequences=17	282	17
SDS-V3-plasma-45_Cluster_1910_sequences=17	282	17

SDS-V3-plasma-45_Cluster_4229_sequences=17	282	17
SDS-V3-plasma-45_Cluster_12368_sequences=17	282	17
SDS-V3-plasma-45_Cluster_26693_sequences=17	282	17
SDS-V3-plasma-45_Cluster_6983_sequences=17	282	17
SDS-V3-plasma-45_Cluster_9379_sequences=17	282	17
SDS-V3-plasma-45_Cluster_66_sequences=17	282	17
SDS-V3-plasma-45_Cluster_6833_sequences=17	282	17
SDS-V3-plasma-45_Cluster_20957_sequences=17	282	17
SDS-V3-plasma-45_Cluster_22684_sequences=17	282	17
SDS-V3-plasma-45_Cluster_15142_sequences=17	282	17
SDS-V3-plasma-45_Cluster_7750_sequences=17	282	17
SDS-V3-plasma-45_Cluster_19767_sequences=17	282	17
SDS-V3-plasma-45_Cluster_6637_sequences=17	282	17
SDS-V3-plasma-45_Cluster_22888_sequences=17	282	17
SDS-V3-plasma-45_Cluster_14274_sequences=17	282	17
SDS-V3-plasma-45_Cluster_11802_sequences=17	282	17
SDS-V3-plasma-45_Cluster_12279_sequences=17	282	17
SDS-V3-plasma-45_Cluster_12816_sequences=17	282	17
SDS-V3-plasma-45_Cluster_13717_sequences=17	282	17
SDS-V3-plasma-45_Cluster_14825_sequences=17	282	17
SDS-V3-plasma-45_Cluster_1850_sequences=17	282	17
SDS-V3-plasma-45_Cluster_2052_sequences=17	282	17
SDS-V3-plasma-45_Cluster_6144_sequences=17	282	17
SDS-V3-plasma-45_Cluster_7254_sequences=17	282	17
SDS-V3-plasma-45_Cluster_741_sequences=17	282	17
SDS-V3-plasma-45_Cluster_7505_sequences=17	282	17
SDS-V3-plasma-45_Cluster_9264_sequences=17	282	17
SDS-V3-plasma-45_Cluster_10212_sequences=17	282	17
SDS-V3-plasma-45_Cluster_15112_sequences=17	282	17
SDS-V3-plasma-45_Cluster_24628_sequences=17	282	17
SDS-V3-plasma-45_Cluster_4094_sequences=17	282	17
SDS-V3-plasma-45_Cluster_4099_sequences=17	282	17
SDS-V3-plasma-45_Cluster_4976_sequences=17	282	17
SDS-V3-plasma-45_Cluster_5770_sequences=17	282	17
SDS-V3-plasma-45_Cluster_6652_sequences=17	282	17
SDS-V3-plasma-45_Cluster_14244_sequences=17	282	17
SDS-V3-plasma-45_Cluster_25240_sequences=17	282	17
SDS-V3-plasma-45_Cluster_4397_sequences=17	282	17
SDS-V3-plasma-45_Cluster_10594_sequences=17	282	17
SDS-V3-plasma-45_Cluster_10868_sequences=17	282	17
SDS-V3-plasma-45_Cluster_4063_sequences=17	282	17
SDS-V3-plasma-45_Cluster_1264_sequences=17	282	17
SDS-V3-plasma-45_Cluster_12820_sequences=17	282	17
SDS-V3-plasma-45_Cluster_9756_sequences=17	282	17
SDS-V3-plasma-45_Cluster_832_sequences=17	282	17

SDS-V3-plasma-45_Cluster_9514_sequences=17	282	17
SDS-V3-plasma-45_Cluster_4644_sequences=17	282	17
SDS-V3-plasma-45_Cluster_4642_sequences=17	282	17
SDS-V3-plasma-45_Cluster_8278_sequences=17	282	17
SDS-V3-plasma-45_Cluster_3860_sequences=17	282	17
SDS-V3-plasma-45_Cluster_2300_sequences=17	282	17
SDS-V3-plasma-45_Cluster_27049_sequences=17	282	17
SDS-V3-plasma-45_Cluster_2796_sequences=17	282	17
SDS-V3-plasma-45_Cluster_6826_sequences=17	282	17
SDS-V3-plasma-45_Cluster_8502_sequences=17	282	17
SDS-V3-plasma-45_Cluster_11489_sequences=17	282	17
SDS-V3-plasma-45_Cluster_5928_sequences=17	282	17
SDS-V3-plasma-45_Cluster_15936_sequences=17	282	17
SDS-V3-plasma-45_Cluster_4768_sequences=17	282	17
SDS-V3-plasma-45_Cluster_2339_sequences=17	282	17
SDS-V3-plasma-45_Cluster_2548_sequences=17	282	17
SDS-V3-plasma-45_Cluster_15005_sequences=17	282	17
SDS-V3-plasma-45_Cluster_20481_sequences=17	282	17
SDS-V3-plasma-45_Cluster_4853_sequences=17	282	17
SDS-V3-plasma-45_Cluster_21897_sequences=17	282	17
SDS-V3-plasma-45_Cluster_19843_sequences=17	282	17
SDS-V3-plasma-45_Cluster_27221_sequences=17	282	17
SDS-V3-plasma-45_Cluster_5787_sequences=17	282	17
SDS-V3-plasma-45_Cluster_21776_sequences=17	282	17
SDS-V3-plasma-45_Cluster_1011_sequences=17	282	17
SDS-V3-plasma-45_Cluster_16346_sequences=17	282	17
SDS-V3-plasma-45_Cluster_5952_sequences=17	282	17
SDS-V3-plasma-45_Cluster_3813_sequences=17	282	17
SDS-V3-plasma-45_Cluster_66623_sequences=17	282	17
SDS-V3-plasma-45_Cluster_4521_sequences=17	282	17
SDS-V3-plasma-45_Cluster_5831_sequences=17	282	17
SDS-V3-plasma-45_Cluster_22874_sequences=17	282	17
SDS-V3-plasma-45_Cluster_3212_sequences=17	282	17
SDS-V3-plasma-45_Cluster_15717_sequences=17	282	17
SDS-V3-plasma-45_Cluster_5095_sequences=17	282	17
SDS-V3-plasma-45_Cluster_651_sequences=17	282	17
SDS-V3-plasma-45_Cluster_8379_sequences=17	282	17
SDS-V3-plasma-45_Cluster_27430_sequences=17	282	17
SDS-V3-plasma-45_Cluster_13732_sequences=17	282	17
SDS-V3-plasma-45_Cluster_5239_sequences=17	282	17
SDS-V3-plasma-45_Cluster_1890_sequences=17	282	17
SDS-V3-plasma-45_Cluster_8554_sequences=17	282	17
SDS-V3-plasma-45_Cluster_33572_sequences=17	282	17
SDS-V3-plasma-45_Cluster_3905_sequences=17	282	17
SDS-V3-plasma-45_Cluster_796_sequences=17	282	17

SDS-V3-plasma-45_Cluster_7878_sequences=17	282	17
SDS-V3-plasma-45_Cluster_6665_sequences=17	282	17
SDS-V3-plasma-45_Cluster_4689_sequences=17	282	17
SDS-V3-plasma-45_Cluster_2559_sequences=17	282	17
SDS-V3-plasma-45_Cluster_6875_sequences=17	282	17
SDS-V3-plasma-45_Cluster_838_sequences=17	282	17
SDS-V3-plasma-45_Cluster_5227_sequences=17	282	17
SDS-V3-plasma-45_Cluster_4756_sequences=17	282	17
SDS-V3-plasma-45_Cluster_9758_sequences=17	282	17
SDS-V3-plasma-45_Cluster_3027_sequences=17	282	17
SDS-V3-plasma-45_Cluster_6310_sequences=17	282	17
SDS-V3-plasma-45_Cluster_22528_sequences=17	282	17
SDS-V3-plasma-45_Cluster_10750_sequences=17	282	17
SDS-V3-plasma-45_Cluster_4946_sequences=17	282	17
SDS-V3-plasma-45_Cluster_13185_sequences=17	282	17
SDS-V3-plasma-45_Cluster_16917_sequences=17	282	17
SDS-V3-plasma-45_Cluster_1986_sequences=17	282	17
SDS-V3-plasma-45_Cluster_9140_sequences=17	282	17
SDS-V3-plasma-45_Cluster_12920_sequences=17	282	17
SDS-V3-plasma-45_Cluster_6401_sequences=17	282	17
SDS-V3-plasma-45_Cluster_2560_sequences=17	282	17
SDS-V3-plasma-45_Cluster_3147_sequences=17	282	17
SDS-V3-plasma-45_Cluster_10243_sequences=17	282	17
SDS-V3-plasma-45_Cluster_12573_sequences=17	282	17
SDS-V3-plasma-45_Cluster_15286_sequences=17	282	17
SDS-V3-plasma-45_Cluster_224_sequences=17	282	17
SDS-V3-plasma-45_Cluster_24014_sequences=17	282	17
SDS-V3-plasma-45_Cluster_33691_sequences=17	282	17
SDS-V3-plasma-46_Cluster_1791_sequences=17	286	17
SDS-V3-plasma-46_Cluster_5932_sequences=17	286	17
SDS-V3-plasma-46_Cluster_8231_sequences=17	286	17
SDS-V3-plasma-46_Cluster_597_sequences=17	286	17
SDS-V3-plasma-46_Cluster_9351_sequences=17	286	17
SDS-V3-plasma-46_Cluster_3967_sequences=17	286	17
SDS-V3-plasma-46_Cluster_1975_sequences=17	286	17
SDS-V3-plasma-46_Cluster_8663_sequences=17	286	17
SDS-V3-plasma-46_Cluster_4877_sequences=17	286	17
SDS-V3-plasma-46_Cluster_15269_sequences=17	286	17
SDS-V3-plasma-46_Cluster_3326_sequences=17	286	17
SDS-V3-plasma-46_Cluster_7740_sequences=17	286	17
SDS-V3-plasma-46_Cluster_835_sequences=17	286	17
SDS-V3-plasma-46_Cluster_3215_sequences=17	286	17
SDS-V3-plasma-46_Cluster_1835_sequences=17	286	17
SDS-V3-plasma-46_Cluster_2475_sequences=17	286	17
SDS-V3-plasma-46_Cluster_948_sequences=17	286	17

SDS-V3-plasma-46_Cluster_7835_sequences=17	286	17
SDS-V3-plasma-46_Cluster_872_sequences=17	286	17
SDS-V3-plasma-46_Cluster_2984_sequences=17	286	17
SDS-V3-plasma-46_Cluster_9048_sequences=17	286	17
SDS-V3-plasma-46_Cluster_3619_sequences=17	286	17
SDS-V3-plasma-46_Cluster_4190_sequences=17	286	17
SDS-V3-plasma-46_Cluster_1331_sequences=17	286	17
SDS-V3-plasma-46_Cluster_11464_sequences=17	286	17
SDS-V3-plasma-46_Cluster_182_sequences=17	286	17
SDS-V3-plasma-46_Cluster_2403_sequences=17	286	17
SDS-V3-plasma-46_Cluster_2841_sequences=17	286	17
SDS-V3-plasma-46_Cluster_310_sequences=17	286	17
SDS-V3-plasma-46_Cluster_34_sequences=17	286	17
SDS-V3-plasma-46_Cluster_3748_sequences=17	286	17
SDS-V3-plasma-46_Cluster_3836_sequences=17	286	17
SDS-V3-plasma-46_Cluster_522_sequences=17	286	17
SDS-V3-plasma-46_Cluster_5854_sequences=17	286	17
SDS-V3-plasma-46_Cluster_7522_sequences=17	286	17
SDS-V3-plasma-46_Cluster_2458_sequences=17	286	17
SDS-V3-plasma-46_Cluster_3311_sequences=17	286	17
SDS-V3-plasma-46_Cluster_1580_sequences=17	286	17
SDS-V3-plasma-46_Cluster_1890_sequences=17	286	17
SDS-V3-plasma-46_Cluster_763_sequences=17	286	17
SDS-V3-plasma-46_Cluster_1090_sequences=17	286	17
SDS-V3-plasma-46_Cluster_2020_sequences=17	286	17
SDS-V3-plasma-46_Cluster_5189_sequences=17	286	17
SDS-V3-plasma-46_Cluster_299_sequences=17	286	17
SDS-V3-plasma-46_Cluster_3561_sequences=17	286	17
SDS-V3-plasma-46_Cluster_4774_sequences=17	286	17
SDS-V3-plasma-46_Cluster_8342_sequences=17	286	17
SDS-V3-plasma-46_Cluster_187_sequences=17	286	17
SDS-V3-plasma-46_Cluster_2553_sequences=17	286	17
SDS-V3-plasma-46_Cluster_5755_sequences=17	286	17
SDS-V3-plasma-46_Cluster_3166_sequences=17	286	17
SDS-V3-plasma-67_Cluster_7529_sequences=17	504	17
SDS-V3-plasma-67_Cluster_16823_sequences=17	504	17
SDS-V3-plasma-67_Cluster_18560_sequences=17	504	17
SDS-V3-plasma-67_Cluster_18730_sequences=17	504	17
SDS-V3-plasma-67_Cluster_13395_sequences=17	504	17
SDS-V3-plasma-67_Cluster_5116_sequences=17	504	17
SDS-V3-plasma-67_Cluster_12058_sequences=17	504	17
SDS-V3-plasma-67_Cluster_18014_sequences=17	504	17
SDS-V3-plasma-67_Cluster_4229_sequences=17	504	17
SDS-V3-plasma-67_Cluster_2054_sequences=17	504	17
SDS-V3-plasma-67_Cluster_820_sequences=17	504	17

SDS-V3-plasma-67_Cluster_31765_sequences=17	504	17
SDS-V3-plasma-67_Cluster_14905_sequences=17	504	17
SDS-V3-plasma-67_Cluster_2466_sequences=17	504	17
SDS-V3-plasma-67_Cluster_19300_sequences=17	504	17
SDS-V3-plasma-67_Cluster_11398_sequences=17	504	17
SDS-V3-plasma-67_Cluster_5453_sequences=17	504	17
SDS-V3-plasma-67_Cluster_1496_sequences=17	504	17
SDS-V3-plasma-67_Cluster_4522_sequences=17	504	17
SDS-V3-plasma-67_Cluster_31920_sequences=17	504	17
SDS-V3-plasma-67_Cluster_7445_sequences=17	504	17
SDS-V3-plasma-67_Cluster_8987_sequences=17	504	17
SDS-V3-plasma-67_Cluster_14164_sequences=17	504	17
SDS-V3-plasma-67_Cluster_16574_sequences=17	504	17
SDS-V3-plasma-67_Cluster_1783_sequences=17	504	17
SDS-V3-plasma-67_Cluster_25064_sequences=17	504	17
SDS-V3-plasma-67_Cluster_31130_sequences=17	504	17
SDS-V3-plasma-67_Cluster_8109_sequences=17	504	17
SDS-V3-plasma-67_Cluster_9594_sequences=17	504	17
SDS-V3-plasma-67_Cluster_5630_sequences=17	504	17
SDS-V3-plasma-67_Cluster_4592_sequences=17	504	17
SDS-V3-plasma-67_Cluster_20931_sequences=17	504	17
SDS-V3-plasma-67_Cluster_3609_sequences=17	504	17
SDS-V3-plasma-67_Cluster_18553_sequences=17	504	17
SDS-V3-plasma-67_Cluster_103_sequences=17	504	17
SDS-V3-plasma-67_Cluster_587_sequences=17	504	17
SDS-V3-plasma-67_Cluster_10542_sequences=17	504	17
SDS-V3-plasma-67_Cluster_16363_sequences=17	504	17
SDS-V3-plasma-67_Cluster_2882_sequences=17	504	17
SDS-V3-plasma-67_Cluster_5910_sequences=17	504	17
SDS-V3-plasma-67_Cluster_13169_sequences=17	504	17
SDS-V3-plasma-67_Cluster_10471_sequences=17	504	17
SDS-V3-plasma-67_Cluster_15247_sequences=17	504	17
SDS-V3-plasma-67_Cluster_11951_sequences=17	504	17
SDS-V3-plasma-67_Cluster_75578_sequences=17	504	17
SDS-V3-plasma-67_Cluster_11306_sequences=17	504	17
SDS-V3-plasma-67_Cluster_13615_sequences=17	504	17
SDS-V3-plasma-67_Cluster_640_sequences=17	504	17
SDS-V3-plasma-67_Cluster_3249_sequences=17	504	17
SDS-V3-plasma-67_Cluster_75588_sequences=17	504	17
SDS-V3-plasma-67_Cluster_9756_sequences=17	504	17
SDS-V3-plasma-67_Cluster_601_sequences=17	504	17
SDS-V3-plasma-67_Cluster_2053_sequences=17	504	17
SDS-V3-plasma-67_Cluster_2304_sequences=17	504	17
SDS-V3-plasma-67_Cluster_3004_sequences=17	504	17
SDS-V3-plasma-67_Cluster_3967_sequences=17	504	17

SDS-V3-plasma-67_Cluster_5007_sequences=17	504	17
SDS-V3-PBMC-45_Cluster_29403_sequences=16	282	16
SDS-V3-plasma-0_Cluster_1695_sequences=16	0	16
SDS-V3-plasma-0_Cluster_1750_sequences=16	0	16
SDS-V3-plasma-0_Cluster_2548_sequences=16	0	16
SDS-V3-plasma-0_Cluster_5902_sequences=16	0	16
SDS-V3-plasma-0_Cluster_1105_sequences=16	0	16
SDS-V3-plasma-0_Cluster_1340_sequences=16	0	16
SDS-V3-plasma-0_Cluster_1609_sequences=16	0	16
SDS-V3-plasma-0_Cluster_1763_sequences=16	0	16
SDS-V3-plasma-0_Cluster_1879_sequences=16	0	16
SDS-V3-plasma-0_Cluster_2512_sequences=16	0	16
SDS-V3-plasma-0_Cluster_317_sequences=16	0	16
SDS-V3-plasma-0_Cluster_4276_sequences=16	0	16
SDS-V3-plasma-0_Cluster_4964_sequences=16	0	16
SDS-V3-plasma-0_Cluster_5531_sequences=16	0	16
SDS-V3-plasma-0_Cluster_7094_sequences=16	0	16
SDS-V3-plasma-0_Cluster_8091_sequences=16	0	16
SDS-V3-plasma-0_Cluster_9588_sequences=16	0	16
SDS-V3-plasma-0_Cluster_4666_sequences=16	0	16
SDS-V3-plasma-0_Cluster_2574_sequences=16	0	16
SDS-V3-plasma-0_Cluster_2541_sequences=16	0	16
SDS-V3-plasma-0_Cluster_2041_sequences=16	0	16
SDS-V3-plasma-0_Cluster_14193_sequences=16	0	16
SDS-V3-plasma-0_Cluster_3728_sequences=16	0	16
SDS-V3-plasma-0_Cluster_4072_sequences=16	0	16
SDS-V3-plasma-0_Cluster_3315_sequences=16	0	16
SDS-V3-plasma-0_Cluster_1057_sequences=16	0	16
SDS-V3-plasma-0_Cluster_1835_sequences=16	0	16
SDS-V3-plasma-0_Cluster_4113_sequences=16	0	16
SDS-V3-plasma-0_Cluster_7406_sequences=16	0	16
SDS-V3-plasma-0_Cluster_269_sequences=16	0	16
SDS-V3-plasma-0_Cluster_1045_sequences=16	0	16
SDS-V3-plasma-0_Cluster_1478_sequences=16	0	16
SDS-V3-plasma-0_Cluster_1998_sequences=16	0	16
SDS-V3-plasma-0_Cluster_4283_sequences=16	0	16
SDS-V3-plasma-0_Cluster_432_sequences=16	0	16
SDS-V3-plasma-0_Cluster_696_sequences=16	0	16
SDS-V3-plasma-0_Cluster_11422_sequences=16	0	16
SDS-V3-plasma-0_Cluster_1854_sequences=16	0	16
SDS-V3-plasma-0_Cluster_452_sequences=16	0	16
SDS-V3-plasma-0_Cluster_9965_sequences=16	0	16
SDS-V3-plasma-0_Cluster_3613_sequences=16	0	16
SDS-V3-plasma-0_Cluster_3938_sequences=16	0	16
SDS-V3-plasma-0_Cluster_4333_sequences=16	0	16

SDS-V3-plasma-0_Cluster_3440_sequences=16	0	16
SDS-V3-plasma-0_Cluster_8755_sequences=16	0	16
SDS-V3-plasma-0_Cluster_5981_sequences=16	0	16
SDS-V3-plasma-0_Cluster_7641_sequences=16	0	16
SDS-V3-plasma-5_Cluster_1575_sequences=16	9	16
SDS-V3-plasma-5_Cluster_531_sequences=16	9	16
SDS-V3-plasma-5_Cluster_7_sequences=16	9	16
SDS-V3-plasma-5_Cluster_666_sequences=16	9	16
SDS-V3-plasma-5_Cluster_1220_sequences=16	9	16
SDS-V3-plasma-5_Cluster_263_sequences=16	9	16
SDS-V3-plasma-5_Cluster_252_sequences=16	9	16
SDS-V3-plasma-5_Cluster_2_sequences=16	9	16
SDS-V3-plasma-5_Cluster_303_sequences=16	9	16
SDS-V3-plasma-5_Cluster_455_sequences=16	9	16
SDS-V3-plasma-5_Cluster_9_sequences=16	9	16
SDS-V3-plasma-7_Cluster_1199_sequences=16	14	16
SDS-V3-plasma-7_Cluster_1899_sequences=16	14	16
SDS-V3-plasma-7_Cluster_2242_sequences=16	14	16
SDS-V3-plasma-7_Cluster_2325_sequences=16	14	16
SDS-V3-plasma-7_Cluster_2252_sequences=16	14	16
SDS-V3-plasma-7_Cluster_190_sequences=16	14	16
SDS-V3-plasma-7_Cluster_573_sequences=16	14	16
SDS-V3-plasma-7_Cluster_2412_sequences=16	14	16
SDS-V3-plasma-7_Cluster_276_sequences=16	14	16
SDS-V3-plasma-7_Cluster_331_sequences=16	14	16
SDS-V3-plasma-7_Cluster_4451_sequences=16	14	16
SDS-V3-plasma-7_Cluster_1729_sequences=16	14	16
SDS-V3-plasma-7_Cluster_318_sequences=16	14	16
SDS-V3-plasma-7_Cluster_40_sequences=16	14	16
SDS-V3-plasma-8_Cluster_630_sequences=16	16	16
SDS-V3-plasma-8_Cluster_90_sequences=16	16	16
SDS-V3-plasma-8_Cluster_2764_sequences=16	16	16
SDS-V3-plasma-8_Cluster_2498_sequences=16	16	16
SDS-V3-plasma-8_Cluster_2964_sequences=16	16	16
SDS-V3-plasma-8_Cluster_4112_sequences=16	16	16
SDS-V3-plasma-8_Cluster_4564_sequences=16	16	16
SDS-V3-plasma-8_Cluster_3327_sequences=16	16	16
SDS-V3-plasma-8_Cluster_2392_sequences=16	16	16
SDS-V3-plasma-8_Cluster_2313_sequences=16	16	16
SDS-V3-plasma-8_Cluster_2871_sequences=16	16	16
SDS-V3-plasma-24_Cluster_1041_sequences=16	124	16
SDS-V3-plasma-24_Cluster_1368_sequences=16	124	16
SDS-V3-plasma-24_Cluster_1393_sequences=16	124	16
SDS-V3-plasma-24_Cluster_2308_sequences=16	124	16
SDS-V3-plasma-24_Cluster_529_sequences=16	124	16

SDS-V3-plasma-24_Cluster_1188_sequences=16	124	16
SDS-V3-plasma-24_Cluster_2673_sequences=16	124	16
SDS-V3-plasma-24_Cluster_211_sequences=16	124	16
SDS-V3-plasma-24_Cluster_5910_sequences=16	124	16
SDS-V3-plasma-24_Cluster_1463_sequences=16	124	16
SDS-V3-plasma-24_Cluster_813_sequences=16	124	16
SDS-V3-plasma-24_Cluster_1438_sequences=16	124	16
SDS-V3-plasma-24_Cluster_841_sequences=16	124	16
SDS-V3-plasma-24_Cluster_3055_sequences=16	124	16
SDS-V3-plasma-27_Cluster_1774_sequences=16	131	16
SDS-V3-plasma-27_Cluster_2081_sequences=16	131	16
SDS-V3-plasma-27_Cluster_2250_sequences=16	131	16
SDS-V3-plasma-27_Cluster_3777_sequences=16	131	16
SDS-V3-plasma-27_Cluster_599_sequences=16	131	16
SDS-V3-plasma-27_Cluster_910_sequences=16	131	16
SDS-V3-plasma-27_Cluster_961_sequences=16	131	16
SDS-V3-plasma-27_Cluster_1289_sequences=16	131	16
SDS-V3-plasma-27_Cluster_736_sequences=16	131	16
SDS-V3-plasma-27_Cluster_3654_sequences=16	131	16
SDS-V3-plasma-27_Cluster_2034_sequences=16	131	16
SDS-V3-plasma-27_Cluster_828_sequences=16	131	16
SDS-V3-plasma-27_Cluster_1993_sequences=16	131	16
SDS-V3-plasma-27_Cluster_304_sequences=16	131	16
SDS-V3-plasma-27_Cluster_807_sequences=16	131	16
SDS-V3-plasma-27_Cluster_2363_sequences=16	131	16
SDS-V3-plasma-27_Cluster_9_sequences=16	131	16
SDS-V3-plasma-27_Cluster_545_sequences=16	131	16
SDS-V3-plasma-27_Cluster_3763_sequences=16	131	16
SDS-V3-plasma-27_Cluster_1905_sequences=16	131	16
SDS-V3-plasma-45_Cluster_8663_sequences=16	282	16
SDS-V3-plasma-45_Cluster_14929_sequences=16	282	16
SDS-V3-plasma-45_Cluster_27375_sequences=16	282	16
SDS-V3-plasma-45_Cluster_7240_sequences=16	282	16
SDS-V3-plasma-45_Cluster_14510_sequences=16	282	16
SDS-V3-plasma-45_Cluster_5900_sequences=16	282	16
SDS-V3-plasma-45_Cluster_504_sequences=16	282	16
SDS-V3-plasma-45_Cluster_7448_sequences=16	282	16
SDS-V3-plasma-45_Cluster_16421_sequences=16	282	16
SDS-V3-plasma-45_Cluster_2741_sequences=16	282	16
SDS-V3-plasma-45_Cluster_43540_sequences=16	282	16
SDS-V3-plasma-45_Cluster_48936_sequences=16	282	16
SDS-V3-plasma-45_Cluster_7266_sequences=16	282	16
SDS-V3-plasma-45_Cluster_9708_sequences=16	282	16
SDS-V3-plasma-45_Cluster_10722_sequences=16	282	16
SDS-V3-plasma-45_Cluster_14384_sequences=16	282	16

SDS-V3-plasma-45_Cluster_3524_sequences=16	282	16
SDS-V3-plasma-45_Cluster_1635_sequences=16	282	16
SDS-V3-plasma-45_Cluster_25726_sequences=16	282	16
SDS-V3-plasma-45_Cluster_7848_sequences=16	282	16
SDS-V3-plasma-45_Cluster_300_sequences=16	282	16
SDS-V3-plasma-45_Cluster_9870_sequences=16	282	16
SDS-V3-plasma-45_Cluster_33704_sequences=16	282	16
SDS-V3-plasma-45_Cluster_2251_sequences=16	282	16
SDS-V3-plasma-45_Cluster_16999_sequences=16	282	16
SDS-V3-plasma-45_Cluster_10425_sequences=16	282	16
SDS-V3-plasma-45_Cluster_23903_sequences=16	282	16
SDS-V3-plasma-45_Cluster_3702_sequences=16	282	16
SDS-V3-plasma-45_Cluster_4736_sequences=16	282	16
SDS-V3-plasma-45_Cluster_6931_sequences=16	282	16
SDS-V3-plasma-45_Cluster_7013_sequences=16	282	16
SDS-V3-plasma-45_Cluster_3762_sequences=16	282	16
SDS-V3-plasma-45_Cluster_1571_sequences=16	282	16
SDS-V3-plasma-45_Cluster_19330_sequences=16	282	16
SDS-V3-plasma-45_Cluster_2190_sequences=16	282	16
SDS-V3-plasma-45_Cluster_6891_sequences=16	282	16
SDS-V3-plasma-45_Cluster_8898_sequences=16	282	16
SDS-V3-plasma-45_Cluster_9252_sequences=16	282	16
SDS-V3-plasma-45_Cluster_2599_sequences=16	282	16
SDS-V3-plasma-45_Cluster_1174_sequences=16	282	16
SDS-V3-plasma-45_Cluster_2179_sequences=16	282	16
SDS-V3-plasma-45_Cluster_14140_sequences=16	282	16
SDS-V3-plasma-45_Cluster_2164_sequences=16	282	16
SDS-V3-plasma-45_Cluster_10923_sequences=16	282	16
SDS-V3-plasma-45_Cluster_12141_sequences=16	282	16
SDS-V3-plasma-45_Cluster_55_sequences=16	282	16
SDS-V3-plasma-45_Cluster_13066_sequences=16	282	16
SDS-V3-plasma-45_Cluster_14710_sequences=16	282	16
SDS-V3-plasma-45_Cluster_8267_sequences=16	282	16
SDS-V3-plasma-45_Cluster_17300_sequences=16	282	16
SDS-V3-plasma-45_Cluster_18416_sequences=16	282	16
SDS-V3-plasma-45_Cluster_21722_sequences=16	282	16
SDS-V3-plasma-45_Cluster_2525_sequences=16	282	16
SDS-V3-plasma-45_Cluster_5924_sequences=16	282	16
SDS-V3-plasma-45_Cluster_4221_sequences=16	282	16
SDS-V3-plasma-45_Cluster_6483_sequences=16	282	16
SDS-V3-plasma-45_Cluster_18975_sequences=16	282	16
SDS-V3-plasma-45_Cluster_29700_sequences=16	282	16
SDS-V3-plasma-45_Cluster_6361_sequences=16	282	16
SDS-V3-plasma-45_Cluster_9977_sequences=16	282	16
SDS-V3-plasma-45_Cluster_34598_sequences=16	282	16

SDS-V3-plasma-45_Cluster_32682_sequences=16	282	16
SDS-V3-plasma-45_Cluster_4354_sequences=16	282	16
SDS-V3-plasma-45_Cluster_35788_sequences=16	282	16
SDS-V3-plasma-45_Cluster_13939_sequences=16	282	16
SDS-V3-plasma-45_Cluster_28344_sequences=16	282	16
SDS-V3-plasma-45_Cluster_13328_sequences=16	282	16
SDS-V3-plasma-45_Cluster_3809_sequences=16	282	16
SDS-V3-plasma-45_Cluster_5195_sequences=16	282	16
SDS-V3-plasma-45_Cluster_22465_sequences=16	282	16
SDS-V3-plasma-45_Cluster_6934_sequences=16	282	16
SDS-V3-plasma-45_Cluster_4107_sequences=16	282	16
SDS-V3-plasma-45_Cluster_6164_sequences=16	282	16
SDS-V3-plasma-45_Cluster_4031_sequences=16	282	16
SDS-V3-plasma-45_Cluster_8564_sequences=16	282	16
SDS-V3-plasma-45_Cluster_27504_sequences=16	282	16
SDS-V3-plasma-45_Cluster_16455_sequences=16	282	16
SDS-V3-plasma-45_Cluster_4144_sequences=16	282	16
SDS-V3-plasma-45_Cluster_12469_sequences=16	282	16
SDS-V3-plasma-45_Cluster_7488_sequences=16	282	16
SDS-V3-plasma-45_Cluster_7190_sequences=16	282	16
SDS-V3-plasma-45_Cluster_30600_sequences=16	282	16
SDS-V3-plasma-45_Cluster_9054_sequences=16	282	16
SDS-V3-plasma-45_Cluster_28054_sequences=16	282	16
SDS-V3-plasma-45_Cluster_11412_sequences=16	282	16
SDS-V3-plasma-45_Cluster_13623_sequences=16	282	16
SDS-V3-plasma-45_Cluster_4438_sequences=16	282	16
SDS-V3-plasma-45_Cluster_6099_sequences=16	282	16
SDS-V3-plasma-45_Cluster_1204_sequences=16	282	16
SDS-V3-plasma-45_Cluster_15415_sequences=16	282	16
SDS-V3-plasma-45_Cluster_1772_sequences=16	282	16
SDS-V3-plasma-45_Cluster_11362_sequences=16	282	16
SDS-V3-plasma-45_Cluster_20483_sequences=16	282	16
SDS-V3-plasma-45_Cluster_6553_sequences=16	282	16
SDS-V3-plasma-45_Cluster_22819_sequences=16	282	16
SDS-V3-plasma-45_Cluster_5561_sequences=16	282	16
SDS-V3-plasma-45_Cluster_1344_sequences=16	282	16
SDS-V3-plasma-45_Cluster_21343_sequences=16	282	16
SDS-V3-plasma-45_Cluster_7374_sequences=16	282	16
SDS-V3-plasma-45_Cluster_5501_sequences=16	282	16
SDS-V3-plasma-45_Cluster_17036_sequences=16	282	16
SDS-V3-plasma-45_Cluster_6205_sequences=16	282	16
SDS-V3-plasma-45_Cluster_7376_sequences=16	282	16
SDS-V3-plasma-45_Cluster_1773_sequences=16	282	16
SDS-V3-plasma-45_Cluster_4847_sequences=16	282	16
SDS-V3-plasma-45_Cluster_34351_sequences=16	282	16

SDS-V3-plasma-45_Cluster_7244_sequences=16	282	16
SDS-V3-plasma-45_Cluster_4383_sequences=16	282	16
SDS-V3-plasma-45_Cluster_16121_sequences=16	282	16
SDS-V3-plasma-45_Cluster_22877_sequences=16	282	16
SDS-V3-plasma-45_Cluster_25435_sequences=16	282	16
SDS-V3-plasma-45_Cluster_10643_sequences=16	282	16
SDS-V3-plasma-45_Cluster_4724_sequences=16	282	16
SDS-V3-plasma-45_Cluster_14552_sequences=16	282	16
SDS-V3-plasma-45_Cluster_7142_sequences=16	282	16
SDS-V3-plasma-45_Cluster_22651_sequences=16	282	16
SDS-V3-plasma-45_Cluster_19905_sequences=16	282	16
SDS-V3-plasma-45_Cluster_22236_sequences=16	282	16
SDS-V3-plasma-45_Cluster_6469_sequences=16	282	16
SDS-V3-plasma-45_Cluster_8119_sequences=16	282	16
SDS-V3-plasma-45_Cluster_9288_sequences=16	282	16
SDS-V3-plasma-45_Cluster_20310_sequences=16	282	16
SDS-V3-plasma-45_Cluster_7993_sequences=16	282	16
SDS-V3-plasma-46_Cluster_3876_sequences=16	286	16
SDS-V3-plasma-46_Cluster_5181_sequences=16	286	16
SDS-V3-plasma-46_Cluster_6284_sequences=16	286	16
SDS-V3-plasma-46_Cluster_2767_sequences=16	286	16
SDS-V3-plasma-46_Cluster_5629_sequences=16	286	16
SDS-V3-plasma-46_Cluster_1169_sequences=16	286	16
SDS-V3-plasma-46_Cluster_1821_sequences=16	286	16
SDS-V3-plasma-46_Cluster_1149_sequences=16	286	16
SDS-V3-plasma-46_Cluster_10736_sequences=16	286	16
SDS-V3-plasma-46_Cluster_15144_sequences=16	286	16
SDS-V3-plasma-46_Cluster_909_sequences=16	286	16
SDS-V3-plasma-46_Cluster_987_sequences=16	286	16
SDS-V3-plasma-46_Cluster_6495_sequences=16	286	16
SDS-V3-plasma-46_Cluster_119_sequences=16	286	16
SDS-V3-plasma-46_Cluster_1379_sequences=16	286	16
SDS-V3-plasma-46_Cluster_4465_sequences=16	286	16
SDS-V3-plasma-46_Cluster_8421_sequences=16	286	16
SDS-V3-plasma-46_Cluster_1422_sequences=16	286	16
SDS-V3-plasma-46_Cluster_3584_sequences=16	286	16
SDS-V3-plasma-46_Cluster_311_sequences=16	286	16
SDS-V3-plasma-46_Cluster_5112_sequences=16	286	16
SDS-V3-plasma-46_Cluster_305_sequences=16	286	16
SDS-V3-plasma-46_Cluster_3477_sequences=16	286	16
SDS-V3-plasma-46_Cluster_8082_sequences=16	286	16
SDS-V3-plasma-46_Cluster_3140_sequences=16	286	16
SDS-V3-plasma-46_Cluster_9083_sequences=16	286	16
SDS-V3-plasma-46_Cluster_2935_sequences=16	286	16
SDS-V3-plasma-46_Cluster_1446_sequences=16	286	16

SDS-V3-plasma-46_Cluster_4094_sequences=16	286	16
SDS-V3-plasma-46_Cluster_4468_sequences=16	286	16
SDS-V3-plasma-46_Cluster_457_sequences=16	286	16
SDS-V3-plasma-46_Cluster_5720_sequences=16	286	16
SDS-V3-plasma-46_Cluster_5899_sequences=16	286	16
SDS-V3-plasma-46_Cluster_7270_sequences=16	286	16
SDS-V3-plasma-46_Cluster_1706_sequences=16	286	16
SDS-V3-plasma-46_Cluster_480_sequences=16	286	16
SDS-V3-plasma-46_Cluster_5196_sequences=16	286	16
SDS-V3-plasma-46_Cluster_3901_sequences=16	286	16
SDS-V3-plasma-46_Cluster_8431_sequences=16	286	16
SDS-V3-plasma-46_Cluster_1376_sequences=16	286	16
SDS-V3-plasma-46_Cluster_295_sequences=16	286	16
SDS-V3-plasma-46_Cluster_753_sequences=16	286	16
SDS-V3-plasma-46_Cluster_851_sequences=16	286	16
SDS-V3-plasma-46_Cluster_478_sequences=16	286	16
SDS-V3-plasma-46_Cluster_3913_sequences=16	286	16
SDS-V3-plasma-46_Cluster_2205_sequences=16	286	16
SDS-V3-plasma-46_Cluster_2512_sequences=16	286	16
SDS-V3-plasma-46_Cluster_1751_sequences=16	286	16
SDS-V3-plasma-46_Cluster_755_sequences=16	286	16
SDS-V3-plasma-46_Cluster_4425_sequences=16	286	16
SDS-V3-plasma-46_Cluster_4947_sequences=16	286	16
SDS-V3-plasma-67_Cluster_20078_sequences=16	504	16
SDS-V3-plasma-67_Cluster_13109_sequences=16	504	16
SDS-V3-plasma-67_Cluster_20801_sequences=16	504	16
SDS-V3-plasma-67_Cluster_76683_sequences=16	504	16
SDS-V3-plasma-67_Cluster_2925_sequences=16	504	16
SDS-V3-plasma-67_Cluster_3120_sequences=16	504	16
SDS-V3-plasma-67_Cluster_4763_sequences=16	504	16
SDS-V3-plasma-67_Cluster_4857_sequences=16	504	16
SDS-V3-plasma-67_Cluster_9944_sequences=16	504	16
SDS-V3-plasma-67_Cluster_18356_sequences=16	504	16
SDS-V3-plasma-67_Cluster_18021_sequences=16	504	16
SDS-V3-plasma-67_Cluster_20551_sequences=16	504	16
SDS-V3-plasma-67_Cluster_2686_sequences=16	504	16
SDS-V3-plasma-67_Cluster_3146_sequences=16	504	16
SDS-V3-plasma-67_Cluster_36170_sequences=16	504	16
SDS-V3-plasma-67_Cluster_40246_sequences=16	504	16
SDS-V3-plasma-67_Cluster_5150_sequences=16	504	16
SDS-V3-plasma-67_Cluster_6062_sequences=16	504	16
SDS-V3-plasma-67_Cluster_12044_sequences=16	504	16
SDS-V3-plasma-67_Cluster_14616_sequences=16	504	16
SDS-V3-plasma-67_Cluster_13337_sequences=16	504	16
SDS-V3-plasma-67_Cluster_2477_sequences=16	504	16

SDS-V3-plasma-67_Cluster_20216_sequences=16	504	16
SDS-V3-plasma-67_Cluster_12367_sequences=16	504	16
SDS-V3-plasma-67_Cluster_5839_sequences=16	504	16
SDS-V3-plasma-67_Cluster_2030_sequences=16	504	16
SDS-V3-plasma-67_Cluster_11221_sequences=16	504	16
SDS-V3-plasma-67_Cluster_75577_sequences=16	504	16
SDS-V3-plasma-67_Cluster_11090_sequences=16	504	16
SDS-V3-plasma-67_Cluster_28936_sequences=16	504	16
SDS-V3-plasma-67_Cluster_19274_sequences=16	504	16
SDS-V3-plasma-67_Cluster_14049_sequences=16	504	16
SDS-V3-plasma-67_Cluster_8622_sequences=16	504	16
SDS-V3-plasma-67_Cluster_925_sequences=16	504	16
SDS-V3-plasma-67_Cluster_9548_sequences=16	504	16
SDS-V3-plasma-67_Cluster_8518_sequences=16	504	16
SDS-V3-plasma-67_Cluster_3375_sequences=16	504	16
SDS-V3-plasma-67_Cluster_12060_sequences=16	504	16
SDS-V3-plasma-67_Cluster_12216_sequences=16	504	16
SDS-V3-plasma-67_Cluster_15358_sequences=16	504	16
SDS-V3-plasma-67_Cluster_18024_sequences=16	504	16
SDS-V3-plasma-67_Cluster_31436_sequences=16	504	16
SDS-V3-plasma-67_Cluster_4510_sequences=16	504	16
SDS-V3-plasma-67_Cluster_6305_sequences=16	504	16
SDS-V3-plasma-67_Cluster_7134_sequences=16	504	16
SDS-V3-plasma-67_Cluster_21521_sequences=16	504	16
SDS-V3-plasma-67_Cluster_35540_sequences=16	504	16
SDS-V3-plasma-67_Cluster_12465_sequences=16	504	16
SDS-V3-plasma-67_Cluster_21900_sequences=16	504	16
SDS-V3-plasma-67_Cluster_52190_sequences=16	504	16
SDS-V3-plasma-67_Cluster_21184_sequences=16	504	16
SDS-V3-plasma-67_Cluster_18940_sequences=16	504	16
SDS-V3-plasma-67_Cluster_1399_sequences=16	504	16
SDS-V3-plasma-67_Cluster_5183_sequences=16	504	16
SDS-V3-plasma-67_Cluster_3856_sequences=16	504	16
SDS-V3-plasma-67_Cluster_4279_sequences=16	504	16
SDS-V3-plasma-67_Cluster_16612_sequences=16	504	16
SDS-V3-plasma-67_Cluster_5083_sequences=16	504	16
SDS-V3-plasma-67_Cluster_34494_sequences=16	504	16
SDS-V3-plasma-67_Cluster_20823_sequences=16	504	16
SDS-V3-plasma-67_Cluster_487_sequences=16	504	16
SDS-V3-plasma-67_Cluster_12703_sequences=16	504	16
SDS-V3-plasma-67_Cluster_4515_sequences=16	504	16
SDS-V3-plasma-67_Cluster_7844_sequences=16	504	16
SDS-V3-plasma-67_Cluster_6411_sequences=16	504	16
SDS-V3-plasma-67_Cluster_14939_sequences=16	504	16
SDS-V3-plasma-67_Cluster_12296_sequences=16	504	16

SDS-V3-plasma-67_Cluster_2057_sequences=16	504	16
SDS-V3-plasma-67_Cluster_32809_sequences=16	504	16
SDS-V3-plasma-67_Cluster_9806_sequences=16	504	16
SDS-V3-plasma-67_Cluster_15276_sequences=16	504	16
SDS-V3-plasma-67_Cluster_6148_sequences=16	504	16
SDS-V3-plasma-67_Cluster_20180_sequences=16	504	16
SDS-V3-plasma-67_Cluster_9268_sequences=16	504	16
SDS-V3-plasma-67_Cluster_3388_sequences=16	504	16
SDS-V3-plasma-67_Cluster_15976_sequences=16	504	16
SDS-V3-plasma-67_Cluster_20393_sequences=16	504	16
SDS-V3-plasma-67_Cluster_7944_sequences=16	504	16
SDS-V3-plasma-67_Cluster_6894_sequences=16	504	16
SDS-V3-plasma-67_Cluster_23657_sequences=16	504	16
SDS-V3-plasma-67_Cluster_75614_sequences=16	504	16
SDS-V3-plasma-67_Cluster_33409_sequences=16	504	16
SDS-V3-plasma-67_Cluster_21626_sequences=16	504	16
SDS-V3-plasma-67_Cluster_3550_sequences=16	504	16
SDS-V3-plasma-67_Cluster_25311_sequences=16	504	16
SDS-V3-plasma-0_Cluster_8980_sequences=15	0	15
SDS-V3-plasma-0_Cluster_2181_sequences=15	0	15
SDS-V3-plasma-0_Cluster_3687_sequences=15	0	15
SDS-V3-plasma-0_Cluster_15019_sequences=15	0	15
SDS-V3-plasma-0_Cluster_1690_sequences=15	0	15
SDS-V3-plasma-0_Cluster_2009_sequences=15	0	15
SDS-V3-plasma-0_Cluster_2530_sequences=15	0	15
SDS-V3-plasma-0_Cluster_3021_sequences=15	0	15
SDS-V3-plasma-0_Cluster_5833_sequences=15	0	15
SDS-V3-plasma-0_Cluster_662_sequences=15	0	15
SDS-V3-plasma-0_Cluster_6838_sequences=15	0	15
SDS-V3-plasma-0_Cluster_7969_sequences=15	0	15
SDS-V3-plasma-0_Cluster_8461_sequences=15	0	15
SDS-V3-plasma-0_Cluster_953_sequences=15	0	15
SDS-V3-plasma-0_Cluster_6356_sequences=15	0	15
SDS-V3-plasma-0_Cluster_2640_sequences=15	0	15
SDS-V3-plasma-0_Cluster_1115_sequences=15	0	15
SDS-V3-plasma-0_Cluster_1506_sequences=15	0	15
SDS-V3-plasma-0_Cluster_5671_sequences=15	0	15
SDS-V3-plasma-0_Cluster_3908_sequences=15	0	15
SDS-V3-plasma-0_Cluster_2626_sequences=15	0	15
SDS-V3-plasma-0_Cluster_594_sequences=15	0	15
SDS-V3-plasma-0_Cluster_10776_sequences=15	0	15
SDS-V3-plasma-0_Cluster_5318_sequences=15	0	15
SDS-V3-plasma-0_Cluster_2883_sequences=15	0	15
SDS-V3-plasma-0_Cluster_598_sequences=15	0	15
SDS-V3-plasma-0_Cluster_8656_sequences=15	0	15

SDS-V3-plasma-0_Cluster_11764_sequences=15	0	15
SDS-V3-plasma-0_Cluster_10317_sequences=15	0	15
SDS-V3-plasma-0_Cluster_3474_sequences=15	0	15
SDS-V3-plasma-0_Cluster_2393_sequences=15	0	15
SDS-V3-plasma-0_Cluster_5192_sequences=15	0	15
SDS-V3-plasma-0_Cluster_9901_sequences=15	0	15
SDS-V3-plasma-0_Cluster_1219_sequences=15	0	15
SDS-V3-plasma-0_Cluster_1160_sequences=15	0	15
SDS-V3-plasma-0_Cluster_12744_sequences=15	0	15
SDS-V3-plasma-0_Cluster_1311_sequences=15	0	15
SDS-V3-plasma-0_Cluster_1649_sequences=15	0	15
SDS-V3-plasma-0_Cluster_3551_sequences=15	0	15
SDS-V3-plasma-0_Cluster_5091_sequences=15	0	15
SDS-V3-plasma-0_Cluster_8775_sequences=15	0	15
SDS-V3-plasma-0_Cluster_9114_sequences=15	0	15
SDS-V3-plasma-0_Cluster_9930_sequences=15	0	15
SDS-V3-plasma-0_Cluster_597_sequences=15	0	15
SDS-V3-plasma-0_Cluster_6042_sequences=15	0	15
SDS-V3-plasma-0_Cluster_1242_sequences=15	0	15
SDS-V3-plasma-0_Cluster_6411_sequences=15	0	15
SDS-V3-plasma-0_Cluster_9387_sequences=15	0	15
SDS-V3-plasma-0_Cluster_7956_sequences=15	0	15
SDS-V3-plasma-0_Cluster_1880_sequences=15	0	15
SDS-V3-plasma-0_Cluster_110_sequences=15	0	15
SDS-V3-plasma-0_Cluster_2486_sequences=15	0	15
SDS-V3-plasma-0_Cluster_588_sequences=15	0	15
SDS-V3-plasma-0_Cluster_5680_sequences=15	0	15
SDS-V3-plasma-0_Cluster_1842_sequences=15	0	15
SDS-V3-plasma-0_Cluster_8263_sequences=15	0	15
SDS-V3-plasma-0_Cluster_2451_sequences=15	0	15
SDS-V3-plasma-0_Cluster_2931_sequences=15	0	15
SDS-V3-plasma-0_Cluster_9975_sequences=15	0	15
SDS-V3-plasma-5_Cluster_107_sequences=15	9	15
SDS-V3-plasma-5_Cluster_201_sequences=15	9	15
SDS-V3-plasma-5_Cluster_38_sequences=15	9	15
SDS-V3-plasma-5_Cluster_705_sequences=15	9	15
SDS-V3-plasma-5_Cluster_212_sequences=15	9	15
SDS-V3-plasma-5_Cluster_228_sequences=15	9	15
SDS-V3-plasma-5_Cluster_81_sequences=15	9	15
SDS-V3-plasma-5_Cluster_352_sequences=15	9	15
SDS-V3-plasma-5_Cluster_520_sequences=15	9	15
SDS-V3-plasma-5_Cluster_57_sequences=15	9	15
SDS-V3-plasma-5_Cluster_78_sequences=15	9	15
SDS-V3-plasma-7_Cluster_57_sequences=15	14	15
SDS-V3-plasma-7_Cluster_954_sequences=15	14	15

SDS-V3-plasma-7_Cluster_3337_sequences=15	14	15
SDS-V3-plasma-7_Cluster_4196_sequences=15	14	15
SDS-V3-plasma-7_Cluster_236_sequences=15	14	15
SDS-V3-plasma-7_Cluster_993_sequences=15	14	15
SDS-V3-plasma-7_Cluster_888_sequences=15	14	15
SDS-V3-plasma-7_Cluster_2264_sequences=15	14	15
SDS-V3-plasma-7_Cluster_1176_sequences=15	14	15
SDS-V3-plasma-7_Cluster_1271_sequences=15	14	15
SDS-V3-plasma-7_Cluster_739_sequences=15	14	15
SDS-V3-plasma-7_Cluster_2726_sequences=15	14	15
SDS-V3-plasma-7_Cluster_95_sequences=15	14	15
SDS-V3-plasma-7_Cluster_90_sequences=15	14	15
SDS-V3-plasma-7_Cluster_5650_sequences=15	14	15
SDS-V3-plasma-7_Cluster_1168_sequences=15	14	15
SDS-V3-plasma-7_Cluster_1004_sequences=15	14	15
SDS-V3-plasma-7_Cluster_1692_sequences=15	14	15
SDS-V3-plasma-7_Cluster_3446_sequences=15	14	15
SDS-V3-plasma-7_Cluster_1627_sequences=15	14	15
SDS-V3-plasma-7_Cluster_3336_sequences=15	14	15
SDS-V3-plasma-7_Cluster_776_sequences=15	14	15
SDS-V3-plasma-8_Cluster_422_sequences=15	16	15
SDS-V3-plasma-8_Cluster_115_sequences=15	16	15
SDS-V3-plasma-8_Cluster_3027_sequences=15	16	15
SDS-V3-plasma-8_Cluster_3306_sequences=15	16	15
SDS-V3-plasma-8_Cluster_2747_sequences=15	16	15
SDS-V3-plasma-8_Cluster_2649_sequences=15	16	15
SDS-V3-plasma-8_Cluster_3498_sequences=15	16	15
SDS-V3-plasma-8_Cluster_2407_sequences=15	16	15
SDS-V3-plasma-8_Cluster_2496_sequences=15	16	15
SDS-V3-plasma-8_Cluster_2963_sequences=15	16	15
SDS-V3-plasma-8_Cluster_3689_sequences=15	16	15
SDS-V3-plasma-8_Cluster_4557_sequences=15	16	15
SDS-V3-plasma-8_Cluster_2778_sequences=15	16	15
SDS-V3-plasma-8_Cluster_2953_sequences=15	16	15
SDS-V3-plasma-8_Cluster_2556_sequences=15	16	15
SDS-V3-plasma-8_Cluster_4196_sequences=15	16	15
SDS-V3-plasma-24_Cluster_2122_sequences=15	124	15
SDS-V3-plasma-24_Cluster_1490_sequences=15	124	15
SDS-V3-plasma-24_Cluster_3203_sequences=15	124	15
SDS-V3-plasma-24_Cluster_3205_sequences=15	124	15
SDS-V3-plasma-24_Cluster_461_sequences=15	124	15
SDS-V3-plasma-24_Cluster_694_sequences=15	124	15
SDS-V3-plasma-24_Cluster_8292_sequences=15	124	15
SDS-V3-plasma-24_Cluster_984_sequences=15	124	15
SDS-V3-plasma-24_Cluster_2291_sequences=15	124	15

SDS-V3-plasma-24_Cluster_844_sequences=15	124	15
SDS-V3-plasma-24_Cluster_4566_sequences=15	124	15
SDS-V3-plasma-24_Cluster_1162_sequences=15	124	15
SDS-V3-plasma-24_Cluster_1867_sequences=15	124	15
SDS-V3-plasma-24_Cluster_1250_sequences=15	124	15
SDS-V3-plasma-24_Cluster_894_sequences=15	124	15
SDS-V3-plasma-24_Cluster_663_sequences=15	124	15
SDS-V3-plasma-24_Cluster_2492_sequences=15	124	15
SDS-V3-plasma-24_Cluster_2196_sequences=15	124	15
SDS-V3-plasma-24_Cluster_485_sequences=15	124	15
SDS-V3-plasma-27_Cluster_1054_sequences=15	131	15
SDS-V3-plasma-27_Cluster_1608_sequences=15	131	15
SDS-V3-plasma-27_Cluster_25_sequences=15	131	15
SDS-V3-plasma-27_Cluster_3105_sequences=15	131	15
SDS-V3-plasma-27_Cluster_716_sequences=15	131	15
SDS-V3-plasma-27_Cluster_726_sequences=15	131	15
SDS-V3-plasma-27_Cluster_2288_sequences=15	131	15
SDS-V3-plasma-27_Cluster_6464_sequences=15	131	15
SDS-V3-plasma-27_Cluster_4784_sequences=15	131	15
SDS-V3-plasma-27_Cluster_845_sequences=15	131	15
SDS-V3-plasma-27_Cluster_4649_sequences=15	131	15
SDS-V3-plasma-27_Cluster_2388_sequences=15	131	15
SDS-V3-plasma-27_Cluster_2197_sequences=15	131	15
SDS-V3-plasma-27_Cluster_5155_sequences=15	131	15
SDS-V3-plasma-45_Cluster_5516_sequences=15	282	15
SDS-V3-plasma-45_Cluster_6406_sequences=15	282	15
SDS-V3-plasma-45_Cluster_15336_sequences=15	282	15
SDS-V3-plasma-45_Cluster_6199_sequences=15	282	15
SDS-V3-plasma-45_Cluster_3011_sequences=15	282	15
SDS-V3-plasma-45_Cluster_8345_sequences=15	282	15
SDS-V3-plasma-45_Cluster_2812_sequences=15	282	15
SDS-V3-plasma-45_Cluster_14632_sequences=15	282	15
SDS-V3-plasma-45_Cluster_17276_sequences=15	282	15
SDS-V3-plasma-45_Cluster_15801_sequences=15	282	15
SDS-V3-plasma-45_Cluster_17947_sequences=15	282	15
SDS-V3-plasma-45_Cluster_10717_sequences=15	282	15
SDS-V3-plasma-45_Cluster_6783_sequences=15	282	15
SDS-V3-plasma-45_Cluster_18764_sequences=15	282	15
SDS-V3-plasma-45_Cluster_5112_sequences=15	282	15
SDS-V3-plasma-45_Cluster_10497_sequences=15	282	15
SDS-V3-plasma-45_Cluster_10220_sequences=15	282	15
SDS-V3-plasma-45_Cluster_23274_sequences=15	282	15
SDS-V3-plasma-45_Cluster_29585_sequences=15	282	15
SDS-V3-plasma-45_Cluster_33051_sequences=15	282	15
SDS-V3-plasma-45_Cluster_6233_sequences=15	282	15

SDS-V3-plasma-45_Cluster_6807_sequences=15	282	15
SDS-V3-plasma-45_Cluster_7069_sequences=15	282	15
SDS-V3-plasma-45_Cluster_7251_sequences=15	282	15
SDS-V3-plasma-45_Cluster_795_sequences=15	282	15
SDS-V3-plasma-45_Cluster_844_sequences=15	282	15
SDS-V3-plasma-45_Cluster_989_sequences=15	282	15
SDS-V3-plasma-45_Cluster_18482_sequences=15	282	15
SDS-V3-plasma-45_Cluster_1905_sequences=15	282	15
SDS-V3-plasma-45_Cluster_19776_sequences=15	282	15
SDS-V3-plasma-45_Cluster_2228_sequences=15	282	15
SDS-V3-plasma-45_Cluster_255_sequences=15	282	15
SDS-V3-plasma-45_Cluster_3897_sequences=15	282	15
SDS-V3-plasma-45_Cluster_43967_sequences=15	282	15
SDS-V3-plasma-45_Cluster_4858_sequences=15	282	15
SDS-V3-plasma-45_Cluster_1277_sequences=15	282	15
SDS-V3-plasma-45_Cluster_1488_sequences=15	282	15
SDS-V3-plasma-45_Cluster_13919_sequences=15	282	15
SDS-V3-plasma-45_Cluster_2081_sequences=15	282	15
SDS-V3-plasma-45_Cluster_9199_sequences=15	282	15
SDS-V3-plasma-45_Cluster_13363_sequences=15	282	15
SDS-V3-plasma-45_Cluster_16077_sequences=15	282	15
SDS-V3-plasma-45_Cluster_263_sequences=15	282	15
SDS-V3-plasma-45_Cluster_34551_sequences=15	282	15
SDS-V3-plasma-45_Cluster_26176_sequences=15	282	15
SDS-V3-plasma-45_Cluster_19558_sequences=15	282	15
SDS-V3-plasma-45_Cluster_23755_sequences=15	282	15
SDS-V3-plasma-45_Cluster_3836_sequences=15	282	15
SDS-V3-plasma-45_Cluster_4845_sequences=15	282	15
SDS-V3-plasma-45_Cluster_4350_sequences=15	282	15
SDS-V3-plasma-45_Cluster_7030_sequences=15	282	15
SDS-V3-plasma-45_Cluster_9094_sequences=15	282	15
SDS-V3-plasma-45_Cluster_15713_sequences=15	282	15
SDS-V3-plasma-45_Cluster_7235_sequences=15	282	15
SDS-V3-plasma-45_Cluster_9319_sequences=15	282	15
SDS-V3-plasma-45_Cluster_17078_sequences=15	282	15
SDS-V3-plasma-45_Cluster_1987_sequences=15	282	15
SDS-V3-plasma-45_Cluster_610_sequences=15	282	15
SDS-V3-plasma-45_Cluster_9801_sequences=15	282	15
SDS-V3-plasma-45_Cluster_15032_sequences=15	282	15
SDS-V3-plasma-45_Cluster_14554_sequences=15	282	15
SDS-V3-plasma-45_Cluster_8415_sequences=15	282	15
SDS-V3-plasma-45_Cluster_488_sequences=15	282	15
SDS-V3-plasma-45_Cluster_38719_sequences=15	282	15
SDS-V3-plasma-45_Cluster_5473_sequences=15	282	15
SDS-V3-plasma-45_Cluster_14827_sequences=15	282	15

SDS-V3-plasma-45_Cluster_495_sequences=15	282	15
SDS-V3-plasma-45_Cluster_220_sequences=15	282	15
SDS-V3-plasma-45_Cluster_9294_sequences=15	282	15
SDS-V3-plasma-45_Cluster_45_sequences=15	282	15
SDS-V3-plasma-45_Cluster_27168_sequences=15	282	15
SDS-V3-plasma-45_Cluster_25215_sequences=15	282	15
SDS-V3-plasma-45_Cluster_5260_sequences=15	282	15
SDS-V3-plasma-45_Cluster_4592_sequences=15	282	15
SDS-V3-plasma-45_Cluster_16483_sequences=15	282	15
SDS-V3-plasma-45_Cluster_7446_sequences=15	282	15
SDS-V3-plasma-45_Cluster_5044_sequences=15	282	15
SDS-V3-plasma-45_Cluster_5918_sequences=15	282	15
SDS-V3-plasma-45_Cluster_2855_sequences=15	282	15
SDS-V3-plasma-45_Cluster_4832_sequences=15	282	15
SDS-V3-plasma-45_Cluster_22635_sequences=15	282	15
SDS-V3-plasma-45_Cluster_2416_sequences=15	282	15
SDS-V3-plasma-45_Cluster_15560_sequences=15	282	15
SDS-V3-plasma-45_Cluster_15368_sequences=15	282	15
SDS-V3-plasma-45_Cluster_21522_sequences=15	282	15
SDS-V3-plasma-45_Cluster_19865_sequences=15	282	15
SDS-V3-plasma-45_Cluster_1082_sequences=15	282	15
SDS-V3-plasma-45_Cluster_4303_sequences=15	282	15
SDS-V3-plasma-45_Cluster_4106_sequences=15	282	15
SDS-V3-plasma-45_Cluster_5499_sequences=15	282	15
SDS-V3-plasma-45_Cluster_5493_sequences=15	282	15
SDS-V3-plasma-45_Cluster_2107_sequences=15	282	15
SDS-V3-plasma-45_Cluster_7937_sequences=15	282	15
SDS-V3-plasma-45_Cluster_10560_sequences=15	282	15
SDS-V3-plasma-45_Cluster_9085_sequences=15	282	15
SDS-V3-plasma-45_Cluster_6573_sequences=15	282	15
SDS-V3-plasma-45_Cluster_16145_sequences=15	282	15
SDS-V3-plasma-45_Cluster_11914_sequences=15	282	15
SDS-V3-plasma-45_Cluster_19679_sequences=15	282	15
SDS-V3-plasma-45_Cluster_5094_sequences=15	282	15
SDS-V3-plasma-45_Cluster_25122_sequences=15	282	15
SDS-V3-plasma-45_Cluster_2270_sequences=15	282	15
SDS-V3-plasma-45_Cluster_1207_sequences=15	282	15
SDS-V3-plasma-45_Cluster_11452_sequences=15	282	15
SDS-V3-plasma-45_Cluster_13106_sequences=15	282	15
SDS-V3-plasma-45_Cluster_13947_sequences=15	282	15
SDS-V3-plasma-45_Cluster_18359_sequences=15	282	15
SDS-V3-plasma-45_Cluster_14521_sequences=15	282	15
SDS-V3-plasma-45_Cluster_12214_sequences=15	282	15
SDS-V3-plasma-45_Cluster_21588_sequences=15	282	15
SDS-V3-plasma-45_Cluster_604_sequences=15	282	15

SDS-V3-plasma-45_Cluster_12636_sequences=15	282	15
SDS-V3-plasma-45_Cluster_1775_sequences=15	282	15
SDS-V3-plasma-45_Cluster_6244_sequences=15	282	15
SDS-V3-plasma-45_Cluster_20184_sequences=15	282	15
SDS-V3-plasma-45_Cluster_7609_sequences=15	282	15
SDS-V3-plasma-45_Cluster_12980_sequences=15	282	15
SDS-V3-plasma-45_Cluster_10485_sequences=15	282	15
SDS-V3-plasma-45_Cluster_1572_sequences=15	282	15
SDS-V3-plasma-45_Cluster_19840_sequences=15	282	15
SDS-V3-plasma-45_Cluster_389_sequences=15	282	15
SDS-V3-plasma-45_Cluster_7899_sequences=15	282	15
SDS-V3-plasma-45_Cluster_9495_sequences=15	282	15
SDS-V3-plasma-45_Cluster_14667_sequences=15	282	15
SDS-V3-plasma-45_Cluster_747_sequences=15	282	15
SDS-V3-plasma-45_Cluster_3649_sequences=15	282	15
SDS-V3-plasma-45_Cluster_7538_sequences=15	282	15
SDS-V3-plasma-45_Cluster_15741_sequences=15	282	15
SDS-V3-plasma-45_Cluster_37129_sequences=15	282	15
SDS-V3-plasma-45_Cluster_10550_sequences=15	282	15
SDS-V3-plasma-45_Cluster_10566_sequences=15	282	15
SDS-V3-plasma-45_Cluster_11022_sequences=15	282	15
SDS-V3-plasma-45_Cluster_15711_sequences=15	282	15
SDS-V3-plasma-45_Cluster_1201_sequences=15	282	15
SDS-V3-plasma-45_Cluster_13270_sequences=15	282	15
SDS-V3-plasma-45_Cluster_1768_sequences=15	282	15
SDS-V3-plasma-45_Cluster_11073_sequences=15	282	15
SDS-V3-plasma-45_Cluster_3194_sequences=15	282	15
SDS-V3-plasma-45_Cluster_5942_sequences=15	282	15
SDS-V3-plasma-45_Cluster_12771_sequences=15	282	15
SDS-V3-plasma-45_Cluster_30795_sequences=15	282	15
SDS-V3-plasma-45_Cluster_4174_sequences=15	282	15
SDS-V3-plasma-45_Cluster_4893_sequences=15	282	15
SDS-V3-plasma-46_Cluster_4305_sequences=15	286	15
SDS-V3-plasma-46_Cluster_3759_sequences=15	286	15
SDS-V3-plasma-46_Cluster_2351_sequences=15	286	15
SDS-V3-plasma-46_Cluster_9341_sequences=15	286	15
SDS-V3-plasma-46_Cluster_3260_sequences=15	286	15
SDS-V3-plasma-46_Cluster_4114_sequences=15	286	15
SDS-V3-plasma-46_Cluster_916_sequences=15	286	15
SDS-V3-plasma-46_Cluster_1425_sequences=15	286	15
SDS-V3-plasma-46_Cluster_1441_sequences=15	286	15
SDS-V3-plasma-46_Cluster_169_sequences=15	286	15
SDS-V3-plasma-46_Cluster_2631_sequences=15	286	15
SDS-V3-plasma-46_Cluster_271_sequences=15	286	15
SDS-V3-plasma-46_Cluster_3555_sequences=15	286	15

SDS-V3-plasma-46_Cluster_5195_sequences=15	286	15
SDS-V3-plasma-46_Cluster_12648_sequences=15	286	15
SDS-V3-plasma-46_Cluster_7782_sequences=15	286	15
SDS-V3-plasma-46_Cluster_93_sequences=15	286	15
SDS-V3-plasma-46_Cluster_1971_sequences=15	286	15
SDS-V3-plasma-46_Cluster_118_sequences=15	286	15
SDS-V3-plasma-46_Cluster_5909_sequences=15	286	15
SDS-V3-plasma-46_Cluster_21592_sequences=15	286	15
SDS-V3-plasma-46_Cluster_2858_sequences=15	286	15
SDS-V3-plasma-46_Cluster_3028_sequences=15	286	15
SDS-V3-plasma-46_Cluster_3583_sequences=15	286	15
SDS-V3-plasma-46_Cluster_10507_sequences=15	286	15
SDS-V3-plasma-46_Cluster_10462_sequences=15	286	15
SDS-V3-plasma-46_Cluster_1647_sequences=15	286	15
SDS-V3-plasma-46_Cluster_2523_sequences=15	286	15
SDS-V3-plasma-46_Cluster_2761_sequences=15	286	15
SDS-V3-plasma-46_Cluster_4341_sequences=15	286	15
SDS-V3-plasma-46_Cluster_8141_sequences=15	286	15
SDS-V3-plasma-46_Cluster_129_sequences=15	286	15
SDS-V3-plasma-46_Cluster_2472_sequences=15	286	15
SDS-V3-plasma-46_Cluster_8537_sequences=15	286	15
SDS-V3-plasma-46_Cluster_5168_sequences=15	286	15
SDS-V3-plasma-46_Cluster_4226_sequences=15	286	15
SDS-V3-plasma-46_Cluster_5226_sequences=15	286	15
SDS-V3-plasma-46_Cluster_12610_sequences=15	286	15
SDS-V3-plasma-46_Cluster_17_sequences=15	286	15
SDS-V3-plasma-46_Cluster_7225_sequences=15	286	15
SDS-V3-plasma-46_Cluster_655_sequences=15	286	15
SDS-V3-plasma-46_Cluster_248_sequences=15	286	15
SDS-V3-plasma-46_Cluster_4981_sequences=15	286	15
SDS-V3-plasma-46_Cluster_3035_sequences=15	286	15
SDS-V3-plasma-46_Cluster_3541_sequences=15	286	15
SDS-V3-plasma-46_Cluster_6235_sequences=15	286	15
SDS-V3-plasma-46_Cluster_9794_sequences=15	286	15
SDS-V3-plasma-46_Cluster_3450_sequences=15	286	15
SDS-V3-plasma-46_Cluster_4166_sequences=15	286	15
SDS-V3-plasma-46_Cluster_2311_sequences=15	286	15
SDS-V3-plasma-46_Cluster_2898_sequences=15	286	15
SDS-V3-plasma-46_Cluster_8506_sequences=15	286	15
SDS-V3-plasma-46_Cluster_7535_sequences=15	286	15
SDS-V3-plasma-46_Cluster_2824_sequences=15	286	15
SDS-V3-plasma-46_Cluster_613_sequences=15	286	15
SDS-V3-plasma-46_Cluster_6880_sequences=15	286	15
SDS-V3-plasma-46_Cluster_442_sequences=15	286	15
SDS-V3-plasma-67_Cluster_13522_sequences=15	504	15

SDS-V3-plasma-67_Cluster_9918_sequences=15	504	15
SDS-V3-plasma-67_Cluster_35451_sequences=15	504	15
SDS-V3-plasma-67_Cluster_13800_sequences=15	504	15
SDS-V3-plasma-67_Cluster_9978_sequences=15	504	15
SDS-V3-plasma-67_Cluster_16107_sequences=15	504	15
SDS-V3-plasma-67_Cluster_17502_sequences=15	504	15
SDS-V3-plasma-67_Cluster_4704_sequences=15	504	15
SDS-V3-plasma-67_Cluster_23843_sequences=15	504	15
SDS-V3-plasma-67_Cluster_8815_sequences=15	504	15
SDS-V3-plasma-67_Cluster_6889_sequences=15	504	15
SDS-V3-plasma-67_Cluster_10354_sequences=15	504	15
SDS-V3-plasma-67_Cluster_4943_sequences=15	504	15
SDS-V3-plasma-67_Cluster_22070_sequences=15	504	15
SDS-V3-plasma-67_Cluster_45872_sequences=15	504	15
SDS-V3-plasma-67_Cluster_12345_sequences=15	504	15
SDS-V3-plasma-67_Cluster_13168_sequences=15	504	15
SDS-V3-plasma-67_Cluster_6874_sequences=15	504	15
SDS-V3-plasma-67_Cluster_10546_sequences=15	504	15
SDS-V3-plasma-67_Cluster_15875_sequences=15	504	15
SDS-V3-plasma-67_Cluster_23619_sequences=15	504	15
SDS-V3-plasma-67_Cluster_45536_sequences=15	504	15
SDS-V3-plasma-67_Cluster_62356_sequences=15	504	15
SDS-V3-plasma-67_Cluster_12319_sequences=15	504	15
SDS-V3-plasma-67_Cluster_1500_sequences=15	504	15
SDS-V3-plasma-67_Cluster_18328_sequences=15	504	15
SDS-V3-plasma-67_Cluster_6090_sequences=15	504	15
SDS-V3-plasma-67_Cluster_7877_sequences=15	504	15
SDS-V3-plasma-67_Cluster_13808_sequences=15	504	15
SDS-V3-plasma-67_Cluster_19434_sequences=15	504	15
SDS-V3-plasma-67_Cluster_19355_sequences=15	504	15
SDS-V3-plasma-67_Cluster_19115_sequences=15	504	15
SDS-V3-plasma-67_Cluster_141_sequences=15	504	15
SDS-V3-plasma-67_Cluster_25947_sequences=15	504	15
SDS-V3-plasma-67_Cluster_15979_sequences=15	504	15
SDS-V3-plasma-67_Cluster_17920_sequences=15	504	15
SDS-V3-plasma-67_Cluster_22004_sequences=15	504	15
SDS-V3-plasma-67_Cluster_22047_sequences=15	504	15
SDS-V3-plasma-67_Cluster_34511_sequences=15	504	15
SDS-V3-plasma-67_Cluster_25460_sequences=15	504	15
SDS-V3-plasma-67_Cluster_1560_sequences=15	504	15
SDS-V3-plasma-67_Cluster_9027_sequences=15	504	15
SDS-V3-plasma-67_Cluster_13243_sequences=15	504	15
SDS-V3-plasma-67_Cluster_732_sequences=15	504	15
SDS-V3-plasma-67_Cluster_787_sequences=15	504	15
SDS-V3-plasma-67_Cluster_9723_sequences=15	504	15

SDS-V3-plasma-67_Cluster_2905_sequences=15	504	15
SDS-V3-plasma-67_Cluster_9772_sequences=15	504	15
SDS-V3-plasma-67_Cluster_9688_sequences=15	504	15
SDS-V3-plasma-67_Cluster_1616_sequences=15	504	15
SDS-V3-plasma-67_Cluster_4709_sequences=15	504	15
SDS-V3-plasma-67_Cluster_17833_sequences=15	504	15
SDS-V3-plasma-67_Cluster_8165_sequences=15	504	15
SDS-V3-plasma-67_Cluster_13607_sequences=15	504	15
SDS-V3-plasma-67_Cluster_28878_sequences=15	504	15
SDS-V3-plasma-67_Cluster_15389_sequences=15	504	15
SDS-V3-plasma-67_Cluster_31579_sequences=15	504	15
SDS-V3-plasma-67_Cluster_9805_sequences=15	504	15
SDS-V3-plasma-67_Cluster_4402_sequences=15	504	15
SDS-V3-plasma-67_Cluster_13095_sequences=15	504	15
SDS-V3-plasma-67_Cluster_4666_sequences=15	504	15
SDS-V3-plasma-67_Cluster_20632_sequences=15	504	15
SDS-V3-plasma-67_Cluster_2435_sequences=15	504	15
SDS-V3-plasma-67_Cluster_13595_sequences=15	504	15
SDS-V3-plasma-67_Cluster_3779_sequences=15	504	15
SDS-V3-plasma-67_Cluster_13209_sequences=15	504	15
SDS-V3-plasma-67_Cluster_24258_sequences=15	504	15
SDS-V3-plasma-67_Cluster_4642_sequences=15	504	15
SDS-V3-plasma-67_Cluster_4399_sequences=15	504	15
SDS-V3-plasma-67_Cluster_16146_sequences=15	504	15
SDS-V3-plasma-67_Cluster_22021_sequences=15	504	15
SDS-V3-plasma-67_Cluster_26483_sequences=15	504	15
SDS-V3-plasma-67_Cluster_10633_sequences=15	504	15
SDS-V3-plasma-67_Cluster_1554_sequences=15	504	15
SDS-V3-plasma-67_Cluster_25414_sequences=15	504	15
SDS-V3-plasma-67_Cluster_13098_sequences=15	504	15
SDS-V3-plasma-67_Cluster_1948_sequences=15	504	15
SDS-V3-plasma-67_Cluster_10992_sequences=15	504	15
SDS-V3-plasma-67_Cluster_23428_sequences=15	504	15
SDS-V3-PBMC-45_Cluster_24928_sequences=14	282	14
SDS-V3-plasma-0_Cluster_7232_sequences=14	0	14
SDS-V3-plasma-0_Cluster_728_sequences=14	0	14
SDS-V3-plasma-0_Cluster_3819_sequences=14	0	14
SDS-V3-plasma-0_Cluster_384_sequences=14	0	14
SDS-V3-plasma-0_Cluster_3649_sequences=14	0	14
SDS-V3-plasma-0_Cluster_14665_sequences=14	0	14
SDS-V3-plasma-0_Cluster_10270_sequences=14	0	14
SDS-V3-plasma-0_Cluster_1248_sequences=14	0	14
SDS-V3-plasma-0_Cluster_1330_sequences=14	0	14
SDS-V3-plasma-0_Cluster_1590_sequences=14	0	14
SDS-V3-plasma-0_Cluster_2083_sequences=14	0	14

SDS-V3-plasma-0_Cluster_3502_sequences=14	0	14
SDS-V3-plasma-0_Cluster_4050_sequences=14	0	14
SDS-V3-plasma-0_Cluster_4509_sequences=14	0	14
SDS-V3-plasma-0_Cluster_4843_sequences=14	0	14
SDS-V3-plasma-0_Cluster_4924_sequences=14	0	14
SDS-V3-plasma-0_Cluster_5985_sequences=14	0	14
SDS-V3-plasma-0_Cluster_730_sequences=14	0	14
SDS-V3-plasma-0_Cluster_2378_sequences=14	0	14
SDS-V3-plasma-0_Cluster_2003_sequences=14	0	14
SDS-V3-plasma-0_Cluster_13728_sequences=14	0	14
SDS-V3-plasma-0_Cluster_11289_sequences=14	0	14
SDS-V3-plasma-0_Cluster_2677_sequences=14	0	14
SDS-V3-plasma-0_Cluster_901_sequences=14	0	14
SDS-V3-plasma-0_Cluster_5140_sequences=14	0	14
SDS-V3-plasma-0_Cluster_12725_sequences=14	0	14
SDS-V3-plasma-0_Cluster_10058_sequences=14	0	14
SDS-V3-plasma-0_Cluster_2660_sequences=14	0	14
SDS-V3-plasma-0_Cluster_4101_sequences=14	0	14
SDS-V3-plasma-0_Cluster_8302_sequences=14	0	14
SDS-V3-plasma-0_Cluster_7042_sequences=14	0	14
SDS-V3-plasma-0_Cluster_1615_sequences=14	0	14
SDS-V3-plasma-0_Cluster_11166_sequences=14	0	14
SDS-V3-plasma-0_Cluster_1120_sequences=14	0	14
SDS-V3-plasma-0_Cluster_1692_sequences=14	0	14
SDS-V3-plasma-0_Cluster_2714_sequences=14	0	14
SDS-V3-plasma-0_Cluster_5588_sequences=14	0	14
SDS-V3-plasma-0_Cluster_685_sequences=14	0	14
SDS-V3-plasma-0_Cluster_9123_sequences=14	0	14
SDS-V3-plasma-0_Cluster_2420_sequences=14	0	14
SDS-V3-plasma-0_Cluster_722_sequences=14	0	14
SDS-V3-plasma-0_Cluster_4141_sequences=14	0	14
SDS-V3-plasma-0_Cluster_3107_sequences=14	0	14
SDS-V3-plasma-0_Cluster_11304_sequences=14	0	14
SDS-V3-plasma-0_Cluster_2216_sequences=14	0	14
SDS-V3-plasma-0_Cluster_9759_sequences=14	0	14
SDS-V3-plasma-0_Cluster_5828_sequences=14	0	14
SDS-V3-plasma-0_Cluster_3310_sequences=14	0	14
SDS-V3-plasma-0_Cluster_4315_sequences=14	0	14
SDS-V3-plasma-0_Cluster_8378_sequences=14	0	14
SDS-V3-plasma-0_Cluster_5188_sequences=14	0	14
SDS-V3-plasma-0_Cluster_2805_sequences=14	0	14
SDS-V3-plasma-0_Cluster_5149_sequences=14	0	14
SDS-V3-plasma-0_Cluster_2237_sequences=14	0	14
SDS-V3-plasma-0_Cluster_6487_sequences=14	0	14
SDS-V3-plasma-0_Cluster_4218_sequences=14	0	14

SDS-V3-plasma-5_Cluster_114_sequences=14	9	14
SDS-V3-plasma-5_Cluster_215_sequences=14	9	14
SDS-V3-plasma-5_Cluster_277_sequences=14	9	14
SDS-V3-plasma-5_Cluster_291_sequences=14	9	14
SDS-V3-plasma-5_Cluster_587_sequences=14	9	14
SDS-V3-plasma-5_Cluster_493_sequences=14	9	14
SDS-V3-plasma-5_Cluster_34_sequences=14	9	14
SDS-V3-plasma-5_Cluster_489_sequences=14	9	14
SDS-V3-plasma-5_Cluster_578_sequences=14	9	14
SDS-V3-plasma-5_Cluster_667_sequences=14	9	14
SDS-V3-plasma-5_Cluster_69_sequences=14	9	14
SDS-V3-plasma-5_Cluster_860_sequences=14	9	14
SDS-V3-plasma-7_Cluster_1109_sequences=14	14	14
SDS-V3-plasma-7_Cluster_1278_sequences=14	14	14
SDS-V3-plasma-7_Cluster_5569_sequences=14	14	14
SDS-V3-plasma-7_Cluster_3537_sequences=14	14	14
SDS-V3-plasma-7_Cluster_1479_sequences=14	14	14
SDS-V3-plasma-7_Cluster_327_sequences=14	14	14
SDS-V3-plasma-7_Cluster_1461_sequences=14	14	14
SDS-V3-plasma-7_Cluster_3627_sequences=14	14	14
SDS-V3-plasma-7_Cluster_1815_sequences=14	14	14
SDS-V3-plasma-7_Cluster_2181_sequences=14	14	14
SDS-V3-plasma-7_Cluster_2544_sequences=14	14	14
SDS-V3-plasma-7_Cluster_1026_sequences=14	14	14
SDS-V3-plasma-7_Cluster_122_sequences=14	14	14
SDS-V3-plasma-7_Cluster_1754_sequences=14	14	14
SDS-V3-plasma-7_Cluster_3172_sequences=14	14	14
SDS-V3-plasma-7_Cluster_403_sequences=14	14	14
SDS-V3-plasma-7_Cluster_8775_sequences=14	14	14
SDS-V3-plasma-7_Cluster_464_sequences=14	14	14
SDS-V3-plasma-7_Cluster_1401_sequences=14	14	14
SDS-V3-plasma-7_Cluster_362_sequences=14	14	14
SDS-V3-plasma-7_Cluster_2187_sequences=14	14	14
SDS-V3-plasma-7_Cluster_1140_sequences=14	14	14
SDS-V3-plasma-7_Cluster_9_sequences=14	14	14
SDS-V3-plasma-8_Cluster_67_sequences=14	16	14
SDS-V3-plasma-8_Cluster_83_sequences=14	16	14
SDS-V3-plasma-8_Cluster_99_sequences=14	16	14
SDS-V3-plasma-8_Cluster_254_sequences=14	16	14
SDS-V3-plasma-8_Cluster_380_sequences=14	16	14
SDS-V3-plasma-8_Cluster_3999_sequences=14	16	14
SDS-V3-plasma-8_Cluster_573_sequences=14	16	14
SDS-V3-plasma-8_Cluster_2838_sequences=14	16	14
SDS-V3-plasma-8_Cluster_2717_sequences=14	16	14
SDS-V3-plasma-8_Cluster_5331_sequences=14	16	14

SDS-V3-plasma-8_Cluster_2805_sequences=14	16	14
SDS-V3-plasma-8_Cluster_5411_sequences=14	16	14
SDS-V3-plasma-8_Cluster_2348_sequences=14	16	14
SDS-V3-plasma-8_Cluster_8834_sequences=14	16	14
SDS-V3-plasma-8_Cluster_2583_sequences=14	16	14
SDS-V3-plasma-8_Cluster_3766_sequences=14	16	14
SDS-V3-plasma-8_Cluster_3068_sequences=14	16	14
SDS-V3-plasma-8_Cluster_4677_sequences=14	16	14
SDS-V3-plasma-8_Cluster_6841_sequences=14	16	14
SDS-V3-plasma-8_Cluster_3045_sequences=14	16	14
SDS-V3-plasma-8_Cluster_4591_sequences=14	16	14
SDS-V3-plasma-8_Cluster_2514_sequences=14	16	14
SDS-V3-plasma-8_Cluster_3901_sequences=14	16	14
SDS-V3-plasma-8_Cluster_175_sequences=14	16	14
SDS-V3-plasma-8_Cluster_180_sequences=14	16	14
SDS-V3-plasma-8_Cluster_3544_sequences=14	16	14
SDS-V3-plasma-24_Cluster_1062_sequences=14	124	14
SDS-V3-plasma-24_Cluster_1918_sequences=14	124	14
SDS-V3-plasma-24_Cluster_3046_sequences=14	124	14
SDS-V3-plasma-24_Cluster_356_sequences=14	124	14
SDS-V3-plasma-24_Cluster_2504_sequences=14	124	14
SDS-V3-plasma-24_Cluster_3712_sequences=14	124	14
SDS-V3-plasma-24_Cluster_1585_sequences=14	124	14
SDS-V3-plasma-24_Cluster_2844_sequences=14	124	14
SDS-V3-plasma-24_Cluster_4751_sequences=14	124	14
SDS-V3-plasma-24_Cluster_1065_sequences=14	124	14
SDS-V3-plasma-24_Cluster_157_sequences=14	124	14
SDS-V3-plasma-24_Cluster_84_sequences=14	124	14
SDS-V3-plasma-24_Cluster_3912_sequences=14	124	14
SDS-V3-plasma-24_Cluster_468_sequences=14	124	14
SDS-V3-plasma-24_Cluster_8205_sequences=14	124	14
SDS-V3-plasma-24_Cluster_1847_sequences=14	124	14
SDS-V3-plasma-24_Cluster_5956_sequences=14	124	14
SDS-V3-plasma-24_Cluster_373_sequences=14	124	14
SDS-V3-plasma-24_Cluster_318_sequences=14	124	14
SDS-V3-plasma-27_Cluster_202_sequences=14	131	14
SDS-V3-plasma-27_Cluster_1047_sequences=14	131	14
SDS-V3-plasma-27_Cluster_1444_sequences=14	131	14
SDS-V3-plasma-27_Cluster_1518_sequences=14	131	14
SDS-V3-plasma-27_Cluster_1775_sequences=14	131	14
SDS-V3-plasma-27_Cluster_2475_sequences=14	131	14
SDS-V3-plasma-27_Cluster_384_sequences=14	131	14
SDS-V3-plasma-27_Cluster_127_sequences=14	131	14
SDS-V3-plasma-27_Cluster_1713_sequences=14	131	14
SDS-V3-plasma-27_Cluster_4025_sequences=14	131	14

SDS-V3-plasma-27_Cluster_1343_sequences=14	131	14
SDS-V3-plasma-27_Cluster_3678_sequences=14	131	14
SDS-V3-plasma-27_Cluster_4715_sequences=14	131	14
SDS-V3-plasma-27_Cluster_2743_sequences=14	131	14
SDS-V3-plasma-27_Cluster_2873_sequences=14	131	14
SDS-V3-plasma-27_Cluster_3426_sequences=14	131	14
SDS-V3-plasma-27_Cluster_128_sequences=14	131	14
SDS-V3-plasma-27_Cluster_2742_sequences=14	131	14
SDS-V3-plasma-27_Cluster_453_sequences=14	131	14
SDS-V3-plasma-27_Cluster_5796_sequences=14	131	14
SDS-V3-plasma-27_Cluster_1469_sequences=14	131	14
SDS-V3-plasma-27_Cluster_3417_sequences=14	131	14
SDS-V3-plasma-27_Cluster_1686_sequences=14	131	14
SDS-V3-plasma-27_Cluster_505_sequences=14	131	14
SDS-V3-plasma-27_Cluster_3560_sequences=14	131	14
SDS-V3-plasma-27_Cluster_2674_sequences=14	131	14
SDS-V3-plasma-45_Cluster_10206_sequences=14	282	14
SDS-V3-plasma-45_Cluster_11351_sequences=14	282	14
SDS-V3-plasma-45_Cluster_1753_sequences=14	282	14
SDS-V3-plasma-45_Cluster_2100_sequences=14	282	14
SDS-V3-plasma-45_Cluster_5738_sequences=14	282	14
SDS-V3-plasma-45_Cluster_815_sequences=14	282	14
SDS-V3-plasma-45_Cluster_14963_sequences=14	282	14
SDS-V3-plasma-45_Cluster_8607_sequences=14	282	14
SDS-V3-plasma-45_Cluster_20419_sequences=14	282	14
SDS-V3-plasma-45_Cluster_24785_sequences=14	282	14
SDS-V3-plasma-45_Cluster_203_sequences=14	282	14
SDS-V3-plasma-45_Cluster_10012_sequences=14	282	14
SDS-V3-plasma-45_Cluster_14930_sequences=14	282	14
SDS-V3-plasma-45_Cluster_31904_sequences=14	282	14
SDS-V3-plasma-45_Cluster_13521_sequences=14	282	14
SDS-V3-plasma-45_Cluster_2288_sequences=14	282	14
SDS-V3-plasma-45_Cluster_3823_sequences=14	282	14
SDS-V3-plasma-45_Cluster_4391_sequences=14	282	14
SDS-V3-plasma-45_Cluster_16869_sequences=14	282	14
SDS-V3-plasma-45_Cluster_8901_sequences=14	282	14
SDS-V3-plasma-45_Cluster_13271_sequences=14	282	14
SDS-V3-plasma-45_Cluster_8858_sequences=14	282	14
SDS-V3-plasma-45_Cluster_43833_sequences=14	282	14
SDS-V3-plasma-45_Cluster_13233_sequences=14	282	14
SDS-V3-plasma-45_Cluster_31680_sequences=14	282	14
SDS-V3-plasma-45_Cluster_7481_sequences=14	282	14
SDS-V3-plasma-45_Cluster_11979_sequences=14	282	14
SDS-V3-plasma-45_Cluster_12300_sequences=14	282	14
SDS-V3-plasma-45_Cluster_12917_sequences=14	282	14

SDS-V3-plasma-45_Cluster_13910_sequences=14	282	14
SDS-V3-plasma-45_Cluster_166_sequences=14	282	14
SDS-V3-plasma-45_Cluster_1782_sequences=14	282	14
SDS-V3-plasma-45_Cluster_20000_sequences=14	282	14
SDS-V3-plasma-45_Cluster_2915_sequences=14	282	14
SDS-V3-plasma-45_Cluster_29614_sequences=14	282	14
SDS-V3-plasma-45_Cluster_31483_sequences=14	282	14
SDS-V3-plasma-45_Cluster_35223_sequences=14	282	14
SDS-V3-plasma-45_Cluster_46_sequences=14	282	14
SDS-V3-plasma-45_Cluster_52670_sequences=14	282	14
SDS-V3-plasma-45_Cluster_5278_sequences=14	282	14
SDS-V3-plasma-45_Cluster_6476_sequences=14	282	14
SDS-V3-plasma-45_Cluster_6533_sequences=14	282	14
SDS-V3-plasma-45_Cluster_9952_sequences=14	282	14
SDS-V3-plasma-45_Cluster_25703_sequences=14	282	14
SDS-V3-plasma-45_Cluster_4429_sequences=14	282	14
SDS-V3-plasma-45_Cluster_24348_sequences=14	282	14
SDS-V3-plasma-45_Cluster_15810_sequences=14	282	14
SDS-V3-plasma-45_Cluster_16767_sequences=14	282	14
SDS-V3-plasma-45_Cluster_17031_sequences=14	282	14
SDS-V3-plasma-45_Cluster_18409_sequences=14	282	14
SDS-V3-plasma-45_Cluster_652_sequences=14	282	14
SDS-V3-plasma-45_Cluster_8355_sequences=14	282	14
SDS-V3-plasma-45_Cluster_9198_sequences=14	282	14
SDS-V3-plasma-45_Cluster_9258_sequences=14	282	14
SDS-V3-plasma-45_Cluster_11114_sequences=14	282	14
SDS-V3-plasma-45_Cluster_24093_sequences=14	282	14
SDS-V3-plasma-45_Cluster_5898_sequences=14	282	14
SDS-V3-plasma-45_Cluster_27330_sequences=14	282	14
SDS-V3-plasma-45_Cluster_28161_sequences=14	282	14
SDS-V3-plasma-45_Cluster_4580_sequences=14	282	14
SDS-V3-plasma-45_Cluster_7078_sequences=14	282	14
SDS-V3-plasma-45_Cluster_826_sequences=14	282	14
SDS-V3-plasma-45_Cluster_9024_sequences=14	282	14
SDS-V3-plasma-45_Cluster_11871_sequences=14	282	14
SDS-V3-plasma-45_Cluster_13950_sequences=14	282	14
SDS-V3-plasma-45_Cluster_558_sequences=14	282	14
SDS-V3-plasma-45_Cluster_17586_sequences=14	282	14
SDS-V3-plasma-45_Cluster_3960_sequences=14	282	14
SDS-V3-plasma-45_Cluster_13884_sequences=14	282	14
SDS-V3-plasma-45_Cluster_14391_sequences=14	282	14
SDS-V3-plasma-45_Cluster_16021_sequences=14	282	14
SDS-V3-plasma-45_Cluster_19110_sequences=14	282	14
SDS-V3-plasma-45_Cluster_22549_sequences=14	282	14
SDS-V3-plasma-45_Cluster_23316_sequences=14	282	14

SDS-V3-plasma-45_Cluster_23898_sequences=14	282	14
SDS-V3-plasma-45_Cluster_32629_sequences=14	282	14
SDS-V3-plasma-45_Cluster_37298_sequences=14	282	14
SDS-V3-plasma-45_Cluster_7348_sequences=14	282	14
SDS-V3-plasma-45_Cluster_6112_sequences=14	282	14
SDS-V3-plasma-45_Cluster_11160_sequences=14	282	14
SDS-V3-plasma-45_Cluster_11003_sequences=14	282	14
SDS-V3-plasma-45_Cluster_9923_sequences=14	282	14
SDS-V3-plasma-45_Cluster_23380_sequences=14	282	14
SDS-V3-plasma-45_Cluster_10964_sequences=14	282	14
SDS-V3-plasma-45_Cluster_19661_sequences=14	282	14
SDS-V3-plasma-45_Cluster_26113_sequences=14	282	14
SDS-V3-plasma-45_Cluster_15251_sequences=14	282	14
SDS-V3-plasma-45_Cluster_5440_sequences=14	282	14
SDS-V3-plasma-45_Cluster_28784_sequences=14	282	14
SDS-V3-plasma-45_Cluster_11716_sequences=14	282	14
SDS-V3-plasma-45_Cluster_16931_sequences=14	282	14
SDS-V3-plasma-45_Cluster_19280_sequences=14	282	14
SDS-V3-plasma-45_Cluster_5552_sequences=14	282	14
SDS-V3-plasma-45_Cluster_580_sequences=14	282	14
SDS-V3-plasma-45_Cluster_1008_sequences=14	282	14
SDS-V3-plasma-45_Cluster_2382_sequences=14	282	14
SDS-V3-plasma-45_Cluster_22368_sequences=14	282	14
SDS-V3-plasma-45_Cluster_4723_sequences=14	282	14
SDS-V3-plasma-45_Cluster_12531_sequences=14	282	14
SDS-V3-plasma-45_Cluster_11785_sequences=14	282	14
SDS-V3-plasma-45_Cluster_12537_sequences=14	282	14
SDS-V3-plasma-45_Cluster_3279_sequences=14	282	14
SDS-V3-plasma-45_Cluster_5792_sequences=14	282	14
SDS-V3-plasma-45_Cluster_66669_sequences=14	282	14
SDS-V3-plasma-45_Cluster_6932_sequences=14	282	14
SDS-V3-plasma-45_Cluster_6854_sequences=14	282	14
SDS-V3-plasma-45_Cluster_1227_sequences=14	282	14
SDS-V3-plasma-45_Cluster_14100_sequences=14	282	14
SDS-V3-plasma-45_Cluster_1529_sequences=14	282	14
SDS-V3-plasma-45_Cluster_13148_sequences=14	282	14
SDS-V3-plasma-45_Cluster_6785_sequences=14	282	14
SDS-V3-plasma-45_Cluster_16574_sequences=14	282	14
SDS-V3-plasma-45_Cluster_4337_sequences=14	282	14
SDS-V3-plasma-45_Cluster_210_sequences=14	282	14
SDS-V3-plasma-45_Cluster_18269_sequences=14	282	14
SDS-V3-plasma-45_Cluster_70127_sequences=14	282	14
SDS-V3-plasma-45_Cluster_12295_sequences=14	282	14
SDS-V3-plasma-45_Cluster_11207_sequences=14	282	14
SDS-V3-plasma-45_Cluster_30124_sequences=14	282	14

SDS-V3-plasma-45_Cluster_6262_sequences=14	282	14
SDS-V3-plasma-45_Cluster_3465_sequences=14	282	14
SDS-V3-plasma-45_Cluster_5267_sequences=14	282	14
SDS-V3-plasma-45_Cluster_5757_sequences=14	282	14
SDS-V3-plasma-45_Cluster_11381_sequences=14	282	14
SDS-V3-plasma-45_Cluster_18264_sequences=14	282	14
SDS-V3-plasma-45_Cluster_10422_sequences=14	282	14
SDS-V3-plasma-45_Cluster_19107_sequences=14	282	14
SDS-V3-plasma-45_Cluster_31758_sequences=14	282	14
SDS-V3-plasma-45_Cluster_10601_sequences=14	282	14
SDS-V3-plasma-45_Cluster_19127_sequences=14	282	14
SDS-V3-plasma-45_Cluster_13693_sequences=14	282	14
SDS-V3-plasma-45_Cluster_21112_sequences=14	282	14
SDS-V3-plasma-45_Cluster_5304_sequences=14	282	14
SDS-V3-plasma-45_Cluster_15510_sequences=14	282	14
SDS-V3-plasma-45_Cluster_16377_sequences=14	282	14
SDS-V3-plasma-45_Cluster_216_sequences=14	282	14
SDS-V3-plasma-45_Cluster_5690_sequences=14	282	14
SDS-V3-plasma-45_Cluster_20257_sequences=14	282	14
SDS-V3-plasma-45_Cluster_9143_sequences=14	282	14
SDS-V3-plasma-45_Cluster_6062_sequences=14	282	14
SDS-V3-plasma-45_Cluster_26238_sequences=14	282	14
SDS-V3-plasma-45_Cluster_7729_sequences=14	282	14
SDS-V3-plasma-45_Cluster_4482_sequences=14	282	14
SDS-V3-plasma-45_Cluster_4895_sequences=14	282	14
SDS-V3-plasma-45_Cluster_6765_sequences=14	282	14
SDS-V3-plasma-45_Cluster_7473_sequences=14	282	14
SDS-V3-plasma-45_Cluster_5197_sequences=14	282	14
SDS-V3-plasma-45_Cluster_12228_sequences=14	282	14
SDS-V3-plasma-45_Cluster_5954_sequences=14	282	14
SDS-V3-plasma-45_Cluster_1814_sequences=14	282	14
SDS-V3-plasma-45_Cluster_29857_sequences=14	282	14
SDS-V3-plasma-45_Cluster_10452_sequences=14	282	14
SDS-V3-plasma-45_Cluster_15863_sequences=14	282	14
SDS-V3-plasma-45_Cluster_4816_sequences=14	282	14
SDS-V3-plasma-45_Cluster_1955_sequences=14	282	14
SDS-V3-plasma-45_Cluster_778_sequences=14	282	14
SDS-V3-plasma-45_Cluster_30694_sequences=14	282	14
SDS-V3-plasma-45_Cluster_5795_sequences=14	282	14
SDS-V3-plasma-45_Cluster_3485_sequences=14	282	14
SDS-V3-plasma-46_Cluster_4939_sequences=14	286	14
SDS-V3-plasma-46_Cluster_2471_sequences=14	286	14
SDS-V3-plasma-46_Cluster_4381_sequences=14	286	14
SDS-V3-plasma-46_Cluster_5326_sequences=14	286	14
SDS-V3-plasma-46_Cluster_6339_sequences=14	286	14

SDS-V3-plasma-46_Cluster_735_sequences=14	286	14
SDS-V3-plasma-46_Cluster_2079_sequences=14	286	14
SDS-V3-plasma-46_Cluster_1847_sequences=14	286	14
SDS-V3-plasma-46_Cluster_1192_sequences=14	286	14
SDS-V3-plasma-46_Cluster_8875_sequences=14	286	14
SDS-V3-plasma-46_Cluster_2379_sequences=14	286	14
SDS-V3-plasma-46_Cluster_4415_sequences=14	286	14
SDS-V3-plasma-46_Cluster_10694_sequences=14	286	14
SDS-V3-plasma-46_Cluster_7946_sequences=14	286	14
SDS-V3-plasma-46_Cluster_2108_sequences=14	286	14
SDS-V3-plasma-46_Cluster_242_sequences=14	286	14
SDS-V3-plasma-46_Cluster_1766_sequences=14	286	14
SDS-V3-plasma-46_Cluster_11724_sequences=14	286	14
SDS-V3-plasma-46_Cluster_6440_sequences=14	286	14
SDS-V3-plasma-46_Cluster_8239_sequences=14	286	14
SDS-V3-plasma-46_Cluster_8559_sequences=14	286	14
SDS-V3-plasma-46_Cluster_14691_sequences=14	286	14
SDS-V3-plasma-46_Cluster_1760_sequences=14	286	14
SDS-V3-plasma-46_Cluster_3445_sequences=14	286	14
SDS-V3-plasma-46_Cluster_3458_sequences=14	286	14
SDS-V3-plasma-46_Cluster_9221_sequences=14	286	14
SDS-V3-plasma-46_Cluster_2066_sequences=14	286	14
SDS-V3-plasma-46_Cluster_1351_sequences=14	286	14
SDS-V3-plasma-46_Cluster_1299_sequences=14	286	14
SDS-V3-plasma-46_Cluster_10394_sequences=14	286	14
SDS-V3-plasma-46_Cluster_1318_sequences=14	286	14
SDS-V3-plasma-46_Cluster_1457_sequences=14	286	14
SDS-V3-plasma-46_Cluster_255_sequences=14	286	14
SDS-V3-plasma-46_Cluster_3091_sequences=14	286	14
SDS-V3-plasma-46_Cluster_562_sequences=14	286	14
SDS-V3-plasma-46_Cluster_3918_sequences=14	286	14
SDS-V3-plasma-46_Cluster_11355_sequences=14	286	14
SDS-V3-plasma-46_Cluster_11_sequences=14	286	14
SDS-V3-plasma-46_Cluster_1371_sequences=14	286	14
SDS-V3-plasma-46_Cluster_2551_sequences=14	286	14
SDS-V3-plasma-46_Cluster_5589_sequences=14	286	14
SDS-V3-plasma-46_Cluster_5823_sequences=14	286	14
SDS-V3-plasma-46_Cluster_6324_sequences=14	286	14
SDS-V3-plasma-46_Cluster_11822_sequences=14	286	14
SDS-V3-plasma-46_Cluster_1389_sequences=14	286	14
SDS-V3-plasma-46_Cluster_2054_sequences=14	286	14
SDS-V3-plasma-46_Cluster_5419_sequences=14	286	14
SDS-V3-plasma-46_Cluster_1025_sequences=14	286	14
SDS-V3-plasma-46_Cluster_2808_sequences=14	286	14
SDS-V3-plasma-46_Cluster_253_sequences=14	286	14

SDS-V3-plasma-46_Cluster_331_sequences=14	286	14
SDS-V3-plasma-46_Cluster_512_sequences=14	286	14
SDS-V3-plasma-46_Cluster_9440_sequences=14	286	14
SDS-V3-plasma-46_Cluster_28622_sequences=14	286	14
SDS-V3-plasma-46_Cluster_3027_sequences=14	286	14
SDS-V3-plasma-46_Cluster_3380_sequences=14	286	14
SDS-V3-plasma-46_Cluster_3716_sequences=14	286	14
SDS-V3-plasma-46_Cluster_1139_sequences=14	286	14
SDS-V3-plasma-46_Cluster_3259_sequences=14	286	14
SDS-V3-plasma-46_Cluster_3253_sequences=14	286	14
SDS-V3-plasma-46_Cluster_4858_sequences=14	286	14
SDS-V3-plasma-46_Cluster_3201_sequences=14	286	14
SDS-V3-plasma-46_Cluster_1419_sequences=14	286	14
SDS-V3-plasma-46_Cluster_4237_sequences=14	286	14
SDS-V3-plasma-46_Cluster_2125_sequences=14	286	14
SDS-V3-plasma-46_Cluster_5603_sequences=14	286	14
SDS-V3-plasma-67_Cluster_16095_sequences=14	504	14
SDS-V3-plasma-67_Cluster_11890_sequences=14	504	14
SDS-V3-plasma-67_Cluster_1275_sequences=14	504	14
SDS-V3-plasma-67_Cluster_6972_sequences=14	504	14
SDS-V3-plasma-67_Cluster_11144_sequences=14	504	14
SDS-V3-plasma-67_Cluster_22096_sequences=14	504	14
SDS-V3-plasma-67_Cluster_4301_sequences=14	504	14
SDS-V3-plasma-67_Cluster_18126_sequences=14	504	14
SDS-V3-plasma-67_Cluster_25850_sequences=14	504	14
SDS-V3-plasma-67_Cluster_52127_sequences=14	504	14
SDS-V3-plasma-67_Cluster_2397_sequences=14	504	14
SDS-V3-plasma-67_Cluster_15031_sequences=14	504	14
SDS-V3-plasma-67_Cluster_9082_sequences=14	504	14
SDS-V3-plasma-67_Cluster_9515_sequences=14	504	14
SDS-V3-plasma-67_Cluster_7525_sequences=14	504	14
SDS-V3-plasma-67_Cluster_3317_sequences=14	504	14
SDS-V3-plasma-67_Cluster_47955_sequences=14	504	14
SDS-V3-plasma-67_Cluster_6912_sequences=14	504	14
SDS-V3-plasma-67_Cluster_39220_sequences=14	504	14
SDS-V3-plasma-67_Cluster_11942_sequences=14	504	14
SDS-V3-plasma-67_Cluster_10231_sequences=14	504	14
SDS-V3-plasma-67_Cluster_694_sequences=14	504	14
SDS-V3-plasma-67_Cluster_22217_sequences=14	504	14
SDS-V3-plasma-67_Cluster_10915_sequences=14	504	14
SDS-V3-plasma-67_Cluster_4557_sequences=14	504	14
SDS-V3-plasma-67_Cluster_24646_sequences=14	504	14
SDS-V3-plasma-67_Cluster_26322_sequences=14	504	14
SDS-V3-plasma-67_Cluster_7513_sequences=14	504	14
SDS-V3-plasma-67_Cluster_12725_sequences=14	504	14

SDS-V3-plasma-67_Cluster_6375_sequences=14	504	14
SDS-V3-plasma-67_Cluster_64677_sequences=14	504	14
SDS-V3-plasma-67_Cluster_23626_sequences=14	504	14
SDS-V3-plasma-67_Cluster_4128_sequences=14	504	14
SDS-V3-plasma-67_Cluster_27307_sequences=14	504	14
SDS-V3-plasma-67_Cluster_11017_sequences=14	504	14
SDS-V3-plasma-67_Cluster_3269_sequences=14	504	14
SDS-V3-plasma-67_Cluster_9850_sequences=14	504	14
SDS-V3-plasma-67_Cluster_3795_sequences=14	504	14
SDS-V3-plasma-67_Cluster_18781_sequences=14	504	14
SDS-V3-plasma-67_Cluster_15227_sequences=14	504	14
SDS-V3-plasma-67_Cluster_11597_sequences=14	504	14
SDS-V3-plasma-67_Cluster_21502_sequences=14	504	14
SDS-V3-plasma-67_Cluster_9106_sequences=14	504	14
SDS-V3-plasma-67_Cluster_16464_sequences=14	504	14
SDS-V3-plasma-67_Cluster_23266_sequences=14	504	14
SDS-V3-plasma-67_Cluster_28923_sequences=14	504	14
SDS-V3-plasma-67_Cluster_33343_sequences=14	504	14
SDS-V3-plasma-67_Cluster_336_sequences=14	504	14
SDS-V3-plasma-67_Cluster_4363_sequences=14	504	14
SDS-V3-plasma-67_Cluster_9396_sequences=14	504	14
SDS-V3-plasma-67_Cluster_4563_sequences=14	504	14
SDS-V3-plasma-67_Cluster_10339_sequences=14	504	14
SDS-V3-plasma-67_Cluster_13658_sequences=14	504	14
SDS-V3-plasma-67_Cluster_13802_sequences=14	504	14
SDS-V3-plasma-67_Cluster_14331_sequences=14	504	14
SDS-V3-plasma-67_Cluster_15142_sequences=14	504	14
SDS-V3-plasma-67_Cluster_15627_sequences=14	504	14
SDS-V3-plasma-67_Cluster_2374_sequences=14	504	14
SDS-V3-plasma-67_Cluster_2855_sequences=14	504	14
SDS-V3-plasma-67_Cluster_404_sequences=14	504	14
SDS-V3-plasma-67_Cluster_5622_sequences=14	504	14
SDS-V3-plasma-67_Cluster_5682_sequences=14	504	14
SDS-V3-plasma-67_Cluster_6151_sequences=14	504	14
SDS-V3-plasma-67_Cluster_6478_sequences=14	504	14
SDS-V3-plasma-67_Cluster_8510_sequences=14	504	14
SDS-V3-plasma-67_Cluster_177_sequences=14	504	14
SDS-V3-plasma-67_Cluster_15044_sequences=14	504	14
SDS-V3-plasma-67_Cluster_12581_sequences=14	504	14
SDS-V3-plasma-67_Cluster_17341_sequences=14	504	14
SDS-V3-plasma-67_Cluster_17772_sequences=14	504	14
SDS-V3-plasma-67_Cluster_37887_sequences=14	504	14
SDS-V3-plasma-67_Cluster_12278_sequences=14	504	14
SDS-V3-plasma-67_Cluster_696_sequences=14	504	14
SDS-V3-plasma-67_Cluster_12069_sequences=14	504	14

SDS-V3-plasma-67_Cluster_16731_sequences=14	504	14
SDS-V3-plasma-67_Cluster_7321_sequences=14	504	14
SDS-V3-plasma-67_Cluster_5491_sequences=14	504	14
SDS-V3-plasma-67_Cluster_22393_sequences=14	504	14
SDS-V3-plasma-67_Cluster_7558_sequences=14	504	14
SDS-V3-plasma-67_Cluster_21459_sequences=14	504	14
SDS-V3-plasma-67_Cluster_8522_sequences=14	504	14
SDS-V3-plasma-67_Cluster_4851_sequences=14	504	14
SDS-V3-plasma-67_Cluster_12127_sequences=14	504	14
SDS-V3-plasma-67_Cluster_9553_sequences=14	504	14
SDS-V3-plasma-67_Cluster_23900_sequences=14	504	14
SDS-V3-plasma-67_Cluster_9487_sequences=14	504	14
SDS-V3-plasma-67_Cluster_3334_sequences=14	504	14
SDS-V3-plasma-67_Cluster_1508_sequences=14	504	14
SDS-V3-plasma-67_Cluster_12135_sequences=14	504	14
SDS-V3-plasma-67_Cluster_257_sequences=14	504	14
SDS-V3-plasma-67_Cluster_23363_sequences=14	504	14
SDS-V3-plasma-67_Cluster_2220_sequences=14	504	14
SDS-V3-plasma-67_Cluster_5573_sequences=14	504	14
SDS-V3-plasma-67_Cluster_5922_sequences=14	504	14
SDS-V3-plasma-67_Cluster_13138_sequences=14	504	14
SDS-V3-plasma-67_Cluster_24971_sequences=14	504	14
SDS-V3-plasma-67_Cluster_9945_sequences=14	504	14
SDS-V3-plasma-67_Cluster_10625_sequences=14	504	14
SDS-V3-plasma-67_Cluster_13199_sequences=14	504	14
SDS-V3-plasma-67_Cluster_22522_sequences=14	504	14
SDS-V3-plasma-67_Cluster_3784_sequences=14	504	14
SDS-V3-plasma-67_Cluster_11305_sequences=14	504	14
SDS-V3-plasma-67_Cluster_2411_sequences=14	504	14
SDS-V3-plasma-67_Cluster_42631_sequences=14	504	14
SDS-V3-plasma-67_Cluster_8938_sequences=14	504	14
SDS-V3-plasma-67_Cluster_14926_sequences=14	504	14
SDS-V3-plasma-0_Cluster_5965_sequences=13	0	13
SDS-V3-plasma-0_Cluster_524_sequences=13	0	13
SDS-V3-plasma-0_Cluster_1935_sequences=13	0	13
SDS-V3-plasma-0_Cluster_1473_sequences=13	0	13
SDS-V3-plasma-0_Cluster_15139_sequences=13	0	13
SDS-V3-plasma-0_Cluster_1911_sequences=13	0	13
SDS-V3-plasma-0_Cluster_1969_sequences=13	0	13
SDS-V3-plasma-0_Cluster_2696_sequences=13	0	13
SDS-V3-plasma-0_Cluster_298_sequences=13	0	13
SDS-V3-plasma-0_Cluster_3714_sequences=13	0	13
SDS-V3-plasma-0_Cluster_3722_sequences=13	0	13
SDS-V3-plasma-0_Cluster_4250_sequences=13	0	13
SDS-V3-plasma-0_Cluster_545_sequences=13	0	13

SDS-V3-plasma-0_Cluster_6830_sequences=13	0	13
SDS-V3-plasma-0_Cluster_7573_sequences=13	0	13
SDS-V3-plasma-0_Cluster_791_sequences=13	0	13
SDS-V3-plasma-0_Cluster_9895_sequences=13	0	13
SDS-V3-plasma-0_Cluster_6187_sequences=13	0	13
SDS-V3-plasma-0_Cluster_7488_sequences=13	0	13
SDS-V3-plasma-0_Cluster_3001_sequences=13	0	13
SDS-V3-plasma-0_Cluster_2321_sequences=13	0	13
SDS-V3-plasma-0_Cluster_1689_sequences=13	0	13
SDS-V3-plasma-0_Cluster_6896_sequences=13	0	13
SDS-V3-plasma-0_Cluster_959_sequences=13	0	13
SDS-V3-plasma-0_Cluster_6901_sequences=13	0	13
SDS-V3-plasma-0_Cluster_5287_sequences=13	0	13
SDS-V3-plasma-0_Cluster_4806_sequences=13	0	13
SDS-V3-plasma-0_Cluster_8414_sequences=13	0	13
SDS-V3-plasma-0_Cluster_8891_sequences=13	0	13
SDS-V3-plasma-0_Cluster_8245_sequences=13	0	13
SDS-V3-plasma-0_Cluster_4450_sequences=13	0	13
SDS-V3-plasma-0_Cluster_3592_sequences=13	0	13
SDS-V3-plasma-0_Cluster_3328_sequences=13	0	13
SDS-V3-plasma-0_Cluster_1600_sequences=13	0	13
SDS-V3-plasma-0_Cluster_1304_sequences=13	0	13
SDS-V3-plasma-0_Cluster_1324_sequences=13	0	13
SDS-V3-plasma-0_Cluster_1539_sequences=13	0	13
SDS-V3-plasma-0_Cluster_2058_sequences=13	0	13
SDS-V3-plasma-0_Cluster_2595_sequences=13	0	13
SDS-V3-plasma-0_Cluster_327_sequences=13	0	13
SDS-V3-plasma-0_Cluster_5976_sequences=13	0	13
SDS-V3-plasma-0_Cluster_1745_sequences=13	0	13
SDS-V3-plasma-0_Cluster_4274_sequences=13	0	13
SDS-V3-plasma-0_Cluster_9225_sequences=13	0	13
SDS-V3-plasma-0_Cluster_5351_sequences=13	0	13
SDS-V3-plasma-0_Cluster_24827_sequences=13	0	13
SDS-V3-plasma-0_Cluster_10539_sequences=13	0	13
SDS-V3-plasma-0_Cluster_3427_sequences=13	0	13
SDS-V3-plasma-0_Cluster_1624_sequences=13	0	13
SDS-V3-plasma-0_Cluster_3522_sequences=13	0	13
SDS-V3-plasma-0_Cluster_1141_sequences=13	0	13
SDS-V3-plasma-0_Cluster_215_sequences=13	0	13
SDS-V3-plasma-0_Cluster_4540_sequences=13	0	13
SDS-V3-plasma-0_Cluster_1839_sequences=13	0	13
SDS-V3-plasma-0_Cluster_4747_sequences=13	0	13
SDS-V3-plasma-0_Cluster_42080_sequences=13	0	13
SDS-V3-plasma-5_Cluster_35_sequences=13	9	13
SDS-V3-plasma-5_Cluster_54_sequences=13	9	13

SDS-V3-plasma-5_Cluster_554_sequences=13	9	13
SDS-V3-plasma-5_Cluster_972_sequences=13	9	13
SDS-V3-plasma-5_Cluster_200_sequences=13	9	13
SDS-V3-plasma-5_Cluster_120_sequences=13	9	13
SDS-V3-plasma-5_Cluster_146_sequences=13	9	13
SDS-V3-plasma-5_Cluster_478_sequences=13	9	13
SDS-V3-plasma-5_Cluster_711_sequences=13	9	13
SDS-V3-plasma-7_Cluster_1183_sequences=13	14	13
SDS-V3-plasma-7_Cluster_2174_sequences=13	14	13
SDS-V3-plasma-7_Cluster_507_sequences=13	14	13
SDS-V3-plasma-7_Cluster_2526_sequences=13	14	13
SDS-V3-plasma-7_Cluster_1040_sequences=13	14	13
SDS-V3-plasma-7_Cluster_1226_sequences=13	14	13
SDS-V3-plasma-7_Cluster_155_sequences=13	14	13
SDS-V3-plasma-7_Cluster_1598_sequences=13	14	13
SDS-V3-plasma-7_Cluster_1013_sequences=13	14	13
SDS-V3-plasma-7_Cluster_1276_sequences=13	14	13
SDS-V3-plasma-7_Cluster_3531_sequences=13	14	13
SDS-V3-plasma-7_Cluster_738_sequences=13	14	13
SDS-V3-plasma-7_Cluster_1803_sequences=13	14	13
SDS-V3-plasma-7_Cluster_117_sequences=13	14	13
SDS-V3-plasma-7_Cluster_1356_sequences=13	14	13
SDS-V3-plasma-8_Cluster_177_sequences=13	16	13
SDS-V3-plasma-8_Cluster_580_sequences=13	16	13
SDS-V3-plasma-8_Cluster_5743_sequences=13	16	13
SDS-V3-plasma-8_Cluster_2860_sequences=13	16	13
SDS-V3-plasma-8_Cluster_2765_sequences=13	16	13
SDS-V3-plasma-8_Cluster_3352_sequences=13	16	13
SDS-V3-plasma-8_Cluster_2311_sequences=13	16	13
SDS-V3-plasma-8_Cluster_2530_sequences=13	16	13
SDS-V3-plasma-8_Cluster_2403_sequences=13	16	13
SDS-V3-plasma-8_Cluster_3983_sequences=13	16	13
SDS-V3-plasma-8_Cluster_4133_sequences=13	16	13
SDS-V3-plasma-8_Cluster_4536_sequences=13	16	13
SDS-V3-plasma-8_Cluster_3321_sequences=13	16	13
SDS-V3-plasma-8_Cluster_4819_sequences=13	16	13
SDS-V3-plasma-8_Cluster_2329_sequences=13	16	13
SDS-V3-plasma-8_Cluster_3036_sequences=13	16	13
SDS-V3-plasma-8_Cluster_3728_sequences=13	16	13
SDS-V3-plasma-8_Cluster_3768_sequences=13	16	13
SDS-V3-plasma-8_Cluster_5150_sequences=13	16	13
SDS-V3-plasma-8_Cluster_2916_sequences=13	16	13
SDS-V3-plasma-8_Cluster_2926_sequences=13	16	13
SDS-V3-plasma-8_Cluster_4256_sequences=13	16	13
SDS-V3-plasma-8_Cluster_2335_sequences=13	16	13

SDS-V3-plasma-24_Cluster_1229_sequences=13	124	13
SDS-V3-plasma-24_Cluster_31_sequences=13	124	13
SDS-V3-plasma-24_Cluster_421_sequences=13	124	13
SDS-V3-plasma-24_Cluster_1718_sequences=13	124	13
SDS-V3-plasma-24_Cluster_2890_sequences=13	124	13
SDS-V3-plasma-24_Cluster_424_sequences=13	124	13
SDS-V3-plasma-24_Cluster_2199_sequences=13	124	13
SDS-V3-plasma-24_Cluster_977_sequences=13	124	13
SDS-V3-plasma-24_Cluster_6374_sequences=13	124	13
SDS-V3-plasma-24_Cluster_1500_sequences=13	124	13
SDS-V3-plasma-24_Cluster_309_sequences=13	124	13
SDS-V3-plasma-24_Cluster_2617_sequences=13	124	13
SDS-V3-plasma-24_Cluster_1205_sequences=13	124	13
SDS-V3-plasma-24_Cluster_3325_sequences=13	124	13
SDS-V3-plasma-27_Cluster_1063_sequences=13	131	13
SDS-V3-plasma-27_Cluster_1660_sequences=13	131	13
SDS-V3-plasma-27_Cluster_2834_sequences=13	131	13
SDS-V3-plasma-27_Cluster_3166_sequences=13	131	13
SDS-V3-plasma-27_Cluster_3641_sequences=13	131	13
SDS-V3-plasma-27_Cluster_419_sequences=13	131	13
SDS-V3-plasma-27_Cluster_1400_sequences=13	131	13
SDS-V3-plasma-27_Cluster_3537_sequences=13	131	13
SDS-V3-plasma-27_Cluster_1733_sequences=13	131	13
SDS-V3-plasma-27_Cluster_1216_sequences=13	131	13
SDS-V3-plasma-27_Cluster_1634_sequences=13	131	13
SDS-V3-plasma-27_Cluster_1285_sequences=13	131	13
SDS-V3-plasma-27_Cluster_4443_sequences=13	131	13
SDS-V3-plasma-27_Cluster_3836_sequences=13	131	13
SDS-V3-plasma-27_Cluster_198_sequences=13	131	13
SDS-V3-plasma-27_Cluster_1246_sequences=13	131	13
SDS-V3-plasma-27_Cluster_3449_sequences=13	131	13
SDS-V3-plasma-45_Cluster_0_sequences=13	282	13
SDS-V3-plasma-45_Cluster_10331_sequences=13	282	13
SDS-V3-plasma-45_Cluster_8787_sequences=13	282	13
SDS-V3-plasma-45_Cluster_1260_sequences=13	282	13
SDS-V3-plasma-45_Cluster_4023_sequences=13	282	13
SDS-V3-plasma-45_Cluster_987_sequences=13	282	13
SDS-V3-plasma-45_Cluster_4695_sequences=13	282	13
SDS-V3-plasma-45_Cluster_1210_sequences=13	282	13
SDS-V3-plasma-45_Cluster_15410_sequences=13	282	13
SDS-V3-plasma-45_Cluster_1882_sequences=13	282	13
SDS-V3-plasma-45_Cluster_34887_sequences=13	282	13
SDS-V3-plasma-45_Cluster_19992_sequences=13	282	13
SDS-V3-plasma-45_Cluster_31296_sequences=13	282	13
SDS-V3-plasma-45_Cluster_664_sequences=13	282	13

SDS-V3-plasma-45_Cluster_13543_sequences=13	282	13
SDS-V3-plasma-45_Cluster_6433_sequences=13	282	13
SDS-V3-plasma-45_Cluster_10318_sequences=13	282	13
SDS-V3-plasma-45_Cluster_17030_sequences=13	282	13
SDS-V3-plasma-45_Cluster_18208_sequences=13	282	13
SDS-V3-plasma-45_Cluster_1949_sequences=13	282	13
SDS-V3-plasma-45_Cluster_24905_sequences=13	282	13
SDS-V3-plasma-45_Cluster_38555_sequences=13	282	13
SDS-V3-plasma-45_Cluster_7948_sequences=13	282	13
SDS-V3-plasma-45_Cluster_9958_sequences=13	282	13
SDS-V3-plasma-45_Cluster_1861_sequences=13	282	13
SDS-V3-plasma-45_Cluster_7065_sequences=13	282	13
SDS-V3-plasma-45_Cluster_10123_sequences=13	282	13
SDS-V3-plasma-45_Cluster_11570_sequences=13	282	13
SDS-V3-plasma-45_Cluster_121_sequences=13	282	13
SDS-V3-plasma-45_Cluster_12497_sequences=13	282	13
SDS-V3-plasma-45_Cluster_12878_sequences=13	282	13
SDS-V3-plasma-45_Cluster_13335_sequences=13	282	13
SDS-V3-plasma-45_Cluster_1860_sequences=13	282	13
SDS-V3-plasma-45_Cluster_19329_sequences=13	282	13
SDS-V3-plasma-45_Cluster_25731_sequences=13	282	13
SDS-V3-plasma-45_Cluster_28290_sequences=13	282	13
SDS-V3-plasma-45_Cluster_3105_sequences=13	282	13
SDS-V3-plasma-45_Cluster_40015_sequences=13	282	13
SDS-V3-plasma-45_Cluster_53483_sequences=13	282	13
SDS-V3-plasma-45_Cluster_5814_sequences=13	282	13
SDS-V3-plasma-45_Cluster_6151_sequences=13	282	13
SDS-V3-plasma-45_Cluster_8044_sequences=13	282	13
SDS-V3-plasma-45_Cluster_34593_sequences=13	282	13
SDS-V3-plasma-45_Cluster_40103_sequences=13	282	13
SDS-V3-plasma-45_Cluster_4287_sequences=13	282	13
SDS-V3-plasma-45_Cluster_2320_sequences=13	282	13
SDS-V3-plasma-45_Cluster_27690_sequences=13	282	13
SDS-V3-plasma-45_Cluster_1147_sequences=13	282	13
SDS-V3-plasma-45_Cluster_10674_sequences=13	282	13
SDS-V3-plasma-45_Cluster_38386_sequences=13	282	13
SDS-V3-plasma-45_Cluster_5497_sequences=13	282	13
SDS-V3-plasma-45_Cluster_20399_sequences=13	282	13
SDS-V3-plasma-45_Cluster_628_sequences=13	282	13
SDS-V3-plasma-45_Cluster_35382_sequences=13	282	13
SDS-V3-plasma-45_Cluster_4234_sequences=13	282	13
SDS-V3-plasma-45_Cluster_8173_sequences=13	282	13
SDS-V3-plasma-45_Cluster_9794_sequences=13	282	13
SDS-V3-plasma-45_Cluster_1634_sequences=13	282	13
SDS-V3-plasma-45_Cluster_6390_sequences=13	282	13

SDS-V3-plasma-45_Cluster_7554_sequences=13	282	13
SDS-V3-plasma-45_Cluster_14480_sequences=13	282	13
SDS-V3-plasma-45_Cluster_14921_sequences=13	282	13
SDS-V3-plasma-45_Cluster_12234_sequences=13	282	13
SDS-V3-plasma-45_Cluster_36099_sequences=13	282	13
SDS-V3-plasma-45_Cluster_5528_sequences=13	282	13
SDS-V3-plasma-45_Cluster_13264_sequences=13	282	13
SDS-V3-plasma-45_Cluster_6425_sequences=13	282	13
SDS-V3-plasma-45_Cluster_17363_sequences=13	282	13
SDS-V3-plasma-45_Cluster_23667_sequences=13	282	13
SDS-V3-plasma-45_Cluster_18747_sequences=13	282	13
SDS-V3-plasma-45_Cluster_7896_sequences=13	282	13
SDS-V3-plasma-45_Cluster_14558_sequences=13	282	13
SDS-V3-plasma-45_Cluster_2792_sequences=13	282	13
SDS-V3-plasma-45_Cluster_45521_sequences=13	282	13
SDS-V3-plasma-45_Cluster_3446_sequences=13	282	13
SDS-V3-plasma-45_Cluster_5648_sequences=13	282	13
SDS-V3-plasma-45_Cluster_18856_sequences=13	282	13
SDS-V3-plasma-45_Cluster_23514_sequences=13	282	13
SDS-V3-plasma-45_Cluster_19320_sequences=13	282	13
SDS-V3-plasma-45_Cluster_20442_sequences=13	282	13
SDS-V3-plasma-45_Cluster_202_sequences=13	282	13
SDS-V3-plasma-45_Cluster_6491_sequences=13	282	13
SDS-V3-plasma-45_Cluster_65693_sequences=13	282	13
SDS-V3-plasma-45_Cluster_10129_sequences=13	282	13
SDS-V3-plasma-45_Cluster_520_sequences=13	282	13
SDS-V3-plasma-45_Cluster_7699_sequences=13	282	13
SDS-V3-plasma-45_Cluster_4837_sequences=13	282	13
SDS-V3-plasma-45_Cluster_66615_sequences=13	282	13
SDS-V3-plasma-45_Cluster_2002_sequences=13	282	13
SDS-V3-plasma-45_Cluster_3099_sequences=13	282	13
SDS-V3-plasma-45_Cluster_4830_sequences=13	282	13
SDS-V3-plasma-45_Cluster_12826_sequences=13	282	13
SDS-V3-plasma-45_Cluster_12544_sequences=13	282	13
SDS-V3-plasma-45_Cluster_13247_sequences=13	282	13
SDS-V3-plasma-45_Cluster_17636_sequences=13	282	13
SDS-V3-plasma-45_Cluster_18721_sequences=13	282	13
SDS-V3-plasma-45_Cluster_3344_sequences=13	282	13
SDS-V3-plasma-45_Cluster_7627_sequences=13	282	13
SDS-V3-plasma-45_Cluster_6690_sequences=13	282	13
SDS-V3-plasma-45_Cluster_6757_sequences=13	282	13
SDS-V3-plasma-45_Cluster_24047_sequences=13	282	13
SDS-V3-plasma-45_Cluster_13107_sequences=13	282	13
SDS-V3-plasma-45_Cluster_27380_sequences=13	282	13
SDS-V3-plasma-45_Cluster_10507_sequences=13	282	13

SDS-V3-plasma-45_Cluster_11820_sequences=13	282	13
SDS-V3-plasma-45_Cluster_3959_sequences=13	282	13
SDS-V3-plasma-45_Cluster_9358_sequences=13	282	13
SDS-V3-plasma-45_Cluster_10690_sequences=13	282	13
SDS-V3-plasma-45_Cluster_24290_sequences=13	282	13
SDS-V3-plasma-45_Cluster_779_sequences=13	282	13
SDS-V3-plasma-45_Cluster_10387_sequences=13	282	13
SDS-V3-plasma-45_Cluster_6646_sequences=13	282	13
SDS-V3-plasma-45_Cluster_21833_sequences=13	282	13
SDS-V3-plasma-45_Cluster_33172_sequences=13	282	13
SDS-V3-plasma-45_Cluster_23119_sequences=13	282	13
SDS-V3-plasma-45_Cluster_4577_sequences=13	282	13
SDS-V3-plasma-45_Cluster_13566_sequences=13	282	13
SDS-V3-plasma-45_Cluster_16829_sequences=13	282	13
SDS-V3-plasma-45_Cluster_14490_sequences=13	282	13
SDS-V3-plasma-45_Cluster_6585_sequences=13	282	13
SDS-V3-plasma-45_Cluster_9326_sequences=13	282	13
SDS-V3-plasma-45_Cluster_11689_sequences=13	282	13
SDS-V3-plasma-45_Cluster_15543_sequences=13	282	13
SDS-V3-plasma-45_Cluster_8014_sequences=13	282	13
SDS-V3-plasma-45_Cluster_1746_sequences=13	282	13
SDS-V3-plasma-45_Cluster_39919_sequences=13	282	13
SDS-V3-plasma-45_Cluster_10529_sequences=13	282	13
SDS-V3-plasma-45_Cluster_42942_sequences=13	282	13
SDS-V3-plasma-45_Cluster_18151_sequences=13	282	13
SDS-V3-plasma-45_Cluster_15235_sequences=13	282	13
SDS-V3-plasma-45_Cluster_1792_sequences=13	282	13
SDS-V3-plasma-45_Cluster_24640_sequences=13	282	13
SDS-V3-plasma-45_Cluster_5096_sequences=13	282	13
SDS-V3-plasma-45_Cluster_6015_sequences=13	282	13
SDS-V3-plasma-45_Cluster_8356_sequences=13	282	13
SDS-V3-plasma-45_Cluster_2567_sequences=13	282	13
SDS-V3-plasma-45_Cluster_40288_sequences=13	282	13
SDS-V3-plasma-45_Cluster_8822_sequences=13	282	13
SDS-V3-plasma-45_Cluster_25595_sequences=13	282	13
SDS-V3-plasma-45_Cluster_19534_sequences=13	282	13
SDS-V3-plasma-45_Cluster_17411_sequences=13	282	13
SDS-V3-plasma-45_Cluster_5382_sequences=13	282	13
SDS-V3-plasma-45_Cluster_51425_sequences=13	282	13
SDS-V3-plasma-45_Cluster_18404_sequences=13	282	13
SDS-V3-plasma-45_Cluster_25459_sequences=13	282	13
SDS-V3-plasma-45_Cluster_9836_sequences=13	282	13
SDS-V3-plasma-45_Cluster_21064_sequences=13	282	13
SDS-V3-plasma-45_Cluster_1149_sequences=13	282	13
SDS-V3-plasma-45_Cluster_3057_sequences=13	282	13

SDS-V3-plasma-45_Cluster_8309_sequences=13	282	13
SDS-V3-plasma-45_Cluster_8724_sequences=13	282	13
SDS-V3-plasma-45_Cluster_11097_sequences=13	282	13
SDS-V3-plasma-45_Cluster_10540_sequences=13	282	13
SDS-V3-plasma-45_Cluster_31931_sequences=13	282	13
SDS-V3-plasma-45_Cluster_34347_sequences=13	282	13
SDS-V3-plasma-45_Cluster_65732_sequences=13	282	13
SDS-V3-plasma-46_Cluster_2588_sequences=13	286	13
SDS-V3-plasma-46_Cluster_6560_sequences=13	286	13
SDS-V3-plasma-46_Cluster_14_sequences=13	286	13
SDS-V3-plasma-46_Cluster_6050_sequences=13	286	13
SDS-V3-plasma-46_Cluster_5715_sequences=13	286	13
SDS-V3-plasma-46_Cluster_19865_sequences=13	286	13
SDS-V3-plasma-46_Cluster_988_sequences=13	286	13
SDS-V3-plasma-46_Cluster_4568_sequences=13	286	13
SDS-V3-plasma-46_Cluster_5153_sequences=13	286	13
SDS-V3-plasma-46_Cluster_8072_sequences=13	286	13
SDS-V3-plasma-46_Cluster_3536_sequences=13	286	13
SDS-V3-plasma-46_Cluster_5285_sequences=13	286	13
SDS-V3-plasma-46_Cluster_7141_sequences=13	286	13
SDS-V3-plasma-46_Cluster_1931_sequences=13	286	13
SDS-V3-plasma-46_Cluster_2578_sequences=13	286	13
SDS-V3-plasma-46_Cluster_2614_sequences=13	286	13
SDS-V3-plasma-46_Cluster_2207_sequences=13	286	13
SDS-V3-plasma-46_Cluster_2704_sequences=13	286	13
SDS-V3-plasma-46_Cluster_7709_sequences=13	286	13
SDS-V3-plasma-46_Cluster_5740_sequences=13	286	13
SDS-V3-plasma-46_Cluster_4968_sequences=13	286	13
SDS-V3-plasma-46_Cluster_4316_sequences=13	286	13
SDS-V3-plasma-46_Cluster_3116_sequences=13	286	13
SDS-V3-plasma-46_Cluster_2555_sequences=13	286	13
SDS-V3-plasma-46_Cluster_1864_sequences=13	286	13
SDS-V3-plasma-46_Cluster_10724_sequences=13	286	13
SDS-V3-plasma-46_Cluster_1853_sequences=13	286	13
SDS-V3-plasma-46_Cluster_2531_sequences=13	286	13
SDS-V3-plasma-46_Cluster_2961_sequences=13	286	13
SDS-V3-plasma-46_Cluster_4604_sequences=13	286	13
SDS-V3-plasma-46_Cluster_5698_sequences=13	286	13
SDS-V3-plasma-46_Cluster_5989_sequences=13	286	13
SDS-V3-plasma-46_Cluster_6369_sequences=13	286	13
SDS-V3-plasma-46_Cluster_5033_sequences=13	286	13
SDS-V3-plasma-46_Cluster_6297_sequences=13	286	13
SDS-V3-plasma-46_Cluster_8054_sequences=13	286	13
SDS-V3-plasma-46_Cluster_2719_sequences=13	286	13
SDS-V3-plasma-46_Cluster_4121_sequences=13	286	13

SDS-V3-plasma-46_Cluster_7048_sequences=13	286	13
SDS-V3-plasma-46_Cluster_3197_sequences=13	286	13
SDS-V3-plasma-46_Cluster_3893_sequences=13	286	13
SDS-V3-plasma-46_Cluster_509_sequences=13	286	13
SDS-V3-plasma-46_Cluster_6173_sequences=13	286	13
SDS-V3-plasma-46_Cluster_10526_sequences=13	286	13
SDS-V3-plasma-46_Cluster_2005_sequences=13	286	13
SDS-V3-plasma-46_Cluster_7767_sequences=13	286	13
SDS-V3-plasma-46_Cluster_3778_sequences=13	286	13
SDS-V3-plasma-46_Cluster_1999_sequences=13	286	13
SDS-V3-plasma-46_Cluster_5224_sequences=13	286	13
SDS-V3-plasma-46_Cluster_6024_sequences=13	286	13
SDS-V3-plasma-46_Cluster_1006_sequences=13	286	13
SDS-V3-plasma-46_Cluster_279_sequences=13	286	13
SDS-V3-plasma-46_Cluster_3410_sequences=13	286	13
SDS-V3-plasma-46_Cluster_12220_sequences=13	286	13
SDS-V3-plasma-46_Cluster_13611_sequences=13	286	13
SDS-V3-plasma-46_Cluster_3352_sequences=13	286	13
SDS-V3-plasma-46_Cluster_4960_sequences=13	286	13
SDS-V3-plasma-67_Cluster_10004_sequences=13	504	13
SDS-V3-plasma-67_Cluster_4646_sequences=13	504	13
SDS-V3-plasma-67_Cluster_3216_sequences=13	504	13
SDS-V3-plasma-67_Cluster_18317_sequences=13	504	13
SDS-V3-plasma-67_Cluster_14055_sequences=13	504	13
SDS-V3-plasma-67_Cluster_2638_sequences=13	504	13
SDS-V3-plasma-67_Cluster_21662_sequences=13	504	13
SDS-V3-plasma-67_Cluster_13409_sequences=13	504	13
SDS-V3-plasma-67_Cluster_2111_sequences=13	504	13
SDS-V3-plasma-67_Cluster_5911_sequences=13	504	13
SDS-V3-plasma-67_Cluster_7752_sequences=13	504	13
SDS-V3-plasma-67_Cluster_35477_sequences=13	504	13
SDS-V3-plasma-67_Cluster_13207_sequences=13	504	13
SDS-V3-plasma-67_Cluster_4911_sequences=13	504	13
SDS-V3-plasma-67_Cluster_3884_sequences=13	504	13
SDS-V3-plasma-67_Cluster_14946_sequences=13	504	13
SDS-V3-plasma-67_Cluster_40363_sequences=13	504	13
SDS-V3-plasma-67_Cluster_21988_sequences=13	504	13
SDS-V3-plasma-67_Cluster_6296_sequences=13	504	13
SDS-V3-plasma-67_Cluster_49136_sequences=13	504	13
SDS-V3-plasma-67_Cluster_6579_sequences=13	504	13
SDS-V3-plasma-67_Cluster_12215_sequences=13	504	13
SDS-V3-plasma-67_Cluster_23058_sequences=13	504	13
SDS-V3-plasma-67_Cluster_5500_sequences=13	504	13
SDS-V3-plasma-67_Cluster_16238_sequences=13	504	13
SDS-V3-plasma-67_Cluster_4749_sequences=13	504	13

SDS-V3-plasma-67_Cluster_24452_sequences=13	504	13
SDS-V3-plasma-67_Cluster_35626_sequences=13	504	13
SDS-V3-plasma-67_Cluster_12230_sequences=13	504	13
SDS-V3-plasma-67_Cluster_14766_sequences=13	504	13
SDS-V3-plasma-67_Cluster_49933_sequences=13	504	13
SDS-V3-plasma-67_Cluster_7990_sequences=13	504	13
SDS-V3-plasma-67_Cluster_8277_sequences=13	504	13
SDS-V3-plasma-67_Cluster_2943_sequences=13	504	13
SDS-V3-plasma-67_Cluster_32572_sequences=13	504	13
SDS-V3-plasma-67_Cluster_4125_sequences=13	504	13
SDS-V3-plasma-67_Cluster_10199_sequences=13	504	13
SDS-V3-plasma-67_Cluster_8952_sequences=13	504	13
SDS-V3-plasma-67_Cluster_17774_sequences=13	504	13
SDS-V3-plasma-67_Cluster_3813_sequences=13	504	13
SDS-V3-plasma-67_Cluster_2082_sequences=13	504	13
SDS-V3-plasma-67_Cluster_11297_sequences=13	504	13
SDS-V3-plasma-67_Cluster_13573_sequences=13	504	13
SDS-V3-plasma-67_Cluster_1707_sequences=13	504	13
SDS-V3-plasma-67_Cluster_604_sequences=13	504	13
SDS-V3-plasma-67_Cluster_36282_sequences=13	504	13
SDS-V3-plasma-67_Cluster_75581_sequences=13	504	13
SDS-V3-plasma-67_Cluster_28492_sequences=13	504	13
SDS-V3-plasma-67_Cluster_8200_sequences=13	504	13
SDS-V3-plasma-67_Cluster_13820_sequences=13	504	13
SDS-V3-plasma-67_Cluster_5216_sequences=13	504	13
SDS-V3-plasma-67_Cluster_10544_sequences=13	504	13
SDS-V3-plasma-67_Cluster_1987_sequences=13	504	13
SDS-V3-plasma-67_Cluster_5989_sequences=13	504	13
SDS-V3-plasma-67_Cluster_13398_sequences=13	504	13
SDS-V3-plasma-67_Cluster_8289_sequences=13	504	13
SDS-V3-plasma-67_Cluster_17808_sequences=13	504	13
SDS-V3-plasma-67_Cluster_2390_sequences=13	504	13
SDS-V3-plasma-67_Cluster_2797_sequences=13	504	13
SDS-V3-plasma-67_Cluster_40066_sequences=13	504	13
SDS-V3-plasma-67_Cluster_4294_sequences=13	504	13
SDS-V3-plasma-67_Cluster_5171_sequences=13	504	13
SDS-V3-plasma-67_Cluster_7446_sequences=13	504	13
SDS-V3-plasma-67_Cluster_6661_sequences=13	504	13
SDS-V3-plasma-67_Cluster_43615_sequences=13	504	13
SDS-V3-plasma-67_Cluster_31818_sequences=13	504	13
SDS-V3-plasma-67_Cluster_16965_sequences=13	504	13
SDS-V3-plasma-67_Cluster_13404_sequences=13	504	13
SDS-V3-plasma-67_Cluster_19918_sequences=13	504	13
SDS-V3-plasma-67_Cluster_22199_sequences=13	504	13
SDS-V3-plasma-67_Cluster_4848_sequences=13	504	13

SDS-V3-plasma-67_Cluster_13937_sequences=13	504	13
SDS-V3-plasma-67_Cluster_5284_sequences=13	504	13
SDS-V3-plasma-67_Cluster_21546_sequences=13	504	13
SDS-V3-plasma-67_Cluster_10846_sequences=13	504	13
SDS-V3-plasma-67_Cluster_8508_sequences=13	504	13
SDS-V3-plasma-67_Cluster_4185_sequences=13	504	13
SDS-V3-plasma-67_Cluster_22363_sequences=13	504	13
SDS-V3-plasma-67_Cluster_31494_sequences=13	504	13
SDS-V3-plasma-67_Cluster_14888_sequences=13	504	13
SDS-V3-plasma-67_Cluster_13460_sequences=13	504	13
SDS-V3-plasma-67_Cluster_1112_sequences=13	504	13
SDS-V3-plasma-67_Cluster_781_sequences=13	504	13
SDS-V3-plasma-67_Cluster_27474_sequences=13	504	13
SDS-V3-plasma-67_Cluster_9579_sequences=13	504	13
SDS-V3-plasma-67_Cluster_10407_sequences=13	504	13
SDS-V3-plasma-67_Cluster_7331_sequences=13	504	13
SDS-V3-plasma-67_Cluster_7861_sequences=13	504	13
SDS-V3-plasma-67_Cluster_18441_sequences=13	504	13
SDS-V3-plasma-67_Cluster_22209_sequences=13	504	13
SDS-V3-plasma-67_Cluster_8846_sequences=13	504	13
SDS-V3-plasma-67_Cluster_4139_sequences=13	504	13
SDS-V3-plasma-67_Cluster_32531_sequences=13	504	13
SDS-V3-plasma-67_Cluster_4331_sequences=13	504	13
SDS-V3-plasma-67_Cluster_11670_sequences=13	504	13
SDS-V3-plasma-67_Cluster_12251_sequences=13	504	13
SDS-V3-plasma-67_Cluster_2587_sequences=13	504	13
SDS-V3-plasma-67_Cluster_5025_sequences=13	504	13
SDS-V3-plasma-67_Cluster_5155_sequences=13	504	13
SDS-V3-plasma-67_Cluster_6406_sequences=13	504	13
SDS-V3-plasma-67_Cluster_828_sequences=13	504	13
SDS-V3-plasma-67_Cluster_8651_sequences=13	504	13
SDS-V3-plasma-67_Cluster_9574_sequences=13	504	13
SDS-V3-plasma-67_Cluster_13086_sequences=13	504	13
SDS-V3-plasma-67_Cluster_58344_sequences=13	504	13
SDS-V3-plasma-67_Cluster_9101_sequences=13	504	13
SDS-V3-plasma-67_Cluster_7542_sequences=13	504	13
SDS-V3-plasma-67_Cluster_17497_sequences=13	504	13
SDS-V3-plasma-67_Cluster_2379_sequences=13	504	13
SDS-V3-plasma-67_Cluster_21551_sequences=13	504	13
SDS-V3-plasma-67_Cluster_2074_sequences=13	504	13
SDS-V3-plasma-67_Cluster_25943_sequences=13	504	13
SDS-V3-plasma-67_Cluster_11416_sequences=13	504	13
SDS-V3-plasma-67_Cluster_20075_sequences=13	504	13
SDS-V3-plasma-67_Cluster_10342_sequences=13	504	13
SDS-V3-plasma-67_Cluster_24968_sequences=13	504	13

SDS-V3-plasma-67_Cluster_23204_sequences=13	504	13
SDS-V3-plasma-67_Cluster_27788_sequences=13	504	13
SDS-V3-plasma-67_Cluster_3038_sequences=13	504	13
SDS-V3-plasma-67_Cluster_39317_sequences=13	504	13
SDS-V3-plasma-67_Cluster_9737_sequences=13	504	13
SDS-V3-plasma-67_Cluster_15336_sequences=13	504	13
SDS-V3-plasma-67_Cluster_20819_sequences=13	504	13
SDS-V3-plasma-67_Cluster_3831_sequences=13	504	13
SDS-V3-plasma-67_Cluster_6189_sequences=13	504	13
SDS-V3-plasma-67_Cluster_3790_sequences=13	504	13
SDS-V3-plasma-67_Cluster_21684_sequences=13	504	13
SDS-V3-plasma-67_Cluster_21995_sequences=13	504	13
SDS-V3-plasma-67_Cluster_2948_sequences=13	504	13
SDS-V3-plasma-67_Cluster_293_sequences=13	504	13
SDS-V3-plasma-0_Cluster_1683_sequences=12	0	12
SDS-V3-plasma-0_Cluster_2075_sequences=12	0	12
SDS-V3-plasma-0_Cluster_5004_sequences=12	0	12
SDS-V3-plasma-0_Cluster_7603_sequences=12	0	12
SDS-V3-plasma-0_Cluster_2465_sequences=12	0	12
SDS-V3-plasma-0_Cluster_2082_sequences=12	0	12
SDS-V3-plasma-0_Cluster_2309_sequences=12	0	12
SDS-V3-plasma-0_Cluster_4837_sequences=12	0	12
SDS-V3-plasma-0_Cluster_4982_sequences=12	0	12
SDS-V3-plasma-0_Cluster_5008_sequences=12	0	12
SDS-V3-plasma-0_Cluster_5051_sequences=12	0	12
SDS-V3-plasma-0_Cluster_5368_sequences=12	0	12
SDS-V3-plasma-0_Cluster_5711_sequences=12	0	12
SDS-V3-plasma-0_Cluster_6373_sequences=12	0	12
SDS-V3-plasma-0_Cluster_6532_sequences=12	0	12
SDS-V3-plasma-0_Cluster_7429_sequences=12	0	12
SDS-V3-plasma-0_Cluster_9587_sequences=12	0	12
SDS-V3-plasma-0_Cluster_1759_sequences=12	0	12
SDS-V3-plasma-0_Cluster_2022_sequences=12	0	12
SDS-V3-plasma-0_Cluster_5123_sequences=12	0	12
SDS-V3-plasma-0_Cluster_8519_sequences=12	0	12
SDS-V3-plasma-0_Cluster_1526_sequences=12	0	12
SDS-V3-plasma-0_Cluster_1620_sequences=12	0	12
SDS-V3-plasma-0_Cluster_6554_sequences=12	0	12
SDS-V3-plasma-0_Cluster_4202_sequences=12	0	12
SDS-V3-plasma-0_Cluster_6570_sequences=12	0	12
SDS-V3-plasma-0_Cluster_11522_sequences=12	0	12
SDS-V3-plasma-0_Cluster_4831_sequences=12	0	12
SDS-V3-plasma-0_Cluster_1387_sequences=12	0	12
SDS-V3-plasma-0_Cluster_7016_sequences=12	0	12
SDS-V3-plasma-0_Cluster_3466_sequences=12	0	12

SDS-V3-plasma-0_Cluster_2396_sequences=12	0	12
SDS-V3-plasma-0_Cluster_4154_sequences=12	0	12
SDS-V3-plasma-0_Cluster_5037_sequences=12	0	12
SDS-V3-plasma-0_Cluster_3078_sequences=12	0	12
SDS-V3-plasma-0_Cluster_3555_sequences=12	0	12
SDS-V3-plasma-0_Cluster_4066_sequences=12	0	12
SDS-V3-plasma-0_Cluster_3104_sequences=12	0	12
SDS-V3-plasma-0_Cluster_4251_sequences=12	0	12
SDS-V3-plasma-0_Cluster_2441_sequences=12	0	12
SDS-V3-plasma-0_Cluster_25235_sequences=12	0	12
SDS-V3-plasma-0_Cluster_4497_sequences=12	0	12
SDS-V3-plasma-0_Cluster_2323_sequences=12	0	12
SDS-V3-plasma-0_Cluster_1401_sequences=12	0	12
SDS-V3-plasma-0_Cluster_1721_sequences=12	0	12
SDS-V3-plasma-0_Cluster_1384_sequences=12	0	12
SDS-V3-plasma-0_Cluster_142_sequences=12	0	12
SDS-V3-plasma-0_Cluster_7201_sequences=12	0	12
SDS-V3-plasma-0_Cluster_8278_sequences=12	0	12
SDS-V3-plasma-0_Cluster_6674_sequences=12	0	12
SDS-V3-plasma-0_Cluster_13798_sequences=12	0	12
SDS-V3-plasma-0_Cluster_8460_sequences=12	0	12
SDS-V3-plasma-0_Cluster_3412_sequences=12	0	12
SDS-V3-plasma-0_Cluster_2382_sequences=12	0	12
SDS-V3-plasma-0_Cluster_7321_sequences=12	0	12
SDS-V3-plasma-0_Cluster_4258_sequences=12	0	12
SDS-V3-plasma-0_Cluster_3672_sequences=12	0	12
SDS-V3-plasma-0_Cluster_1054_sequences=12	0	12
SDS-V3-plasma-0_Cluster_3329_sequences=12	0	12
SDS-V3-plasma-0_Cluster_1372_sequences=12	0	12
SDS-V3-plasma-0_Cluster_919_sequences=12	0	12
SDS-V3-plasma-0_Cluster_4980_sequences=12	0	12
SDS-V3-plasma-0_Cluster_1712_sequences=12	0	12
SDS-V3-plasma-0_Cluster_78_sequences=12	0	12
SDS-V3-plasma-0_Cluster_1791_sequences=12	0	12
SDS-V3-plasma-0_Cluster_2860_sequences=12	0	12
SDS-V3-plasma-0_Cluster_3567_sequences=12	0	12
SDS-V3-plasma-0_Cluster_2274_sequences=12	0	12
SDS-V3-plasma-0_Cluster_347_sequences=12	0	12
SDS-V3-plasma-5_Cluster_0_sequences=12	9	12
SDS-V3-plasma-5_Cluster_283_sequences=12	9	12
SDS-V3-plasma-5_Cluster_364_sequences=12	9	12
SDS-V3-plasma-5_Cluster_434_sequences=12	9	12
SDS-V3-plasma-5_Cluster_637_sequences=12	9	12
SDS-V3-plasma-5_Cluster_164_sequences=12	9	12
SDS-V3-plasma-5_Cluster_594_sequences=12	9	12

SDS-V3-plasma-5_Cluster_407_sequences=12	9	12
SDS-V3-plasma-5_Cluster_1148_sequences=12	9	12
SDS-V3-plasma-5_Cluster_1245_sequences=12	9	12
SDS-V3-plasma-5_Cluster_491_sequences=12	9	12
SDS-V3-plasma-5_Cluster_483_sequences=12	9	12
SDS-V3-plasma-5_Cluster_484_sequences=12	9	12
SDS-V3-plasma-5_Cluster_739_sequences=12	9	12
SDS-V3-plasma-5_Cluster_699_sequences=12	9	12
SDS-V3-plasma-5_Cluster_70_sequences=12	9	12
SDS-V3-plasma-5_Cluster_75_sequences=12	9	12
SDS-V3-plasma-7_Cluster_1655_sequences=12	14	12
SDS-V3-plasma-7_Cluster_4457_sequences=12	14	12
SDS-V3-plasma-7_Cluster_2074_sequences=12	14	12
SDS-V3-plasma-7_Cluster_1487_sequences=12	14	12
SDS-V3-plasma-7_Cluster_1022_sequences=12	14	12
SDS-V3-plasma-7_Cluster_1666_sequences=12	14	12
SDS-V3-plasma-7_Cluster_521_sequences=12	14	12
SDS-V3-plasma-7_Cluster_880_sequences=12	14	12
SDS-V3-plasma-7_Cluster_986_sequences=12	14	12
SDS-V3-plasma-7_Cluster_1564_sequences=12	14	12
SDS-V3-plasma-7_Cluster_222_sequences=12	14	12
SDS-V3-plasma-7_Cluster_49_sequences=12	14	12
SDS-V3-plasma-7_Cluster_587_sequences=12	14	12
SDS-V3-plasma-7_Cluster_440_sequences=12	14	12
SDS-V3-plasma-7_Cluster_676_sequences=12	14	12
SDS-V3-plasma-7_Cluster_3427_sequences=12	14	12
SDS-V3-plasma-7_Cluster_959_sequences=12	14	12
SDS-V3-plasma-7_Cluster_1971_sequences=12	14	12
SDS-V3-plasma-7_Cluster_1526_sequences=12	14	12
SDS-V3-plasma-7_Cluster_2189_sequences=12	14	12
SDS-V3-plasma-7_Cluster_2694_sequences=12	14	12
SDS-V3-plasma-7_Cluster_1438_sequences=12	14	12
SDS-V3-plasma-7_Cluster_2098_sequences=12	14	12
SDS-V3-plasma-7_Cluster_4949_sequences=12	14	12
SDS-V3-plasma-7_Cluster_1122_sequences=12	14	12
SDS-V3-plasma-7_Cluster_631_sequences=12	14	12
SDS-V3-plasma-7_Cluster_1571_sequences=12	14	12
SDS-V3-plasma-7_Cluster_2215_sequences=12	14	12
SDS-V3-plasma-7_Cluster_1750_sequences=12	14	12
SDS-V3-plasma-7_Cluster_4905_sequences=12	14	12
SDS-V3-plasma-7_Cluster_2204_sequences=12	14	12
SDS-V3-plasma-8_Cluster_101_sequences=12	16	12
SDS-V3-plasma-8_Cluster_26_sequences=12	16	12
SDS-V3-plasma-8_Cluster_111_sequences=12	16	12
SDS-V3-plasma-8_Cluster_782_sequences=12	16	12

SDS-V3-plasma-8_Cluster_530_sequences=12	16	12
SDS-V3-plasma-8_Cluster_93_sequences=12	16	12
SDS-V3-plasma-8_Cluster_4903_sequences=12	16	12
SDS-V3-plasma-8_Cluster_2549_sequences=12	16	12
SDS-V3-plasma-8_Cluster_9276_sequences=12	16	12
SDS-V3-plasma-8_Cluster_2486_sequences=12	16	12
SDS-V3-plasma-8_Cluster_2689_sequences=12	16	12
SDS-V3-plasma-8_Cluster_2984_sequences=12	16	12
SDS-V3-plasma-8_Cluster_3673_sequences=12	16	12
SDS-V3-plasma-8_Cluster_3619_sequences=12	16	12
SDS-V3-plasma-8_Cluster_3243_sequences=12	16	12
SDS-V3-plasma-8_Cluster_4514_sequences=12	16	12
SDS-V3-plasma-8_Cluster_3853_sequences=12	16	12
SDS-V3-plasma-8_Cluster_3858_sequences=12	16	12
SDS-V3-plasma-8_Cluster_2774_sequences=12	16	12
SDS-V3-plasma-8_Cluster_4512_sequences=12	16	12
SDS-V3-plasma-8_Cluster_5504_sequences=12	16	12
SDS-V3-plasma-8_Cluster_3008_sequences=12	16	12
SDS-V3-plasma-8_Cluster_2418_sequences=12	16	12
SDS-V3-plasma-8_Cluster_3018_sequences=12	16	12
SDS-V3-plasma-8_Cluster_6020_sequences=12	16	12
SDS-V3-plasma-8_Cluster_4434_sequences=12	16	12
SDS-V3-plasma-8_Cluster_3184_sequences=12	16	12
SDS-V3-plasma-8_Cluster_329_sequences=12	16	12
SDS-V3-plasma-8_Cluster_7324_sequences=12	16	12
SDS-V3-plasma-24_Cluster_1628_sequences=12	124	12
SDS-V3-plasma-24_Cluster_1668_sequences=12	124	12
SDS-V3-plasma-24_Cluster_279_sequences=12	124	12
SDS-V3-plasma-24_Cluster_2808_sequences=12	124	12
SDS-V3-plasma-24_Cluster_4048_sequences=12	124	12
SDS-V3-plasma-24_Cluster_884_sequences=12	124	12
SDS-V3-plasma-24_Cluster_945_sequences=12	124	12
SDS-V3-plasma-24_Cluster_4982_sequences=12	124	12
SDS-V3-plasma-24_Cluster_765_sequences=12	124	12
SDS-V3-plasma-24_Cluster_685_sequences=12	124	12
SDS-V3-plasma-24_Cluster_2028_sequences=12	124	12
SDS-V3-plasma-24_Cluster_2043_sequences=12	124	12
SDS-V3-plasma-24_Cluster_327_sequences=12	124	12
SDS-V3-plasma-24_Cluster_4209_sequences=12	124	12
SDS-V3-plasma-24_Cluster_1785_sequences=12	124	12
SDS-V3-plasma-24_Cluster_3358_sequences=12	124	12
SDS-V3-plasma-24_Cluster_1136_sequences=12	124	12
SDS-V3-plasma-24_Cluster_2363_sequences=12	124	12
SDS-V3-plasma-24_Cluster_2992_sequences=12	124	12
SDS-V3-plasma-24_Cluster_397_sequences=12	124	12

SDS-V3-plasma-24_Cluster_2381_sequences=12	124	12
SDS-V3-plasma-24_Cluster_1042_sequences=12	124	12
SDS-V3-plasma-24_Cluster_2974_sequences=12	124	12
SDS-V3-plasma-27_Cluster_2700_sequences=12	131	12
SDS-V3-plasma-27_Cluster_3177_sequences=12	131	12
SDS-V3-plasma-27_Cluster_4234_sequences=12	131	12
SDS-V3-plasma-27_Cluster_488_sequences=12	131	12
SDS-V3-plasma-27_Cluster_653_sequences=12	131	12
SDS-V3-plasma-27_Cluster_2145_sequences=12	131	12
SDS-V3-plasma-27_Cluster_1534_sequences=12	131	12
SDS-V3-plasma-27_Cluster_4194_sequences=12	131	12
SDS-V3-plasma-27_Cluster_220_sequences=12	131	12
SDS-V3-plasma-27_Cluster_3735_sequences=12	131	12
SDS-V3-plasma-27_Cluster_722_sequences=12	131	12
SDS-V3-plasma-27_Cluster_164_sequences=12	131	12
SDS-V3-plasma-27_Cluster_190_sequences=12	131	12
SDS-V3-plasma-27_Cluster_2809_sequences=12	131	12
SDS-V3-plasma-27_Cluster_2160_sequences=12	131	12
SDS-V3-plasma-27_Cluster_2143_sequences=12	131	12
SDS-V3-plasma-27_Cluster_1230_sequences=12	131	12
SDS-V3-plasma-27_Cluster_1502_sequences=12	131	12
SDS-V3-plasma-27_Cluster_909_sequences=12	131	12
SDS-V3-plasma-27_Cluster_4857_sequences=12	131	12
SDS-V3-plasma-45_Cluster_10403_sequences=12	282	12
SDS-V3-plasma-45_Cluster_10430_sequences=12	282	12
SDS-V3-plasma-45_Cluster_11083_sequences=12	282	12
SDS-V3-plasma-45_Cluster_12613_sequences=12	282	12
SDS-V3-plasma-45_Cluster_11507_sequences=12	282	12
SDS-V3-plasma-45_Cluster_13121_sequences=12	282	12
SDS-V3-plasma-45_Cluster_13405_sequences=12	282	12
SDS-V3-plasma-45_Cluster_37249_sequences=12	282	12
SDS-V3-plasma-45_Cluster_45873_sequences=12	282	12
SDS-V3-plasma-45_Cluster_577_sequences=12	282	12
SDS-V3-plasma-45_Cluster_7892_sequences=12	282	12
SDS-V3-plasma-45_Cluster_317_sequences=12	282	12
SDS-V3-plasma-45_Cluster_17757_sequences=12	282	12
SDS-V3-plasma-45_Cluster_30885_sequences=12	282	12
SDS-V3-plasma-45_Cluster_17474_sequences=12	282	12
SDS-V3-plasma-45_Cluster_32514_sequences=12	282	12
SDS-V3-plasma-45_Cluster_9852_sequences=12	282	12
SDS-V3-plasma-45_Cluster_21974_sequences=12	282	12
SDS-V3-plasma-45_Cluster_2104_sequences=12	282	12
SDS-V3-plasma-45_Cluster_51978_sequences=12	282	12
SDS-V3-plasma-45_Cluster_7877_sequences=12	282	12
SDS-V3-plasma-45_Cluster_9626_sequences=12	282	12

SDS-V3-plasma-45_Cluster_11973_sequences=12	282	12
SDS-V3-plasma-45_Cluster_13516_sequences=12	282	12
SDS-V3-plasma-45_Cluster_18849_sequences=12	282	12
SDS-V3-plasma-45_Cluster_7765_sequences=12	282	12
SDS-V3-plasma-45_Cluster_5348_sequences=12	282	12
SDS-V3-plasma-45_Cluster_21819_sequences=12	282	12
SDS-V3-plasma-45_Cluster_21988_sequences=12	282	12
SDS-V3-plasma-45_Cluster_19741_sequences=12	282	12
SDS-V3-plasma-45_Cluster_6119_sequences=12	282	12
SDS-V3-plasma-45_Cluster_27433_sequences=12	282	12
SDS-V3-plasma-45_Cluster_3012_sequences=12	282	12
SDS-V3-plasma-45_Cluster_11992_sequences=12	282	12
SDS-V3-plasma-45_Cluster_5744_sequences=12	282	12
SDS-V3-plasma-45_Cluster_19010_sequences=12	282	12
SDS-V3-plasma-45_Cluster_22537_sequences=12	282	12
SDS-V3-plasma-45_Cluster_32119_sequences=12	282	12
SDS-V3-plasma-45_Cluster_3931_sequences=12	282	12
SDS-V3-plasma-45_Cluster_4993_sequences=12	282	12
SDS-V3-plasma-45_Cluster_50125_sequences=12	282	12
SDS-V3-plasma-45_Cluster_8129_sequences=12	282	12
SDS-V3-plasma-45_Cluster_5407_sequences=12	282	12
SDS-V3-plasma-45_Cluster_2875_sequences=12	282	12
SDS-V3-plasma-45_Cluster_10347_sequences=12	282	12
SDS-V3-plasma-45_Cluster_15122_sequences=12	282	12
SDS-V3-plasma-45_Cluster_15138_sequences=12	282	12
SDS-V3-plasma-45_Cluster_16235_sequences=12	282	12
SDS-V3-plasma-45_Cluster_16588_sequences=12	282	12
SDS-V3-plasma-45_Cluster_16595_sequences=12	282	12
SDS-V3-plasma-45_Cluster_17229_sequences=12	282	12
SDS-V3-plasma-45_Cluster_17298_sequences=12	282	12
SDS-V3-plasma-45_Cluster_17786_sequences=12	282	12
SDS-V3-plasma-45_Cluster_1799_sequences=12	282	12
SDS-V3-plasma-45_Cluster_18982_sequences=12	282	12
SDS-V3-plasma-45_Cluster_27537_sequences=12	282	12
SDS-V3-plasma-45_Cluster_3207_sequences=12	282	12
SDS-V3-plasma-45_Cluster_34447_sequences=12	282	12
SDS-V3-plasma-45_Cluster_38434_sequences=12	282	12
SDS-V3-plasma-45_Cluster_4927_sequences=12	282	12
SDS-V3-plasma-45_Cluster_54927_sequences=12	282	12
SDS-V3-plasma-45_Cluster_607_sequences=12	282	12
SDS-V3-plasma-45_Cluster_6442_sequences=12	282	12
SDS-V3-plasma-45_Cluster_7317_sequences=12	282	12
SDS-V3-plasma-45_Cluster_552_sequences=12	282	12
SDS-V3-plasma-45_Cluster_6718_sequences=12	282	12
SDS-V3-plasma-45_Cluster_12696_sequences=12	282	12

SDS-V3-plasma-45_Cluster_10098_sequences=12	282	12
SDS-V3-plasma-45_Cluster_5145_sequences=12	282	12
SDS-V3-plasma-45_Cluster_7730_sequences=12	282	12
SDS-V3-plasma-45_Cluster_9607_sequences=12	282	12
SDS-V3-plasma-45_Cluster_12851_sequences=12	282	12
SDS-V3-plasma-45_Cluster_8074_sequences=12	282	12
SDS-V3-plasma-45_Cluster_15585_sequences=12	282	12
SDS-V3-plasma-45_Cluster_4565_sequences=12	282	12
SDS-V3-plasma-45_Cluster_23438_sequences=12	282	12
SDS-V3-plasma-45_Cluster_23045_sequences=12	282	12
SDS-V3-plasma-45_Cluster_11498_sequences=12	282	12
SDS-V3-plasma-45_Cluster_13605_sequences=12	282	12
SDS-V3-plasma-45_Cluster_11768_sequences=12	282	12
SDS-V3-plasma-45_Cluster_12755_sequences=12	282	12
SDS-V3-plasma-45_Cluster_14663_sequences=12	282	12
SDS-V3-plasma-45_Cluster_16062_sequences=12	282	12
SDS-V3-plasma-45_Cluster_9506_sequences=12	282	12
SDS-V3-plasma-45_Cluster_2139_sequences=12	282	12
SDS-V3-plasma-45_Cluster_49656_sequences=12	282	12
SDS-V3-plasma-45_Cluster_673_sequences=12	282	12
SDS-V3-plasma-45_Cluster_49970_sequences=12	282	12
SDS-V3-plasma-45_Cluster_10117_sequences=12	282	12
SDS-V3-plasma-45_Cluster_10323_sequences=12	282	12
SDS-V3-plasma-45_Cluster_18044_sequences=12	282	12
SDS-V3-plasma-45_Cluster_10713_sequences=12	282	12
SDS-V3-plasma-45_Cluster_2035_sequences=12	282	12
SDS-V3-plasma-45_Cluster_20161_sequences=12	282	12
SDS-V3-plasma-45_Cluster_2859_sequences=12	282	12
SDS-V3-plasma-45_Cluster_1246_sequences=12	282	12
SDS-V3-plasma-45_Cluster_24416_sequences=12	282	12
SDS-V3-plasma-45_Cluster_9970_sequences=12	282	12
SDS-V3-plasma-45_Cluster_10315_sequences=12	282	12
SDS-V3-plasma-45_Cluster_1318_sequences=12	282	12
SDS-V3-plasma-45_Cluster_2317_sequences=12	282	12
SDS-V3-plasma-45_Cluster_10114_sequences=12	282	12
SDS-V3-plasma-45_Cluster_1125_sequences=12	282	12
SDS-V3-plasma-45_Cluster_15226_sequences=12	282	12
SDS-V3-plasma-45_Cluster_15477_sequences=12	282	12
SDS-V3-plasma-45_Cluster_1376_sequences=12	282	12
SDS-V3-plasma-45_Cluster_22585_sequences=12	282	12
SDS-V3-plasma-45_Cluster_12238_sequences=12	282	12
SDS-V3-plasma-45_Cluster_18368_sequences=12	282	12
SDS-V3-plasma-45_Cluster_4441_sequences=12	282	12
SDS-V3-plasma-45_Cluster_10891_sequences=12	282	12
SDS-V3-plasma-45_Cluster_24993_sequences=12	282	12

SDS-V3-plasma-45_Cluster_10980_sequences=12	282	12
SDS-V3-plasma-45_Cluster_22292_sequences=12	282	12
SDS-V3-plasma-45_Cluster_18273_sequences=12	282	12
SDS-V3-plasma-45_Cluster_24353_sequences=12	282	12
SDS-V3-plasma-45_Cluster_26493_sequences=12	282	12
SDS-V3-plasma-45_Cluster_34736_sequences=12	282	12
SDS-V3-plasma-45_Cluster_65798_sequences=12	282	12
SDS-V3-plasma-45_Cluster_8841_sequences=12	282	12
SDS-V3-plasma-45_Cluster_1606_sequences=12	282	12
SDS-V3-plasma-45_Cluster_14012_sequences=12	282	12
SDS-V3-plasma-45_Cluster_8220_sequences=12	282	12
SDS-V3-plasma-45_Cluster_4777_sequences=12	282	12
SDS-V3-plasma-45_Cluster_4152_sequences=12	282	12
SDS-V3-plasma-45_Cluster_21284_sequences=12	282	12
SDS-V3-plasma-45_Cluster_66625_sequences=12	282	12
SDS-V3-plasma-45_Cluster_8601_sequences=12	282	12
SDS-V3-plasma-45_Cluster_6246_sequences=12	282	12
SDS-V3-plasma-45_Cluster_10261_sequences=12	282	12
SDS-V3-plasma-45_Cluster_19788_sequences=12	282	12
SDS-V3-plasma-45_Cluster_8600_sequences=12	282	12
SDS-V3-plasma-45_Cluster_31837_sequences=12	282	12
SDS-V3-plasma-45_Cluster_5728_sequences=12	282	12
SDS-V3-plasma-45_Cluster_3090_sequences=12	282	12
SDS-V3-plasma-45_Cluster_22050_sequences=12	282	12
SDS-V3-plasma-45_Cluster_5394_sequences=12	282	12
SDS-V3-plasma-45_Cluster_7028_sequences=12	282	12
SDS-V3-plasma-45_Cluster_7683_sequences=12	282	12
SDS-V3-plasma-45_Cluster_14162_sequences=12	282	12
SDS-V3-plasma-45_Cluster_12454_sequences=12	282	12
SDS-V3-plasma-45_Cluster_87210_sequences=12	282	12
SDS-V3-plasma-45_Cluster_12155_sequences=12	282	12
SDS-V3-plasma-45_Cluster_16459_sequences=12	282	12
SDS-V3-plasma-45_Cluster_20304_sequences=12	282	12
SDS-V3-plasma-45_Cluster_26700_sequences=12	282	12
SDS-V3-plasma-45_Cluster_18392_sequences=12	282	12
SDS-V3-plasma-45_Cluster_29747_sequences=12	282	12
SDS-V3-plasma-45_Cluster_13654_sequences=12	282	12
SDS-V3-plasma-45_Cluster_5163_sequences=12	282	12
SDS-V3-plasma-45_Cluster_6784_sequences=12	282	12
SDS-V3-plasma-45_Cluster_2739_sequences=12	282	12
SDS-V3-plasma-45_Cluster_16786_sequences=12	282	12
SDS-V3-plasma-45_Cluster_9148_sequences=12	282	12
SDS-V3-plasma-45_Cluster_19936_sequences=12	282	12
SDS-V3-plasma-45_Cluster_30715_sequences=12	282	12
SDS-V3-plasma-45_Cluster_8136_sequences=12	282	12

SDS-V3-plasma-45_Cluster_3925_sequences=12	282	12
SDS-V3-plasma-45_Cluster_19221_sequences=12	282	12
SDS-V3-plasma-45_Cluster_16988_sequences=12	282	12
SDS-V3-plasma-45_Cluster_4186_sequences=12	282	12
SDS-V3-plasma-45_Cluster_3648_sequences=12	282	12
SDS-V3-plasma-45_Cluster_5943_sequences=12	282	12
SDS-V3-plasma-45_Cluster_14786_sequences=12	282	12
SDS-V3-plasma-45_Cluster_11399_sequences=12	282	12
SDS-V3-plasma-45_Cluster_27962_sequences=12	282	12
SDS-V3-plasma-45_Cluster_8702_sequences=12	282	12
SDS-V3-plasma-45_Cluster_5901_sequences=12	282	12
SDS-V3-plasma-45_Cluster_1710_sequences=12	282	12
SDS-V3-plasma-45_Cluster_8349_sequences=12	282	12
SDS-V3-plasma-45_Cluster_6165_sequences=12	282	12
SDS-V3-plasma-45_Cluster_4614_sequences=12	282	12
SDS-V3-plasma-45_Cluster_4161_sequences=12	282	12
SDS-V3-plasma-45_Cluster_4733_sequences=12	282	12
SDS-V3-plasma-45_Cluster_10765_sequences=12	282	12
SDS-V3-plasma-45_Cluster_30457_sequences=12	282	12
SDS-V3-plasma-45_Cluster_50574_sequences=12	282	12
SDS-V3-plasma-45_Cluster_4210_sequences=12	282	12
SDS-V3-plasma-45_Cluster_11377_sequences=12	282	12
SDS-V3-plasma-45_Cluster_11873_sequences=12	282	12
SDS-V3-plasma-45_Cluster_12293_sequences=12	282	12
SDS-V3-plasma-45_Cluster_13280_sequences=12	282	12
SDS-V3-plasma-45_Cluster_341_sequences=12	282	12
SDS-V3-plasma-45_Cluster_3689_sequences=12	282	12
SDS-V3-plasma-45_Cluster_7692_sequences=12	282	12
SDS-V3-plasma-45_Cluster_8995_sequences=12	282	12
SDS-V3-plasma-45_Cluster_156_sequences=12	282	12
SDS-V3-plasma-45_Cluster_16193_sequences=12	282	12
SDS-V3-plasma-45_Cluster_15648_sequences=12	282	12
SDS-V3-plasma-45_Cluster_10865_sequences=12	282	12
SDS-V3-plasma-45_Cluster_7905_sequences=12	282	12
SDS-V3-plasma-45_Cluster_5057_sequences=12	282	12
SDS-V3-plasma-45_Cluster_769_sequences=12	282	12
SDS-V3-plasma-45_Cluster_12417_sequences=12	282	12
SDS-V3-plasma-45_Cluster_25284_sequences=12	282	12
SDS-V3-plasma-45_Cluster_4751_sequences=12	282	12
SDS-V3-plasma-45_Cluster_5524_sequences=12	282	12
SDS-V3-plasma-45_Cluster_29534_sequences=12	282	12
SDS-V3-plasma-45_Cluster_16470_sequences=12	282	12
SDS-V3-plasma-45_Cluster_10139_sequences=12	282	12
SDS-V3-plasma-45_Cluster_16781_sequences=12	282	12
SDS-V3-plasma-45_Cluster_4684_sequences=12	282	12

SDS-V3-plasma-45_Cluster_3222_sequences=12	282	12
SDS-V3-plasma-45_Cluster_27550_sequences=12	282	12
SDS-V3-plasma-45_Cluster_12374_sequences=12	282	12
SDS-V3-plasma-45_Cluster_24042_sequences=12	282	12
SDS-V3-plasma-45_Cluster_10074_sequences=12	282	12
SDS-V3-plasma-45_Cluster_25610_sequences=12	282	12
SDS-V3-plasma-45_Cluster_25286_sequences=12	282	12
SDS-V3-plasma-45_Cluster_18582_sequences=12	282	12
SDS-V3-plasma-45_Cluster_20868_sequences=12	282	12
SDS-V3-plasma-45_Cluster_601_sequences=12	282	12
SDS-V3-plasma-45_Cluster_13548_sequences=12	282	12
SDS-V3-plasma-45_Cluster_26561_sequences=12	282	12
SDS-V3-plasma-45_Cluster_4603_sequences=12	282	12
SDS-V3-plasma-45_Cluster_14330_sequences=12	282	12
SDS-V3-plasma-45_Cluster_14338_sequences=12	282	12
SDS-V3-plasma-45_Cluster_14397_sequences=12	282	12
SDS-V3-plasma-45_Cluster_14968_sequences=12	282	12
SDS-V3-plasma-45_Cluster_24771_sequences=12	282	12
SDS-V3-plasma-45_Cluster_4527_sequences=12	282	12
SDS-V3-plasma-45_Cluster_4889_sequences=12	282	12
SDS-V3-plasma-45_Cluster_15514_sequences=12	282	12
SDS-V3-plasma-45_Cluster_12779_sequences=12	282	12
SDS-V3-plasma-45_Cluster_16247_sequences=12	282	12
SDS-V3-plasma-45_Cluster_10196_sequences=12	282	12
SDS-V3-plasma-46_Cluster_1645_sequences=12	286	12
SDS-V3-plasma-46_Cluster_722_sequences=12	286	12
SDS-V3-plasma-46_Cluster_10119_sequences=12	286	12
SDS-V3-plasma-46_Cluster_1038_sequences=12	286	12
SDS-V3-plasma-46_Cluster_17139_sequences=12	286	12
SDS-V3-plasma-46_Cluster_15938_sequences=12	286	12
SDS-V3-plasma-46_Cluster_4593_sequences=12	286	12
SDS-V3-plasma-46_Cluster_648_sequences=12	286	12
SDS-V3-plasma-46_Cluster_12257_sequences=12	286	12
SDS-V3-plasma-46_Cluster_3750_sequences=12	286	12
SDS-V3-plasma-46_Cluster_6015_sequences=12	286	12
SDS-V3-plasma-46_Cluster_1949_sequences=12	286	12
SDS-V3-plasma-46_Cluster_1953_sequences=12	286	12
SDS-V3-plasma-46_Cluster_6098_sequences=12	286	12
SDS-V3-plasma-46_Cluster_6512_sequences=12	286	12
SDS-V3-plasma-46_Cluster_5727_sequences=12	286	12
SDS-V3-plasma-46_Cluster_1256_sequences=12	286	12
SDS-V3-plasma-46_Cluster_721_sequences=12	286	12
SDS-V3-plasma-46_Cluster_8407_sequences=12	286	12
SDS-V3-plasma-46_Cluster_10243_sequences=12	286	12
SDS-V3-plasma-46_Cluster_11861_sequences=12	286	12

SDS-V3-plasma-46_Cluster_5393_sequences=12	286	12
SDS-V3-plasma-46_Cluster_611_sequences=12	286	12
SDS-V3-plasma-46_Cluster_9667_sequences=12	286	12
SDS-V3-plasma-46_Cluster_3012_sequences=12	286	12
SDS-V3-plasma-46_Cluster_7129_sequences=12	286	12
SDS-V3-plasma-46_Cluster_5329_sequences=12	286	12
SDS-V3-plasma-46_Cluster_4668_sequences=12	286	12
SDS-V3-plasma-46_Cluster_10416_sequences=12	286	12
SDS-V3-plasma-46_Cluster_1069_sequences=12	286	12
SDS-V3-plasma-46_Cluster_3691_sequences=12	286	12
SDS-V3-plasma-46_Cluster_5078_sequences=12	286	12
SDS-V3-plasma-46_Cluster_4799_sequences=12	286	12
SDS-V3-plasma-46_Cluster_1648_sequences=12	286	12
SDS-V3-plasma-46_Cluster_2750_sequences=12	286	12
SDS-V3-plasma-46_Cluster_1643_sequences=12	286	12
SDS-V3-plasma-46_Cluster_2070_sequences=12	286	12
SDS-V3-plasma-46_Cluster_3286_sequences=12	286	12
SDS-V3-plasma-46_Cluster_4466_sequences=12	286	12
SDS-V3-plasma-46_Cluster_4700_sequences=12	286	12
SDS-V3-plasma-46_Cluster_2353_sequences=12	286	12
SDS-V3-plasma-46_Cluster_1235_sequences=12	286	12
SDS-V3-plasma-46_Cluster_1315_sequences=12	286	12
SDS-V3-plasma-46_Cluster_167_sequences=12	286	12
SDS-V3-plasma-46_Cluster_2570_sequences=12	286	12
SDS-V3-plasma-46_Cluster_2575_sequences=12	286	12
SDS-V3-plasma-46_Cluster_3171_sequences=12	286	12
SDS-V3-plasma-46_Cluster_3488_sequences=12	286	12
SDS-V3-plasma-46_Cluster_3879_sequences=12	286	12
SDS-V3-plasma-46_Cluster_6007_sequences=12	286	12
SDS-V3-plasma-46_Cluster_6078_sequences=12	286	12
SDS-V3-plasma-46_Cluster_13797_sequences=12	286	12
SDS-V3-plasma-46_Cluster_179_sequences=12	286	12
SDS-V3-plasma-46_Cluster_8276_sequences=12	286	12
SDS-V3-plasma-46_Cluster_9847_sequences=12	286	12
SDS-V3-plasma-46_Cluster_2477_sequences=12	286	12
SDS-V3-plasma-46_Cluster_3302_sequences=12	286	12
SDS-V3-plasma-46_Cluster_5806_sequences=12	286	12
SDS-V3-plasma-46_Cluster_5916_sequences=12	286	12
SDS-V3-plasma-46_Cluster_212_sequences=12	286	12
SDS-V3-plasma-46_Cluster_1280_sequences=12	286	12
SDS-V3-plasma-46_Cluster_5134_sequences=12	286	12
SDS-V3-plasma-46_Cluster_7396_sequences=12	286	12
SDS-V3-plasma-46_Cluster_4832_sequences=12	286	12
SDS-V3-plasma-46_Cluster_699_sequences=12	286	12
SDS-V3-plasma-46_Cluster_834_sequences=12	286	12

SDS-V3-plasma-46_Cluster_697_sequences=12	286	12
SDS-V3-plasma-46_Cluster_13567_sequences=12	286	12
SDS-V3-plasma-46_Cluster_6400_sequences=12	286	12
SDS-V3-plasma-46_Cluster_1194_sequences=12	286	12
SDS-V3-plasma-46_Cluster_4726_sequences=12	286	12
SDS-V3-plasma-46_Cluster_8833_sequences=12	286	12
SDS-V3-plasma-46_Cluster_2640_sequences=12	286	12
SDS-V3-plasma-46_Cluster_5403_sequences=12	286	12
SDS-V3-plasma-46_Cluster_5034_sequences=12	286	12
SDS-V3-plasma-46_Cluster_1140_sequences=12	286	12
SDS-V3-plasma-46_Cluster_7267_sequences=12	286	12
SDS-V3-plasma-46_Cluster_998_sequences=12	286	12
SDS-V3-plasma-46_Cluster_6137_sequences=12	286	12
SDS-V3-plasma-46_Cluster_5778_sequences=12	286	12
SDS-V3-plasma-46_Cluster_893_sequences=12	286	12
SDS-V3-plasma-46_Cluster_5737_sequences=12	286	12
SDS-V3-plasma-67_Cluster_26617_sequences=12	504	12
SDS-V3-plasma-67_Cluster_29550_sequences=12	504	12
SDS-V3-plasma-67_Cluster_14843_sequences=12	504	12
SDS-V3-plasma-67_Cluster_4048_sequences=12	504	12
SDS-V3-plasma-67_Cluster_297_sequences=12	504	12
SDS-V3-plasma-67_Cluster_10516_sequences=12	504	12
SDS-V3-plasma-67_Cluster_40344_sequences=12	504	12
SDS-V3-plasma-67_Cluster_18829_sequences=12	504	12
SDS-V3-plasma-67_Cluster_12502_sequences=12	504	12
SDS-V3-plasma-67_Cluster_52849_sequences=12	504	12
SDS-V3-plasma-67_Cluster_6855_sequences=12	504	12
SDS-V3-plasma-67_Cluster_14201_sequences=12	504	12
SDS-V3-plasma-67_Cluster_100396_sequences=12	504	12
SDS-V3-plasma-67_Cluster_2823_sequences=12	504	12
SDS-V3-plasma-67_Cluster_4332_sequences=12	504	12
SDS-V3-plasma-67_Cluster_40614_sequences=12	504	12
SDS-V3-plasma-67_Cluster_32404_sequences=12	504	12
SDS-V3-plasma-67_Cluster_19531_sequences=12	504	12
SDS-V3-plasma-67_Cluster_10418_sequences=12	504	12
SDS-V3-plasma-67_Cluster_8417_sequences=12	504	12
SDS-V3-plasma-67_Cluster_5117_sequences=12	504	12
SDS-V3-plasma-67_Cluster_12588_sequences=12	504	12
SDS-V3-plasma-67_Cluster_9826_sequences=12	504	12
SDS-V3-plasma-67_Cluster_15245_sequences=12	504	12
SDS-V3-plasma-67_Cluster_6067_sequences=12	504	12
SDS-V3-plasma-67_Cluster_19122_sequences=12	504	12
SDS-V3-plasma-67_Cluster_28823_sequences=12	504	12
SDS-V3-plasma-67_Cluster_4747_sequences=12	504	12
SDS-V3-plasma-67_Cluster_15024_sequences=12	504	12

SDS-V3-plasma-67_Cluster_18410_sequences=12	504	12
SDS-V3-plasma-67_Cluster_38216_sequences=12	504	12
SDS-V3-plasma-67_Cluster_6341_sequences=12	504	12
SDS-V3-plasma-67_Cluster_7684_sequences=12	504	12
SDS-V3-plasma-67_Cluster_1780_sequences=12	504	12
SDS-V3-plasma-67_Cluster_6578_sequences=12	504	12
SDS-V3-plasma-67_Cluster_6462_sequences=12	504	12
SDS-V3-plasma-67_Cluster_14779_sequences=12	504	12
SDS-V3-plasma-67_Cluster_16972_sequences=12	504	12
SDS-V3-plasma-67_Cluster_5973_sequences=12	504	12
SDS-V3-plasma-67_Cluster_10461_sequences=12	504	12
SDS-V3-plasma-67_Cluster_4824_sequences=12	504	12
SDS-V3-plasma-67_Cluster_18533_sequences=12	504	12
SDS-V3-plasma-67_Cluster_15784_sequences=12	504	12
SDS-V3-plasma-67_Cluster_23045_sequences=12	504	12
SDS-V3-plasma-67_Cluster_7964_sequences=12	504	12
SDS-V3-plasma-67_Cluster_21691_sequences=12	504	12
SDS-V3-plasma-67_Cluster_11301_sequences=12	504	12
SDS-V3-plasma-67_Cluster_11551_sequences=12	504	12
SDS-V3-plasma-67_Cluster_9941_sequences=12	504	12
SDS-V3-plasma-67_Cluster_7288_sequences=12	504	12
SDS-V3-plasma-67_Cluster_9554_sequences=12	504	12
SDS-V3-plasma-67_Cluster_5471_sequences=12	504	12
SDS-V3-plasma-67_Cluster_18396_sequences=12	504	12
SDS-V3-plasma-67_Cluster_3841_sequences=12	504	12
SDS-V3-plasma-67_Cluster_5010_sequences=12	504	12
SDS-V3-plasma-67_Cluster_14296_sequences=12	504	12
SDS-V3-plasma-67_Cluster_20189_sequences=12	504	12
SDS-V3-plasma-67_Cluster_20318_sequences=12	504	12
SDS-V3-plasma-67_Cluster_75590_sequences=12	504	12
SDS-V3-plasma-67_Cluster_54890_sequences=12	504	12
SDS-V3-plasma-67_Cluster_5452_sequences=12	504	12
SDS-V3-plasma-67_Cluster_10809_sequences=12	504	12
SDS-V3-plasma-67_Cluster_3825_sequences=12	504	12
SDS-V3-plasma-67_Cluster_9765_sequences=12	504	12
SDS-V3-plasma-67_Cluster_32008_sequences=12	504	12
SDS-V3-plasma-67_Cluster_4179_sequences=12	504	12
SDS-V3-plasma-67_Cluster_30228_sequences=12	504	12
SDS-V3-plasma-67_Cluster_326_sequences=12	504	12
SDS-V3-plasma-67_Cluster_6178_sequences=12	504	12
SDS-V3-plasma-67_Cluster_6737_sequences=12	504	12
SDS-V3-plasma-67_Cluster_7839_sequences=12	504	12
SDS-V3-plasma-67_Cluster_7956_sequences=12	504	12
SDS-V3-plasma-67_Cluster_9809_sequences=12	504	12
SDS-V3-plasma-67_Cluster_14397_sequences=12	504	12

SDS-V3-plasma-67_Cluster_7383_sequences=12	504	12
SDS-V3-plasma-67_Cluster_5278_sequences=12	504	12
SDS-V3-plasma-67_Cluster_32980_sequences=12	504	12
SDS-V3-plasma-67_Cluster_1486_sequences=12	504	12
SDS-V3-plasma-67_Cluster_1745_sequences=12	504	12
SDS-V3-plasma-67_Cluster_19753_sequences=12	504	12
SDS-V3-plasma-67_Cluster_30188_sequences=12	504	12
SDS-V3-plasma-67_Cluster_8251_sequences=12	504	12
SDS-V3-plasma-67_Cluster_9004_sequences=12	504	12
SDS-V3-plasma-67_Cluster_30562_sequences=12	504	12
SDS-V3-plasma-67_Cluster_55_sequences=12	504	12
SDS-V3-plasma-67_Cluster_19986_sequences=12	504	12
SDS-V3-plasma-67_Cluster_22328_sequences=12	504	12
SDS-V3-plasma-67_Cluster_5796_sequences=12	504	12
SDS-V3-plasma-67_Cluster_5184_sequences=12	504	12
SDS-V3-plasma-67_Cluster_16843_sequences=12	504	12
SDS-V3-plasma-67_Cluster_2188_sequences=12	504	12
SDS-V3-plasma-67_Cluster_12525_sequences=12	504	12
SDS-V3-plasma-67_Cluster_7623_sequences=12	504	12
SDS-V3-plasma-67_Cluster_7355_sequences=12	504	12
SDS-V3-plasma-67_Cluster_2913_sequences=12	504	12
SDS-V3-plasma-67_Cluster_49477_sequences=12	504	12
SDS-V3-plasma-67_Cluster_14502_sequences=12	504	12
SDS-V3-plasma-67_Cluster_11372_sequences=12	504	12
SDS-V3-plasma-67_Cluster_48691_sequences=12	504	12
SDS-V3-plasma-67_Cluster_6372_sequences=12	504	12
SDS-V3-plasma-67_Cluster_1968_sequences=12	504	12
SDS-V3-plasma-67_Cluster_2165_sequences=12	504	12
SDS-V3-plasma-67_Cluster_10346_sequences=12	504	12
SDS-V3-plasma-67_Cluster_54129_sequences=12	504	12
SDS-V3-plasma-67_Cluster_353_sequences=12	504	12
SDS-V3-plasma-67_Cluster_52586_sequences=12	504	12
SDS-V3-plasma-67_Cluster_7934_sequences=12	504	12
SDS-V3-plasma-67_Cluster_17037_sequences=12	504	12
SDS-V3-plasma-67_Cluster_12439_sequences=12	504	12
SDS-V3-plasma-67_Cluster_1957_sequences=12	504	12
SDS-V3-plasma-67_Cluster_19645_sequences=12	504	12
SDS-V3-plasma-67_Cluster_26465_sequences=12	504	12
SDS-V3-plasma-67_Cluster_31850_sequences=12	504	12
SDS-V3-plasma-67_Cluster_6724_sequences=12	504	12
SDS-V3-plasma-67_Cluster_75574_sequences=12	504	12
SDS-V3-plasma-67_Cluster_76618_sequences=12	504	12
SDS-V3-plasma-67_Cluster_10534_sequences=12	504	12
SDS-V3-plasma-67_Cluster_4662_sequences=12	504	12
SDS-V3-plasma-67_Cluster_13273_sequences=12	504	12

SDS-V3-plasma-67_Cluster_16195_sequences=12	504	12
SDS-V3-plasma-67_Cluster_22252_sequences=12	504	12
SDS-V3-plasma-67_Cluster_22253_sequences=12	504	12
SDS-V3-plasma-67_Cluster_8259_sequences=12	504	12
SDS-V3-plasma-67_Cluster_12098_sequences=12	504	12
SDS-V3-plasma-67_Cluster_15200_sequences=12	504	12
SDS-V3-plasma-67_Cluster_39446_sequences=12	504	12
SDS-V3-plasma-67_Cluster_18641_sequences=12	504	12
SDS-V3-plasma-67_Cluster_3914_sequences=12	504	12
SDS-V3-plasma-67_Cluster_26717_sequences=12	504	12
SDS-V3-plasma-67_Cluster_16541_sequences=12	504	12
SDS-V3-plasma-67_Cluster_22721_sequences=12	504	12
SDS-V3-plasma-67_Cluster_25526_sequences=12	504	12
SDS-V3-plasma-67_Cluster_27975_sequences=12	504	12
SDS-V3-plasma-67_Cluster_29311_sequences=12	504	12
SDS-V3-plasma-67_Cluster_31945_sequences=12	504	12
SDS-V3-plasma-67_Cluster_11652_sequences=12	504	12
SDS-V3-plasma-67_Cluster_21609_sequences=12	504	12
SDS-V3-plasma-67_Cluster_22463_sequences=12	504	12
SDS-V3-plasma-67_Cluster_59546_sequences=12	504	12
SDS-V3-plasma-67_Cluster_6225_sequences=12	504	12
SDS-V3-plasma-67_Cluster_15439_sequences=12	504	12
SDS-V3-plasma-67_Cluster_27459_sequences=12	504	12
SDS-V3-plasma-67_Cluster_6120_sequences=12	504	12
SDS-V3-plasma-67_Cluster_76748_sequences=12	504	12
SDS-V3-plasma-67_Cluster_27650_sequences=12	504	12
SDS-V3-plasma-67_Cluster_45617_sequences=12	504	12
SDS-V3-plasma-67_Cluster_8170_sequences=12	504	12
SDS-V3-plasma-67_Cluster_8882_sequences=12	504	12
SDS-V3-plasma-67_Cluster_2491_sequences=12	504	12
SDS-V3-plasma-67_Cluster_12309_sequences=12	504	12
SDS-V3-plasma-67_Cluster_14899_sequences=12	504	12
SDS-V3-plasma-67_Cluster_17594_sequences=12	504	12
SDS-V3-plasma-67_Cluster_18402_sequences=12	504	12
SDS-V3-plasma-67_Cluster_19736_sequences=12	504	12
SDS-V3-plasma-67_Cluster_37024_sequences=12	504	12
SDS-V3-plasma-67_Cluster_18590_sequences=12	504	12
SDS-V3-plasma-0_Cluster_7187_sequences=11	0	11
SDS-V3-plasma-0_Cluster_6038_sequences=11	0	11
SDS-V3-plasma-0_Cluster_6322_sequences=11	0	11
SDS-V3-plasma-0_Cluster_7415_sequences=11	0	11
SDS-V3-plasma-0_Cluster_2280_sequences=11	0	11
SDS-V3-plasma-0_Cluster_2986_sequences=11	0	11
SDS-V3-plasma-0_Cluster_10449_sequences=11	0	11
SDS-V3-plasma-0_Cluster_10653_sequences=11	0	11

SDS-V3-plasma-0_Cluster_16210_sequences=11	0	11
SDS-V3-plasma-0_Cluster_1910_sequences=11	0	11
SDS-V3-plasma-0_Cluster_196_sequences=11	0	11
SDS-V3-plasma-0_Cluster_203_sequences=11	0	11
SDS-V3-plasma-0_Cluster_2544_sequences=11	0	11
SDS-V3-plasma-0_Cluster_3685_sequences=11	0	11
SDS-V3-plasma-0_Cluster_6422_sequences=11	0	11
SDS-V3-plasma-0_Cluster_9450_sequences=11	0	11
SDS-V3-plasma-0_Cluster_12961_sequences=11	0	11
SDS-V3-plasma-0_Cluster_24643_sequences=11	0	11
SDS-V3-plasma-0_Cluster_19614_sequences=11	0	11
SDS-V3-plasma-0_Cluster_1787_sequences=11	0	11
SDS-V3-plasma-0_Cluster_1078_sequences=11	0	11
SDS-V3-plasma-0_Cluster_5185_sequences=11	0	11
SDS-V3-plasma-0_Cluster_7982_sequences=11	0	11
SDS-V3-plasma-0_Cluster_6706_sequences=11	0	11
SDS-V3-plasma-0_Cluster_2839_sequences=11	0	11
SDS-V3-plasma-0_Cluster_3706_sequences=11	0	11
SDS-V3-plasma-0_Cluster_1588_sequences=11	0	11
SDS-V3-plasma-0_Cluster_3629_sequences=11	0	11
SDS-V3-plasma-0_Cluster_2145_sequences=11	0	11
SDS-V3-plasma-0_Cluster_9331_sequences=11	0	11
SDS-V3-plasma-0_Cluster_6434_sequences=11	0	11
SDS-V3-plasma-0_Cluster_6536_sequences=11	0	11
SDS-V3-plasma-0_Cluster_6792_sequences=11	0	11
SDS-V3-plasma-0_Cluster_4350_sequences=11	0	11
SDS-V3-plasma-0_Cluster_3383_sequences=11	0	11
SDS-V3-plasma-0_Cluster_7038_sequences=11	0	11
SDS-V3-plasma-0_Cluster_2958_sequences=11	0	11
SDS-V3-plasma-0_Cluster_7699_sequences=11	0	11
SDS-V3-plasma-0_Cluster_6529_sequences=11	0	11
SDS-V3-plasma-0_Cluster_8586_sequences=11	0	11
SDS-V3-plasma-0_Cluster_8872_sequences=11	0	11
SDS-V3-plasma-0_Cluster_1316_sequences=11	0	11
SDS-V3-plasma-0_Cluster_15098_sequences=11	0	11
SDS-V3-plasma-0_Cluster_1667_sequences=11	0	11
SDS-V3-plasma-0_Cluster_2755_sequences=11	0	11
SDS-V3-plasma-0_Cluster_25178_sequences=11	0	11
SDS-V3-plasma-0_Cluster_4169_sequences=11	0	11
SDS-V3-plasma-0_Cluster_8171_sequences=11	0	11
SDS-V3-plasma-0_Cluster_735_sequences=11	0	11
SDS-V3-plasma-0_Cluster_19306_sequences=11	0	11
SDS-V3-plasma-0_Cluster_2889_sequences=11	0	11
SDS-V3-plasma-0_Cluster_3821_sequences=11	0	11
SDS-V3-plasma-0_Cluster_7020_sequences=11	0	11

SDS-V3-plasma-0_Cluster_810_sequences=11	0	11
SDS-V3-plasma-0_Cluster_8228_sequences=11	0	11
SDS-V3-plasma-0_Cluster_975_sequences=11	0	11
SDS-V3-plasma-0_Cluster_13894_sequences=11	0	11
SDS-V3-plasma-0_Cluster_17368_sequences=11	0	11
SDS-V3-plasma-0_Cluster_3197_sequences=11	0	11
SDS-V3-plasma-0_Cluster_1402_sequences=11	0	11
SDS-V3-plasma-0_Cluster_4696_sequences=11	0	11
SDS-V3-plasma-0_Cluster_3297_sequences=11	0	11
SDS-V3-plasma-0_Cluster_3724_sequences=11	0	11
SDS-V3-plasma-0_Cluster_3460_sequences=11	0	11
SDS-V3-plasma-0_Cluster_9515_sequences=11	0	11
SDS-V3-plasma-0_Cluster_3887_sequences=11	0	11
SDS-V3-plasma-0_Cluster_3032_sequences=11	0	11
SDS-V3-plasma-0_Cluster_3131_sequences=11	0	11
SDS-V3-plasma-0_Cluster_3082_sequences=11	0	11
SDS-V3-plasma-0_Cluster_2446_sequences=11	0	11
SDS-V3-plasma-0_Cluster_7125_sequences=11	0	11
SDS-V3-plasma-0_Cluster_9496_sequences=11	0	11
SDS-V3-plasma-0_Cluster_8260_sequences=11	0	11
SDS-V3-plasma-0_Cluster_6903_sequences=11	0	11
SDS-V3-plasma-0_Cluster_4353_sequences=11	0	11
SDS-V3-plasma-5_Cluster_147_sequences=11	9	11
SDS-V3-plasma-5_Cluster_152_sequences=11	9	11
SDS-V3-plasma-5_Cluster_1615_sequences=11	9	11
SDS-V3-plasma-5_Cluster_312_sequences=11	9	11
SDS-V3-plasma-5_Cluster_382_sequences=11	9	11
SDS-V3-plasma-5_Cluster_445_sequences=11	9	11
SDS-V3-plasma-5_Cluster_556_sequences=11	9	11
SDS-V3-plasma-5_Cluster_703_sequences=11	9	11
SDS-V3-plasma-5_Cluster_726_sequences=11	9	11
SDS-V3-plasma-5_Cluster_387_sequences=11	9	11
SDS-V3-plasma-5_Cluster_414_sequences=11	9	11
SDS-V3-plasma-5_Cluster_181_sequences=11	9	11
SDS-V3-plasma-5_Cluster_197_sequences=11	9	11
SDS-V3-plasma-5_Cluster_315_sequences=11	9	11
SDS-V3-plasma-5_Cluster_429_sequences=11	9	11
SDS-V3-plasma-5_Cluster_957_sequences=11	9	11
SDS-V3-plasma-5_Cluster_572_sequences=11	9	11
SDS-V3-plasma-5_Cluster_573_sequences=11	9	11
SDS-V3-plasma-5_Cluster_586_sequences=11	9	11
SDS-V3-plasma-5_Cluster_73_sequences=11	9	11
SDS-V3-plasma-5_Cluster_965_sequences=11	9	11
SDS-V3-plasma-7_Cluster_161_sequences=11	14	11
SDS-V3-plasma-7_Cluster_1752_sequences=11	14	11

SDS-V3-plasma-7_Cluster_342_sequences=11	14	11
SDS-V3-plasma-7_Cluster_1385_sequences=11	14	11
SDS-V3-plasma-7_Cluster_4103_sequences=11	14	11
SDS-V3-plasma-7_Cluster_979_sequences=11	14	11
SDS-V3-plasma-7_Cluster_4493_sequences=11	14	11
SDS-V3-plasma-7_Cluster_4747_sequences=11	14	11
SDS-V3-plasma-7_Cluster_3578_sequences=11	14	11
SDS-V3-plasma-7_Cluster_247_sequences=11	14	11
SDS-V3-plasma-7_Cluster_3565_sequences=11	14	11
SDS-V3-plasma-7_Cluster_1480_sequences=11	14	11
SDS-V3-plasma-7_Cluster_1777_sequences=11	14	11
SDS-V3-plasma-7_Cluster_401_sequences=11	14	11
SDS-V3-plasma-7_Cluster_566_sequences=11	14	11
SDS-V3-plasma-7_Cluster_1886_sequences=11	14	11
SDS-V3-plasma-7_Cluster_608_sequences=11	14	11
SDS-V3-plasma-7_Cluster_8015_sequences=11	14	11
SDS-V3-plasma-7_Cluster_3998_sequences=11	14	11
SDS-V3-plasma-7_Cluster_4090_sequences=11	14	11
SDS-V3-plasma-7_Cluster_558_sequences=11	14	11
SDS-V3-plasma-7_Cluster_2930_sequences=11	14	11
SDS-V3-plasma-7_Cluster_1422_sequences=11	14	11
SDS-V3-plasma-7_Cluster_2163_sequences=11	14	11
SDS-V3-plasma-7_Cluster_974_sequences=11	14	11
SDS-V3-plasma-7_Cluster_1785_sequences=11	14	11
SDS-V3-plasma-7_Cluster_3454_sequences=11	14	11
SDS-V3-plasma-7_Cluster_1694_sequences=11	14	11
SDS-V3-plasma-7_Cluster_4170_sequences=11	14	11
SDS-V3-plasma-7_Cluster_1399_sequences=11	14	11
SDS-V3-plasma-8_Cluster_647_sequences=11	16	11
SDS-V3-plasma-8_Cluster_236_sequences=11	16	11
SDS-V3-plasma-8_Cluster_396_sequences=11	16	11
SDS-V3-plasma-8_Cluster_2265_sequences=11	16	11
SDS-V3-plasma-8_Cluster_3034_sequences=11	16	11
SDS-V3-plasma-8_Cluster_2571_sequences=11	16	11
SDS-V3-plasma-8_Cluster_3004_sequences=11	16	11
SDS-V3-plasma-8_Cluster_6138_sequences=11	16	11
SDS-V3-plasma-8_Cluster_3939_sequences=11	16	11
SDS-V3-plasma-8_Cluster_3178_sequences=11	16	11
SDS-V3-plasma-8_Cluster_5058_sequences=11	16	11
SDS-V3-plasma-8_Cluster_3885_sequences=11	16	11
SDS-V3-plasma-8_Cluster_3187_sequences=11	16	11
SDS-V3-plasma-8_Cluster_3316_sequences=11	16	11
SDS-V3-plasma-8_Cluster_2317_sequences=11	16	11
SDS-V3-plasma-8_Cluster_2746_sequences=11	16	11
SDS-V3-plasma-8_Cluster_2934_sequences=11	16	11

SDS-V3-plasma-8_Cluster_3849_sequences=11	16	11
SDS-V3-plasma-8_Cluster_4073_sequences=11	16	11
SDS-V3-plasma-8_Cluster_2800_sequences=11	16	11
SDS-V3-plasma-8_Cluster_2697_sequences=11	16	11
SDS-V3-plasma-8_Cluster_3107_sequences=11	16	11
SDS-V3-plasma-8_Cluster_3204_sequences=11	16	11
SDS-V3-plasma-8_Cluster_4739_sequences=11	16	11
SDS-V3-plasma-8_Cluster_4263_sequences=11	16	11
SDS-V3-plasma-8_Cluster_2400_sequences=11	16	11
SDS-V3-plasma-8_Cluster_6424_sequences=11	16	11
SDS-V3-plasma-8_Cluster_2801_sequences=11	16	11
SDS-V3-plasma-8_Cluster_4224_sequences=11	16	11
SDS-V3-plasma-8_Cluster_3049_sequences=11	16	11
SDS-V3-plasma-8_Cluster_3613_sequences=11	16	11
SDS-V3-plasma-8_Cluster_2777_sequences=11	16	11
SDS-V3-plasma-8_Cluster_3749_sequences=11	16	11
SDS-V3-plasma-8_Cluster_3777_sequences=11	16	11
SDS-V3-plasma-8_Cluster_788_sequences=11	16	11
SDS-V3-plasma-8_Cluster_3210_sequences=11	16	11
SDS-V3-plasma-24_Cluster_3150_sequences=11	124	11
SDS-V3-plasma-24_Cluster_2322_sequences=11	124	11
SDS-V3-plasma-24_Cluster_4539_sequences=11	124	11
SDS-V3-plasma-24_Cluster_918_sequences=11	124	11
SDS-V3-plasma-24_Cluster_710_sequences=11	124	11
SDS-V3-plasma-24_Cluster_3243_sequences=11	124	11
SDS-V3-plasma-24_Cluster_2762_sequences=11	124	11
SDS-V3-plasma-24_Cluster_2143_sequences=11	124	11
SDS-V3-plasma-24_Cluster_376_sequences=11	124	11
SDS-V3-plasma-24_Cluster_1198_sequences=11	124	11
SDS-V3-plasma-24_Cluster_6934_sequences=11	124	11
SDS-V3-plasma-24_Cluster_1114_sequences=11	124	11
SDS-V3-plasma-24_Cluster_1534_sequences=11	124	11
SDS-V3-plasma-24_Cluster_1123_sequences=11	124	11
SDS-V3-plasma-24_Cluster_1156_sequences=11	124	11
SDS-V3-plasma-24_Cluster_93_sequences=11	124	11
SDS-V3-plasma-24_Cluster_1717_sequences=11	124	11
SDS-V3-plasma-24_Cluster_7728_sequences=11	124	11
SDS-V3-plasma-24_Cluster_3627_sequences=11	124	11
SDS-V3-plasma-24_Cluster_1744_sequences=11	124	11
SDS-V3-plasma-27_Cluster_1003_sequences=11	131	11
SDS-V3-plasma-27_Cluster_667_sequences=11	131	11
SDS-V3-plasma-27_Cluster_2194_sequences=11	131	11
SDS-V3-plasma-27_Cluster_582_sequences=11	131	11
SDS-V3-plasma-27_Cluster_479_sequences=11	131	11
SDS-V3-plasma-27_Cluster_2190_sequences=11	131	11

SDS-V3-plasma-27_Cluster_4061_sequences=11	131	11
SDS-V3-plasma-27_Cluster_2339_sequences=11	131	11
SDS-V3-plasma-27_Cluster_501_sequences=11	131	11
SDS-V3-plasma-27_Cluster_775_sequences=11	131	11
SDS-V3-plasma-27_Cluster_616_sequences=11	131	11
SDS-V3-plasma-27_Cluster_2950_sequences=11	131	11
SDS-V3-plasma-27_Cluster_477_sequences=11	131	11
SDS-V3-plasma-27_Cluster_2177_sequences=11	131	11
SDS-V3-plasma-27_Cluster_3868_sequences=11	131	11
SDS-V3-plasma-27_Cluster_1303_sequences=11	131	11
SDS-V3-plasma-27_Cluster_423_sequences=11	131	11
SDS-V3-plasma-27_Cluster_1621_sequences=11	131	11
SDS-V3-plasma-27_Cluster_2582_sequences=11	131	11
SDS-V3-plasma-27_Cluster_1619_sequences=11	131	11
SDS-V3-plasma-45_Cluster_8230_sequences=11	282	11
SDS-V3-plasma-45_Cluster_8440_sequences=11	282	11
SDS-V3-plasma-45_Cluster_8167_sequences=11	282	11
SDS-V3-plasma-45_Cluster_11916_sequences=11	282	11
SDS-V3-plasma-45_Cluster_14091_sequences=11	282	11
SDS-V3-plasma-45_Cluster_2149_sequences=11	282	11
SDS-V3-plasma-45_Cluster_16351_sequences=11	282	11
SDS-V3-plasma-45_Cluster_20780_sequences=11	282	11
SDS-V3-plasma-45_Cluster_8815_sequences=11	282	11
SDS-V3-plasma-45_Cluster_47493_sequences=11	282	11
SDS-V3-plasma-45_Cluster_3189_sequences=11	282	11
SDS-V3-plasma-45_Cluster_25104_sequences=11	282	11
SDS-V3-plasma-45_Cluster_4360_sequences=11	282	11
SDS-V3-plasma-45_Cluster_2151_sequences=11	282	11
SDS-V3-plasma-45_Cluster_2543_sequences=11	282	11
SDS-V3-plasma-45_Cluster_7974_sequences=11	282	11
SDS-V3-plasma-45_Cluster_16915_sequences=11	282	11
SDS-V3-plasma-45_Cluster_1058_sequences=11	282	11
SDS-V3-plasma-45_Cluster_4691_sequences=11	282	11
SDS-V3-plasma-45_Cluster_14339_sequences=11	282	11
SDS-V3-plasma-45_Cluster_16723_sequences=11	282	11
SDS-V3-plasma-45_Cluster_7420_sequences=11	282	11
SDS-V3-plasma-45_Cluster_7383_sequences=11	282	11
SDS-V3-plasma-45_Cluster_26300_sequences=11	282	11
SDS-V3-plasma-45_Cluster_15100_sequences=11	282	11
SDS-V3-plasma-45_Cluster_44113_sequences=11	282	11
SDS-V3-plasma-45_Cluster_9090_sequences=11	282	11
SDS-V3-plasma-45_Cluster_14785_sequences=11	282	11
SDS-V3-plasma-45_Cluster_3416_sequences=11	282	11
SDS-V3-plasma-45_Cluster_11011_sequences=11	282	11
SDS-V3-plasma-45_Cluster_11679_sequences=11	282	11

SDS-V3-plasma-45_Cluster_13951_sequences=11	282	11
SDS-V3-plasma-45_Cluster_19155_sequences=11	282	11
SDS-V3-plasma-45_Cluster_20699_sequences=11	282	11
SDS-V3-plasma-45_Cluster_20743_sequences=11	282	11
SDS-V3-plasma-45_Cluster_3458_sequences=11	282	11
SDS-V3-plasma-45_Cluster_37080_sequences=11	282	11
SDS-V3-plasma-45_Cluster_4722_sequences=11	282	11
SDS-V3-plasma-45_Cluster_9076_sequences=11	282	11
SDS-V3-plasma-45_Cluster_13140_sequences=11	282	11
SDS-V3-plasma-45_Cluster_2625_sequences=11	282	11
SDS-V3-plasma-45_Cluster_11499_sequences=11	282	11
SDS-V3-plasma-45_Cluster_4444_sequences=11	282	11
SDS-V3-plasma-45_Cluster_10744_sequences=11	282	11
SDS-V3-plasma-45_Cluster_127_sequences=11	282	11
SDS-V3-plasma-45_Cluster_13988_sequences=11	282	11
SDS-V3-plasma-45_Cluster_36405_sequences=11	282	11
SDS-V3-plasma-45_Cluster_3680_sequences=11	282	11
SDS-V3-plasma-45_Cluster_39010_sequences=11	282	11
SDS-V3-plasma-45_Cluster_465_sequences=11	282	11
SDS-V3-plasma-45_Cluster_54_sequences=11	282	11
SDS-V3-plasma-45_Cluster_7503_sequences=11	282	11
SDS-V3-plasma-45_Cluster_7645_sequences=11	282	11
SDS-V3-plasma-45_Cluster_7966_sequences=11	282	11
SDS-V3-plasma-45_Cluster_25499_sequences=11	282	11
SDS-V3-plasma-45_Cluster_12585_sequences=11	282	11
SDS-V3-plasma-45_Cluster_7581_sequences=11	282	11
SDS-V3-plasma-45_Cluster_4553_sequences=11	282	11
SDS-V3-plasma-45_Cluster_3087_sequences=11	282	11
SDS-V3-plasma-45_Cluster_1935_sequences=11	282	11
SDS-V3-plasma-45_Cluster_18734_sequences=11	282	11
SDS-V3-plasma-45_Cluster_28773_sequences=11	282	11
SDS-V3-plasma-45_Cluster_53318_sequences=11	282	11
SDS-V3-plasma-45_Cluster_11769_sequences=11	282	11
SDS-V3-plasma-45_Cluster_16175_sequences=11	282	11
SDS-V3-plasma-45_Cluster_21981_sequences=11	282	11
SDS-V3-plasma-45_Cluster_12182_sequences=11	282	11
SDS-V3-plasma-45_Cluster_10913_sequences=11	282	11
SDS-V3-plasma-45_Cluster_18692_sequences=11	282	11
SDS-V3-plasma-45_Cluster_22089_sequences=11	282	11
SDS-V3-plasma-45_Cluster_23933_sequences=11	282	11
SDS-V3-plasma-45_Cluster_6056_sequences=11	282	11
SDS-V3-plasma-45_Cluster_3402_sequences=11	282	11
SDS-V3-plasma-45_Cluster_35300_sequences=11	282	11
SDS-V3-plasma-45_Cluster_8378_sequences=11	282	11
SDS-V3-plasma-45_Cluster_4404_sequences=11	282	11

SDS-V3-plasma-45_Cluster_44463_sequences=11	282	11
SDS-V3-plasma-45_Cluster_497_sequences=11	282	11
SDS-V3-plasma-45_Cluster_15076_sequences=11	282	11
SDS-V3-plasma-45_Cluster_8709_sequences=11	282	11
SDS-V3-plasma-45_Cluster_5630_sequences=11	282	11
SDS-V3-plasma-45_Cluster_7702_sequences=11	282	11
SDS-V3-plasma-45_Cluster_24427_sequences=11	282	11
SDS-V3-plasma-45_Cluster_8117_sequences=11	282	11
SDS-V3-plasma-45_Cluster_36817_sequences=11	282	11
SDS-V3-plasma-45_Cluster_9208_sequences=11	282	11
SDS-V3-plasma-45_Cluster_7731_sequences=11	282	11
SDS-V3-plasma-45_Cluster_2342_sequences=11	282	11
SDS-V3-plasma-45_Cluster_3632_sequences=11	282	11
SDS-V3-plasma-45_Cluster_9797_sequences=11	282	11
SDS-V3-plasma-45_Cluster_6907_sequences=11	282	11
SDS-V3-plasma-45_Cluster_21079_sequences=11	282	11
SDS-V3-plasma-45_Cluster_31813_sequences=11	282	11
SDS-V3-plasma-45_Cluster_13188_sequences=11	282	11
SDS-V3-plasma-45_Cluster_10513_sequences=11	282	11
SDS-V3-plasma-45_Cluster_7681_sequences=11	282	11
SDS-V3-plasma-45_Cluster_22190_sequences=11	282	11
SDS-V3-plasma-45_Cluster_4572_sequences=11	282	11
SDS-V3-plasma-45_Cluster_36337_sequences=11	282	11
SDS-V3-plasma-45_Cluster_20562_sequences=11	282	11
SDS-V3-plasma-45_Cluster_12499_sequences=11	282	11
SDS-V3-plasma-45_Cluster_1064_sequences=11	282	11
SDS-V3-plasma-45_Cluster_12393_sequences=11	282	11
SDS-V3-plasma-45_Cluster_1869_sequences=11	282	11
SDS-V3-plasma-45_Cluster_28868_sequences=11	282	11
SDS-V3-plasma-45_Cluster_12304_sequences=11	282	11
SDS-V3-plasma-45_Cluster_22796_sequences=11	282	11
SDS-V3-plasma-45_Cluster_20142_sequences=11	282	11
SDS-V3-plasma-45_Cluster_47245_sequences=11	282	11
SDS-V3-plasma-45_Cluster_1096_sequences=11	282	11
SDS-V3-plasma-45_Cluster_4933_sequences=11	282	11
SDS-V3-plasma-45_Cluster_23334_sequences=11	282	11
SDS-V3-plasma-45_Cluster_24055_sequences=11	282	11
SDS-V3-plasma-45_Cluster_15462_sequences=11	282	11
SDS-V3-plasma-45_Cluster_5159_sequences=11	282	11
SDS-V3-plasma-45_Cluster_1626_sequences=11	282	11
SDS-V3-plasma-45_Cluster_40396_sequences=11	282	11
SDS-V3-plasma-45_Cluster_14583_sequences=11	282	11
SDS-V3-plasma-45_Cluster_23891_sequences=11	282	11
SDS-V3-plasma-45_Cluster_8346_sequences=11	282	11
SDS-V3-plasma-45_Cluster_9595_sequences=11	282	11

SDS-V3-plasma-45_Cluster_3180_sequences=11	282	11
SDS-V3-plasma-45_Cluster_26191_sequences=11	282	11
SDS-V3-plasma-45_Cluster_11363_sequences=11	282	11
SDS-V3-plasma-45_Cluster_19094_sequences=11	282	11
SDS-V3-plasma-45_Cluster_16248_sequences=11	282	11
SDS-V3-plasma-45_Cluster_18431_sequences=11	282	11
SDS-V3-plasma-45_Cluster_4191_sequences=11	282	11
SDS-V3-plasma-45_Cluster_17994_sequences=11	282	11
SDS-V3-plasma-45_Cluster_66674_sequences=11	282	11
SDS-V3-plasma-45_Cluster_18791_sequences=11	282	11
SDS-V3-plasma-45_Cluster_21474_sequences=11	282	11
SDS-V3-plasma-45_Cluster_15403_sequences=11	282	11
SDS-V3-plasma-45_Cluster_6647_sequences=11	282	11
SDS-V3-plasma-45_Cluster_5487_sequences=11	282	11
SDS-V3-plasma-45_Cluster_2873_sequences=11	282	11
SDS-V3-plasma-45_Cluster_17651_sequences=11	282	11
SDS-V3-plasma-45_Cluster_20844_sequences=11	282	11
SDS-V3-plasma-45_Cluster_15359_sequences=11	282	11
SDS-V3-plasma-45_Cluster_13009_sequences=11	282	11
SDS-V3-plasma-45_Cluster_37656_sequences=11	282	11
SDS-V3-plasma-45_Cluster_27239_sequences=11	282	11
SDS-V3-plasma-45_Cluster_14364_sequences=11	282	11
SDS-V3-plasma-45_Cluster_4419_sequences=11	282	11
SDS-V3-plasma-45_Cluster_3735_sequences=11	282	11
SDS-V3-plasma-45_Cluster_10061_sequences=11	282	11
SDS-V3-plasma-45_Cluster_9683_sequences=11	282	11
SDS-V3-plasma-45_Cluster_17793_sequences=11	282	11
SDS-V3-plasma-45_Cluster_8152_sequences=11	282	11
SDS-V3-plasma-45_Cluster_49694_sequences=11	282	11
SDS-V3-plasma-45_Cluster_20630_sequences=11	282	11
SDS-V3-plasma-45_Cluster_19395_sequences=11	282	11
SDS-V3-plasma-45_Cluster_16736_sequences=11	282	11
SDS-V3-plasma-45_Cluster_24008_sequences=11	282	11
SDS-V3-plasma-45_Cluster_33188_sequences=11	282	11
SDS-V3-plasma-45_Cluster_10236_sequences=11	282	11
SDS-V3-plasma-45_Cluster_19029_sequences=11	282	11
SDS-V3-plasma-45_Cluster_21771_sequences=11	282	11
SDS-V3-plasma-45_Cluster_10273_sequences=11	282	11
SDS-V3-plasma-45_Cluster_7139_sequences=11	282	11
SDS-V3-plasma-45_Cluster_17091_sequences=11	282	11
SDS-V3-plasma-45_Cluster_2681_sequences=11	282	11
SDS-V3-plasma-45_Cluster_22628_sequences=11	282	11
SDS-V3-plasma-45_Cluster_7811_sequences=11	282	11
SDS-V3-plasma-45_Cluster_3295_sequences=11	282	11
SDS-V3-plasma-45_Cluster_8571_sequences=11	282	11

SDS-V3-plasma-45_Cluster_37017_sequences=11	282	11
SDS-V3-plasma-45_Cluster_12123_sequences=11	282	11
SDS-V3-plasma-45_Cluster_14849_sequences=11	282	11
SDS-V3-plasma-45_Cluster_18957_sequences=11	282	11
SDS-V3-plasma-45_Cluster_14723_sequences=11	282	11
SDS-V3-plasma-45_Cluster_12105_sequences=11	282	11
SDS-V3-plasma-45_Cluster_7290_sequences=11	282	11
SDS-V3-plasma-45_Cluster_31406_sequences=11	282	11
SDS-V3-plasma-45_Cluster_2412_sequences=11	282	11
SDS-V3-plasma-45_Cluster_12073_sequences=11	282	11
SDS-V3-plasma-45_Cluster_13261_sequences=11	282	11
SDS-V3-plasma-45_Cluster_8811_sequences=11	282	11
SDS-V3-plasma-45_Cluster_2759_sequences=11	282	11
SDS-V3-plasma-45_Cluster_1744_sequences=11	282	11
SDS-V3-plasma-45_Cluster_4195_sequences=11	282	11
SDS-V3-plasma-45_Cluster_17591_sequences=11	282	11
SDS-V3-plasma-45_Cluster_10267_sequences=11	282	11
SDS-V3-plasma-45_Cluster_14386_sequences=11	282	11
SDS-V3-plasma-45_Cluster_1525_sequences=11	282	11
SDS-V3-plasma-45_Cluster_18391_sequences=11	282	11
SDS-V3-plasma-45_Cluster_19351_sequences=11	282	11
SDS-V3-plasma-45_Cluster_20671_sequences=11	282	11
SDS-V3-plasma-45_Cluster_36912_sequences=11	282	11
SDS-V3-plasma-45_Cluster_40051_sequences=11	282	11
SDS-V3-plasma-45_Cluster_4632_sequences=11	282	11
SDS-V3-plasma-45_Cluster_114_sequences=11	282	11
SDS-V3-plasma-45_Cluster_2313_sequences=11	282	11
SDS-V3-plasma-45_Cluster_9739_sequences=11	282	11
SDS-V3-plasma-45_Cluster_19612_sequences=11	282	11
SDS-V3-plasma-45_Cluster_60577_sequences=11	282	11
SDS-V3-plasma-45_Cluster_13553_sequences=11	282	11
SDS-V3-plasma-45_Cluster_9828_sequences=11	282	11
SDS-V3-plasma-45_Cluster_12635_sequences=11	282	11
SDS-V3-plasma-45_Cluster_18412_sequences=11	282	11
SDS-V3-plasma-45_Cluster_37026_sequences=11	282	11
SDS-V3-plasma-45_Cluster_4032_sequences=11	282	11
SDS-V3-plasma-45_Cluster_8433_sequences=11	282	11
SDS-V3-plasma-45_Cluster_927_sequences=11	282	11
SDS-V3-plasma-45_Cluster_9426_sequences=11	282	11
SDS-V3-plasma-45_Cluster_5667_sequences=11	282	11
SDS-V3-plasma-45_Cluster_9338_sequences=11	282	11
SDS-V3-plasma-45_Cluster_32681_sequences=11	282	11
SDS-V3-plasma-45_Cluster_18076_sequences=11	282	11
SDS-V3-plasma-45_Cluster_20117_sequences=11	282	11
SDS-V3-plasma-45_Cluster_6271_sequences=11	282	11

SDS-V3-plasma-45_Cluster_17804_sequences=11	282	11
SDS-V3-plasma-45_Cluster_22598_sequences=11	282	11
SDS-V3-plasma-45_Cluster_4744_sequences=11	282	11
SDS-V3-plasma-45_Cluster_8636_sequences=11	282	11
SDS-V3-plasma-45_Cluster_9567_sequences=11	282	11
SDS-V3-plasma-45_Cluster_24223_sequences=11	282	11
SDS-V3-plasma-45_Cluster_8401_sequences=11	282	11
SDS-V3-plasma-45_Cluster_16112_sequences=11	282	11
SDS-V3-plasma-45_Cluster_8781_sequences=11	282	11
SDS-V3-plasma-45_Cluster_16883_sequences=11	282	11
SDS-V3-plasma-45_Cluster_4372_sequences=11	282	11
SDS-V3-plasma-45_Cluster_7179_sequences=11	282	11
SDS-V3-plasma-45_Cluster_1406_sequences=11	282	11
SDS-V3-plasma-45_Cluster_14734_sequences=11	282	11
SDS-V3-plasma-45_Cluster_3460_sequences=11	282	11
SDS-V3-plasma-45_Cluster_6501_sequences=11	282	11
SDS-V3-plasma-45_Cluster_6098_sequences=11	282	11
SDS-V3-plasma-45_Cluster_35491_sequences=11	282	11
SDS-V3-plasma-45_Cluster_16137_sequences=11	282	11
SDS-V3-plasma-46_Cluster_1005_sequences=11	286	11
SDS-V3-plasma-46_Cluster_6648_sequences=11	286	11
SDS-V3-plasma-46_Cluster_5387_sequences=11	286	11
SDS-V3-plasma-46_Cluster_1947_sequences=11	286	11
SDS-V3-plasma-46_Cluster_13099_sequences=11	286	11
SDS-V3-plasma-46_Cluster_2909_sequences=11	286	11
SDS-V3-plasma-46_Cluster_828_sequences=11	286	11
SDS-V3-plasma-46_Cluster_5202_sequences=11	286	11
SDS-V3-plasma-46_Cluster_5292_sequences=11	286	11
SDS-V3-plasma-46_Cluster_815_sequences=11	286	11
SDS-V3-plasma-46_Cluster_15883_sequences=11	286	11
SDS-V3-plasma-46_Cluster_1816_sequences=11	286	11
SDS-V3-plasma-46_Cluster_464_sequences=11	286	11
SDS-V3-plasma-46_Cluster_2360_sequences=11	286	11
SDS-V3-plasma-46_Cluster_6046_sequences=11	286	11
SDS-V3-plasma-46_Cluster_1916_sequences=11	286	11
SDS-V3-plasma-46_Cluster_1188_sequences=11	286	11
SDS-V3-plasma-46_Cluster_2746_sequences=11	286	11
SDS-V3-plasma-46_Cluster_3497_sequences=11	286	11
SDS-V3-plasma-46_Cluster_4233_sequences=11	286	11
SDS-V3-plasma-46_Cluster_4207_sequences=11	286	11
SDS-V3-plasma-46_Cluster_3026_sequences=11	286	11
SDS-V3-plasma-46_Cluster_443_sequences=11	286	11
SDS-V3-plasma-46_Cluster_5264_sequences=11	286	11
SDS-V3-plasma-46_Cluster_6924_sequences=11	286	11
SDS-V3-plasma-46_Cluster_8144_sequences=11	286	11

SDS-V3-plasma-46_Cluster_4711_sequences=11	286	11
SDS-V3-plasma-46_Cluster_1106_sequences=11	286	11
SDS-V3-plasma-46_Cluster_11903_sequences=11	286	11
SDS-V3-plasma-46_Cluster_3313_sequences=11	286	11
SDS-V3-plasma-46_Cluster_5058_sequences=11	286	11
SDS-V3-plasma-46_Cluster_5479_sequences=11	286	11
SDS-V3-plasma-46_Cluster_572_sequences=11	286	11
SDS-V3-plasma-46_Cluster_7667_sequences=11	286	11
SDS-V3-plasma-46_Cluster_6600_sequences=11	286	11
SDS-V3-plasma-46_Cluster_11580_sequences=11	286	11
SDS-V3-plasma-46_Cluster_13552_sequences=11	286	11
SDS-V3-plasma-46_Cluster_737_sequences=11	286	11
SDS-V3-plasma-46_Cluster_10474_sequences=11	286	11
SDS-V3-plasma-46_Cluster_1304_sequences=11	286	11
SDS-V3-plasma-46_Cluster_1309_sequences=11	286	11
SDS-V3-plasma-46_Cluster_139_sequences=11	286	11
SDS-V3-plasma-46_Cluster_1407_sequences=11	286	11
SDS-V3-plasma-46_Cluster_14704_sequences=11	286	11
SDS-V3-plasma-46_Cluster_1493_sequences=11	286	11
SDS-V3-plasma-46_Cluster_1515_sequences=11	286	11
SDS-V3-plasma-46_Cluster_16833_sequences=11	286	11
SDS-V3-plasma-46_Cluster_364_sequences=11	286	11
SDS-V3-plasma-46_Cluster_3838_sequences=11	286	11
SDS-V3-plasma-46_Cluster_3972_sequences=11	286	11
SDS-V3-plasma-46_Cluster_4151_sequences=11	286	11
SDS-V3-plasma-46_Cluster_4810_sequences=11	286	11
SDS-V3-plasma-46_Cluster_5087_sequences=11	286	11
SDS-V3-plasma-46_Cluster_5317_sequences=11	286	11
SDS-V3-plasma-46_Cluster_5365_sequences=11	286	11
SDS-V3-plasma-46_Cluster_6684_sequences=11	286	11
SDS-V3-plasma-46_Cluster_7025_sequences=11	286	11
SDS-V3-plasma-46_Cluster_775_sequences=11	286	11
SDS-V3-plasma-46_Cluster_8866_sequences=11	286	11
SDS-V3-plasma-46_Cluster_9114_sequences=11	286	11
SDS-V3-plasma-46_Cluster_1317_sequences=11	286	11
SDS-V3-plasma-46_Cluster_1576_sequences=11	286	11
SDS-V3-plasma-46_Cluster_19927_sequences=11	286	11
SDS-V3-plasma-46_Cluster_2782_sequences=11	286	11
SDS-V3-plasma-46_Cluster_5743_sequences=11	286	11
SDS-V3-plasma-46_Cluster_7309_sequences=11	286	11
SDS-V3-plasma-46_Cluster_796_sequences=11	286	11
SDS-V3-plasma-46_Cluster_9501_sequences=11	286	11
SDS-V3-plasma-46_Cluster_19844_sequences=11	286	11
SDS-V3-plasma-46_Cluster_984_sequences=11	286	11
SDS-V3-plasma-46_Cluster_5223_sequences=11	286	11

SDS-V3-plasma-46_Cluster_565_sequences=11	286	11
SDS-V3-plasma-46_Cluster_12366_sequences=11	286	11
SDS-V3-plasma-46_Cluster_1420_sequences=11	286	11
SDS-V3-plasma-46_Cluster_768_sequences=11	286	11
SDS-V3-plasma-46_Cluster_3267_sequences=11	286	11
SDS-V3-plasma-46_Cluster_20258_sequences=11	286	11
SDS-V3-plasma-46_Cluster_10703_sequences=11	286	11
SDS-V3-plasma-46_Cluster_1050_sequences=11	286	11
SDS-V3-plasma-46_Cluster_3565_sequences=11	286	11
SDS-V3-plasma-46_Cluster_570_sequences=11	286	11
SDS-V3-plasma-46_Cluster_2533_sequences=11	286	11
SDS-V3-plasma-46_Cluster_548_sequences=11	286	11
SDS-V3-plasma-46_Cluster_3204_sequences=11	286	11
SDS-V3-plasma-46_Cluster_2402_sequences=11	286	11
SDS-V3-plasma-46_Cluster_2621_sequences=11	286	11
SDS-V3-plasma-46_Cluster_9552_sequences=11	286	11
SDS-V3-plasma-46_Cluster_4275_sequences=11	286	11
SDS-V3-plasma-46_Cluster_7322_sequences=11	286	11
SDS-V3-plasma-46_Cluster_10073_sequences=11	286	11
SDS-V3-plasma-46_Cluster_12544_sequences=11	286	11
SDS-V3-plasma-46_Cluster_3240_sequences=11	286	11
SDS-V3-plasma-46_Cluster_6292_sequences=11	286	11
SDS-V3-plasma-46_Cluster_6645_sequences=11	286	11
SDS-V3-plasma-67_Cluster_5951_sequences=11	504	11
SDS-V3-plasma-67_Cluster_37898_sequences=11	504	11
SDS-V3-plasma-67_Cluster_46373_sequences=11	504	11
SDS-V3-plasma-67_Cluster_51795_sequences=11	504	11
SDS-V3-plasma-67_Cluster_45118_sequences=11	504	11
SDS-V3-plasma-67_Cluster_3433_sequences=11	504	11
SDS-V3-plasma-67_Cluster_6077_sequences=11	504	11
SDS-V3-plasma-67_Cluster_1334_sequences=11	504	11
SDS-V3-plasma-67_Cluster_5250_sequences=11	504	11
SDS-V3-plasma-67_Cluster_14487_sequences=11	504	11
SDS-V3-plasma-67_Cluster_9703_sequences=11	504	11
SDS-V3-plasma-67_Cluster_34956_sequences=11	504	11
SDS-V3-plasma-67_Cluster_7399_sequences=11	504	11
SDS-V3-plasma-67_Cluster_12190_sequences=11	504	11
SDS-V3-plasma-67_Cluster_15772_sequences=11	504	11
SDS-V3-plasma-67_Cluster_1669_sequences=11	504	11
SDS-V3-plasma-67_Cluster_18697_sequences=11	504	11
SDS-V3-plasma-67_Cluster_24813_sequences=11	504	11
SDS-V3-plasma-67_Cluster_261_sequences=11	504	11
SDS-V3-plasma-67_Cluster_14615_sequences=11	504	11
SDS-V3-plasma-67_Cluster_7069_sequences=11	504	11
SDS-V3-plasma-67_Cluster_16174_sequences=11	504	11

SDS-V3-plasma-67_Cluster_3558_sequences=11	504	11
SDS-V3-plasma-67_Cluster_2893_sequences=11	504	11
SDS-V3-plasma-67_Cluster_4653_sequences=11	504	11
SDS-V3-plasma-67_Cluster_15610_sequences=11	504	11
SDS-V3-plasma-67_Cluster_2197_sequences=11	504	11
SDS-V3-plasma-67_Cluster_29171_sequences=11	504	11
SDS-V3-plasma-67_Cluster_36792_sequences=11	504	11
SDS-V3-plasma-67_Cluster_2324_sequences=11	504	11
SDS-V3-plasma-67_Cluster_8944_sequences=11	504	11
SDS-V3-plasma-67_Cluster_12174_sequences=11	504	11
SDS-V3-plasma-67_Cluster_17659_sequences=11	504	11
SDS-V3-plasma-67_Cluster_18790_sequences=11	504	11
SDS-V3-plasma-67_Cluster_2973_sequences=11	504	11
SDS-V3-plasma-67_Cluster_18113_sequences=11	504	11
SDS-V3-plasma-67_Cluster_28907_sequences=11	504	11
SDS-V3-plasma-67_Cluster_14241_sequences=11	504	11
SDS-V3-plasma-67_Cluster_19818_sequences=11	504	11
SDS-V3-plasma-67_Cluster_17563_sequences=11	504	11
SDS-V3-plasma-67_Cluster_10062_sequences=11	504	11
SDS-V3-plasma-67_Cluster_16953_sequences=11	504	11
SDS-V3-plasma-67_Cluster_1772_sequences=11	504	11
SDS-V3-plasma-67_Cluster_18842_sequences=11	504	11
SDS-V3-plasma-67_Cluster_21407_sequences=11	504	11
SDS-V3-plasma-67_Cluster_22494_sequences=11	504	11
SDS-V3-plasma-67_Cluster_42079_sequences=11	504	11
SDS-V3-plasma-67_Cluster_4541_sequences=11	504	11
SDS-V3-plasma-67_Cluster_7978_sequences=11	504	11
SDS-V3-plasma-67_Cluster_8692_sequences=11	504	11
SDS-V3-plasma-67_Cluster_10986_sequences=11	504	11
SDS-V3-plasma-67_Cluster_4309_sequences=11	504	11
SDS-V3-plasma-67_Cluster_37279_sequences=11	504	11
SDS-V3-plasma-67_Cluster_2019_sequences=11	504	11
SDS-V3-plasma-67_Cluster_9337_sequences=11	504	11
SDS-V3-plasma-67_Cluster_4934_sequences=11	504	11
SDS-V3-plasma-67_Cluster_9277_sequences=11	504	11
SDS-V3-plasma-67_Cluster_4798_sequences=11	504	11
SDS-V3-plasma-67_Cluster_4735_sequences=11	504	11
SDS-V3-plasma-67_Cluster_22341_sequences=11	504	11
SDS-V3-plasma-67_Cluster_9439_sequences=11	504	11
SDS-V3-plasma-67_Cluster_3866_sequences=11	504	11
SDS-V3-plasma-67_Cluster_31757_sequences=11	504	11
SDS-V3-plasma-67_Cluster_551_sequences=11	504	11
SDS-V3-plasma-67_Cluster_7161_sequences=11	504	11
SDS-V3-plasma-67_Cluster_11122_sequences=11	504	11
SDS-V3-plasma-67_Cluster_27694_sequences=11	504	11

SDS-V3-plasma-67_Cluster_18700_sequences=11	504	11
SDS-V3-plasma-67_Cluster_13845_sequences=11	504	11
SDS-V3-plasma-67_Cluster_6976_sequences=11	504	11
SDS-V3-plasma-67_Cluster_31612_sequences=11	504	11
SDS-V3-plasma-67_Cluster_5220_sequences=11	504	11
SDS-V3-plasma-67_Cluster_17979_sequences=11	504	11
SDS-V3-plasma-67_Cluster_4488_sequences=11	504	11
SDS-V3-plasma-67_Cluster_10829_sequences=11	504	11
SDS-V3-plasma-67_Cluster_17678_sequences=11	504	11
SDS-V3-plasma-67_Cluster_17173_sequences=11	504	11
SDS-V3-plasma-67_Cluster_11364_sequences=11	504	11
SDS-V3-plasma-67_Cluster_6798_sequences=11	504	11
SDS-V3-plasma-67_Cluster_25117_sequences=11	504	11
SDS-V3-plasma-67_Cluster_249_sequences=11	504	11
SDS-V3-plasma-67_Cluster_3707_sequences=11	504	11
SDS-V3-plasma-67_Cluster_1735_sequences=11	504	11
SDS-V3-plasma-67_Cluster_39500_sequences=11	504	11
SDS-V3-plasma-67_Cluster_749_sequences=11	504	11
SDS-V3-plasma-67_Cluster_20597_sequences=11	504	11
SDS-V3-plasma-67_Cluster_17406_sequences=11	504	11
SDS-V3-plasma-67_Cluster_25633_sequences=11	504	11
SDS-V3-plasma-67_Cluster_4159_sequences=11	504	11
SDS-V3-plasma-67_Cluster_7514_sequences=11	504	11
SDS-V3-plasma-67_Cluster_10699_sequences=11	504	11
SDS-V3-plasma-67_Cluster_11170_sequences=11	504	11
SDS-V3-plasma-67_Cluster_11818_sequences=11	504	11
SDS-V3-plasma-67_Cluster_15543_sequences=11	504	11
SDS-V3-plasma-67_Cluster_21799_sequences=11	504	11
SDS-V3-plasma-67_Cluster_3648_sequences=11	504	11
SDS-V3-plasma-67_Cluster_41532_sequences=11	504	11
SDS-V3-plasma-67_Cluster_20030_sequences=11	504	11
SDS-V3-plasma-67_Cluster_29613_sequences=11	504	11
SDS-V3-plasma-67_Cluster_30860_sequences=11	504	11
SDS-V3-plasma-67_Cluster_11989_sequences=11	504	11
SDS-V3-plasma-67_Cluster_1487_sequences=11	504	11
SDS-V3-plasma-67_Cluster_16729_sequences=11	504	11
SDS-V3-plasma-67_Cluster_26807_sequences=11	504	11
SDS-V3-plasma-67_Cluster_2743_sequences=11	504	11
SDS-V3-plasma-67_Cluster_3056_sequences=11	504	11
SDS-V3-plasma-67_Cluster_4865_sequences=11	504	11
SDS-V3-plasma-67_Cluster_32698_sequences=11	504	11
SDS-V3-plasma-67_Cluster_9521_sequences=11	504	11
SDS-V3-plasma-67_Cluster_14239_sequences=11	504	11
SDS-V3-plasma-67_Cluster_8003_sequences=11	504	11
SDS-V3-plasma-67_Cluster_37707_sequences=11	504	11

SDS-V3-plasma-67_Cluster_1852_sequences=11	504	11
SDS-V3-plasma-67_Cluster_6349_sequences=11	504	11
SDS-V3-plasma-67_Cluster_25452_sequences=11	504	11
SDS-V3-plasma-67_Cluster_26817_sequences=11	504	11
SDS-V3-plasma-67_Cluster_11841_sequences=11	504	11
SDS-V3-plasma-67_Cluster_3304_sequences=11	504	11
SDS-V3-plasma-67_Cluster_11411_sequences=11	504	11
SDS-V3-plasma-67_Cluster_30034_sequences=11	504	11
SDS-V3-plasma-67_Cluster_8839_sequences=11	504	11
SDS-V3-plasma-67_Cluster_18623_sequences=11	504	11
SDS-V3-plasma-67_Cluster_32009_sequences=11	504	11
SDS-V3-plasma-67_Cluster_12871_sequences=11	504	11
SDS-V3-plasma-67_Cluster_8653_sequences=11	504	11
SDS-V3-plasma-67_Cluster_7763_sequences=11	504	11
SDS-V3-plasma-67_Cluster_25537_sequences=11	504	11
SDS-V3-plasma-67_Cluster_8220_sequences=11	504	11
SDS-V3-plasma-67_Cluster_10430_sequences=11	504	11
SDS-V3-plasma-67_Cluster_2438_sequences=11	504	11
SDS-V3-plasma-67_Cluster_12723_sequences=11	504	11
SDS-V3-plasma-67_Cluster_16643_sequences=11	504	11
SDS-V3-plasma-67_Cluster_17310_sequences=11	504	11
SDS-V3-plasma-67_Cluster_44418_sequences=11	504	11
SDS-V3-plasma-67_Cluster_57998_sequences=11	504	11
SDS-V3-plasma-67_Cluster_6866_sequences=11	504	11
SDS-V3-plasma-67_Cluster_4435_sequences=11	504	11
SDS-V3-plasma-67_Cluster_25213_sequences=11	504	11
SDS-V3-plasma-67_Cluster_46293_sequences=11	504	11
SDS-V3-plasma-67_Cluster_10884_sequences=11	504	11
SDS-V3-plasma-67_Cluster_10946_sequences=11	504	11
SDS-V3-plasma-67_Cluster_33305_sequences=11	504	11
SDS-V3-plasma-67_Cluster_34555_sequences=11	504	11
SDS-V3-plasma-67_Cluster_35840_sequences=11	504	11
SDS-V3-plasma-67_Cluster_4841_sequences=11	504	11
SDS-V3-plasma-67_Cluster_651_sequences=11	504	11
SDS-V3-plasma-67_Cluster_9432_sequences=11	504	11
SDS-V3-plasma-67_Cluster_13564_sequences=11	504	11
SDS-V3-plasma-67_Cluster_23829_sequences=11	504	11
SDS-V3-plasma-67_Cluster_7236_sequences=11	504	11
SDS-V3-plasma-67_Cluster_15418_sequences=11	504	11
SDS-V3-plasma-67_Cluster_289_sequences=11	504	11
SDS-V3-plasma-67_Cluster_16692_sequences=11	504	11
SDS-V3-plasma-67_Cluster_4387_sequences=11	504	11
SDS-V3-plasma-67_Cluster_45955_sequences=11	504	11
SDS-V3-plasma-67_Cluster_6281_sequences=11	504	11
SDS-V3-plasma-67_Cluster_74569_sequences=11	504	11

SDS-V3-plasma-67_Cluster_9880_sequences=11	504	11
SDS-V3-plasma-67_Cluster_18285_sequences=11	504	11
SDS-V3-plasma-67_Cluster_5470_sequences=11	504	11
SDS-V3-plasma-67_Cluster_12453_sequences=11	504	11
SDS-V3-plasma-67_Cluster_10377_sequences=11	504	11
SDS-V3-plasma-67_Cluster_14596_sequences=11	504	11
SDS-V3-plasma-67_Cluster_16178_sequences=11	504	11
SDS-V3-plasma-67_Cluster_40555_sequences=11	504	11
SDS-V3-plasma-67_Cluster_24192_sequences=11	504	11
SDS-V3-plasma-67_Cluster_58755_sequences=11	504	11
SDS-V3-plasma-67_Cluster_14548_sequences=11	504	11
SDS-V3-plasma-67_Cluster_6116_sequences=11	504	11
SDS-V3-plasma-67_Cluster_18622_sequences=11	504	11
SDS-V3-plasma-67_Cluster_10529_sequences=11	504	11
SDS-V3-plasma-67_Cluster_13239_sequences=11	504	11
SDS-V3-plasma-67_Cluster_19705_sequences=11	504	11
SDS-V3-plasma-67_Cluster_3521_sequences=11	504	11
SDS-V3-plasma-67_Cluster_9465_sequences=11	504	11
SDS-V3-plasma-67_Cluster_35811_sequences=11	504	11
SDS-V3-plasma-67_Cluster_11459_sequences=11	504	11
SDS-V3-plasma-67_Cluster_188_sequences=11	504	11
SDS-V3-plasma-67_Cluster_32638_sequences=11	504	11
SDS-V3-plasma-67_Cluster_772_sequences=11	504	11
SDS-V3-plasma-67_Cluster_5793_sequences=11	504	11
SDS-V3-plasma-67_Cluster_14019_sequences=11	504	11
SDS-V3-plasma-67_Cluster_8588_sequences=11	504	11
SDS-V3-plasma-46_Cluster_ACATTGAGT_sequences=10	286	10
SDS-V3-plasma-0_Cluster_3184_sequences=10	0	10
SDS-V3-plasma-0_Cluster_6243_sequences=10	0	10
SDS-V3-plasma-0_Cluster_2575_sequences=10	0	10
SDS-V3-plasma-0_Cluster_20142_sequences=10	0	10
SDS-V3-plasma-0_Cluster_4710_sequences=10	0	10
SDS-V3-plasma-0_Cluster_664_sequences=10	0	10
SDS-V3-plasma-0_Cluster_1342_sequences=10	0	10
SDS-V3-plasma-0_Cluster_1607_sequences=10	0	10
SDS-V3-plasma-0_Cluster_6575_sequences=10	0	10
SDS-V3-plasma-0_Cluster_9764_sequences=10	0	10
SDS-V3-plasma-0_Cluster_13379_sequences=10	0	10
SDS-V3-plasma-0_Cluster_1009_sequences=10	0	10
SDS-V3-plasma-0_Cluster_10738_sequences=10	0	10
SDS-V3-plasma-0_Cluster_12159_sequences=10	0	10
SDS-V3-plasma-0_Cluster_1515_sequences=10	0	10
SDS-V3-plasma-0_Cluster_2029_sequences=10	0	10
SDS-V3-plasma-0_Cluster_3755_sequences=10	0	10
SDS-V3-plasma-0_Cluster_3999_sequences=10	0	10

SDS-V3-plasma-0_Cluster_4089_sequences=10	0	10
SDS-V3-plasma-0_Cluster_4478_sequences=10	0	10
SDS-V3-plasma-0_Cluster_4836_sequences=10	0	10
SDS-V3-plasma-0_Cluster_5990_sequences=10	0	10
SDS-V3-plasma-0_Cluster_7802_sequences=10	0	10
SDS-V3-plasma-0_Cluster_16119_sequences=10	0	10
SDS-V3-plasma-0_Cluster_4297_sequences=10	0	10
SDS-V3-plasma-0_Cluster_3232_sequences=10	0	10
SDS-V3-plasma-0_Cluster_5024_sequences=10	0	10
SDS-V3-plasma-0_Cluster_3389_sequences=10	0	10
SDS-V3-plasma-0_Cluster_4600_sequences=10	0	10
SDS-V3-plasma-0_Cluster_1349_sequences=10	0	10
SDS-V3-plasma-0_Cluster_1333_sequences=10	0	10
SDS-V3-plasma-0_Cluster_3217_sequences=10	0	10
SDS-V3-plasma-0_Cluster_1542_sequences=10	0	10
SDS-V3-plasma-0_Cluster_5429_sequences=10	0	10
SDS-V3-plasma-0_Cluster_11618_sequences=10	0	10
SDS-V3-plasma-0_Cluster_5877_sequences=10	0	10
SDS-V3-plasma-0_Cluster_886_sequences=10	0	10
SDS-V3-plasma-0_Cluster_25386_sequences=10	0	10
SDS-V3-plasma-0_Cluster_10140_sequences=10	0	10
SDS-V3-plasma-0_Cluster_1976_sequences=10	0	10
SDS-V3-plasma-0_Cluster_20434_sequences=10	0	10
SDS-V3-plasma-0_Cluster_8545_sequences=10	0	10
SDS-V3-plasma-0_Cluster_987_sequences=10	0	10
SDS-V3-plasma-0_Cluster_647_sequences=10	0	10
SDS-V3-plasma-0_Cluster_3695_sequences=10	0	10
SDS-V3-plasma-0_Cluster_2457_sequences=10	0	10
SDS-V3-plasma-0_Cluster_6590_sequences=10	0	10
SDS-V3-plasma-0_Cluster_3585_sequences=10	0	10
SDS-V3-plasma-0_Cluster_1517_sequences=10	0	10
SDS-V3-plasma-0_Cluster_10333_sequences=10	0	10
SDS-V3-plasma-0_Cluster_366_sequences=10	0	10
SDS-V3-plasma-0_Cluster_1599_sequences=10	0	10
SDS-V3-plasma-0_Cluster_1583_sequences=10	0	10
SDS-V3-plasma-0_Cluster_5667_sequences=10	0	10
SDS-V3-plasma-0_Cluster_2005_sequences=10	0	10
SDS-V3-plasma-0_Cluster_4993_sequences=10	0	10
SDS-V3-plasma-0_Cluster_2148_sequences=10	0	10
SDS-V3-plasma-0_Cluster_23353_sequences=10	0	10
SDS-V3-plasma-0_Cluster_5946_sequences=10	0	10
SDS-V3-plasma-0_Cluster_1007_sequences=10	0	10
SDS-V3-plasma-0_Cluster_1561_sequences=10	0	10
SDS-V3-plasma-0_Cluster_3638_sequences=10	0	10
SDS-V3-plasma-0_Cluster_5507_sequences=10	0	10

SDS-V3-plasma-0_Cluster_6141_sequences=10	0	10
SDS-V3-plasma-0_Cluster_6702_sequences=10	0	10
SDS-V3-plasma-0_Cluster_8869_sequences=10	0	10
SDS-V3-plasma-0_Cluster_9472_sequences=10	0	10
SDS-V3-plasma-0_Cluster_14134_sequences=10	0	10
SDS-V3-plasma-0_Cluster_7185_sequences=10	0	10
SDS-V3-plasma-0_Cluster_8564_sequences=10	0	10
SDS-V3-plasma-0_Cluster_7062_sequences=10	0	10
SDS-V3-plasma-0_Cluster_4253_sequences=10	0	10
SDS-V3-plasma-0_Cluster_3156_sequences=10	0	10
SDS-V3-plasma-0_Cluster_204_sequences=10	0	10
SDS-V3-plasma-0_Cluster_2061_sequences=10	0	10
SDS-V3-plasma-0_Cluster_4030_sequences=10	0	10
SDS-V3-plasma-0_Cluster_856_sequences=10	0	10
SDS-V3-plasma-0_Cluster_1972_sequences=10	0	10
SDS-V3-plasma-0_Cluster_6825_sequences=10	0	10
SDS-V3-plasma-0_Cluster_7441_sequences=10	0	10
SDS-V3-plasma-0_Cluster_3337_sequences=10	0	10
SDS-V3-plasma-0_Cluster_7774_sequences=10	0	10
SDS-V3-plasma-0_Cluster_1294_sequences=10	0	10
SDS-V3-plasma-0_Cluster_3576_sequences=10	0	10
SDS-V3-plasma-0_Cluster_9480_sequences=10	0	10
SDS-V3-plasma-0_Cluster_170_sequences=10	0	10
SDS-V3-plasma-0_Cluster_2062_sequences=10	0	10
SDS-V3-plasma-0_Cluster_2151_sequences=10	0	10
SDS-V3-plasma-0_Cluster_9190_sequences=10	0	10
SDS-V3-plasma-0_Cluster_6486_sequences=10	0	10
SDS-V3-plasma-0_Cluster_5663_sequences=10	0	10
SDS-V3-plasma-0_Cluster_7552_sequences=10	0	10
SDS-V3-plasma-5_Cluster_1063_sequences=10	9	10
SDS-V3-plasma-5_Cluster_1087_sequences=10	9	10
SDS-V3-plasma-5_Cluster_1179_sequences=10	9	10
SDS-V3-plasma-5_Cluster_129_sequences=10	9	10
SDS-V3-plasma-5_Cluster_2085_sequences=10	9	10
SDS-V3-plasma-5_Cluster_22_sequences=10	9	10
SDS-V3-plasma-5_Cluster_296_sequences=10	9	10
SDS-V3-plasma-5_Cluster_353_sequences=10	9	10
SDS-V3-plasma-5_Cluster_3_sequences=10	9	10
SDS-V3-plasma-5_Cluster_607_sequences=10	9	10
SDS-V3-plasma-5_Cluster_850_sequences=10	9	10
SDS-V3-plasma-5_Cluster_909_sequences=10	9	10
SDS-V3-plasma-5_Cluster_917_sequences=10	9	10
SDS-V3-plasma-5_Cluster_596_sequences=10	9	10
SDS-V3-plasma-5_Cluster_134_sequences=10	9	10
SDS-V3-plasma-5_Cluster_487_sequences=10	9	10

SDS-V3-plasma-5_Cluster_115_sequences=10	9	10
SDS-V3-plasma-5_Cluster_203_sequences=10	9	10
SDS-V3-plasma-5_Cluster_1607_sequences=10	9	10
SDS-V3-plasma-5_Cluster_16_sequences=10	9	10
SDS-V3-plasma-5_Cluster_18_sequences=10	9	10
SDS-V3-plasma-5_Cluster_334_sequences=10	9	10
SDS-V3-plasma-5_Cluster_449_sequences=10	9	10
SDS-V3-plasma-5_Cluster_834_sequences=10	9	10
SDS-V3-plasma-5_Cluster_321_sequences=10	9	10
SDS-V3-plasma-7_Cluster_2194_sequences=10	14	10
SDS-V3-plasma-7_Cluster_2447_sequences=10	14	10
SDS-V3-plasma-7_Cluster_3131_sequences=10	14	10
SDS-V3-plasma-7_Cluster_3755_sequences=10	14	10
SDS-V3-plasma-7_Cluster_3874_sequences=10	14	10
SDS-V3-plasma-7_Cluster_468_sequences=10	14	10
SDS-V3-plasma-7_Cluster_788_sequences=10	14	10
SDS-V3-plasma-7_Cluster_882_sequences=10	14	10
SDS-V3-plasma-7_Cluster_369_sequences=10	14	10
SDS-V3-plasma-7_Cluster_1962_sequences=10	14	10
SDS-V3-plasma-7_Cluster_5532_sequences=10	14	10
SDS-V3-plasma-7_Cluster_5736_sequences=10	14	10
SDS-V3-plasma-7_Cluster_2337_sequences=10	14	10
SDS-V3-plasma-7_Cluster_297_sequences=10	14	10
SDS-V3-plasma-7_Cluster_5052_sequences=10	14	10
SDS-V3-plasma-7_Cluster_4020_sequences=10	14	10
SDS-V3-plasma-7_Cluster_5835_sequences=10	14	10
SDS-V3-plasma-7_Cluster_1472_sequences=10	14	10
SDS-V3-plasma-7_Cluster_1491_sequences=10	14	10
SDS-V3-plasma-7_Cluster_531_sequences=10	14	10
SDS-V3-plasma-7_Cluster_1243_sequences=10	14	10
SDS-V3-plasma-7_Cluster_353_sequences=10	14	10
SDS-V3-plasma-7_Cluster_4781_sequences=10	14	10
SDS-V3-plasma-7_Cluster_780_sequences=10	14	10
SDS-V3-plasma-7_Cluster_2559_sequences=10	14	10
SDS-V3-plasma-7_Cluster_1986_sequences=10	14	10
SDS-V3-plasma-7_Cluster_458_sequences=10	14	10
SDS-V3-plasma-7_Cluster_5213_sequences=10	14	10
SDS-V3-plasma-7_Cluster_653_sequences=10	14	10
SDS-V3-plasma-7_Cluster_1386_sequences=10	14	10
SDS-V3-plasma-7_Cluster_1575_sequences=10	14	10
SDS-V3-plasma-7_Cluster_576_sequences=10	14	10
SDS-V3-plasma-7_Cluster_7894_sequences=10	14	10
SDS-V3-plasma-7_Cluster_1244_sequences=10	14	10
SDS-V3-plasma-7_Cluster_3788_sequences=10	14	10
SDS-V3-plasma-7_Cluster_2601_sequences=10	14	10

SDS-V3-plasma-7_Cluster_2744_sequences=10	14	10
SDS-V3-plasma-7_Cluster_2311_sequences=10	14	10
SDS-V3-plasma-7_Cluster_4115_sequences=10	14	10
SDS-V3-plasma-7_Cluster_2217_sequences=10	14	10
SDS-V3-plasma-7_Cluster_2063_sequences=10	14	10
SDS-V3-plasma-7_Cluster_937_sequences=10	14	10
SDS-V3-plasma-7_Cluster_1084_sequences=10	14	10
SDS-V3-plasma-8_Cluster_243_sequences=10	16	10
SDS-V3-plasma-8_Cluster_16_sequences=10	16	10
SDS-V3-plasma-8_Cluster_73_sequences=10	16	10
SDS-V3-plasma-8_Cluster_233_sequences=10	16	10
SDS-V3-plasma-8_Cluster_664_sequences=10	16	10
SDS-V3-plasma-8_Cluster_81_sequences=10	16	10
SDS-V3-plasma-8_Cluster_5732_sequences=10	16	10
SDS-V3-plasma-8_Cluster_2278_sequences=10	16	10
SDS-V3-plasma-8_Cluster_3380_sequences=10	16	10
SDS-V3-plasma-8_Cluster_2463_sequences=10	16	10
SDS-V3-plasma-8_Cluster_6044_sequences=10	16	10
SDS-V3-plasma-8_Cluster_3951_sequences=10	16	10
SDS-V3-plasma-8_Cluster_3683_sequences=10	16	10
SDS-V3-plasma-8_Cluster_2821_sequences=10	16	10
SDS-V3-plasma-8_Cluster_3890_sequences=10	16	10
SDS-V3-plasma-8_Cluster_5203_sequences=10	16	10
SDS-V3-plasma-8_Cluster_2591_sequences=10	16	10
SDS-V3-plasma-8_Cluster_4101_sequences=10	16	10
SDS-V3-plasma-8_Cluster_4245_sequences=10	16	10
SDS-V3-plasma-8_Cluster_3245_sequences=10	16	10
SDS-V3-plasma-8_Cluster_3411_sequences=10	16	10
SDS-V3-plasma-8_Cluster_4230_sequences=10	16	10
SDS-V3-plasma-8_Cluster_5701_sequences=10	16	10
SDS-V3-plasma-8_Cluster_2357_sequences=10	16	10
SDS-V3-plasma-8_Cluster_3914_sequences=10	16	10
SDS-V3-plasma-8_Cluster_4064_sequences=10	16	10
SDS-V3-plasma-8_Cluster_2726_sequences=10	16	10
SDS-V3-plasma-8_Cluster_3137_sequences=10	16	10
SDS-V3-plasma-8_Cluster_4038_sequences=10	16	10
SDS-V3-plasma-8_Cluster_8929_sequences=10	16	10
SDS-V3-plasma-8_Cluster_4345_sequences=10	16	10
SDS-V3-plasma-8_Cluster_3398_sequences=10	16	10
SDS-V3-plasma-8_Cluster_211_sequences=10	16	10
SDS-V3-plasma-8_Cluster_4455_sequences=10	16	10
SDS-V3-plasma-8_Cluster_3120_sequences=10	16	10
SDS-V3-plasma-24_Cluster_1775_sequences=10	124	10
SDS-V3-plasma-24_Cluster_2323_sequences=10	124	10
SDS-V3-plasma-24_Cluster_366_sequences=10	124	10

SDS-V3-plasma-24_Cluster_3891_sequences=10	124	10
SDS-V3-plasma-24_Cluster_728_sequences=10	124	10
SDS-V3-plasma-24_Cluster_903_sequences=10	124	10
SDS-V3-plasma-24_Cluster_2175_sequences=10	124	10
SDS-V3-plasma-24_Cluster_1735_sequences=10	124	10
SDS-V3-plasma-24_Cluster_2290_sequences=10	124	10
SDS-V3-plasma-24_Cluster_3645_sequences=10	124	10
SDS-V3-plasma-24_Cluster_2051_sequences=10	124	10
SDS-V3-plasma-24_Cluster_4114_sequences=10	124	10
SDS-V3-plasma-24_Cluster_1364_sequences=10	124	10
SDS-V3-plasma-24_Cluster_4770_sequences=10	124	10
SDS-V3-plasma-24_Cluster_2152_sequences=10	124	10
SDS-V3-plasma-24_Cluster_3133_sequences=10	124	10
SDS-V3-plasma-24_Cluster_6033_sequences=10	124	10
SDS-V3-plasma-24_Cluster_3069_sequences=10	124	10
SDS-V3-plasma-24_Cluster_1772_sequences=10	124	10
SDS-V3-plasma-24_Cluster_1401_sequences=10	124	10
SDS-V3-plasma-24_Cluster_47_sequences=10	124	10
SDS-V3-plasma-24_Cluster_2396_sequences=10	124	10
SDS-V3-plasma-24_Cluster_7389_sequences=10	124	10
SDS-V3-plasma-24_Cluster_1934_sequences=10	124	10
SDS-V3-plasma-24_Cluster_531_sequences=10	124	10
SDS-V3-plasma-27_Cluster_1465_sequences=10	131	10
SDS-V3-plasma-27_Cluster_2436_sequences=10	131	10
SDS-V3-plasma-27_Cluster_4350_sequences=10	131	10
SDS-V3-plasma-27_Cluster_456_sequences=10	131	10
SDS-V3-plasma-27_Cluster_3023_sequences=10	131	10
SDS-V3-plasma-27_Cluster_1436_sequences=10	131	10
SDS-V3-plasma-27_Cluster_420_sequences=10	131	10
SDS-V3-plasma-27_Cluster_1232_sequences=10	131	10
SDS-V3-plasma-27_Cluster_2000_sequences=10	131	10
SDS-V3-plasma-27_Cluster_1457_sequences=10	131	10
SDS-V3-plasma-27_Cluster_4183_sequences=10	131	10
SDS-V3-plasma-27_Cluster_4971_sequences=10	131	10
SDS-V3-plasma-27_Cluster_70_sequences=10	131	10
SDS-V3-plasma-27_Cluster_913_sequences=10	131	10
SDS-V3-plasma-27_Cluster_8484_sequences=10	131	10
SDS-V3-plasma-27_Cluster_5942_sequences=10	131	10
SDS-V3-plasma-45_Cluster_1258_sequences=10	282	10
SDS-V3-plasma-45_Cluster_24503_sequences=10	282	10
SDS-V3-plasma-45_Cluster_7708_sequences=10	282	10
SDS-V3-plasma-45_Cluster_34204_sequences=10	282	10
SDS-V3-plasma-45_Cluster_8075_sequences=10	282	10
SDS-V3-plasma-45_Cluster_9283_sequences=10	282	10
SDS-V3-plasma-45_Cluster_19409_sequences=10	282	10

SDS-V3-plasma-45_Cluster_17469_sequences=10	282	10
SDS-V3-plasma-45_Cluster_42303_sequences=10	282	10
SDS-V3-plasma-45_Cluster_15271_sequences=10	282	10
SDS-V3-plasma-45_Cluster_2618_sequences=10	282	10
SDS-V3-plasma-45_Cluster_31853_sequences=10	282	10
SDS-V3-plasma-45_Cluster_5788_sequences=10	282	10
SDS-V3-plasma-45_Cluster_17085_sequences=10	282	10
SDS-V3-plasma-45_Cluster_1883_sequences=10	282	10
SDS-V3-plasma-45_Cluster_12434_sequences=10	282	10
SDS-V3-plasma-45_Cluster_1678_sequences=10	282	10
SDS-V3-plasma-45_Cluster_9786_sequences=10	282	10
SDS-V3-plasma-45_Cluster_25232_sequences=10	282	10
SDS-V3-plasma-45_Cluster_25634_sequences=10	282	10
SDS-V3-plasma-45_Cluster_2584_sequences=10	282	10
SDS-V3-plasma-45_Cluster_7876_sequences=10	282	10
SDS-V3-plasma-45_Cluster_1652_sequences=10	282	10
SDS-V3-plasma-45_Cluster_23535_sequences=10	282	10
SDS-V3-plasma-45_Cluster_9918_sequences=10	282	10
SDS-V3-plasma-45_Cluster_12558_sequences=10	282	10
SDS-V3-plasma-45_Cluster_5074_sequences=10	282	10
SDS-V3-plasma-45_Cluster_20629_sequences=10	282	10
SDS-V3-plasma-45_Cluster_5447_sequences=10	282	10
SDS-V3-plasma-45_Cluster_28107_sequences=10	282	10
SDS-V3-plasma-45_Cluster_27175_sequences=10	282	10
SDS-V3-plasma-45_Cluster_25396_sequences=10	282	10
SDS-V3-plasma-45_Cluster_32114_sequences=10	282	10
SDS-V3-plasma-45_Cluster_21140_sequences=10	282	10
SDS-V3-plasma-45_Cluster_73951_sequences=10	282	10
SDS-V3-plasma-45_Cluster_83057_sequences=10	282	10
SDS-V3-plasma-45_Cluster_30179_sequences=10	282	10
SDS-V3-plasma-45_Cluster_16068_sequences=10	282	10
SDS-V3-plasma-45_Cluster_9271_sequences=10	282	10
SDS-V3-plasma-45_Cluster_16654_sequences=10	282	10
SDS-V3-plasma-45_Cluster_18772_sequences=10	282	10
SDS-V3-plasma-45_Cluster_18891_sequences=10	282	10
SDS-V3-plasma-45_Cluster_23835_sequences=10	282	10
SDS-V3-plasma-45_Cluster_25631_sequences=10	282	10
SDS-V3-plasma-45_Cluster_29078_sequences=10	282	10
SDS-V3-plasma-45_Cluster_33334_sequences=10	282	10
SDS-V3-plasma-45_Cluster_43227_sequences=10	282	10
SDS-V3-plasma-45_Cluster_7920_sequences=10	282	10
SDS-V3-plasma-45_Cluster_15230_sequences=10	282	10
SDS-V3-plasma-45_Cluster_13452_sequences=10	282	10
SDS-V3-plasma-45_Cluster_10521_sequences=10	282	10
SDS-V3-plasma-45_Cluster_12916_sequences=10	282	10

SDS-V3-plasma-45_Cluster_15437_sequences=10	282	10
SDS-V3-plasma-45_Cluster_16185_sequences=10	282	10
SDS-V3-plasma-45_Cluster_1920_sequences=10	282	10
SDS-V3-plasma-45_Cluster_20946_sequences=10	282	10
SDS-V3-plasma-45_Cluster_29708_sequences=10	282	10
SDS-V3-plasma-45_Cluster_34203_sequences=10	282	10
SDS-V3-plasma-45_Cluster_59867_sequences=10	282	10
SDS-V3-plasma-45_Cluster_72_sequences=10	282	10
SDS-V3-plasma-45_Cluster_8587_sequences=10	282	10
SDS-V3-plasma-45_Cluster_9925_sequences=10	282	10
SDS-V3-plasma-45_Cluster_9934_sequences=10	282	10
SDS-V3-plasma-45_Cluster_12765_sequences=10	282	10
SDS-V3-plasma-45_Cluster_57807_sequences=10	282	10
SDS-V3-plasma-45_Cluster_1666_sequences=10	282	10
SDS-V3-plasma-45_Cluster_11164_sequences=10	282	10
SDS-V3-plasma-45_Cluster_10105_sequences=10	282	10
SDS-V3-plasma-45_Cluster_1553_sequences=10	282	10
SDS-V3-plasma-45_Cluster_2940_sequences=10	282	10
SDS-V3-plasma-45_Cluster_33129_sequences=10	282	10
SDS-V3-plasma-45_Cluster_6521_sequences=10	282	10
SDS-V3-plasma-45_Cluster_24424_sequences=10	282	10
SDS-V3-plasma-45_Cluster_5537_sequences=10	282	10
SDS-V3-plasma-45_Cluster_9562_sequences=10	282	10
SDS-V3-plasma-45_Cluster_36113_sequences=10	282	10
SDS-V3-plasma-45_Cluster_5108_sequences=10	282	10
SDS-V3-plasma-45_Cluster_48721_sequences=10	282	10
SDS-V3-plasma-45_Cluster_1989_sequences=10	282	10
SDS-V3-plasma-45_Cluster_10300_sequences=10	282	10
SDS-V3-plasma-45_Cluster_12020_sequences=10	282	10
SDS-V3-plasma-45_Cluster_12081_sequences=10	282	10
SDS-V3-plasma-45_Cluster_10437_sequences=10	282	10
SDS-V3-plasma-45_Cluster_150_sequences=10	282	10
SDS-V3-plasma-45_Cluster_18387_sequences=10	282	10
SDS-V3-plasma-45_Cluster_21188_sequences=10	282	10
SDS-V3-plasma-45_Cluster_21521_sequences=10	282	10
SDS-V3-plasma-45_Cluster_5755_sequences=10	282	10
SDS-V3-plasma-45_Cluster_2600_sequences=10	282	10
SDS-V3-plasma-45_Cluster_4519_sequences=10	282	10
SDS-V3-plasma-45_Cluster_4869_sequences=10	282	10
SDS-V3-plasma-45_Cluster_8658_sequences=10	282	10
SDS-V3-plasma-45_Cluster_9507_sequences=10	282	10
SDS-V3-plasma-45_Cluster_6057_sequences=10	282	10
SDS-V3-plasma-45_Cluster_15608_sequences=10	282	10
SDS-V3-plasma-45_Cluster_25559_sequences=10	282	10
SDS-V3-plasma-45_Cluster_6812_sequences=10	282	10

SDS-V3-plasma-45_Cluster_31365_sequences=10	282	10
SDS-V3-plasma-45_Cluster_1165_sequences=10	282	10
SDS-V3-plasma-45_Cluster_7921_sequences=10	282	10
SDS-V3-plasma-45_Cluster_14729_sequences=10	282	10
SDS-V3-plasma-45_Cluster_13216_sequences=10	282	10
SDS-V3-plasma-45_Cluster_12866_sequences=10	282	10
SDS-V3-plasma-45_Cluster_842_sequences=10	282	10
SDS-V3-plasma-45_Cluster_8348_sequences=10	282	10
SDS-V3-plasma-45_Cluster_2708_sequences=10	282	10
SDS-V3-plasma-45_Cluster_31703_sequences=10	282	10
SDS-V3-plasma-45_Cluster_38383_sequences=10	282	10
SDS-V3-plasma-45_Cluster_28304_sequences=10	282	10
SDS-V3-plasma-45_Cluster_48012_sequences=10	282	10
SDS-V3-plasma-45_Cluster_19682_sequences=10	282	10
SDS-V3-plasma-45_Cluster_48542_sequences=10	282	10
SDS-V3-plasma-45_Cluster_8430_sequences=10	282	10
SDS-V3-plasma-45_Cluster_2350_sequences=10	282	10
SDS-V3-plasma-45_Cluster_15397_sequences=10	282	10
SDS-V3-plasma-45_Cluster_37626_sequences=10	282	10
SDS-V3-plasma-45_Cluster_17206_sequences=10	282	10
SDS-V3-plasma-45_Cluster_2806_sequences=10	282	10
SDS-V3-plasma-45_Cluster_8114_sequences=10	282	10
SDS-V3-plasma-45_Cluster_19303_sequences=10	282	10
SDS-V3-plasma-45_Cluster_16874_sequences=10	282	10
SDS-V3-plasma-45_Cluster_16799_sequences=10	282	10
SDS-V3-plasma-45_Cluster_15414_sequences=10	282	10
SDS-V3-plasma-45_Cluster_1581_sequences=10	282	10
SDS-V3-plasma-45_Cluster_19874_sequences=10	282	10
SDS-V3-plasma-45_Cluster_16607_sequences=10	282	10
SDS-V3-plasma-45_Cluster_30827_sequences=10	282	10
SDS-V3-plasma-45_Cluster_16308_sequences=10	282	10
SDS-V3-plasma-45_Cluster_12792_sequences=10	282	10
SDS-V3-plasma-45_Cluster_18170_sequences=10	282	10
SDS-V3-plasma-45_Cluster_2549_sequences=10	282	10
SDS-V3-plasma-45_Cluster_30343_sequences=10	282	10
SDS-V3-plasma-45_Cluster_1533_sequences=10	282	10
SDS-V3-plasma-45_Cluster_65822_sequences=10	282	10
SDS-V3-plasma-45_Cluster_3660_sequences=10	282	10
SDS-V3-plasma-45_Cluster_19108_sequences=10	282	10
SDS-V3-plasma-45_Cluster_17374_sequences=10	282	10
SDS-V3-plasma-45_Cluster_10901_sequences=10	282	10
SDS-V3-plasma-45_Cluster_7744_sequences=10	282	10
SDS-V3-plasma-45_Cluster_7612_sequences=10	282	10
SDS-V3-plasma-45_Cluster_3355_sequences=10	282	10
SDS-V3-plasma-45_Cluster_66700_sequences=10	282	10

SDS-V3-plasma-45_Cluster_2110_sequences=10	282	10
SDS-V3-plasma-45_Cluster_5106_sequences=10	282	10
SDS-V3-plasma-45_Cluster_10258_sequences=10	282	10
SDS-V3-plasma-45_Cluster_25001_sequences=10	282	10
SDS-V3-plasma-45_Cluster_8432_sequences=10	282	10
SDS-V3-plasma-45_Cluster_813_sequences=10	282	10
SDS-V3-plasma-45_Cluster_43977_sequences=10	282	10
SDS-V3-plasma-45_Cluster_10201_sequences=10	282	10
SDS-V3-plasma-45_Cluster_33335_sequences=10	282	10
SDS-V3-plasma-45_Cluster_3430_sequences=10	282	10
SDS-V3-plasma-45_Cluster_1039_sequences=10	282	10
SDS-V3-plasma-45_Cluster_14438_sequences=10	282	10
SDS-V3-plasma-45_Cluster_25023_sequences=10	282	10
SDS-V3-plasma-45_Cluster_2801_sequences=10	282	10
SDS-V3-plasma-45_Cluster_16699_sequences=10	282	10
SDS-V3-plasma-45_Cluster_25957_sequences=10	282	10
SDS-V3-plasma-45_Cluster_3907_sequences=10	282	10
SDS-V3-plasma-45_Cluster_69453_sequences=10	282	10
SDS-V3-plasma-45_Cluster_8801_sequences=10	282	10
SDS-V3-plasma-45_Cluster_1719_sequences=10	282	10
SDS-V3-plasma-45_Cluster_21178_sequences=10	282	10
SDS-V3-plasma-45_Cluster_11339_sequences=10	282	10
SDS-V3-plasma-45_Cluster_6738_sequences=10	282	10
SDS-V3-plasma-45_Cluster_4183_sequences=10	282	10
SDS-V3-plasma-45_Cluster_46704_sequences=10	282	10
SDS-V3-plasma-45_Cluster_5214_sequences=10	282	10
SDS-V3-plasma-45_Cluster_11332_sequences=10	282	10
SDS-V3-plasma-45_Cluster_10295_sequences=10	282	10
SDS-V3-plasma-45_Cluster_2990_sequences=10	282	10
SDS-V3-plasma-45_Cluster_18927_sequences=10	282	10
SDS-V3-plasma-45_Cluster_4791_sequences=10	282	10
SDS-V3-plasma-45_Cluster_4043_sequences=10	282	10
SDS-V3-plasma-45_Cluster_17662_sequences=10	282	10
SDS-V3-plasma-45_Cluster_22909_sequences=10	282	10
SDS-V3-plasma-45_Cluster_1912_sequences=10	282	10
SDS-V3-plasma-45_Cluster_28589_sequences=10	282	10
SDS-V3-plasma-45_Cluster_12403_sequences=10	282	10
SDS-V3-plasma-45_Cluster_468_sequences=10	282	10
SDS-V3-plasma-45_Cluster_3141_sequences=10	282	10
SDS-V3-plasma-45_Cluster_11350_sequences=10	282	10
SDS-V3-plasma-45_Cluster_20341_sequences=10	282	10
SDS-V3-plasma-45_Cluster_1163_sequences=10	282	10
SDS-V3-plasma-45_Cluster_18949_sequences=10	282	10
SDS-V3-plasma-45_Cluster_11269_sequences=10	282	10
SDS-V3-plasma-45_Cluster_12695_sequences=10	282	10

SDS-V3-plasma-45_Cluster_27009_sequences=10	282	10
SDS-V3-plasma-45_Cluster_9276_sequences=10	282	10
SDS-V3-plasma-45_Cluster_5219_sequences=10	282	10
SDS-V3-plasma-45_Cluster_3891_sequences=10	282	10
SDS-V3-plasma-45_Cluster_25534_sequences=10	282	10
SDS-V3-plasma-45_Cluster_29961_sequences=10	282	10
SDS-V3-plasma-45_Cluster_16323_sequences=10	282	10
SDS-V3-plasma-45_Cluster_14960_sequences=10	282	10
SDS-V3-plasma-45_Cluster_2195_sequences=10	282	10
SDS-V3-plasma-45_Cluster_2273_sequences=10	282	10
SDS-V3-plasma-45_Cluster_29065_sequences=10	282	10
SDS-V3-plasma-45_Cluster_30976_sequences=10	282	10
SDS-V3-plasma-45_Cluster_33648_sequences=10	282	10
SDS-V3-plasma-45_Cluster_42109_sequences=10	282	10
SDS-V3-plasma-45_Cluster_48673_sequences=10	282	10
SDS-V3-plasma-45_Cluster_53512_sequences=10	282	10
SDS-V3-plasma-45_Cluster_7496_sequences=10	282	10
SDS-V3-plasma-45_Cluster_9404_sequences=10	282	10
SDS-V3-plasma-45_Cluster_3827_sequences=10	282	10
SDS-V3-plasma-45_Cluster_26542_sequences=10	282	10
SDS-V3-plasma-45_Cluster_4309_sequences=10	282	10
SDS-V3-plasma-45_Cluster_15864_sequences=10	282	10
SDS-V3-plasma-45_Cluster_25514_sequences=10	282	10
SDS-V3-plasma-45_Cluster_995_sequences=10	282	10
SDS-V3-plasma-45_Cluster_9974_sequences=10	282	10
SDS-V3-plasma-45_Cluster_13534_sequences=10	282	10
SDS-V3-plasma-45_Cluster_3778_sequences=10	282	10
SDS-V3-plasma-45_Cluster_12625_sequences=10	282	10
SDS-V3-plasma-45_Cluster_2366_sequences=10	282	10
SDS-V3-plasma-45_Cluster_12183_sequences=10	282	10
SDS-V3-plasma-45_Cluster_12692_sequences=10	282	10
SDS-V3-plasma-45_Cluster_13940_sequences=10	282	10
SDS-V3-plasma-45_Cluster_38710_sequences=10	282	10
SDS-V3-plasma-45_Cluster_40393_sequences=10	282	10
SDS-V3-plasma-45_Cluster_4841_sequences=10	282	10
SDS-V3-plasma-45_Cluster_58919_sequences=10	282	10
SDS-V3-plasma-45_Cluster_31012_sequences=10	282	10
SDS-V3-plasma-45_Cluster_7636_sequences=10	282	10
SDS-V3-plasma-45_Cluster_13644_sequences=10	282	10
SDS-V3-plasma-45_Cluster_17038_sequences=10	282	10
SDS-V3-plasma-45_Cluster_21707_sequences=10	282	10
SDS-V3-plasma-45_Cluster_65241_sequences=10	282	10
SDS-V3-plasma-45_Cluster_5009_sequences=10	282	10
SDS-V3-plasma-45_Cluster_22491_sequences=10	282	10
SDS-V3-plasma-45_Cluster_2312_sequences=10	282	10

SDS-V3-plasma-45_Cluster_2586_sequences=10	282	10
SDS-V3-plasma-45_Cluster_4198_sequences=10	282	10
SDS-V3-plasma-45_Cluster_4427_sequences=10	282	10
SDS-V3-plasma-45_Cluster_69783_sequences=10	282	10
SDS-V3-plasma-45_Cluster_7015_sequences=10	282	10
SDS-V3-plasma-45_Cluster_7641_sequences=10	282	10
SDS-V3-plasma-45_Cluster_5441_sequences=10	282	10
SDS-V3-plasma-45_Cluster_9362_sequences=10	282	10
SDS-V3-plasma-45_Cluster_4021_sequences=10	282	10
SDS-V3-plasma-45_Cluster_10329_sequences=10	282	10
SDS-V3-plasma-45_Cluster_10673_sequences=10	282	10
SDS-V3-plasma-45_Cluster_16600_sequences=10	282	10
SDS-V3-plasma-45_Cluster_1808_sequences=10	282	10
SDS-V3-plasma-45_Cluster_34400_sequences=10	282	10
SDS-V3-plasma-45_Cluster_3624_sequences=10	282	10
SDS-V3-plasma-45_Cluster_5287_sequences=10	282	10
SDS-V3-plasma-45_Cluster_5956_sequences=10	282	10
SDS-V3-plasma-45_Cluster_9345_sequences=10	282	10
SDS-V3-plasma-45_Cluster_1881_sequences=10	282	10
SDS-V3-plasma-45_Cluster_28018_sequences=10	282	10
SDS-V3-plasma-46_Cluster_10405_sequences=10	286	10
SDS-V3-plasma-46_Cluster_209_sequences=10	286	10
SDS-V3-plasma-46_Cluster_4529_sequences=10	286	10
SDS-V3-plasma-46_Cluster_7249_sequences=10	286	10
SDS-V3-plasma-46_Cluster_4937_sequences=10	286	10
SDS-V3-plasma-46_Cluster_9487_sequences=10	286	10
SDS-V3-plasma-46_Cluster_2437_sequences=10	286	10
SDS-V3-plasma-46_Cluster_1496_sequences=10	286	10
SDS-V3-plasma-46_Cluster_2246_sequences=10	286	10
SDS-V3-plasma-46_Cluster_5870_sequences=10	286	10
SDS-V3-plasma-46_Cluster_10916_sequences=10	286	10
SDS-V3-plasma-46_Cluster_4404_sequences=10	286	10
SDS-V3-plasma-46_Cluster_4104_sequences=10	286	10
SDS-V3-plasma-46_Cluster_6251_sequences=10	286	10
SDS-V3-plasma-46_Cluster_12020_sequences=10	286	10
SDS-V3-plasma-46_Cluster_2415_sequences=10	286	10
SDS-V3-plasma-46_Cluster_1587_sequences=10	286	10
SDS-V3-plasma-46_Cluster_4526_sequences=10	286	10
SDS-V3-plasma-46_Cluster_4510_sequences=10	286	10
SDS-V3-plasma-46_Cluster_1466_sequences=10	286	10
SDS-V3-plasma-46_Cluster_5756_sequences=10	286	10
SDS-V3-plasma-46_Cluster_16500_sequences=10	286	10
SDS-V3-plasma-46_Cluster_1775_sequences=10	286	10
SDS-V3-plasma-46_Cluster_1937_sequences=10	286	10
SDS-V3-plasma-46_Cluster_2740_sequences=10	286	10

SDS-V3-plasma-46_Cluster_2851_sequences=10	286	10
SDS-V3-plasma-46_Cluster_2990_sequences=10	286	10
SDS-V3-plasma-46_Cluster_3618_sequences=10	286	10
SDS-V3-plasma-46_Cluster_3842_sequences=10	286	10
SDS-V3-plasma-46_Cluster_4714_sequences=10	286	10
SDS-V3-plasma-46_Cluster_7354_sequences=10	286	10
SDS-V3-plasma-46_Cluster_8733_sequences=10	286	10
SDS-V3-plasma-46_Cluster_4719_sequences=10	286	10
SDS-V3-plasma-46_Cluster_3353_sequences=10	286	10
SDS-V3-plasma-46_Cluster_16565_sequences=10	286	10
SDS-V3-plasma-46_Cluster_5674_sequences=10	286	10
SDS-V3-plasma-46_Cluster_874_sequences=10	286	10
SDS-V3-plasma-46_Cluster_10401_sequences=10	286	10
SDS-V3-plasma-46_Cluster_13674_sequences=10	286	10
SDS-V3-plasma-46_Cluster_1608_sequences=10	286	10
SDS-V3-plasma-46_Cluster_3391_sequences=10	286	10
SDS-V3-plasma-46_Cluster_3900_sequences=10	286	10
SDS-V3-plasma-46_Cluster_5194_sequences=10	286	10
SDS-V3-plasma-46_Cluster_850_sequences=10	286	10
SDS-V3-plasma-46_Cluster_100_sequences=10	286	10
SDS-V3-plasma-46_Cluster_10776_sequences=10	286	10
SDS-V3-plasma-46_Cluster_32_sequences=10	286	10
SDS-V3-plasma-46_Cluster_7065_sequences=10	286	10
SDS-V3-plasma-46_Cluster_4397_sequences=10	286	10
SDS-V3-plasma-46_Cluster_4930_sequences=10	286	10
SDS-V3-plasma-46_Cluster_4780_sequences=10	286	10
SDS-V3-plasma-46_Cluster_14047_sequences=10	286	10
SDS-V3-plasma-46_Cluster_5949_sequences=10	286	10
SDS-V3-plasma-46_Cluster_587_sequences=10	286	10
SDS-V3-plasma-46_Cluster_4262_sequences=10	286	10
SDS-V3-plasma-46_Cluster_3681_sequences=10	286	10
SDS-V3-plasma-46_Cluster_6564_sequences=10	286	10
SDS-V3-plasma-46_Cluster_158_sequences=10	286	10
SDS-V3-plasma-46_Cluster_2026_sequences=10	286	10
SDS-V3-plasma-46_Cluster_2354_sequences=10	286	10
SDS-V3-plasma-46_Cluster_2488_sequences=10	286	10
SDS-V3-plasma-46_Cluster_3081_sequences=10	286	10
SDS-V3-plasma-46_Cluster_344_sequences=10	286	10
SDS-V3-plasma-46_Cluster_4881_sequences=10	286	10
SDS-V3-plasma-46_Cluster_6698_sequences=10	286	10
SDS-V3-plasma-46_Cluster_7059_sequences=10	286	10
SDS-V3-plasma-46_Cluster_7363_sequences=10	286	10
SDS-V3-plasma-46_Cluster_7478_sequences=10	286	10
SDS-V3-plasma-46_Cluster_871_sequences=10	286	10
SDS-V3-plasma-46_Cluster_1763_sequences=10	286	10

SDS-V3-plasma-46_Cluster_254_sequences=10	286	10
SDS-V3-plasma-46_Cluster_4524_sequences=10	286	10
SDS-V3-plasma-46_Cluster_4878_sequences=10	286	10
SDS-V3-plasma-46_Cluster_8915_sequences=10	286	10
SDS-V3-plasma-46_Cluster_3631_sequences=10	286	10
SDS-V3-plasma-46_Cluster_1768_sequences=10	286	10
SDS-V3-plasma-46_Cluster_14788_sequences=10	286	10
SDS-V3-plasma-46_Cluster_7275_sequences=10	286	10
SDS-V3-plasma-46_Cluster_1870_sequences=10	286	10
SDS-V3-plasma-46_Cluster_5945_sequences=10	286	10
SDS-V3-plasma-46_Cluster_1041_sequences=10	286	10
SDS-V3-plasma-46_Cluster_71_sequences=10	286	10
SDS-V3-plasma-46_Cluster_7209_sequences=10	286	10
SDS-V3-plasma-46_Cluster_3167_sequences=10	286	10
SDS-V3-plasma-46_Cluster_12390_sequences=10	286	10
SDS-V3-plasma-46_Cluster_3732_sequences=10	286	10
SDS-V3-plasma-46_Cluster_4360_sequences=10	286	10
SDS-V3-plasma-46_Cluster_4830_sequences=10	286	10
SDS-V3-plasma-46_Cluster_7093_sequences=10	286	10
SDS-V3-plasma-46_Cluster_4551_sequences=10	286	10
SDS-V3-plasma-46_Cluster_7623_sequences=10	286	10
SDS-V3-plasma-46_Cluster_8638_sequences=10	286	10
SDS-V3-plasma-46_Cluster_1078_sequences=10	286	10
SDS-V3-plasma-46_Cluster_13944_sequences=10	286	10
SDS-V3-plasma-46_Cluster_7825_sequences=10	286	10
SDS-V3-plasma-46_Cluster_10276_sequences=10	286	10
SDS-V3-plasma-46_Cluster_17791_sequences=10	286	10
SDS-V3-plasma-46_Cluster_19198_sequences=10	286	10
SDS-V3-plasma-46_Cluster_719_sequences=10	286	10
SDS-V3-plasma-46_Cluster_7313_sequences=10	286	10
SDS-V3-plasma-46_Cluster_2955_sequences=10	286	10
SDS-V3-plasma-46_Cluster_2343_sequences=10	286	10
SDS-V3-plasma-46_Cluster_2788_sequences=10	286	10
SDS-V3-plasma-46_Cluster_2924_sequences=10	286	10
SDS-V3-plasma-46_Cluster_14719_sequences=10	286	10
SDS-V3-plasma-46_Cluster_10325_sequences=10	286	10
SDS-V3-plasma-46_Cluster_11774_sequences=10	286	10
SDS-V3-plasma-46_Cluster_2779_sequences=10	286	10
SDS-V3-plasma-46_Cluster_8934_sequences=10	286	10
SDS-V3-plasma-46_Cluster_60_sequences=10	286	10
SDS-V3-plasma-67_Cluster_1356_sequences=10	504	10
SDS-V3-plasma-67_Cluster_3385_sequences=10	504	10
SDS-V3-plasma-67_Cluster_38736_sequences=10	504	10
SDS-V3-plasma-67_Cluster_13283_sequences=10	504	10
SDS-V3-plasma-67_Cluster_21140_sequences=10	504	10

SDS-V3-plasma-67_Cluster_22288_sequences=10	504	10
SDS-V3-plasma-67_Cluster_11822_sequences=10	504	10
SDS-V3-plasma-67_Cluster_7724_sequences=10	504	10
SDS-V3-plasma-67_Cluster_49341_sequences=10	504	10
SDS-V3-plasma-67_Cluster_27833_sequences=10	504	10
SDS-V3-plasma-67_Cluster_4761_sequences=10	504	10
SDS-V3-plasma-67_Cluster_67414_sequences=10	504	10
SDS-V3-plasma-67_Cluster_18253_sequences=10	504	10
SDS-V3-plasma-67_Cluster_18975_sequences=10	504	10
SDS-V3-plasma-67_Cluster_33379_sequences=10	504	10
SDS-V3-plasma-67_Cluster_51301_sequences=10	504	10
SDS-V3-plasma-67_Cluster_592_sequences=10	504	10
SDS-V3-plasma-67_Cluster_8666_sequences=10	504	10
SDS-V3-plasma-67_Cluster_15939_sequences=10	504	10
SDS-V3-plasma-67_Cluster_7453_sequences=10	504	10
SDS-V3-plasma-67_Cluster_21170_sequences=10	504	10
SDS-V3-plasma-67_Cluster_2965_sequences=10	504	10
SDS-V3-plasma-67_Cluster_39227_sequences=10	504	10
SDS-V3-plasma-67_Cluster_7034_sequences=10	504	10
SDS-V3-plasma-67_Cluster_8072_sequences=10	504	10
SDS-V3-plasma-67_Cluster_12958_sequences=10	504	10
SDS-V3-plasma-67_Cluster_15656_sequences=10	504	10
SDS-V3-plasma-67_Cluster_3860_sequences=10	504	10
SDS-V3-plasma-67_Cluster_9195_sequences=10	504	10
SDS-V3-plasma-67_Cluster_12507_sequences=10	504	10
SDS-V3-plasma-67_Cluster_21488_sequences=10	504	10
SDS-V3-plasma-67_Cluster_18284_sequences=10	504	10
SDS-V3-plasma-67_Cluster_9830_sequences=10	504	10
SDS-V3-plasma-67_Cluster_34492_sequences=10	504	10
SDS-V3-plasma-67_Cluster_8496_sequences=10	504	10
SDS-V3-plasma-67_Cluster_19432_sequences=10	504	10
SDS-V3-plasma-67_Cluster_5107_sequences=10	504	10
SDS-V3-plasma-67_Cluster_21101_sequences=10	504	10
SDS-V3-plasma-67_Cluster_11062_sequences=10	504	10
SDS-V3-plasma-67_Cluster_20140_sequences=10	504	10
SDS-V3-plasma-67_Cluster_8671_sequences=10	504	10
SDS-V3-plasma-67_Cluster_25893_sequences=10	504	10
SDS-V3-plasma-67_Cluster_28944_sequences=10	504	10
SDS-V3-plasma-67_Cluster_42036_sequences=10	504	10
SDS-V3-plasma-67_Cluster_4692_sequences=10	504	10
SDS-V3-plasma-67_Cluster_15632_sequences=10	504	10
SDS-V3-plasma-67_Cluster_14809_sequences=10	504	10
SDS-V3-plasma-67_Cluster_15192_sequences=10	504	10
SDS-V3-plasma-67_Cluster_30318_sequences=10	504	10
SDS-V3-plasma-67_Cluster_19840_sequences=10	504	10

SDS-V3-plasma-67_Cluster_22847_sequences=10	504	10
SDS-V3-plasma-67_Cluster_29979_sequences=10	504	10
SDS-V3-plasma-67_Cluster_4105_sequences=10	504	10
SDS-V3-plasma-67_Cluster_5720_sequences=10	504	10
SDS-V3-plasma-67_Cluster_86078_sequences=10	504	10
SDS-V3-plasma-67_Cluster_8861_sequences=10	504	10
SDS-V3-plasma-67_Cluster_2633_sequences=10	504	10
SDS-V3-plasma-67_Cluster_31925_sequences=10	504	10
SDS-V3-plasma-67_Cluster_4839_sequences=10	504	10
SDS-V3-plasma-67_Cluster_9323_sequences=10	504	10
SDS-V3-plasma-67_Cluster_4751_sequences=10	504	10
SDS-V3-plasma-67_Cluster_39033_sequences=10	504	10
SDS-V3-plasma-67_Cluster_13045_sequences=10	504	10
SDS-V3-plasma-67_Cluster_9504_sequences=10	504	10
SDS-V3-plasma-67_Cluster_43212_sequences=10	504	10
SDS-V3-plasma-67_Cluster_64908_sequences=10	504	10
SDS-V3-plasma-67_Cluster_5930_sequences=10	504	10
SDS-V3-plasma-67_Cluster_18302_sequences=10	504	10
SDS-V3-plasma-67_Cluster_2635_sequences=10	504	10
SDS-V3-plasma-67_Cluster_35847_sequences=10	504	10
SDS-V3-plasma-67_Cluster_9304_sequences=10	504	10
SDS-V3-plasma-67_Cluster_2711_sequences=10	504	10
SDS-V3-plasma-67_Cluster_4213_sequences=10	504	10
SDS-V3-plasma-67_Cluster_7550_sequences=10	504	10
SDS-V3-plasma-67_Cluster_21044_sequences=10	504	10
SDS-V3-plasma-67_Cluster_4486_sequences=10	504	10
SDS-V3-plasma-67_Cluster_67068_sequences=10	504	10
SDS-V3-plasma-67_Cluster_19687_sequences=10	504	10
SDS-V3-plasma-67_Cluster_44146_sequences=10	504	10
SDS-V3-plasma-67_Cluster_3112_sequences=10	504	10
SDS-V3-plasma-67_Cluster_24291_sequences=10	504	10
SDS-V3-plasma-67_Cluster_31825_sequences=10	504	10
SDS-V3-plasma-67_Cluster_2864_sequences=10	504	10
SDS-V3-plasma-67_Cluster_29087_sequences=10	504	10
SDS-V3-plasma-67_Cluster_7762_sequences=10	504	10
SDS-V3-plasma-67_Cluster_28816_sequences=10	504	10
SDS-V3-plasma-67_Cluster_17138_sequences=10	504	10
SDS-V3-plasma-67_Cluster_25988_sequences=10	504	10
SDS-V3-plasma-67_Cluster_35454_sequences=10	504	10
SDS-V3-plasma-67_Cluster_8693_sequences=10	504	10
SDS-V3-plasma-67_Cluster_39536_sequences=10	504	10
SDS-V3-plasma-67_Cluster_32882_sequences=10	504	10
SDS-V3-plasma-67_Cluster_4068_sequences=10	504	10
SDS-V3-plasma-67_Cluster_6185_sequences=10	504	10
SDS-V3-plasma-67_Cluster_9698_sequences=10	504	10

SDS-V3-plasma-67_Cluster_22784_sequences=10	504	10
SDS-V3-plasma-67_Cluster_30969_sequences=10	504	10
SDS-V3-plasma-67_Cluster_5363_sequences=10	504	10
SDS-V3-plasma-67_Cluster_34623_sequences=10	504	10
SDS-V3-plasma-67_Cluster_55936_sequences=10	504	10
SDS-V3-plasma-67_Cluster_24489_sequences=10	504	10
SDS-V3-plasma-67_Cluster_28835_sequences=10	504	10
SDS-V3-plasma-67_Cluster_1939_sequences=10	504	10
SDS-V3-plasma-67_Cluster_871_sequences=10	504	10
SDS-V3-plasma-67_Cluster_64390_sequences=10	504	10
SDS-V3-plasma-67_Cluster_48109_sequences=10	504	10
SDS-V3-plasma-67_Cluster_7909_sequences=10	504	10
SDS-V3-plasma-67_Cluster_12637_sequences=10	504	10
SDS-V3-plasma-67_Cluster_14279_sequences=10	504	10
SDS-V3-plasma-67_Cluster_21980_sequences=10	504	10
SDS-V3-plasma-67_Cluster_4014_sequences=10	504	10
SDS-V3-plasma-67_Cluster_46289_sequences=10	504	10
SDS-V3-plasma-67_Cluster_6491_sequences=10	504	10
SDS-V3-plasma-67_Cluster_44944_sequences=10	504	10
SDS-V3-plasma-67_Cluster_13996_sequences=10	504	10
SDS-V3-plasma-67_Cluster_10082_sequences=10	504	10
SDS-V3-plasma-67_Cluster_11621_sequences=10	504	10
SDS-V3-plasma-67_Cluster_17758_sequences=10	504	10
SDS-V3-plasma-67_Cluster_218_sequences=10	504	10
SDS-V3-plasma-67_Cluster_22894_sequences=10	504	10
SDS-V3-plasma-67_Cluster_282_sequences=10	504	10
SDS-V3-plasma-67_Cluster_30555_sequences=10	504	10
SDS-V3-plasma-67_Cluster_9729_sequences=10	504	10
SDS-V3-plasma-67_Cluster_7413_sequences=10	504	10
SDS-V3-plasma-67_Cluster_5036_sequences=10	504	10
SDS-V3-plasma-67_Cluster_13733_sequences=10	504	10
SDS-V3-plasma-67_Cluster_7466_sequences=10	504	10
SDS-V3-plasma-67_Cluster_15478_sequences=10	504	10
SDS-V3-plasma-67_Cluster_4037_sequences=10	504	10
SDS-V3-plasma-67_Cluster_42390_sequences=10	504	10
SDS-V3-plasma-67_Cluster_14427_sequences=10	504	10
SDS-V3-plasma-67_Cluster_22538_sequences=10	504	10
SDS-V3-plasma-67_Cluster_6229_sequences=10	504	10
SDS-V3-plasma-67_Cluster_10823_sequences=10	504	10
SDS-V3-plasma-67_Cluster_9335_sequences=10	504	10
SDS-V3-plasma-67_Cluster_13703_sequences=10	504	10
SDS-V3-plasma-67_Cluster_34223_sequences=10	504	10
SDS-V3-plasma-67_Cluster_49687_sequences=10	504	10
SDS-V3-plasma-67_Cluster_24362_sequences=10	504	10
SDS-V3-plasma-67_Cluster_12413_sequences=10	504	10

SDS-V3-plasma-67_Cluster_3725_sequences=10	504	10
SDS-V3-plasma-67_Cluster_13718_sequences=10	504	10
SDS-V3-plasma-67_Cluster_56568_sequences=10	504	10
SDS-V3-plasma-67_Cluster_70573_sequences=10	504	10
SDS-V3-plasma-67_Cluster_3218_sequences=10	504	10
SDS-V3-plasma-67_Cluster_24803_sequences=10	504	10
SDS-V3-plasma-67_Cluster_11351_sequences=10	504	10
SDS-V3-plasma-67_Cluster_19367_sequences=10	504	10
SDS-V3-plasma-67_Cluster_26325_sequences=10	504	10
SDS-V3-plasma-67_Cluster_7366_sequences=10	504	10
SDS-V3-plasma-67_Cluster_7772_sequences=10	504	10
SDS-V3-plasma-67_Cluster_5026_sequences=10	504	10
SDS-V3-plasma-67_Cluster_18224_sequences=10	504	10
SDS-V3-plasma-67_Cluster_45165_sequences=10	504	10
SDS-V3-plasma-67_Cluster_5249_sequences=10	504	10
SDS-V3-plasma-67_Cluster_7444_sequences=10	504	10
SDS-V3-plasma-67_Cluster_80636_sequences=10	504	10
SDS-V3-plasma-67_Cluster_10713_sequences=10	504	10
SDS-V3-plasma-67_Cluster_27304_sequences=10	504	10
SDS-V3-plasma-67_Cluster_20614_sequences=10	504	10
SDS-V3-plasma-67_Cluster_24233_sequences=10	504	10
SDS-V3-plasma-67_Cluster_3922_sequences=10	504	10
SDS-V3-plasma-67_Cluster_70889_sequences=10	504	10
SDS-V3-plasma-67_Cluster_26839_sequences=10	504	10
SDS-V3-plasma-67_Cluster_11863_sequences=10	504	10
SDS-V3-plasma-67_Cluster_12944_sequences=10	504	10
SDS-V3-plasma-67_Cluster_13537_sequences=10	504	10
SDS-V3-plasma-67_Cluster_21236_sequences=10	504	10
SDS-V3-plasma-67_Cluster_28640_sequences=10	504	10
SDS-V3-plasma-67_Cluster_3872_sequences=10	504	10
SDS-V3-plasma-67_Cluster_59912_sequences=10	504	10
SDS-V3-plasma-67_Cluster_8190_sequences=10	504	10
SDS-V3-plasma-67_Cluster_43913_sequences=10	504	10
SDS-V3-plasma-67_Cluster_12025_sequences=10	504	10
SDS-V3-plasma-67_Cluster_14073_sequences=10	504	10
SDS-V3-plasma-67_Cluster_14817_sequences=10	504	10
SDS-V3-plasma-67_Cluster_22985_sequences=10	504	10
SDS-V3-plasma-67_Cluster_33455_sequences=10	504	10
SDS-V3-plasma-67_Cluster_35111_sequences=10	504	10
SDS-V3-plasma-67_Cluster_7408_sequences=10	504	10
SDS-V3-plasma-67_Cluster_8734_sequences=10	504	10
SDS-V3-plasma-67_Cluster_9602_sequences=10	504	10
SDS-V3-plasma-67_Cluster_10145_sequences=10	504	10
SDS-V3-plasma-67_Cluster_14532_sequences=10	504	10
SDS-V3-plasma-67_Cluster_1977_sequences=10	504	10

SDS-V3-plasma-67_Cluster_40319_sequences=10	504	10
SDS-V3-plasma-67_Cluster_72282_sequences=10	504	10
SDS-V3-plasma-67_Cluster_23185_sequences=10	504	10
SDS-V3-plasma-67_Cluster_15991_sequences=10	504	10
SDS-V3-plasma-67_Cluster_8792_sequences=10	504	10
SDS-V3-plasma-67_Cluster_12749_sequences=10	504	10
SDS-V3-plasma-67_Cluster_25433_sequences=10	504	10
SDS-V3-plasma-67_Cluster_8305_sequences=10	504	10
SDS-V3-plasma-67_Cluster_49052_sequences=10	504	10
SDS-V3-plasma-67_Cluster_18839_sequences=10	504	10
SDS-V3-plasma-67_Cluster_37501_sequences=10	504	10
SDS-V3-plasma-67_Cluster_13925_sequences=10	504	10
SDS-V3-plasma-67_Cluster_20389_sequences=10	504	10
SDS-V3-plasma-67_Cluster_4885_sequences=10	504	10
SDS-V3-plasma-67_Cluster_28068_sequences=10	504	10
SDS-V3-plasma-67_Cluster_130626_sequences=10	504	10
SDS-V3-plasma-67_Cluster_34298_sequences=10	504	10
SDS-V3-plasma-67_Cluster_28851_sequences=10	504	10
SDS-V3-plasma-67_Cluster_7521_sequences=10	504	10
SDS-V3-plasma-67_Cluster_4858_sequences=10	504	10
SDS-V3-PBMC-45_Cluster_23576_sequences=9	282	9
SDS-V3-PBMC-45_Cluster_23779_sequences=9	282	9
SDS-V3-plasma-46_Cluster_ATAACTTAT_sequences=8	286	8
SDS-V3-plasma-67_Cluster_TAAGTATTG_sequences=7	504	7
SDS-V3-plasma-46_Cluster_CGGTTTTAT_sequences=7	286	7
SDS-V3-plasma-46_Cluster_TAATTGCGT_sequences=7	286	7
SDS-V3-plasma-46_Cluster_TACAGGTCA_sequences=7	286	7
SDS-V3-plasma-46_Cluster_TGCGACGAG_sequences=7	286	7
SDS-V3-PBMC-45_Cluster_33596_sequences=6	282	6
SDS-V3-PBMC-45_Cluster_34009_sequences=6	282	6
SDS-V3-PBMC-45_Cluster_28267_sequences=6	282	6
SDS-V3-plasma-46_Cluster_AGTGGGCTT_sequences=6	286	6
SDS-V3-plasma-46_Cluster_GACGGTTTT_sequences=6	286	6
SDS-V3-plasma-46_Cluster_TGAATTTAC_sequences=6	286	6
SDS-V3-plasma-0_Cluster_ATAGGGCAG_sequences=5	0	5
SDS-V3-plasma-0_Cluster_GCAGTCACA_sequences=5	0	5
SDS-V3-plasma-0_Cluster_TAGCACGCG_sequences=5	0	5
SDS-V3-plasma-67_Cluster_TATAAATAC_sequences=5	504	5
SDS-V3-plasma-67_Cluster_TGATTCAGT_sequences=5	504	5
SDS-V3-plasma-46_Cluster_AGAAAAAAG_sequences=5	286	5
SDS-V3-plasma-46_Cluster_CAATGCCTC_sequences=5	286	5
SDS-V3-plasma-46_Cluster_GGTGCTCCA_sequences=5	286	5
SDS-V3-plasma-46_Cluster_GTGGTTGAA_sequences=5	286	5
SDS-V3-plasma-46_Cluster_TAGTTCCAT_sequences=5	286	5
SDS-V3-plasma-46_Cluster_TCCTTTATC_sequences=5	286	5

SDS-V3-plasma-46_Cluster_TTTAGTTAT_sequences=5	286	5
SDS-V3-plasma-45_Cluster_CCGTGTCTT_sequences=5	282	5

Patient JOR10

haplotype	number of reads	timepoint
JOR-V3-plasma-97_Cluster_8_sequences=8393	8393	622
JOR-V3-plasma-97_Cluster_32_sequences=134	134	622
JOR-V3-plasma-97_Cluster_20_sequences=117	117	622
JOR-V3-plasma-97_Cluster_35_sequences=98	98	622
JOR-V3-plasma-14_TCGTGGGTG_sequences=95	95	33
JOR-V3-plasma-97_Cluster_96_sequences=66	66	622
JOR-V3-plasma-97_Cluster_19_sequences=57	57	622
JOR-V3-plasma-97_Cluster_21_sequences=39	39	622
JOR-V3-plasma-97_Cluster_73_sequences=37	37	622
JOR-V3-PBMC-14_Cluster_31_sequences=27	27	33
JOR-V3-PBMC-14_Cluster_13706_sequences=26	26	33
JOR-V3-plasma-97_Cluster_222_sequences=19	19	622
JOR-V3-plasma-97_Cluster_36_sequences=19	19	622
JOR-V3-plasma-97_Cluster_44_sequences=18	18	622
JOR-V3-PBMC-14_Cluster_7758_sequences=17	17	33
JOR-V3-plasma-97_Cluster_91_sequences=16	16	622
JOR-V3-plasma-97_Cluster_175_sequences=15	15	622
JOR-V3-PBMC-14_Cluster_598_sequences=13	13	33
JOR-V3-plasma-97_Cluster_159_sequences=13	13	622
JOR-V3-PBMC-14_Cluster_317_sequences=12	12	33
JOR-V3-plasma-97_Cluster_42_sequences=12	12	622
JOR-V3-plasma-97_Cluster_TGTGGCGCG_sequences=11	11	622
JOR-V3-plasma-14_Cluster_TGCTGAGAT_sequences=11	11	33
JOR-V3-plasma-97_Cluster_161_sequences=11	11	622
JOR-V3-PBMC-14_Cluster_4941_sequences=10	10	33
JOR-V3-plasma-97_Cluster_30_sequences=10	10	622
JOR-V3-plasma-97_Cluster_234_sequences=10	10	622
JOR-V3-plasma-97_Cluster_203_sequences=10	10	622
JOR-V3-plasma-97_Cluster_261_sequences=10	10	622
JOR-V3-plasma-97_Cluster_60_sequences=10	10	622
JOR-V3-PBMC-14_Cluster_31532_sequences=9	9	33
JOR-V3-plasma-14_Cluster_TCATTCTCT_sequences=8	8	33
JOR-V3-PBMC-14_Cluster_2959_sequences=7	7	33
JOR-V3-plasma-97_Cluster_GGGGGTATG_sequences=7	7	622
JOR-V3-plasma-97_Cluster_GGTGGGGGA_sequences=7	7	622
JOR-V3-plasma-14_Cluster_AGAGCCCGG_sequences=7	7	33
JOR-V3-plasma-14_Cluster_CCGGGGCAA_sequences=7	7	33
JOR-V3-plasma-14_Cluster_GTCACGAGT_sequences=7	7	33
JOR-V3-plasma-14_Cluster_TTTCGTTTT_sequences=7	7	33
JOR-V3-plasma-97_Cluster_GTTGGTGCG_sequences=6	6	622
JOR-V3-plasma-97_Cluster_AGGGTGTGT_sequences=5	5	622
JOR-V3-plasma-97_Cluster_GCGTTGTGG_sequences=5	5	622
JOR-V3-plasma-97_Cluster_GTGCGTGTT_sequences=5	5	622

JOR-V3-plasma-14_Cluster_AAGGATTAA_sequences=5	5	33
JOR-V3-plasma-14_Cluster_CACGTCGAG_sequences=5	5	33
JOR-V3-plasma-14_Cluster_CTTTCCATT_sequences=5	5	33
JOR-V3-plasma-14_Cluster_GAATCAGAA_sequences=5	5	33
JOR-V3-PBMC-14_Cluster_32_sequences=5	5	33

Table S4. Composition of haplotypes per timepoint and number of reads across patients. Excel file.

Table S5. Intra-patient genetic distance.

Patient	Distance	SE
VBP2	0.063	0.008
PCG3	0.046	0.007
SDS4	0.063	0.008
JOR10	0.095	0.011
VBP2-subB	0.050	0.008
JOR10-subB	0.062	0.009

SE: Standard Error estimate

Table S6. Between virion (RNA) and provirus (DNA) sequences genetic distance.

Patient	Distance	SE
VBP2	0.16	0.02
PCG3	0.045	0.009
SDS4	0.07	0.01
JOR10	0.171	0.02
VBP2-subB	0.068	0.011
JOR10-subB	0.081	0.014
